# Conjoining cell reprogramming and mass spectrometry to identify the proteomic variations in the reprogrammed bladder cancer cells: finding cues of normalisation

**DOI:** 10.1186/s12885-026-15634-x

**Published:** 2026-02-06

**Authors:** Banu Iskender, Mehmet Sarihan, Bengi Su Rumeysa Barlak, Gurler Akpinar, Murat Kasap

**Affiliations:** Faculty of Medicine, Department of Medical Biology, Protein Research and Proteomics Laboratory, Kocaeli University, Umuttepe, Izmit, 41001 Kocaeli Türkiye

**Keywords:** Cancer cell reprogramming, Bladder cancer, Partial reprogramming, Proteomics, Pluripotency, Biomarker

## Abstract

**Background:**

Cancer cell reprogramming is a critical area of research that holds the power to transform malignancies into benign states while revealing key mechanisms of carcinogenesis. This study aimed to develop a more effective in vitro bladder cancer model using induced pluripotent stem cell technology and identify potential diagnostic and therapeutic biomarkers for bladder cancer.

**Methods:**

Sendai virus-based reprogramming was utilised to reprogram the bladder cancer cell line HTB-5. The reprogrammed cells were characterised by expressing pluripotency-associated markers, colony formation abilities, cell migration, and drug responses. LC-MS/MS reveals changes in protein composition among parental cancer cells, reprogrammed cancer cells, and normal uroepithelial cells.

**Results:**

Reprogrammed bladder cancer cells display the expression of pluripotency-associated markers and demonstrate altered behaviours, including cell migration and responses to anticancer therapies. The genome-wide regulation by Sendai-virus delivery of Yamanaka factors resulted in distinctive protein expression patterns in reprogrammed bladder cancer cells, indicative of the pluripotency as well as spontaneous differentiation. A total of 297 dysregulated proteins in bladder cancer cells were normalised upon reprogramming. We proposed 25 potential biomarker candidates for diagnostic and therapeutic purposes, of which 12 candidates were demonstrated for the first time at the protein level.

**Conclusions:**

Differentially regulated proteins in parental bladder cancer cells and reprogrammed bladder cancer cells highlighted the critical protein-protein interactions that indicate the normalisation process of the parental bladder cancer cells. These cues could be used to pinpoint the candidate proteins to optimise the controlled partial/full reprogramming, to discover the therapeutic potential of reprogramming and to propose clinically relevant biomarker candidates.

**Graphical Abstract:**

**Figure:**
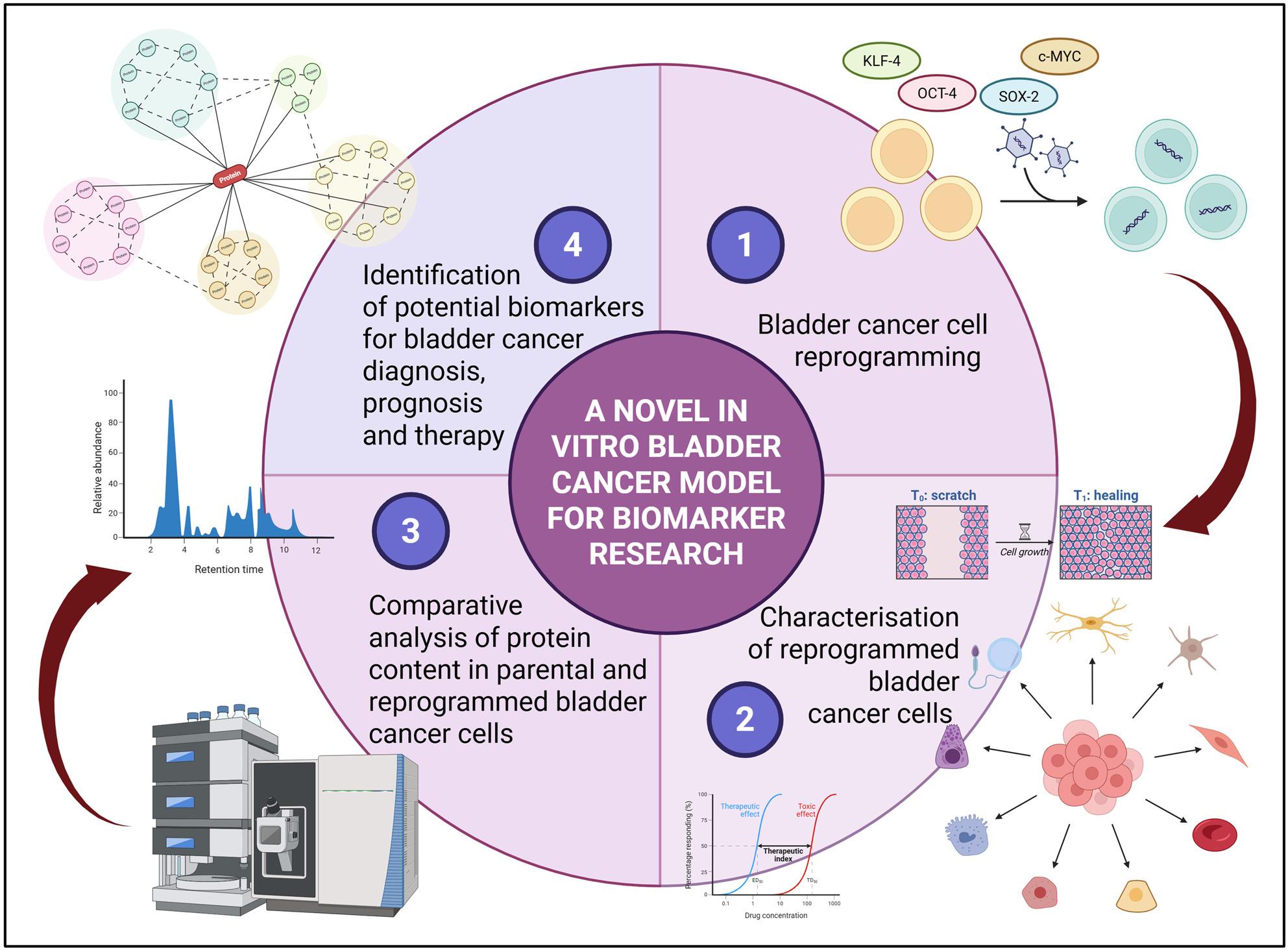
Cancer cell reprogramming highlights the early stages of bladder cancer, suggesting that the transient expression of pluripotency factors may serve as an initial step in the normalisation process of bladder cancer cells. Proteomic analysis of differentially expressed proteins in parental and reprogrammed cancer cells identifies potential biomarker candidates and paves the way for further exploration in future research. Created in BioRender. Iskender Izgi, B. (2025) https://BioRender.com/2aeyx7m

**Supplementary Information:**

The online version contains supplementary material available at 10.1186/s12885-026-15634-x.

## Introduction

Reprogramming of somatic cells leads the way to expand cell populations with the capacity to self-renew, which offers a huge promise for regenerative therapies. Reprogramming relies on core transcription factors Oct-4, Sox-2, Klf-4 and c-Myc (abbreviated here as OSKM) introduced to the cell using integrative or nonintegrating viral or nonviral systems [1, 2]. The ectopic expression of OSKM ideally produces pluripotent stem cells that are comparable to human embryonic stem cell counterparts. The acquisition of a pluripotent phenotype is inherently complex and never straightforward; it unfolds through a definitive, step-by-step process. Several models have been suggested for reprogramming, albeit the delivery techniques. In essence, reprogramming involves variable activation events including asynchronous gene activation, transcriptional bottlenecks and heterogeneous intermediate cell states whose number and nature have not been fully determined yet [3, 4].

Accumulating evidence suggests that reprogramming is at least a two-step process starting with the stochastic priming with the OSKM factors characterised by latency, transcriptional bottlenecks and intermediate states or partially reprogrammed cells followed by the second step of the deterministic phase where transition to a fully reprogrammed pluripotent state occurs [5]. OSKM components create an auto-regulatory loop that controls both their promoters and the promoters/enhancers of other genes involved in pluripotency and early differentiation [6]. Applying this process to cancer cells results in diverse, cancer-specific phenotypic and molecular consequences driven by the distinct interplay between the dysregulated epigenome of cancer cells and pluripotency induction pathways [7].

Cancer cells share a unique transcriptional signature with embryonic pluripotent stem cells, displaying high malignancy when less differentiated and exhibiting features of pluripotency [8, 9]. While reprogramming of somatic cells offers an unlimited source of undifferentiated cells that can be directed to differentiate towards all cell types in the human body, reprogrammed cancer cells pave the way to recapitulation of carcinogenesis, drug development and biomarker identification [10]. The efficiency of reprogramming cancer cells is significantly influenced by a disrupted epigenetic landscape, an impaired DNA damage response, genetic instability, and the onset of oncogene-induced senescence [7, 11, 12].

Cancer cells undergoing reprogramming often fail to achieve full pluripotency, instead adopting a *partially reprogrammed* state defined by increased cellular plasticity, transcriptional heterogeneity and hybrid phenotypes that combine stem-like and tumour-specific features [13]. Partial reprogramming either increases cellular stress responses, including apoptosis, senescence and genomic instability or enhances cell migration, invasion and metastatic properties in cancer cells [14, 15]. Several studies employed a non-integrative Sendai virus (SeV) for generating transgene-free induced Pluripotent Stem Cells (iPSCs), but detailed transcriptional, genetic and epigenetic changes underlying reprogramming remained poorly characterised [16]. Still, a stepwise approach addressing the mechanistic processes underlying pluripotency and the comparative analysis of the proteomic content of the resulting cell population remained underexplored. Previous studies addressing the resetting of the proteome during reprogramming have been hampered by the limited availability of protein-based reprogramming markers and poorly characterised intermediary cells during the multi-stage reprogramming process [2, 17]. Although dynamic changes in chromatin and metabolome remodelling and mesenchymal-to-epithelial transitions (MET) were identified as key processes during the reprogramming of normal cells, these studies focus on generic iPSC reprogramming of healthy cells [18].

Cancer cell reprogramming is an important yet underexplored topic. Currently, there is a notable gap in the literature regarding the specific proteomic changes induced by the Sendai virus in the reprogramming of cancer cells. This highlights a critical area for further research and exploration. In this study, we investigate the unique proteomic changes associated with Sendai virus-mediated reprogramming dynamics in bladder cancer cells. After we consider the changes both at the cellular and statistical levels, the degree of deviation from the normal uroepithelial cell state is quantified. We define “normalisation” in bladder cancer cells as restoring malignant phenotype toward a normal phenotype that is coupled with the approximation of the protein abundances to the values detected in normal bladder epithelial cells. Therefore, we aim to determine whether these cells can be reverted to a non-malignant or fully normal state and to define the extent of “normalisation” in bladder cancer cells following reprogramming, utilising the comparative analysis of functional units in parental and reprogrammed bladder cancer cells.

## Materials and methods

### Cell culture

Human grade 4 bladder cancer cell line HTB-5 (ATCC TCCSUP, RRID: CVCL_1738) and immortalised uroepithelial cell line SV-HUC-1 (ATCC CRL-9520, RRID: CVCL_3798**)** were obtained from the American Type Culture Collection (ATCC, USA). SV-HUC-1 cells are immortalised with simian virus 40 (SV40) large T antigen and are widely used as a normal urothelial control. Since primary urothelial cells have a limited lifespan and senesce after 5–7 passages, immortalised SV-HUC-1 cells represent stable and reproducible cells. Therefore, SV-HUC-1 cells were considered a baseline reference for the study [19].

All cell lines have been authenticated in the past 3 years, and all experiments were performed with mycoplasma-free cells (Certificate of Analysis for HTB-5 cells, Lot Number 70035661; Certificate of Analysis for SV-HUC-1 cells, Lot Number 70045213). HTB-5 cells were cultured in Minimum essential medium (Eagle) in Earle’s BSS with non-essential amino acids (MEM) (ATCC 30-2003) supplemented with 1 mM sodium pyruvate and 10% fetal bovine serum (FBS) at 37 °C in a 5% CO_2_ incubator. SV-HUC-1 cells were cultured with F-12 K Medium (ATCC 30-2004) supplemented with 10% FBS. After reprogramming, reprogrammed HTB-5 cells (HTB-5 PR) were maintained in Essential 8™ Medium (Gibco, A1517001) supplemented with 1% FBS and 4ng/ml basic Fibroblast Growth Factor (bFGF). Medium was changed every other day, and the cells were passaged using TrypLe Express (Invitrogen).

### Reprogramming bladder cancer cells

Bladder cancer cells were reprogrammed using an integration-free Sendai-virus-based system (CytoTune^®^-iPS 2.0 Sendai Reprogramming Kit (Thermo Fisher Scientific). Sendai virus particles carrying Yamanaka factors (human Oct3/4, Sox2, Klf4, c-Myc) were used to transduce HTB-5 cells. We conducted MOI optimisation using the CytoTune™ EmGFP Sendai Fluorescence Reporter to control the infection potential of the cell line and observed the cells up to 7 days to determine the ideal MOIs for transfection before reprogramming. Based on the GFP reporter assay and our previous cancer cell reprogramming experience [20], we used polycistronic KLF-4-OCT4-SOX2, monocistronic c-MYC and monocistronic KLF4 were used at a multiplicity of infection of 6:6:3, respectively. HTB-5 cells were counted and seeded at a density of 1 × 10^5^ cells, transfected with the determined MOIs and maintained in MEM medium post-transduction, which was changed with fresh medium every other day. At day 7, reprogrammed HTB-5 cells were transferred onto vitronectin-coated plates but kept in MEM media until the next day. At day 8 post-transduction, the medium was shifted to Essential 8™ Medium (Gibco, A1517001), and the medium was refreshed daily thereafter. Cells exhibiting morphology change and colony-formation were manually picked and expanded at 37 °C, 5% CO₂ in E8 medium, dissociated with TrypLe Express (Invitrogen) for 1 min at room temperature and resuspended with iPSC medium supplemented with ROCK inhibitor Y-27632 (Gibco).

### Immunofluorescence

Immunocytochemistry validated reprogramming using antibodies against reprogramming and pluripotency-related factors, including OCT-4, SOX-2, NANOG, c-MYC, KLF-4 and LIN28. Cells were fixed with 4% (*w*/*v*) paraformaldehyde for 10 min at room temperature which were then blocked in blocking solution consisting of Dulbecco’s phosphate-buffered saline (DPBS) with Ca2 + and Mg2 + and 5% Normal Goat Serum. All primary antibodies were obtained from Cell Signaling Technology and were used with the following dilution factors: NANOG (Nanog (D73G4) XP^®^ Rabbit mAb #4903) used in 1:50, OCT-4 A (Oct-4 A (C30A3) Rabbit mAb #2840) used in 1:100; SOX-2 (Sox2 (D6D9) XP^®^ Rabbit mAb #3579) used in 1:100, c-MYC (c-Myc (D84C12) Rabbit mAb #5605) used in 1:200, KLF4 (KLF4 Antibody #4038) used in 1:200 and LIN28A (LIN28A (D84C11) XP^®^ Rabbit mAb #3695) used in 1:200. Fluorescent-dye conjugated Alexa Fluor 488- and Alexa Fluor 594 (Invitrogen) were used as secondary antibodies. Cells were stained with DAPI (Abcam) and mounted using Mowiol before visualising using a fluorescence microscope (Olympus CKx41 microscope equipped with a DP74 digital camera system) equipped with x10, x20, x40 objective lenses and a digital camera.

### Proliferation assay

Cell proliferation was assessed using the Cell Counting Kit-8 (CCK-8 assay kit, ELabScience). Cells were seeded at a density of 6 × 10^3^ cells/well in 96-well plates. HTB-5 and HTB-5-PR cells were cultured in complete medium (RPMI and Essential 8, respectively), some wells were treated with or without increasing doses of doxorubicin (1, 2.5, 5, 10, 25, 50, 75 and100 µM) or paclitaxel (25, 50, 100, 250, 500, 750 and 1000nM) to test the drug effect on cell proliferation. All conditions were conducted in triplicate. At 36 h, 10 µl CCK-8 solution was applied to each well, incubated for 1 h at 37℃ and the optical densities were calculated by measuring absorbance at 450 nm.

### Wound healing assay

Control HTB-5 cells and HTB-5-PR cells were seeded in a 12-well plate and cultured to become a confluent monolayer. Wounds were formed with a sterile 200 µl tip, incubated in medium containing 1% FBS without or with increasing doses of paclitaxel (25, 250, 1000nM). Migration of cells toward the scratched area was monitored for 72 h and photographed by a phase-contrast microscope.

### Cell invasion assay

Invasion assays were performed using polycarbonate transwell inserts with 8.0 μm pore size coated with Geltrex. Both control HTB-5 cells and HTB-5-PR cells were seeded at a density of 2 × 10^5^ cells on the upper chamber in 0.2 ml serum-free RPMI medium. Complete medium supplemented with 10 µg/ml TGF-β was placed in the lower chamber and incubated for 24–36 h. The remaining cells in the upper chamber were removed, migrated cells in the lower chamber were fixed and stained with 0.1% crystal violet solution. The intensity of crystal violet was measured at 560 nm.

### Colony forming assay

Colony-forming ability of reprogrammed and parental bladder cancer cell lines was demonstrated by colony formation assay in the presence and absence of anticancer drugs. HTB-5 and HTB-5-PR cells were seeded at 6 × 10^3^ cells per well of a 6-well cell culture plate and allowed to grow for 10–20 days at 37 °C in a 5% CO_2_ incubator. To demonstrate the effects of anticancer drug doses on colony forming ability, cells were treated with different doses of doxorubicin (2.5, 5, 10, 25 and 50µ) and paclitaxel (5, 10, 50, 100 and 500nM), let to grow for 10–15 days, stained with 1% crystal violet solution and photographed. Semi-quantification was performed by measuring dissolved crystal violet intensity at 560 nm.

### Preparation of protein samples

For protein isolation, the growth medium was removed, and the cells were washed three times with ice-cold PBS. The cells were removed by scraping, washed with ice-cold PBS, transferred to 1.5 ml Eppendorf tubes and collected by centrifugation at 1500×*g* for 5 min at 4 °C. The supernatant was removed, and the pellet was resuspended in RIPA lysis buffer (Thermo Scientific, USA) supplemented with a protease inhibitor cocktail, then incubated on ice for 30 min. Cell disruption was performed using stainless steel beads in a Bullet Blender homogeniser (NextAdvance, USA). Cell-free extracts were clarified by centrifugation at 10,000×*g* for 10 min at 4 °C. Protein concentrations were determined using Bradford assay (Bio-Rad, USA) and protein qualities were assessed by SDS-PAGE analysis [21].

Protein pools were prepared by combining equal amounts of protein extracted from triplicate cell samples. For peptide pool preparation, 200 µg of protein from each protein pool was digested with trypsin using the filter-aided sample preparation (FASP) method, as described by Rençber et al. [22]. After tryptic digestion, the peptides were concentrated in a SpeedVac centrifuge (Eppendorf, USA), reconstituted in 30 µl of 0.1% formic acid. The peptide concentrations were determined using the Qubit Protein Assay Kit (Invitrogen) and stored at − 80 °C until nLC-MS/MS analysis.

For the preparation of a peptide sample for nLC-MS/MS analysis, the protein samples were calculated and then loaded to the SDS-PAGE gel to confirm the concentration and quality of proteins. The total intensities of the protein lanes were measured to normalise protein concentrations. Following tryptic digestion of equal amounts of protein, a second normalisation step is performed based on peptide concentrations to correct any variability introduced during sample processing. Then, equal amounts of peptides were loaded onto the LC-MS/MS system as three technical replicates. Finally, peptide and protein abundances are normalised using spectral peak intensities. These normalisation steps at each stage of the workflow aim to minimise variation and reduce the overall error rate in the analysis. To ensure consistency throughout the study, all samples were analysed without interruption. When differences in sample quantities, imbalances in spectral measurements, or abnormalities in chromatograms were detected, those samples were reanalysed to minimise instrument-related issues and maintain the overall reliability and accuracy of the data.

### Protein identification and label-free quantification by nHPLC LC–MS/MS

The LC–MS/MS analysis was performed using an Ultimate 3000 RSLC nanosystem (Dionex, Thermo Fisher Scientific, USA) coupled to a Q Exactive mass spectrometer (Thermo Fisher Scientific, USA). For each run 300 ng peptides were loaded onto the system and separated on a C18 reversed-phase analytical column (2 μm, 75 μm×250 mm Thermo Scientific) using mobile phases A (0.1% formic acid) and B (80% acetonitrile with 0.1% formic acid) at a flow rate of 300 nL/minutes. The chromatographic separation gradient of the peptides started at 6% of phase B for the first 8 min. Followed by a linear increase to 18% over the next 52 min. Phase B was then increased from 18% to 35% over 45 min, and subsequently ramped up to 90% over 20 min. The phase B was kept at 90% for 5 min. Then gradually decreased to 6% in 5 min and kept at 6% for 5 min in a total run time of 140 min.

The data-dependent spectra acquisition was carried out with the following parameters: resolution 70.000, scan range 250–2000 m/z, target automatic gain control (AGC) 3 × 10^6^, maximum injection time 100 ms, spray voltage 2.4 kV. MS/MS analysis was performed by data-dependent acquisition, selecting the top ten precursor ions. The MS2 analysis is composed of collision-induced dissociation (higher-energy collisional dissociation (HCD) with the following parameters: resolution 17,500, AGC 1E6; maximum injection time 100 ms, isolation window 2.0 m/z normalised and collision energy (NCE) 27.

### MS data analysis

Protein identification from raw MS/MS data was performed with Proteome Discoverer 2.2 SEQUEST software (Thermo Scientific, USA). Database searches were primarily conducted against the UniProt *Homo sapiens* reference proteome. In addition, targeted analyses were carried out separately for proteins associated with cell differentiation (Gene Ontology term: GO:0030154) and the extracellular matrix, to enhance sensitivity and obtain more focused insights relevant to induced IPSCs.

The following parameters were used for the analysis: peptide mass tolerance of 10 ppm, MS/MS mass tolerance of 0.2 Da, mass accuracy of 2 ppm, tolerant miscarriage of 1, minimum peptide length of 6 amino acids, fixed modification of carbamidomethylation of cysteines. The target false discovery rate (FDR) threshold was set as 0.01 (strict) and 0.05 (relaxed) using the Percolator node.

### Bioinformatic analysis of MS-MS data

For the evaluation of the biological functions of the regulated proteins, the STRING and g: Profiler analyses were employed using Gene Ontology (GO) functional classification annotation. All analysis were carried out using the Uniprot accession numbers after the organism was specified as *Homo Sapiens*. Regulated proteins were defined as those identified with more than two unique peptides (Unique peptide (UP) ≥ 2) and exhibiting a fold change (FC) greater than 2 or less than 0.5 in abundance levels compared with the expression observed in control SV-HUC-1 cells. These proteins were considered to display substantial alterations in expression, indicating potential biological regulation upon reprogramming. In contrast, unregulated proteins were defined as those whose expression values did not meet these fold change thresholds, exhibiting abundance levels comparable to those of the control SV-HUC-1 cells. Proteins with a 2-fold change (FC > 2 or < 0.5) in abundance values were considered as differentially expressed and used in an in-depth analysis. To enhance the robustness and reliability of protein identification, a more stringent filtering criterion was applied, retaining only proteins with more than five unique peptides (Unique peptide (UP) ≥ 5), while maintaining the same fold change thresholds (FC > 2 or FC < 0.5). We included these cut-off values to all abundance ratios for the subsequent relative quantification to reduce false positives before identifying protein-protein interactions through the Search Tool for the Retrieval of Interacting Genes/Proteins (STRING, https://string-db.org).

Protein–protein interaction (PPI) analyses were conducted using the STRING database to elucidate potential functional and physical associations among differentially expressed proteins (FC > 2 or < 0.5). The full STRING network analysis mode was employed, integrating interactions derived from multiple active evidence sources, including text mining, experimental data, curated databases, co-expression, gene neighbourhood, gene fusion, and co-occurrence. A medium confidence score threshold (interaction score ≥ 0.400) was applied to ensure reliable interaction mapping. The differentially expressed and exclusively expressed proteins in HTB-5 and HTB-5 PR cells were analysed separately. For assessing the group similarities, the heatmap clustering analysis was conducted using the heatmapper online tool (http://www.heatmapper.ca/expression/). Proteins exhibiting an absolute fold change (FC) greater than 2 or less than 0.5 were considered differentially expressed and included in the analysis. The data were clustered using the average-linkage method, and Euclidean distance was applied as the distance measurement metric. The presence of a Z-score scale on the generated heatmap confirmed that the dataset had been normalised before visualisation. This normalisation process was automatically performed by the Heatmapper tool following data upload [23].

### GEPIA analysis

In an attempt to identify whether these normalised proteins could be used as prognostic markers and/or therapeutic targets, we selected the differentially regulated proteins in HTB-5 cells under the functional annotations of extracellular matrix (GO:0031012), plasma membrane (GO:0005886) and cell junction (GO:0030054). We applied a strict regime of including at least 5 or more times regulated 25 proteins in these GO categories and verified our experimental results using GEPIA database (http://gepia.cancer-pku.cn/index.html). The expression profile of these proteins in 41 different cancer types was retrieved from GTEx and TCGA data. The differential expression analysis of the 25 proteins that are dysregulated but normalised upon reprogramming was conducted using GEPIA [24].

## Results

### SeV-based transduction leads to partial reprogramming of bladder cancer cells that gradually provoke cell differentiation

**Fig. 1:**
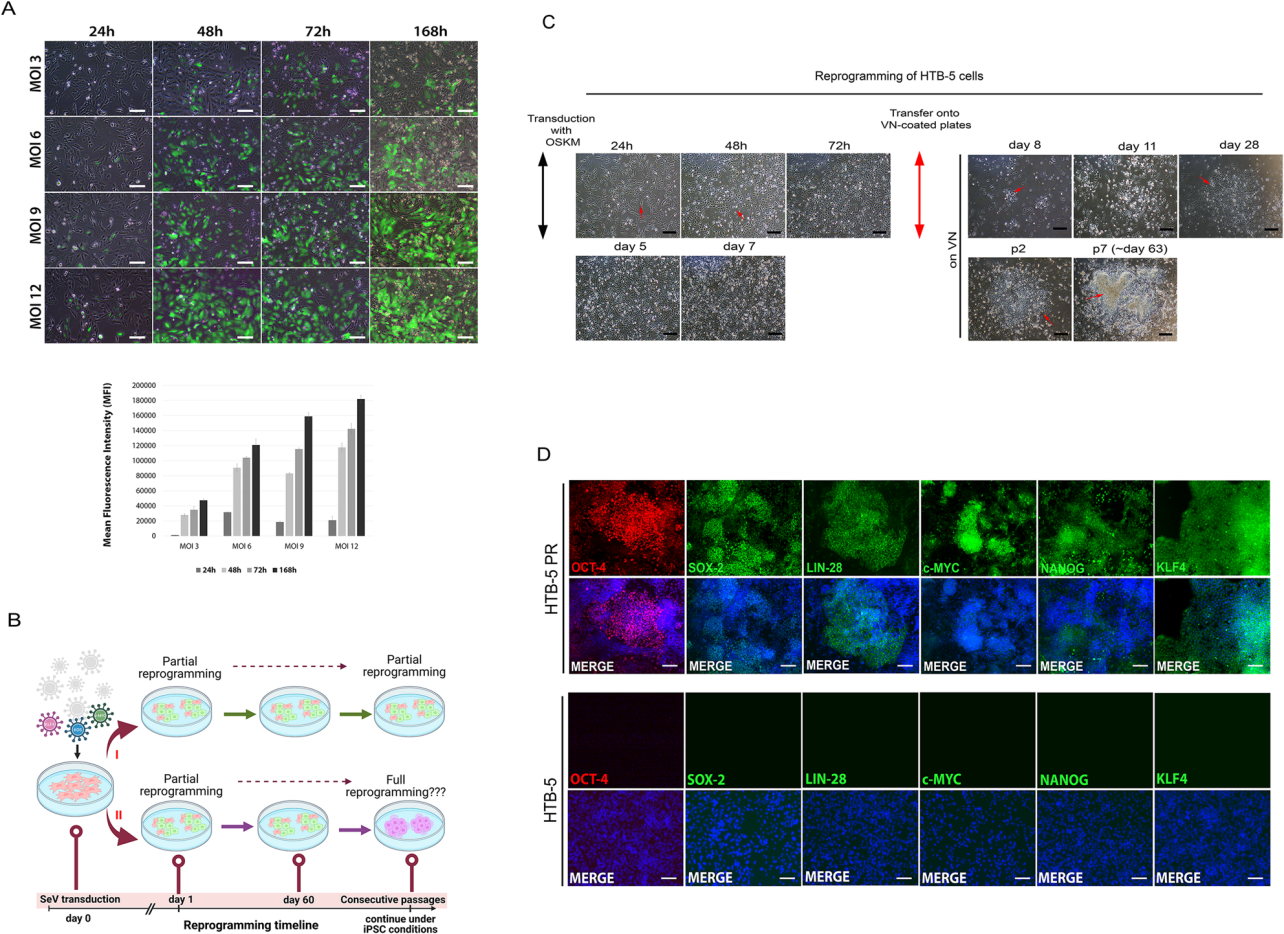
Reprogramming process of the bladder cancer cell line HTB-5. **A** Multiplicity of Infection (MOI) optimisation for Sendai virus was performed using EmGFP Sendai Fluorescence Reporter with ranging MOI concentrations from 3 to 12 and the cultures were monitored for 168 h. Mean fluorescence intensity was calculated using Image J and the change in intensities were shown as bar graphs. HTB-5 cells were compatible with all MOIs but MOI 6 was selected for further experiments. **B** A visual depiction of reprogramming procedure to create a distinct cell line from the parental bladder cancer cells. Two alternative routes the cells may choose to follow are indicated as partial and full reprogramming. Created in BioRender. Iskender Izgi, B. (2025) https://BioRender.com/qb3h9ze. **C** HTB-5 cells transfected with SeV-OSKM were monitored and photographed during the reprogramming stages. After transduction, HTB-5 were shown to lose its elongated, multi-sided structure and gained an epithelial-like morphology while forming colonies resembling iPSCs. **D** At passage 7, the reprogrammed HTB-5 cells were characterised for pluripotency-associated marker expression. The pluripotency markers OCT4, LIN28, SOX-2, c-MYC and NANOG were positively expressed in the reprogrammed HTB-5 cells and newly established cell line was referred as HTB-5 PR. **E** The parental HTB-5 cells failed to express pluripotency-associated markers. Scale bars represent 100 μm

To address whether bladder cancer cells are amenable to reprogramming and reprogramming-induced changes in cells, we used a nonintegrating Sendai virus-based system carrying the Yamanaka factors. GFP-expressing Sendai virus was used to track the inclusion status of the virus at 4 different MOIs (3, 6, 9 and 12). GFP expression was visualised from day 1 and gradually increased in culture depending on the MOI concentrations. We determined MOI 6 as the efficient and safer MOI to reduce possible virus-mediated toxicity, and HTB-5 cells transduced with SeV-OSKM (MOI 6:6:3) were monitored during the reprogramming (Fig. 1A).

We used HTB-5 cells without viral vectors as a mock control. Since the reprogrammed cells are vulnerable and costly to propagate in culture, we used a non-destructive qualitative analysis to predict the success of reprogramming. The morphological changes were evident in the reprogrammed HTB-5 cells, which abandoned their migratory phenotype and became rounder with scant cytoplasm and prominent nuclei. However, we noticed independent cells with no evident morphological change. We proposed two hypothetical routes that bladder cancer cells may have chosen during reprogramming (Fig. 1B). In the course of reprogramming, some colonies were detected as cracked colonies with some differentiation at the periphery. In contrast, some colonies were observed as built-up colonies in which the cells stacked on top of each other. These colonies were identified as moderately good and evaluated for pluripotency-associated marker expression (Fig. 1C).

After demonstrating positive marker expression for the master pluripotency regulators, we deemed the reprogrammed HTB-5 cells as induced pluripotent stem cell (iPS)-like cells and referred to the reprogrammed bladder cancer cell line as HTB-5 PR cells from here onwards (Fig. 1D-upper panel). Although the expression of pluripotency-associated markers was decreased upon continuous passaging in culture, the reprogrammed cells retain their functional characteristics indicative of spontaneous differentiation and divergence from parental cancer cells [25]. The parental bladder cancer cells were used as a control and did not show expression of pluripotency-associated markers at the protein level (Fig. 1D-lower panel).

### Reprogramming induced behavioural changes in rep HTB-5 cells

**Fig. 2:**
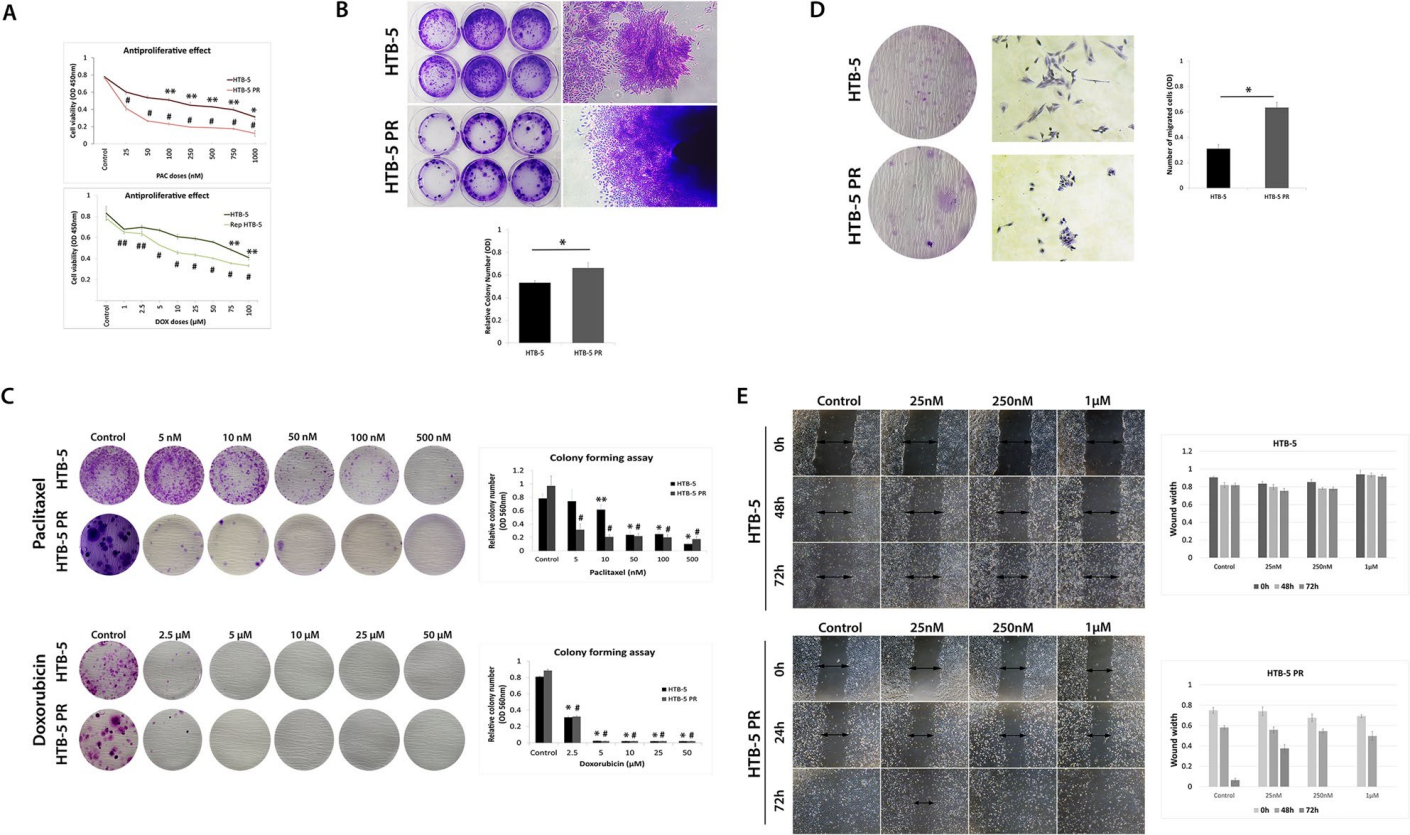
Behavioural assays with bladder cancer cells and reprogrammed bladder cancer cells. **A** Crystal violet staining of colonies of HTB-5 and HTB-5 PR. HTB-5 cells formed smaller and more colonies, while HTB-5 PR cells form fewer but larger colonies (± SD, *n* = 3, 2-tailed unpaired t-test, HTB-5 v HTB-5 PR, *P < 0.05) **B** Cell proliferation was assessed using the CCK-8 assay. HTB-5 and HTB-5 PR cells were treated with increasing doses of paclitaxel or with doxorubicin for 36 h. Paclitaxel inhibited the growth of both HTB-5 and HTB-5 PR cells in a dose-dependent manner (upper graph). Doxorubicin inhibited the growth of both HTB-5 and rep HTB-5 in a dose-dependent manner (lower graph). Data are presented as the means ± SD (error bars) from two independent experiments. (Mean ± SD, *n* = 3, 2-tailed unpaired t-test, HTB-5 v control; **p < 0.05, *p < 0.01. HTB-5 PR vs. control; ^##^*p* < 0.05, ^#^*p* < 0.01. CCK-8: Cell-Counting Kit-8; Pac: Paclitaxel, Dox: Doxorubicin; SD: standard deviation.) **C** A clonogenic assay was performed after exposing the cells to increasing doses of doxorubicin or paclitaxel. Colony forming ability was depicted as relative colony number at 560 nm (right). Mean ± SD, *n* = 3, 2-tailed unpaired t-test, HTB-5 v control; **p < 0.05, *p < 0.01. HTB-5 PR vs. control; ^##^*p* < 0.05, ^#^*p* < 0.01) **D** Effect of cancer cell reprogramming on transwell migration and invasion. The number of migrating HTB-5 PR cells was determined by quantifying the amount of crystal violet at an OD at 560 nm (± SD, *n* = 3, 2-tailed unpaired t-test, HTB-5 v HTB-5 PR, *p < 0.05) **E** Comparison of migration abilities by wound healing assay. HTB-5 and HTB-5 PR cells were treated with increasing doses of paclitaxel, and cell migration was observed at 24, 48 and 72 h. HTB-5 PR cells migrated to close the wound, while different doses of paclitaxel inhibited cell migration, increased cell death as observed in parental HTB-5 cells. The wound widths are depicted as bar graphs (lower panel)

We investigated how reprogramming affects cancer cell growth, proliferation, migration and invasion for behavioural characterisation of iPSC-like bladder cancer cells. For evaluating the clonal expansion, the colony-forming assay was conducted. Both cell lines showed the ability to form a large colony from a single cell. However, the colony size and number changed remarkably. HTB-5 PR cells formed bigger but fewer colonies, while parental HTB-5 cells built smaller but more colonies. Semi-quantitative analysis of colony numbers indicated a significant difference between the cell groups (Fig. 2A).

The role of the overexpression of pluripotency factors in cancer cells is considered to increase cellular vulnerability to therapeutic agents, but some studies suggest otherwise [26, 27]. Therefore, we next examined the effects of anticancer drugs on parental and reprogrammed HTB-5 cells. Increasing doses of doxorubicin (1, 2.5, 5, 10, 25, 50, 75 and 100µM) and paclitaxel (25, 50, 100, 250, 500, 750 and 1000 nM) were used in both cell lines and cell viability was assessed using CCK-8 after 36 h. The CCK-8 assay showed that HTB-5 PR cells acquired sensitivity to both doxorubicin and paclitaxel to a greater degree than parental cancer cells (Fig. 2B). Colony formation after drug treatment was also affected in both parental and reprogrammed bladder cancer cells. After doxorubicin treatment, both cell lines exhibited a higher sensitivity and failed to form colonies. HTB-5 PR cells became more sensitive to paclitaxel treatment, and even with the lowest doses (2.5 and 5nM), the number of colonies formed was scarce. However, the parental HTB-5 cells were more resistant to paclitaxel treatment at the lowest doses, consistent with the proliferation assay results (Fig. 2C).

We next sought to determine reprogrammed bladder cancer cells’ migratory and invasive abilities. Cell invasion requires cell adhesion, degradation of the extracellular matrix and migration through a membrane. Invasion assay results demonstrate that the relative number of HTB-5 PR cells that invaded through the membrane was significantly higher than the parental HTB-5 cells. Although parental HTB-5 cells also invade through the membrane, the strongest invasion ability was observed in HTB-5 PR cells (*p* < 0.05). These results suggest that both directed movement of cells on a 2D surface and infiltration through the matrix were enhanced in HTB-5 PR cells (Fig. 2D). These results are not surprising since one of the reprogramming factors, Klf4, is known to promote cell invasion and migration [28]. Previous studies also showed an increase in iPSC tropism toward growth factors and tumour-associated specific growth factors [29].

Here, we also showed that HTB-5 PR cells are responsive to transforming growth factor-β1 (TGF-β1) stimulation and invade through the membrane. Scratch assay results indicate closure of the wound by 72 h in HTB-5 PR cells while parental HTB-5 cells failed to migrate in the same time course (Fig. 2E). Although the control HTB-5 cells were further monitored until 168 h, the cells could not achieve wound closure (data not shown). Together, these results demonstrate that OSKM induction of bladder cancer cells changed the morphological features but also the behavioural characteristics of the cells.

### Functional characterisation of differentially expressed proteins in HTB-5 cells upon reprogramming reflected as the normalisation of multiple proteins

**Fig. 3:**
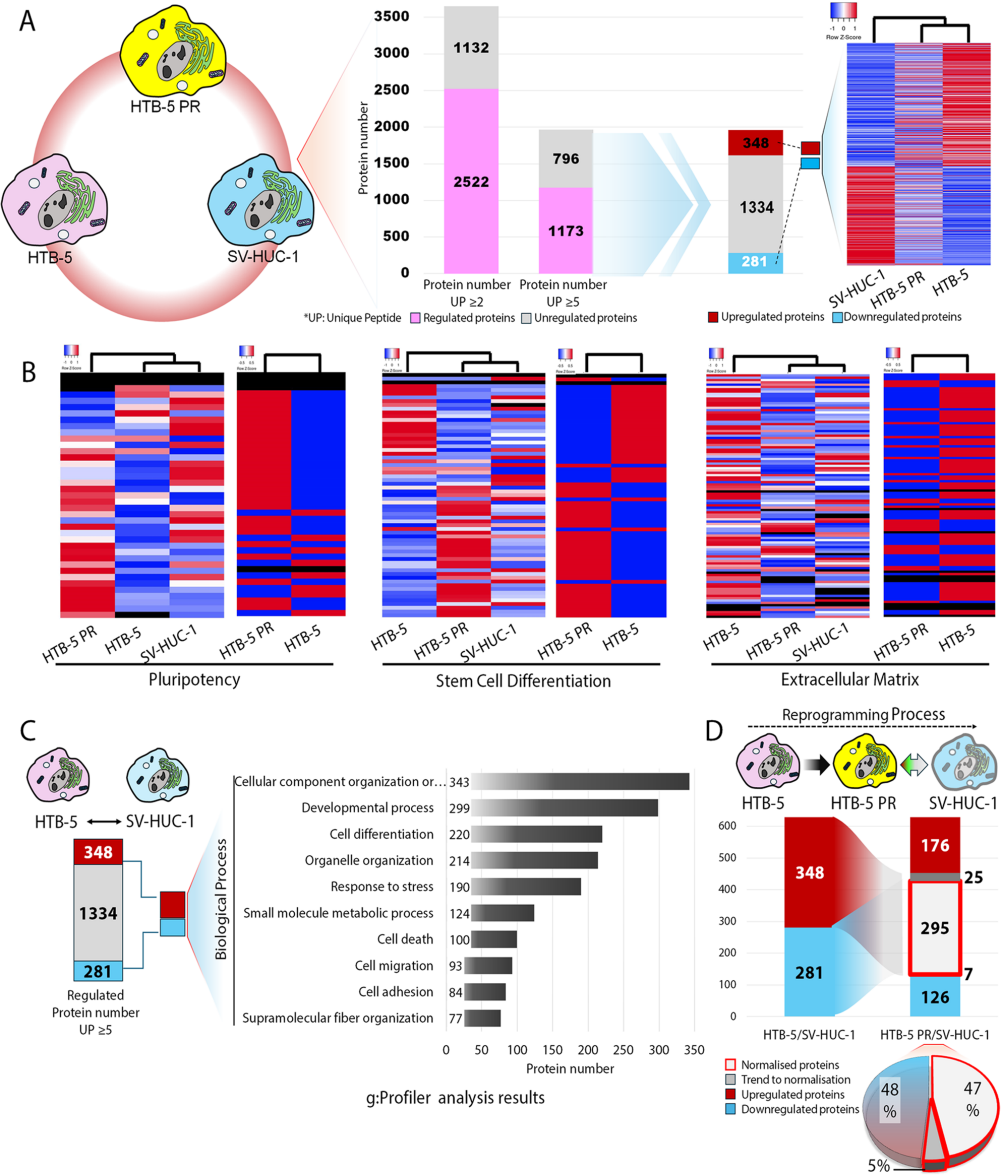
Comparative proteomic analysis of bladder cancer cell line HTB-5, reprogrammed bladder cancer cell line HTB-5-PR and human normal bladder epithelial cell line SV-HUC-1. **A** In total, 3654 proteins were identified with high confidence (false discovery rate < 1%) with at least two or more unique peptide fragments. Data analysis was enhanced by filtering the identified proteins to 1969 proteins that contain at least 5 or more unique peptides. In the hierarchical clustering of proteins with 2-fold change in abundance (FC > 2 or < 0.5), HTB-5 PR cells exhibit a transition state between normal epithelial cells and grade 4 bladder cancer cells. **B** Heat maps representing the regulated proteins involved in pluripotency, stem cell differentiation and extracellular matrix in each sample. The dendrogram depicts a separation of HTB-5-PR from HTB-5 and SV-HUC-1 cells in terms of pluripotency-associated protein expression. The cluster analysis was performed using Pearson correlation for the distance measure, protein intensities are shown using a colour scale ranging from red (high) to blue (low). The dendrogram above the heatmap shows the degree of relationship between protein profiles in the three samples. Red colour corresponds to higher z-scores. A z-score equivalent to 0 represents the mean abundance value for that protein. **C** During the reprogramming process, the abundance ratios of HTB-5/SV-HUC-1 were analysed against the expression levels of proteins in the normal bladder epithelial cell line. In the transition phase from grade 4 bladder cancer cell line toward reprogrammed HTB-5 PR cells, a variety of biological processes were shown to be regulated by g: Profiler analysis. **D** Out of 348 proteins were upregulated and out of 281 proteins downregulated, the expression levels of 295 proteins, which comprise 47% of the regulated proteins, were normalised in HTB-5 cells upon reprogramming

A total of 3443 proteins were identified: 3443 proteins in SV-HUC-1 cells, 3424 proteins in HTB-5 cells and 3400 proteins in rep HTB-5 cells, respectively. A systematic approach was applied to acquire proteins showing differential expression abundances to determine the changes in protein content after reprogramming and spontaneous differentiation of HTB-5 cells. 2522 regulated proteins were defined as those with ≥ 2 unique peptides and a fold change (FC) > 2 or < 0.5 relative to control SV-HUC-1 cells. Unregulated proteins (1132) showed expression levels within these thresholds and were comparable to controls. Proteins meeting the differential expression criteria (FC > 2 or < 0.5) were included in further analyses. A stricter filter (≥ 5 unique peptides) using the same FC thresholds was applied for increased reliability, which yielded 1173 regulated proteins. This approach enabled us to narrow down the number of proteins, resulting in a more confident inference of protein regulation patterns (Fig. 3A).

In global protein expression analysis, HTB-5 PR cells reflected a transition phase and shared similar protein expression profiles with both normal bladder epithelial cell line SV-HUC-1 and the parental HTB-5 cells (Fig. 3A). Distinctive expression patterns between the parental HTB-5 cells and HTB-5 PR cells were demonstrated in the proteins involved in pluripotency, stem cell differentiation and extracellular matrix. The hierarchical clustering analysis reveals the separation of the reprogrammed cell line HTB-5 PR from the parental bladder cancer cells as well as normal epithelial cells for the expression of pluripotency regulators. Not surprisingly, HTB-5 PR cells exhibited elevated expressions of proteins having a critical role in early developmental stages and regulating the pluripotency including but not limited to Fibroblast Growth Factor 2 (FGF2), Transcription Factor A, Mitochondrial (TFAM), Essential for Mitotic Growth 1 (EMG1), GTPase Activating Protein (SH3 Domain) Binding Protein 2 (G3BP2), DEAD-Box Helicase 21 (DDX21), LINE-1 Type Transposase Domain Containing 1 (L1TD1) and MutS Homolog 2 (MSH2) [30–36]. As an indication of normalisation, the HTB-5 PR cells and SV-HUC-1 cells were grouped for the proteins involved in stem cell differentiation and extracellular matrix as opposed to the parental bladder cancer cell line HTB-5 (Supporting Information 2, Fig. 3B). The common expression profile of these proteins may be attributed to their roles in maintaining epithelial characteristics and cellular reprogramming processes. For instance, Yes-Associated Protein 1 (YAP1) is involved in reprogramming and cellular plasticity, while Epithelial Cell Adhesion Molecule (EPCAM) is crucial for epithelial identity and is essential during the mesenchymal-to-epithelial transition (MET) in reprogramming [37, 38]. HTB-5 PR cells and SV-HUC-1 cells were also clustered together, indicating the deviation of HTB-5 PR cells from parental bladder cancer cells in terms of extracellular matrix content. The expressions of extracellular markers such as Laminin Subunit Beta 1 (LAMB1) and Fibronectin 1(FN1) reflect their integral roles in maintaining epithelial properties and facilitating the dynamic microenvironmental changes necessary during the reprogramming (Fig. 3A) [39–41].

The abundance ratios of HTB-5 PR/SV-HUC-1 were compared to the protein expression levels HTB-5/SV-HUC-1 during the reprogramming process. During the reprogramming process, out of the upregulated (348) and the downregulated (281) proteins, the expressions of 295 proteins were found to be normalised in the parental HTB-5 cells. 47% of the proteins containing at least 5 unique peptides in the reprogrammed bladder cancer cells were normalised, 5% were in a trend towards normalisation and 48% did not show differential expression. g: Profiler research demonstrated that several biological processes, including the developmental process, cell adhesion, and cell migration, were controlled during the transition phase from grade 4 bladder cancer cell line toward reprogrammed HTB-5 PR cells (Fig. 3C and D).

Since there are no proteomic studies directly comparing SV-HUC-1 cells and normal urothelial cells. Although there may be a shift in proteomic composition due to interference with core regulatory pathways in the cell cycle and DNA repair, SV-HUC-1 is expected to cluster closer to normal cells than cancer cell lines. Therefore, we considered SV-HUC-1 cells as a baseline reference for the study. Upon reprogramming in HTB-5 cells, among the proteins detected, 41% did not show any differential expression, and 47% were normalised either by upregulation or downregulation and became comparable to the abundances in the immortalised normal bladder epithelial cell line SV-HUC-1. This normalisation was not limited to over- or under-expressed proteins but was also detected in some proteins as a normalisation trend. We refer to the protein abundances detected outside the pre-determined cut-off values, but appear to continue to increase or decrease as opposed to the bladder cancer cells, as the expressions in the normalisation trend (Fig. 3D). Together, these data suggest that critical components in cellular identity change are highly regulated upon reprogramming of bladder cancer cells that may contribute to the normalisation of protein expression levels.

### The major biological processes represented in reprogrammed bladder cancer cells are associated with distinct processes feeding into the normalisation of bladder cancer cells

**Fig. 4:**
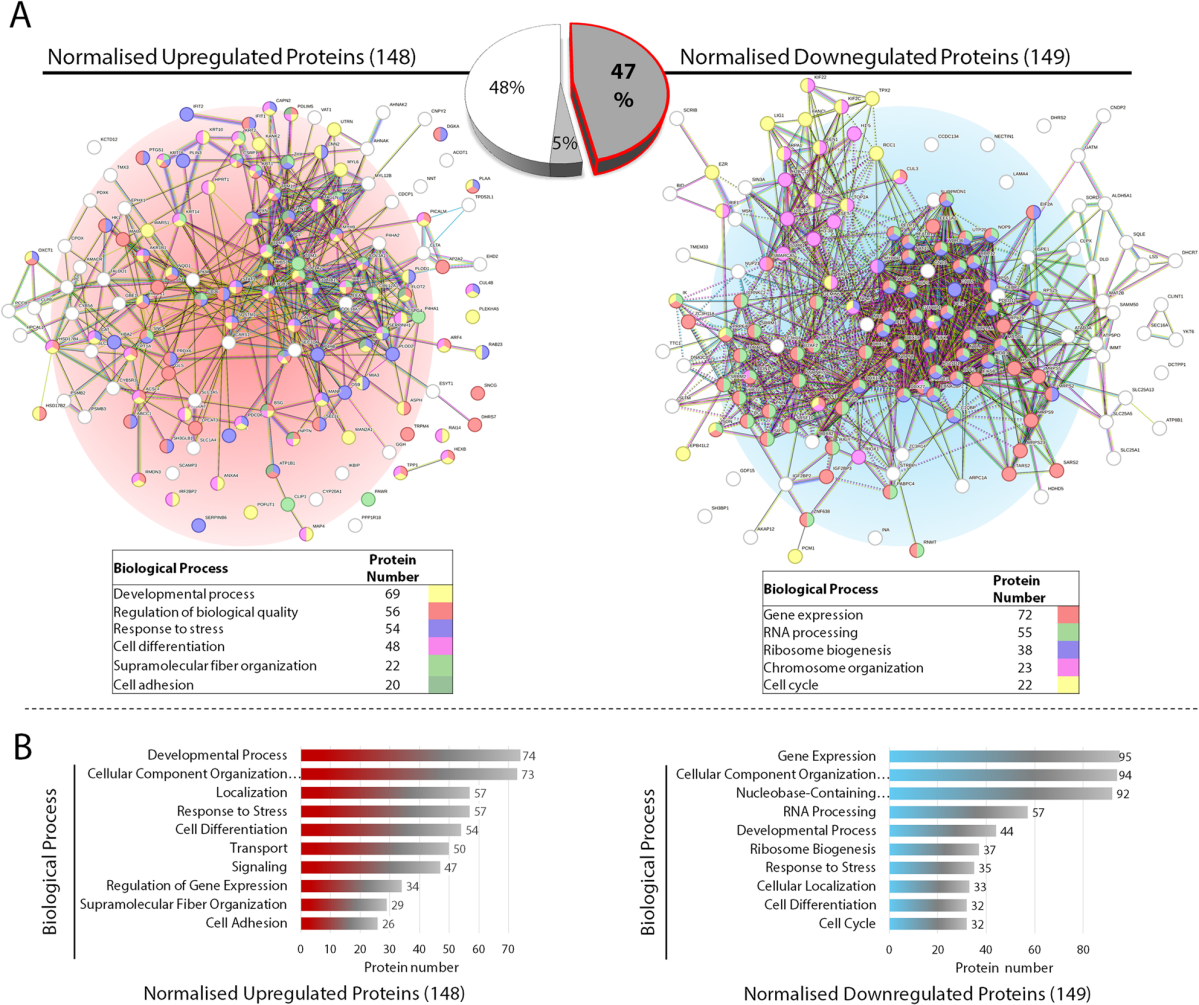
STRING network analysis of upregulated and downregulated proteins indicate progress toward normalisation in parental HTB-5 cells. Results of annotated keywords (by UniProt) obtained using functional enrichment analysis in STRING 12.5 (FDR < 0.05), indicating enriched categories and number of proteins mapped. After reprogramming, 47% of total proteins in parental HTB-5 cells that approximate the expression levels to those of the normal bladder epithelial cell line SV-HUC-1 cells were considered to be normalised and analysed for protein-protein interaction. **A** Protein-protein interactions of upregulated 149 proteins and downregulated 148 proteins (upper panel) and the proteins involved in the GO annotations showing the most clustering are presented at the bottom of each protein-protein interaction network (lower panel). Nodes represent proteins, lines between nodes represent edges showing interactions with a minimum confidence score of 0.7. Each GO annotation is indicated with a different colour, and the nodes are coloured depending on their involvement in certain biological processes. **B** g: Profiler analysis of upregulated and downregulated proteins during the reprogramming process. The graphs show the enriched terms, most of which are also highlighted in the STRING analysis. The ten most highly upregulated terms (lower left panel) and downregulated terms (lower right panel) are ranked by decreasing protein number detected in each term

Bioinformatics enrichment analyses were conducted using the Gene Ontology (GO) classification system. Protein groups were analysed in two groups: normalised upregulated proteins and normalised downregulated proteins after reprogramming (Fig. 4A and B). The STRING analysis of the upregulated proteins (148) in the category of biological process revealed “supramolecular fiber organisation,” “response to stress,” “developmental process” and “cell adhesion” as enriched ontologies upon reprogramming of HTB-5 cells. Proteins involved in response to stress and developmental process represent the most abundant clusters among the upregulated proteins (Fig. 4A-left panel). Since iPSC-like cells are substrate-selective and attachment-dependent and cytoskeletal remodelling is necessary for reprogramming, remodelling in the proteins involved in supramolecular fiber organisation and cell adhesion were not surprising [42]. Supramolecular fibril organisation (GO:0097435) include 16 subcategories that take part in the assembly and arrangement of polymerised fibrillar structures. Proteins upregulated in fiber organisation are involved in both cell-cell and cell-matrix adhesion. The content of the extracellular matrix not only affects the colony shape, being either dome-like or monolayer, but also defines cell spreading and the formation of cellular protrusions [43]. Consistent with the behavioural experiments, HTB-5 cells modulated cytoskeletal constituents and extracellular matrix constituents, gained migration and invasion ability upon reprogramming. Moderate fibronectin level is known to support mesenchymal-to-epithelial transition (MET) and contribute to E-cadherin stabilisation [44]. However, increased extracellular matrix (ECM) deposition is inhibitory for reprogramming, and cell fate conversion rates reduce accordingly [45]. Therefore, repression of excessive collagen and fibronectin fibril formation could have served to reduce ECM-mediated restraints after reprogramming.

STRING analysis of the downregulated proteins (149) after reprogramming were enriched in biological processes including “RNA processing”, “gene expression”, “ribosome biogenesis”, “Cell cycle” and “DNA recombination” (Fig. 4A-right panel). It is evident that ribosome biosynthesis is activated post-OSKM-induction, rRNA transcription, and ribonucleoprotein gene expression are critical for successful reprogramming [46, 47]. Moreover, one of the reprogramming factors, cMYC, is known to coordinate protein synthesis through transcriptional regulation of genes involved in rRNA processing, ribosomal biogenesis and protein translation [48]. Downregulation of these proteins could indicate a shift in translational control mechanisms during reprogramming of cancer cells. Indeed, downregulated ribosomal proteins and RNA processing factors usually lead to inhibition of cancer cell proliferation, an increase in therapeutic efficacy and apoptosis [49]. In support of the latter, we also showed that HTB-5 cells became more vulnerable to anticancer drugs and reduced proliferation after reprogramming. g: Profiler analysis of protein enrichments revealed a broader yet consistent network with the STRING database for similar biological processes, but with a higher number of detected proteins in each category (Fig. 4B).

### Proteins involved in pluripotency and stem cell differentiation propose partial reprogramming and spontaneous differentiation of HTB-5-PR cells

**Fig. 5:**
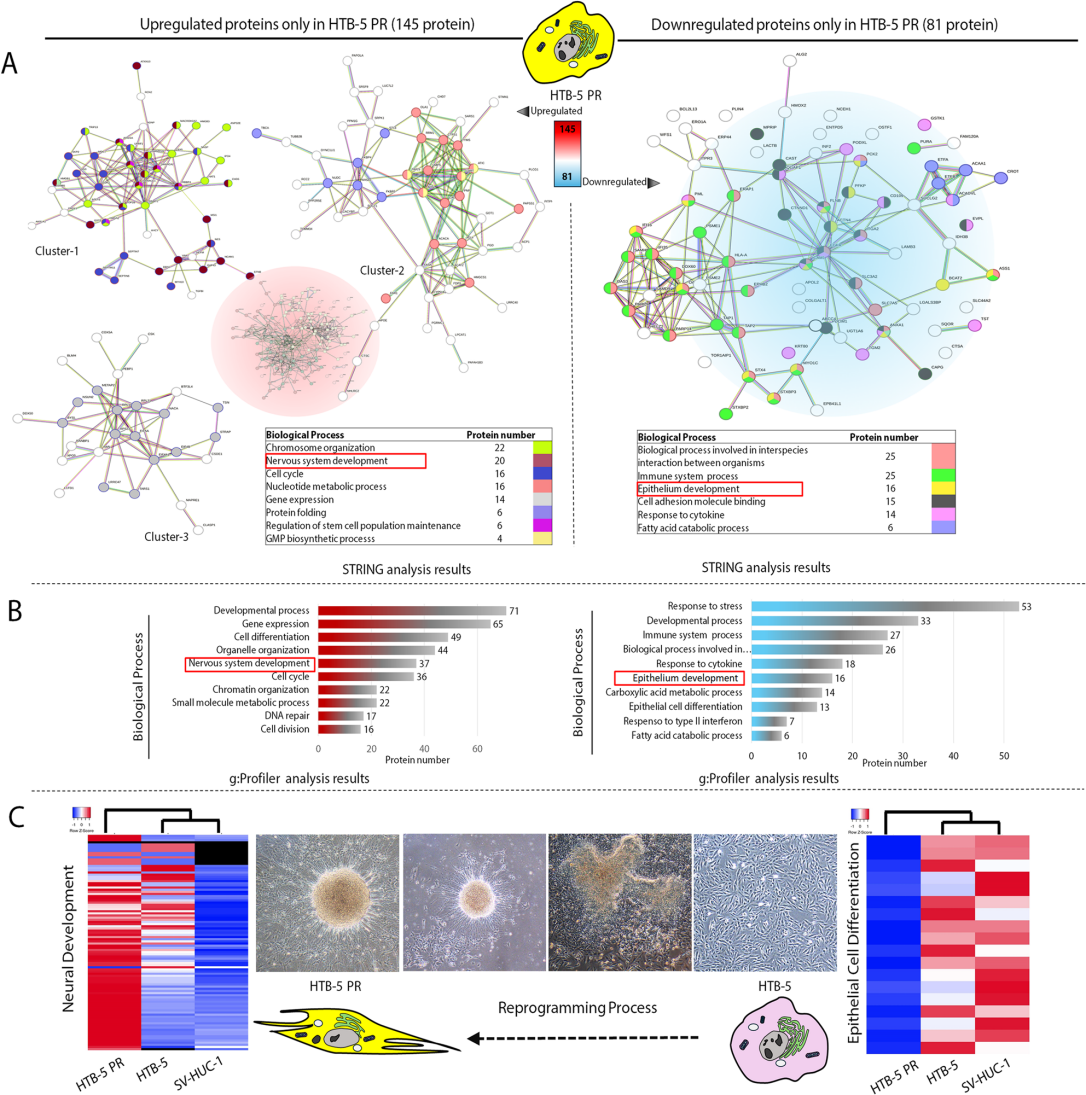
Functional enrichment analysis of reprogrammed HTB-5 PR cells. **A** The STRING database was used to construct the protein-protein interaction (PPI) networks of upregulated 145 proteins (left panel) and downregulated 85 proteins (right panel) in HTB-5 PR cells. Nodes represent proteins, and the lines between them depict interactions with a minimum confidence score of 0.7. **B** g: Profiler analysis of upregulated and downregulated proteins in the reprogrammed HTB-5 PR cells. The graph represents the GO enrichment analysis of upregulated proteins (left panel in shaded red) in the biological process and downregulated proteins (right panel in shaded blue) in the biological process. Ten highly upregulated and downregulated GO terms are ranked by the number of proteins detected in each term, listed in decreasing order. Spontaneous differentiation upon reprogramming leads to an increase in nervous system development markers and to a decrease in epithelium development markers (marked in blue) as detected by both STRING and g: Profiler analysis. **C** The clustering of differentially expressed proteins involved in neural development and epithelial cell differentiation is displayed in heatmaps. The parental HTB-5 cells and normal epithelial cell line SV-HUC-1 are grouped based on similar protein expression profiles showing downregulation of neuronal markers and upregulation of epithelial markers, while the reprogrammed bladder cancer cell line HTB-5 PR exhibits an opposite expression profile. The data is consistent with the morphological evidence documented during the reprogramming process (from right to left), showing the parental HTB-5 cells transform into pluripotent-like colonies and gain neuron-like trajectories as a result of spontaneous differentiation in time. The protein intensities are shown using a colour scale ranging from red (high) to blue (low). Red colour corresponds to higher z-scores. A z-score equivalent to 0 represents the mean abundance value for that protein

To further investigate the proteomic changes in HTB-5-PR cells, we performed functional analysis on differentially expressed proteins with a minimum of 2 identified unique peptides and having a 2-fold change in abundance (FC > 2 or < 0.5). We observed proteomic differences of reprogrammed bladder cancer cells, including 145 upregulated and 81 downregulated differentially expressed proteins (Fig. 5A). Gene Ontology (GO) analysis was performed separately on the upregulated and downregulated proteins which revealed the most prominent biological processes as cell cycle, nucleotide metabolic process, regulation of stem cell population maintenance, nervous system development for the upregulated proteins and immune system process, epithelium development, fatty acid catabolic process and cell adhesion molecule binding for the downregulated proteins in HTB-5-PR cells. During iPSC generation, nucleotide metabolism plays an essential role in supporting nucleotide biosynthesis and DNA repair in cells [50], while chromatin remodelling and chromosome organisation susceptible to gene expression favour reprogramming [51]. Upon reprogramming, elevated expression levels in these processes could support that reprogrammed cells are undergoing significant molecular changes indicative of the establishment of stem cell-like properties or a transformed phenotype. Both STRING and g: Profiler analysis confirmed the enrichment of similar biological processes with the upregulation of nervous system development and the downregulation of epithelium development (Supporting Information 3, Fig. 5A and B).

Interestingly, increased expression of proteins involved in both stem cell population maintenance and nervous system development after reprogramming could indicate the progression of reprogrammed cancer cells towards a neural progenitor state or neural differentiation. Pluripotent cells were previously shown to retain pluripotency but also tended to differentiate toward the neuronal lineage [52]. Therefore, we might speculate that transient activation of OSKM factors induces progression of bladder cancer cells toward the iPSC-like state in which the cells self-renew and differentiate into multiple lineages, including neuronal cells. Alternatively, the reprogrammed bladder cancer cells remain in a transitional state between pluripotency and neural differentiation, and some cells of this intermediary phase undergo a transdifferentiation or dedifferentiation process. Consistent with these, we obtained phase contrast images of late passages (passage number > 12–15) of HTB-5-PR cells, where among the heterogeneous population of reprogrammed cells, some acquired neuronal-like phenotype with long trajectories and formed interconnected groups resembling neural networks (Fig. 5C). This suggests the possibility of spontaneous differentiation toward neuronal lineage at the later stages of reprogramming. Indeed, we detected that HTB-5 PR exhibited differential expression of neural development and epithelial cell differentiation markers, which could be observed from hierarchical clustering analysis (Fig. 5C). HTB-5 PR cells upregulated proteins involved in neural development and downregulated proteins involved in epithelial cell differentiation and clustered separately from bladder cancer cells and normal bladder epithelial cells. Upon reprogramming differential expression of these proteins in the HTB-5 PR cells was a clear indication of normalisation independent from the choice of cellular differentiation after pluripotency.

Although we incorporated both biological and technical replicates, inherent limitations, including batch-to-batch variations in the sample preparation or masking of low-abundance proteins and incomplete quantification, remain. First, despite the repeated sampling, low-abundance proteins may be underrepresented due to the dynamic range of mass spectrometry. Second, biological replicates of one bladder cancer cell line cannot fully capture the tumour heterogeneity and the control immortalised cell line may diverge from primary urothelial cells in proteomic signature. Third, the use of a stringent threshold comprising unique peptide number and fold-change cut-offs may exclude the borderline signals and introduce bias toward well-characterised proteins. Nevertheless, our study is primarily a descriptive study introducing a novel technique for bladder cancer biomarker research; our focus was on providing a comprehensive proteomic overview rather than on mechanistic dissection. Furthermore, validation across the transcriptomic datasets via the GEPIA tool enhances the robustness and confidence of our data.

## Discussion

The key objective of the study is to acquire a snapshot of the proteomic landscape in the transition of bladder cancer cells through reprogramming and to present proteins involved in the dedifferentiation process. We proposed to direct the bladder cancer cells towards differentiation or a non-malignant state and identified the proteome of the resulting cell population. Here we tested partial reprogramming with transient expression of reprogramming factors followed by spontaneous differentiation, thereby avoiding full iPSC reprogramming that bears the inherent risk of tumorigenicity [26, 53]. Studies on SeV-mediated reprogramming of cancer cells predominantly rely on transcriptomics and epigenetics that provide limited mechanistic insights into protein-level changes upon reprogramming [12, 54, 55]. This is the first study to conduct the global protein expression analysis of reprogrammed bladder cancer cells. Previously, only one study provided evidence for differentially expressed proteins in reprogrammed breast cancer cells versus non-reprogrammed cells and revealed substantial proteomic regulation in critical pathways [56]. However, no literature exists on the actual proteomic signature underpinning the cancer fate reversal. Here, we aimed to determine how reprogramming influences the protein content of bladder cancer cells and to provide an empirical, protein-level dataset for cancer cell normalisation upon reprogramming.

The generation of iPSCs from cancer cells is an innovative and powerful tool for studying tumorigenesis, drug resistance, intratumoral heterogeneity and reversing malignancy into a more benign phenotype [57, 58]. To date reprogramming of cancer cells have been reported in multiple cancer types including colorectal [59], leukemia [60, 61], melanoma [62], glioblastoma [63], gastrointestinal [12], liver [64], pancreas [54], lung [27] and neurofibroma [65]. We previously reprogrammed bladder cancer cell line T24 successfully and demonstrated functional changes in certain signalling pathways at the protein level [20]. Although some of these studies provide evidence for genetic and epigenetic differences after reprogramming, studies examining the proteome upon reprogramming in cancer cells are lacking. Here, we showed that bladder cancer cell line HTB-5 is also amenable to reprogramming, and can be reversed to a pluripotent state, differentiate and normalise the expression levels of multiple proteins.

Reprogramming cancer cells is challenged by variable reprogramming efficiency arising from patient and tumour heterogeneity, tumour-associated DNA methylation and epigenetic barriers [11, 66]. Cancer cells usually suffer from incomplete reprogramming due to epigenetic abnormalities, including DNA methylation and histone modifications at tumour suppressor loci [67]. We may speculate that this might be the reason underlying the partial reprogramming of HTB-5 cells since transient expression of OSKM was not stabilised in our experimental setting. Still, behavioural characteristics and the total number of normalised proteins provided concrete results since our final aim was to characterise the resulting cell population rather than directed differentiation toward a certain lineage.

The acquisition of the malignant phenotype does not solely depend on the combination of reprogramming factors but also the genetic and epigenetic resetting of the starting cell population. Indeed, increasing evidence suggests that cancer cells attain epigenetic changes during cancer initiation and tumour development [68]. In line with the concept of dominant epigenetic regulation in the conversion of cell identity, studies showed evidence for resetting the epigenetic profile of pancreatic ductal adenocarcinoma and colorectal cancer cells, resulting in partial reprogramming, low tumorigenicity and decreased tumour aggressiveness [53, 55]. Previous reports have proposed that three-factor (Oct-4-Sox2-Klf4) or four-factor (Oct-4-Sox2-Klf4-c-Myc) reprogramming could transiently generate cancer cells retaining pluripotent stem cell characteristics in hepatocellular carcinoma and colon cancer cells [25, 69]. Cancer stem cell markers in reprogrammed gastrointestinal cancer cells were markedly decreased after reprogramming but increased after spontaneous differentiation of iPSC-like cells toward postiPC cells, which were contradictingly also more sensitive to anticancer drugs (12). Consistent with the latter, although Oct-4, Sox2, Klf4 and c-Myc are reported to be associated with malignancy and poor prognosis in various cancers, we also demonstrated that the forced overexpression of these factors increased the chemotherapeutic response in HTB-5 PR cells.

In the last part, we reasoned how the reversal of differentially expressed proteins before and after reprogramming of bladder cancer cells or *the normalisation process* could benefit us in the practical applications and performed an in-depth analysis for the prospective biomarkers. Bladder cancer (BCa) is a diverse disease that poses significant challenges in diagnosis, treatment, and prognosis. Current tools, including invasive procedures like cystoscopy and biopsy, along with imaging techniques, often miss understaged patients and micrometastases [70]. Although multi-omic studies provide candidate prognostic and therapeutic biomarkers for bladder cancer, most of these markers suffer from lack of multicenter human validation [70, 71]. Due to the disease’s heterogeneous nature, it is not ideal to characterise and define the treatment options relying on a single biomarker. Therefore, *omic* approaches represent a great potential for diagnosis and personalising the disease models and treatment strategies [72]. The current study not only developed an in vitro model system for biomarker exploratory studies by recapitulation of bladder carcinogenesis but also defined the future prospects for biomarker development. The idea of bladder cancer cell reversal to the pluripotent-like state, followed by spontaneous differentiation, delineates the normalisation of multiple functional units in the HTB-5 PR cells. Mass spectrometry revealed that the proteins with dysregulated expression levels in the parental HTB-5 cell line approached normal expression levels upon reprogramming. Here we identified 25 candidate proteins in the category of cellular component, including the extracellular matrix, plasma membrane and cell junction with prognostic/therapeutic value in bladder carcinoma. We showed that most of these prospective biomarker candidates represent consistent expression profiles with the previous literature (Supporting Information 4). Out of these, 12 proteins were characterised at the protein level for the first time by our study, most of which were also consistent with the previous genomic data (Table 1). Still, there is a pressing need to explore additional candidate molecules for biomarker development and validate these candidate biomarkers across disease stages and diverse populations to generate clinically relevant tools [73].

**Table 1:**
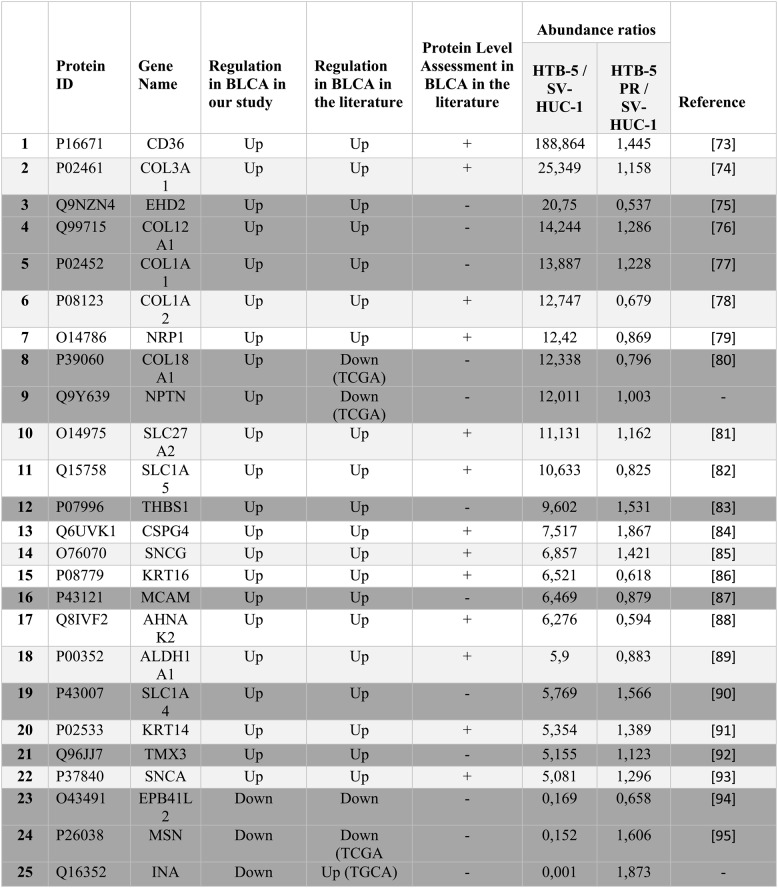
List of diagnostic and/or therapeutic biomarker candidates for bladder cancer. The expression status and abundance ratios of CD36, COL3A1, EHD2, COL12A1, COL1A1, COL1A2, NRP1, COL18A1, NPTN, SLC27A2, SLC1A5, THBS1, CSPG4, SNCG, KRT16, MCAM, AHNAK2, ALDH1A1, SLC1A4, KRT14, TMX3, SNCA, APB41L2, MSN and INA were compared with the previous literature. The protein level expression of 12 markers that were indicated in grey shading was shown first time in this study [74–96].

Although we provided a descriptive analysis of partially reprogrammed bladder cancer cells, there are still limitations to our research. First, although we previously reprogrammed two other bladder cancer cell lines, we provided evidence only for a single cell line in the current study. It would be intriguing, but challenging at the same time, to perform a comparative analysis of different bladder cancer cell lines representative of distinct cancer stages to their reprogrammed counterparts and normal cells to make precise inferences on cancer cell reprogramming and stage-specific biomarker development. Second, partial reprogramming has demonstrated the potential to reverse the cancer phenotype, as we also showed through the regulation of the normalisation of multiple proteins. Further refinement in reprogramming protocols is necessary to control the transient expression of pluripotency factors and terminal differentiation of reprogrammed cancer cells, preferably using a directed differentiation toward bladder epithelium. Lastly, partial reprogramming might induce cancer stem cell-like traits and increase metastasis without achieving true pluripotency, which complicates the interpretation of the results. Therefore, omics data should be evaluated carefully to dissect potential cancer stem cell markers that could be used for diagnostic and therapeutic purposes. We believe partial reprogramming with Yamanaka factors holds a promise for the reversal of cancer phenotypes. However, the technically inherent risk of oncogenic transformation, more refined interventions that focus on different time points of the reprogramming process are necessary to elucidate the proteomic profile of reprogramming in cancer cells.

## Conclusions

Here, we aimed to add novel data that might contribute to solving the complexity of bladder cancer and expand the frame of possibilities to find a more robust diagnostic and/or therapeutic marker by conjoining somatic cell reprogramming and proteomics. Our data suggest the possibility of cancer cell reprogramming and open the gateway to the undifferentiated state of bladder cancer cells, highlighting the proteomic signature during the cancer cell fate reversal and normalisation process. We propose that utilising reprogramming technology for the derivation of patient-specific iPSCs could be used to distinguish variabilities in tumorigenesis, to pinpoint the candidate molecules for therapeutic purposes and to establish tailored therapies for cancer patients.

## Supplementary Information

Supplementary Material 1. Proteome data including the proteins with a minimum of 5 identified unique peptides. All regulated proteins (upregulated and downregulated) in the reprogrammed HTB-5 cells are listed.

Supplementary Material 2. Abundance values of proteins involved in pluripotency, stem cell differentiation and extracellular matrix used in hierarchical clustering.

Supplementary Material 3. Abundance values of proteins involved in epithelial cell differentiation and neural development used in hierarchical clustering.

Supplementary Material 4. Comparative expression analysis of the prospective biomarker candidates revealed by mass spectrometry during the reprogramming of bladder cancer cells with the expression profiles in multiple cancer types using the GEPIA tool.

Supplementary Material 5. Proteome data including all identified proteins.

## Data Availability

Data supporting the results are presented as Supplementary Information in the manuscript (Supporting Information 1, 2, 3 and 5). Supporting Information 4 was created using the publicly available datasets in GEPIA.

## Abbreviations

SeV: Sendai virus
MOI: Multiplicity of Infection
OSKM: Oct-4, Sox-2, Klf-4 and c-Myc
MET: Mesenchymal-to-epithelial transition
MEM: Minimum essential medium (Eagle) in Earle’s BSS with non-essential amino acids
bFGF: Basic Fibroblast Growth Factor
CCK-8: Cell Counting Kit-8
HTB-5-PR: HTB-5 Partially Reprogrammed
PBS: Phosphate-buffered saline
LC/MS-MS: Liquid chromatography-mass spectrometry/ mass spectrometry
UniProt: Universal Protein Resource
GO: Gene Ontology
GEPIA: Gene Expression Profiling Interactive Analysis
iPSC: Induced Pluripotent Stem Cell
Pac: Paclitaxel
Dox: Doxorubicin
TGF-β1: Transforming growth factor-β1
ECM: Extracellular matrix
