# Supplementary material for: Conjoining cell reprogramming and mass spectrometry to identify the proteomic variations in the reprogrammed bladder cancer cells: finding cues of normalisation
Source: BMC Cancer. 2026 Feb 6;26:338. doi: 10.1186/s12885-026-15634-x (PMC12977642; doi:10.1186/s12885-026-15634-x)

Supplementary File 1

Conjoining cell reprogramming and mass spectrometry to identify the proteomic variations in the reprogrammed bladder cancer cells: Finding cues of normalisation

Banu Iskender[1,*], Mehmet Sarihan[1], Bengi Su Rumeysa Barlak[1], Gurler Akpinar[1], Murat Kasap[1]

[1] Kocaeli University Faculty of Medicine Department of Medical Biology Protein Research and Proteomics Laboratory, Umuttepe, 41001, Izmit, Kocaeli-Turkey

*Corresponding Author: Kocaeli University Faculty of Medicine Department of Medical Biology Protein Research and Proteomics Laboratory, Umuttepe, 41001, Izmit, Kocaeli-Turkey banu.iskender@yahoo.com

Supplementary File 1 Proteome data including the proteins with a minimum of 5 identified unique peptides. All regulated proteins (upregulated and downregulated) in the reprogrammed HTB-5 cells are listed.

Whole protein list

Accession	Gene	Description	# Unique Peptides	Abundance Ratio: (HTB-5) / (HHTB-5 PR)	Abundance Ratio: (HTB-5 PR) / (SV-HUC-1)	Abundance Ratio: (HTB-5) / (SV-HUC-1)	Abundances (Grouped): HTB-5 PR	Abundances (Grouped): HTB-5	Abundances (Grouped): SV-HUC-1
P07476	IVL	Involucrin OS=Homo sapiens OX=9606 GN=IVL PE=1 SV=2	14		0,636		381421,3297	276113,28	595748,242
Q8N335	GPD1L	Glycerol-3-phosphate dehydrogenase 1-like protein OS=Homo sapiens OX=9606 GN=GPD1L PE=	6	1000	0,001			103589,6717	99506,78176
P00450	CP	Ceruloplasmin OS=Homo sapiens OX=9606 GN=CP PE=1 SV=2	6	1000	0,001			37680,26668	236658,2951
Q6P5R6	RPL22L1	Ribosomal protein eL22-like OS=Homo sapiens OX=9606 GN=RPL22L1 PE=1 SV=2	3	1000	0,001			343575,0364	161458,6916
O94887	FARP2	FERM, ARHGEF and pleckstrin domain-containing protein 2 OS=Homo sapiens OX=9606 GN=FAR	2	1000	0,001			385192,9603	242415,2396
Q9H4L5	OSBPL3	Oxysterol-binding protein-related protein 3 OS=Homo sapiens OX=9606 GN=OSBPL3 PE=1 SV=1	2	1000	0,001			75506,16651	482833,4055
Q6UW63	POGLUT2	Protein O-glucosyltransferase 2 OS=Homo sapiens OX=9606 GN=POGLUT2 PE=1 SV=1	4	6,102	8,592		841727,4248	753505,0568	51515,35218
Q9NXS2	QPCTL	Glutaminyl-peptide cyclotransferase-like protein OS=Homo sapiens OX=9606 GN=QPCTL PE=1 S	2	1,378	0,797		2291167,473	706521,5458	2284571,84
Q99598	TSNAX	Translin-associated protein X OS=Homo sapiens OX=9606 GN=TSNAX PE=1 SV=1	2	0,621	1,396		749947,8375	390766,9675	93770,45464
O00560	SDCBP	Syntenin-1 OS=Homo sapiens OX=9606 GN=SDCBP PE=1 SV=1	4	0,551	2,778		538479,6978	131037,4198	109958,0698
O95104	SCAF4	SR-related and CTD-associated factor 4 OS=Homo sapiens OX=9606 GN=SCAF4 PE=1 SV=3	4	0,527	1,02		3324851,246	350001,0244	2631201,661
Q15813	TBCE	Tubulin-specific chaperone E OS=Homo sapiens OX=9606 GN=TBCE PE=1 SV=1	5	0,373	3,654		2254641,524	256827,0373	465993,4125
Q9HB07	MYG1	MYG1 exonuclease OS=Homo sapiens OX=9606 GN=MYG1 PE=1 SV=4	3	0,218	5,517		1349904,224	42730,16052	176519,2625
P08237	PFKM	ATP-dependent 6-phosphofructokinase, muscle type OS=Homo sapiens OX=9606 GN=PFKM PE=	3	0,155	8,783		2677250,881	147232,1526	155729,5156
Q8N158	GPC2	Glypican-2 OS=Homo sapiens OX=9606 GN=GPC2 PE=1 SV=1	4	0,147	3,812		1828548,111	40373,70202	155160,5941
P07196	NEFL	Neurofilament light polypeptide OS=Homo sapiens OX=9606 GN=NEFL PE=1 SV=3	12	0,127	180,587		30416481,91	1479326,413	26024,42699
O75363	BCAS1	Breast carcinoma-amplified sequence 1 OS=Homo sapiens OX=9606 GN=BCAS1 PE=1 SV=2	9	0,001	1000		695458,8972		
Q96C23	GALM	Galactose mutarotase OS=Homo sapiens OX=9606 GN=GALM PE=1 SV=1	4	0,001	1000		166664,4928		
Q16790	CA9	Carbonic anhydrase 9 OS=Homo sapiens OX=9606 GN=CA9 PE=1 SV=2	3	0,001	1000		75423,57011		
Q86TG7	PEG10	Retrotransposon-derived protein PEG10 OS=Homo sapiens OX=9606 GN=PEG10 PE=1 SV=2	3	0,001	1000		2113180,538		
Q9UJQ4	SALL4	Sal-like protein 4 OS=Homo sapiens OX=9606 GN=SALL4 PE=1 SV=1	3	0,001	1000		1428385,849		
Q9BW30	TPPP3	Tubulin polymerization-promoting protein family member 3 OS=Homo sapiens OX=9606 GN=TPF	2	0,001	1000		572934,4692		
Q5SZK8	FREM2	FRAS1-related extracellular matrix protein 2 OS=Homo sapiens OX=9606 GN=FREM2 PE=1 SV=3	2	0,001	1000		393138,8366		
Q13449	LSAMP	Limbic system-associated membrane protein OS=Homo sapiens OX=9606 GN=LSAMP PE=1 SV=2	2	0,001	1000		647670,7044		
Q9UQR0	SCML2	Sex comb on midleg-like protein 2 OS=Homo sapiens OX=9606 GN=SCML2 PE=1 SV=1	2	0,001	1000		969506,7663		
Q6PGN9	PSRC1	Proline/serine-rich coiled-coil protein 1 OS=Homo sapiens OX=9606 GN=PSRC1 PE=1 SV=1	2	0,001	1000		794290,0139		
Q9Y4B5	MTCL1	Microtubule cross-linking factor 1 OS=Homo sapiens OX=9606 GN=MTCL1 PE=1 SV=5	2	0,001	1000		134908,3729		
O15397	IPO8	Importin-8 OS=Homo sapiens OX=9606 GN=IPO8 PE=1 SV=2	2	0,001	1000		308670,9949		
P31271	HOXA13	Homeobox protein Hox-A13 OS=Homo sapiens OX=9606 GN=HOXA13 PE=1 SV=3	2	0,001	1000		622805,475		
Q5T0N5	FNBP1L	Formin-binding protein 1-like OS=Homo sapiens OX=9606 GN=FNBP1L PE=1 SV=3	2	0,001	1000		144486,62		
P49407	ARRB1	Beta-arrestin-1 OS=Homo sapiens OX=9606 GN=ARRB1 PE=1 SV=2	2	0,001	1000		109317,2174		
O75110	ATP9A	Probable phospholipid-transporting ATPase IIA OS=Homo sapiens OX=9606 GN=ATP9A PE=1 SV=	2	0,001	1000		168872,4815		
Q9C004	SPRY4	Protein sprouty homolog 4 OS=Homo sapiens OX=9606 GN=SPRY4 PE=1 SV=2	2	0,001	1000		1975946,043		
Q8IWS0	PHF6	PHD finger protein 6 OS=Homo sapiens OX=9606 GN=PHF6 PE=1 SV=1	2	0,001	1000		1919712,785		
Q9NZD2	GLTP	Glycolipid transfer protein OS=Homo sapiens OX=9606 GN=GLTP PE=1 SV=3	2	0,001	1000		206256,9305		
Q9H0C8	ILKAP	Integrin-linked kinase-associated serine/threonine phosphatase 2C OS=Homo sapiens OX=9606	2	0,001	1000		194289,5197		
Q9UEY8	ADD3	Gamma-adducin OS=Homo sapiens OX=9606 GN=ADD3 PE=1 SV=1	2	0,001	1000		300496,6324		
Q4ZIN3	TMEM259	Membralin OS=Homo sapiens OX=9606 GN=TMEM259 PE=1 SV=1	2	0,001	1000		82771,06657		
Q9UDR5	AASS	Alpha-aminoadipic semialdehyde synthase, mitochondrial OS=Homo sapiens OX=9606 GN=AASS	6		1000	1000	2112982,601	49423,593	
Q13336	SLC14A1	Urea transporter 1 OS=Homo sapiens OX=9606 GN=SLC14A1 PE=1 SV=2	3		1000	1000	96281,46607	492893,6472	
P51884	LUM	Lumican OS=Homo sapiens OX=9606 GN=LUM PE=1 SV=2	7	1000		1000		15940967,65	
P05106	ITGB3	Integrin beta-3 OS=Homo sapiens OX=9606 GN=ITGB3 PE=1 SV=2	4	1000		1000		1782929,246	
P31513	FMO3	Flavin-containing monooxygenase 3 OS=Homo sapiens OX=9606 GN=FMO3 PE=1 SV=5	4	1000		1000		4196968,802	
Q9ULW8	PADI3	Protein-arginine deiminase type-3 OS=Homo sapiens OX=9606 GN=PADI3 PE=1 SV=2	3	1000		1000		3723,752119	
Q9HAW9	UGT1A8	UDP-glucuronosyltransferase 1A8 OS=Homo sapiens OX=9606 GN=UGT1A8 PE=1 SV=1	3	1000		1000		2756758,745	
P01023	A2M	Alpha-2-macroglobulin OS=Homo sapiens OX=9606 GN=A2M PE=1 SV=3	3	1000		1000		1318523,732	
P29350	PTPN6	Tyrosine-protein phosphatase non-receptor type 6 OS=Homo sapiens OX=9606 GN=PTPN6 PE=1	3	1000		1000		388574,4812	
P51911	CNN1	Calponin-1 OS=Homo sapiens OX=9606 GN=CNN1 PE=1 SV=2	3	1000		1000		4825100,172	
P30740	SERPINB1	Leukocyte elastase inhibitor OS=Homo sapiens OX=9606 GN=SERPINB1 PE=1 SV=1	3	1000		1000		690215,1391	
Q15345	LRRC41	Leucine-rich repeat-containing protein 41 OS=Homo sapiens OX=9606 GN=LRRC41 PE=1 SV=3	2	1000		1000		58938,43075	
P34949	MPI	Mannose-6-phosphate isomerase OS=Homo sapiens OX=9606 GN=MPI PE=1 SV=2	2	1000		1000		291948,0974	
Q8N3V7	SYNPO	Synaptopodin OS=Homo sapiens OX=9606 GN=SYNPO PE=1 SV=2	2	1000		1000		1306899,39	
O14639	ABLIM1	Actin-binding LIM protein 1 OS=Homo sapiens OX=9606 GN=ABLIM1 PE=1 SV=3	2	1000		1000		318917,9333	
Q96S99	PLEKHF1	Pleckstrin homology domain-containing family F member 1 OS=Homo sapiens OX=9606 GN=PLEI	2	1000		1000		55648,12183	
Q16832	DDR2	Discoidin domain-containing receptor 2 OS=Homo sapiens OX=9606 GN=DDR2 PE=1 SV=2	2	1000		1000		284809,9175	
Q7L1W4	LRRC8D	Volume-regulated anion channel subunit LRRC8D OS=Homo sapiens OX=9606 GN=LRRC8D PE=1	2	1000		1000		750977,5248	
Q9UJ72	ANXA10	Annexin A10 OS=Homo sapiens OX=9606 GN=ANXA10 PE=1 SV=3	2	1000		1000		47832,4755	
Q3SY69	ALDH1L2	Mitochondrial 10-formyltetrahydrofolate dehydrogenase OS=Homo sapiens OX=9606 GN=ALDH	2	1000		1000		1436524,762	
P26447	S100A4	Protein S100-A4 OS=Homo sapiens OX=9606 GN=S100A4 PE=1 SV=1	3	64,867	1000	1000	499494,494	46508783,28	
Q0ZGT2	NEXN	Nexilin OS=Homo sapiens OX=9606 GN=NEXN PE=1 SV=1	3	12,684	1000	1000	24511,03854	1286634,002	
Q6EMK4	VASN	Vasorin OS=Homo sapiens OX=9606 GN=VASN PE=1 SV=1	2	8,301	1000	1000	228387,1982	2062924,347	
P07093	SERPINE2	Glia-derived nexin OS=Homo sapiens OX=9606 GN=SERPINE2 PE=1 SV=1	2	6,95	1000	1000	111623,3897	1426027,352	
O95084	PRSS23	Serine protease 23 OS=Homo sapiens OX=9606 GN=PRSS23 PE=1 SV=1	2	6,529	1000	1000	314562,077	1961124,762	
Q14627	IL13RA2	Interleukin-13 receptor subunit alpha-2 OS=Homo sapiens OX=9606 GN=IL13RA2 PE=1 SV=1	2	5,348	1000	1000	213422,6176	1448061,769	
A0A0G2JPR0	C4A	Complement C4A (Rodgers blood group) OS=Homo sapiens OX=9606 GN=C4A PE=1 SV=1	3	5,023	1000	1000	37219,80078	1208246,624	
P35354	PTGS2	Prostaglandin G/H synthase 2 OS=Homo sapiens OX=9606 GN=PTGS2 PE=1 SV=2	4	4,186	1000	1000	141994,6152	1563993,303	
P21810	BGN	Biglycan OS=Homo sapiens OX=9606 GN=BGN PE=1 SV=2	2	3,494	1000	1000	347084,8424	1212725,278	
Q96HW7	INTS4	Integrator complex subunit 4 OS=Homo sapiens OX=9606 GN=INTS4 PE=1 SV=2	2	3,231	1000	1000	403059,254	1436262,287	
P26022	PTX3	Pentraxin-related protein PTX3 OS=Homo sapiens OX=9606 GN=PTX3 PE=1 SV=3	5	3,168	1000	1000	121965,0819	690871,6229	
Q13228	SELENBP1	Methanethiol oxidase OS=Homo sapiens OX=9606 GN=SELENBP1 PE=1 SV=2	2	2,906	1000	1000	437425,0185	1177913,164	
Q99985	SEMA3C	Semaphorin-3C OS=Homo sapiens OX=9606 GN=SEMA3C PE=2 SV=2	5	2,819	1000	1000	397932,8909	1055090,214	
Q2PPJ7	RALGAPA2	Ral GTPase-activating protein subunit alpha-2 OS=Homo sapiens OX=9606 GN=RALGAPA2 PE=1	2	2,525	1000	1000	55012,46534	42679,32719	
P17302	GJA1	Gap junction alpha-1 protein OS=Homo sapiens OX=9606 GN=GJA1 PE=1 SV=2	2	2,066	1000	1000	629236,5748	1085956,365	
Q14571	ITPR2	Inositol 1,4,5-trisphosphate receptor type 2 OS=Homo sapiens OX=9606 GN=ITPR2 PE=1 SV=2	3	1,836	1000	1000	94090,66459	164293,8118	
P52895	AKR1C2	Aldo-keto reductase family 1 member C2 OS=Homo sapiens OX=9606 GN=AKR1C2 PE=1 SV=3	6	1,722	1000	1000	443572,7934	645802,7443	
O00746	NME4	Nucleoside diphosphate kinase, mitochondrial OS=Homo sapiens OX=9606 GN=NME4 PE=1 SV=1	3	1,666	1000	1000	498238,1217	2413434,86	
Q32P41	TRMT5	tRNA (guanine(37)-N1)-methyltransferase OS=Homo sapiens OX=9606 GN=TRMT5 PE=1 SV=3	2	1,657	1000	1000	595568,0252	1383102,531	
P12109	COL6A1	Collagen alpha-1(VI) chain OS=Homo sapiens OX=9606 GN=COL6A1 PE=1 SV=3	2	1,606	1000	1000	764259,1486	1263873,34	
Q16222	UAP1	UDP-N-acetylhexosamine pyrophosphorylase OS=Homo sapiens OX=9606 GN=UAP1 PE=1 SV=3	2	1,546	1000	1000	843073,3224	1376552,3	
P00751	CFB	Complement factor B OS=Homo sapiens OX=9606 GN=CFB PE=1 SV=2	2	1,442	1000	1000	65381,06988	94275,16776	
Q9UGP4	LIMD1	LIM domain-containing protein 1 OS=Homo sapiens OX=9606 GN=LIMD1 PE=1 SV=1	2	1,413	1000	1000	65921,80904	98306,35975	
Q9HAV0	GNB4	Guanine nucleotide-binding protein subunit beta-4 OS=Homo sapiens OX=9606 GN=GNB4 PE=1	2	1,277	1000	1000	501577,1405	640365,3733	
Q9NP58	ABCB6	ATP-binding cassette sub-family B member 6 OS=Homo sapiens OX=9606 GN=ABCB6 PE=1 SV=1	2	1,134	1000	1000	653708,1974	504080,7938	
P15428	HPGD	15-hydroxyprostaglandin dehydrogenase [NAD(+)] OS=Homo sapiens OX=9606 GN=HPGD PE=1 S	7	1,039	1000	1000	86492,94813	750211,8274	
P54868	HMGCS2	Hydroxymethylglutaryl-CoA synthase, mitochondrial OS=Homo sapiens OX=9606 GN=HMGCS2 P	10	1,027	1000	1000	1374717,867	218758,2434	
Q10472	GALNT1	Polypeptide N-acetylgalactosaminyltransferase 1 OS=Homo sapiens OX=9606 GN=GALNT1 PE=1	5	0,935	1000	1000	1192616,983	729045,5117	
P40424	PBX1	Pre-B-cell leukemia transcription factor 1 OS=Homo sapiens OX=9606 GN=PBX1 PE=1 SV=1	2	0,799	1000	1000	1050051,474	818776,2227	
Q92673	SORL1	Sortilin-related receptor OS=Homo sapiens OX=9606 GN=SORL1 PE=1 SV=2	6	0,785	1000	1000	396328,1509	1669888,783	
P13611	VCAN	Versican core protein OS=Homo sapiens OX=9606 GN=VCAN PE=1 SV=3	2	0,772	1000	1000	1514391,736	1131697,24	
O75688	PPM1B	Protein phosphatase 1B OS=Homo sapiens OX=9606 GN=PPM1B PE=1 SV=1	2	0,728	1000	1000	832689,8057	311794,5494	
P36969	GPX4	Phospholipid hydroperoxide glutathione peroxidase GPX4 OS=Homo sapiens OX=9606 GN=GPX4	2	0,711	1000	1000	1123211,974	719081,2633	
Q96GU1	PAGE5	P antigen family member 5 OS=Homo sapiens OX=9606 GN=PAGE5 PE=1 SV=2	4	0,662	1000	1000	6250230,371	3943969,558	
Q03001	DST	Dystonin OS=Homo sapiens OX=9606 GN=DST PE=1 SV=4	3	0,632	1000	1000	781205,9359	1085664,657	
Q93062	RBPMS	RNA-binding protein with multiple splicing OS=Homo sapiens OX=9606 GN=RBPMS PE=1 SV=1	2	0,59	1000	1000	540048,7003	326854,2429	
Q8TDY4	ASAP3	Arf-GAP with SH3 domain, ANK repeat and PH domain-containing protein 3 OS=Homo sapiens O	2	0,579	1000	1000	782808,8382	168383,4473	
P82970	HMGN5	High mobility group nucleosome-binding domain-containing protein 5 OS=Homo sapiens OX=96	2	0,552	1000	1000	678793,0139	362037,3283	
Q9UKZ1	CNOT11	CCR4-NOT transcription complex subunit 11 OS=Homo sapiens OX=9606 GN=CNOT11 PE=1 SV=1	2	0,545	1000	1000	134900,3507	73456,27652	
O00470	MEIS1	Homeobox protein Meis1 OS=Homo sapiens OX=9606 GN=MEIS1 PE=1 SV=1	2	0,528	1000	1000	931114,3422	693010,1368	
Q9Y6U3	SCIN	Scinderin OS=Homo sapiens OX=9606 GN=SCIN PE=1 SV=4	2	0,402	1000	1000	267855,1084	118260,1879	
O60245	PCDH7	Protocadherin-7 OS=Homo sapiens OX=9606 GN=PCDH7 PE=1 SV=2	5	0,4	1000	1000	168503,5574	922913,1866	
Q9UBQ6	EXTL2	Exostosin-like 2 OS=Homo sapiens OX=9606 GN=EXTL2 PE=1 SV=1	2	0,336	1000	1000	721432,3014	319333,2672	
P54105	CLNS1A	Methylosome subunit pICln OS=Homo sapiens OX=9606 GN=CLNS1A PE=1 SV=1	2	0,312	1000	1000	1027033,912	153393,442	
Q8IZA0	KIAA0319L	Dyslexia-associated protein KIAA0319-like protein OS=Homo sapiens OX=9606 GN=KIAA0319L P	2	0,293	1000	1000	654805,8125	196159,6715	
Q9NSK0	KLC4	Kinesin light chain 4 OS=Homo sapiens OX=9606 GN=KLC4 PE=1 SV=3	2	0,277	1000	1000	801200,3123	97633,13064	
P35520	CBS	Cystathionine beta-synthase OS=Homo sapiens OX=9606 GN=CBS PE=1 SV=2	2	0,275	1000	1000	1707799,373	476874,8028	
Q9UN66	PCDHB8	Protocadherin beta-8 OS=Homo sapiens OX=9606 GN=PCDHB8 PE=2 SV=4	2	0,259	1000	1000	619426,8538	65889,87844	
Q9Y4G6	TLN2	Talin-2 OS=Homo sapiens OX=9606 GN=TLN2 PE=1 SV=4	2	0,249	1000	1000	853085,4176	67970,32172	
Q99856	ARID3A	AT-rich interactive domain-containing protein 3A OS=Homo sapiens OX=9606 GN=ARID3A PE=1	3	0,243	1000	1000	1679463,222	182753,7555	

Q9H993	ARMT1	Damage-control phosphatase ARMT1 OS=Homo sapiens OX=9606 GN=ARMT1 PE=1 SV=1	2	0,223	1000	1000	504580,3534	112603,9199	
Q9Y2H0	DLGAP4	Disks large-associated protein 4 OS=Homo sapiens OX=9606 GN=DLGAP4 PE=1 SV=3	2	0,218	1000	1000	1333285,233	319948,2389	
Q9UN81	L1RE1	LINE-1 retrotransposable element ORF1 protein OS=Homo sapiens OX=9606 GN=L1RE1 PE=1 SV=	4	0,177	1000	1000	2908551,955	313766,0651	
P12111	COL6A3	Collagen alpha-3(VI) chain OS=Homo sapiens OX=9606 GN=COL6A3 PE=1 SV=5	3	0,17	1000	1000	706415,3514	39619,40836	
Q13642	FHL1	Four and a half LIM domains protein 1 OS=Homo sapiens OX=9606 GN=FHL1 PE=1 SV=4	2	0,15	1000	1000	3582980,359	447882,8653	
P15882	CHN1	N-chimaerin OS=Homo sapiens OX=9606 GN=CHN1 PE=1 SV=3	2	0,122	1000	1000	230680,7619	28257,59966	
P05186	ALPL	Alkaline phosphatase, tissue-nonspecific isozyme OS=Homo sapiens OX=9606 GN=ALPL PE=1 SV=	4	0,104	1000	1000	1557431,371	70794,45118	
O60282	KIF5C	Kinesin heavy chain isoform 5C OS=Homo sapiens OX=9606 GN=KIF5C PE=1 SV=1	2	0,088	1000	1000	697941,7189	26848,44008	
Q9H9Z2	LIN28A	Protein lin-28 homolog A OS=Homo sapiens OX=9606 GN=LIN28A PE=1 SV=1	4	0,04	1000	1000	10703950,88	31905,98097	
Q9BPU6	DPYSL5	Dihydropyrimidinase-related protein 5 OS=Homo sapiens OX=9606 GN=DPYSL5 PE=1 SV=1	9	0,025	1000	1000	15706303,05	150501,1907	
P16671	CD36	Platelet glycoprotein 4 OS=Homo sapiens OX=9606 GN=CD36 PE=1 SV=2	8	53,466	1,445	188,864	872002,9421	99417726,13	651962,2264
Q5EB52	MEST	Mesoderm-specific transcript homolog protein OS=Homo sapiens OX=9606 GN=MEST PE=1 SV=2	4	1,004	54,127	86,36	8050483,066	9136023,119	65589,31037
P04216	THY1	Thy-1 membrane glycoprotein OS=Homo sapiens OX=9606 GN=THY1 PE=1 SV=2	3	6,42	5,697	48,536	3823281,631	25819320,25	394580,7283
P08133	ANXA6	Annexin A6 OS=Homo sapiens OX=9606 GN=ANXA6 PE=1 SV=3	30	0,67	63,943	45,162	111788999,6	73929609,25	621924,5784
P08648	ITGA5	Integrin alpha-5 OS=Homo sapiens OX=9606 GN=ITGA5 PE=1 SV=2	11	11,868	2,979	44,474	3547361,7	45977368,48	818230,2829
P23219	PTGS1	Prostaglandin G/H synthase 1 OS=Homo sapiens OX=9606 GN=PTGS1 PE=1 SV=2	5	27,162	1,863	32,559	48982,90625	4008078,612	139518,517
Q8NBQ5	HSD17B11	Estradiol 17-beta-dehydrogenase 11 OS=Homo sapiens OX=9606 GN=HSD17B11 PE=1 SV=4	6	1,758	14,022	28,187	6316562,193	12328754,91	320117,6706
P06703	S100A6	Protein S100-A6 OS=Homo sapiens OX=9606 GN=S100A6 PE=1 SV=1	5	12,677	2,181	27,387	5034805,567	92846719,3	2517376,319
O95340	PAPSS2	Bifunctional 3'-phosphoadenosine 5'-phosphosulfate synthase 2 OS=Homo sapiens OX=9606 GN	11	4,006	5,778	27,152	5195814,369	22664948,62	265064,0351
P12277	CKB	Creatine kinase B-type OS=Homo sapiens OX=9606 GN=CKB PE=1 SV=1	13	0,229	104,267	26,258	178899578,5	41112571,44	1322434,482
P02461	COL3A1	Collagen alpha-1(III) chain OS=Homo sapiens OX=9606 GN=COL3A1 PE=1 SV=4	13	12,524	1,158	25,349	1316355,683	34879145,93	326421,893
Q9NS25	SPANXB1	Sperm protein associated with the nucleus on the X chromosome B1 OS=Homo sapiens OX=9606	3	1000	0,001	24,355		13522268,12	88657,57368
O43294	TGFB1I1	Transforming growth factor beta-1-induced transcript 1 protein OS=Homo sapiens OX=9606 GN=	2	3,15	1,629	23,816	92613,85663	809577,7606	77918,77037
P98082	DAB2	Disabled homolog 2 OS=Homo sapiens OX=9606 GN=DAB2 PE=1 SV=3	9	3,788	14,418	21,621	2574876,221	10425942,31	144261,6288
P07099	EPHX1	Epoxide hydrolase 1 OS=Homo sapiens OX=9606 GN=EPHX1 PE=1 SV=1	18	14,594	1,285	20,891	13486875,91	194354890,7	9034725,843
Q9NZN4	EHD2	EH domain-containing protein 2 OS=Homo sapiens OX=9606 GN=EHD2 PE=1 SV=2	11	25,512	0,537	20,75	978945,2094	22543239,22	1194428,834
P40261	NNMT	Nicotinamide N-methyltransferase OS=Homo sapiens OX=9606 GN=NNMT PE=1 SV=1	3	7,298	1,876	20,629	378461,356	10553884,77	544951,2503
P08473	MME	Neprilysin OS=Homo sapiens OX=9606 GN=MME PE=1 SV=2	22	6,421	5,829	17,823	13436493,01	97741475,15	902872,9581
Q9ULT8	HECTD1	E3 ubiquitin-protein ligase HECTD1 OS=Homo sapiens OX=9606 GN=HECTD1 PE=1 SV=4	2	3,086	5,164	17,406	94707,31739	292268,7973	18340,63102
P09467	FBP1	Fructose-1,6-bisphosphatase 1 OS=Homo sapiens OX=9606 GN=FBP1 PE=1 SV=5	7	2,242	4,091	17,006	1602500,658	3196701,303	109730,318
O75368	SH3BGRL	Adapter SH3BGRL OS=Homo sapiens OX=9606 GN=SH3BGRL PE=1 SV=1	5	0,723	23,356	16,746	6729074,307	4219561,418	137631,4997
Q14195	DPYSL3	Dihydropyrimidinase-related protein 3 OS=Homo sapiens OX=9606 GN=DPYSL3 PE=1 SV=1	12	4,356	4,095	16,599	11456347,83	50296053,38	2216617,835
P35052	GPC1	Glypican-1 OS=Homo sapiens OX=9606 GN=GPC1 PE=1 SV=2	7	6,776	2,642	16,466	1464345,972	10849334,99	379000,6621
Q14194	CRMP1	Dihydropyrimidinase-related protein 1 OS=Homo sapiens OX=9606 GN=CRMP1 PE=1 SV=1	15	0,568	14,76	16,085	18958553,15	1050586,314	259510,6531
A0AAG2UVC5	COL3A1	Collagen type III alpha 1 chain OS=Homo sapiens OX=9606 GN=COL3A1 PE=1 SV=1	13	9,677	0,884	15,686	3632336,485	38982703,18	3806095,118
P05120	SERPINB2	Plasminogen activator inhibitor 2 OS=Homo sapiens OX=9606 GN=SERPINB2 PE=1 SV=2	4	1000	0,001	15,249		6311526,041	265281,8244
Q9UBG0	MRC2	C-type mannose receptor 2 OS=Homo sapiens OX=9606 GN=MRC2 PE=1 SV=2	4	0,795	10,883	14,388	4707206,201	2903921,014	119223,3718
O76070	SNCG	Gamma-synuclein OS=Homo sapiens OX=9606 GN=SNCG PE=1 SV=2	9	16,882	1,286	14,244	1530270,361	16107179,01	962435,3175
Q99715	COL12A1	Collagen alpha-1(XII) chain OS=Homo sapiens OX=9606 GN=COL12A1 PE=1 SV=2	49	12,761	1,228	13,887	4591347,219	73263797,89	4173557,536
Q9UI42	CPA4	Carboxypeptidase A4 OS=Homo sapiens OX=9606 GN=CPA4 PE=1 SV=2	3	13,932	0,453	13,824	255953,0087	2828604,308	101271,3426
P08962	CD63	CD63 antigen OS=Homo sapiens OX=9606 GN=CD63 PE=1 SV=2	2	0,824	17,864	13,044	6874842,922	7326892,115	557922,5605
P02452	COL1A1	Collagen alpha-1(I) chain OS=Homo sapiens OX=9606 GN=COL1A1 PE=1 SV=6	29	20,744	0,679	12,747	5111451,656	140008907,6	94110,57986
P08123	COL1A2	Collagen alpha-2(I) chain OS=Homo sapiens OX=9606 GN=COL1A2 PE=1 SV=7	22	10,135	0,869	12,42	1489252,904	72595529,17	4958899,404
O14786	NRP1	Neuropilin-1 OS=Homo sapiens OX=9606 GN=NRP1 PE=1 SV=3	6	14,65	0,796	12,338	107627,6806	5380069,826	135325,9065
P08779	KRT16	Keratin, type I cytoskeletal 16 OS=Homo sapiens OX=9606 GN=KRT16 PE=1 SV=4	11	12,439	1,003	12,011	2944829,574	39463858,68	2865191,493
P62736	ACTA2	Actin, aortic smooth muscle OS=Homo sapiens OX=9606 GN=ACTA2 PE=1 SV=1	8	3,388	3,025	11,967	240390199,2	437907829,1	336235321,5
Q86SJ2	AMIGO2	Amphoterin-induced protein 2 OS=Homo sapiens OX=9606 GN=AMIGO2 PE=1 SV=1	3	8,809	1,429	11,884	117865,4411	1509064,021	79451,69401
O75487	GPC4	Glypican-4 OS=Homo sapiens OX=9606 GN=GPC4 PE=1 SV=4	4	0,257	45,255	11,614	4011469,452	728614,3524	62735,55576
P19022	CDH2	Cadherin-2 OS=Homo sapiens OX=9606 GN=CDH2 PE=1 SV=4	7	2,634	2,363	11,598	2465161,62	9209150,048	454104,6754
P01889	HLA-B	HLA class I histocompatibility antigen, B alpha chain OS=Homo sapiens OX=9606 GN=HLA-B PE=1	4	18,028	0,465	11,434	5220416,932	104594368,4	34183285,86
P04259	KRT6B	Keratin, type II cytoskeletal 6B OS=Homo sapiens OX=9606 GN=KRT6B PE=1 SV=5	3	20,879	0,05	11,23	229887,2953	4484463,369	8044668,846
Q14554	PDIA5	Protein disulfide-isomerase A5 OS=Homo sapiens OX=9606 GN=PDIA5 PE=1 SV=1	5	1,816	6,327	11,155	2635093,27	4643716,112	211136,8272
P43121	MCAM	Cell surface glycoprotein MUC18 OS=Homo sapiens OX=9606 GN=MCAM PE=1 SV=2	9	12,418	1,162	11,131	1389664,803	21655785,15	1675506,498
Q9UKY3	CES1P1	Putative inactive carboxylesterase 4 OS=Homo sapiens OX=9606 GN=CES1P1 PE=5 SV=2	3	8,315	5,347	11,115	3624548,734	13322591,43	516449,5748
P23634	ATP2B4	Plasma membrane calcium-transporting ATPase 4 OS=Homo sapiens OX=9606 GN=ATP2B4 PE=1	15	4,119	2,68	10,937	14129901,93	64460807,52	5468529,636
Q9Y2V2	CARHSP1	Calcium-regulated heat-stable protein 1 OS=Homo sapiens OX=9606 GN=CARHSP1 PE=1 SV=2	2	0,994	10,97	10,908	2120254,544	2108279,799	193277,9107
Q8IVF2	AHNAK2	Protein AHNAK2 OS=Homo sapiens OX=9606 GN=AHNAK2 PE=1 SV=2	25	12,164	0,825	10,633	1101896,523	39392930,04	3192024,329
P32456	GBP2	Guanylate-binding protein 2 OS=Homo sapiens OX=9606 GN=GBP2 PE=1 SV=3	2	16,466	0,641	10,467	84217,75733	1871275,147	183353,6263
Q96QD8	SLC38A2	Sodium-coupled neutral amino acid symporter 2 OS=Homo sapiens OX=9606 GN=SLC38A2 PE=1	4	1,694	5,205	10,197	2743947,888	4049491,674	478978,9709
Q16881	TXNRD1	Thioredoxin reductase 1, cytoplasmic OS=Homo sapiens OX=9606 GN=TXNRD1 PE=1 SV=3	18	1,442	8,604	10,196	26337195,91	34231512,5	3191076,281
Q05682	CALD1	Caldesmon OS=Homo sapiens OX=9606 GN=CALD1 PE=1 SV=3	17	4,576	2,396	10,126	31903641,68	151978429,9	13806538,3
P62328	TMSB4X	Thymosin beta-4 OS=Homo sapiens OX=9606 GN=TMSB4X PE=1 SV=2	3	1,149	9,348	10,111	412385,4716	482855,0106	44114,60856
P27487	DPP4	Dipeptidyl peptidase 4 OS=Homo sapiens OX=9606 GN=DPP4 PE=1 SV=2	5	1000	0,001	9,904		8230473,032	30497,91259
P00352	ALDH1A1	Aldehyde dehydrogenase 1A1 OS=Homo sapiens OX=9606 GN=ALDH1A1 PE=1 SV=2	15	12,07	1,531	9,602	4783303,966	61852487,23	1810896,644
Q96DG6	CMBL	Carboxymethylenebutenolidase homolog OS=Homo sapiens OX=9606 GN=CMBL PE=1 SV=1	3	0,882	7,967	9,515	2740878,75	2492923,096	104952,5811
P23141	CES1	Liver carboxylesterase 1 OS=Homo sapiens OX=9606 GN=CES1 PE=1 SV=2	14	10,603	0,497	9,496	4607173,459	54133586,87	3491498,465
Q01995	TAGLN	Transgelin OS=Homo sapiens OX=9606 GN=TAGLN PE=1 SV=4	11	5,218	1,79	8,959	18807737,59	108518228,6	11154557,45
Q9Y680	FKBP7	Peptidyl-prolyl cis-trans isomerase FKBP7 OS=Homo sapiens OX=9606 GN=FKBP7 PE=1 SV=2	4	1,347	6,343	8,582	2467305,462	3358704,788	296564,337
Q9UKX5	ITGA11	Integrin alpha-11 OS=Homo sapiens OX=9606 GN=ITGA11 PE=1 SV=2	7	7,375		8,385	118915,1242	6822269,763	54805,4583
O75891	ALDH1L1	Cytosolic 10-formyltetrahydrofolate dehydrogenase OS=Homo sapiens OX=9606 GN=ALDH1L1 PI	24	1,305	6,744	8,354	3023362,604	5952357,221	526615,8088
Q07954	LRP1	Prolow-density lipoprotein receptor-related protein 1 OS=Homo sapiens OX=9606 GN=LRP1 PE=	22	2,553	3,941	8,319	6900570,003	19212610,1	981840,002
Q687X5	STEAP4	Metalloreductase STEAP4 OS=Homo sapiens OX=9606 GN=STEAP4 PE=1 SV=1	4	14,449	0,743	8,23	86234,1406	4619859,875	589391,9998
P36551	CPOX	Oxygen-dependent coproporphyrinogen-III oxidase, mitochondrial OS=Homo sapiens OX=9606 G	11	10,064	0,861	8,163	9163437,351	81469823,53	9724111,517
Q16658	FSCN1	Fascin OS=Homo sapiens OX=9606 GN=FSCN1 PE=1 SV=3	11	0,38	20,954	8,108	110311753,2	39422798,73	3858392,049
Q01814	ATP2B2	Plasma membrane calcium-transporting ATPase 2 OS=Homo sapiens OX=9606 GN=ATP2B2 PE=1	4	1,758	4,047	7,969	2938578,938	7071875,284	638735,407
P09486	SPARC	SPARC OS=Homo sapiens OX=9606 GN=SPARC PE=1 SV=1	4	5,1	1,64	7,606	5171587,635	23202215,19	2749136,884
Q68EM7	ARHGAP17	Rho GTPase-activating protein 17 OS=Homo sapiens OX=9606 GN=ARHGAP17 PE=1 SV=1	3	4,066	1,868	7,594	772981,376	3088344,661	462195,837
B1AHL2	FBLN1	Fibulin-1 OS=Homo sapiens OX=9606 GN=FBLN1 PE=1 SV=1	4	1,65	4,365	7,573	1736668,781	3459174,343	110585,7778
P39060	COL18A1	Collagen alpha-1(XVIII) chain OS=Homo sapiens OX=9606 GN=COL18A1 PE=1 SV=5	9	4,957	1,867	7,517	1856971,2	10068331,12	941327,4971
P09601	HMOX1	Heme oxygenase 1 OS=Homo sapiens OX=9606 GN=HMOX1 PE=1 SV=1	7	12,767	0,618	7,406	338680,7086	13456104,69	1363057,842
O95865	DDAH2	Putative hydrolase DDAH2 OS=Homo sapiens OX=9606 GN=DDAH2 PE=1 SV=1	5	0,291	25,508	7,11	18728073,53	4867972,007	280463,3299
P80303	NUCB2	Nucleobindin-2 OS=Homo sapiens OX=9606 GN=NUCB2 PE=1 SV=3	13	2,727	2,372	7,102	11522910,39	30600177,16	4202358,938
Q9Y639	NPTN	Neuroplastin OS=Homo sapiens OX=9606 GN=NPTN PE=1 SV=2	5	4,85	1,421	6,857	2036020,949	11110321,29	1293277,252
Q9P109	GCNT4	Beta-1,3-galactosyl-O-glycosyl-glycoprotein beta-1,6-N-acetylglucosaminyltransferase 4 OS=Hom	2	5,754	1,011	6,764	902213,8034	5191133,403	892431,6158
Q32P28	P3H1	Prolyl 3-hydroxylase 1 OS=Homo sapiens OX=9606 GN=P3H1 PE=1 SV=2	8	1,395	5,484	6,743	12447930,02	18128824,76	1383508,668
P46821	MAP1B	Microtubule-associated protein 1B OS=Homo sapiens OX=9606 GN=MAP1B PE=1 SV=2	51	0,255	17,908	6,686	123185487,7	27340813,2	1936345,255
Q07065	CKAP4	Cytoskeleton-associated protein 4 OS=Homo sapiens OX=9606 GN=CKAP4 PE=1 SV=2	29	2,13	3,353	6,667	121264077,9	243606120,8	45252662,53
O14975	SLC27A2	Long-chain fatty acid transport protein 2 OS=Homo sapiens OX=9606 GN=SLC27A2 PE=1 SV=2	11	5,824	0,618	6,521	5771665,005	18025371,27	4396773,698
P04406	GAPDH	Glyceraldehyde-3-phosphate dehydrogenase OS=Homo sapiens OX=9606 GN=GAPDH PE=1 SV=3	17	0,878	5,057	6,508	446724572,2	419930662,6	101922339,4
Q15758	SLC1A5	Neutral amino acid transporter B(0) OS=Homo sapiens OX=9606 GN=SLC1A5 PE=1 SV=2	9	7,18	0,879	6,469	13495081,48	89644360,35	15456440,13
Q15035	TRAM2	Translocating chain-associated membrane protein 2 OS=Homo sapiens OX=9606 GN=TRAM2 PE=	4	5,128	1,26	6,463	173677,9185	4017752,271	137817,002
P21266	GSTM3	Glutathione S-transferase Mu 3 OS=Homo sapiens OX=9606 GN=GSTM3 PE=1 SV=3	7	0,57	10,182	6,463	13949731,14	6695998,185	948857,9991
Q969H8	MYDGF	Myeloid-derived growth factor OS=Homo sapiens OX=9606 GN=MYDGF PE=1 SV=1	5	2,755	2,222	6,364	11798538,67	30329564,13	4351514,457
P23142	FBLN1	Fibulin-1 OS=Homo sapiens OX=9606 GN=FBLN1 PE=1 SV=4	4	1,809	3,499	6,33	1485431,426	3682780,904	143842,7532
Q16890	TPD52L1	Tumor protein D53 OS=Homo sapiens OX=9606 GN=TPD52L1 PE=1 SV=1	5	3,282	1,661	6,29	2068937,536	8020394,604	1106241,049
Q5VV42	CDKAL1	Threonylcarbamoyladenosine tRNA methylthiotransferase OS=Homo sapiens OX=9606 GN=CDKA	12	1,094	8,077	6,282	23221294,68	22773376,99	2285950,373
P43007	SLC1A4	Neutral amino acid transporter A OS=Homo sapiens OX=9606 GN=SLC1A4 PE=1 SV=1	5	4,636	0,594	6,276	412534,3506	3506245,451	727740,7234
O43854	EDIL3	EGF-like repeat and discoidin I-like domain-containing protein 3 OS=Homo sapiens OX=9606 GN=	4	3,58	1,576	6,189	726552,4009	4464904,621	349382,8037
Q9NWV4	CZIB	CXXC motif containing zinc binding protein OS=Homo sapiens OX=9606 GN=CZIB PE=1 SV=1	3	0,391	15,137	6,164	3832603,296	1161765,226	129753,3723
P20020	ATP2B1	Plasma membrane calcium-transporting ATPase 1 OS=Homo sapiens OX=9606 GN=ATP2B1 PE=1	10	1,539	3,758	6,154	6491152,247	9750254,304	1735485,827
P23469	PTPRE	Receptor-type tyrosine-protein phosphatase epsilon OS=Homo sapiens OX=9606 GN=PTPRE PE=	2	7,108	0,571	6,126	24233,32725	537005,0636	103234,0528
P15090	FABP4	Fatty acid-binding protein, adipocyte OS=Homo sapiens OX=9606 GN=FABP4 PE=1 SV=3	6	1,256	8,735	6,075	3334683,856	3368695,514	190606,6304
P29353	SHC1	SHC-transforming protein 1 OS=Homo sapiens OX=9606 GN=SHC1 PE=1 SV=4	3	3,691	1,668	6,052	51557,2617	702022,6523	30910,07311
P11717	IGF2R	Cation-independent mannose-6-phosphate receptor OS=Homo sapiens OX=9606 GN=IGF2R PE=	16	1,506	3,781	6,052	14807069,79	19992051,03	3017547,89
Q6DKJ4	NXN	Nucleoredoxin OS=Homo sapiens OX=9606 GN=NXN PE=1 SV=2	7	0,379	15,184	5,997	3253388,066	1006170,019	167150,6353
Q8IUE6	H2AC21	Histone H2A type 2-B OS=Homo sapiens OX=9606 GN=H2AC21 PE=1 SV=3	3	0,964	3,966	5,918	19952926,72	26231635,29	3857696,686
P07996	THBS1	Thrombospondin-1 OS=Homo sapiens OX=9606 GN=THBS1 PE=1 SV=2	14	6,014	0,883	5,9	804693,0865	6455306,819	761951,8412
Q16555	DPYSL2	Dihydropyrimidinase-related protein 2 OS=Homo sapiens OX=9606 GN=DPYSL2 PE=1 SV=1	15	0,37	17,36	5,877	171394729,1	57858150,03	22402491,45
O14494	PLPP1	Phospholipid phosphatase 1 OS=Homo sapiens OX=9606 GN=PLPP1 PE=1 SV=1	2	4,552		5,86	265805,6882	2410785,309	185020,638
P02533	KRT14	Keratin, type I cytoskeletal 14 OS=Homo sapiens OX=9606 GN=KRT14 PE=1 SV=4	7	4,489	1,566	5,769	15365623,54	63650182	15700189,15
Q13418	ILK	Integrin-linked protein kinase OS=Homo sapiens OX=9606 GN=ILK PE=1 SV=2	6	1,589	3,425	5,766	2424477,552	3755565,898	644552,3192

Q96RQ1	ERGIC2	Endoplasmic reticulum-Golgi intermediate compartment protein 2 OS=Homo sapiens OX=9606 G	2	2,501	2,1	5,757	623125,7429	1417603,912	296135,8219
Q15646	OASL	2'-5'-oligoadenylate synthase-like protein OS=Homo sapiens OX=9606 GN=OASL PE=1 SV=2	9	10,758	0,466	5,752	926161,1987	10368458,12	1847869,917
O75718	CRTAP	Cartilage-associated protein OS=Homo sapiens OX=9606 GN=CRTAP PE=1 SV=1	6	1,988	2,177	5,752	4590630,99	14010680,42	2054585,288
P09038	FGF2	Fibroblast growth factor 2 OS=Homo sapiens OX=9606 GN=FGF2 PE=1 SV=3	3	1,208	3,1	5,692	1416366,695	1448439,688	108751,7056
A0A1C7CYX9	DPYSL2	Dihydropyrimidinase-related protein 2 OS=Homo sapiens OX=9606 GN=DPYSL2 PE=1 SV=1	17	0,316	19,439	5,612	166277920,4	47175115,52	17989852,04
P09913	IFIT2	Interferon-induced protein with tetratricopeptide repeats 2 OS=Homo sapiens OX=9606 GN=IFIT	5	5,636	0,646	5,547	1291137,206	3692659,528	1343708,442
Q9UL25	RAB21	Ras-related protein Rab-21 OS=Homo sapiens OX=9606 GN=RAB21 PE=1 SV=3	4	0,945	3,813	5,479	4262176,034	3590679,825	796344,612
Q63ZY3	KANK2	KN motif and ankyrin repeat domain-containing protein 2 OS=Homo sapiens OX=9606 GN=KANK	5	3,619	1,5	5,435	896387,6567	3025609,898	249678,8222
Q14764	MVP	Major vault protein OS=Homo sapiens OX=9606 GN=MVP PE=1 SV=4	27	7,786	0,291	5,413	3811233,525	47994894,8	10115538,57
Q96JJ7	TMX3	Protein disulfide-isomerase TMX3 OS=Homo sapiens OX=9606 GN=TMX3 PE=1 SV=2	10	3,569	1,389	5,354	7392316,408	27498887,76	5108378,004
Q9NPH3	IL1RAP	Interleukin-1 receptor accessory protein OS=Homo sapiens OX=9606 GN=IL1RAP PE=1 SV=2	2	1000	0,001	5,337		919934,2662	211504,4174
O43824	GTPBP6	Putative GTP-binding protein 6 OS=Homo sapiens OX=9606 GN=GTPBP6 PE=1 SV=4	3	2,531	8,523	5,3	1105667,597	3079171,261	490041,25
Q9C0H2	TTYH3	Protein tweety homolog 3 OS=Homo sapiens OX=9606 GN=TTYH3 PE=1 SV=3	2	0,394	12,108	5,286	2234462,763	949612,3493	253081,2872
P27105	STOM	Stomatin OS=Homo sapiens OX=9606 GN=STOM PE=1 SV=3	4	3,893	1,563	5,267	1455026,869	4428665,475	551537,9299
Q9UNF1	MAGED2	Melanoma-associated antigen D2 OS=Homo sapiens OX=9606 GN=MAGED2 PE=1 SV=2	14	0,241	17,202	5,229	16883883,91	3932382,869	424137,1877
Q6Y288	B3GLCT	Beta-1,3-glucosyltransferase OS=Homo sapiens OX=9606 GN=B3GLCT PE=1 SV=2	4	1,78	3,415	5,206	948921,0821	2257914,487	214585,3769
Q6UVK1	CSPG4	Chondroitin sulfate proteoglycan 4 OS=Homo sapiens OX=9606 GN=CSPG4 PE=1 SV=2	16	5,202	1,123	5,155	1666897,471	10706168,42	638981,1789
O75962	TRIO	Triple functional domain protein OS=Homo sapiens OX=9606 GN=TRIO PE=1 SV=2	5	1,913	2,653	5,152	878412,8252	1274867,912	232950,2359
Q13232	NME3	Nucleoside diphosphate kinase 3 OS=Homo sapiens OX=9606 GN=NME3 PE=1 SV=2	3	9,12	0,461	5,126	125122,6196	3698154,117	570602,4903
P50454	SERPINH1	Serpin H1 OS=Homo sapiens OX=9606 GN=SERPINH1 PE=1 SV=2	22	3,257	1,644	5,12	234006982,8	745518486,2	149655930,2
P37840	SNCA	Alpha-synuclein OS=Homo sapiens OX=9606 GN=SNCA PE=1 SV=1	5	6,154	1,296	5,081	816925,787	6191530,247	289632,4401
Q8IWE2	FAM114A1	Protein NOXP20 OS=Homo sapiens OX=9606 GN=FAM114A1 PE=1 SV=2	4	5,155	1,326	5,053	709413,75	3524154,338	761309,0121
Q8TCZ2	CD99L2	CD99 antigen-like protein 2 OS=Homo sapiens OX=9606 GN=CD99L2 PE=1 SV=1	2	1,376	3,776	4,967	1596807,308	2237970,27	589989,049
Q8IVL6	P3H3	Prolyl 3-hydroxylase 3 OS=Homo sapiens OX=9606 GN=P3H3 PE=1 SV=1	5	2,208	5,284	4,932	573178,3038	2540145,342	199050,185
P52630	STAT2	Signal transducer and activator of transcription 2 OS=Homo sapiens OX=9606 GN=STAT2 PE=1 SV	4	5,923	1,128	4,884	237530,1876	1546956,33	396798,1089
Q8TED1	GPX8	Probable glutathione peroxidase 8 OS=Homo sapiens OX=9606 GN=GPX8 PE=1 SV=2	3	2,869	1,686	4,854	4921903,906	13672146,14	2357769,713
P28161	GSTM2	Glutathione S-transferase Mu 2 OS=Homo sapiens OX=9606 GN=GSTM2 PE=1 SV=2	2	0,462	9,184	4,841	5151552,86	2039441,122	369798,0699
Q7Z4H8	POGLUT3	Protein O-glucosyltransferase 3 OS=Homo sapiens OX=9606 GN=POGLUT3 PE=1 SV=2	9	2,038	2,138	4,817	6558589,375	11743258,96	3126946,89
O94907	DKK1	Dickkopf-related protein 1 OS=Homo sapiens OX=9606 GN=DKK1 PE=1 SV=1	2	0,127	96,062	4,816	4364435,465	431266,7402	33714,9307
Q9Y490	TLN1	Talin-1 OS=Homo sapiens OX=9606 GN=TLN1 PE=1 SV=3	56	3,157	1,57	4,806	43933294,18	135255864,1	29116532,9
Q13509	TUBB3	Tubulin beta-3 chain OS=Homo sapiens OX=9606 GN=TUBB3 PE=1 SV=2	6	0,157	28,988	4,788	43466043,04	7189985,557	1376677,726
O60613	SELENOF	Selenoprotein F OS=Homo sapiens OX=9606 GN=SELENOF PE=1 SV=4	2	1,619	2,179	4,776	1556911,469	2369528,007	400299,5445
P35080	PFN2	Profilin-2 OS=Homo sapiens OX=9606 GN=PFN2 PE=1 SV=3	3	2,149	2,165	4,754	7236743,723	5841519,04	2203852,491
P50281	MMP14	Matrix metalloproteinase-14 OS=Homo sapiens OX=9606 GN=MMP14 PE=1 SV=3	9	2,546	2,091	4,725	7457858,304	19392031,43	2323321,118
Q9Y625	GPC6	Glypican-6 OS=Homo sapiens OX=9606 GN=GPC6 PE=1 SV=1	5	1,855	2,285	4,722	5979459,144	4823859,819	164735,5958
P11413	G6PD	Glucose-6-phosphate 1-dehydrogenase OS=Homo sapiens OX=9606 GN=G6PD PE=1 SV=4	18	1,823	3,141	4,715	21649632,72	39008525,61	6013502,893
P37059	HSD17B2	17-beta-hydroxysteroid dehydrogenase type 2 OS=Homo sapiens OX=9606 GN=HSD17B2 PE=1 S'	11	1,034	1,202	4,71	5686693,918	3278691,014	2328409,614
Q9ULC3	RAB23	Ras-related protein Rab-23 OS=Homo sapiens OX=9606 GN=RAB23 PE=1 SV=1	5	3,797	1,46	4,669	1887892,885	7876180,217	1571271,167
P09936	UCHL1	Ubiquitin carboxyl-terminal hydrolase isozyme L1 OS=Homo sapiens OX=9606 GN=UCHL1 PE=1 S	5	0,458	12,322	4,662	31673785,16	13950756	2428715,709
P36507	MAP2K2	Dual specificity mitogen-activated protein kinase kinase 2 OS=Homo sapiens OX=9606 GN=MAP2	3	0,839	7,634	4,645	827648,2105	617737,4507	61542,31017
Q8N0X7	SPART	Spartin OS=Homo sapiens OX=9606 GN=SPART PE=1 SV=1	9	1,42	3,01	4,641	5271412,856	7288037,116	1552064,524
Q9UHK6	AMACR	Alpha-methylacyl-CoA racemase OS=Homo sapiens OX=9606 GN=AMACR PE=1 SV=2	7	1,494	0,865	4,627	212592,7036	704391,7429	152989,6748
P50416	CPT1A	Carnitine O-palmitoyltransferase 1, liver isoform OS=Homo sapiens OX=9606 GN=CPT1A PE=1 SV	16	6,22	0,881	4,582	2557522,5	14038723,56	3159398,024
O95487	SEC24B	Protein transport protein Sec24B OS=Homo sapiens OX=9606 GN=SEC24B PE=1 SV=2	3		0,424	4,581	223682,0349	1059371,191	735336,8762
P07437	TUBB	Tubulin beta chain OS=Homo sapiens OX=9606 GN=TUBB PE=1 SV=2	5	0,296	15,06	4,527	397899588,8	117490583,3	26408562,71
P37235	HPCAL1	Hippocalcin-like protein 1 OS=Homo sapiens OX=9606 GN=HPCAL1 PE=1 SV=3	5	6,634	0,713	4,521	2203041,1	12732187,78	2948018,625
Q8TB61	SLC35B2	Adenosine 3'-phospho 5'-phosphosulfate transporter 1 OS=Homo sapiens OX=9606 GN=SLC35B2	4	2,017	2,233	4,521	2705391,853	7169546,766	1078151,159
P78330	PSPH	Phosphoserine phosphatase OS=Homo sapiens OX=9606 GN=PSPH PE=1 SV=2	2	0,66	6,055	4,498	2633535,327	1721165,849	283145,9201
Q96AY3	FKBP10	Peptidyl-prolyl cis-trans isomerase FKBP10 OS=Homo sapiens OX=9606 GN=FKBP10 PE=1 SV=1	13	2,046	2,363	4,491	17756273,15	41959080	8744223,649
Q15417	CNN3	Calponin-3 OS=Homo sapiens OX=9606 GN=CNN3 PE=1 SV=1	9	0,372	10,989	4,484	52497391,1	14623920,48	4336610,45
P22694	PRKACB	cAMP-dependent protein kinase catalytic subunit beta OS=Homo sapiens OX=9606 GN=PRKACB	3	0,591	7,612	4,483	848792,2495	242507,7822	57965,70926
Q68CZ2	TNS3	Tensin-3 OS=Homo sapiens OX=9606 GN=TNS3 PE=1 SV=2	4	1,692	2,665	4,481	1445596,261	2356002,912	458041,2914
O43592	XPOT	Exportin-T OS=Homo sapiens OX=9606 GN=XPOT PE=1 SV=2	4	0,83	5,563	4,466	2389664,173	1495031,156	294515,6716
Q13557	CAMK2D	Calcium/calmodulin-dependent protein kinase type II subunit delta OS=Homo sapiens OX=9606	2	0,894	4,657	4,455	893098,1701	787797,947	108852,2611
O43852	CALU	Calumenin OS=Homo sapiens OX=9606 GN=CALU PE=1 SV=2	23	2,119	2,193	4,432	106393558,9	243716075,9	48520677,06
P15941	MUC1	Mucin-1 OS=Homo sapiens OX=9606 GN=MUC1 PE=1 SV=3	2	1000	0,001	4,41		2098993,345	450355,2379
O60888	CUTA	Protein CutA OS=Homo sapiens OX=9606 GN=CUTA PE=1 SV=2	2	0,833	5,721	4,385	10598017,11	8011999,74	1500488,898
O00299	CLIC1	Chloride intracellular channel protein 1 OS=Homo sapiens OX=9606 GN=CLIC1 PE=1 SV=4	7	0,843	5,251	4,378	54061046,55	49061940,07	8034758,168
P69905	HBA1	Hemoglobin subunit alpha OS=Homo sapiens OX=9606 GN=HBA1 PE=1 SV=2	6	4,909	1,083	4,375	8552518,894	42740973,06	9700757,422
P15289	ARSA	Arylsulfatase A OS=Homo sapiens OX=9606 GN=ARSA PE=1 SV=3	3	1,879	2,297	4,369	621133,7007	1029973,75	319008,8706
A0A0C4DFZ2	ARSA	Arylsulfatase A OS=Homo sapiens OX=9606 GN=ARSA PE=1 SV=1	2	1,914	2,284	4,364	386024,0374	706815,407	158632,0622
P15586	GNS	N-acetylglucosamine-6-sulfatase OS=Homo sapiens OX=9606 GN=GNS PE=1 SV=3	6	0,813	5,593	4,344	9714119,884	8017509,626	1615696,861
P21589	NT5E	5'-nucleotidase OS=Homo sapiens OX=9606 GN=NT5E PE=1 SV=1	11	16,121	0,266	4,339	1344624,398	15288843,49	3823563,297
P09493	TPM1	Tropomyosin alpha-1 chain OS=Homo sapiens OX=9606 GN=TPM1 PE=1 SV=2	6	3,664	1,45	4,302	92205427,92	316191407,2	84309737,49
P02538	KRT6A	Keratin, type II cytoskeletal 6A OS=Homo sapiens OX=9606 GN=KRT6A PE=1 SV=3	2	11,857	0,535	4,301	3241931,883	55089801,71	12137428,38
O60664	PLIN3	Perilipin-3 OS=Homo sapiens OX=9606 GN=PLIN3 PE=1 SV=3	10	2,224	1,823	4,248	13543928,61	30682188,18	6108209,729
P53004	BLVRA	Biliverdin reductase A OS=Homo sapiens OX=9606 GN=BLVRA PE=1 SV=2	4	1,309	3,348	4,246	4330498,226	5113375,287	1371576,161
P17931	LGALS3	Galectin-3 OS=Homo sapiens OX=9606 GN=LGALS3 PE=1 SV=5	7	10,68	0,392	4,218	3656954,22	37718584,44	6733711,658
Q86W92	PPFIBP1	Liprin-beta-1 OS=Homo sapiens OX=9606 GN=PPFIBP1 PE=1 SV=2	4	3,14	1,306	4,199	599058,4771	2254523,127	468225,6045
Q93052	LPP	Lipoma-preferred partner OS=Homo sapiens OX=9606 GN=LPP PE=1 SV=1	7	1,878	2,441	4,195	1976991,076	4820223,671	667834,5849
O00469	PLOD2	Procollagen-lysine,2-oxoglutarate 5-dioxygenase 2 OS=Homo sapiens OX=9606 GN=PLOD2 PE=1	18	9,396	0,514	4,183	7909583,029	78308071,82	27960480,62
Q9BTY2	FUCA2	Plasma alpha-L-fucosidase OS=Homo sapiens OX=9606 GN=FUCA2 PE=1 SV=2	2	1,56	2,599	4,173	1002262,403	1558672,567	385636,7893
P0C0L4	C4A	Complement C4-A OS=Homo sapiens OX=9606 GN=C4A PE=1 SV=2	3	5,28	0,682	4,12	38483,30633	1286433,667	56387,14358
Q9NQP4	PFDN4	Prefoldin subunit 4 OS=Homo sapiens OX=9606 GN=PFDN4 PE=1 SV=1	3	0,621	5,708	4,087	8837222,453	6163906,804	1116507,372
Q13308	PTK7	Inactive tyrosine-protein kinase 7 OS=Homo sapiens OX=9606 GN=PTK7 PE=1 SV=2	13	1,478	2,497	4,082	16299813,46	22595639,98	5434317,005
Q96G23	CERS2	Ceramide synthase 2 OS=Homo sapiens OX=9606 GN=CERS2 PE=1 SV=1	3	1,97	1,846	4,038	2128905,985	3296562,784	1911600,745
P21333	FLNA	Filamin-A OS=Homo sapiens OX=9606 GN=FLNA PE=1 SV=4	78	1,593	2,822	4,008	494200379,4	727031350,4	177284201
Q8N129	CNPY4	Protein canopy homolog 4 OS=Homo sapiens OX=9606 GN=CNPY4 PE=1 SV=1	2	1,876	2,393	3,993	984402,3311	1903174,653	562684,5783
Q53EP0	FNDC3B	Fibronectin type III domain-containing protein 3B OS=Homo sapiens OX=9606 GN=FNDC3B PE=1	2	3,017	1,359	3,983	379977,2634	806782,9083	224708,6212
P14618	PKM	Pyruvate kinase PKM OS=Homo sapiens OX=9606 GN=PKM PE=1 SV=4	27	2,435	1,672	3,97	369113213,9	787968050,5	228106654,6
Q9UNI6	DUSP12	Dual specificity protein phosphatase 12 OS=Homo sapiens OX=9606 GN=DUSP12 PE=1 SV=1	2	0,75	5,805	3,942	660113,2719	464999,9656	61672,60962
Q96AC1	FERMT2	Fermitin family homolog 2 OS=Homo sapiens OX=9606 GN=FERMT2 PE=1 SV=1	9	1,102	3,649	3,928	5734145,938	5601163,988	885087,9323
P18206	VCL	Vinculin OS=Homo sapiens OX=9606 GN=VCL PE=1 SV=4	42	3,418	1,196	3,911	64089686,01	202749268,7	56491093,08
O94925	GLS	Glutaminase kidney isoform, mitochondrial OS=Homo sapiens OX=9606 GN=GLS PE=1 SV=1	9	3,367	1,195	3,91	7820516,625	24463052,57	6530137,889
Q86YB8	ERO1B	ERO1-like protein beta OS=Homo sapiens OX=9606 GN=ERO1B PE=1 SV=2	3	0,953	4,15	3,906	156543,3704	149127,5742	38178,91611
P09525	ANXA4	Annexin A4 OS=Homo sapiens OX=9606 GN=ANXA4 PE=1 SV=4	12	6,688	0,569	3,894	2418489,783	17469320,39	4448722,125
Q9NVD7	PARVA	Alpha-parvin OS=Homo sapiens OX=9606 GN=PARVA PE=1 SV=1	4	1,934	2,228	3,888	2898742,197	4372052,545	1478941,446
O95858	TSPAN15	Tetraspanin-15 OS=Homo sapiens OX=9606 GN=TSPAN15 PE=1 SV=1	3	1000	0,001	3,873		3269633,017	889902,5371
Q9Y2G5	POFUT2	GDP-fucose protein O-fucosyltransferase 2 OS=Homo sapiens OX=9606 GN=POFUT2 PE=1 SV=3	3	1,179	3,21	3,871	1123113,632	984312,3792	166601,9586
Q15274	QPRT	Nicotinate-nucleotide pyrophosphorylase [carboxylating] OS=Homo sapiens OX=9606 GN=QPRT	3	0,283	12,311	3,855	6264526,003	1724837,943	707649,4263
O95479	H6PD	GDH/6PGL endoplasmic bifunctional protein OS=Homo sapiens OX=9606 GN=H6PD PE=1 SV=2	4	5,653	0,425	3,845	138104,8356	2385260,702	406250,6842
Q4G148	GXYLT1	Glucoside xylosyltransferase 1 OS=Homo sapiens OX=9606 GN=GXYLT1 PE=1 SV=2	2	1,022	3,744	3,825	107516,0918	109849,9508	28717,31795
O43847	NRDC	Nardilysin OS=Homo sapiens OX=9606 GN=NRDC PE=1 SV=3	6	0,555	5,424	3,824	3138335,906	2134064,833	812742,5869
Q9HAW8	UGT1A10	UDP-glucuronosyltransferase 1A10 OS=Homo sapiens OX=9606 GN=UGT1A10 PE=1 SV=1	3	1,795	2,128	3,819	219581,7115	389196,996	270671,7083
P26640	VARS1	Valine--tRNA ligase OS=Homo sapiens OX=9606 GN=VARS1 PE=1 SV=4	21	0,439	8,117	3,819	30979153,69	16655591,56	2981088,892
O60218	AKR1B10	Aldo-keto reductase family 1 member B10 OS=Homo sapiens OX=9606 GN=AKR1B10 PE=1 SV=2	7	10,329	0,454	3,814	737977,7028	14300642,79	1482633,818
P09104	ENO2	Gamma-enolase OS=Homo sapiens OX=9606 GN=ENO2 PE=1 SV=3	7	0,806	11,044	3,809	5173416,354	4344728,976	132164,5822
P16070	CD44	CD44 antigen OS=Homo sapiens OX=9606 GN=CD44 PE=1 SV=3	8	58,416	0,083	3,779	1616635,96	99551066,66	24438738,27
Q09666	AHNAK	Neuroblast differentiation-associated protein AHNAK OS=Homo sapiens OX=9606 GN=AHNAK PE	203	7,13	0,555	3,765	134397691,9	953589597	255212753
P61163	ACTR1A	Alpha-centractin OS=Homo sapiens OX=9606 GN=ACTR1A PE=1 SV=1	5	0,924	3,9	3,765	13150686,08	11430005,94	3663471,729
Q08431	MFGE8	Lactadherin OS=Homo sapiens OX=9606 GN=MFGE8 PE=1 SV=3	4	0,816	4,479	3,752	2159962,761	1421872,335	170546,0539
P49903	SEPHS1	Selenide, water dikinase 1 OS=Homo sapiens OX=9606 GN=SEPHS1 PE=1 SV=2	2	0,62	6,193	3,747	1455910,836	871323,2463	222134,9688
P30041	PRDX6	Peroxiredoxin-6 OS=Homo sapiens OX=9606 GN=PRDX6 PE=1 SV=3	14	2,286	1,514	3,746	51776072,81	78233562,97	34263597,91
Q6NYC8	PPP1R18	Phostensin OS=Homo sapiens OX=9606 GN=PPP1R18 PE=1 SV=1	8	2,311	1,607	3,737	3075804,17	8638284,323	2028610,916
P29218	IMPA1	Inositol monophosphatase 1 OS=Homo sapiens OX=9606 GN=IMPA1 PE=1 SV=1	3	0,669	5,806	3,727	2429909,423	1621844,377	174517,3825
O15067	PFAS	Phosphoribosylformylglycinamidine synthase OS=Homo sapiens OX=9606 GN=PFAS PE=1 SV=4	13	0,311	14,321	3,725	19253887,62	6656088,078	1838869,366
Q8TD43	TRPM4	Transient receptor potential cation channel subfamily M member 4 OS=Homo sapiens OX=9606	6	2,207	1,607	3,719	128722,1189	459961,3023	621655,5676
Q8TCB0	IFI44	Interferon-induced protein 44 OS=Homo sapiens OX=9606 GN=IFI44 PE=1 SV=2	2	3,202	1,119	3,717	143583,0196	1301183,763	123668,2008
Q13620	CUL4B	Cullin-4B OS=Homo sapiens OX=9606 GN=CUL4B PE=1 SV=4	6	2,412	1,674	3,702	4942969,117	10402413,49	2294899,125
O15212	PFDN6	Prefoldin subunit 6 OS=Homo sapiens OX=9606 GN=PFDN6 PE=1 SV=1	3	0,639	5,065	3,7	7687820,89	4606038,455	1620011,579
O95870	ABHD16A	Phosphatidylserine lipase ABHD16A OS=Homo sapiens OX=9606 GN=ABHD16A PE=1 SV=3	6	1,22	3,025	3,684	2634675,642	3461100,124	860657,3653

Q92930	RAB8B	Ras-related protein Rab-8B OS=Homo sapiens OX=9606 GN=RAB8B PE=1 SV=2	2	4,145	0,885	3,681	1816744,586	7337253,62	1741897,083
Q9H299	SH3BGRL3	SH3 domain-binding glutamic acid-rich-like protein 3 OS=Homo sapiens OX=9606 GN=SH3BGRL3	3	2,889	2,302	3,669	5600801,051	14923263,45	2880102,114
P49773	HINT1	Adenosine 5'-monophosphoramidase HINT1 OS=Homo sapiens OX=9606 GN=HINT1 PE=1 SV=2	3	0,837	1,633	3,657	11525809,71	10065642,07	4492529,354
O75955	FLOT1	Flotillin-1 OS=Homo sapiens OX=9606 GN=FLOT1 PE=1 SV=3	14	1,441	2,361	3,647	12378351,05	17943268,03	4845662,452
O60925	PFDN1	Prefoldin subunit 1 OS=Homo sapiens OX=9606 GN=PFDN1 PE=1 SV=2	2	0,587	6,176	3,644	4588023,276	2693137,722	898869,3594
A1L020	MEX3A	RNA-binding protein MEX3A OS=Homo sapiens OX=9606 GN=MEX3A PE=1 SV=1	4	0,249	15,559	3,638	1804049,34	140724,9561	34214,60871
P54687	BCAT1	Branched-chain-amino-acid aminotransferase, cytosolic OS=Homo sapiens OX=9606 GN=BCAT1 I	4	0,224	16,87	3,632	7597979,456	1240835,582	533147,7065
Q16706	MAN2A1	Alpha-mannosidase 2 OS=Homo sapiens OX=9606 GN=MAN2A1 PE=1 SV=2	7	3,948	0,887	3,625	1271616,297	6990243,029	1315452,696
Q70UQ0	IKBIP	Inhibitor of nuclear factor kappa-B kinase-interacting protein OS=Homo sapiens OX=9606 GN=IK	10	4,296	0,831	3,616	3654010,294	17861045,27	4776474,134
Q15942	ZYX	Zyxin OS=Homo sapiens OX=9606 GN=ZYX PE=1 SV=1	6	2,317	1,312	3,611	5490672,989	7532292,86	4305676,725
O15438	ABCC3	ATP-binding cassette sub-family C member 3 OS=Homo sapiens OX=9606 GN=ABCC3 PE=1 SV=3	8	1000	0,001	3,606		2003455,888	732069,3192
P07108	DBI	Acyl-CoA-binding protein OS=Homo sapiens OX=9606 GN=DBI PE=1 SV=2	5	0,519	4,073	3,601	9775549,457	4845521,971	4086881,412
P80723	BASP1	Brain acid soluble protein 1 OS=Homo sapiens OX=9606 GN=BASP1 PE=1 SV=2	11	1,788	2,223	3,586	10975890,12	20817539,17	4715297,3
Q9UEU0	VTI1B	Vesicle transport through interaction with t-SNAREs homolog 1B OS=Homo sapiens OX=9606 GN	2	1000	0,001	3,584		707199,9011	197295,2657
Q92604	LPGAT1	Acyl-CoA:lysophosphatidylglycerol acyltransferase 1 OS=Homo sapiens OX=9606 GN=LPGAT1 PE=	4	1,765	1,891	3,583	833861,029	1463383,046	131719,3658
P11802	CDK4	Cyclin-dependent kinase 4 OS=Homo sapiens OX=9606 GN=CDK4 PE=1 SV=2	6	0,371	7,389	3,576	5666304,095	2043215,565	630428,7736
P05556	ITGB1	Integrin beta-1 OS=Homo sapiens OX=9606 GN=ITGB1 PE=1 SV=2	11	6,891	0,512	3,571	30266229,94	195189669,3	57634200,3
Q86TX2	ACOT1	Acyl-coenzyme A thioesterase 1 OS=Homo sapiens OX=9606 GN=ACOT1 PE=1 SV=1	11	2,429	1,424	3,558	3101223,472	6173837,937	1569453,462
P30101	PDIA3	Protein disulfide-isomerase A3 OS=Homo sapiens OX=9606 GN=PDIA3 PE=1 SV=4	30	1,377	2,39	3,539	409034403,5	573378025,8	176602062,9
Q9NZ45	CISD1	CDGSH iron-sulfur domain-containing protein 1 OS=Homo sapiens OX=9606 GN=CISD1 PE=1 SV=	3	4,049	1,099	3,531	2603134,439	10658340,56	2296760,178
O14657	TOR1B	Torsin-1B OS=Homo sapiens OX=9606 GN=TOR1B PE=1 SV=2	3	4,946	0,715	3,488	187714,776	1771088,519	580427,0316
Q5D862	FLG2	Filaggrin-2 OS=Homo sapiens OX=9606 GN=FLG2 PE=1 SV=1	3	3,136	1,145	3,475	920456,2335	3159702,502	947246,0736
Q8N8S7	ENAH	Protein enabled homolog OS=Homo sapiens OX=9606 GN=ENAH PE=1 SV=2	7	1,078	3,158	3,464	4557553,882	3570897,127	1237584,269
Q13425	SNTB2	Beta-2-syntrophin OS=Homo sapiens OX=9606 GN=SNTB2 PE=1 SV=1	4	5,508	0,699	3,437	675227,2219	3700945,491	1089082,7
Q9HB40	SCPEP1	Retinoid-inducible serine carboxypeptidase OS=Homo sapiens OX=9606 GN=SCPEP1 PE=1 SV=1	7	1,512	2,106	3,435	2437277,038	1227791,588	320741,917
P14735	IDE	Insulin-degrading enzyme OS=Homo sapiens OX=9606 GN=IDE PE=1 SV=4	7	1,009	3,651	3,431	5498648,549	4395310,854	1379914,817
Q8IVL5	P3H2	Prolyl 3-hydroxylase 2 OS=Homo sapiens OX=9606 GN=P3H2 PE=1 SV=1	8	14,224	0,122	3,429	478339,912	8024000,484	6912313,848
P28062	PSMB8	Proteasome subunit beta type-8 OS=Homo sapiens OX=9606 GN=PSMB8 PE=1 SV=3	3	41,389	0,113	3,424	200685,1571	10520829,05	2269598,478
P20591	MX1	Interferon-induced GTP-binding protein Mx1 OS=Homo sapiens OX=9606 GN=MX1 PE=1 SV=4	27	10,262	0,337	3,424	27553628,62	277482899,4	91046233,58
P28065	PSMB9	Proteasome subunit beta type-9 OS=Homo sapiens OX=9606 GN=PSMB9 PE=1 SV=2	5	52,231	0,075	3,421	115209,6434	17273226,24	5205235,754
Q14CN4	KRT72	Keratin, type II cytoskeletal 72 OS=Homo sapiens OX=9606 GN=KRT72 PE=1 SV=2	3	1,389	2,658	3,405	2569344,734	3568858,149	3673798,501
P07195	LDHB	L-lactate dehydrogenase B chain OS=Homo sapiens OX=9606 GN=LDHB PE=1 SV=2	16	0,303	9,268	3,396	296807299,7	94034031,56	32153779,88
P06753	TPM3	Tropomyosin alpha-3 chain OS=Homo sapiens OX=9606 GN=TPM3 PE=1 SV=2	5	2,401	1,306	3,394	37150650,93	80793297,27	28730869,1
Q9UIQ6	LNPEP	Leucyl-cystinyl aminopeptidase OS=Homo sapiens OX=9606 GN=LNPEP PE=1 SV=3	18	8,079	0,496	3,393	5598385,492	29136545,22	9726302,639
P18085	ARF4	ADP-ribosylation factor 4 OS=Homo sapiens OX=9606 GN=ARF4 PE=1 SV=3	5	1,438	1,767	3,387	41222355,03	54392649,63	20842908,65
Q15165	PON2	Serum paraoxonase/arylesterase 2 OS=Homo sapiens OX=9606 GN=PON2 PE=1 SV=4	7	1,551	2,177	3,384	3664547,323	5464502,093	1544284,392
P01040	CSTA	Cystatin-A OS=Homo sapiens OX=9606 GN=CSTA PE=1 SV=1	2	3,239	1,02	3,379	444413,6934	1522881,301	466465,6181
Q9HAU0	PLEKHA5	Pleckstrin homology domain-containing family A member 5 OS=Homo sapiens OX=9606 GN=PLE	6	2,073	1,757	3,345	896101,5248	1775955,878	164875,2821
Q9Y617	PSAT1	Phosphoserine aminotransferase OS=Homo sapiens OX=9606 GN=PSAT1 PE=1 SV=2	10	1,062	3,486	3,336	17434340,51	17228378,84	4461222,198
P13674	P4HA1	Prolyl 4-hydroxylase subunit alpha-1 OS=Homo sapiens OX=9606 GN=P4HA1 PE=1 SV=2	14	2,452	1,419	3,332	13595893,85	35979989	10680626,94
Q99439	CNN2	Calponin-2 OS=Homo sapiens OX=9606 GN=CNN2 PE=1 SV=4	6	1,716	1,877	3,33	10754370,28	19728020,11	6094527,195
Q12841	FSTL1	Follistatin-related protein 1 OS=Homo sapiens OX=9606 GN=FSTL1 PE=1 SV=1	3	0,77	4,861	3,325	1620260,144	930149,9826	310526,0238
O60488	ACSL4	Long-chain-fatty-acid--CoA ligase 4 OS=Homo sapiens OX=9606 GN=ACSL4 PE=1 SV=2	12	2,475	1,428	3,315	5486795,273	13158422,26	4602330,785
P07602	PSAP	Prosaposin OS=Homo sapiens OX=9606 GN=PSAP PE=1 SV=2	4	2,552	1,241	3,309	1402058,79	3565060,435	1496618,41
Q8WWQ0	PHIP	PH-interacting protein OS=Homo sapiens OX=9606 GN=PHIP PE=1 SV=2	4	1,186	1,026	3,3	798336,0267	1030407,812	1110492,688
Q9BRQ8	AIFM2	Ferroptosis suppressor protein 1 OS=Homo sapiens OX=9606 GN=AIFM2 PE=1 SV=1	5	1000	0,001	3,285		3861319,912	53653,49075
P60660	MYL6	Myosin light polypeptide 6 OS=Homo sapiens OX=9606 GN=MYL6 PE=1 SV=2	8	3,548	1,238	3,269	26721744,05	84885802,98	30865610,34
O60271	SPAG9	C-Jun-amino-terminal kinase-interacting protein 4 OS=Homo sapiens OX=9606 GN=SPAG9 PE=1 S	17	1,397	2,357	3,267	4735556,94	5394838,617	1445847,909
Q96JJ3	ELMO2	Engulfment and cell motility protein 2 OS=Homo sapiens OX=9606 GN=ELMO2 PE=1 SV=2	3	0,757	3,806	3,264	1077438,256	565471,2457	425985,3443
Q9BZF1	OSBPL8	Oxysterol-binding protein-related protein 8 OS=Homo sapiens OX=9606 GN=OSBPL8 PE=1 SV=3	7	0,866	3,582	3,257	2154476,976	1982472,353	394120,01
Q9UHD9	UBQLN2	Ubiquilin-2 OS=Homo sapiens OX=9606 GN=UBQLN2 PE=1 SV=2	4	0,618	4,715	3,25	4121110,492	2091049,64	1047879,819
Q13564	NAE1	NEDD8-activating enzyme E1 regulatory subunit OS=Homo sapiens OX=9606 GN=NAE1 PE=1 SV=	3	1,024	2,913	3,246	1363482,191	1295448,402	334352,3124
Q9ULC5	ACSL5	Long-chain-fatty-acid--CoA ligase 5 OS=Homo sapiens OX=9606 GN=ACSL5 PE=1 SV=1	14	17,123	0,161	3,245	394461,6447	8976013,906	2138044,248
P55735	SEC13	Protein SEC13 homolog OS=Homo sapiens OX=9606 GN=SEC13 PE=1 SV=3	4	2,315	1,35	3,244	2108721,116	4603129,013	1754855,068
O15460	P4HA2	Prolyl 4-hydroxylase subunit alpha-2 OS=Homo sapiens OX=9606 GN=P4HA2 PE=1 SV=1	12	6,156	0,546	3,243	4580130,845	30072060,27	10604386,9
P15559	NQO1	NAD(P)H dehydrogenase [quinone] 1 OS=Homo sapiens OX=9606 GN=NQO1 PE=1 SV=1	7	2,385	1,283	3,233	15794904,15	36395577,18	10301222,94
E9PFN4	SLC4A7	Anion exchange protein OS=Homo sapiens OX=9606 GN=SLC4A7 PE=1 SV=1	2	0,909	3,539	3,216	1405711,441	1248839,228	362906,876
P08651	NFIC	Nuclear factor 1 C-type OS=Homo sapiens OX=9606 GN=NFIC PE=1 SV=2	3	5,619	0,522	3,202	438235,1391	2378146,082	1085445,365
P28370	SMARCA1	Probable global transcription activator SNF2L1 OS=Homo sapiens OX=9606 GN=SMARCA1 PE=1 S	3	0,579	7,506	3,2	1835749,419	1336197,451	153116,2661
O43175	PHGDH	D-3-phosphoglycerate dehydrogenase OS=Homo sapiens OX=9606 GN=PHGDH PE=1 SV=4	12	0,425	7,112	3,188	140615948,5	60026042,68	20803892,16
A0A0U1RRM6	ENAH	ENAH actin regulator OS=Homo sapiens OX=9606 GN=ENAH PE=1 SV=1	5	0,443	5,557	3,179	4022561,153	2411338,594	858598,8026
Q9BSJ8	ESYT1	Extended synaptotagmin-1 OS=Homo sapiens OX=9606 GN=ESYT1 PE=1 SV=1	28	2,715	1,296	3,176	17305225,48	46739829,94	12732197,45
P18669	PGAM1	Phosphoglycerate mutase 1 OS=Homo sapiens OX=9606 GN=PGAM1 PE=1 SV=2	10	0,828	3,52	3,175	75109203,38	62024677,76	31210650,09
P49023	PXN	Paxillin OS=Homo sapiens OX=9606 GN=PXN PE=1 SV=3	8	2,541	0,96	3,173	3921135,194	10406209,55	4868956,916
Q7Z2K6	ERMP1	Endoplasmic reticulum metallopeptidase 1 OS=Homo sapiens OX=9606 GN=ERMP1 PE=1 SV=2	19	12,958	0,273	3,163	2957976,203	35592132,73	10205760,78
Q5NDL2	EOGT	EGF domain-specific O-linked N-acetylglucosamine transferase OS=Homo sapiens OX=9606 GN=I	2	3,931	0,861	3,157	118944,3816	467613,8967	150282,2377
O14773	TPP1	Tripeptidyl-peptidase 1 OS=Homo sapiens OX=9606 GN=TPP1 PE=1 SV=2	6	3,586	0,966	3,15	3677076,39	13219051,16	3957715,051
P23743	DGKA	Diacylglycerol kinase alpha OS=Homo sapiens OX=9606 GN=DGKA PE=1 SV=3	5	2,69	0,982	3,134	171556,401	281071,2526	82585,6744
O14770	MEIS2	Homeobox protein Meis2 OS=Homo sapiens OX=9606 GN=MEIS2 PE=1 SV=2	3	0,579	6,524	3,132	1284676,714	878420,7427	47604,68039
Q03169	TNFAIP2	Tumor necrosis factor alpha-induced protein 2 OS=Homo sapiens OX=9606 GN=TNFAIP2 PE=1 SV	18	3,923		3,124	1037049,988	6904613,71	661495,7442
Q4KMQ2	ANO6	Anoctamin-6 OS=Homo sapiens OX=9606 GN=ANO6 PE=1 SV=2	4	2,576	1,097	3,112	467933,3574	2258577,398	340216,4417
P49588	AARS1	Alanine--tRNA ligase, cytoplasmic OS=Homo sapiens OX=9606 GN=AARS1 PE=1 SV=2	24	1,618	2,03	3,094	23968777,69	35654508,75	11803855,04
Q9P0K7	RAI14	Ankycorbin OS=Homo sapiens OX=9606 GN=RAI14 PE=1 SV=2	7	2,251	1,35	3,091	2264614,999	4773503,785	1983677,54
A6NDU8	RIMOC1	RAB7A-interacting MON1-CCZ1 complex subunit 1 OS=Homo sapiens OX=9606 GN=RIMOC1 PE=	2	0,487	6,377	3,088	804365,5357	317560,6521	120552,0636
Q9BWM7	SFXN3	Sideroflexin-3 OS=Homo sapiens OX=9606 GN=SFXN3 PE=1 SV=3	8	9,579	0,282	3,084	1642234,124	20876774,74	6608573,498
Q13423	NNT	NAD(P) transhydrogenase, mitochondrial OS=Homo sapiens OX=9606 GN=NNT PE=1 SV=3	23	2,134	1,403	3,082	36583699,56	68959005,39	25553418,33
P55809	OXCT1	Succinyl-CoA:3-ketoacid coenzyme A transferase 1, mitochondrial OS=Homo sapiens OX=9606 GI	14	2,901	1,043	3,078	18461888,83	52242907,92	15078877,81
Q9UNH7	SNX6	Sorting nexin-6 OS=Homo sapiens OX=9606 GN=SNX6 PE=1 SV=1	8	1,212	2,337	3,075	4477034,078	5928946,77	2530119,604
Q9BTW9	TBCD	Tubulin-specific chaperone D OS=Homo sapiens OX=9606 GN=TBCD PE=1 SV=2	10	0,502	5,372	3,068	7427083,358	12548865,94	3388855,016
Q99471	PFDN5	Prefoldin subunit 5 OS=Homo sapiens OX=9606 GN=PFDN5 PE=1 SV=2	7	0,43	5,899	3,058	13435841,2	6231798,765	1988280,671
Q8NEW0	SLC30A7	Zinc transporter 7 OS=Homo sapiens OX=9606 GN=SLC30A7 PE=1 SV=1	2	3,091	1,369	3,054	718990,4389	2359586,141	784618,9644
Q8IY17	PNPLA6	Patatin-like phospholipase domain-containing protein 6 OS=Homo sapiens OX=9606 GN=PNPLA6	4	3,342	0,968	3,053	164665,0476	670226,3499	168378,4545
P11047	LAMC1	Laminin subunit gamma-1 OS=Homo sapiens OX=9606 GN=LAMC1 PE=1 SV=3	8	1,174	2,708	3,05	8697430,432	10772642,86	2966573,655
P09496	CLTA	Clathrin light chain A OS=Homo sapiens OX=9606 GN=CLTA PE=1 SV=1	5	1,59	1,89	3,049	12557062,47	19608806,13	6960767,475
P04150	NR3C1	Glucocorticoid receptor OS=Homo sapiens OX=9606 GN=NR3C1 PE=1 SV=1	2	1000	0,001	3,041		546401,8027	148782,5419
Q709C8	VPS13C	Intermembrane lipid transfer protein VPS13C OS=Homo sapiens OX=9606 GN=VPS13C PE=1 SV=	5	0,544	2,215	3,041	466838,5804	255257,0835	99685,16599
Q02809	PLOD1	Procollagen-lysine,2-oxoglutarate 5-dioxygenase 1 OS=Homo sapiens OX=9606 GN=PLOD1 PE=1	19	2,502	1,013	3,038	14122625,21	33599292,93	15328688,28
Q6UW02	CYP20A1	Cytochrome P450 20A1 OS=Homo sapiens OX=9606 GN=CYP20A1 PE=1 SV=1	6	1,455	1,731	3,037	1425580,376	1992142,036	574283,1992
Q9NWM8	FKBP14	Peptidyl-prolyl cis-trans isomerase FKBP14 OS=Homo sapiens OX=9606 GN=FKBP14 PE=1 SV=1	2	0,93	2,065	3,037	923932,4297	1002366,752	222238,5759
P15121	AKR1B1	Aldo-keto reductase family 1 member B1 OS=Homo sapiens OX=9606 GN=AKR1B1 PE=1 SV=3	11	5,855	0,533	3,036	11983982,03	60685890,97	25446231,24
Q96HC4	PDLIM5	PDZ and LIM domain protein 5 OS=Homo sapiens OX=9606 GN=PDLIM5 PE=1 SV=5	14	3,859	1,013	3,022	9594273,19	24090575,24	10117915,61
Q9NRX5	SERINC1	Serine incorporator 1 OS=Homo sapiens OX=9606 GN=SERINC1 PE=1 SV=1	2	1,765	1,679	3,021	419576,8777	825773,4071	319546,3999
Q9BTT6	LRRC1	Leucine-rich repeat-containing protein 1 OS=Homo sapiens OX=9606 GN=LRRC1 PE=1 SV=1	6	6,783	0,443	3,02	157792,9788	1590737,482	598542,3386
Q14699	RFTN1	Raftlin OS=Homo sapiens OX=9606 GN=RFTN1 PE=1 SV=4	3	5,053	0,382	3,017	220687,3391	2464495,329	769105,37
P46939	UTRN	Utrophin OS=Homo sapiens OX=9606 GN=UTRN PE=1 SV=2	15	3,604	1,066	3,005	1908772,623	9280128,761	1851463,261
Q96CX2	KCTD12	BTB/POZ domain-containing protein KCTD12 OS=Homo sapiens OX=9606 GN=KCTD12 PE=1 SV=1	5	1,657	1,976	3,002	2121535,94	3399221,62	819261,9558
P18084	ITGB5	Integrin beta-5 OS=Homo sapiens OX=9606 GN=ITGB5 PE=1 SV=1	2	2,128	1,366	3,001	265993,2422	879589,6453	195419,8
A0A669KBH5	SNCA	Alpha-synuclein OS=Homo sapiens OX=9606 GN=SNCA PE=1 SV=1	5	3,626	2,696	2,975	2541846,514	6806783,599	965073,8666
P31150	GDI1	Rab GDP dissociation inhibitor alpha OS=Homo sapiens OX=9606 GN=GDI1 PE=1 SV=2	5	0,629	5,764	2,958	11432592,38	6173522,932	1944844,476
A0A0J9YXF2	PON2	Paraoxonase OS=Homo sapiens OX=9606 GN=PON2 PE=1 SV=1	5	2,111	1,361	2,955	2299159,716	4247753,999	1191532,861
P42330	AKR1C3	Aldo-keto reductase family 1 member C3 OS=Homo sapiens OX=9606 GN=AKR1C3 PE=1 SV=4	7	2,604	2,68	2,949	2716052,111	8535558,683	755185,7093
P35908	KRT2	Keratin, type II cytoskeletal 2 epidermal OS=Homo sapiens OX=9606 GN=KRT2 PE=1 SV=2	26	2,136	1,422	2,948	125441165,7	266870111,3	95057207,06
P30622	CLIP1	CAP-Gly domain-containing linker protein 1 OS=Homo sapiens OX=9606 GN=CLIP1 PE=1 SV=2	6	1,451	1,405	2,948	879843,6941	3658127,336	1066865,701
P54802	NAGLU	Alpha-N-acetylglucosaminidase OS=Homo sapiens OX=9606 GN=NAGLU PE=1 SV=2	3	6,057	0,465	2,941	41988,668	328370,1203	97691,0346
Q03135	CAV1	Caveolin-1 OS=Homo sapiens OX=9606 GN=CAV1 PE=1 SV=4	6	26,078	0,113	2,937	2117705,827	65081314,33	20244127,46
Q9UER7	DAXX	Death domain-associated protein 6 OS=Homo sapiens OX=9606 GN=DAXX PE=1 SV=2	2	0,676	3,574	2,934	925213,9306	611984,9974	138900,5796
P17655	CAPN2	Calpain-2 catalytic subunit OS=Homo sapiens OX=9606 GN=CAPN2 PE=1 SV=6	10	5,889	0,556	2,93	1013809,362	8200274,684	2578064,361
P49721	PSMB2	Proteasome subunit beta type-2 OS=Homo sapiens OX=9606 GN=PSMB2 PE=1 SV=1	5	1,742	1,835	2,924	11609917,68	11135061,4	6178009,547
O15164	TRIM24	Transcription intermediary factor 1-alpha OS=Homo sapiens OX=9606 GN=TRIM24 PE=1 SV=3	4	0,278	5,624	2,923	3482690,861	1027148,975	549734,4068
Q12797	ASPH	Aspartyl/asparaginyl beta-hydroxylase OS=Homo sapiens OX=9606 GN=ASPH PE=1 SV=3	24	3,33	0,863	2,92	15367655,75	45800066,29	16547930,76
Q13438	OS9	Protein OS-9 OS=Homo sapiens OX=9606 GN=OS9 PE=1 SV=1	5	2,175	1,23	2,912	1199796,937	3019865,078	1137674,713

P48506	GCLC	Glutamate--cysteine ligase catalytic subunit OS=Homo sapiens OX=9606 GN=GCLC PE=1 SV=2	6	0,718	6,77	2,895	2105407,697	1181063,039	266642,3118
P33897	ABCD1	ATP-binding cassette sub-family D member 1 OS=Homo sapiens OX=9606 GN=ABCD1 PE=1 SV=2	3	2,305	1,312	2,884	1180283,243	2861343,996	898167,3476
P08758	ANXA5	Annexin A5 OS=Homo sapiens OX=9606 GN=ANXA5 PE=1 SV=2	13	1,194	2,732	2,884	64764609,95	76792810,41	23666196,35
P02794	FTH1	Ferritin heavy chain OS=Homo sapiens OX=9606 GN=FTH1 PE=1 SV=2	3	0,806	5,128	2,88	781412,8485	728581,0289	145250,0816
Q15363	TMED2	Transmembrane emp24 domain-containing protein 2 OS=Homo sapiens OX=9606 GN=TMED2 PE	5	1,244	2,359	2,878	12881402,91	15909449,26	4549580,655
Q5ZPR3	CD276	CD276 antigen OS=Homo sapiens OX=9606 GN=CD276 PE=1 SV=1	3	1,078	2,678	2,878	4760712,372	5143992,524	1796186,578
O14879	IFIT3	Interferon-induced protein with tetratricopeptide repeats 3 OS=Homo sapiens OX=9606 GN=IFIT	10	5,775	0,485	2,87	4357030,18	27740500,73	12230219,19
P00167	CYB5A	Cytochrome b5 OS=Homo sapiens OX=9606 GN=CYB5A PE=1 SV=2	5	2,845	0,997	2,867	5791361,456	16477668,77	5597291,46
Q8N1G2	CMTR1	Cap-specific mRNA (nucleoside-2'-O-)-methyltransferase 1 OS=Homo sapiens OX=9606 GN=CMT	2	1,458	1,976	2,866	490157,5663	521268,7061	183639,0213
Q16527	CSRP2	Cysteine and glycine-rich protein 2 OS=Homo sapiens OX=9606 GN=CSRP2 PE=1 SV=3	5	0,157	10,63	2,856	11844888,78	1949157,705	825594,1041
P01024	C3	Complement C3 OS=Homo sapiens OX=9606 GN=C3 PE=1 SV=2	9	3,539	0,286	2,854	148746,7554	3282091,635	1017127,088
Q9NVM9	INTS13	Integrator complex subunit 13 OS=Homo sapiens OX=9606 GN=INTS13 PE=1 SV=2	2	0,341	4,083	2,853	1952769,64	1207083,418	600831,1263
Q13443	ADAM9	Disintegrin and metalloproteinase domain-containing protein 9 OS=Homo sapiens OX=9606 GN=	3	7,814	0,366	2,845	234843,377	2047106,504	1029735,658
P11171	EPB41	Protein 4.1 OS=Homo sapiens OX=9606 GN=EPB41 PE=1 SV=4	6	0,417	6,053	2,822	4369984,342	1634786,92	310898,83
Q13126	MTAP	S-methyl-5'-thioadenosine phosphorylase OS=Homo sapiens OX=9606 GN=MTAP PE=1 SV=2	5	1,011	2,938	2,82	3839063,034	3708396,69	1274066,127
P55011	SLC12A2	Solute carrier family 12 member 2 OS=Homo sapiens OX=9606 GN=SLC12A2 PE=1 SV=1	11	1,293	2,075	2,815	4322736,394	5038701,564	3009520,908
P04066	FUCA1	Tissue alpha-L-fucosidase OS=Homo sapiens OX=9606 GN=FUCA1 PE=1 SV=4	4	3,281	0,873	2,813	1830794,739	4356221,694	1441549,989
P21291	CSRP1	Cysteine and glycine-rich protein 1 OS=Homo sapiens OX=9606 GN=CSRP1 PE=1 SV=3	5	2,557	1,206	2,811	7705444,399	20077583,29	6381902,945
Q9BRR6	ADPGK	ADP-dependent glucokinase OS=Homo sapiens OX=9606 GN=ADPGK PE=1 SV=1	3	2,04	1,348	2,811	1696318,384	3210165,09	572814,9067
P55209	NAP1L1	Nucleosome assembly protein 1-like 1 OS=Homo sapiens OX=9606 GN=NAP1L1 PE=1 SV=1	8	0,307	8,387	2,806	73551223,07	32060816,54	9517477,583
Q9NR31	SAR1A	GTP-binding protein SAR1a OS=Homo sapiens OX=9606 GN=SAR1A PE=1 SV=1	3	1,109	2,256	2,803	12549687,85	24091352,96	10388968,3
Q13325	IFIT5	Interferon-induced protein with tetratricopeptide repeats 5 OS=Homo sapiens OX=9606 GN=IFIT	4	2,911	1,212	2,799	697852,7469	2686326,09	767644,2616
Q13454	TUSC3	Tumor suppressor candidate 3 OS=Homo sapiens OX=9606 GN=TUSC3 PE=1 SV=1	2	0,679	2,73	2,797	966459,9661	714081,6343	457092,7516
Q12884	FAP	Prolyl endopeptidase FAP OS=Homo sapiens OX=9606 GN=FAP PE=1 SV=5	3	1000	0,001	2,794		1876645,169	441868,545
Q3SXM5	HSDL1	Inactive hydroxysteroid dehydrogenase-like protein 1 OS=Homo sapiens OX=9606 GN=HSDL1 PE	2	0,366	7,527	2,791	323991,848	118702,4435	43041,67436
Q9Y2B0	CNPY2	Protein canopy homolog 2 OS=Homo sapiens OX=9606 GN=CNPY2 PE=1 SV=1	6	1,5	1,84	2,784	11731071,65	18220329,48	6244081,042
O00429	DNM1L	Dynamin-1-like protein OS=Homo sapiens OX=9606 GN=DNM1L PE=1 SV=2	14	0,786	3,213	2,778	5371305,532	3291630,969	1383529,49
P46063	RECQL	ATP-dependent DNA helicase Q1 OS=Homo sapiens OX=9606 GN=RECQL PE=1 SV=3	4	1,638	1,63	2,772	3210344,023	3367981,936	1321317,671
P60174	TPI1	Triosephosphate isomerase OS=Homo sapiens OX=9606 GN=TPI1 PE=1 SV=4	10	0,707	3,679	2,756	110656562,9	76500940,44	31197667,19
P30043	BLVRB	Flavin reductase (NADPH) OS=Homo sapiens OX=9606 GN=BLVRB PE=1 SV=3	4	4,499	0,458	2,754	1235992,265	6159025,993	2552781,449
P48960	ADGRE5	Adhesion G protein-coupled receptor E5 OS=Homo sapiens OX=9606 GN=ADGRE5 PE=1 SV=4	2	5,736	0,432	2,742	210608,682	1527135,572	531557,6675
P51812	RPS6KA3	Ribosomal protein S6 kinase alpha-3 OS=Homo sapiens OX=9606 GN=RPS6KA3 PE=1 SV=1	2	1,104	2,796	2,735	1577463,455	1591818,132	454925,6367
P31944	CASP14	Caspase-14 OS=Homo sapiens OX=9606 GN=CASP14 PE=1 SV=2	3	2,435	1,225	2,732	641417,7107	1587122,074	529490,9639
P35527	KRT9	Keratin, type I cytoskeletal 9 OS=Homo sapiens OX=9606 GN=KRT9 PE=1 SV=3	29	1,11	2,482	2,729	238208104,6	268251735,1	104045549,7
P08243	ASNS	Asparagine synthetase [glutamine-hydrolyzing] OS=Homo sapiens OX=9606 GN=ASNS PE=1 SV=4	11	0,84	3,17	2,72	8650305,728	6211270,616	2744490,186
P04080	CSTB	Cystatin-B OS=Homo sapiens OX=9606 GN=CSTB PE=1 SV=2	3	1,045	2,926	2,715	21006487,06	20674622,52	6995225
Q16134	ETFDH	Electron transfer flavoprotein-ubiquinone oxidoreductase, mitochondrial OS=Homo sapiens OX=	3	2,274	0,896	2,713	656332,9686	1813395,487	755989,0371
P48723	HSPA13	Heat shock 70 kDa protein 13 OS=Homo sapiens OX=9606 GN=HSPA13 PE=1 SV=1	5	0,749	2,803	2,713	3980776,725	3201658,193	1298446,442
Q8IZ83	ALDH16A1	Aldehyde dehydrogenase family 16 member A1 OS=Homo sapiens OX=9606 GN=ALDH16A1 PE=1	4	1,124	2,425	2,712	867314,5173	748915,1901	275714,447
P42771	CDKN2A	Cyclin-dependent kinase inhibitor 2A OS=Homo sapiens OX=9606 GN=CDKN2A PE=1 SV=2	3	0,782	3,699	2,712	6397075,279	4174862,408	1659005,972
A0A087X1N8	SERPINB6	Serpin B6 OS=Homo sapiens OX=9606 GN=SERPINB6 PE=1 SV=1	7	4,273	0,592	2,7	1747937,249	10156667,32	3066878,225
O00291	HIP1	Huntingtin-interacting protein 1 OS=Homo sapiens OX=9606 GN=HIP1 PE=1 SV=5	9	1,171	1,827	2,699	6363133,782	6325592,218	3197182,769
Q6NZI2	CAVIN1	Caveolae-associated protein 1 OS=Homo sapiens OX=9606 GN=CAVIN1 PE=1 SV=1	10	34,161	0,095	2,681	3378664,7	60687653,12	27248275,25
Q04760	GLO1	Lactoylglutathione lyase OS=Homo sapiens OX=9606 GN=GLO1 PE=1 SV=4	6	0,313	11,785	2,674	20802828,2	5985926,683	2073791,184
Q00765	REEP5	Receptor expression-enhancing protein 5 OS=Homo sapiens OX=9606 GN=REEP5 PE=1 SV=3	3	1,855	1,906	2,671	1009104,743	2058938,87	5137672,411
A0A804HL01	ADD1	Adducin 1 OS=Homo sapiens OX=9606 GN=ADD1 PE=1 SV=1	4	3,118	0,651	2,667	2129067,126	13304288,32	3315764,903
Q16798	ME3	NADP-dependent malic enzyme, mitochondrial OS=Homo sapiens OX=9606 GN=ME3 PE=1 SV=2	2	2,805	0,946	2,666	76647,59601	212897,1292	45937,21722
P20645	M6PR	Cation-dependent mannose-6-phosphate receptor OS=Homo sapiens OX=9606 GN=M6PR PE=1 S	3	1,566	4,832	2,662	4480833,172	6594015,562	1184842,638
Q16719	KYNU	Kynureninase OS=Homo sapiens OX=9606 GN=KYNU PE=1 SV=1	5	1000	0,001	2,661		1123303,511	64704,56822
Q99961	SH3GL1	Endophilin-A2 OS=Homo sapiens OX=9606 GN=SH3GL1 PE=1 SV=1	3	1,277	2,125	2,658	2237406,315	3003141,217	1246907,9
Q9Y394	DHRS7	Dehydrogenase/reductase SDR family member 7 OS=Homo sapiens OX=9606 GN=DHRS7 PE=1 SV	8	2,709	0,91	2,657	9116991,825	19906748,27	8864545,623
P19474	TRIM21	E3 ubiquitin-protein ligase TRIM21 OS=Homo sapiens OX=9606 GN=TRIM21 PE=1 SV=1	3	4,372	0,559	2,652	404517,8394	1607090,131	652515,0092
Q9Y6Y8	SEC23IP	SEC23-interacting protein OS=Homo sapiens OX=9606 GN=SEC23IP PE=1 SV=1	6	1,347	2,074	2,638	2917442,921	3601228,634	1446724,48
P09669	COX6C	Cytochrome c oxidase subunit 6C OS=Homo sapiens OX=9606 GN=COX6C PE=1 SV=2	4	0,941	2,007	2,636	8284094,394	7051090,086	10546699,14
Q9BRF8	CPPED1	Serine/threonine-protein phosphatase CPPED1 OS=Homo sapiens OX=9606 GN=CPPED1 PE=1 SV	3	1000	0,001	2,634		1011883,149	380691,4905
Q01518	CAP1	Adenylyl cyclase-associated protein 1 OS=Homo sapiens OX=9606 GN=CAP1 PE=1 SV=5	16	1,278	2,378	2,634	49505802,71	52373539,09	23699010,68
Q9HC35	EML4	Echinoderm microtubule-associated protein-like 4 OS=Homo sapiens OX=9606 GN=EML4 PE=1 S	5	0,531	4,956	2,628	1536284,557	586710,1087	180897,5938
P10301	RRAS	Ras-related protein R-Ras OS=Homo sapiens OX=9606 GN=RRAS PE=1 SV=1	2	16,243	0,145	2,625	214177,0759	3823824,933	1750231,975
Q9C0B1	FTO	Alpha-ketoglutarate-dependent dioxygenase FTO OS=Homo sapiens OX=9606 GN=FTO PE=1 SV=	2	0,285	4,648	2,623	255999,3893	104984,8692	36466,23762
P02042	HBD	Hemoglobin subunit delta OS=Homo sapiens OX=9606 GN=HBD PE=1 SV=2	2	1,638	1,691	2,622	3218014,99	4985769,598	1797052,006
O95219	SNX4	Sorting nexin-4 OS=Homo sapiens OX=9606 GN=SNX4 PE=1 SV=1	4	1,55	1,609	2,611	1247058,44	1587573,269	622134,4063
Q14738	PPP2R5D	Serine/threonine-protein phosphatase 2A 56 kDa regulatory subunit delta isoform OS=Homo sap	7	0,506	7,85	2,61	6988996,813	2898526,694	454077,366
P39059	COL15A1	Collagen alpha-1(XV) chain OS=Homo sapiens OX=9606 GN=COL15A1 PE=1 SV=2	2	1000	0,001	2,606		1069210,799	230659,1329
Q9NX55	HYPK	Huntingtin-interacting protein K OS=Homo sapiens OX=9606 GN=HYPK PE=1 SV=3	2	0,432	6,095	2,599	1144418,827	488079,8428	187774,9823
Q08378	GOLGA3	Golgin subfamily A member 3 OS=Homo sapiens OX=9606 GN=GOLGA3 PE=1 SV=2	9	1,417	2,012	2,596	2407185,814	4500655,717	734029,6366
Q8N884	CGAS	Cyclic GMP-AMP synthase OS=Homo sapiens OX=9606 GN=CGAS PE=1 SV=2	2	0,632	4,166	2,596	546969,3236	266934,2261	102829,4561
Q9NZJ9	NUDT4	Diphosphoinositol polyphosphate phosphohydrolase 2 OS=Homo sapiens OX=9606 GN=NUDT4 P	4	1,004	2,101	2,589	1378631,402	690575,7336	265210,8278
P07237	P4HB	Protein disulfide-isomerase OS=Homo sapiens OX=9606 GN=P4HB PE=1 SV=3	32	2,417	1,073	2,583	246198165,7	564714462,4	213581899,8
Q96S97	MYADM	Myeloid-associated differentiation marker OS=Homo sapiens OX=9606 GN=MYADM PE=1 SV=2	2	4,705	0,549	2,582	1525770,486	6812399,66	2714147,432
O95571	ETHE1	Persulfide dioxygenase ETHE1, mitochondrial OS=Homo sapiens OX=9606 GN=ETHE1 PE=1 SV=2	3	10,974	0,233	2,581	783925,572	7463322,825	3321196,718
P61758	VBP1	Prefoldin subunit 3 OS=Homo sapiens OX=9606 GN=VBP1 PE=1 SV=4	4	0,577	3,637	2,581	2306825,882	1050683,423	816085,5273
P35237	SERPINB6	Serpin B6 OS=Homo sapiens OX=9606 GN=SERPINB6 PE=1 SV=3	8	4,218	0,616	2,573	2043849,402	11192999,81	3489149,125
P40306	PSMB10	Proteasome subunit beta type-10 OS=Homo sapiens OX=9606 GN=PSMB10 PE=1 SV=1	2	13,509	0,159	2,565	239594,9364	4707512,208	2046249,635
Q13641	TPBG	Trophoblast glycoprotein OS=Homo sapiens OX=9606 GN=TPBG PE=1 SV=1	4	3,705	0,646	2,552	992308,8127	4738665,754	1764459,509
P20073	ANXA7	Annexin A7 OS=Homo sapiens OX=9606 GN=ANXA7 PE=1 SV=3	6	1,944	1,319	2,552	7019329,247	14171690,37	6819345,632
Q96CV9	OPTN	Optineurin OS=Homo sapiens OX=9606 GN=OPTN PE=1 SV=3	2	18,146	0,123	2,55	13141,1904	375342,1575	119770,6304
Q86Y82	STX12	Syntaxin-12 OS=Homo sapiens OX=9606 GN=STX12 PE=1 SV=1	3	1,655	1,265	2,549	614722,9493	4010815,907	1760111,116
P07942	LAMB1	Laminin subunit beta-1 OS=Homo sapiens OX=9606 GN=LAMB1 PE=1 SV=2	15	1,042	2,388	2,547	15641842,85	15922350,62	5619862,19
O00401	WASL	Actin nucleation-promoting factor WASL OS=Homo sapiens OX=9606 GN=WASL PE=1 SV=2	2	1,386	1,854	2,544	59999,77022	83152,49577	32679,38357
P07858	CTSB	Cathepsin B OS=Homo sapiens OX=9606 GN=CTSB PE=1 SV=3	5	1,355	2,046	2,539	7755469,468	5779314,211	3603143,127
Q9NW15	ANO10	Anoctamin-10 OS=Homo sapiens OX=9606 GN=ANO10 PE=1 SV=2	2	2,275	1,112	2,533	945231,2385	1616235,148	592741,5626
Q9Y6M5	SLC30A1	Proton-coupled zinc antiporter SLC30A1 OS=Homo sapiens OX=9606 GN=SLC30A1 PE=1 SV=3	8	9,33	0,39	2,53	188712,6007	9581534,175	3180842,419
Q02413	DSG1	Desmoglein-1 OS=Homo sapiens OX=9606 GN=DSG1 PE=1 SV=2	2	1000	0,001	2,52		622293,507	246990,7946
P07711	CTSL	Procathepsin L OS=Homo sapiens OX=9606 GN=CTSL PE=1 SV=2	2	9,577		2,509	28891,97221	718593,6459	156345,4986
P26006	ITGA3	Integrin alpha-3 OS=Homo sapiens OX=9606 GN=ITGA3 PE=1 SV=5	13	11,518	0,186	2,506	357766,6462	39885796,25	15118375,7
P35613	BSG	Basigin OS=Homo sapiens OX=9606 GN=BSG PE=1 SV=2	7	2,234	1,158	2,481	25715998,76	57621069,91	24057952,91
P56381	ATP5F1E	ATP synthase subunit epsilon, mitochondrial OS=Homo sapiens OX=9606 GN=ATP5F1E PE=1 SV=	2	1,464	0,667	2,481	1175703,97	1032361,638	2172064,086
O15533	TAPBP	Tapasin OS=Homo sapiens OX=9606 GN=TAPBP PE=1 SV=2	5	28,115	0,224	2,475	538141,0958	17822643,86	5790504,308
P35579	MYH9	Myosin-9 OS=Homo sapiens OX=9606 GN=MYH9 PE=1 SV=4	79	4,205	0,63	2,467	133731486,9	544512842	226769021,6
O75874	IDH1	Isocitrate dehydrogenase [NADP] cytoplasmic OS=Homo sapiens OX=9606 GN=IDH1 PE=1 SV=2	15	0,429	6,203	2,467	50470559,54	20219553,01	8404912,198
O95817	BAG3	BAG family molecular chaperone regulator 3 OS=Homo sapiens OX=9606 GN=BAG3 PE=1 SV=3	3	2,998	0,89	2,464	458823,3364	1386388,344	549754,0592
Q9UEW8	STK39	STE20/SPS1-related proline-alanine-rich protein kinase OS=Homo sapiens OX=9606 GN=STK39 PE	2	0,823	2,956	2,462	331880,1636	266942,794	108437,8608
P32455	GBP1	Guanylate-binding protein 1 OS=Homo sapiens OX=9606 GN=GBP1 PE=1 SV=2	11	19,063	0,117	2,455	4620716,328	29092822,56	13992785,48
Q15043	SLC39A14	Metal cation symporter ZIP14 OS=Homo sapiens OX=9606 GN=SLC39A14 PE=1 SV=3	2	2,404	1,098	2,454	1261228,046	2965153,452	1218847,205
P50453	SERPINB9	Serpin B9 OS=Homo sapiens OX=9606 GN=SERPINB9 PE=1 SV=1	2	1,825	1,332	2,454	964841,2458	1804654,162	1119131,164
P13645	KRT10	Keratin, type I cytoskeletal 10 OS=Homo sapiens OX=9606 GN=KRT10 PE=1 SV=6	22	2,024	1,242	2,444	190852038,6	394474538,2	178482542,1
P09914	IFIT1	Interferon-induced protein with tetratricopeptide repeats 1 OS=Homo sapiens OX=9606 GN=IFIT	12	5,499	0,501	2,44	7493577,789	33909111,81	15589522,35
P55210	CASP7	Caspase-7 OS=Homo sapiens OX=9606 GN=CASP7 PE=1 SV=1	2	2,789	0,778	2,44	105055,9597	522156,8461	215015,6359
Q13501	SQSTM1	Sequestosome-1 OS=Homo sapiens OX=9606 GN=SQSTM1 PE=1 SV=1	6	1,681	0,506	2,438	2073847,751	6106285,01	1999715,64
Q8NHP8	PLBD2	Putative phospholipase B-like 2 OS=Homo sapiens OX=9606 GN=PLBD2 PE=1 SV=2	6	1,101	2,031	2,436	4336395,248	3925615,35	2109634,854
Q9H3H5	DPAGT1	UDP-N-acetylglucosamine--dolichyl-phosphate N-acetylglucosaminephosphotransferase OS=Hom	2	1,347	1,734	2,435	611000,9979	955318,5853	369363,7969
P36639	NUDT1	Oxidized purine nucleoside triphosphate hydrolase OS=Homo sapiens OX=9606 GN=NUDT1 PE=1	3	0,411	6,023	2,434	3266437,237	1591941,996	508494,9054
Q8N726	CDKN2A	Tumor suppressor ARF OS=Homo sapiens OX=9606 GN=CDKN2A PE=1 SV=2	2	18,656	0,135	2,431	310341,1518	2682317,212	1426886,037
P53999	SUB1	Activated RNA polymerase II transcriptional coactivator p15 OS=Homo sapiens OX=9606 GN=SUB	5	0,636	3,681	2,431	26501376,4	16798401,7	7365693,337
P00387	CYB5R3	NADH-cytochrome b5 reductase 3 OS=Homo sapiens OX=9606 GN=CYB5R3 PE=1 SV=3	9	2,275	0,919	2,423	25482432	52042808,55	25401538,63
Q7Z5L9	IRF2BP2	Interferon regulatory factor 2-binding protein 2 OS=Homo sapiens OX=9606 GN=IRF2BP2 PE=1 S	5	1,05	1,498	2,423	1150972,891	537892,5148	818237,4786
P27816	MAP4	Microtubule-associated protein 4 OS=Homo sapiens OX=9606 GN=MAP4 PE=1 SV=3	27	1,829	1,22	2,421	33449797,09	63903621,67	27038323,67
O75832	PSMD10	26S proteasome non-ATPase regulatory subunit 10 OS=Homo sapiens OX=9606 GN=PSMD10 PE=	2	0,785	3,57	2,421	659585,4426	305572,2421	229232,988
P04040	CAT	Catalase OS=Homo sapiens OX=9606 GN=CAT PE=1 SV=3	23	2,631	0,863	2,419	11874234,93	34251481,89	12903667,8
O14828	SCAMP3	Secretory carrier-associated membrane protein 3 OS=Homo sapiens OX=9606 GN=SCAMP3 PE=1	5	2,453	0,945	2,419	5749854,338	14777357,38	6362436,303
Q6P1A2	LPCAT3	Lysophospholipid acyltransferase 5 OS=Homo sapiens OX=9606 GN=LPCAT3 PE=1 SV=1	8	3,237	0,716	2,417	1101370,78	4523260,186	571020,5196

O94788	ALDH1A2	Retinal dehydrogenase 2 OS=Homo sapiens OX=9606 GN=ALDH1A2 PE=1 SV=3	2	2,219	0,964	2,414	3865297,985	8192786,795	4161121,916
Q9NZI8	IGF2BP1	Insulin-like growth factor 2 mRNA-binding protein 1 OS=Homo sapiens OX=9606 GN=IGF2BP1 PE	16	0,112	22,163	2,413	88667919,71	10525286,07	5737997,111
Q53G44	IFI44L	Interferon-induced protein 44-like OS=Homo sapiens OX=9606 GN=IFI44L PE=1 SV=3	4	5,17	0,493	2,411	486015,7329	2538534,446	1081253,53
P15170	GSPT1	Eukaryotic peptide chain release factor GTP-binding subunit ERF3A OS=Homo sapiens OX=9606 (	6	0,848	2,28	2,405	4787232,031	3698755,127	2214134,115
O43432	EIF4G3	Eukaryotic translation initiation factor 4 gamma 3 OS=Homo sapiens OX=9606 GN=EIF4G3 PE=1 :	4	0,751	3,203	2,404	1113640,681	602656,7802	437109,3801
P22314	UBA1	Ubiquitin-like modifier-activating enzyme 1 OS=Homo sapiens OX=9606 GN=UBA1 PE=1 SV=3	32	0,669	3,562	2,398	136086104,3	82784903,64	38120157,32
P21964	COMT	Catechol O-methyltransferase OS=Homo sapiens OX=9606 GN=COMT PE=1 SV=2	6	6,477	0,451	2,397	994221,3592	6736109,53	2448008,572
O75340	PDCD6	Programmed cell death protein 6 OS=Homo sapiens OX=9606 GN=PDCD6 PE=1 SV=1	6	1,266	1,585	2,396	10635458,38	12006659,49	7417389,536
O60502	OGA	Protein O-GlcNAcase OS=Homo sapiens OX=9606 GN=OGA PE=1 SV=2	10	1,226	2,056	2,395	4280321,269	5390387,215	2210952,988
E7EVA0	MAP4	Microtubule-associated protein OS=Homo sapiens OX=9606 GN=MAP4 PE=1 SV=1	27	1,577	1,319	2,391	33425972,51	54857856,12	32965226,71
Q96KA5	CLPTM1L	Lipid scramblase CLPTM1L OS=Homo sapiens OX=9606 GN=CLPTM1L PE=1 SV=1	7	0,973	2,879	2,39	8214442,556	6903910,11	2882037,637
O00461	GOLIM4	Golgi integral membrane protein 4 OS=Homo sapiens OX=9606 GN=GOLIM4 PE=1 SV=1	5	1,098	2,358	2,378	3856574,811	4384127,225	1181757,531
Q07687	DLX2	Homeobox protein DLX-2 OS=Homo sapiens OX=9606 GN=DLX2 PE=1 SV=2	2	0,303	8,053	2,365	1403982,645	177083,9035	74880,43818
Q9NZM1	MYOF	Myoferlin OS=Homo sapiens OX=9606 GN=MYOF PE=1 SV=1	76	25,704	0,097	2,364	6303190,731	212791179	88110337,23
P05026	ATP1B1	Sodium/potassium-transporting ATPase subunit beta-1 OS=Homo sapiens OX=9606 GN=ATP1B1	6	3,26	0,809	2,364	4234006,913	12235152,01	5951971,607
P67936	TPM4	Tropomyosin alpha-4 chain OS=Homo sapiens OX=9606 GN=TPM4 PE=1 SV=3	8	4,216	0,583	2,363	38888967,98	172669471,1	69011490,93
Q9BYX2	TBC1D2	TBC1 domain family member 2A OS=Homo sapiens OX=9606 GN=TBC1D2 PE=1 SV=3	3	0,778	0,55	2,358	473230,9714	746786,5953	972474,0126
O94979	SEC31A	Protein transport protein Sec31A OS=Homo sapiens OX=9606 GN=SEC31A PE=1 SV=3	12	1,578	1,503	2,357	6253319,545	9986799,485	3689984,625
P10155	RO60	RNA-binding protein RO60 OS=Homo sapiens OX=9606 GN=RO60 PE=1 SV=2	9	0,518	4,314	2,357	8064282,741	4271689,299	2537632,03
Q5T9L3	WLS	Protein wntless homolog OS=Homo sapiens OX=9606 GN=WLS PE=1 SV=2	2	0,579	4,314	2,351	2705519,612	1583218,755	656455,2677
Q8WWI1	LMO7	LIM domain only protein 7 OS=Homo sapiens OX=9606 GN=LMO7 PE=1 SV=3	19	6,777	0,292	2,35	2210603,546	18033437,38	8287883,194
P05997	COL5A2	Collagen alpha-2(V) chain OS=Homo sapiens OX=9606 GN=COL5A2 PE=1 SV=3	7	3,627	0,463	2,347	562790,2693	5351295,129	402917,993
Q9BZQ8	NIBAN1	Protein Niban 1 OS=Homo sapiens OX=9606 GN=NIBAN1 PE=1 SV=1	4	7,751	0,211	2,346	456692,4309	4430411,045	1199546,62
Q9Y618	NCOR2	Nuclear receptor corepressor 2 OS=Homo sapiens OX=9606 GN=NCOR2 PE=1 SV=3	2	1,143	2,296	2,344	311547,9454	364880,784	135853,4737
P54577	YARS1	Tyrosine--tRNA ligase, cytoplasmic OS=Homo sapiens OX=9606 GN=YARS1 PE=1 SV=4	15	0,822	3,067	2,342	22543028,82	14991939,71	6613286,251
Q96IZ0	PAWR	PRKC apoptosis WT1 regulator protein OS=Homo sapiens OX=9606 GN=PAWR PE=1 SV=1	5	2,118	1,142	2,341	2612595,744	5513985,621	2562447,463
Q16740	CLPP	ATP-dependent Clp protease proteolytic subunit, mitochondrial OS=Homo sapiens OX=9606 GN=	5	1,657	1,523	2,336	2780129,657	4011742,27	2111867,93
Q9Y4L1	HYOU1	Hypoxia up-regulated protein 1 OS=Homo sapiens OX=9606 GN=HYOU1 PE=1 SV=1	37	0,801	3,135	2,331	188459422,1	124736040,8	59338192,55
P41250	GARS1	Glycine--tRNA ligase OS=Homo sapiens OX=9606 GN=GARS1 PE=1 SV=3	21	1,114	2,335	2,327	33755868,91	24460121,45	13088685,7
Q86VP6	CAND1	Cullin-associated NEDD8-dissociated protein 1 OS=Homo sapiens OX=9606 GN=CAND1 PE=1 SV=	25	0,55	3,661	2,326	44664757,1	25691737,9	10860702,33
O95834	EML2	Echinoderm microtubule-associated protein-like 2 OS=Homo sapiens OX=9606 GN=EML2 PE=1 S	4	1,816	1,423	2,325	861808,0541	1566655,707	1245977,294
Q04446	GBE1	1,4-alpha-glucan-branching enzyme OS=Homo sapiens OX=9606 GN=GBE1 PE=1 SV=3	6	2,042	1,439	2,324	739928,3456	2665259,711	921256,2337
Q8NE86	MCU	Calcium uniporter protein, mitochondrial OS=Homo sapiens OX=9606 GN=MCU PE=1 SV=1	6	4,978	0,48	2,323	1732456,769	6772938,674	3442601,248
Q3LXA3	TKFC	Triokinase/FMN cyclase OS=Homo sapiens OX=9606 GN=TKFC PE=1 SV=2	9	0,506	3,418	2,323	1324939,656	679448,7168	218878,4603
Q9P2E7	PCDH10	Protocadherin-10 OS=Homo sapiens OX=9606 GN=PCDH10 PE=1 SV=2	2	1000	0,001	2,32		1711747,135	239889,1132
P61020	RAB5B	Ras-related protein Rab-5B OS=Homo sapiens OX=9606 GN=RAB5B PE=1 SV=1	4	1,14	1,853	2,316	1976668,658	1973087,666	1286709,415
P13639	EEF2	Elongation factor 2 OS=Homo sapiens OX=9606 GN=EEF2 PE=1 SV=4	29	0,645	3,803	2,313	182006289,5	115894237,9	55722139,02
Q93050	ATP6V0A1	V-type proton ATPase 116 kDa subunit a 1 OS=Homo sapiens OX=9606 GN=ATP6V0A1 PE=1 SV=:	3	3,003	0,825	2,307	682211,0157	1921228,689	849768,6986
Q9BX59	TAPBPL	Tapasin-related protein OS=Homo sapiens OX=9606 GN=TAPBPL PE=1 SV=2	3	1000	0,001	2,306		2115749,242	1045550,583
Q15629	TRAM1	Translocating chain-associated membrane protein 1 OS=Homo sapiens OX=9606 GN=TRAM1 PE=	7	3,627	0,497	2,306	1875563,004	7550421,186	4526737,002
Q86XL3	ANKLE2	Ankyrin repeat and LEM domain-containing protein 2 OS=Homo sapiens OX=9606 GN=ANKLE2 P	4	0,853	1,554	2,305	1269627,127	871531,4142	926495,6721
Q9H5V8	CDCP1	CUB domain-containing protein 1 OS=Homo sapiens OX=9606 GN=CDCP1 PE=1 SV=3	6	1,366	1,198	2,304	475833,0674	10465027,65	3972721,586
P23381	WARS1	Tryptophan--tRNA ligase, cytoplasmic OS=Homo sapiens OX=9606 GN=WARS1 PE=1 SV=2	13	2,395	1,309	2,3	22237195,64	52308752,59	19556439,09
Q14254	FLOT2	Flotillin-2 OS=Homo sapiens OX=9606 GN=FLOT2 PE=1 SV=2	9	1,518	1,689	2,296	5858186,604	9086841,458	3845969,607
Q9UBV2	SEL1L	Protein sel-1 homolog 1 OS=Homo sapiens OX=9606 GN=SEL1L PE=1 SV=3	6	1,74	1,254	2,289	2205179,748	4042777,601	1939908,291
O00400	SLC33A1	Acetyl-coenzyme A transporter 1 OS=Homo sapiens OX=9606 GN=SLC33A1 PE=1 SV=1	2	3,194	0,605	2,288	323849,2984	1203853,374	510965,8811
Q96EL3	MRPL53	Large ribosomal subunit protein mL53 OS=Homo sapiens OX=9606 GN=MRPL53 PE=1 SV=1	2	0,58	3,945	2,288	172392,2567	99997,19672	1340289,343
P07686	HEXB	Beta-hexosaminidase subunit beta OS=Homo sapiens OX=9606 GN=HEXB PE=1 SV=4	11	3,672	0,591	2,286	12986587,34	45994654,01	22428893,13
O60784	TOM1	Target of Myb1 membrane trafficking protein OS=Homo sapiens OX=9606 GN=TOM1 PE=1 SV=2	2	1,403	1,216	2,281	424369,8563	263361,7978	319023,7339
O60701	UGDH	UDP-glucose 6-dehydrogenase OS=Homo sapiens OX=9606 GN=UGDH PE=1 SV=1	15	0,699	3,129	2,275	21558724,07	22752187,09	11014734,02
Q9BV20	MRI1	Methylthioribose-1-phosphate isomerase OS=Homo sapiens OX=9606 GN=MRI1 PE=1 SV=1	2	0,782	2,892	2,263	624326,7443	479601,4509	199690,3107
A0A1B0GVI3	KRT10	Keratin, type I cytoskeletal 10 OS=Homo sapiens OX=9606 GN=KRT10 PE=1 SV=2	23	1,733	1,361	2,26	225678706,1	403118989	185359586,3
Q13561	DCTN2	Dynactin subunit 2 OS=Homo sapiens OX=9606 GN=DCTN2 PE=1 SV=4	9	0,919	2,329	2,26	13321234,25	11237456,73	4995716,162
Q9UMY4	SNX12	Sorting nexin-12 OS=Homo sapiens OX=9606 GN=SNX12 PE=1 SV=4	3	0,793	2,829	2,245	3275178,664	1159637,599	1065458,426
P33527	ABCC1	Multidrug resistance-associated protein 1 OS=Homo sapiens OX=9606 GN=ABCC1 PE=1 SV=3	15	3,653	0,559	2,243	1886531,204	8441374,61	4257393,447
P00492	HPRT1	Hypoxanthine-guanine phosphoribosyltransferase OS=Homo sapiens OX=9606 GN=HPRT1 PE=1 !	7	1,496	1,783	2,242	7439841,694	9067355,268	4272443,941
Q14956	GPNMB	Transmembrane glycoprotein NMB OS=Homo sapiens OX=9606 GN=GPNMB PE=1 SV=2	3	2,164	0,295	2,238	23771,26651	1546408,72	102807,372
Q96TC7	RMDN3	Regulator of microtubule dynamics protein 3 OS=Homo sapiens OX=9606 GN=RMDN3 PE=1 SV=2	9	1,432	1,342	2,238	1891462,297	3094021,91	1390675,841
Q9BW19	KIFC1	Kinesin-like protein KIFC1 OS=Homo sapiens OX=9606 GN=KIFC1 PE=1 SV=2	8	0,797	2,48	2,23	3114628,783	1668654,329	844930,0208
P52272	HNRNPM	Heterogeneous nuclear ribonucleoprotein M OS=Homo sapiens OX=9606 GN=HNRNPM PE=1 SV-	30	0,856	2,875	2,229	148536900,4	125593529,8	66144071,89
Q5JRA6	MIA3	Transport and Golgi organization protein 1 homolog OS=Homo sapiens OX=9606 GN=MIA3 PE=1	10	1,84	1,313	2,226	2890446,411	4615160,352	2224770,586
Q9H0U4	RAB1B	Ras-related protein Rab-1B OS=Homo sapiens OX=9606 GN=RAB1B PE=1 SV=1	3	1,627	1,525	2,214	12494215,76	19573277,02	9866444,64
P29373	CRABP2	Cellular retinoic acid-binding protein 2 OS=Homo sapiens OX=9606 GN=CRABP2 PE=1 SV=2	4	0,021	7,869	2,214	27429064,67	680548,474	896462,116
O14880	MGST3	Glutathione S-transferase 3, mitochondrial OS=Homo sapiens OX=9606 GN=MGST3 PE=1 SV=1	2	1,626	1,361	2,213	2146464,098	3335093,522	1507341,285
O00483	NDUFA4	Cytochrome c oxidase subunit NDUFA4 OS=Homo sapiens OX=9606 GN=NDUFA4 PE=1 SV=1	3	1,227	1,724	2,212	6742434,694	9147861,898	4185931,59
Q13162	PRDX4	Peroxiredoxin-4 OS=Homo sapiens OX=9606 GN=PRDX4 PE=1 SV=1	7	1,161	2,436	2,212	49400094,29	48624057,83	22739500,94
Q9H1B7	IRF2BPL	Probable E3 ubiquitin-protein ligase IRF2BPL OS=Homo sapiens OX=9606 GN=IRF2BPL PE=1 SV=:	6	0,548	3,995	2,212	5903982,37	2949833,501	1365480,273
Q9Y371	SH3GLB1	Endophilin-B1 OS=Homo sapiens OX=9606 GN=SH3GLB1 PE=1 SV=1	6	1,584	1,033	2,209	2812572,446	4118480,316	2422736,664
Q6FI13	H2AC18	Histone H2A type 2-A OS=Homo sapiens OX=9606 GN=H2AC18 PE=1 SV=3	4	0,763	1,694	2,209	1686281137	1252668505	1629207543
Q9ULC4	MCTS1	Malignant T-cell-amplified sequence 1 OS=Homo sapiens OX=9606 GN=MCTS1 PE=1 SV=1	3	0,667	3,059	2,209	2159251,712	920185,2264	638688,0131
Q96GD0	PDXP	Chronophin OS=Homo sapiens OX=9606 GN=PDXP PE=1 SV=2	4	0,256	4,991	2,203	1572249,81	8185875,806	490302,0925
P27797	CALR	Calreticulin OS=Homo sapiens OX=9606 GN=CALR PE=1 SV=1	19	2,258	0,956	2,201	189100692,2	403597277,4	227533989,9
P53680	AP2S1	AP-2 complex subunit sigma OS=Homo sapiens OX=9606 GN=AP2S1 PE=1 SV=2	3	2,524	0,672	2,199	1506655,972	2997019,715	1211781,09
P18827	SDC1	Syndecan-1 OS=Homo sapiens OX=9606 GN=SDC1 PE=1 SV=3	4	1,176	2,187	2,199	5702639,334	6292659,876	2787170,532
Q99536	VAT1	Synaptic vesicle membrane protein VAT-1 homolog OS=Homo sapiens OX=9606 GN=VAT1 PE=1 !	11	1,537	1,34	2,196	25181087,33	38029177,88	17093127,84
P02768	ALB	Albumin OS=Homo sapiens OX=9606 GN=ALB PE=1 SV=2	7	0,913	0,709	2,19	23257838,75	21047699,17	30849898,2
Q9NUQ9	CYRIB	CYFIP-related Rac1 interactor B OS=Homo sapiens OX=9606 GN=CYRIB PE=1 SV=1	4	1,15	1,973	2,189	5867865,91	6499289,667	2564003,524
Q99808	SLC29A1	Equilibrative nucleoside transporter 1 OS=Homo sapiens OX=9606 GN=SLC29A1 PE=1 SV=3	2	1000	0,001	2,188		1585639,766	724545,9545
Q9NZJ7	MTCH1	Mitochondrial carrier homolog 1 OS=Homo sapiens OX=9606 GN=MTCH1 PE=1 SV=1	4	1,065	2,078	2,187	2722468,319	3299687,426	1074682,464
Q14203	DCTN1	Dynactin subunit 1 OS=Homo sapiens OX=9606 GN=DCTN1 PE=1 SV=3	10	0,856	2,79	2,187	7628511,365	5856516,815	2250387,103
P11766	ADH5	Alcohol dehydrogenase class-3 OS=Homo sapiens OX=9606 GN=ADH5 PE=1 SV=4	8	0,425	5,136	2,187	19736701,55	7562204,638	6488320,598
P21397	MAOA	Amine oxidase [flavin-containing] A OS=Homo sapiens OX=9606 GN=MAOA PE=1 SV=1	19	1,476	1,256	2,184	4467740,083	5056629,065	2636183,393
Q99816	TSG101	Tumor susceptibility gene 101 protein OS=Homo sapiens OX=9606 GN=TSG101 PE=1 SV=2	5	0,823	2,23	2,182	1697168,49	1388484,841	627751,5138
P04264	KRT1	Keratin, type II cytoskeletal 1 OS=Homo sapiens OX=9606 GN=KRT1 PE=1 SV=6	34	1,361	1,559	2,18	613171087,8	890289781	358962770,6
Q96BJ3	AIDA	Axin interactor, dorsalization-associated protein OS=Homo sapiens OX=9606 GN=AIDA PE=1 SV=	2	1,733	2,035	2,179	608530,7649	301320,1649	272451,3841
O14910	LIN7A	Protein lin-7 homolog A OS=Homo sapiens OX=9606 GN=LIN7A PE=1 SV=2	2	3,388	0,875	2,178	1027463,181	2379869,579	1019002,455
H0YD14	MYOF	Myoferlin (Fragment) OS=Homo sapiens OX=9606 GN=MYOF PE=1 SV=1	37	14,692	0,141	2,177	15890010,84	114393079,4	58693594,53
P07355	ANXA2	Annexin A2 OS=Homo sapiens OX=9606 GN=ANXA2 PE=1 SV=2	23	6,359	0,345	2,175	133073302,4	782607685,8	407645832,8
Q9H3K6	BOLA2	BolA-like protein 2 OS=Homo sapiens OX=9606 GN=BOLA2 PE=1 SV=1	2	0,746	2,797	2,173	2408137,386	1803708,52	766378,94
Q12857	NFIA	Nuclear factor 1 A-type OS=Homo sapiens OX=9606 GN=NFIA PE=1 SV=2	2	4,038	0,349	2,172	251570,8745	1676840,933	878069,0615
Q9UGI8	TES	Testin OS=Homo sapiens OX=9606 GN=TES PE=1 SV=1	4	1,546	1,4	2,167	2759301,016	4124500,837	1979773,271
P51659	HSD17B4	Peroxisomal multifunctional enzyme type 2 OS=Homo sapiens OX=9606 GN=HSD17B4 PE=1 SV=:	28	1,493	1,627	2,167	49182614,05	70598227,47	34529191,46
Q14165	MLEC	Malectin OS=Homo sapiens OX=9606 GN=MLEC PE=1 SV=1	13	0,809	2,631	2,167	32401013,32	24362794,72	10667790,72
P49589	CARS1	Cysteine--tRNA ligase, cytoplasmic OS=Homo sapiens OX=9606 GN=CARS1 PE=1 SV=3	10	1,062	1,974	2,162	7766331,516	5732915,44	3858076,191
Q13492	PICALM	Phosphatidylinositol-binding clathrin assembly protein OS=Homo sapiens OX=9606 GN=PICALM	11	2,671	0,752	2,161	4679387,916	13394412,46	6324508,838
P55145	MANF	Mesencephalic astrocyte-derived neurotrophic factor OS=Homo sapiens OX=9606 GN=MANF PE=	6	1,177	1,754	2,16	14121788,47	15675481,11	9121332,527
P52788	SMS	Spermine synthase OS=Homo sapiens OX=9606 GN=SMS PE=1 SV=2	5	0,848	2,386	2,153	2919348,422	2084929,374	1079406,306
P49720	PSMB3	Proteasome subunit beta type-3 OS=Homo sapiens OX=9606 GN=PSMB3 PE=1 SV=2	6	1,342	1,829	2,149	11521144,04	13514108,86	8552439,478
Q9NP97	DYNLRB1	Dynein light chain roadblock-type 1 OS=Homo sapiens OX=9606 GN=DYNLRB1 PE=1 SV=3	4	0,8	2,77	2,149	5584472,197	4342914,431	1897357,414
A0A0A0MQV6	FGF2	Fibroblast growth factor OS=Homo sapiens OX=9606 GN=FGF2 PE=1 SV=1	2	1,52	1,393	2,145	955343,1902	1366986,636	922786,0296
O95816	BAG2	BAG family molecular chaperone regulator 2 OS=Homo sapiens OX=9606 GN=BAG2 PE=1 SV=1	2	1,335	1,745	2,142	1850512,031	2604196,276	539173,6187
Q8TBC4	UBA3	NEDD8-activating enzyme E1 catalytic subunit OS=Homo sapiens OX=9606 GN=UBA3 PE=1 SV=2	3	0,862	3,125	2,142	922157,1771	1105606,807	119815,1173
Q96P70	IPO9	Importin-9 OS=Homo sapiens OX=9606 GN=IPO9 PE=1 SV=3	11	0,681	3,326	2,14	8402607,915	5257927,193	2703168,292
Q9GZZ9	UBA5	Ubiquitin-like modifier-activating enzyme 5 OS=Homo sapiens OX=9606 GN=UBA5 PE=1 SV=1	3	0,946	1,997	2,138	733628,5451	239507,9639	136032,8715
Q99757	TXN2	Thioredoxin, mitochondrial OS=Homo sapiens OX=9606 GN=TXN2 PE=1 SV=2	3	2,166	1,156	2,137	700289,6311	1216296,921	1047822,574
P37837	TALDO1	Transaldolase OS=Homo sapiens OX=9606 GN=TALDO1 PE=1 SV=2	10	1,464	1,217	2,132	20377248,21	32250474,46	14994424,99
P10589	NR2F1	COUP transcription factor 1 OS=Homo sapiens OX=9606 GN=NR2F1 PE=1 SV=1	2	2,221	1,008	2,13	553468,7871	1205410,979	553601,6544
Q71U36	TUBA1A	Tubulin alpha-1A chain OS=Homo sapiens OX=9606 GN=TUBA1A PE=1 SV=1	12	0,335	6,994	2,127	1432941966	470275138,7	216182703,1
P51809	VAMP7	Vesicle-associated membrane protein 7 OS=Homo sapiens OX=9606 GN=VAMP7 PE=1 SV=3	3	2,014	1,049	2,12	568336,4932	1110115,274	463963,0829
Q9UI12	ATP6V1H	V-type proton ATPase subunit H OS=Homo sapiens OX=9606 GN=ATP6V1H PE=1 SV=1	5	0,882	2,648	2,12	1845311,577	2160280,374	1187948,336
P30046	DDT	D-dopachrome decarboxylase OS=Homo sapiens OX=9606 GN=DDT PE=1 SV=3	3	0,946	2,343	2,119	5525771,702	4619236,743	2411813,94

P00390	GSR	Glutathione reductase, mitochondrial OS=Homo sapiens OX=9606 GN=GSR PE=1 SV=2	11	0,446	4,372	2,119	15048690,5	6012672,476	3683408,196
P11498	PC	Pyruvate carboxylase, mitochondrial OS=Homo sapiens OX=9606 GN=PC PE=1 SV=2	6	2,082	1,07	2,117	1328690,76	3511013,734	1426227,975
P63096	GNAI1	Guanine nucleotide-binding protein G(i) subunit alpha-1 OS=Homo sapiens OX=9606 GN=GNAI1	2	0,762	3,182	2,114	2303618,64	1919404,836	755553,668
Q6P996	PDXDC1	Pyridoxal-dependent decarboxylase domain-containing protein 1 OS=Homo sapiens OX=9606 GN	4	6,207	0,793	2,111	199686,3603	2346282,276	592382,7073
P21926	CD9	CD9 antigen OS=Homo sapiens OX=9606 GN=CD9 PE=1 SV=4	2	26,706	0,076	2,109	33601,02644	1202337,911	544419,2193
P52565	ARHGDIA	Rho GDP-dissociation inhibitor 1 OS=Homo sapiens OX=9606 GN=ARHGDIA PE=1 SV=3	6	0,912	2,37	2,108	32316892,51	27419708,51	12856766,04
P08238	HSP90AB1	Heat shock protein HSP 90-beta OS=Homo sapiens OX=9606 GN=HSP90AB1 PE=1 SV=4	23	0,403	5,484	2,104	1316990502	540605526	318482852,6
Q9H488	POFUT1	GDP-fucose protein O-fucosyltransferase 1 OS=Homo sapiens OX=9606 GN=POFUT1 PE=1 SV=1	6	1,675	1,321	2,103	6952302,954	10767281,77	5770051,384
Q92820	GGH	Gamma-glutamyl hydrolase OS=Homo sapiens OX=9606 GN=GGH PE=1 SV=2	8	0,986	1,797	2,098	9383498,257	6945447,102	4712368,689
O00764	PDXK	Pyridoxal kinase OS=Homo sapiens OX=9606 GN=PDXK PE=1 SV=1	6	2,055	0,886	2,097	2166405,336	4897248,031	2544612,879
O94973	AP2A2	AP-2 complex subunit alpha-2 OS=Homo sapiens OX=9606 GN=AP2A2 PE=1 SV=2	5	1,603	1,349	2,095	1173552,275	2820389,818	784826,9271
P05166	PCCB	Propionyl-CoA carboxylase beta chain, mitochondrial OS=Homo sapiens OX=9606 GN=PCCB PE=1	8	0,757	1,514	2,095	2013042,233	2156593,883	849827,5997
P81605	DCD	Dermcidin OS=Homo sapiens OX=9606 GN=DCD PE=1 SV=2	3	2,468	0,863	2,094	4820638,788	12289879,87	6820681,181
Q9Y2L1	DIS3	Exosome complex exonuclease RRP44 OS=Homo sapiens OX=9606 GN=DIS3 PE=1 SV=2	4	0,804	2,041	2,094	1241342,93	1423342,792	539904,9631
P11279	LAMP1	Lysosome-associated membrane glycoprotein 1 OS=Homo sapiens OX=9606 GN=LAMP1 PE=1 SV	4	1,256	1,588	2,085	17719423,99	22152025,91	10001787,45
O75663	TIPRL	TIP41-like protein OS=Homo sapiens OX=9606 GN=TIPRL PE=1 SV=2	4	0,957	1,8	2,084	1505299,429	1376899,297	485988,23
P02545	LMNA	Prelamin-A/C OS=Homo sapiens OX=9606 GN=LMNA PE=1 SV=1	40	17,715	0,133	2,081	76741724,44	1203766543	525079651,1
P19367	HK1	Hexokinase-1 OS=Homo sapiens OX=9606 GN=HK1 PE=1 SV=3	21	2,64	0,743	2,08	19397316,83	45170451,21	28085216,29
Q9H2G2	SLK	STE20-like serine/threonine-protein kinase OS=Homo sapiens OX=9606 GN=SLK PE=1 SV=1	2	1,048	1,957	2,076	679477,1372	723111,466	94457,38139
P49354	FNTA	Protein farnesyltransferase/geranylgeranyltransferase type-1 subunit alpha OS=Homo sapiens O	4	1,007	1,485	2,072	315782,7054	1691135,982	148689,3067
Q99541	PLIN2	Perilipin-2 OS=Homo sapiens OX=9606 GN=PLIN2 PE=1 SV=2	7	1,215	2,008	2,07	1055022,404	939932,8131	636812,4345
Q9Y263	PLAA	Phospholipase A-2-activating protein OS=Homo sapiens OX=9606 GN=PLAA PE=1 SV=2	6	1,544	1,314	2,067	3391383,883	4840754,418	1971061,596
O14950	MYL12B	Myosin regulatory light chain 12B OS=Homo sapiens OX=9606 GN=MYL12B PE=1 SV=2	7	2,965	0,657	2,062	31146720,12	95818602,07	47581947,13
Q9HCN8	SDF2L1	Stromal cell-derived factor 2-like protein 1 OS=Homo sapiens OX=9606 GN=SDF2L1 PE=1 SV=2	4	1,234	2,245	2,057	3151124,491	3116874,033	929150,8718
Q14344	GNA13	Guanine nucleotide-binding protein subunit alpha-13 OS=Homo sapiens OX=9606 GN=GNA13 PE	3	3,76	0,826	2,054	863116,1671	3979866,835	1784326,325
Q15526	SURF1	Surfeit locus protein 1 OS=Homo sapiens OX=9606 GN=SURF1 PE=1 SV=1	3	3,676	0,636	2,046	216701,5548	1903859,988	1959445,924
Q15075	EEA1	Early endosome antigen 1 OS=Homo sapiens OX=9606 GN=EEA1 PE=1 SV=2	28	4,426	0,555	2,04	3416392,996	21047179,52	9041838,585
P40763	STAT3	Signal transducer and activator of transcription 3 OS=Homo sapiens OX=9606 GN=STAT3 PE=1 SV	9	1,796	1,128	2,04	3059619,249	5504608,978	4238649,282
Q9BRP8	PYM1	Partner of Y14 and mago OS=Homo sapiens OX=9606 GN=PYM1 PE=1 SV=1	6	0,593	3,593	2,04	3950265,056	2613256,611	700334,5877
Q96TA1	NIBAN2	Protein Niban 2 OS=Homo sapiens OX=9606 GN=NIBAN2 PE=1 SV=3	16	5,315	0,353	2,038	2949486,372	16972381,35	8849681,912
P06733	ENO1	Alpha-enolase OS=Homo sapiens OX=9606 GN=ENO1 PE=1 SV=2	18	0,724	3,015	2,036	574162400,8	369549332,7	198805389,9
O15427	SLC16A3	Monocarboxylate transporter 4 OS=Homo sapiens OX=9606 GN=SLC16A3 PE=1 SV=1	5	60,36	0,036	2,028	365355,4157	22342477	9053754,327
P24844	MYL9	Myosin regulatory light polypeptide 9 OS=Homo sapiens OX=9606 GN=MYL9 PE=1 SV=4	5	2,385	0,759	2,025	17354052,26	46801372,95	22934800,81
Q15208	STK38	Serine/threonine-protein kinase 38 OS=Homo sapiens OX=9606 GN=STK38 PE=1 SV=1	2	1,86	1,062	2,022	615143,2607	1189263,534	572945,952
Q9NQX3	GPHN	Gephyrin OS=Homo sapiens OX=9606 GN=GPHN PE=1 SV=1	2	0,575	3,378	2,021	910992,4669	536017,2239	281434,0623
P13473	LAMP2	Lysosome-associated membrane glycoprotein 2 OS=Homo sapiens OX=9606 GN=LAMP2 PE=1 SV	3	2,564	1,261	2,018	1187466,11	1788318,697	780320,8642
Q92783	STAM	Signal transducing adapter molecule 1 OS=Homo sapiens OX=9606 GN=STAM PE=1 SV=3	4	1,072	1,701	2,018	3203746,665	2221418,483	1132549,76
Q9P000	COMMD9	COMM domain-containing protein 9 OS=Homo sapiens OX=9606 GN=COMMD9 PE=1 SV=2	2	0,875	2,647	2,016	160193,0866	109748,8888	60519,90239
O75935	DCTN3	Dynactin subunit 3 OS=Homo sapiens OX=9606 GN=DCTN3 PE=1 SV=1	2	1,34	1,528	2,013	657658,6822	854809,4771	424580,9437
P60903	S100A10	Protein S100-A10 OS=Homo sapiens OX=9606 GN=S100A10 PE=1 SV=2	2	11,507	0,172	2,012	3626464,124	43490973,79	18593707,26
P04179	SOD2	Superoxide dismutase [Mn], mitochondrial OS=Homo sapiens OX=9606 GN=SOD2 PE=1 SV=3	11	5,853	0,383	2,012	12799446,86	76497763,59	29459137,94
H0YMW4	ANXA2	Annexin OS=Homo sapiens OX=9606 GN=ANXA2 PE=1 SV=2	23	6,036	0,327	2,009	130570335,9	713738321,9	394470179,5
Q9HDC9	APMAP	Adipocyte plasma membrane-associated protein OS=Homo sapiens OX=9606 GN=APMAP PE=1 S	17	1,731	1,196	1,996	39829658,34	66433925,31	35007741,89
Q16666	IFI16	Gamma-interferon-inducible protein 16 OS=Homo sapiens OX=9606 GN=IFI16 PE=1 SV=3	12	6,619	0,296	1,994	1993122,769	16677431,6	7557418,426
P06756	ITGAV	Integrin alpha-V OS=Homo sapiens OX=9606 GN=ITGAV PE=1 SV=2	19	2,738	0,645	1,992	7674977,465	21786288,59	11696048,68
P63104	YWHAZ	14-3-3 protein zeta/delta OS=Homo sapiens OX=9606 GN=YWHAZ PE=1 SV=1	10	0,848	2,402	1,988	249263074,8	214651200,1	103594596,9
O75390	CS	Citrate synthase, mitochondrial OS=Homo sapiens OX=9606 GN=CS PE=1 SV=2	15	1,288	1,665	1,987	46966086,93	55333725,11	29842843,86
P78357	CNTNAP1	Contactin-associated protein 1 OS=Homo sapiens OX=9606 GN=CNTNAP1 PE=1 SV=1	2	5,131	0,452	1,986	104838,9973	983796,233	225049,9113
Q4ZHG4	FNDC1	Fibronectin type III domain-containing protein 1 OS=Homo sapiens OX=9606 GN=FNDC1 PE=1 SV	2	2,213	0,839	1,984	3313644,672	3366083,68	4478967,588
P19013	KRT4	Keratin, type II cytoskeletal 4 OS=Homo sapiens OX=9606 GN=KRT4 PE=1 SV=5	2	1000	0,001	1,983		1457477,127	41864,5112
P12532	CKMT1A	Creatine kinase U-type, mitochondrial OS=Homo sapiens OX=9606 GN=CKMT1A PE=1 SV=1	7	1,567	1,425	1,983	3800214,902	4524428,705	1713366,896
P07737	PFN1	Profilin-1 OS=Homo sapiens OX=9606 GN=PFN1 PE=1 SV=2	8	0,769	2,607	1,979	218755052,1	167130708,5	100326067,1
P10768	ESD	S-formylglutathione hydrolase OS=Homo sapiens OX=9606 GN=ESD PE=1 SV=2	5	0,785	3,385	1,978	4466108,916	4123486,069	1921495,564
O43598	DNPH1	5-hydroxymethyl-dUMP N-hydrolase OS=Homo sapiens OX=9606 GN=DNPH1 PE=1 SV=1	3	0,591	2,721	1,975	1141432,508	1027171,459	610089,9611
Q9UHV9	PFDN2	Prefoldin subunit 2 OS=Homo sapiens OX=9606 GN=PFDN2 PE=1 SV=1	2	0,596	3,344	1,973	8011937,799	4385891,406	2537554,177
P54619	PRKAG1	5'-AMP-activated protein kinase subunit gamma-1 OS=Homo sapiens OX=9606 GN=PRKAG1 PE=1	2	0,958	1,625	1,972	838980,0846	855538,7569	255172,5725
P53634	CTSC	Dipeptidyl peptidase 1 OS=Homo sapiens OX=9606 GN=CTSC PE=1 SV=2	5	0,274	4,167	1,971	10818050,07	2479466,204	1689115,303
P16615	ATP2A2	Sarcoplasmic/endoplasmic reticulum calcium ATPase 2 OS=Homo sapiens OX=9606 GN=ATP2A2	31	2,066	0,936	1,967	53662561,98	110420884,5	59501147,06
Q8WZA9	IRGQ	Immunity-related GTPase family Q protein OS=Homo sapiens OX=9606 GN=IRGQ PE=1 SV=1	5	0,571	3,695	1,966	1511228,116	883621,0059	448078,2535
Q7Z3E5	ARMC9	LisH domain-containing protein ARMC9 OS=Homo sapiens OX=9606 GN=ARMC9 PE=1 SV=3	3	0,379	5,718	1,963	553273,5473	266031,8893	107678,9726
Q9Y3Z3	SAMHD1	Deoxynucleoside triphosphate triphosphohydrolase SAMHD1 OS=Homo sapiens OX=9606 GN=SA	19	5,894	0,335	1,961	6648306,944	43153266,39	22729923,86
Q12792	TWF1	Twinfilin-1 OS=Homo sapiens OX=9606 GN=TWF1 PE=1 SV=3	7	1,393	1,513	1,961	3805013,369	4496590,874	1954508,677
Q8IXM6	NRM	Nurim OS=Homo sapiens OX=9606 GN=NRM PE=1 SV=1	5	0,692	0,562	1,96	2385993,39	1237165,562	1471468,862
Q5JPE7	NOMO2	BOS complex subunit NOMO2 OS=Homo sapiens OX=9606 GN=NOMO2 PE=1 SV=1	2	2,447	0,794	1,959	18330082,73	45902575,83	22479132,45
H0Y987	PGM3	Phosphoacetylglucosamine mutase OS=Homo sapiens OX=9606 GN=PGM3 PE=1 SV=2	2	1,134	1,626	1,959	1230164,586	1299498,644	611148,2402
O00625	PIR	Pirin OS=Homo sapiens OX=9606 GN=PIR PE=1 SV=1	5	0,416	5,021	1,958	3363303,626	1394603,225	305904,728
Q9NXG2	THUMPD1	THUMP domain-containing protein 1 OS=Homo sapiens OX=9606 GN=THUMPD1 PE=1 SV=2	2	1,953	0,977	1,957	495365,3233	955080,5352	506799,2542
P29401	TKT	Transketolase OS=Homo sapiens OX=9606 GN=TKT PE=1 SV=3	24	0,968	1,935	1,957	199921525,5	189706138	98898660,94
O95394	PGM3	Phosphoacetylglucosamine mutase OS=Homo sapiens OX=9606 GN=PGM3 PE=1 SV=1	2	1,33	1,397	1,949	959176,1355	1279358,027	697747,1869
O43570	CA12	Carbonic anhydrase 12 OS=Homo sapiens OX=9606 GN=CA12 PE=1 SV=1	7	0,938	1,714	1,948	1043306,407	1303233,789	99856,18392
P43490	NAMPT	Nicotinamide phosphoribosyltransferase OS=Homo sapiens OX=9606 GN=NAMPT PE=1 SV=1	17	1,684	1,234	1,947	15773443,43	27116270,53	12500791,36
Q9Y6G9	DYNC1LI1	Cytoplasmic dynein 1 light intermediate chain 1 OS=Homo sapiens OX=9606 GN=DYNC1LI1 PE=1	10	0,449	3,753	1,945	9244001,053	4643647,943	2314844,792
Q9Y6I3	EPN1	Epsin-1 OS=Homo sapiens OX=9606 GN=EPN1 PE=1 SV=2	4	1,773	1,09	1,944	1067371,271	2768923,057	1362731,669
P50552	VASP	Vasodilator-stimulated phosphoprotein OS=Homo sapiens OX=9606 GN=VASP PE=1 SV=3	9	3,335	0,57	1,943	2260114,625	9754461,614	4702439,351
P27708	CAD	Multifunctional protein CAD OS=Homo sapiens OX=9606 GN=CAD PE=1 SV=3	23	0,391	5,145	1,942	22693184,98	7017529,733	3568815,166
Q9Y6B6	SAR1B	GTP-binding protein SAR1b OS=Homo sapiens OX=9606 GN=SAR1B PE=1 SV=1	2	1,844	1,037	1,941	1829631,522	3283166,754	1805998,429
P49902	NT5C2	Cytosolic purine 5'-nucleotidase OS=Homo sapiens OX=9606 GN=NT5C2 PE=1 SV=1	7	0,991	2,372	1,941	1729178,329	1170219,164	745721,1798
Q96C90	PPP1R14B	Protein phosphatase 1 regulatory subunit 14B OS=Homo sapiens OX=9606 GN=PPP1R14B PE=1 S	2	0,496	3,773	1,936	1610366,855	876895,392	370335,2092
Q9UNN5	FAF1	FAS-associated factor 1 OS=Homo sapiens OX=9606 GN=FAF1 PE=1 SV=2	3	0,498	3,054	1,933	2110985,064	236038,2073	796325,8351
P50336	PPOX	Protoporphyrinogen oxidase OS=Homo sapiens OX=9606 GN=PPOX PE=1 SV=1	2	1,628	1,193	1,926	972714,7352	1761620,228	272033,4094
Q96B97	SH3KBP1	SH3 domain-containing kinase-binding protein 1 OS=Homo sapiens OX=9606 GN=SH3KBP1 PE=1	3	4,907	0,359	1,923	307395,9938	1771774,087	600927,1206
Q03013	GSTM4	Glutathione S-transferase Mu 4 OS=Homo sapiens OX=9606 GN=GSTM4 PE=1 SV=3	2	0,762	2,52	1,92	1787683,079	1179058,9	692713,2662
Q9UBN7	HDAC6	Histone deacetylase 6 OS=Homo sapiens OX=9606 GN=HDAC6 PE=1 SV=2	2	0,548	3,637	1,92	1313177,281	863488,195	387799,1341
Q9Y2U8	LEMD3	Inner nuclear membrane protein Man1 OS=Homo sapiens OX=9606 GN=LEMD3 PE=1 SV=2	6	1,12	1,919	1,913	1537823,17	1101757,509	839323,7345
P31431	SDC4	Syndecan-4 OS=Homo sapiens OX=9606 GN=SDC4 PE=1 SV=2	3	5,837	0,326	1,912	579397,7931	3831094,812	1766959,105
P37108	SRP14	Signal recognition particle 14 kDa protein OS=Homo sapiens OX=9606 GN=SRP14 PE=1 SV=2	3	0,437	4,265	1,912	12780649,54	4639051,157	2779603,581
P29992	GNA11	Guanine nucleotide-binding protein subunit alpha-11 OS=Homo sapiens OX=9606 GN=GNA11 PE	5	3,154	0,628	1,911	2858061,038	9453392,221	4472288,323
P17301	ITGA2	Integrin alpha-2 OS=Homo sapiens OX=9606 GN=ITGA2 PE=1 SV=1	22	27,598	0,041	1,909	987991,3987	64536012,64	34095142,15
P21281	ATP6V1B2	V-type proton ATPase subunit B, brain isoform OS=Homo sapiens OX=9606 GN=ATP6V1B2 PE=1	8	0,919	2,017	1,909	5301755,79	4040805,352	3231191,438
Q4U2R6	MRPL51	Large ribosomal subunit protein mL51 OS=Homo sapiens OX=9606 GN=MRPL51 PE=1 SV=1	2	2,117	1,525	1,908	156003,2683	437659,8923	209514,903
Q9H6V9	LDAH	Lipid droplet-associated hydrolase OS=Homo sapiens OX=9606 GN=LDAH PE=1 SV=1	2	0,375	4,569	1,907	946184,8609	503025,5355	217002,1
O14967	CLGN	Calmegin OS=Homo sapiens OX=9606 GN=CLGN PE=1 SV=1	3	0,838	3,685	1,9	1640032,668	1182117,738	179890,9208
Q15654	TRIP6	Thyroid receptor-interacting protein 6 OS=Homo sapiens OX=9606 GN=TRIP6 PE=1 SV=3	3	2,875	0,877	1,899	818888,858	2254924,433	1133962,271
P04792	HSPB1	Heat shock protein beta-1 OS=Homo sapiens OX=9606 GN=HSPB1 PE=1 SV=2	10	3,737	0,682	1,898	49162198,26	144444262,1	82844944,11
Q9UBW8	COPS7A	COP9 signalosome complex subunit 7a OS=Homo sapiens OX=9606 GN=COPS7A PE=1 SV=1	2	1,666	1,123	1,898	565260,7599	961114,1476	244312,8545
P26885	FKBP2	Peptidyl-prolyl cis-trans isomerase FKBP2 OS=Homo sapiens OX=9606 GN=FKBP2 PE=1 SV=2	6	1,465	1,195	1,896	8361197,142	10755203,41	6941656,928
O95861	BPNT1	3'(2'),5'-bisphosphate nucleotidase 1 OS=Homo sapiens OX=9606 GN=BPNT1 PE=1 SV=1	3	1,442	1,309	1,895	502171,7736	723972,768	395000,9657
P61970	NUTF2	Nuclear transport factor 2 OS=Homo sapiens OX=9606 GN=NUTF2 PE=1 SV=1	2	0,606	3,03	1,894	4385303,242	2609342,597	1572606,668
Q07866	KLC1	Kinesin light chain 1 OS=Homo sapiens OX=9606 GN=KLC1 PE=1 SV=2	10	0,932	1,884	1,893	10331907,73	7257722,376	3773325,402
Q14204	DYNC1H1	Cytoplasmic dynein 1 heavy chain 1 OS=Homo sapiens OX=9606 GN=DYNC1H1 PE=1 SV=5	109	0,576	3,143	1,892	141099097,5	72388089,05	42208698,98
P24593	IGFBP5	Insulin-like growth factor-binding protein 5 OS=Homo sapiens OX=9606 GN=IGFBP5 PE=1 SV=1	2	5,6	0,334	1,884	239372,9384	1302624,449	151322,3231
Q9P0V9	SEPTIN10	Septin-10 OS=Homo sapiens OX=9606 GN=SEPTIN10 PE=1 SV=2	4	0,866	2,103	1,884	1679328,591	1420612,36	665961,0242
Q9UPN3	MACF1	Microtubule-actin cross-linking factor 1, isoforms 1/2/3/4/5 OS=Homo sapiens OX=9606 GN=MA	32	0,634	3,055	1,882	14002956,84	8027948,003	3414746,778
O95297	MPZL1	Myelin protein zero-like protein 1 OS=Homo sapiens OX=9606 GN=MPZL1 PE=1 SV=1	3	2,827	2,127	1,881	200439,8025	2551443,439	1868544,165
Q8NBX0	SCCPDH	Saccharopine dehydrogenase-like oxidoreductase OS=Homo sapiens OX=9606 GN=SCCPDH PE=1	7	2,143	0,788	1,881	5255887,361	10789150,25	6648410,793
P25445	FAS	Tumor necrosis factor receptor superfamily member 6 OS=Homo sapiens OX=9606 GN=FAS PE=1	2	1000	0,001	1,88		977271,193	495093,6909
O95155	UBE4B	Ubiquitin conjugation factor E4 B OS=Homo sapiens OX=9606 GN=UBE4B PE=1 SV=1	3	0,471	4,12	1,88	953877,0112	238788,2761	120117,6446
Q96M27	PRRC1	Protein PRRC1 OS=Homo sapiens OX=9606 GN=PRRC1 PE=1 SV=1	5	1,295	1,631	1,879	4769799,86	5016123,991	2125762,96
P62873	GNB1	Guanine nucleotide-binding protein G(I)/G(S)/G(T) subunit beta-1 OS=Homo sapiens OX=9606 G	3	1,707	1,226	1,878	38854787,26	55580984,54	31058637,41
P06737	PYGL	Glycogen phosphorylase, liver form OS=Homo sapiens OX=9606 GN=PYGL PE=1 SV=4	23	1,802	1,027	1,877	22434985,84	30000129,62	18536466,06
P07203	GPX1	Glutathione peroxidase 1 OS=Homo sapiens OX=9606 GN=GPX1 PE=1 SV=4	5	2,404	0,879	1,876	5727416,573	18793489,28	5163066,825

Q9H4A4	RNPEP	Aminopeptidase B OS=Homo sapiens OX=9606 GN=RNPEP PE=1 SV=2	9	1,212	1,597	1,875	4886611,625	5746850,99	2797750,614
Q15293	RCN1	Reticulocalbin-1 OS=Homo sapiens OX=9606 GN=RCN1 PE=1 SV=1	17	1,339	1,353	1,873	73325609,49	106553302,2	55262248,26
Q96PU8	QKI	KH domain-containing RNA-binding protein QKI OS=Homo sapiens OX=9606 GN=QKI PE=1 SV=1	4	1,154	1,735	1,87	5792546,39	6230056,159	3246931,107
Q96RF0	SNX18	Sorting nexin-18 OS=Homo sapiens OX=9606 GN=SNX18 PE=1 SV=2	3	1,784	1,149	1,866	769554,0006	1038641,566	815109,7285
Q15642	TRIP10	Cdc42-interacting protein 4 OS=Homo sapiens OX=9606 GN=TRIP10 PE=1 SV=3	2	2,736	2,135	1,864	110246,9969	923964,3304	475522,876
P80188	LCN2	Neutrophil gelatinase-associated lipocalin OS=Homo sapiens OX=9606 GN=LCN2 PE=1 SV=2	5	2,604	0,716	1,864	117605,0237	109013,6369	1757226,734
Q8NBL1	POGLUT1	Protein O-glucosyltransferase 1 OS=Homo sapiens OX=9606 GN=POGLUT1 PE=1 SV=1	2	1,238	1,403	1,862	706765,0857	785143,3854	497900,2594
P00558	PGK1	Phosphoglycerate kinase 1 OS=Homo sapiens OX=9606 GN=PGK1 PE=1 SV=3	18	1,048	1,712	1,862	135204395,7	128064263,7	73491171,83
P42785	PRCP	Lysosomal Pro-X carboxypeptidase OS=Homo sapiens OX=9606 GN=PRCP PE=1 SV=1	4	1,042	1,72	1,858	4079098,966	4277101,872	2332986,873
O15116	LSM1	U6 snRNA-associated Sm-like protein LSm1 OS=Homo sapiens OX=9606 GN=LSM1 PE=1 SV=1	2	0,568	3,403	1,858	1879875,557	767454,2451	471146,1777
Q9NY65	TUBA8	Tubulin alpha-8 chain OS=Homo sapiens OX=9606 GN=TUBA8 PE=1 SV=1	2	0,328	6,155	1,858	29973643,04	9046338,361	4869643,858
O43768	ENSA	Alpha-endosulfine OS=Homo sapiens OX=9606 GN=ENSA PE=1 SV=1	2	0,25	7,22	1,858	4054089,244	999558,6994	267367,706
Q92538	GBF1	Golgi-specific brefeldin A-resistance guanine nucleotide exchange factor 1 OS=Homo sapiens OX	4	1,744	1,236	1,857	1523893,105	1289099,459	993439,9209
O75027	ABCB7	Iron-sulfur clusters transporter ABCB7, mitochondrial OS=Homo sapiens OX=9606 GN=ABCB7 PE	6	0,753	1,784	1,857	2005655,026	860583,5925	799253,0332
P06396	GSN	Gelsolin OS=Homo sapiens OX=9606 GN=GSN PE=1 SV=1	11	0,889	1,806	1,852	12239469	9599964,919	5203455,371
Q96AQ6	PBXIP1	Pre-B-cell leukemia transcription factor-interacting protein 1 OS=Homo sapiens OX=9606 GN=PB	4	0,948	1,724	1,849	1638062,205	1507993,927	987607,105
P13667	PDIA4	Protein disulfide-isomerase A4 OS=Homo sapiens OX=9606 GN=PDIA4 PE=1 SV=2	34	0,71	2,818	1,847	280748711,1	187570183,4	99227513,29
Q9H173	SIL1	Nucleotide exchange factor SIL1 OS=Homo sapiens OX=9606 GN=SIL1 PE=1 SV=1	2	2,795	0,659	1,846	282711,6096	790065,5542	428916,3878
O94851	MICAL2	[F-actin]-monooxygenase MICAL2 OS=Homo sapiens OX=9606 GN=MICAL2 PE=1 SV=2	4	1000	0,001	1,842		1188087,308	971351,7776
P29372	MPG	DNA-3-methyladenine glycosylase OS=Homo sapiens OX=9606 GN=MPG PE=1 SV=3	2	2,106	0,831	1,841	368285,1318	731730,2469	498446,4831
P25788	PSMA3	Proteasome subunit alpha type-3 OS=Homo sapiens OX=9606 GN=PSMA3 PE=1 SV=2	7	1,181	1,433	1,84	17498463,03	21976834,21	11411334,53
Q9NRW7	VPS45	Vacuolar protein sorting-associated protein 45 OS=Homo sapiens OX=9606 GN=VPS45 PE=1 SV=	2	2,797	0,638	1,839	368442,0446	1036345,483	602412,8264
P45877	PPIC	Peptidyl-prolyl cis-trans isomerase C OS=Homo sapiens OX=9606 GN=PPIC PE=1 SV=1	2	2,826	0,663	1,838	1455795,047	4031328,826	2136189,138
P29317	EPHA2	Ephrin type-A receptor 2 OS=Homo sapiens OX=9606 GN=EPHA2 PE=1 SV=2	8	2,344	0,769	1,833	5034069,657	10103028,81	6429390,105
P49591	SARS1	Serine--tRNA ligase, cytoplasmic OS=Homo sapiens OX=9606 GN=SARS1 PE=1 SV=3	12	0,361	5,134	1,833	25623203,32	9548396,541	4749395,939
Q9NRY5	FAM114A2	Protein FAM114A2 OS=Homo sapiens OX=9606 GN=FAM114A2 PE=1 SV=4	2	1,489	1,225	1,832	668706,1197	876833,3633	484784,2714
Q9Y678	COPG1	Coatomer subunit gamma-1 OS=Homo sapiens OX=9606 GN=COPG1 PE=1 SV=1	14	1,695	1,251	1,831	10279175,14	16670645,25	8978466,474
Q8IXK0	PHC2	Polyhomeotic-like protein 2 OS=Homo sapiens OX=9606 GN=PHC2 PE=1 SV=1	4	1,025	1,1	1,831	2519643,576	4308177,468	2999669,686
O43657	TSPAN6	Tetraspanin-6 OS=Homo sapiens OX=9606 GN=TSPAN6 PE=1 SV=1	2	0,29	6,212	1,831	2235354,616	499076,8537	335190,0252
P10619	CTSA	Lysosomal protective protein OS=Homo sapiens OX=9606 GN=CTSA PE=1 SV=2	5	4,211	0,341	1,83	2223037,188	9807197,085	5955021,817
O15155	BET1	BET1 homolog OS=Homo sapiens OX=9606 GN=BET1 PE=1 SV=1	2	3,042	0,615	1,83	1625782,286	4664195,964	2779668,588
Q8TAD4	SLC30A5	Proton-coupled zinc antiporter SLC30A5 OS=Homo sapiens OX=9606 GN=SLC30A5 PE=1 SV=1	2	1,504	1,088	1,83	233214,3146	620627,7858	364984,1602
Q14914	PTGR1	Prostaglandin reductase 1 OS=Homo sapiens OX=9606 GN=PTGR1 PE=1 SV=2	6	2,738	0,658	1,826	484375,2655	1959668,713	1088450,715
Q9NRR5	UBQLN4	Ubiquilin-4 OS=Homo sapiens OX=9606 GN=UBQLN4 PE=1 SV=2	4	0,912	2,024	1,824	858480,4605	694504,9567	407177,8139
P25786	PSMA1	Proteasome subunit alpha type-1 OS=Homo sapiens OX=9606 GN=PSMA1 PE=1 SV=1	8	1,31	1,404	1,82	19696148,52	25137991,53	13340560,87
O14974	PPP1R12A	Protein phosphatase 1 regulatory subunit 12A OS=Homo sapiens OX=9606 GN=PPP1R12A PE=1 S	9	1,686	1,01	1,819	8783897,177	6942731,585	11261020,57
Q9H074	PAIP1	Polyadenylate-binding protein-interacting protein 1 OS=Homo sapiens OX=9606 GN=PAIP1 PE=1	5	0,637	3,293	1,817	9711683,063	6114247,469	1834878,252
P04632	CAPNS1	Calpain small subunit 1 OS=Homo sapiens OX=9606 GN=CAPNS1 PE=1 SV=1	5	4,362	0,395	1,816	3380996,563	13159158,44	5145372,652
Q53T59	HS1BP3	HCLS1-binding protein 3 OS=Homo sapiens OX=9606 GN=HS1BP3 PE=1 SV=1	2	0,567	2,893	1,813	501678,5931	283735,3869	157566,7229
P30520	ADSS2	Adenylosuccinate synthetase isozyme 2 OS=Homo sapiens OX=9606 GN=ADSS2 PE=1 SV=3	4	1,426	1,273	1,812	2232805,003	2830558,248	1752422,988
Q9NQW7	XPNPEP1	Xaa-Pro aminopeptidase 1 OS=Homo sapiens OX=9606 GN=XPNPEP1 PE=1 SV=3	5	1,361	1,356	1,81	2629106,289	3832703,204	2359311,505
P07197	NEFM	Neurofilament medium polypeptide OS=Homo sapiens OX=9606 GN=NEFM PE=1 SV=3	23	0,053	19,872	1,805	79890034,49	2620938,373	1780917,706
P45974	USP5	Ubiquitin carboxyl-terminal hydrolase 5 OS=Homo sapiens OX=9606 GN=USP5 PE=1 SV=2	16	0,552	3,702	1,804	17526157,19	10251393,99	4839520,984
Q15436	SEC23A	Protein transport protein Sec23A OS=Homo sapiens OX=9606 GN=SEC23A PE=1 SV=2	8	1,288	1,659	1,801	9091318,253	10985582,11	5668425,815
P49189	ALDH9A1	4-trimethylaminobutyraldehyde dehydrogenase OS=Homo sapiens OX=9606 GN=ALDH9A1 PE=1	8	1,24	1,299	1,801	9424409,049	11473200,38	5994548,27
Q07960	ARHGAP1	Rho GTPase-activating protein 1 OS=Homo sapiens OX=9606 GN=ARHGAP1 PE=1 SV=1	11	1,423	1,296	1,8	6053147,176	10479726,56	5294513,527
Q14258	TRIM25	E3 ubiquitin/ISG15 ligase TRIM25 OS=Homo sapiens OX=9606 GN=TRIM25 PE=1 SV=2	12	3,261	0,588	1,799	2845855,99	8046970,772	7029456,425
Q92896	GLG1	Golgi apparatus protein 1 OS=Homo sapiens OX=9606 GN=GLG1 PE=1 SV=2	5	1,873	0,893	1,796	4888328,248	4519454,555	5528249,552
Q8NBF2	NHLRC2	NHL repeat-containing protein 2 OS=Homo sapiens OX=9606 GN=NHLRC2 PE=1 SV=1	6	0,367	7,052	1,795	2087403,67	688390,2552	151282,0051
Q16610	ECM1	Extracellular matrix protein 1 OS=Homo sapiens OX=9606 GN=ECM1 PE=1 SV=2	2	8,435	0,213	1,793	67554,94721	1207719,099	317840,1284
P28070	PSMB4	Proteasome subunit beta type-4 OS=Homo sapiens OX=9606 GN=PSMB4 PE=1 SV=4	6	1,205	1,487	1,792	12527669,77	12671709,89	12380836,06
P42224	STAT1	Signal transducer and activator of transcription 1-alpha/beta OS=Homo sapiens OX=9606 GN=ST	27	4,084	0,414	1,79	17928619,03	73363656,94	43292493,99
P22033	MMUT	Methylmalonyl-CoA mutase, mitochondrial OS=Homo sapiens OX=9606 GN=MMUT PE=1 SV=4	10	1,302	1,284	1,789	6240094,042	9018939,65	5231346,367
P51153	RAB13	Ras-related protein Rab-13 OS=Homo sapiens OX=9606 GN=RAB13 PE=1 SV=1	5	3,134	0,544	1,784	946456,6983	5615799,892	3342026,821
Q68CQ7	GLT8D1	Glycosyltransferase 8 domain-containing protein 1 OS=Homo sapiens OX=9606 GN=GLT8D1 PE=1	2	4,623	0,421	1,783	128061,3147	912338,9534	566415,6263
Q13636	RAB31	Ras-related protein Rab-31 OS=Homo sapiens OX=9606 GN=RAB31 PE=1 SV=2	2	0,824	2,261	1,78	2350213,594	1931513,483	1194335,832
P61106	RAB14	Ras-related protein Rab-14 OS=Homo sapiens OX=9606 GN=RAB14 PE=1 SV=4	10	3,853	0,54	1,779	5460337,072	20450512,9	11998386,86
P46778	RPL21	Large ribosomal subunit protein eL21 OS=Homo sapiens OX=9606 GN=RPL21 PE=1 SV=2	4	0,509	4,424	1,777	24969305,71	12240304,6	14072386,13
Q8NC56	LEMD2	LEM domain-containing protein 2 OS=Homo sapiens OX=9606 GN=LEMD2 PE=1 SV=1	9	1,311	1,38	1,776	5657160,059	6977339,062	4143055,789
P04844	RPN2	Dolichyl-diphosphooligosaccharide--protein glycosyltransferase subunit 2 OS=Homo sapiens OX=	22	1,151	1,511	1,775	113016275,1	129612324	74012024,46
Q10471	GALNT2	Polypeptide N-acetylgalactosaminyltransferase 2 OS=Homo sapiens OX=9606 GN=GALNT2 PE=1	11	3,06	0,572	1,774	5601544,284	17908312,7	9614399,03
Q9H1E5	TMX4	Thioredoxin-related transmembrane protein 4 OS=Homo sapiens OX=9606 GN=TMX4 PE=1 SV=1	4	1,874	0,917	1,772	1586243,84	3214197,257	1582023,014
P61764	STXBP1	Syntaxin-binding protein 1 OS=Homo sapiens OX=9606 GN=STXBP1 PE=1 SV=1	3	1,029	1,551	1,772	1375996,341	1381062,294	848037,618
P55265	ADAR	Double-stranded RNA-specific adenosine deaminase OS=Homo sapiens OX=9606 GN=ADAR PE=1	24	2,333	0,718	1,771	20308714,97	49301544,67	32510505,55
O60749	SNX2	Sorting nexin-2 OS=Homo sapiens OX=9606 GN=SNX2 PE=1 SV=2	9	0,482	2,809	1,769	7170769,977	3511906,385	2555169,137
Q86WV6	STING1	Stimulator of interferon genes protein OS=Homo sapiens OX=9606 GN=STING1 PE=1 SV=1	2	1000	0,001	1,768		1616139,726	929902,1504
Q96T76	MMS19	MMS19 nucleotide excision repair protein homolog OS=Homo sapiens OX=9606 GN=MMS19 PE=	3	0,641	2,567	1,767	1096766,304	473777,3065	469874,2288
P78417	GSTO1	Glutathione S-transferase omega-1 OS=Homo sapiens OX=9606 GN=GSTO1 PE=1 SV=2	10	1	1,927	1,766	19990424	16125490,92	10694193,54
Q01658	DR1	Protein Dr1 OS=Homo sapiens OX=9606 GN=DR1 PE=1 SV=1	2	0,798	2,209	1,764	1871387,871	1476321,461	891896,6702
P30085	CMPK1	UMP-CMP kinase OS=Homo sapiens OX=9606 GN=CMPK1 PE=1 SV=3	8	0,714	2,052	1,761	5113801,263	3147169,772	2114444,226
Q9BRG1	VPS25	Vacuolar protein-sorting-associated protein 25 OS=Homo sapiens OX=9606 GN=VPS25 PE=1 SV=	2	0,555	4,508	1,761	1118879,219	533587,3557	195270,2281
P26639	TARS1	Threonine--tRNA ligase 1, cytoplasmic OS=Homo sapiens OX=9606 GN=TARS1 PE=1 SV=3	22	0,49	3,958	1,761	58531030	24720336,1	13035415,48
P34897	SHMT2	Serine hydroxymethyltransferase, mitochondrial OS=Homo sapiens OX=9606 GN=SHMT2 PE=1 S\	19	0,741	2,025	1,758	87094095,08	67339926,45	39433218,59
Q92616	GCN1	Stalled ribosome sensor GCN1 OS=Homo sapiens OX=9606 GN=GCN1 PE=1 SV=7	44	0,808	2,06	1,756	27113751,46	22782011,21	12243268,91
O14936	CASK	Peripheral plasma membrane protein CASK OS=Homo sapiens OX=9606 GN=CASK PE=1 SV=3	15	1,643	1,153	1,755	4942337,313	10381763,44	4787304,381
Q14697	GANAB	Neutral alpha-glucosidase AB OS=Homo sapiens OX=9606 GN=GANAB PE=1 SV=3	36	1,478	1,304	1,755	203132246,5	267943918,4	159316604
Q16401	PSMD5	26S proteasome non-ATPase regulatory subunit 5 OS=Homo sapiens OX=9606 GN=PSMD5 PE=1	8	1,462	1,258	1,755	2194856,399	3271036,572	1580728,77
Q9Y237	PIN4	Peptidyl-prolyl cis-trans isomerase NIMA-interacting 4 OS=Homo sapiens OX=9606 GN=PIN4 PE=	3	0,239	7,317	1,755	3267628,419	979832,3704	852154,3966
O75976	CPD	Carboxypeptidase D OS=Homo sapiens OX=9606 GN=CPD PE=1 SV=2	9	0,958	1,824	1,754	2812935,864	3028908,713	1898930,697
P35610	SOAT1	Sterol O-acyltransferase 1 OS=Homo sapiens OX=9606 GN=SOAT1 PE=1 SV=3	3	2,211	0,893	1,749	2116985,637	2791963,595	2225800,662
Q7L5N7	LPCAT2	Lysophosphatidylcholine acyltransferase 2 OS=Homo sapiens OX=9606 GN=LPCAT2 PE=1 SV=1	9	1,519	0,338	1,749	1442505,54	10122505,54	4808731,413
P98172	EFNB1	Ephrin-B1 OS=Homo sapiens OX=9606 GN=EFNB1 PE=1 SV=1	5	0,287	2,495	1,749	2986905,082	1153225,904	401505,0178
Q9Y6E2	BZW2	eIF5-mimic protein 1 OS=Homo sapiens OX=9606 GN=BZW2 PE=1 SV=1	5	0,455	4,131	1,745	6750902,798	2660659,698	1600660,368
P35611	ADD1	Alpha-adducin OS=Homo sapiens OX=9606 GN=ADD1 PE=1 SV=2	5	1,08	1,154	1,743	2200560,045	2354352,034	1979736,707
P04083	ANXA1	Annexin A1 OS=Homo sapiens OX=9606 GN=ANXA1 PE=1 SV=2	19	5,962	0,31	1,742	42157131,2	236563154	135698673,1
O75347	TBCA	Tubulin-specific chaperone A OS=Homo sapiens OX=9606 GN=TBCA PE=1 SV=3	11	0,309	5,711	1,741	47329060,06	15175188,23	8206219,517
Q15125	EBP	3-beta-hydroxysteroid-Delta(8),Delta(7)-isomerase OS=Homo sapiens OX=9606 GN=EBP PE=1 SV	3	1,41	0,694	1,738	4701813,976	2892334,783	7512347,65
Q6P9B6	MEAK7	MTOR-associated protein MEAK7 OS=Homo sapiens OX=9606 GN=MEAK7 PE=1 SV=2	2	7,053	0,296	1,737	118073,9119	884190,5661	579888,5205
P63151	PPP2R2A	Serine/threonine-protein phosphatase 2A 55 kDa regulatory subunit B alpha isoform OS=Homo s	7	0,523	2,121	1,737	4799136,802	4857902,513	1920552,588
Q86Y39	NDUFA11	NADH dehydrogenase [ubiquinone] 1 alpha subcomplex subunit 11 OS=Homo sapiens OX=9606	2	3,027	0,557	1,736	133977,8658	405536,772	285515,4003
P38435	GGCX	Vitamin K-dependent gamma-carboxylase OS=Homo sapiens OX=9606 GN=GGCX PE=1 SV=2	2	1000	0,001	1,728		620460,7976	580108,4689
Q00535	CDK5	Cyclin-dependent kinase 5 OS=Homo sapiens OX=9606 GN=CDK5 PE=1 SV=3	2	0,808	2,081	1,728	627348,986	477362,0116	287456,0758
Q9NR12	PDLIM7	PDZ and LIM domain protein 7 OS=Homo sapiens OX=9606 GN=PDLIM7 PE=1 SV=1	10	2,427	0,573	1,727	7856628,342	19903162,51	13450125,16
Q8N2K0	ABHD12	Lysophosphatidylserine lipase ABHD12 OS=Homo sapiens OX=9606 GN=ABHD12 PE=1 SV=2	4	1,267	1,293	1,727	4776958,022	4954569,634	3674166,725
O14735	CDIPT	CDP-diacylglycerol--inositol 3-phosphatidyltransferase OS=Homo sapiens OX=9606 GN=CDIPT PE	2	6,859	0,271	1,725	123051,8416	804629,8109	467294,556
P10644	PRKAR1A	cAMP-dependent protein kinase type I-alpha regulatory subunit OS=Homo sapiens OX=9606 GN	7	2,052	0,784	1,724	4690652,461	9644278,045	5800015,642
P14625	HSP90B1	Endoplasmin OS=Homo sapiens OX=9606 GN=HSP90B1 PE=1 SV=1	43	0,933	1,841	1,724	796320639,5	681698827	399923481,4
P33176	KIF5B	Kinesin-1 heavy chain OS=Homo sapiens OX=9606 GN=KIF5B PE=1 SV=1	12	0,822	2,15	1,724	18211527,55	14167684,89	8054712,614
Q5SW79	CEP170	Centrosomal protein of 170 kDa OS=Homo sapiens OX=9606 GN=CEP170 PE=1 SV=1	5	0,801	2,371	1,723	1911972,899	1572680,674	579813,8276
Q16539	MAPK14	Mitogen-activated protein kinase 14 OS=Homo sapiens OX=9606 GN=MAPK14 PE=1 SV=3	3	1,126	1,871	1,722	1064621,437	1288645,481	305555,8454
P60468	SEC61B	Protein transport protein Sec61 subunit beta OS=Homo sapiens OX=9606 GN=SEC61B PE=1 SV=2	2	1,929	0,819	1,721	6864464,281	15631719,22	9157177,325
P05161	ISG15	Ubiquitin-like protein ISG15 OS=Homo sapiens OX=9606 GN=ISG15 PE=1 SV=5	4	1,128	1,582	1,718	49375340,75	57644082,23	32319212,81
O15260	SURF4	Surfeit locus protein 4 OS=Homo sapiens OX=9606 GN=SURF4 PE=1 SV=3	3	1,787	0,941	1,716	4234346,631	7644052,191	5032606,29
P04843	RPN1	Dolichyl-diphosphooligosaccharide--protein glycosyltransferase subunit 1 OS=Homo sapiens OX=	23	1,351	1,498	1,716	146349718,4	167688930,2	108210602,3
Q92542	NCSTN	Nicastrin OS=Homo sapiens OX=9606 GN=NCSTN PE=1 SV=2	6	1,775	0,986	1,715	4983040,964	8914553,171	5710917,998
P46977	STT3A	Dolichyl-diphosphooligosaccharide--protein glycosyltransferase subunit STT3A OS=Homo sapiens	12	1,128	1,382	1,714	25928067,31	25044569,34	16641097,09
P43897	TSFM	Elongation factor Ts, mitochondrial OS=Homo sapiens OX=9606 GN=TSFM PE=1 SV=2	6	1,55	1,115	1,713	3244917,441	6319017,176	2884327,552
Q15382	RHEB	GTP-binding protein Rheb OS=Homo sapiens OX=9606 GN=RHEB PE=1 SV=1	4	0,93	1,984	1,711	3809861,93	3492793,409	2379540,261
Q12888	TP53BP1	TP53-binding protein 1 OS=Homo sapiens OX=9606 GN=TP53BP1 PE=1 SV=2	17	1,095	1,442	1,71	9667148,667	11328647,33	7043232,106
P05067	APP	Amyloid-beta precursor protein OS=Homo sapiens OX=9606 GN=APP PE=1 SV=3	3	1,038	1,75	1,71	1884633,019	1900990,749	1330968,107
Q9GZU8	PSME3IP1	PSME3-interacting protein OS=Homo sapiens OX=9606 GN=PSME3IP1 PE=1 SV=1	2	0,876	1,897	1,709	1203452,556	1071232,454	617539,3533

P31937	HIBADH	3-hydroxyisobutyrate dehydrogenase, mitochondrial OS=Homo sapiens OX=9606 GN=HIBADH PE	11	4,216	0,391	1,708	4308971,301	14897574,94	8596756,818
Q9Y6K5	OAS3	2'-5'-oligoadenylate synthase 3 OS=Homo sapiens OX=9606 GN=OAS3 PE=1 SV=3	15	6,286	0,206	1,705	2972325,305	22732372,31	17362878,82
Q16186	ADRM1	Proteasomal ubiquitin receptor ADRM1 OS=Homo sapiens OX=9606 GN=ADRM1 PE=1 SV=2	4	0,844	1,629	1,704	2014944,34	1558726,223	1135873,109
Q96CN7	ISOC1	Isochorismatase domain-containing protein 1 OS=Homo sapiens OX=9606 GN=ISOC1 PE=1 SV=3	4	0,82	1,943	1,704	2736312,076	2123101,283	1493505,811
Q13951	CBFB	Core-binding factor subunit beta OS=Homo sapiens OX=9606 GN=CBFB PE=1 SV=2	2	0,871	1,917	1,703	4316359,38	3741100,538	2141610,451
Q9H3Q1	CDC42EP4	Cdc42 effector protein 4 OS=Homo sapiens OX=9606 GN=CDC42EP4 PE=1 SV=1	2	0,584	2,498	1,702	981657,3555	642983,5916	263182,5168
O00182	LGALS9	Galectin-9 OS=Homo sapiens OX=9606 GN=LGALS9 PE=1 SV=2	3	2,149	0,791	1,699	1406861,899	4481095,984	1705543,36
Q00610	CLTC	Clathrin heavy chain 1 OS=Homo sapiens OX=9606 GN=CLTC PE=1 SV=5	61	1,61	1,095	1,698	178208306,3	275011169,9	169206662,7
P39656	DDOST	Dolichyl-diphosphooligosaccharide--protein glycosyltransferase 48 kDa subunit OS=Homo sapier	14	1,279	1,337	1,697	70814669,23	76136525,39	45977482,33
P00973	OAS1	2'-5'-oligoadenylate synthase 1 OS=Homo sapiens OX=9606 GN=OAS1 PE=1 SV=4	4	1000	0,001	1,696		417908,8952	217429,0424
P62244	RPS15A	Small ribosomal subunit protein uS8 OS=Homo sapiens OX=9606 GN=RPS15A PE=1 SV=2	6	0,86	2,076	1,696	21047989,15	15884056,44	9694457,098
P20618	PSMB1	Proteasome subunit beta type-1 OS=Homo sapiens OX=9606 GN=PSMB1 PE=1 SV=2	6	1,285	1,269	1,695	18318170,51	21863921,24	16942151,73
Q9UMX5	NENF	Neudesin OS=Homo sapiens OX=9606 GN=NENF PE=1 SV=1	4	2,579	0,612	1,687	3367539,489	9017471,739	5336709,426
P30048	PRDX3	Thioredoxin-dependent peroxide reductase, mitochondrial OS=Homo sapiens OX=9606 GN=PRD	8	1,871	0,92	1,685	37078141,77	61273794,24	33235139,11
Q9UBE0	SAE1	SUMO-activating enzyme subunit 1 OS=Homo sapiens OX=9606 GN=SAE1 PE=1 SV=1	9	0,865	1,698	1,685	5620772,624	7063675,668	2666404,263
A1X283	SH3PXD2B	SH3 and PX domain-containing protein 2B OS=Homo sapiens OX=9606 GN=SH3PXD2B PE=1 SV=3	5	0,524	3,923	1,684	3451913,412	1529313,201	866743,6449
Q13217	DNAJC3	DnaJ homolog subfamily C member 3 OS=Homo sapiens OX=9606 GN=DNAJC3 PE=1 SV=1	10	1,112	1,49	1,683	7441633,513	10850626,14	4737090,978
P61981	YWHAG	14-3-3 protein gamma OS=Homo sapiens OX=9606 GN=YWHAG PE=1 SV=2	6	0,792	1,894	1,681	38940017,46	36165081,45	20375399,13
O75787	ATP6AP2	Renin receptor OS=Homo sapiens OX=9606 GN=ATP6AP2 PE=1 SV=2	3	1,186	1,338	1,677	1380046,959	1511529,474	969994,8439
P51159	RAB27A	Ras-related protein Rab-27A OS=Homo sapiens OX=9606 GN=RAB27A PE=1 SV=3	2	5,352	0,212	1,674	258399,2935	1762220,85	1024489,534
O95299	NDUFA10	NADH dehydrogenase [ubiquinone] 1 alpha subcomplex subunit 10, mitochondrial OS=Homo sap	6	1,857	0,623	1,673	2214259,67	4157975,914	3696540,833
P42677	RPS27	Small ribosomal subunit protein eS27 OS=Homo sapiens OX=9606 GN=RPS27 PE=1 SV=3	3	0,724	2,35	1,671	17245218,9	11783205,89	8663106,837
P04899	GNAI2	Guanine nucleotide-binding protein G(i) subunit alpha-2 OS=Homo sapiens OX=9606 GN=GNAI2	5	1,758	0,825	1,669	19040663,03	31865717,77	21244212,47
Q8NBS9	TXNDC5	Thioredoxin domain-containing protein 5 OS=Homo sapiens OX=9606 GN=TXNDC5 PE=1 SV=2	13	1,625	1,313	1,669	23945256,16	37864369,08	16262281,54
Q5TFE4	NT5DC1	5'-nucleotidase domain-containing protein 1 OS=Homo sapiens OX=9606 GN=NT5DC1 PE=1 SV=1	2	1,831	0,886	1,668	782338,2341	1434330,712	833468,1977
P78318	IGBP1	Immunoglobulin-binding protein 1 OS=Homo sapiens OX=9606 GN=IGBP1 PE=1 SV=1	2	1,252	1,289	1,666	584662,0373	731925,4795	466131,2742
P46379	BAG6	Large proline-rich protein BAG6 OS=Homo sapiens OX=9606 GN=BAG6 PE=1 SV=2	10	0,555	3,046	1,666	10718711,81	4964149,654	3564765,509
Q9UJA5	TRMT6	tRNA (adenine(58)-N(1))-methyltransferase non-catalytic subunit TRM6 OS=Homo sapiens OX=9	4	0,728	2,67	1,665	901660,0601	1176838,022	577969,2419
Q15369	ELOC	Elongin-C OS=Homo sapiens OX=9606 GN=ELOC PE=1 SV=1	2	0,713	2,409	1,665	7647634,692	6226651,06	3191684,363
P19623	SRM	Spermidine synthase OS=Homo sapiens OX=9606 GN=SRM PE=1 SV=1	4	0,656	3,476	1,664	6665975,679	3931735,892	3181372,532
P51572	BCAP31	B-cell receptor-associated protein 31 OS=Homo sapiens OX=9606 GN=BCAP31 PE=1 SV=3	12	1,393	1,105	1,663	33909974,93	46758032,38	34695033,67
O00487	PSMD14	26S proteasome non-ATPase regulatory subunit 14 OS=Homo sapiens OX=9606 GN=PSMD14 PE=	6	0,857	1,994	1,663	6226174,624	5504707,805	4133222,881
P53618	COPB1	Coatomer subunit beta OS=Homo sapiens OX=9606 GN=COPB1 PE=1 SV=3	23	1,223	1,396	1,662	25707642,33	30415963,12	20479625,69
P63172	DYNLT1	Dynein light chain Tctex-type 1 OS=Homo sapiens OX=9606 GN=DYNLT1 PE=1 SV=1	2	0,595	3,302	1,662	2847201,451	1582085,459	1016917,249
Q15746	MYLK	Myosin light chain kinase, smooth muscle OS=Homo sapiens OX=9606 GN=MYLK PE=1 SV=4	4	2,781	0,653	1,66	541696,028	1742078,856	318089,4687
Q9UKK9	NUDT5	ADP-sugar pyrophosphatase OS=Homo sapiens OX=9606 GN=NUDT5 PE=1 SV=1	5	0,492	1,926	1,659	4293866,644	5419018,697	3288114,601
P14550	AKR1A1	Aldo-keto reductase family 1 member A1 OS=Homo sapiens OX=9606 GN=AKR1A1 PE=1 SV=3	9	0,475	4,519	1,657	10032552,37	3276833,684	2354064,382
Q9H9A6	LRRC40	Leucine-rich repeat-containing protein 40 OS=Homo sapiens OX=9606 GN=LRRC40 PE=1 SV=1	5	0,432	3,703	1,657	3532544,929	1320143,75	790871,2499
P04062	GBA1	Lysosomal acid glucosylceramidase OS=Homo sapiens OX=9606 GN=GBA1 PE=1 SV=3	6	3,57	0,406	1,654	1933527,911	5370515,803	5090509,636
Q15717	ELAVL1	ELAV-like protein 1 OS=Homo sapiens OX=9606 GN=ELAVL1 PE=1 SV=2	11	0,915	1,731	1,653	42246789,2	38646558,22	25942181,86
Q16698	DECR1	2,4-dienoyl-CoA reductase [(3E)-enoyl-CoA-producing], mitochondrial OS=Homo sapiens OX=960	10	1,782	0,887	1,651	10106267,06	17489585,93	12679345,78
O75396	SEC22B	Vesicle-trafficking protein SEC22b OS=Homo sapiens OX=9606 GN=SEC22B PE=1 SV=5	9	1,485	1,144	1,648	25567714,44	40239979,93	22129141,23
O95486	SEC24A	Protein transport protein Sec24A OS=Homo sapiens OX=9606 GN=SEC24A PE=1 SV=2	2	0,958	1,665	1,648	592373,8466	566742,7363	346261,7764
P04818	TYMS	Thymidylate synthase OS=Homo sapiens OX=9606 GN=TYMS PE=1 SV=3	6	0,186	10,026	1,648	6578345,282	1049541,453	329559,3412
Q92974	ARHGEF2	Rho guanine nucleotide exchange factor 2 OS=Homo sapiens OX=9606 GN=ARHGEF2 PE=1 SV=4	2	0,669	2,461	1,647	808044,3859	529357,3962	359254,3856
P38606	ATP6V1A	V-type proton ATPase catalytic subunit A OS=Homo sapiens OX=9606 GN=ATP6V1A PE=1 SV=2	10	0,666	2,497	1,645	11785549,86	6627832,149	4939293,881
Q6KB66	KRT80	Keratin, type II cytoskeletal 80 OS=Homo sapiens OX=9606 GN=KRT80 PE=1 SV=2	5	6,32	0,265	1,644	240744,5006	1809433,343	2459048,458
A0FGR8	ESYT2	Extended synaptotagmin-2 OS=Homo sapiens OX=9606 GN=ESYT2 PE=1 SV=1	16	3,65	0,463	1,642	3813899,939	17062879,98	9638108,235
P53396	ACLY	ATP-citrate synthase OS=Homo sapiens OX=9606 GN=ACLY PE=1 SV=3	31	0,331	4,718	1,641	86621418,53	29134480,69	18720034,28
Q9NXU5	ARL15	ADP-ribosylation factor-like protein 15 OS=Homo sapiens OX=9606 GN=ARL15 PE=1 SV=1	6	0,778	2,063	1,639	1040165,987	825979,0057	694828,8884
Q658P3	STEAP3	Metalloreductase STEAP3 OS=Homo sapiens OX=9606 GN=STEAP3 PE=1 SV=2	3	4,034	0,338	1,638	52838,84657	796496,5459	514801,6546
A0A7I2V508	BAG6	Large proline-rich protein BAG6 OS=Homo sapiens OX=9606 GN=BAG6 PE=1 SV=1	10	0,48	3,439	1,637	10379408,18	4153546,714	3069259,669
Q9UBQ0	VPS29	Vacuolar protein sorting-associated protein 29 OS=Homo sapiens OX=9606 GN=VPS29 PE=1 SV=	3	1,079	1,516	1,635	5872181,817	6388498,268	3929003,619
A0A994J6T1	RAC1	Ras-related C3 botulinum toxin substrate 1 OS=Homo sapiens OX=9606 GN=RAC1 PE=1 SV=1	2	1,908	1,065	1,634	9096480,206	14782669,35	10001335,3
Q9NZD8	SPG21	Maspardin OS=Homo sapiens OX=9606 GN=SPG21 PE=1 SV=1	3	0,702	2,764	1,634	150201,7653	585818,8842	359452,6425
P51648	ALDH3A2	Aldehyde dehydrogenase family 3 member A2 OS=Homo sapiens OX=9606 GN=ALDH3A2 PE=1 S	9	1,062	1,356	1,631	10726023	10793985,82	7882195,795
Q15019	SEPTIN2	Septin-2 OS=Homo sapiens OX=9606 GN=SEPTIN2 PE=1 SV=1	13	0,706	2,245	1,631	39284397,99	28063963,84	15501867,3
O15230	LAMA5	Laminin subunit alpha-5 OS=Homo sapiens OX=9606 GN=LAMA5 PE=1 SV=8	2	2,512	0,653	1,629	257946,1341	338575,9938	207829,4005
Q14558	PRPSAP1	Phosphoribosyl pyrophosphate synthase-associated protein 1 OS=Homo sapiens OX=9606 GN=P	3	0,512	3,178	1,628	3534787,149	990031,4819	750328,3758
Q13586	STIM1	Stromal interaction molecule 1 OS=Homo sapiens OX=9606 GN=STIM1 PE=1 SV=3	9	3,242	0,509	1,626	1430925,102	7258005,394	5010019,79
P12074	COX6A1	Cytochrome c oxidase subunit 6A1, mitochondrial OS=Homo sapiens OX=9606 GN=COX6A1 PE=1	2	1,648	0,966	1,626	3410541,516	5296575,141	3244881,704
P04075	ALDOA	Fructose-bisphosphate aldolase A OS=Homo sapiens OX=9606 GN=ALDOA PE=1 SV=2	15	1,845	0,906	1,625	115641228,4	217073358,5	131936966,8
Q9NZ32	ACTR10	Actin-related protein 10 OS=Homo sapiens OX=9606 GN=ACTR10 PE=1 SV=1	2	0,526	4,497	1,625	802662,4953	330122,6644	201325,281
P28066	PSMA5	Proteasome subunit alpha type-5 OS=Homo sapiens OX=9606 GN=PSMA5 PE=1 SV=3	8	1,104	1,617	1,623	33260899,63	34703124,22	22050002,11
Q9BWJ5	SF3B5	Splicing factor 3B subunit 5 OS=Homo sapiens OX=9606 GN=SF3B5 PE=1 SV=1	2	0,98	1,778	1,623	2137430,862	2072194,155	1163959,312
Q71RC2	LARP4	La-related protein 4 OS=Homo sapiens OX=9606 GN=LARP4 PE=1 SV=3	2	0,969	1,866	1,622	608124,7952	593950,0475	400313,0439
O14579	COPE	Coatomer subunit epsilon OS=Homo sapiens OX=9606 GN=COPE PE=1 SV=3	4	1,284	1,082	1,621	3809381,312	4740408,314	2870757,625
Q12929	EPS8	Epidermal growth factor receptor kinase substrate 8 OS=Homo sapiens OX=9606 GN=EPS8 PE=1	5	0,446	1,585	1,621	1429994,889	543703,7096	695097,2199
Q96A49	SYAP1	Synapse-associated protein 1 OS=Homo sapiens OX=9606 GN=SYAP1 PE=1 SV=1	9	0,983	1,666	1,62	4332502,339	4155908,2	2139869,394
Q93008	USP9X	Probable ubiquitin carboxyl-terminal hydrolase FAF-X OS=Homo sapiens OX=9606 GN=USP9X PE	6	0,639	2,009	1,62	1376527,042	864571,9157	490916,6288
P21283	ATP6V1C1	V-type proton ATPase subunit C 1 OS=Homo sapiens OX=9606 GN=ATP6V1C1 PE=1 SV=4	3	1,181	1,226	1,618	767371,6358	868365,9705	592204,177
P26599	PTBP1	Polypyrimidine tract-binding protein 1 OS=Homo sapiens OX=9606 GN=PTBP1 PE=1 SV=1	14	0,849	1,854	1,618	100586237,5	89820235,89	55055048,31
Q8IVU3	HERC6	Probable E3 ubiquitin-protein ligase HERC6 OS=Homo sapiens OX=9606 GN=HERC6 PE=1 SV=2	2	1,524	0,803	1,616	196895,2072	288707,6952	260124,0072
P11441	UBL4A	Ubiquitin-like protein 4A OS=Homo sapiens OX=9606 GN=UBL4A PE=1 SV=1	5	0,749	2,624	1,614	2698024,839	1838498,4	959732,0613
Q9BVA1	TUBB2B	Tubulin beta-2B chain OS=Homo sapiens OX=9606 GN=TUBB2B PE=1 SV=1	7	0,238	6,357	1,614	837942432,5	235811531,9	116805192,1
Q09028	RBBP4	Histone-binding protein RBBP4 OS=Homo sapiens OX=9606 GN=RBBP4 PE=1 SV=3	5	0,411	5,9	1,613	32844736,61	12769209,98	10328456,07
P29144	TPP2	Tripeptidyl-peptidase 2 OS=Homo sapiens OX=9606 GN=TPP2 PE=1 SV=4	22	0,954	1,642	1,612	17019759,45	12456105,85	8680193,93
Q14573	ITPR3	Inositol 1,4,5-trisphosphate receptor type 3 OS=Homo sapiens OX=9606 GN=ITPR3 PE=1 SV=2	21	7,769	0,212	1,61	2023904,139	9383450,525	7766170,271
Q5TC12	ATPAF1	ATP synthase mitochondrial F1 complex assembly factor 1 OS=Homo sapiens OX=9606 GN=ATPA	2	0,581	2,771	1,61	1005437,127	311560,9343	200482,9852
Q6DD88	ATL3	Atlastin-3 OS=Homo sapiens OX=9606 GN=ATL3 PE=1 SV=1	15	4,245	0,395	1,608	6997574,81	27716194,75	17900731,75
A0A494C0G5	AGRN	Agrin OS=Homo sapiens OX=9606 GN=AGRN PE=1 SV=1	2	3,914	0,405	1,608	94614,16479	375723,1957	233681,8801
Q15286	RAB35	Ras-related protein Rab-35 OS=Homo sapiens OX=9606 GN=RAB35 PE=1 SV=1	3	1,943	0,71	1,607	1649530,094	3259031,923	2110538,552
Q92945	KHSRP	Far upstream element-binding protein 2 OS=Homo sapiens OX=9606 GN=KHSRP PE=1 SV=4	20	0,92	1,944	1,607	72425783,25	64637961,47	35434590,09
Q9BTE7	DCUN1D5	DCN1-like protein 5 OS=Homo sapiens OX=9606 GN=DCUN1D5 PE=1 SV=1	2	0,562	3,049	1,604	592171,2817	363832,7569	52782,92132
Q6NUM9	RETSAT	All-trans-retinol 13,14-reductase OS=Homo sapiens OX=9606 GN=RETSAT PE=1 SV=2	3	4,863	0,367	1,603	190888,2124	1372115,553	934801,0628
A0A0A0MTS7	TTN	Titin OS=Homo sapiens OX=9606 GN=TTN PE=1 SV=1	2	0,714	2,083	1,602	21884658,38	15837284,31	10225046,23
Q9HAV4	XPO5	Exportin-5 OS=Homo sapiens OX=9606 GN=XPO5 PE=1 SV=1	17	0,288	6,008	1,6	12801597,44	3517510,41	2026179,637
Q99584	S100A13	Protein S100-A13 OS=Homo sapiens OX=9606 GN=S100A13 PE=1 SV=1	3	2,309	0,694	1,599	3535932,322	8177155,769	5642835,019
Q86UP2	KTN1	Kinectin OS=Homo sapiens OX=9606 GN=KTN1 PE=1 SV=1	52	1,908	0,834	1,599	35489733,5	64783434,56	39753558,38
Q9BTV4	TMEM43	Transmembrane protein 43 OS=Homo sapiens OX=9606 GN=TMEM43 PE=1 SV=1	18	2,451	0,717	1,598	19137625,21	41062605,51	25865830,25
Q9NT62	ATG3	Ubiquitin-like-conjugating enzyme ATG3 OS=Homo sapiens OX=9606 GN=ATG3 PE=1 SV=1	3	1,056	1,593	1,597	1896203,016	1906571,521	1210295,29
Q9NUP9	LIN7C	Protein lin-7 homolog C OS=Homo sapiens OX=9606 GN=LIN7C PE=1 SV=1	5	2,053	0,66	1,596	2489503,753	5874646,082	2746096,607
P54886	ALDH18A1	Delta-1-pyrroline-5-carboxylate synthase OS=Homo sapiens OX=9606 GN=ALDH18A1 PE=1 SV=2	27	1,035	1,466	1,595	35161176,84	29314711,67	24342879,84
Q8N9N7	LRRC57	Leucine-rich repeat-containing protein 57 OS=Homo sapiens OX=9606 GN=LRRC57 PE=1 SV=1	2	0,943	1,902	1,593	1256052,904	1325750,878	805071,479
P09960	LTA4H	Leukotriene A-4 hydrolase OS=Homo sapiens OX=9606 GN=LTA4H PE=1 SV=2	16	0,77	1,998	1,593	10760785,43	8345780,421	4752402,879
P15374	UCHL3	Ubiquitin carboxyl-terminal hydrolase isozyme L3 OS=Homo sapiens OX=9606 GN=UCHL3 PE=1 S	2	0,784	2,728	1,592	1027175,409	869919,2569	491734,4485
Q9BUT1	BDH2	Dehydrogenase/reductase SDR family member 6 OS=Homo sapiens OX=9606 GN=BDH2 PE=1 SV	2	0,21	7,632	1,591	2642894,704	299444,3581	188245,099
Q92692	NECTIN2	Nectin-2 OS=Homo sapiens OX=9606 GN=NECTIN2 PE=1 SV=1	6	2,477	0,695	1,59	1846122,658	5903664,438	2673551,954
P47755	CAPZA2	F-actin-capping protein subunit alpha-2 OS=Homo sapiens OX=9606 GN=CAPZA2 PE=1 SV=3	9	1,584	0,976	1,586	20035264,7	29415481,45	18876211,87
O60645	EXOC3	Exocyst complex component 3 OS=Homo sapiens OX=9606 GN=EXOC3 PE=1 SV=3	2	1,361	1,106	1,586	319111,6755	434171,0466	273683,4577
Q9BVC6	TMEM109	Voltage-gated monoatomic cation channel TMEM109 OS=Homo sapiens OX=9606 GN=TMEM10	2	1,597	0,949	1,585	16046830,93	24482384,59	15339087,88
Q9UBB6	NCDN	Neurochondrin OS=Homo sapiens OX=9606 GN=NCDN PE=1 SV=1	3	0,705	4,495	1,583	698558,6646	382485,9584	172537,6916
Q9UMR2	DDX19B	ATP-dependent RNA helicase DDX19B OS=Homo sapiens OX=9606 GN=DDX19B PE=1 SV=1	7	0,713	2,148	1,582	7893357,48	5759181,492	3461065,886
P48735	IDH2	Isocitrate dehydrogenase [NADP], mitochondrial OS=Homo sapiens OX=9606 GN=IDH2 PE=1 SV=	18	1,115	1,419	1,58	65875268,68	75973059,05	46786248,78
Q92973	TNPO1	Transportin-1 OS=Homo sapiens OX=9606 GN=TNPO1 PE=1 SV=2	9	0,898	1,754	1,579	16069048,36	11767655,72	7341798,373
Q07666	KHDRBS1	KH domain-containing, RNA-binding, signal transduction-associated protein 1 OS=Homo sapiens	8	0,795	1,935	1,576	68448361,47	57487339,36	37312177,98
P19224	UGT1A6	UDP-glucuronosyltransferase 1-6 OS=Homo sapiens OX=9606 GN=UGT1A6 PE=1 SV=2	11	2,682	0,085	1,574	291097,9259	4219570,869	7372470,541
O43237	DYNC1LI2	Cytoplasmic dynein 1 light intermediate chain 2 OS=Homo sapiens OX=9606 GN=DYNC1LI2 PE=1	7	1,033	1,598	1,574	4692137,698	4118612,302	2723374,55
P61619	SEC61A1	Protein transport protein Sec61 subunit alpha isoform 1 OS=Homo sapiens OX=9606 GN=SEC61A	6	1,841	1,045	1,573	17276600,8	32342748,66	18050232,39
Q7L5N1	COPS6	COP9 signalosome complex subunit 6 OS=Homo sapiens OX=9606 GN=COPS6 PE=1 SV=1	3	0,823	1,919	1,572	1638291,182	1296413,001	898467,6736
P23284	PPIB	Peptidyl-prolyl cis-trans isomerase B OS=Homo sapiens OX=9606 GN=PPIB PE=1 SV=2	13	1,208	1,453	1,569	142975365,2	165585422	101790001,8

O95994	AGR2	Anterior gradient protein 2 homolog OS=Homo sapiens OX=9606 GN=AGR2 PE=1 SV=1	7	1,527	0,972	1,566	604948,4816	1001418,282	348644,909
O14737	PDCD5	Programmed cell death protein 5 OS=Homo sapiens OX=9606 GN=PDCD5 PE=1 SV=3	4	0,755	1,977	1,565	5553564,068	4352607,428	2946607,565
Q14315	FLNC	Filamin-C OS=Homo sapiens OX=9606 GN=FLNC PE=1 SV=3	29	0,655	2,426	1,565	34472519,29	20141413,09	12804407,5
Q13526	PIN1	Peptidyl-prolyl cis-trans isomerase NIMA-interacting 1 OS=Homo sapiens OX=9606 GN=PIN1 PE=	3	0,511	3,034	1,565	2105015,765	1182286,823	373315,4755
Q15582	TGFBI	Transforming growth factor-beta-induced protein ig-h3 OS=Homo sapiens OX=9606 GN=TGFBI P	13	0,277	5,99	1,565	18611723,27	4449815,987	3410048,357
O76024	WFS1	Wolframin OS=Homo sapiens OX=9606 GN=WFS1 PE=1 SV=2	8	4,431	0,331	1,563	1539717,707	6400290,96	3340250,689
O43251	RBFOX2	RNA binding protein fox-1 homolog 2 OS=Homo sapiens OX=9606 GN=RBFOX2 PE=1 SV=3	4	0,802	2,244	1,562	4066272,569	2409085,127	1658710,479
Q99460	PSMD1	26S proteasome non-ATPase regulatory subunit 1 OS=Homo sapiens OX=9606 GN=PSMD1 PE=1	15	0,786	1,999	1,559	16013959	11384448,68	9782945,085
A0A8Q3WKT3	RBFOX2	RNA binding protein fox-1 homolog 2 OS=Homo sapiens OX=9606 GN=RBFOX2 PE=1 SV=1	4	0,618	4,369	1,558	4793407,377	2406944,449	1297069,916
P30044	PRDX5	Peroxiredoxin-5, mitochondrial OS=Homo sapiens OX=9606 GN=PRDX5 PE=1 SV=4	8	1,324	1,127	1,557	32199313,45	38533817,54	26506981,07
P61421	ATP6V0D1	V-type proton ATPase subunit d 1 OS=Homo sapiens OX=9606 GN=ATP6V0D1 PE=1 SV=1	5	1,751	0,805	1,556	3281054,93	6504794,86	6786465,361
Q9UBS4	DNAJB11	DnaJ homolog subfamily B member 11 OS=Homo sapiens OX=9606 GN=DNAJB11 PE=1 SV=1	8	1,352	0,973	1,554	15144902,54	20064367,26	13306673,92
O00161	SNAP23	Synaptosomal-associated protein 23 OS=Homo sapiens OX=9606 GN=SNAP23 PE=1 SV=1	5	1,2	1,477	1,554	2690271,878	3578128,486	1145812,906
P55268	LAMB2	Laminin subunit beta-2 OS=Homo sapiens OX=9606 GN=LAMB2 PE=1 SV=2	4	3,158	0,382	1,552	160790,4151	1173655,097	700456,5274
Q9NVA2	SEPTIN11	Septin-11 OS=Homo sapiens OX=9606 GN=SEPTIN11 PE=1 SV=3	7	0,478	3,124	1,551	23670704,18	10139822,31	6310624,416
O14818	PSMA7	Proteasome subunit alpha type-7 OS=Homo sapiens OX=9606 GN=PSMA7 PE=1 SV=1	10	1,229	1,316	1,549	39092426,61	46780844,59	27464585,23
Q8NBJ7	SUMF2	Inactive C-alpha-formylglycine-generating enzyme 2 OS=Homo sapiens OX=9606 GN=SUMF2 PE=	5	2,167	0,712	1,547	2874306,263	6932235,38	3818669,688
Q92734	TFG	Protein TFG OS=Homo sapiens OX=9606 GN=TFG PE=1 SV=2	4	1,458	1,154	1,547	5968378,309	7635995,849	5463591,094
Q96MY1	NOL4L	Nucleolar protein 4-like OS=Homo sapiens OX=9606 GN=NOL4L PE=1 SV=2	2	0,552	2,764	1,547	734755,0148	392351,7534	257571,8492
P50995	ANXA11	Annexin A11 OS=Homo sapiens OX=9606 GN=ANXA11 PE=1 SV=1	10	3,436	0,477	1,545	4374114,577	14034181,32	9510141,459
Q9Y5K6	CD2AP	CD2-associated protein OS=Homo sapiens OX=9606 GN=CD2AP PE=1 SV=1	16	3,435	0,462	1,545	1090873,581	2943322,745	2079677,627
Q9H3P7	ACBD3	Golgi resident protein GCP60 OS=Homo sapiens OX=9606 GN=ACBD3 PE=1 SV=4	7	1,744	0,827	1,543	4167238,283	4284799,737	3339591,447
Q9NYU2	UGGT1	UDP-glucose:glycoprotein glucosyltransferase 1 OS=Homo sapiens OX=9606 GN=UGGT1 PE=1 SV	35	1,312	1,146	1,543	29762371,93	40010391,88	29791534,62
Q15366	PCBP2	Poly(rC)-binding protein 2 OS=Homo sapiens OX=9606 GN=PCBP2 PE=1 SV=1	6	0,504	3,019	1,543	37739616,29	11791635,07	11993131,04
P21399	ACO1	Cytoplasmic aconitate hydratase OS=Homo sapiens OX=9606 GN=ACO1 PE=1 SV=3	7	1,225	1,775	1,542	1435325,879	1416246,299	541610,5232
P09651	HNRNPA1	Heterogeneous nuclear ribonucleoprotein A1 OS=Homo sapiens OX=9606 GN=HNRNPA1 PE=1 SV	12	0,775	2,024	1,542	366870285,7	311176752,2	221963405,4
P49458	SRP9	Signal recognition particle 9 kDa protein OS=Homo sapiens OX=9606 GN=SRP9 PE=1 SV=2	2	0,161	14,494	1,542	10275399,59	1478774,765	1094019,567
Q05D32	CTDSPL2	CTD small phosphatase-like protein 2 OS=Homo sapiens OX=9606 GN=CTDSPL2 PE=1 SV=2	2	0,783	2,28	1,54	718430,1851	159706,0363	295366,3498
Q96QD9	FYTTD1	UAP56-interacting factor OS=Homo sapiens OX=9606 GN=FYTTD1 PE=1 SV=3	2	1,867	0,556	1,538	390200,0552	390637,2187	701427,8086
O94905	ERLIN2	Erlin-2 OS=Homo sapiens OX=9606 GN=ERLIN2 PE=1 SV=1	9	1,735	1,019	1,538	15093619,23	23772739,49	16940658,1
P08579	SNRPB2	U2 small nuclear ribonucleoprotein B'' OS=Homo sapiens OX=9606 GN=SNRPB2 PE=1 SV=1	2	0,868	1,729	1,538	3424764,579	3146192,105	3503335,2
Q7Z2W4	ZC3HAV1	Zinc finger CCCH-type antiviral protein 1 OS=Homo sapiens OX=9606 GN=ZC3HAV1 PE=1 SV=3	11	1,236	1,171	1,537	6484156,941	9963352,486	5296352,632
P68104	EEF1A1	Elongation factor 1-alpha 1 OS=Homo sapiens OX=9606 GN=EEF1A1 PE=1 SV=1	6	0,67	6,263	1,536	228372694,1	137525944,3	89863976,19
Q96IJ6	GMPPA	Mannose-1-phosphate guanyltransferase alpha OS=Homo sapiens OX=9606 GN=GMPPA PE=1 SV	4	1,292	1,55	1,535	1237504,175	1473879,907	837923,4844
P28290	ITPRID2	Protein ITPRID2 OS=Homo sapiens OX=9606 GN=ITPRID2 PE=1 SV=3	3	1,293	1,51	1,534	780234,401	1035429,576	467444,1909
O00410	IPO5	Importin-5 OS=Homo sapiens OX=9606 GN=IPO5 PE=1 SV=4	19	0,514	3,429	1,534	50786377,75	23203628,8	14353058,62
P16435	POR	NADPH--cytochrome P450 reductase OS=Homo sapiens OX=9606 GN=POR PE=1 SV=2	26	1,784	0,909	1,533	18195326,57	31854143,13	23165802,32
Q13177	PAK2	Serine/threonine-protein kinase PAK 2 OS=Homo sapiens OX=9606 GN=PAK2 PE=1 SV=3	8	0,648	2,806	1,53	9606441,591	5449281,04	3484390,372
Q14739	LBR	Delta(14)-sterol reductase LBR OS=Homo sapiens OX=9606 GN=LBR PE=1 SV=2	8	0,722	2,106	1,525	15922213,18	11510795,47	9647134,302
P49915	GMPS	GMP synthase [glutamine-hydrolyzing] OS=Homo sapiens OX=9606 GN=GMPS PE=1 SV=1	18	0,597	2,16	1,523	26227597,2	15327120,82	13234777,99
Q14232	EIF2B1	Translation initiation factor eIF2B subunit alpha OS=Homo sapiens OX=9606 GN=EIF2B1 PE=1 SV	7	0,737	2,929	1,522	4965045,107	2331621,91	1653093,366
P22307	SCP2	Sterol carrier protein 2 OS=Homo sapiens OX=9606 GN=SCP2 PE=1 SV=2	16	2,879	0,632	1,52	12997336,02	34136695,07	20176958,76
O95140	MFN2	Mitofusin-2 OS=Homo sapiens OX=9606 GN=MFN2 PE=1 SV=3	2	1,276	1,155	1,52	688050,395	868593,8107	784920,4965
Q9P016	THYN1	Thymocyte nuclear protein 1 OS=Homo sapiens OX=9606 GN=THYN1 PE=1 SV=1	3	0,697	2,362	1,519	2375102,873	1518489,878	977715,9017
Q9NQC3	RTN4	Reticulon-4 OS=Homo sapiens OX=9606 GN=RTN4 PE=1 SV=2	12	2,4	1,042	1,517	31530815,89	41838285,07	33805526,45
Q7Z7H5	TMED4	Transmembrane emp24 domain-containing protein 4 OS=Homo sapiens OX=9606 GN=TMED4 PE	6	1,175	1,937	1,517	7647184,014	8614141,093	4686178,261
O43660	PLRG1	Pleiotropic regulator 1 OS=Homo sapiens OX=9606 GN=PLRG1 PE=1 SV=1	2	0,372	1,734	1,517	955686,2949	640919,5426	454980,4324
Q6YHK3	CD109	CD109 antigen OS=Homo sapiens OX=9606 GN=CD109 PE=1 SV=2	17	7,208	0,087	1,514	754114,1429	15454281,64	10849376,27
Q15181	PPA1	Inorganic pyrophosphatase OS=Homo sapiens OX=9606 GN=PPA1 PE=1 SV=2	12	0,511	3,092	1,514	32502484,62	15783655,81	9705541,965
B7ZLQ5	SMARCA1	SMARCA1 protein OS=Homo sapiens OX=9606 GN=SMARCA1 PE=1 SV=1	10	0,389	4,033	1,513	20219978,95	17050532,8	5199429,183
O00214	LGALS8	Galectin-8 OS=Homo sapiens OX=9606 GN=LGALS8 PE=1 SV=4	3	2,125	0,549	1,512	146277,6707	1121602,645	726164,7518
Q15645	TRIP13	Pachytene checkpoint protein 2 homolog OS=Homo sapiens OX=9606 GN=TRIP13 PE=1 SV=2	6	0,426	3,34	1,512	8068801,623	3094899,008	2326519,305
Q6P587	FAHD1	Acylpyruvase FAHD1, mitochondrial OS=Homo sapiens OX=9606 GN=FAHD1 PE=1 SV=2	3	2,524	0,6	1,511	1238269,043	2906391,994	3013235,252
Q92917	GPKOW	G-patch domain and KOW motifs-containing protein OS=Homo sapiens OX=9606 GN=GPKOW PE	2	0,783	1,901	1,511	1404637,411	1111537,711	723961,3397
P32322	PYCR1	Pyrroline-5-carboxylate reductase 1, mitochondrial OS=Homo sapiens OX=9606 GN=PYCR1 PE=1	7	1,154	1,144	1,509	4641857,11	7199376,209	4706555,286
P17174	GOT1	Aspartate aminotransferase, cytoplasmic OS=Homo sapiens OX=9606 GN=GOT1 PE=1 SV=3	10	0,493	2,616	1,509	11467479,66	3566147,801	4548787,313
Q8WVV9	HNRNPLL	Heterogeneous nuclear ribonucleoprotein L-like OS=Homo sapiens OX=9606 GN=HNRNPLL PE=1	3	0,485	2,971	1,509	1320042,357	643795,9433	280160,3691
P61769	B2M	Beta-2-microglobulin OS=Homo sapiens OX=9606 GN=B2M PE=1 SV=1	3	20,967	0,081	1,508	9283483,31	170560923,4	100386346,5
P49755	TMED10	Transmembrane emp24 domain-containing protein 10 OS=Homo sapiens OX=9606 GN=TMED10	7	1,406	1,146	1,508	43908250,54	50672058,62	35626602,41
P51665	PSMD7	26S proteasome non-ATPase regulatory subunit 7 OS=Homo sapiens OX=9606 GN=PSMD7 PE=1	7	0,725	1,967	1,508	10033331,38	7010097,626	4845475,883
O75477	ERLIN1	Erlin-1 OS=Homo sapiens OX=9606 GN=ERLIN1 PE=1 SV=2	5	2,672	0,552	1,506	2931073,844	8182213,085	5260791,992
Q15904	ATP6AP1	V-type proton ATPase subunit S1 OS=Homo sapiens OX=9606 GN=ATP6AP1 PE=1 SV=2	5	0,918	1,771	1,506	2609034,96	1760720,328	1408987,846
P15531	NME1	Nucleoside diphosphate kinase A OS=Homo sapiens OX=9606 GN=NME1 PE=1 SV=1	5	0,583	2,152	1,504	115751977,8	74813634,03	63170344,56
P35241	RDX	Radixin OS=Homo sapiens OX=9606 GN=RDX PE=1 SV=1	14	1,211	1,374	1,502	54348019,25	44769948,58	61938852,2
Q5JTZ9	AARS2	Alanine--tRNA ligase, mitochondrial OS=Homo sapiens OX=9606 GN=AARS2 PE=1 SV=1	10	1,118	1,862	1,501	1889475,196	2189848,751	1081740,386
Q6P3W7	SCYL2	SCY1-like protein 2 OS=Homo sapiens OX=9606 GN=SCYL2 PE=1 SV=1	3	1,063	1,614	1,501	959579,4701	841223,1351	633180,6221
P82912	MRPS11	Small ribosomal subunit protein uS11m OS=Homo sapiens OX=9606 GN=MRPS11 PE=1 SV=2	3	2,443	0,653	1,5	1169800,86	3554626,465	3330715,005
Q03518	TAP1	Antigen peptide transporter 1 OS=Homo sapiens OX=9606 GN=TAP1 PE=1 SV=3	11	28,515	0,102	1,497	198255,005	29321677,62	17907787,86
Q96H79	ZC3HAV1L	Zinc finger CCCH-type antiviral protein 1-like OS=Homo sapiens OX=9606 GN=ZC3HAV1L PE=1 SV	2	0,181	8,332	1,496	1648041,291	282385,1038	182493,3208
Q5BJH7	YIF1B	Protein YIF1B OS=Homo sapiens OX=9606 GN=YIF1B PE=1 SV=1	2	2,067	0,695	1,495	833399,0084	1722354,475	1608996,579
Q8TAA3	PSMA8	Proteasome subunit alpha-type 8 OS=Homo sapiens OX=9606 GN=PSMA8 PE=2 SV=3	4	1,064	1,519	1,495	17987934,34	19528581,89	10809978,65
P61803	DAD1	Dolichyl-diphosphooligosaccharide--protein glycosyltransferase subunit DAD1 OS=Homo sapiens	4	1,36	1,226	1,489	18132177,26	23897155,14	14801819,48
Q14141	SEPTIN6	Septin-6 OS=Homo sapiens OX=9606 GN=SEPTIN6 PE=1 SV=4	7	0,364	5,865	1,486	14446789,44	3856982,692	2687450,169
O76003	GLRX3	Glutaredoxin-3 OS=Homo sapiens OX=9606 GN=GLRX3 PE=1 SV=2	11	0,624	2,937	1,485	17386675,68	26154435,78	6661013,569
Q9H3M7	TXNIP	Thioredoxin-interacting protein OS=Homo sapiens OX=9606 GN=TXNIP PE=1 SV=1	2	24,334	0,076	1,484	30535,55464	1429221,086	872280,1396
O75915	ARL6IP5	PRA1 family protein 3 OS=Homo sapiens OX=9606 GN=ARL6IP5 PE=1 SV=1	5	2,396	0,668	1,483	3383895,508	5906217,61	4594319,482
Q8WXG1	RSAD2	S-adenosylmethionine-dependent nucleotide dehydratase RSAD2 OS=Homo sapiens OX=9606 G	8	2,275	0,43	1,483	155580,5519	1281473,953	596480,4484
P23258	TUBG1	Tubulin gamma-1 chain OS=Homo sapiens OX=9606 GN=TUBG1 PE=1 SV=2	3	0,846	1,716	1,483	2406801,347	2011654,287	1640236,015
Q10589	BST2	Bone marrow stromal antigen 2 OS=Homo sapiens OX=9606 GN=BST2 PE=1 SV=1	4	14,448	0,078	1,482	1516459,366	32678856,22	24937394,58
Q8NBU5	ATAD1	Outer mitochondrial transmembrane helix translocase OS=Homo sapiens OX=9606 GN=ATAD1 P	3	1,487	1,549	1,482	1723939,228	2821487,843	1649398,75
Q9NZW5	PALS2	Protein PALS2 OS=Homo sapiens OX=9606 GN=PALS2 PE=1 SV=2	6	1,187	1,145	1,482	3360226,617	4176141,473	2947575,844
Q9UI15	TAGLN3	Transgelin-3 OS=Homo sapiens OX=9606 GN=TAGLN3 PE=1 SV=2	2	1,391	0,954	1,481	644353,6387	895994,0144	715277,4832
P63261	ACTG1	Actin, cytoplasmic 2 OS=Homo sapiens OX=9606 GN=ACTG1 PE=1 SV=1	2	1,733	0,86	1,48	2979428140	5260138922	4063382240
Q6P1N9	TATDN1	Deoxyribonuclease TATDN1 OS=Homo sapiens OX=9606 GN=TATDN1 PE=1 SV=2	2	0,904	1,334	1,48	755536,0525	572457,551	485195,553
O60568	PLOD3	Multifunctional procollagen lysine hydroxylase and glycosyltransferase LH3 OS=Homo sapiens O	11	2,196	0,555	1,478	3349061,368	10783515,05	6430559,044
Q9Y282	ERGIC3	Endoplasmic reticulum-Golgi intermediate compartment protein 3 OS=Homo sapiens OX=9606 G	3	1,906	0,781	1,478	1100676,39	2139182,036	1461507,755
Q14019	COTL1	Coactosin-like protein OS=Homo sapiens OX=9606 GN=COTL1 PE=1 SV=3	7	1,284	1,255	1,477	11188950,13	11389672,39	8789054,702
Q9NY33	DPP3	Dipeptidyl peptidase 3 OS=Homo sapiens OX=9606 GN=DPP3 PE=1 SV=2	11	0,671	2,092	1,476	6699676,895	3770938,005	2565267,831
Q9UK41	VPS28	Vacuolar protein sorting-associated protein 28 homolog OS=Homo sapiens OX=9606 GN=VPS28	2	2,5	0,9	1,475	857277,3139	1541477,73	971680,3838
Q8NFW8	CMAS	N-acylneuraminate cytidylyltransferase OS=Homo sapiens OX=9606 GN=CMAS PE=1 SV=2	3	0,954	1,292	1,473	1125784,719	1151368,843	752211,5527
Q9NUJ1	ABHD10	Palmitoyl-protein thioesterase ABHD10, mitochondrial OS=Homo sapiens OX=9606 GN=ABHD10	6	1,458	0,978	1,472	2796392,866	4149272,662	2957760,935
Q9UKY7	CDV3	Protein CDV3 homolog OS=Homo sapiens OX=9606 GN=CDV3 PE=1 SV=1	6	0,797	1,808	1,472	8565272,807	6762500,145	4896511,885
Q8WW12	PCNP	PEST proteolytic signal-containing nuclear protein OS=Homo sapiens OX=9606 GN=PCNP PE=1 SV	5	0,581	2,523	1,471	8411515,219	5079354,935	3284207,872
Q9UHY1	NRBP1	Nuclear receptor-binding protein OS=Homo sapiens OX=9606 GN=NRBP1 PE=1 SV=1	2	1,211	1,113	1,47	1439958,392	1773362,923	2066046,38
Q13510	ASAH1	Acid ceramidase OS=Homo sapiens OX=9606 GN=ASAH1 PE=1 SV=5	7	2,454	0,613	1,466	1293331,662	3376845,305	1839783,186
O14976	GAK	Cyclin-G-associated kinase OS=Homo sapiens OX=9606 GN=GAK PE=1 SV=2	2	1,911	0,766	1,465	308533,0731	521331,9643	355931,5256
Q96AB3	ISOC2	Isochorismatase domain-containing protein 2 OS=Homo sapiens OX=9606 GN=ISOC2 PE=1 SV=1	4	3,837	0,371	1,463	301368,7192	1130320,259	857535,0582
O60493	SNX3	Sorting nexin-3 OS=Homo sapiens OX=9606 GN=SNX3 PE=1 SV=3	4	1,378	1,022	1,461	3712108,405	5287792,01	3733730,606
Q9UHB6	LIMA1	LIM domain and actin-binding protein 1 OS=Homo sapiens OX=9606 GN=LIMA1 PE=1 SV=1	27	5,248	0,374	1,46	8448702,841	46545829,63	25209191,64
P61019	RAB2A	Ras-related protein Rab-2A OS=Homo sapiens OX=9606 GN=RAB2A PE=1 SV=1	4	1,462	1,005	1,459	24504598,13	32511599,21	25400813,42
Q9HBL7	PLGRKT	Plasminogen receptor (KT) OS=Homo sapiens OX=9606 GN=PLGRKT PE=1 SV=1	2	2,22	0,567	1,458	890512,6828	2250029,062	1538891,524
Q13011	ECH1	Delta(3,5)-Delta(2,4)-dienoyl-CoA isomerase, mitochondrial OS=Homo sapiens OX=9606 GN=ECH	9	1,867	0,831	1,458	7600619,243	14128640,55	10860348,62
O15126	SCAMP1	Secretory carrier-associated membrane protein 1 OS=Homo sapiens OX=9606 GN=SCAMP1 PE=1	5	1,439	1,2	1,458	3649085,143	5472051,392	3538502,163
Q8IYM9	TRIM22	E3 ubiquitin-protein ligase TRIM22 OS=Homo sapiens OX=9606 GN=TRIM22 PE=1 SV=1	4	3,337	0,291	1,456	171090,3021	1997547,778	1894394,915
Q9BX68	HINT2	Adenosine 5'-monophosphoramidase HINT2 OS=Homo sapiens OX=9606 GN=HINT2 PE=1 SV=1	3	2,947	0,489	1,456	4148504,404	11543166,44	7861938,124
Q99426	TBCB	Tubulin-folding cofactor B OS=Homo sapiens OX=9606 GN=TBCB PE=1 SV=2	3	0,476	1,962	1,455	6548854,374	3120351,904	1470343,553
P61088	UBE2N	Ubiquitin-conjugating enzyme E2 N OS=Homo sapiens OX=9606 GN=UBE2N PE=1 SV=1	7	0,538	2,748	1,453	42652221,34	22412475,5	15750778,02
P55285	CDH6	Cadherin-6 OS=Homo sapiens OX=9606 GN=CDH6 PE=1 SV=1	5	0,216	5,604	1,453	4443431,185	323351,4348	316723,9596
Q49AR2	C5orf22	UPF0489 protein C5orf22 OS=Homo sapiens OX=9606 GN=C5orf22 PE=1 SV=2	3	0,086	18,263	1,451	1484421,117	80831,24286	28466,54308
P00568	AK1	Adenylate kinase isoenzyme 1 OS=Homo sapiens OX=9606 GN=AK1 PE=1 SV=3	5	1,699	0,805	1,449	3596137,244	5796539,453	4612924,711
P41212	ETV6	Transcription factor ETV6 OS=Homo sapiens OX=9606 GN=ETV6 PE=1 SV=1	7	1,662	0,87	1,449	245316,2271	614822,5776	383479,0395

P20339	RAB5A	Ras-related protein Rab-5A OS=Homo sapiens OX=9606 GN=RAB5A PE=1 SV=2	3	1,267	0,85	1,449	3468290,466	4556312,914	3916897,058
Q53TN4	CYBRD1	Plasma membrane ascorbate-dependent reductase CYBRD1 OS=Homo sapiens OX=9606 GN=CYE	2	6,401		1,448	79112,06913	1731880,331	833476,8694
P37802	TAGLN2	Transgelin-2 OS=Homo sapiens OX=9606 GN=TAGLN2 PE=1 SV=3	9	3,127	0,553	1,447	43094477,63	118856300,8	85557962,2
Q9BT22	ALG1	Chitobiosyldiphosphodolichol beta-mannosyltransferase OS=Homo sapiens OX=9606 GN=ALG1 F	3	2,987	0,466	1,447	728469,4927	1585479,597	1683651,274
Q8NEZ5	FBXO22	F-box only protein 22 OS=Homo sapiens OX=9606 GN=FBXO22 PE=1 SV=1	2		3,017	1,446	364672,8303	244481,1802	277374,286
Q99436	PSMB7	Proteasome subunit beta type-7 OS=Homo sapiens OX=9606 GN=PSMB7 PE=1 SV=1	6	0,767	1,683	1,446	9492698,087	8393186,849	7013100,946
O75348	ATP6V1G1	V-type proton ATPase subunit G 1 OS=Homo sapiens OX=9606 GN=ATP6V1G1 PE=1 SV=3	2	0,886	1,617	1,445	2019308,924	1805029,568	1249053,117
Q9Y547	IFT25	Intraflagellar transport protein 25 homolog OS=Homo sapiens OX=9606 GN=IFT25 PE=1 SV=1	3	0,376	3,006	1,443	4272250,188	1807219,242	1218381,243
Q9HC07	TMEM165	Putative divalent cation/proton antiporter TMEM165 OS=Homo sapiens OX=9606 GN=TMEM165	5	3,722	0,369	1,442	1841034,827	7226836,857	5020877,282
P60842	EIF4A1	Eukaryotic initiation factor 4A-I OS=Homo sapiens OX=9606 GN=EIF4A1 PE=1 SV=1	12	0,495	2,812	1,438	169841859,5	82951321,64	73763109,15
P29323	EPHB2	Ephrin type-B receptor 2 OS=Homo sapiens OX=9606 GN=EPHB2 PE=1 SV=5	5	4,006	0,2	1,436	265128,9939	3702160,72	2993467,53
Q06210	GFPT1	Glutamine--fructose-6-phosphate aminotransferase [isomerizing] 1 OS=Homo sapiens OX=9606	10	0,544	2,744	1,435	8732615,501	4051580,413	2936694,787
P48637	GSS	Glutathione synthetase OS=Homo sapiens OX=9606 GN=GSS PE=1 SV=1	8	1,011	1,38	1,434	4381874,641	4088241,657	5849917,954
O14617	AP3D1	AP-3 complex subunit delta-1 OS=Homo sapiens OX=9606 GN=AP3D1 PE=1 SV=1	6	0,624	2,19	1,434	1760132,908	824291,8159	346668,6982
Q15643	TRIP11	Thyroid receptor-interacting protein 11 OS=Homo sapiens OX=9606 GN=TRIP11 PE=1 SV=3	4		0,468	1,431	153842,2308	433226,1334	838652,4631
P05121	SERPINE1	Plasminogen activator inhibitor 1 OS=Homo sapiens OX=9606 GN=SERPINE1 PE=1 SV=1	2	1000	0,001	1,431		174638,27	485084,9789
Q8NCW5	NAXE	NAD(P)H-hydrate epimerase OS=Homo sapiens OX=9606 GN=NAXE PE=1 SV=2	4	1,294	1,397	1,427	2153299,297	1678464,842	1845395,141
P62256	UBE2H	Ubiquitin-conjugating enzyme E2 H OS=Homo sapiens OX=9606 GN=UBE2H PE=1 SV=1	3	0,755	1,94	1,423	1107946,499	785306,6004	638647,4546
P11216	PYGB	Glycogen phosphorylase, brain form OS=Homo sapiens OX=9606 GN=PYGB PE=1 SV=5	12	1,064	1,497	1,422	16320868,25	15364991,94	10180933,96
Q03519	TAP2	Antigen peptide transporter 2 OS=Homo sapiens OX=9606 GN=TAP2 PE=1 SV=1	10	16,388	0,073	1,42	1163550,669	22870405,06	11144874,26
P62195	PSMC5	26S proteasome regulatory subunit 8 OS=Homo sapiens OX=9606 GN=PSMC5 PE=1 SV=1	13	0,883	1,594	1,42	24119682,1	20601851,8	14140537,59
P62136	PPP1CA	Serine/threonine-protein phosphatase PP1-alpha catalytic subunit OS=Homo sapiens OX=9606 G	3	0,976	1,488	1,419	20532406,24	16854696,61	10929046,87
Q96FV9	THOC1	THO complex subunit 1 OS=Homo sapiens OX=9606 GN=THOC1 PE=1 SV=1	4	0,792	2,218	1,418	6069459,994	1447969,636	1129441,427
Q9Y606	PUS1	Pseudouridylate synthase 1 homolog OS=Homo sapiens OX=9606 GN=PUS1 PE=1 SV=3	7	0,741	1,629	1,418	2861845,388	1556122,175	1501417,263
P62937	PPIA	Peptidyl-prolyl cis-trans isomerase A OS=Homo sapiens OX=9606 GN=PPIA PE=1 SV=2	8	0,558	2,222	1,418	302597542,3	169833609,9	138544752,2
Q9Y3B3	TMED7	Transmembrane emp24 domain-containing protein 7 OS=Homo sapiens OX=9606 GN=TMED7 PE	4	0,949	1,484	1,417	8553254,955	13084994,04	6093300,162
P13591	NCAM1	Neural cell adhesion molecule 1 OS=Homo sapiens OX=9606 GN=NCAM1 PE=1 SV=3	7	0,157	4,503	1,417	4492993,052	265042,0383	547692,3051
Q96IW7	SEC22A	Vesicle-trafficking protein SEC22a OS=Homo sapiens OX=9606 GN=SEC22A PE=1 SV=1	2	1000	0,001	1,416		416916,8823	322600,7297
Q12907	LMAN2	Vesicular integral-membrane protein VIP36 OS=Homo sapiens OX=9606 GN=LMAN2 PE=1 SV=1	12	1,637	0,888	1,416	24037450,14	34745284,05	26715996,13
Q9NPA0	EMC7	ER membrane protein complex subunit 7 OS=Homo sapiens OX=9606 GN=EMC7 PE=1 SV=1	4	1,855	0,879	1,412	2444140,322	4791075,069	3185990,203
P13489	RNH1	Ribonuclease inhibitor OS=Homo sapiens OX=9606 GN=RNH1 PE=1 SV=2	3	1,271	1,095	1,409	5066507,033	6459671,374	4551814,805
Q86XZ4	SPATS2	Spermatogenesis-associated serine-rich protein 2 OS=Homo sapiens OX=9606 GN=SPATS2 PE=1	2	0,676	2,278	1,409	763456,1148	425381,8981	202072,7342
Q9NP72	RAB18	Ras-related protein Rab-18 OS=Homo sapiens OX=9606 GN=RAB18 PE=1 SV=1	6	2,141	0,701	1,407	4824069,742	10317269,99	7361427,744
Q15404	RSU1	Ras suppressor protein 1 OS=Homo sapiens OX=9606 GN=RSU1 PE=1 SV=3	4	2,072	0,7	1,406	1265069,999	2370616,097	1618503,453
O75695	RP2	Protein XRP2 OS=Homo sapiens OX=9606 GN=RP2 PE=1 SV=4	3	1,941	0,891	1,406	1412877,078	1756228,017	1747929,868
Q02218	OGDH	2-oxoglutarate dehydrogenase complex component E1 OS=Homo sapiens OX=9606 GN=OGDH P	30	0,915	1,674	1,406	41305100,69	33320816,12	27191226,28
P46940	IQGAP1	Ras GTPase-activating-like protein IQGAP1 OS=Homo sapiens OX=9606 GN=IQGAP1 PE=1 SV=1	61	4,945	0,288	1,405	18607553,86	96132518,63	71353105,34
O95786	RIGI	Antiviral innate immune response receptor RIG-I OS=Homo sapiens OX=9606 GN=RIGI PE=1 SV=	5	3,222	0,427	1,404	622503,7171	3805012,699	1611831,836
Q9NV96	TMEM30A	Cell cycle control protein 50A OS=Homo sapiens OX=9606 GN=TMEM30A PE=1 SV=1	2	1,46	1,067	1,403	546440,6324	781721,5866	580816,6925
Q96HQ2	CDKN2AIPNL	CDKN2AIP N-terminal-like protein OS=Homo sapiens OX=9606 GN=CDKN2AIPNL PE=1 SV=1	3	0,623	3,736	1,403	777940,3995	972254,1855	1724193,442
Q13409	DYNC1I2	Cytoplasmic dynein 1 intermediate chain 2 OS=Homo sapiens OX=9606 GN=DYNC1I2 PE=1 SV=3	8	0,604	2,689	1,403	9006383,056	5080132,797	3510063,066
P18031	PTPN1	Tyrosine-protein phosphatase non-receptor type 1 OS=Homo sapiens OX=9606 GN=PTPN1 PE=1	11	1,541	0,844	1,402	10816762,91	16417373,98	14243885,42
Q15121	PEA15	Astrocytic phosphoprotein PEA-15 OS=Homo sapiens OX=9606 GN=PEA15 PE=1 SV=2	4	0,635	2,187	1,4	6401225,005	3578557,212	2790200,464
P52926	HMGA2	High mobility group protein HMGI-C OS=Homo sapiens OX=9606 GN=HMGA2 PE=1 SV=1	5	0,019	68,759	1,398	30710387,85	212027,8041	152651,2583
O14964	HGS	Hepatocyte growth factor-regulated tyrosine kinase substrate OS=Homo sapiens OX=9606 GN=H	5	1,428	0,933	1,397	1678259,206	3569636,504	2031007,445
P62888	RPL30	Large ribosomal subunit protein eL30 OS=Homo sapiens OX=9606 GN=RPL30 PE=1 SV=2	4	1,043	1,413	1,397	10244388,4	10622388,23	6455070,747
O00232	PSMD12	26S proteasome non-ATPase regulatory subunit 12 OS=Homo sapiens OX=9606 GN=PSMD12 PE=	9	0,85	1,416	1,397	7725334,322	6626868,105	5913725,159
Q13885	TUBB2A	Tubulin beta-2A chain OS=Homo sapiens OX=9606 GN=TUBB2A PE=1 SV=1	4	0,305	6,412	1,397	152273653,1	59682424,86	13142700,22
P16930	FAH	Fumarylacetoacetase OS=Homo sapiens OX=9606 GN=FAH PE=1 SV=2	2	2,268	0,616	1,396	228862,958	508597,6323	370302,8602
P53992	SEC24C	Protein transport protein Sec24C OS=Homo sapiens OX=9606 GN=SEC24C PE=1 SV=3	12	1,065	1,236	1,396	9255178,574	9083199,753	7203608,871
O95456	PSMG1	Proteasome assembly chaperone 1 OS=Homo sapiens OX=9606 GN=PSMG1 PE=1 SV=1	2	0,924	1,411	1,396	884504,2115	817295,8023	585364,2511
Q9Y6C9	MTCH2	Mitochondrial carrier homolog 2 OS=Homo sapiens OX=9606 GN=MTCH2 PE=1 SV=1	7	1,687	0,799	1,395	9337054,673	14476369,21	11799464,52
P82909	KGD4	Alpha-ketoglutarate dehydrogenase component 4 OS=Homo sapiens OX=9606 GN=KGD4 PE=1 S'	3	0,976	1,278	1,395	2970560,552	2893071,87	1825426,858
O60306	AQR	RNA helicase aquarius OS=Homo sapiens OX=9606 GN=AQR PE=1 SV=4	5	0,893	1,399	1,395	2291186,02	2020727,907	1681403,859
P30153	PPP2R1A	Serine/threonine-protein phosphatase 2A 65 kDa regulatory subunit A alpha isoform OS=Homo s	16	0,725	1,954	1,395	46206548,17	31039521,82	24475311,83
P60866	RPS20	Small ribosomal subunit protein uS10 OS=Homo sapiens OX=9606 GN=RPS20 PE=1 SV=1	3	0,641	1,788	1,394	52959105,01	25168619,03	33371385,24
P98179	RBM3	RNA-binding protein 3 OS=Homo sapiens OX=9606 GN=RBM3 PE=1 SV=1	3	0,606	1,806	1,394	11695518,08	6724882,895	4883293,612
O95881	TXNDC12	Thioredoxin domain-containing protein 12 OS=Homo sapiens OX=9606 GN=TXNDC12 PE=1 SV=1	6	1,076	1,756	1,392	7502269,354	5017041,889	4530576,302
P19256	CD58	Lymphocyte function-associated antigen 3 OS=Homo sapiens OX=9606 GN=CD58 PE=1 SV=1	2	4,859	0,203	1,39	308352,5005	1993774,231	1802751,815
Q96PC5	MIA2	Melanoma inhibitory activity protein 2 OS=Homo sapiens OX=9606 GN=MIA2 PE=1 SV=4	5	2,225	0,953	1,39	443301,2526	1063194,356	1290616,431
Q96L92	SNX27	Sorting nexin-27 OS=Homo sapiens OX=9606 GN=SNX27 PE=1 SV=2	2	1,572	2,041	1,39	1815954,34	176608,8602	846470,3226
O14949	UQCRQ	Cytochrome b-c1 complex subunit 8 OS=Homo sapiens OX=9606 GN=UQCRQ PE=1 SV=4	3	1,238	0,606	1,39	2228141,63	3282253,035	3629793,84
P35606	COPB2	Coatomer subunit beta' OS=Homo sapiens OX=9606 GN=COPB2 PE=1 SV=2	21	1,003	1,368	1,39	21578728,41	19272786,3	16309785,55
O00592	PODXL	Podocalyxin OS=Homo sapiens OX=9606 GN=PODXL PE=1 SV=2	6	5,203	0,212	1,389	2794981,569	7694068,768	12405023,66
P16949	STMN1	Stathmin OS=Homo sapiens OX=9606 GN=STMN1 PE=1 SV=3	6	0,287	4,946	1,388	107796960,6	31267457,42	20703041,18
Q8NBJ5	COLGALT1	Procollagen galactosyltransferase 1 OS=Homo sapiens OX=9606 GN=COLGALT1 PE=1 SV=1	14	3,861	0,337	1,387	8482769,429	32501189,02	28757150,13
O00443	PIK3C2A	Phosphatidylinositol 4-phosphate 3-kinase C2 domain-containing subunit alpha OS=Homo sapier	2	0,554	2,522	1,384	516133,3576	280728,1929	193756,7638
Q9Y6D9	MAD1L1	Mitotic spindle assembly checkpoint protein MAD1 OS=Homo sapiens OX=9606 GN=MAD1L1 PE	4	1,161	1,048	1,383	939447,9257	5685937,123	3378285,988
Q9H857	NT5DC2	5'-nucleotidase domain-containing protein 2 OS=Homo sapiens OX=9606 GN=NT5DC2 PE=1 SV=1	10	0,184	5,426	1,379	7337464,705	1185025,036	1253765,304
Q9NX62	BPNT2	Golgi-resident adenosine 3',5'-bisphosphate 3'-phosphatase OS=Homo sapiens OX=9606 GN=BPI	3	1,848	0,727	1,378	1602401,019	2736519,385	2214696,866
Q9BXK5	BCL2L13	Bcl-2-like protein 13 OS=Homo sapiens OX=9606 GN=BCL2L13 PE=1 SV=1	7	6,421	0,207	1,377	618836,4391	7293658,55	4733564,18
P62191	PSMC1	26S proteasome regulatory subunit 4 OS=Homo sapiens OX=9606 GN=PSMC1 PE=1 SV=1	13	0,779	1,719	1,377	19121677,79	13512685,11	12840558,48
P49841	GSK3B	Glycogen synthase kinase-3 beta OS=Homo sapiens OX=9606 GN=GSK3B PE=1 SV=2	4	0,625	2,447	1,374	1469127,178	882402,8336	637014,1516
Q9UBF2	COPG2	Coatomer subunit gamma-2 OS=Homo sapiens OX=9606 GN=COPG2 PE=1 SV=1	11	0,596	2,32	1,374	8363348,974	4786599,624	4360127,325
Q8IWA5	SLC44A2	Choline transporter-like protein 2 OS=Homo sapiens OX=9606 GN=SLC44A2 PE=1 SV=3	5	9,129	0,167	1,373	304622,8777	3220208,195	2644090,63
Q9HD45	TM9SF3	Transmembrane 9 superfamily member 3 OS=Homo sapiens OX=9606 GN=TM9SF3 PE=1 SV=2	7	2,003	0,598	1,373	2838314,175	6982738,12	4898652,691
Q96B49	TOMM6	Mitochondrial import receptor subunit TOM6 homolog OS=Homo sapiens OX=9606 GN=TOMM6	2	1,606	0,855	1,373	1478124,102	2373553,194	1728688,554
Q9UHQ4	BCAP29	B-cell receptor-associated protein 29 OS=Homo sapiens OX=9606 GN=BCAP29 PE=1 SV=2	3	1,522	0,889	1,373	574811,3416	1417844,034	535454,839
P46459	NSF	Vesicle-fusing ATPase OS=Homo sapiens OX=9606 GN=NSF PE=1 SV=3	18	1,637	0,924	1,372	10220388,98	17479953,96	13492909,6
Q9UK76	JPT1	Jupiter microtubule associated homolog 1 OS=Homo sapiens OX=9606 GN=JPT1 PE=1 SV=3	7	0,653	2,826	1,369	18039142,66	10650814,55	7918021,604
Q9Y315	DERA	Deoxyribose-phosphate aldolase OS=Homo sapiens OX=9606 GN=DERA PE=1 SV=2	2	0,927	1,562	1,368	320841,047	267072,3783	188624,574
P52888	THOP1	Thimet oligopeptidase OS=Homo sapiens OX=9606 GN=THOP1 PE=1 SV=2	2	0,479	1,51	1,368	1075076,244	845738,4022	699798,6994
Q96IU4	ABHD14B	Putative protein-lysine deacylase ABHD14B OS=Homo sapiens OX=9606 GN=ABHD14B PE=1 SV=	4	3,248	0,339	1,366	48080,32187	1446271,339	714367,3146
Q99962	SH3GL2	Endophilin-A1 OS=Homo sapiens OX=9606 GN=SH3GL2 PE=1 SV=1	2	0,77	1,722	1,364	2047046,598	1899825,714	1344187,571
P00403	MT-CO2	Cytochrome c oxidase subunit 2 OS=Homo sapiens OX=9606 GN=MT-CO2 PE=1 SV=1	4	1,313	0,738	1,363	24195366,84	33650156,59	34130149,23
P14324	FDPS	Farnesyl pyrophosphate synthase OS=Homo sapiens OX=9606 GN=FDPS PE=1 SV=4	6	0,276	4,836	1,363	23981891,45	6406911,459	5125193,432
A0A5H1ZRP4	NELFB	Negative elongation factor B OS=Homo sapiens OX=9606 GN=NELFB PE=1 SV=1	4	0,727	1,558	1,362	4276442,098	4994286,692	3741939,701
P16152	CBR1	Carbonyl reductase [NADPH] 1 OS=Homo sapiens OX=9606 GN=CBR1 PE=1 SV=3	11	0,71	1,925	1,362	21754370,3	14789747,52	11292065,7
Q9Y5Z4	HEBP2	Heme-binding protein 2 OS=Homo sapiens OX=9606 GN=HEBP2 PE=1 SV=1	5	0,918	1,529	1,361	1243181,739	1134950,957	551800,6367
Q16864	ATP6V1F	V-type proton ATPase subunit F OS=Homo sapiens OX=9606 GN=ATP6V1F PE=1 SV=2	2	0,78	1,552	1,36	2542215,947	1823789,292	1099832,584
Q9GZS3	SKIC8	Superkiller complex protein 8 OS=Homo sapiens OX=9606 GN=SKIC8 PE=1 SV=1	6	1,247	1,031	1,359	2912009,407	2157694,744	2209070,765
Q7Z6Z7	HUWE1	E3 ubiquitin-protein ligase HUWE1 OS=Homo sapiens OX=9606 GN=HUWE1 PE=1 SV=3	24	0,508	2,608	1,359	12235154,57	4858354,356	4655159,646
P53985	SLC16A1	Monocarboxylate transporter 1 OS=Homo sapiens OX=9606 GN=SLC16A1 PE=1 SV=3	5	0,653	1,655	1,358	13704891,4	9546909,863	4531220,338
Q8N128	FAM177A1	Protein FAM177A1 OS=Homo sapiens OX=9606 GN=FAM177A1 PE=1 SV=1	2	2,412	0,592	1,356	265272,3886	973400,6082	719115,4246
P26358	DNMT1	DNA (cytosine-5)-methyltransferase 1 OS=Homo sapiens OX=9606 GN=DNMT1 PE=1 SV=2	7	0,348	2,379	1,354	3143898,719	722252,9475	924434,7243
P05165	PCCA	Propionyl-CoA carboxylase alpha chain, mitochondrial OS=Homo sapiens OX=9606 GN=PCCA PE=	13	0,628	1,754	1,353	4447798,519	2654283,769	1166372,069
P55036	PSMD4	26S proteasome non-ATPase regulatory subunit 4 OS=Homo sapiens OX=9606 GN=PSMD4 PE=1	8	0,815	1,67	1,352	12599694,8	11280862,81	7558492,884
Q7L5D6	GET4	Golgi to ER traffic protein 4 homolog OS=Homo sapiens OX=9606 GN=GET4 PE=1 SV=1	3	0,745	2,665	1,352	1794599,185	785780,0383	833022,1846
Q9Y5K5	UCHL5	Ubiquitin carboxyl-terminal hydrolase isozyme L5 OS=Homo sapiens OX=9606 GN=UCHL5 PE=1 S	5	1,135	1,16	1,351	2751061,079	2662431,375	2152089,066
Q8TD30	GPT2	Alanine aminotransferase 2 OS=Homo sapiens OX=9606 GN=GPT2 PE=1 SV=1	5	1,083	1,531	1,35	1666751,391	1628412,964	764157,243
P30405	PPIF	Peptidyl-prolyl cis-trans isomerase F, mitochondrial OS=Homo sapiens OX=9606 GN=PPIF PE=1 S	3	1,067	0,518	1,348	3806235,519	5427971,926	5213405,314
Q96QK1	VPS35	Vacuolar protein sorting-associated protein 35 OS=Homo sapiens OX=9606 GN=VPS35 PE=1 SV=	16	0,779	2,06	1,348	20768927,41	13581696,4	11918230,63
Q9BXP2	SLC12A9	Solute carrier family 12 member 9 OS=Homo sapiens OX=9606 GN=SLC12A9 PE=1 SV=1	3	4,026	0,329	1,347	290571,5572	1080988,921	847958,0627
Q01813	PFKP	ATP-dependent 6-phosphofructokinase, platelet type OS=Homo sapiens OX=9606 GN=PFKP PE=	13	3,97	0,295	1,345	6107892,754	23852723,86	22516059,09
O60762	DPM1	Dolichol-phosphate mannosyltransferase subunit 1 OS=Homo sapiens OX=9606 GN=DPM1 PE=1	7	1,303	1,137	1,345	5110495,383	10427594,39	8290196,158
O15347	HMGB3	High mobility group protein B3 OS=Homo sapiens OX=9606 GN=HMGB3 PE=1 SV=4	7	0,254	5,672	1,344	41244630,2	10154357,51	7613404,975
Q12904	AIMP1	Aminoacyl tRNA synthase complex-interacting multifunctional protein 1 OS=Homo sapiens OX=9	6	1,095	1,137	1,343	7329154,122	8587574,375	6911866,412
P62070	RRAS2	Ras-related protein R-Ras2 OS=Homo sapiens OX=9606 GN=RRAS2 PE=1 SV=1	2	4,205	0,353	1,342	1659851,102	8467336,042	6805688,056
P60520	GABARAPL2	Gamma-aminobutyric acid receptor-associated protein-like 2 OS=Homo sapiens OX=9606 GN=G/	3	0,37	3,192	1,341	2256797,992	112044,4993	1084828,97
Q15370	ELOB	Elongin-B OS=Homo sapiens OX=9606 GN=ELOB PE=1 SV=1	4	1,096	1,227	1,34	7437576,442	8409324,309	6154070,677
Q9P253	VPS18	Vacuolar protein sorting-associated protein 18 homolog OS=Homo sapiens OX=9606 GN=VPS18	8	1,376	0,702	1,339	918495,9914	1264621,697	1245106,506

Q9H2D6	TRIOBP	TRIO and F-actin-binding protein OS=Homo sapiens OX=9606 GN=TRIOBP PE=1 SV=3	2	1,236	1,018	1,339	700720,0232	920439,2035	698078,6445
Q9UHD8	SEPTIN9	Septin-9 OS=Homo sapiens OX=9606 GN=SEPTIN9 PE=1 SV=2	18	0,843	1,754	1,338	38190505,13	31289915,27	25021539,29
Q13576	IQGAP2	Ras GTPase-activating-like protein IQGAP2 OS=Homo sapiens OX=9606 GN=IQGAP2 PE=1 SV=4	15	0,51	2,883	1,338	9479859,536	1174007,919	855594,0079
O00629	KPNA4	Importin subunit alpha-3 OS=Homo sapiens OX=9606 GN=KPNA4 PE=1 SV=1	3	0,902	1,352	1,336	2778246,804	2363094,427	1858918,237
P11021	HSPA5	Endoplasmic reticulum chaperone BiP OS=Homo sapiens OX=9606 GN=HSPA5 PE=1 SV=2	34	1,561	0,952	1,335	757861218,6	1031921737	843618526,8
P51148	RAB5C	Ras-related protein Rab-5C OS=Homo sapiens OX=9606 GN=RAB5C PE=1 SV=2	5	1,618	0,859	1,334	19924967,79	34414194,84	26055154,95
P17568	NDUFB7	NADH dehydrogenase [ubiquinone] 1 beta subcomplex subunit 7 OS=Homo sapiens OX=9606 GN	2	2,205	0,574	1,331	252725,5775	553134,9118	404933,5816
O75886	STAM2	Signal transducing adapter molecule 2 OS=Homo sapiens OX=9606 GN=STAM2 PE=1 SV=1	2	1,138	1,053	1,329	374621,8236	447320,1663	334563,4671
Q9Y3F4	STRAP	Serine-threonine kinase receptor-associated protein OS=Homo sapiens OX=9606 GN=STRAP PE=	12	0,399	3,063	1,329	26137047,04	9948574,251	7437551,045
O00231	PSMD11	26S proteasome non-ATPase regulatory subunit 11 OS=Homo sapiens OX=9606 GN=PSMD11 PE=	17	0,737	1,635	1,328	35008755,37	23255596,09	18229717,87
Q13404	UBE2V1	Ubiquitin-conjugating enzyme E2 variant 1 OS=Homo sapiens OX=9606 GN=UBE2V1 PE=1 SV=2	6	0,563	2,363	1,328	18639029	7441052,978	7172226,791
Q96SQ9	CYP2S1	Cytochrome P450 2S1 OS=Homo sapiens OX=9606 GN=CYP2S1 PE=1 SV=2	6	2,191	0,792	1,327	266534,7259	516817,4051	273712,2119
P62333	PSMC6	26S proteasome regulatory subunit 10B OS=Homo sapiens OX=9606 GN=PSMC6 PE=1 SV=1	14	0,67	2,039	1,327	22853634,88	15116066,53	10980972,05
Q13895	BYSL	Bystin OS=Homo sapiens OX=9606 GN=BYSL PE=1 SV=3	5	0,427	2,265	1,327	2170158,717	1041053,283	943142,5785
Q9HD20	ATP13A1	Endoplasmic reticulum transmembrane helix translocase OS=Homo sapiens OX=9606 GN=ATP13	10	3,135	0,45	1,326	2374594,476	5156923,494	4446077,986
O75083	WDR1	WD repeat-containing protein 1 OS=Homo sapiens OX=9606 GN=WDR1 PE=1 SV=4	18	1,626	0,87	1,326	23098492,01	37184221,87	24323032,04
Q969V3	NCLN	BOS complex subunit NCLN OS=Homo sapiens OX=9606 GN=NCLN PE=1 SV=2	13	1,433	0,988	1,326	6120079,692	10054003,77	6873494,606
P13995	MTHFD2	Bifunctional methylenetetrahydrofolate dehydrogenase/cyclohydrolase, mitochondrial OS=Hom	7	0,964	1,038	1,326	6696105,333	7832961,905	6703982,995
O95373	IPO7	Importin-7 OS=Homo sapiens OX=9606 GN=IPO7 PE=1 SV=1	13	0,597	2,205	1,326	30779926,35	31960286,82	18074415,9
Q9NVJ2	ARL8B	ADP-ribosylation factor-like protein 8B OS=Homo sapiens OX=9606 GN=ARL8B PE=1 SV=1	6	1,533	0,827	1,324	7529876,33	12244622,96	7937998,553
Q8TAQ2	SMARCC2	SWI/SNF complex subunit SMARCC2 OS=Homo sapiens OX=9606 GN=SMARCC2 PE=1 SV=1	8	0,757	1,716	1,324	7875879,201	5750243,448	4246512,363
Q9UH62	ARMCX3	Armadillo repeat-containing X-linked protein 3 OS=Homo sapiens OX=9606 GN=ARMCX3 PE=1 SV	5	0,873	1,545	1,322	2238132,407	1332643,44	1521596,561
P56556	NDUFA6	NADH dehydrogenase [ubiquinone] 1 alpha subcomplex subunit 6 OS=Homo sapiens OX=9606 G	4	1,981	0,923	1,321	4939875,698	7724649,798	6050429,643
P40855	PEX19	Peroxisomal biogenesis factor 19 OS=Homo sapiens OX=9606 GN=PEX19 PE=1 SV=1	3	0,968	1,34	1,321	960667,8863	421986,405	706619,3096
O43169	CYB5B	Cytochrome b5 type B OS=Homo sapiens OX=9606 GN=CYB5B PE=1 SV=3	6	2,963	0,538	1,32	8879468,447	37111704,12	33795572,03
P29728	OAS2	2'-5'-oligoadenylate synthase 2 OS=Homo sapiens OX=9606 GN=OAS2 PE=1 SV=3	11	11,096	0,099	1,319	2943227,041	21377220,5	14839428,66
P36404	ARL2	ADP-ribosylation factor-like protein 2 OS=Homo sapiens OX=9606 GN=ARL2 PE=1 SV=4	3	0,603	2,264	1,317	1100818,657	642526,6354	727057,3346
O00754	MAN2B1	Lysosomal alpha-mannosidase OS=Homo sapiens OX=9606 GN=MAN2B1 PE=1 SV=3	6	2,015	0,693	1,316	1614211,868	3230035,415	2427036,976
P61160	ACTR2	Actin-related protein 2 OS=Homo sapiens OX=9606 GN=ACTR2 PE=1 SV=1	7	1,543	0,862	1,316	11119666,36	17058380,69	13246622,25
P41227	NAA10	N-alpha-acetyltransferase 10 OS=Homo sapiens OX=9606 GN=NAA10 PE=1 SV=1	5	0,645	2,165	1,316	3066694,388	2001536,324	1435367,483
Q9NRN7	AASDHPPT	L-aminoadipate-semialdehyde dehydrogenase-phosphopantetheinyl transferase OS=Homo sapie	3	0,361	4,104	1,314	1320918,136	501101,7217	452287,878
O60333	KIF1B	Kinesin-like protein KIF1B OS=Homo sapiens OX=9606 GN=KIF1B PE=1 SV=5	3	0,37	3,63	1,312	1233409,176	168603,6373	404429,9078
Q9BT09	CNPY3	Protein canopy homolog 3 OS=Homo sapiens OX=9606 GN=CNPY3 PE=1 SV=1	2	0,567	2,03	1,31	1599749,274	880701,1735	837478,2245
Q9ULK4	MED23	Mediator of RNA polymerase II transcription subunit 23 OS=Homo sapiens OX=9606 GN=MED23	2	1,835	0,748	1,309	103051,5079	162344,2228	137773,1535
A0A0A0MQS9	LAMA4	Laminin subunit alpha 4 OS=Homo sapiens OX=9606 GN=LAMA4 PE=1 SV=1	6	1,561	1,391	1,308	9635287,643	6261703,739	10096335,41
Q9UJ70	NAGK	N-acetyl-D-glucosamine kinase OS=Homo sapiens OX=9606 GN=NAGK PE=1 SV=4	6	1,151	1,05	1,308	2173841,408	2646108,45	2071625,657
P61586	RHOA	Transforming protein RhoA OS=Homo sapiens OX=9606 GN=RHOA PE=1 SV=1	2	1,001	1,021	1,308	7432665,865	9996640,661	6862378,22
Q06124	PTPN11	Tyrosine-protein phosphatase non-receptor type 11 OS=Homo sapiens OX=9606 GN=PTPN11 PE=	4	0,337	3,655	1,308	1674940,214	389735,7013	395776,0719
P84095	RHOG	Rho-related GTP-binding protein RhoG OS=Homo sapiens OX=9606 GN=RHOG PE=1 SV=1	4	4,042	0,367	1,307	1789595,63	6062159,634	5445599,777
Q9C0C2	TNKS1BP1	182 kDa tankyrase-1-binding protein OS=Homo sapiens OX=9606 GN=TNKS1BP1 PE=1 SV=4	28	2,741	0,515	1,304	5724283,321	18668276,02	10788314,06
P78527	PRKDC	DNA-dependent protein kinase catalytic subunit OS=Homo sapiens OX=9606 GN=PRKDC PE=1 SV	106	0,957	1,357	1,303	172274807,5	150907362,6	121266641,9
O75306	NDUFS2	NADH dehydrogenase [ubiquinone] iron-sulfur protein 2, mitochondrial OS=Homo sapiens OX=9	11	1,937	0,768	1,302	12436936,63	15176606,2	8850980,488
Q9Y2Z4	YARS2	Tyrosine--tRNA ligase, mitochondrial OS=Homo sapiens OX=9606 GN=YARS2 PE=1 SV=2	10	0,956	1,545	1,302	1848470,281	1758788,734	2108503,63
O95302	FKBP9	Peptidyl-prolyl cis-trans isomerase FKBP9 OS=Homo sapiens OX=9606 GN=FKBP9 PE=1 SV=2	6	1,306	1,003	1,299	5346921,767	7188783,403	5360826,892
Q12955	ANK3	Ankyrin-3 OS=Homo sapiens OX=9606 GN=ANK3 PE=1 SV=3	21	1,104	1,155	1,295	1806685,939	4100347,184	3457252,6
A0A8V8TNT7	WDR1	WD repeat domain 1 OS=Homo sapiens OX=9606 GN=WDR1 PE=1 SV=1	17	1,388	1,016	1,294	33791112,5	38681023,15	32605788,5
P17612	PRKACA	cAMP-dependent protein kinase catalytic subunit alpha OS=Homo sapiens OX=9606 GN=PRKACA	5	1,457	0,945	1,293	6597335,769	7677419,305	7713992,636
P30533	LRPAP1	Alpha-2-macroglobulin receptor-associated protein OS=Homo sapiens OX=9606 GN=LRPAP1 PE=	12	0,829	1,53	1,293	11617476,44	9047888,884	7340805,051
P55072	VCP	Transitional endoplasmic reticulum ATPase OS=Homo sapiens OX=9606 GN=VCP PE=1 SV=4	30	0,858	1,671	1,292	133099974,7	101602294,4	81629566,55
P0DMV9	HSPA1B	Heat shock 70 kDa protein 1B OS=Homo sapiens OX=9606 GN=HSPA1B PE=1 SV=1	27	1,573	0,836	1,291	63727729,14	105830738,9	85386309,31
O75448	MED24	Mediator of RNA polymerase II transcription subunit 24 OS=Homo sapiens OX=9606 GN=MED24	2	1,224	0,854	1,291	164260,774	199479,3041	464709,2305
Q9Y5U9	IER3IP1	Immediate early response 3-interacting protein 1 OS=Homo sapiens OX=9606 GN=IER3IP1 PE=1	2	1,641	0,787	1,289	3576047,464	5745045,768	4738957,219
P43034	PAFAH1B1	Platelet-activating factor acetylhydrolase IB subunit beta OS=Homo sapiens OX=9606 GN=PAFAH	6	0,52	2,474	1,289	8696370,776	4344094,629	3417137,344
O15305	PMM2	Phosphomannomutase 2 OS=Homo sapiens OX=9606 GN=PMM2 PE=1 SV=1	2	2,19	0,625	1,288	416833,5627	789030,5734	669990,934
Q6NZY4	ZCCHC8	Zinc finger CCHC domain-containing protein 8 OS=Homo sapiens OX=9606 GN=ZCCHC8 PE=1 SV=	4	1,99	0,659	1,288	862146,7588	1629169,171	1321450,376
P51149	RAB7A	Ras-related protein Rab-7a OS=Homo sapiens OX=9606 GN=RAB7A PE=1 SV=1	13	1,183	1,12	1,288	55328174,39	63020448,28	53715257,46
Q8IWZ3	ANKHD1	Ankyrin repeat and KH domain-containing protein 1 OS=Homo sapiens OX=9606 GN=ANKHD1 PE	7	0,569	2,722	1,288	3824859,069	2317466,79	1162507,438
P46926	GNPDA1	Glucosamine-6-phosphate isomerase 1 OS=Homo sapiens OX=9606 GN=GNPDA1 PE=1 SV=1	7	0,557	3,085	1,288	7034867,277	4103933,161	3010776,358
P27348	YWHAQ	14-3-3 protein theta OS=Homo sapiens OX=9606 GN=YWHAQ PE=1 SV=1	7	0,536	2,45	1,288	69618308,17	38762806,55	29218509,78
Q92817	EVPL	Envoplakin OS=Homo sapiens OX=9606 GN=EVPL PE=1 SV=3	18	2,871	0,303	1,287	395517,2665	2561680,227	5257663,071
P49770	EIF2B2	Translation initiation factor eIF2B subunit beta OS=Homo sapiens OX=9606 GN=EIF2B2 PE=1 SV=	4	0,636	1,933	1,287	1489710,16	809183,8067	645311,3573
Q13765	NACA	Nascent polypeptide-associated complex subunit alpha OS=Homo sapiens OX=9606 GN=NACA PI	3	0,356	3,97	1,287	16031119,79	5525973,849	5119614,83
P61011	SRP54	Signal recognition particle subunit SRP54 OS=Homo sapiens OX=9606 GN=SRP54 PE=1 SV=1	8	1,136	1,164	1,286	5445989,144	5631200,231	5044752,867
P25787	PSMA2	Proteasome subunit alpha type-2 OS=Homo sapiens OX=9606 GN=PSMA2 PE=1 SV=2	7	0,828	1,987	1,286	12281525	7804028,322	6384726,383
P17812	CTPS1	CTP synthase 1 OS=Homo sapiens OX=9606 GN=CTPS1 PE=1 SV=2	7	0,351	3,701	1,285	14275754,08	4953046,758	3765609,211
Q00587	CDC42EP1	Cdc42 effector protein 1 OS=Homo sapiens OX=9606 GN=CDC42EP1 PE=1 SV=1	2	5,79	0,188	1,284	112880,851	1156725,81	840759,2903
P06280	GLA	Alpha-galactosidase A OS=Homo sapiens OX=9606 GN=GLA PE=1 SV=1	3	0,834	1,351	1,284	1391206,401	1156934,588	833291,247
P51610	HCFC1	Host cell factor 1 OS=Homo sapiens OX=9606 GN=HCFC1 PE=1 SV=2	17	0,798	1,716	1,284	14504275,55	8198667,338	8050902,516
P46934	NEDD4	E3 ubiquitin-protein ligase NEDD4 OS=Homo sapiens OX=9606 GN=NEDD4 PE=1 SV=4	2	4,414	0,521	1,283	75065,26995	312440,6104	104867,9394
Q6PI78	TMEM65	Transmembrane protein 65 OS=Homo sapiens OX=9606 GN=TMEM65 PE=1 SV=2	3	2,072	0,507	1,283	1800921,918	4153666,66	3179787,179
O43181	NDUFS4	NADH dehydrogenase [ubiquinone] iron-sulfur protein 4, mitochondrial OS=Homo sapiens OX=9	3	1,76	0,705	1,283	3139150,802	5806536,506	4709823,975
O60684	KPNA6	Importin subunit alpha-7 OS=Homo sapiens OX=9606 GN=KPNA6 PE=1 SV=1	2	1,355	1,042	1,283	3449879,897	5260788,18	4916791,002
Q9Y5M8	SRPRB	Signal recognition particle receptor subunit beta OS=Homo sapiens OX=9606 GN=SRPRB PE=1 SV	8	1,345	1,026	1,283	10215217,9	13237870,13	12009507,38
P42892	ECE1	Endothelin-converting enzyme 1 OS=Homo sapiens OX=9606 GN=ECE1 PE=1 SV=2	8	3,461	0,352	1,282	1215679,78	4342998,862	3629150,666
P54920	NAPA	Alpha-soluble NSF attachment protein OS=Homo sapiens OX=9606 GN=NAPA PE=1 SV=3	11	1,777	0,743	1,281	9938022,557	15479196,26	14458168,13
P43686	PSMC4	26S proteasome regulatory subunit 6B OS=Homo sapiens OX=9606 GN=PSMC4 PE=1 SV=2	16	0,746	2,075	1,28	17090205,49	11666464,5	8709060,42
P06702	S100A9	Protein S100-A9 OS=Homo sapiens OX=9606 GN=S100A9 PE=1 SV=1	3	2,946	0,487	1,279	640068,1882	1674559,394	1362689,113
Q96C86	DCPS	m7GpppX diphosphatase OS=Homo sapiens OX=9606 GN=DCPS PE=1 SV=2	4	0,584	2,049	1,278	1159082,523	650901,3166	1206370,045
P50395	GDI2	Rab GDP dissociation inhibitor beta OS=Homo sapiens OX=9606 GN=GDI2 PE=1 SV=2	14	0,515	2,551	1,276	73882740,58	27718933,5	29914305,94
P49247	RPIA	Ribose-5-phosphate isomerase OS=Homo sapiens OX=9606 GN=RPIA PE=1 SV=3	2	0,433	2,655	1,276	688891,1878	287534,2291	314493,7559
Q6YN16	HSDL2	Hydroxysteroid dehydrogenase-like protein 2 OS=Homo sapiens OX=9606 GN=HSDL2 PE=1 SV=1	11	2,931	0,437	1,275	3083149,664	11252307,42	8846095,871
P49257	LMAN1	Protein ERGIC-53 OS=Homo sapiens OX=9606 GN=LMAN1 PE=1 SV=2	13	1,302	0,934	1,275	25542495,13	31473304,7	25340573,41
Q96S52	PIGS	GPI transamidase component PIG-S OS=Homo sapiens OX=9606 GN=PIGS PE=1 SV=3	8	1,286	1,088	1,273	3421783,804	3318458,573	2865201,804
P55039	DRG2	Developmentally-regulated GTP-binding protein 2 OS=Homo sapiens OX=9606 GN=DRG2 PE=1 S	2	0,408	1,493	1,273	882849,0353	84680,045	623352,651
Q8WUM4	PDCD6IP	Programmed cell death 6-interacting protein OS=Homo sapiens OX=9606 GN=PDCD6IP PE=1 SV=	23	1,426	0,853	1,272	16118017,37	20318144,15	18516434,63
P61201	COPS2	COP9 signalosome complex subunit 2 OS=Homo sapiens OX=9606 GN=COPS2 PE=1 SV=1	9	0,561	1,967	1,271	6289433,83	3072907,722	2692284,007
Q5T4S7	UBR4	E3 ubiquitin-protein ligase UBR4 OS=Homo sapiens OX=9606 GN=UBR4 PE=1 SV=1	4	0,541	1,958	1,271	1766550,827	711640,5057	815566,5178
O75934	BCAS2	Pre-mRNA-splicing factor SPF27 OS=Homo sapiens OX=9606 GN=BCAS2 PE=1 SV=1	8	0,864	1,142	1,27	5800657,051	4999999,421	5810190,856
P36871	PGM1	Phosphoglucomutase-1 OS=Homo sapiens OX=9606 GN=PGM1 PE=1 SV=3	9	1,339	1,02	1,268	3171520,879	4405838,478	3025671,774
Q9H0U3	MAGT1	Magnesium transporter protein 1 OS=Homo sapiens OX=9606 GN=MAGT1 PE=1 SV=1	5	0,924	0,958	1,268	5451498,706	5140498,158	4688600,168
Q8TCJ2	STT3B	Dolichyl-diphosphooligosaccharide--protein glycosyltransferase subunit STT3B OS=Homo sapiens	7	0,723	1,628	1,268	9078279,425	6474835,603	5138373,982
P19174	PLCG1	1-phosphatidylinositol 4,5-bisphosphate phosphodiesterase gamma-1 OS=Homo sapiens OX=96	9	0,344	3,286	1,266	9714718,718	1821681,461	1440957,9
Q92544	TM9SF4	Transmembrane 9 superfamily member 4 OS=Homo sapiens OX=9606 GN=TM9SF4 PE=1 SV=2	8	1,47	0,904	1,265	4165948,693	7239343,876	5037327,837
P36873	PPP1CC	Serine/threonine-protein phosphatase PP1-gamma catalytic subunit OS=Homo sapiens OX=9606	5	0,747	1,745	1,265	17774315,99	12344244,88	9175066,713
Q9P0J0	NDUFA13	NADH dehydrogenase [ubiquinone] 1 alpha subcomplex subunit 13 OS=Homo sapiens OX=9606	7	1,561	0,833	1,264	4827043,811	11563188,92	11911345,09
P14678	SNRPB	Small nuclear ribonucleoprotein-associated proteins B and B' OS=Homo sapiens OX=9606 GN=SN	7	0,936	1,208	1,264	12505496,75	11090763,85	10193785,41
O95433	AHSA1	Activator of 90 kDa heat shock protein ATPase homolog 1 OS=Homo sapiens OX=9606 GN=AHSA	5	0,383	2,685	1,264	6569537,605	2357643,046	2109448,532
Q9Y3A6	TMED5	Transmembrane emp24 domain-containing protein 5 OS=Homo sapiens OX=9606 GN=TMED5 PE	5	1,221	1,033	1,263	7972390,628	9907147,487	8846960,74
P46109	CRKL	Crk-like protein OS=Homo sapiens OX=9606 GN=CRKL PE=1 SV=1	4	0,547	2,521	1,263	3995627,275	2124058,627	1923251,656
P62330	ARF6	ADP-ribosylation factor 6 OS=Homo sapiens OX=9606 GN=ARF6 PE=1 SV=2	5	2,081	0,476	1,26	2834931,058	5745740,43	4716717,453
Q13867	BLMH	Bleomycin hydrolase OS=Homo sapiens OX=9606 GN=BLMH PE=1 SV=1	5	0,355	2,911	1,259	6647660,87	2219643,97	1849169,085
Q92890	UFD1	Ubiquitin recognition factor in ER-associated degradation protein 1 OS=Homo sapiens OX=9606	7	1,084	1,184	1,258	5260723,547	6008637,718	4786261,178
Q9UHD1	CHORDC1	Cysteine and histidine-rich domain-containing protein 1 OS=Homo sapiens OX=9606 GN=CHORD	4	0,439	2,685	1,257	2147404,74	908384,3257	650868,004
O14929	HAT1	Histone acetyltransferase type B catalytic subunit OS=Homo sapiens OX=9606 GN=HAT1 PE=1 SV	5	0,465	2,402	1,256	6434923,148	1963126,92	2715332,502
O60341	KDM1A	Lysine-specific histone demethylase 1A OS=Homo sapiens OX=9606 GN=KDM1A PE=1 SV=2	14	0,307	3,614	1,256	12619350,5	3485041,467	3079857,073
O14672	ADAM10	Disintegrin and metalloproteinase domain-containing protein 10 OS=Homo sapiens OX=9606 GN	5	2,033	0,544	1,253	864731,5306	4066401,055	5722179,63
P32969	RPL9	Large ribosomal subunit protein uL6 OS=Homo sapiens OX=9606 GN=RPL9 PE=1 SV=1	6	0,984	1,053	1,252	21729337,07	19657500,91	18419334,41
P61353	RPL27	Large ribosomal subunit protein eL27 OS=Homo sapiens OX=9606 GN=RPL27 PE=1 SV=2	5	0,977	1,362	1,25	49361821,33	38101179,77	37535918,95
Q8IXB1	DNAJC10	DnaJ homolog subfamily C member 10 OS=Homo sapiens OX=9606 GN=DNAJC10 PE=1 SV=2	7	0,711	1,886	1,249	2214073,115	1379522,492	1171672,837
Q04917	YWHAH	14-3-3 protein eta OS=Homo sapiens OX=9606 GN=YWHAH PE=1 SV=4	7	0,799	1,36	1,248	17291333,54	17183323,95	13683638,49
O75907	DGAT1	Diacylglycerol O-acyltransferase 1 OS=Homo sapiens OX=9606 GN=DGAT1 PE=1 SV=2	2	1,619	0,586	1,244	281206,8776	208056,0898	465950,8361

Q9UNM6	PSMD13	26S proteasome non-ATPase regulatory subunit 13 OS=Homo sapiens OX=9606 GN=PSMD13 PE=	16	0,881	1,392	1,244	19249603,32	16553361,17	14830958,31
P63000	RAC1	Ras-related C3 botulinum toxin substrate 1 OS=Homo sapiens OX=9606 GN=RAC1 PE=1 SV=1	2	2,051	0,94	1,243	9393844,826	17773766,84	11780106,87
Q9BVL2	NUP58	Nucleoporin p58/p45 OS=Homo sapiens OX=9606 GN=NUP58 PE=1 SV=1	4	1,1	1,148	1,243	1642281,478	1807485,518	1467536,633
A0A6Q8PFU3	PAFAH1B1	Platelet-activating factor acetylhydrolase IB subunit alpha OS=Homo sapiens OX=9606 GN=PAFA	7	0,509	2,645	1,243	8935284,102	4055587,583	3516295,227
P41240	CSK	Tyrosine-protein kinase CSK OS=Homo sapiens OX=9606 GN=CSK PE=1 SV=1	5	0,44	3,833	1,242	1643316,449	457801,4024	202448,3679
P50991	CCT4	T-complex protein 1 subunit delta OS=Homo sapiens OX=9606 GN=CCT4 PE=1 SV=4	17	0,678	1,828	1,241	92258583,41	59472916,13	51675341,49
P51553	IDH3G	Isocitrate dehydrogenase [NAD] subunit gamma, mitochondrial OS=Homo sapiens OX=9606 GN=	3	3,505	0,376	1,239	602726,3762	1884050,784	1699113,801
Q9Y696	CLIC4	Chloride intracellular channel protein 4 OS=Homo sapiens OX=9606 GN=CLIC4 PE=1 SV=4	8	0,395	2,809	1,239	21207388,94	9345952,332	6704064,963
Q15833	STXBP2	Syntaxin-binding protein 2 OS=Homo sapiens OX=9606 GN=STXBP2 PE=1 SV=2	8	1000	0,001	1,238		1536115,494	1686160,891
P30086	PEBP1	Phosphatidylethanolamine-binding protein 1 OS=Homo sapiens OX=9606 GN=PEBP1 PE=1 SV=3	8	0,318	3,688	1,238	47429660,28	14575787,41	10538408,19
P24539	ATP5PB	ATP synthase F(0) complex subunit B1, mitochondrial OS=Homo sapiens OX=9606 GN=ATP5PB P	10	1,265	0,592	1,237	13516423,6	16181451,33	18881242,08
Q9UIJ7	AK3	GTP:AMP phosphotransferase AK3, mitochondrial OS=Homo sapiens OX=9606 GN=AK3 PE=1 SV=	11	2,122	0,607	1,236	4643073,791	11892031,43	8576018,494
P61923	COPZ1	Coatomer subunit zeta-1 OS=Homo sapiens OX=9606 GN=COPZ1 PE=1 SV=1	4	0,876	1,333	1,236	7563639,44	6770962,544	5674667,137
Q9UHN6	CEMIP2	Inactive cell surface hyaluronidase CEMIP2 OS=Homo sapiens OX=9606 GN=CEMIP2 PE=1 SV=1	2	0,933	1,311	1,235	337377,1083	291765,7329	237850,3888
P23528	CFL1	Cofilin-1 OS=Homo sapiens OX=9606 GN=CFL1 PE=1 SV=3	5	0,675	2,161	1,235	167608271,9	85257284,54	78290052,57
Q16181	SEPTIN7	Septin-7 OS=Homo sapiens OX=9606 GN=SEPTIN7 PE=1 SV=2	12	0,462	2,815	1,235	35208074,62	15867445,64	14171423,48
O43390	HNRNPR	Heterogeneous nuclear ribonucleoprotein R OS=Homo sapiens OX=9606 GN=HNRNPR PE=1 SV=:	18	0,92	1,412	1,234	96698570,33	84227836,11	68026725,35
P60900	PSMA6	Proteasome subunit alpha type-6 OS=Homo sapiens OX=9606 GN=PSMA6 PE=1 SV=1	8	1,048	1,303	1,232	45263012,69	52188631,06	85497494,03
P36543	ATP6V1E1	V-type proton ATPase subunit E 1 OS=Homo sapiens OX=9606 GN=ATP6V1E1 PE=1 SV=1	6	1,04	1,055	1,232	6407056,295	6147046,687	4556850,135
P62081	RPS7	Small ribosomal subunit protein eS7 OS=Homo sapiens OX=9606 GN=RPS7 PE=1 SV=1	9	0,726	1,563	1,23	41585921,87	31594053,59	34418476,99
O15143	ARPC1B	Actin-related protein 2/3 complex subunit 1B OS=Homo sapiens OX=9606 GN=ARPC1B PE=1 SV=	6	2,418	0,517	1,227	5241149	12872272,05	10290665,77
Q8NFF5	FLAD1	FAD synthase OS=Homo sapiens OX=9606 GN=FLAD1 PE=1 SV=1	3	1,043	1,399	1,226	702575,0645	691701,6443	294656,4354
P55795	HNRNPH2	Heterogeneous nuclear ribonucleoprotein H2 OS=Homo sapiens OX=9606 GN=HNRNPH2 PE=1 S'	5	0,84	1,528	1,225	5099684,522	4165344,203	3752798,727
Q9NWY4	HPF1	Histone PARylation factor 1 OS=Homo sapiens OX=9606 GN=HPF1 PE=1 SV=2	2	0,27	4,54	1,225	445325,7493	48575,49697	39648,91087
O14653	GOSR2	Golgi SNAP receptor complex member 2 OS=Homo sapiens OX=9606 GN=GOSR2 PE=1 SV=2	2	1,725	0,669	1,223	460166,3774	832431,1827	851592,702
Q8IWA4	MFN1	Mitofusin-1 OS=Homo sapiens OX=9606 GN=MFN1 PE=1 SV=3	2	0,792	0,904	1,221	902634,1791	268993,705	1042102,661
Q96Q06	PLIN4	Perilipin-4 OS=Homo sapiens OX=9606 GN=PLIN4 PE=1 SV=3	5	1000	0,001	1,22		516880,9259	171196,7496
P52789	HK2	Hexokinase-2 OS=Homo sapiens OX=9606 GN=HK2 PE=1 SV=2	2	1,357	0,892	1,22	264179,7286	464174,9909	296314,9609
Q06830	PRDX1	Peroxiredoxin-1 OS=Homo sapiens OX=9606 GN=PRDX1 PE=1 SV=1	8	0,388	3,022	1,22	225692756,8	93345524,65	81482680,12
Q96RT1	ERBIN	Erbin OS=Homo sapiens OX=9606 GN=ERBIN PE=1 SV=2	5	2,161	0,523	1,219	138735,549	468265,4832	440923,4835
P52597	HNRNPF	Heterogeneous nuclear ribonucleoprotein F OS=Homo sapiens OX=9606 GN=HNRNPF PE=1 SV=3	10	0,93	0,758	1,219	26428584,42	23992547,99	33954577,26
O43242	PSMD3	26S proteasome non-ATPase regulatory subunit 3 OS=Homo sapiens OX=9606 GN=PSMD3 PE=1 :	17	0,792	1,607	1,219	24541992,98	16231268,68	13049766,18
Q9BVT8	TMUB1	Transmembrane and ubiquitin-like domain-containing protein 1 OS=Homo sapiens OX=9606 GN=	3	1,855	0,714	1,218	1713832,281	3138328,293	2418857,501
P29692	EEF1D	Elongation factor 1-delta OS=Homo sapiens OX=9606 GN=EEF1D PE=1 SV=5	12	0,707	1,271	1,218	54022733,97	37421949,55	44833474,28
P53621	COPA	Coatomer subunit alpha OS=Homo sapiens OX=9606 GN=COPA PE=1 SV=2	21	0,951	1,363	1,216	24732498,61	21129097,28	18810451,33
Q9Y6N5	SQOR	Sulfide:quinone oxidoreductase, mitochondrial OS=Homo sapiens OX=9606 GN=SQOR PE=1 SV=:	13	8,136	0,183	1,215	121160,2516	31534483,97	26771468,46
P48556	PSMD8	26S proteasome non-ATPase regulatory subunit 8 OS=Homo sapiens OX=9606 GN=PSMD8 PE=1 :	6	0,848	1,513	1,215	8484074,104	6250077,311	3979809,342
Q6NUK1	SLC25A24	Mitochondrial adenyl nucleotide antiporter SLC25A24 OS=Homo sapiens OX=9606 GN=SLC25A24	16	1,379	0,748	1,214	12004691,95	16401750,04	20103272,67
Q12765	SCRN1	Secernin-1 OS=Homo sapiens OX=9606 GN=SCRN1 PE=1 SV=2	8	0,5	2,448	1,214	7376394,446	2974825,915	2472112,166
Q9BRK5	SDF4	45 kDa calcium-binding protein OS=Homo sapiens OX=9606 GN=SDF4 PE=1 SV=1	4	2,191	0,573	1,212	1403543,888	3205948,557	2668992,059
Q8WVC0	LEO1	RNA polymerase-associated protein LEO1 OS=Homo sapiens OX=9606 GN=LEO1 PE=1 SV=1	2	1,161	1,044	1,211	546965,5945	601575,6584	499703,735
P52209	PGD	6-phosphogluconate dehydrogenase, decarboxylating OS=Homo sapiens OX=9606 GN=PGD PE=1	16	0,152	7,988	1,211	98765834,65	11162162,13	11574844,51
Q6IBS0	TWF2	Twinfilin-2 OS=Homo sapiens OX=9606 GN=TWF2 PE=1 SV=2	6	1,488	0,83	1,21	1840598,904	3234010,103	3063000,089
P62979	RPS27A	Ubiquitin-ribosomal protein eS31 fusion protein OS=Homo sapiens OX=9606 GN=RPS27A PE=1 S	5	0,994	1,333	1,21	252388348,9	253599655,3	204731067,1
O75436	VPS26A	Vacuolar protein sorting-associated protein 26A OS=Homo sapiens OX=9606 GN=VPS26A PE=1 S'	5	0,992	1,317	1,21	6680617,278	5740647,103	5397721,967
Q9UBT2	UBA2	SUMO-activating enzyme subunit 2 OS=Homo sapiens OX=9606 GN=UBA2 PE=1 SV=2	10	0,788	1,508	1,209	12461844,8	9296348,27	8077902,021
Q9BUP3	HTATIP2	Oxidoreductase HTATIP2 OS=Homo sapiens OX=9606 GN=HTATIP2 PE=1 SV=2	3	4,079	0,251	1,208	384001,471	3860037,418	3021470,401
Q96EK6	GNPNAT1	Glucosamine 6-phosphate N-acetyltransferase OS=Homo sapiens OX=9606 GN=GNPNAT1 PE=1 S	2	0,643	1,994	1,208	619145,8639	398687,1963	396490,691
P51784	USP11	Ubiquitin carboxyl-terminal hydrolase 11 OS=Homo sapiens OX=9606 GN=USP11 PE=1 SV=3	3	0,524	2,35	1,208	1429204,63	703206,2453	592651,5006
P57105	SYNJ2BP	Synaptojanin-2-binding protein OS=Homo sapiens OX=9606 GN=SYNJ2BP PE=1 SV=2	7	1,429	0,919	1,207	2756241,034	6428978,592	3988998,559
Q99497	PARK7	Parkinson disease protein 7 OS=Homo sapiens OX=9606 GN=PARK7 PE=1 SV=2	6	0,518	2,396	1,207	30091959,58	15851174,74	13198206,73
Q8N201	INTS1	Integrator complex subunit 1 OS=Homo sapiens OX=9606 GN=INTS1 PE=1 SV=2	3	0,981	0,996	1,202	556729,7692	1079218,312	832515,198
O96019	ACTL6A	Actin-like protein 6A OS=Homo sapiens OX=9606 GN=ACTL6A PE=1 SV=1	10	0,683	1,593	1,2	11237951,25	6052206,802	8234813,821
Q9Y5J1	UTP18	U3 small nucleolar RNA-associated protein 18 homolog OS=Homo sapiens OX=9606 GN=UTP18 F	2	1,83	0,632	1,199	528141,346	963031,1871	835656,6571
Q16563	SYPL1	Synaptophysin-like protein 1 OS=Homo sapiens OX=9606 GN=SYPL1 PE=1 SV=1	3	1,288	0,364	1,199	2296779,71	6443825,516	5546986,399
P11177	PDHB	Pyruvate dehydrogenase E1 component subunit beta, mitochondrial OS=Homo sapiens OX=9606	11	0,93	1,262	1,199	20153355,16	18293099,21	13492462,34
Q6UWP7	LCLAT1	Lysocardiolipin acyltransferase 1 OS=Homo sapiens OX=9606 GN=LCLAT1 PE=1 SV=1	6	0,982	1,036	1,198	4283101,688	3056450,682	5852492,401
J3QRU1	YES1	Tyrosine-protein kinase OS=Homo sapiens OX=9606 GN=YES1 PE=1 SV=1	3	0,861	1,438	1,198	3472092,337	2646836,631	2511779,153
Q15155	NOMO1	BOS complex subunit NOMO1 OS=Homo sapiens OX=9606 GN=NOMO1 PE=1 SV=5	2	1,903	0,912	1,197	153233,7342	552743,8871	383000,1686
Q13596	SNX1	Sorting nexin-1 OS=Homo sapiens OX=9606 GN=SNX1 PE=1 SV=3	6	0,63	1,759	1,197	3895253,234	2414861,589	2138778,87
O15144	ARPC2	Actin-related protein 2/3 complex subunit 2 OS=Homo sapiens OX=9606 GN=ARPC2 PE=1 SV=1	11	1,571	0,752	1,195	17357752,08	22793762,28	18779684,32
P07954	FH	Fumarate hydratase, mitochondrial OS=Homo sapiens OX=9606 GN=FH PE=1 SV=3	15	1,518	0,813	1,195	41322367,46	58132809,11	50028657,51
P58107	EPPK1	Epiplakin OS=Homo sapiens OX=9606 GN=EPPK1 PE=1 SV=3	11	1,676	0,492	1,193	3269575,937	3903117,671	2411541,897
P23378	GLDC	Glycine dehydrogenase (decarboxylating), mitochondrial OS=Homo sapiens OX=9606 GN=GLDC F	8	1,637	1,546	1,193	845786,3674	462209,3956	630533,4146
P78371	CCT2	T-complex protein 1 subunit beta OS=Homo sapiens OX=9606 GN=CCT2 PE=1 SV=4	28	0,617	2,223	1,193	130617736,6	76735368,94	62402306,89
Q8TE77	SSH3	Protein phosphatase Slingshot homolog 3 OS=Homo sapiens OX=9606 GN=SSH3 PE=1 SV=2	6	1,856	0,655	1,192	1870521,889	2510028,48	2143851,395
Q9UNF0	PACSIN2	Protein kinase C and casein kinase substrate in neurons protein 2 OS=Homo sapiens OX=9606 GI	8	1,949	0,55	1,191	4081816,227	11041630,07	6856427,858
Q9Y2D5	PALM2AKAP2	PALM2-AKAP2 fusion protein OS=Homo sapiens OX=9606 GN=PALM2AKAP2 PE=1 SV=4	8	3,083	0,428	1,19	879321,8611	3293174,659	2132522,958
P17858	PFKL	ATP-dependent 6-phosphofructokinase, liver type OS=Homo sapiens OX=9606 GN=PFKL PE=1 SV	7	1,042	1,089	1,19	4289422,82	4393775,277	4244424,164
Q9NZ08	ERAP1	Endoplasmic reticulum aminopeptidase 1 OS=Homo sapiens OX=9606 GN=ERAP1 PE=1 SV=3	27	9,746	0,131	1,189	3790699,094	31842283,41	35088766,78
P49441	INPP1	Inositol polyphosphate 1-phosphatase OS=Homo sapiens OX=9606 GN=INPP1 PE=1 SV=1	3	2,003	0,848	1,189	139050,853	475393,5632	417510,6075
O75531	BANF1	Barrier-to-autointegration factor OS=Homo sapiens OX=9606 GN=BANF1 PE=1 SV=1	3	1,081	0,797	1,189	21790851,05	25841742,66	30274015,69
Q15008	PSMD6	26S proteasome non-ATPase regulatory subunit 6 OS=Homo sapiens OX=9606 GN=PSMD6 PE=1 :	13	0,794	1,665	1,189	19107483,86	13928852,52	11287106,08
Q9H5K3	POMK	Protein O-mannose kinase OS=Homo sapiens OX=9606 GN=POMK PE=1 SV=1	2	0,775	1,353	1,189	501575,387	468588,4737	392840,3774
Q8WZA0	LZIC	Protein LZIC OS=Homo sapiens OX=9606 GN=LZIC PE=1 SV=1	2	0,69	1,914	1,189	641003,1654	392504,5759	353929,3251
Q15796	SMAD2	Mothers against decapentaplegic homolog 2 OS=Homo sapiens OX=9606 GN=SMAD2 PE=1 SV=1	2	0,574	1,984	1,189	1186036,184	681051,1619	572940,1876
O14561	NDUFAB1	Acyl carrier protein, mitochondrial OS=Homo sapiens OX=9606 GN=NDUFAB1 PE=1 SV=3	2	0,606	2,105	1,188	3247455,084	1786420,911	1469259,336
Q14CN2	CLCA4	Calcium-activated chloride channel regulator 4 OS=Homo sapiens OX=9606 GN=CLCA4 PE=1 SV=:	14			1,187	80583,9898	207794,8706	174989,0886
P35998	PSMC2	26S proteasome regulatory subunit 7 OS=Homo sapiens OX=9606 GN=PSMC2 PE=1 SV=3	15	0,807	1,542	1,187	27742822,78	23482398,44	21458641,19
Q8N2F6	ARMC10	Armadillo repeat-containing protein 10 OS=Homo sapiens OX=9606 GN=ARMC10 PE=1 SV=1	4	1,627	0,675	1,185	427227,513	945387,8703	774705,4843
P52907	CAPZA1	F-actin-capping protein subunit alpha-1 OS=Homo sapiens OX=9606 GN=CAPZA1 PE=1 SV=3	8	1,2	1,055	1,185	27296645,47	33246507,99	26562838,49
P13010	XRCC5	X-ray repair cross-complementing protein 5 OS=Homo sapiens OX=9606 GN=XRCC5 PE=1 SV=3	26	0,658	1,769	1,185	85489832,21	54832155,33	51995971,24
Q16537	PPP2R5E	Serine/threonine-protein phosphatase 2A 56 kDa regulatory subunit epsilon isoform OS=Homo s	6	0,457	2,543	1,185	3047985,325	1483446,047	918436,2186
P51798	CLCN7	H(+)/Cl(-) exchange transporter 7 OS=Homo sapiens OX=9606 GN=CLCN7 PE=1 SV=2	2	1,542	1,016	1,184	768592,2177	1441269,344	1334608,759
Q9NRX2	MRPL17	Large ribosomal subunit protein bL17m OS=Homo sapiens OX=9606 GN=MRPL17 PE=1 SV=1	2	1,312	0,868	1,183	457101,6383	628021,7598	447531,9221
Q8N0U8	VKORC1L1	Vitamin K epoxide reductase complex subunit 1-like protein 1 OS=Homo sapiens OX=9606 GN=V	2	1,615	0,727	1,182	1483256,954	1919303,576	1804882,435
Q15046	KARS1	Lysine--tRNA ligase OS=Homo sapiens OX=9606 GN=KARS1 PE=1 SV=3	16	0,86	1,433	1,179	23241243,94	20056299,29	17672961,26
P52943	CRIP2	Cysteine-rich protein 2 OS=Homo sapiens OX=9606 GN=CRIP2 PE=1 SV=1	4	0,653	2,025	1,179	8523566,932	5181358,92	4415029,73
P40925	MDH1	Malate dehydrogenase, cytoplasmic OS=Homo sapiens OX=9606 GN=MDH1 PE=1 SV=4	9	0,508	2,096	1,179	50740389,85	25509430,38	24926187,02
Q9NQ88	TIGAR	Fructose-2,6-bisphosphatase TIGAR OS=Homo sapiens OX=9606 GN=TIGAR PE=1 SV=1	2	0,461	2,515	1,178	207831,7584	86421,94332	73365,74983
Q9NPQ8	RIC8A	Synembryn-A OS=Homo sapiens OX=9606 GN=RIC8A PE=1 SV=3	5	0,728	1,39	1,176	1816381,604	1407404,019	1275638,707
Q9BRA2	TXNDC17	Thioredoxin domain-containing protein 17 OS=Homo sapiens OX=9606 GN=TXNDC17 PE=1 SV=1	3	0,306	3,841	1,175	8357639,339	2366817,922	2118849,961
P63098	PPP3R1	Calcineurin subunit B type 1 OS=Homo sapiens OX=9606 GN=PPP3R1 PE=1 SV=2	2	1,497	0,783	1,173	1032627,291	1321247,217	1320981,11
O95747	OXSR1	Serine/threonine-protein kinase OSR1 OS=Homo sapiens OX=9606 GN=OXSR1 PE=1 SV=1	7	0,859	1,289	1,173	8428035,529	4889915,943	6841992,884
Q14BN4	SLMAP	Sarcolemmal membrane-associated protein OS=Homo sapiens OX=9606 GN=SLMAP PE=1 SV=1	4	1,27	0,472	1,171	722452,4784	790182,6588	969421,7076
P30050	RPL12	Large ribosomal subunit protein uL11 OS=Homo sapiens OX=9606 GN=RPL12 PE=1 SV=1	2	0,897	1,158	1,171	16015074,97	13582244,9	14710291,3
Q86UE4	MTDH	Protein LYRIC OS=Homo sapiens OX=9606 GN=MTDH PE=1 SV=2	9	1,344	0,95	1,17	4212815,355	6025267,984	5337865,516
Q9H910	JPT2	Jupiter microtubule associated homolog 2 OS=Homo sapiens OX=9606 GN=JPT2 PE=1 SV=1	4	0,652	1,877	1,17	6233874,846	4161300,767	3453786,631
P06744	GPI	Glucose-6-phosphate isomerase OS=Homo sapiens OX=9606 GN=GPI PE=1 SV=4	17	1,358	0,841	1,169	29588233,71	42424272,41	37419449,97
P62310	LSM3	U6 snRNA-associated Sm-like protein LSm3 OS=Homo sapiens OX=9606 GN=LSM3 PE=1 SV=2	4	0,755	1,378	1,169	5520900,429	4292078,601	3724803,307
P61026	RAB10	Ras-related protein Rab-10 OS=Homo sapiens OX=9606 GN=RAB10 PE=1 SV=1	8	1,607	0,65	1,167	44019763,18	71991862,43	64156544,32
A0A9L9PXH6	NUCB1	Nucleobindin-1 OS=Homo sapiens OX=9606 GN=NUCB1 PE=1 SV=1	13	1,147	1,11	1,167	16205451,94	18432822,91	14513587,19
E7EPK1	SEPTIN7	Septin OS=Homo sapiens OX=9606 GN=SEPTIN7 PE=1 SV=2	12	0,401	3,588	1,167	34064216,7	13472459,73	12526000,14
Q12959	DLG1	Disks large homolog 1 OS=Homo sapiens OX=9606 GN=DLG1 PE=1 SV=2	12	2,24	0,528	1,164	2868074,763	6393275,192	6985583,758
Q13617	CUL2	Cullin-2 OS=Homo sapiens OX=9606 GN=CUL2 PE=1 SV=2	3	0,957	1,286	1,164	1097772,63	1018801,532	539352,3613
O43402	EMC8	ER membrane protein complex subunit 8 OS=Homo sapiens OX=9606 GN=EMC8 PE=1 SV=1	2	1,529	0,743	1,163	1199396,552	2064282,806	1375067,286
Q9H2P0	ADNP	Activity-dependent neuroprotector homeobox protein OS=Homo sapiens OX=9606 GN=ADNP PE	12	0,375	2,91	1,163	8951366,828	3475165,29	2548314,698
Q5EBM0	CMPK2	UMP-CMP kinase 2, mitochondrial OS=Homo sapiens OX=9606 GN=CMPK2 PE=1 SV=3	2	2,445	0,534	1,16	570365,9968	1477580,414	1058518,867
A5YKK6	CNOT1	CCR4-NOT transcription complex subunit 1 OS=Homo sapiens OX=9606 GN=CNOT1 PE=1 SV=2	16	0,614	1,628	1,159	7758066,352	3820004,12	4955788,721
Q9Y4Z0	LSM4	U6 snRNA-associated Sm-like protein LSm4 OS=Homo sapiens OX=9606 GN=LSM4 PE=1 SV=1	2	0,877	1,254	1,158	2652828,145	2100281,615	2838978,118
Q01433	AMPD2	AMP deaminase 2 OS=Homo sapiens OX=9606 GN=AMPD2 PE=1 SV=3	5	0,539	4,16	1,157	3192683,076	1378404,057	1068582,158

Q13601	KRR1	KRR1 small subunit processome component homolog OS=Homo sapiens OX=9606 GN=KRR1 PE=	3	1,296	0,854	1,155	1375895,939	1787051,513	1613736,367
O14497	ARID1A	AT-rich interactive domain-containing protein 1A OS=Homo sapiens OX=9606 GN=ARID1A PE=1	12	0,437	2,38	1,155	5198113,916	1815257,096	2101443,404
Q9HCD5	NCOA5	Nuclear receptor coactivator 5 OS=Homo sapiens OX=9606 GN=NCOA5 PE=1 SV=2	4	0,414	2,943	1,155	1502816,956	543694,4669	399926,5999
Q99714	HSD17B10	3-hydroxyacyl-CoA dehydrogenase type-2 OS=Homo sapiens OX=9606 GN=HSD17B10 PE=1 SV=3	12	1,487	0,783	1,154	40620946,22	57934517,19	49707649,4
P50402	EMD	Emerin OS=Homo sapiens OX=9606 GN=EMD PE=1 SV=1	8	1,725	0,64	1,153	12568515,38	22978883,95	19263262,68
Q13547	HDAC1	Histone deacetylase 1 OS=Homo sapiens OX=9606 GN=HDAC1 PE=1 SV=1	4	0,686	1,852	1,153	3898452,001	2767620,516	3023026,503
P48643	CCT5	T-complex protein 1 subunit epsilon OS=Homo sapiens OX=9606 GN=CCT5 PE=1 SV=1	24	0,606	2,021	1,152	81152608,11	42454205,17	42974487,51
Q96FW1	OTUB1	Ubiquitin thioesterase OTUB1 OS=Homo sapiens OX=9606 GN=OTUB1 PE=1 SV=2	6	0,423	2,875	1,152	14206571,79	5797979,38	4245554,005
Q02818	NUCB1	Nucleobindin-1 OS=Homo sapiens OX=9606 GN=NUCB1 PE=1 SV=4	16	1,109	1,11	1,151	16627196,86	19454085,39	15458359,3
O14656	TOR1A	Torsin-1A OS=Homo sapiens OX=9606 GN=TOR1A PE=1 SV=1	5	2,508	0,423	1,149	2062389,771	4359974,994	4032032,157
P55786	NPEPPS	Puromycin-sensitive aminopeptidase OS=Homo sapiens OX=9606 GN=NPEPPS PE=1 SV=2	25	1,211	0,97	1,149	23528190,53	27461386,36	25728873,08
P68036	UBE2L3	Ubiquitin-conjugating enzyme E2 L3 OS=Homo sapiens OX=9606 GN=UBE2L3 PE=1 SV=1	5	0,567	2,03	1,148	14724866,48	8781259,677	7805607,46
O75822	EIF3J	Eukaryotic translation initiation factor 3 subunit J OS=Homo sapiens OX=9606 GN=EIF3J PE=1 SV	10	0,706	1,743	1,147	13603174,82	11744567,04	7561527,326
O00499	BIN1	Myc box-dependent-interacting protein 1 OS=Homo sapiens OX=9606 GN=BIN1 PE=1 SV=1	3	0,949	1,053	1,146	1086105,442	768799,359	1167527,733
Q2TAL8	QRICH1	Transcriptional regulator QRICH1 OS=Homo sapiens OX=9606 GN=QRICH1 PE=1 SV=1	3	0,686	1,671	1,146	1834397,086	1305378,275	988615,2081
Q99523	SORT1	Sortilin OS=Homo sapiens OX=9606 GN=SORT1 PE=1 SV=3	2	0,291	3,781	1,146	2512646,933	705893,2751	746535,0539
P34059	GALNS	N-acetylgalactosamine-6-sulfatase OS=Homo sapiens OX=9606 GN=GALNS PE=1 SV=1	4	1,845	0,656	1,145	1084223,976	1595473,099	1653001,505
P25789	PSMA4	Proteasome subunit alpha type-4 OS=Homo sapiens OX=9606 GN=PSMA4 PE=1 SV=1	5	1,121	1,044	1,145	16595738,41	18629488,75	14831590,55
Q9NRG0	CHRAC1	Chromatin accessibility complex protein 1 OS=Homo sapiens OX=9606 GN=CHRAC1 PE=1 SV=1	2	0,847	1,138	1,145	1174727,966	1034862,583	1268875,572
O00154	ACOT7	Cytosolic acyl coenzyme A thioester hydrolase OS=Homo sapiens OX=9606 GN=ACOT7 PE=1 SV=	8	0,658	1,838	1,145	18148261,42	9770900,534	10913268,75
P03905	MT-ND4	NADH-ubiquinone oxidoreductase chain 4 OS=Homo sapiens OX=9606 GN=MT-ND4 PE=1 SV=1	3	2,088	0,463	1,144	993424,6789	1798829,037	702432,4005
Q10567	AP1B1	AP-1 complex subunit beta-1 OS=Homo sapiens OX=9606 GN=AP1B1 PE=1 SV=3	10	1,215	1,158	1,143	8086250,973	8718465,165	9592957,524
Q13464	ROCK1	Rho-associated protein kinase 1 OS=Homo sapiens OX=9606 GN=ROCK1 PE=1 SV=1	3	0,823	1,613	1,143	1281246,427	920736,0801	692552,2458
Q9NTZ6	RBM12	RNA-binding protein 12 OS=Homo sapiens OX=9606 GN=RBM12 PE=1 SV=1	9	0,542	2,215	1,142	6958637,519	3294670,762	3114305,834
P08581	MET	Hepatocyte growth factor receptor OS=Homo sapiens OX=9606 GN=MET PE=1 SV=4	3	1000	0,001	1,141		694184,0165	826248,2399
Q96DZ1	ERLEC1	Endoplasmic reticulum lectin 1 OS=Homo sapiens OX=9606 GN=ERLEC1 PE=1 SV=1	4	1,891	0,565	1,141	940361,7918	1611004,01	1540630,714
Q14166	TTLL12	Tubulin--tyrosine ligase-like protein 12 OS=Homo sapiens OX=9606 GN=TTLL12 PE=1 SV=2	3	0,682	1,785	1,141	1976800,292	1287981,984	1158648,015
O00139	KIF2A	Kinesin-like protein KIF2A OS=Homo sapiens OX=9606 GN=KIF2A PE=1 SV=3	6	0,53	2,069	1,141	4242176,517	2323078,09	2090987,914
Q9Y2Q3	GSTK1	Glutathione S-transferase kappa 1 OS=Homo sapiens OX=9606 GN=GSTK1 PE=1 SV=3	8	8,118	0,106	1,14	3575783,596	38710510,89	38564799,91
Q53H12	AGK	Acylglycerol kinase, mitochondrial OS=Homo sapiens OX=9606 GN=AGK PE=1 SV=2	5	0,897	1,253	1,14	6761074,74	5167825,446	4613573,423
P03915	MT-ND5	NADH-ubiquinone oxidoreductase chain 5 OS=Homo sapiens OX=9606 GN=MT-ND5 PE=1 SV=2	3	2,411	0,493	1,139	544960,0912	1648691,003	1211114,557
Q9H3N1	TMX1	Thioredoxin-related transmembrane protein 1 OS=Homo sapiens OX=9606 GN=TMX1 PE=1 SV=1	14	1,356	0,937	1,139	27801474,46	35489785,67	28427631,5
Q4G176	ACSF3	Malonate--CoA ligase ACSF3, mitochondrial OS=Homo sapiens OX=9606 GN=ACSF3 PE=1 SV=3	2	9,348	0,161	1,138	151838,3011	133070,5207	865561,3336
P48147	PREP	Prolyl endopeptidase OS=Homo sapiens OX=9606 GN=PREP PE=1 SV=2	7	0,496	1,885	1,138	3065223,485	1798933,507	1463022,908
Q9UQ80	PA2G4	Proliferation-associated protein 2G4 OS=Homo sapiens OX=9606 GN=PA2G4 PE=1 SV=3	15	0,579	2,134	1,137	49684317,04	28344893,31	22062827,24
P61024	CKS1B	Cyclin-dependent kinases regulatory subunit 1 OS=Homo sapiens OX=9606 GN=CKS1B PE=1 SV=	2	0,405	4,369	1,137	1592465,343	726187,4228	379097,7399
Q03252	LMNB2	Lamin-B2 OS=Homo sapiens OX=9606 GN=LMNB2 PE=1 SV=4	26	1,513	0,763	1,136	64836999,05	96113749,13	84504753,07
Q9BVK6	TMED9	Transmembrane emp24 domain-containing protein 9 OS=Homo sapiens OX=9606 GN=TMED9 PE	8	1,476	0,873	1,134	15896724,71	26595107,88	24848201,62
Q13200	PSMD2	26S proteasome non-ATPase regulatory subunit 2 OS=Homo sapiens OX=9606 GN=PSMD2 PE=1	27	0,644	1,71	1,134	35181434,7	19476392,48	20905521,28
Q16513	PKN2	Serine/threonine-protein kinase N2 OS=Homo sapiens OX=9606 GN=PKN2 PE=1 SV=1	7	0,473	2,914	1,134	2946584,513	1566823,817	1349337,773
Q9UIV1	CNOT7	CCR4-NOT transcription complex subunit 7 OS=Homo sapiens OX=9606 GN=CNOT7 PE=1 SV=3	2	0,669	1,477	1,133	1017670,835	706756,8085	655245,68
Q9BUJ2	HNRNPUL1	Heterogeneous nuclear ribonucleoprotein U-like protein 1 OS=Homo sapiens OX=9606 GN=HNRI	11	1,374	0,77	1,132	9631655,496	11177313,87	14058729,19
Q9Y2Q5	LAMTOR2	Ragulator complex protein LAMTOR2 OS=Homo sapiens OX=9606 GN=LAMTOR2 PE=1 SV=1	4	1,328	0,88	1,132	1240022,176	1599396,896	1310118,957
Q9H061	TMEM126A	Transmembrane protein 126A OS=Homo sapiens OX=9606 GN=TMEM126A PE=1 SV=1	6	1,395	0,897	1,131	1643820,455	3252629,022	2729836,192
Q16795	NDUFA9	NADH dehydrogenase [ubiquinone] 1 alpha subcomplex subunit 9, mitochondrial OS=Homo sapi	14	1,344	0,783	1,131	7494561,885	8578638,74	10865910,54
Q9NR45	NANS	Sialic acid synthase OS=Homo sapiens OX=9606 GN=NANS PE=1 SV=2	4	0,985	1,586	1,131	4086202,393	3055577,532	2990918,124
Q92600	CNOT9	CCR4-NOT transcription complex subunit 9 OS=Homo sapiens OX=9606 GN=CNOT9 PE=1 SV=1	4	0,667	2,081	1,13	1055812,112	933362,7663	909628,7054
Q9UI30	TRMT112	Multifunctional methyltransferase subunit TRM112-like protein OS=Homo sapiens OX=9606 GN=	3	0,373	2,023	1,128	4567896,238	2710408,936	2512076,311
P84103	SRSF3	Serine/arginine-rich splicing factor 3 OS=Homo sapiens OX=9606 GN=SRSF3 PE=1 SV=1	4	0,873	1,728	1,127	13811577,81	9252617,052	24701297,3
Q02750	MAP2K1	Dual specificity mitogen-activated protein kinase kinase 1 OS=Homo sapiens OX=9606 GN=MAP2	3	0,525	0,952	1,127	1187799,601	114233,6649	1143813,385
O75506	HSBP1	Heat shock factor-binding protein 1 OS=Homo sapiens OX=9606 GN=HSBP1 PE=1 SV=1	4	0,603	1,885	1,126	2327146,911	1399579,816	1067861,25
P17987	TCP1	T-complex protein 1 subunit alpha OS=Homo sapiens OX=9606 GN=TCP1 PE=1 SV=1	20	0,701	1,645	1,125	82209394,05	47034197,13	48458274,58
P07199	CENPB	Major centromere autoantigen B OS=Homo sapiens OX=9606 GN=CENPB PE=1 SV=2	4	2,566	0,492	1,124	1269623,422	3181624,856	2516056,175
O60547	GMDS	GDP-mannose 4,6 dehydratase OS=Homo sapiens OX=9606 GN=GMDS PE=1 SV=1	4	2,484	0,346	1,124	3038883,76	189587,7332	739599,8826
P48739	PITPNB	Phosphatidylinositol transfer protein beta isoform OS=Homo sapiens OX=9606 GN=PITPNB PE=1	3	0,525	1,972	1,124	2195351,483	1189228,238	1130272,732
Q9NPD8	UBE2T	Ubiquitin-conjugating enzyme E2 T OS=Homo sapiens OX=9606 GN=UBE2T PE=1 SV=1	3	0,484	2,308	1,124	1393506,393	529063,4997	616375,509
Q460N5	PARP14	Protein mono-ADP-ribosyltransferase PARP14 OS=Homo sapiens OX=9606 GN=PARP14 PE=1 SV=	10	3,581	0,298	1,123	589229,2778	2029524,1	2665322,956
O00116	AGPS	Alkyldihydroxyacetonephosphate synthase, peroxisomal OS=Homo sapiens OX=9606 GN=AGPS P	16	1,325	0,839	1,123	13286068,43	18515563,69	17608731,56
O15382	BCAT2	Branched-chain-amino-acid aminotransferase, mitochondrial OS=Homo sapiens OX=9606 GN=BC	11	9,156	0,029	1,122	13974,9425	1950763,652	1755003,96
O43747	AP1G1	AP-1 complex subunit gamma-1 OS=Homo sapiens OX=9606 GN=AP1G1 PE=1 SV=5	7	1,825	0,776	1,122	4292577,893	6796385,164	5655729,696
P13073	COX4I1	Cytochrome c oxidase subunit 4 isoform 1, mitochondrial OS=Homo sapiens OX=9606 GN=COX4I	7	1	1,088	1,122	45455522,47	53657690,87	53676397,14
Q8NFQ8	TOR1AIP2	Torsin-1A-interacting protein 2 OS=Homo sapiens OX=9606 GN=TOR1AIP2 PE=1 SV=1	6	1,82	0,505	1,119	1141929,747	2194012,306	2883944,242
Q9BTT0	ANP32E	Acidic leucine-rich nuclear phosphoprotein 32 family member E OS=Homo sapiens OX=9606 GN=	5	0,418	2,491	1,119	9438474,383	4645786,796	4319311,45
P60981	DSTN	Destrin OS=Homo sapiens OX=9606 GN=DSTN PE=1 SV=3	4	1,051	2,34	1,118	4679910,617	10389982,28	8120326,475
Q14240	EIF4A2	Eukaryotic initiation factor 4A-II OS=Homo sapiens OX=9606 GN=EIF4A2 PE=1 SV=2	4	1,012	1,126	1,118	2374055,107	2342319,896	2053433,781
P12081	HARS1	Histidine--tRNA ligase, cytoplasmic OS=Homo sapiens OX=9606 GN=HARS1 PE=1 SV=2	7	0,941	1,253	1,117	18159001,91	15767437,3	14404482,39
Q15233	NONO	Non-POU domain-containing octamer-binding protein OS=Homo sapiens OX=9606 GN=NONO PE	19	0,598	2,034	1,116	171473712,6	98067138,56	92433884,23
P20338	RAB4A	Ras-related protein Rab-4A OS=Homo sapiens OX=9606 GN=RAB4A PE=1 SV=3	2	1,054	1,099	1,115	374726,0099	395081,1964	354357,7899
Q13263	TRIM28	Transcription intermediary factor 1-beta OS=Homo sapiens OX=9606 GN=TRIM28 PE=1 SV=5	23	0,889	1,287	1,115	85633654,76	71743346,79	68682509,22
P31153	MAT2A	S-adenosylmethionine synthase isoform type-2 OS=Homo sapiens OX=9606 GN=MAT2A PE=1 SV	8	0,507	2,384	1,115	22017272,02	10973132,58	9999914,894
Q9Y3A5	SBDS	Ribosome maturation protein SBDS OS=Homo sapiens OX=9606 GN=SBDS PE=1 SV=4	2	1,01	1,071	1,114	803806,9404	1837484,945	750784,2202
P47756	CAPZB	F-actin-capping protein subunit beta OS=Homo sapiens OX=9606 GN=CAPZB PE=1 SV=5	11	1,18	0,96	1,113	29491116,81	36890785,69	32469378,96
Q9P032	NDUFAF4	NADH dehydrogenase [ubiquinone] 1 alpha subcomplex assembly factor 4 OS=Homo sapiens OX	6	0,855	2,254	1,113	934896,3462	1113247,842	1079599,274
P62841	RPS15	Small ribosomal subunit protein uS19 OS=Homo sapiens OX=9606 GN=RPS15 PE=1 SV=2	3	0,703	1,741	1,112	8786662,713	6239619,978	5251035,234
Q92769	HDAC2	Histone deacetylase 2 OS=Homo sapiens OX=9606 GN=HDAC2 PE=1 SV=2	4	0,264	3,666	1,112	24681204,82	9079370,914	11227002,36
Q6FI81	CIAPIN1	Anamorsin OS=Homo sapiens OX=9606 GN=CIAPIN1 PE=1 SV=2	5	0,469	3,448	1,111	5329233,936	2191576,112	1641167,25
O14933	UBE2L6	Ubiquitin/ISG15-conjugating enzyme E2 L6 OS=Homo sapiens OX=9606 GN=UBE2L6 PE=1 SV=4	3	4,811	0,243	1,11	485930,4552	2394961,727	2133172,446
Q8TAT6	NPLOC4	Nuclear protein localization protein 4 homolog OS=Homo sapiens OX=9606 GN=NPLOC4 PE=1 SV	8	0,853	1,666	1,11	5256548,986	4797561,526	3101790,355
O95822	MLYCD	Malonyl-CoA decarboxylase, mitochondrial OS=Homo sapiens OX=9606 GN=MLYCD PE=1 SV=3	3	1,443	0,6	1,108	197642,6772	436319,1817	618284,3948
Q52LJ0	FAM98B	Protein FAM98B OS=Homo sapiens OX=9606 GN=FAM98B PE=1 SV=2	4	0,419	2,213	1,108	3092550,524	1157978,959	2056900,008
O94901	SUN1	SUN domain-containing protein 1 OS=Homo sapiens OX=9606 GN=SUN1 PE=1 SV=4	8	3,197	0,348	1,107	959612,0599	4422756,068	6484784,098
Q13287	NMI	N-myc-interactor OS=Homo sapiens OX=9606 GN=NMI PE=1 SV=2	4	1000	0,001	1,106		2827823,718	2419997,228
Q9Y305	ACOT9	Acyl-coenzyme A thioesterase 9, mitochondrial OS=Homo sapiens OX=9606 GN=ACOT9 PE=1 SV=	13	2,27	0,531	1,106	5012389,881	11770701,1	10135542,57
P50990	CCT8	T-complex protein 1 subunit theta OS=Homo sapiens OX=9606 GN=CCT8 PE=1 SV=4	23	0,584	1,914	1,106	131660251,9	69902905,06	65173021,07
Q92520	FAM3C	Protein FAM3C OS=Homo sapiens OX=9606 GN=FAM3C PE=1 SV=1	6	2,077	0,492	1,105	5385856,566	8139006,725	11655519,64
Q7Z3K3	POGZ	Pogo transposable element with ZNF domain OS=Homo sapiens OX=9606 GN=POGZ PE=1 SV=2	8	0,549	1,893	1,105	3527171,009	1850393,138	1584640,951
Q96DA6	DNAJC19	Mitochondrial import inner membrane translocase subunit TIM14 OS=Homo sapiens OX=9606 G	2	1,863	0,534	1,103	954579,0824	960343,6623	1789671,081
P27635	RPL10	Large ribosomal subunit protein uL16 OS=Homo sapiens OX=9606 GN=RPL10 PE=1 SV=5	8	0,488	3,342	1,102	25548237,97	10955732,65	11291979,33
O60934	NBN	Nibrin OS=Homo sapiens OX=9606 GN=NBN PE=1 SV=1	3	2,305	0,449	1,101	345864,3148	789707,3054	763342,2923
Q5VT52	RPRD2	Regulation of nuclear pre-mRNA domain-containing protein 2 OS=Homo sapiens OX=9606 GN=R	3	0,958	0,704	1,101	808938,2239	914308,0272	918777,3785
Q6Y7W6	GIGYF2	GRB10-interacting GYF protein 2 OS=Homo sapiens OX=9606 GN=GIGYF2 PE=1 SV=1	3	0,688	1,631	1,101	625024,7646	449862,9387	397956,5025
Q969G3	SMARCE1	SWI/SNF-related matrix-associated actin-dependent regulator of chromatin subfamily E member	8	0,562	1,853	1,101	8944915,046	4710492,829	4209843,213
P0CG47	UBB	Polyubiquitin-B OS=Homo sapiens OX=9606 GN=UBB PE=1 SV=1	4	0,856	1,47	1,1	228889869,5	197467003,5	163688989,4
Q8IXQ6	PARP9	Protein mono-ADP-ribosyltransferase PARP9 OS=Homo sapiens OX=9606 GN=PARP9 PE=1 SV=2	5	5,744	0,186	1,099	405178,8756	1087762,678	1041192,98
P49368	CCT3	T-complex protein 1 subunit gamma OS=Homo sapiens OX=9606 GN=CCT3 PE=1 SV=4	25	0,709	1,921	1,099	106964725,4	70228416,1	65082111,48
P43243	MATR3	Matrin-3 OS=Homo sapiens OX=9606 GN=MATR3 PE=1 SV=2	26	0,647	1,579	1,099	180368491,8	85771177,16	97392232,38
P20810	CAST	Calpastatin OS=Homo sapiens OX=9606 GN=CAST PE=1 SV=4	9	3,399	0,331	1,097	2625193,296	9102283,44	8342735,989
P09543	CNP	2',3'-cyclic-nucleotide 3'-phosphodiesterase OS=Homo sapiens OX=9606 GN=CNP PE=1 SV=2	8	1,791	0,61	1,097	7519911,4	10738494,16	13140277,65
P60510	PPP4C	Serine/threonine-protein phosphatase 4 catalytic subunit OS=Homo sapiens OX=9606 GN=PPP4	4	0,797	1,399	1,097	2163636,726	1021434,508	1636805,184
P17480	UBTF	Nucleolar transcription factor 1 OS=Homo sapiens OX=9606 GN=UBTF PE=1 SV=1	9	0,529	1,574	1,097	5709797,292	4410238,004	4535103,977
Q9Y3P9	RABGAP1	Rab GTPase-activating protein 1 OS=Homo sapiens OX=9606 GN=RABGAP1 PE=1 SV=3	2	0,484	0,587	1,097	133765,022	1220990,43	587047,0449
Q92796	DLG3	Disks large homolog 3 OS=Homo sapiens OX=9606 GN=DLG3 PE=1 SV=2	4	0,426	2,666	1,097	2788635,261	1042761,197	886699,1804
O75131	CPNE3	Copine-3 OS=Homo sapiens OX=9606 GN=CPNE3 PE=1 SV=1	6	1,015	1,162	1,095	4952752,014	4925461,649	5552240,867
Q16531	DDB1	DNA damage-binding protein 1 OS=Homo sapiens OX=9606 GN=DDB1 PE=1 SV=1	23	0,718	1,53	1,095	25451345,18	16985118,07	18680560,03
Q15084	PDIA6	Protein disulfide-isomerase A6 OS=Homo sapiens OX=9606 GN=PDIA6 PE=1 SV=1	15	0,657	1,714	1,095	222352193,3	124368444	125911756,2
A6NHR9	SMCHD1	Structural maintenance of chromosomes flexible hinge domain-containing protein 1 OS=Homo s	18	1,224	0,944	1,093	8385958,677	8118845,368	9200862,308
Q8IY67	RAVER1	Ribonucleoprotein PTB-binding 1 OS=Homo sapiens OX=9606 GN=RAVER1 PE=1 SV=1	6	1,284	1,021	1,092	2036942,113	2744826,019	2677525,432
P12955	PEPD	Xaa-Pro dipeptidase OS=Homo sapiens OX=9606 GN=PEPD PE=1 SV=3	5	0,709	2,374	1,092	4373066,606	2202535,266	2587946,583
P40121	CAPG	Macrophage-capping protein OS=Homo sapiens OX=9606 GN=CAPG PE=1 SV=2	8	6,594	0,2	1,091	3115018,989	20563435,71	19217330,72
P21796	VDAC1	Voltage-dependent anion-selective channel protein 1 OS=Homo sapiens OX=9606 GN=VDAC1 PE	13	1,855	0,631	1,091	87320413,07	147387731,7	151282840,9
Q8NCA5	FAM98A	Protein FAM98A OS=Homo sapiens OX=9606 GN=FAM98A PE=1 SV=2	3	1,149	0,726	1,091	2071172,074	2424211,301	2365505,939

Q9HCE1	MOV10	Helicase MOV-10 OS=Homo sapiens OX=9606 GN=MOV10 PE=1 SV=2	8	0,817	1,423	1,091	3781640,905	2309741,642	2493133,993
A0A6Q8JFT6	PABPC1L	Polyadenylate-binding protein OS=Homo sapiens OX=9606 GN=PABPC1L PE=1 SV=1	2	0,805	1,382	1,089	21648947,5	15942863,85	15149403,3
Q8NI27	THOC2	THO complex subunit 2 OS=Homo sapiens OX=9606 GN=THOC2 PE=1 SV=2	8	0,696	1,463	1,089	2814847,482	1746552,495	1882796,192
Q9UKA9	PTBP2	Polypyrimidine tract-binding protein 2 OS=Homo sapiens OX=9606 GN=PTBP2 PE=1 SV=1	11	0,06	14,792	1,089	20174180,75	932619,2201	743084,1903
P39687	ANP32A	Acidic leucine-rich nuclear phosphoprotein 32 family member A OS=Homo sapiens OX=9606 GN=	4	0,497	2,351	1,088	59143946,61	30199773,22	28406268,73
P47897	QARS1	Glutamine--tRNA ligase OS=Homo sapiens OX=9606 GN=QARS1 PE=1 SV=1	24	0,736	1,578	1,087	29925824,46	16627094,72	20984660,25
Q8WWM7	ATXN2L	Ataxin-2-like protein OS=Homo sapiens OX=9606 GN=ATXN2L PE=1 SV=2	10	0,88	1,167	1,086	7316973,969	6244015,258	6026184,028
P50750	CDK9	Cyclin-dependent kinase 9 OS=Homo sapiens OX=9606 GN=CDK9 PE=1 SV=3	5	0,615	1,975	1,086	1611684,068	1361670,649	1202547,851
P62820	RAB1A	Ras-related protein Rab-1A OS=Homo sapiens OX=9606 GN=RAB1A PE=1 SV=3	4	1,216	0,962	1,084	24027902,28	26823215,6	30117607,56
P31946	YWHAB	14-3-3 protein beta/alpha OS=Homo sapiens OX=9606 GN=YWHAB PE=1 SV=3	6	1,055	0,961	1,084	51156629,67	59061572,58	53202788,52
Q13098	GPS1	COP9 signalosome complex subunit 1 OS=Homo sapiens OX=9606 GN=GPS1 PE=1 SV=4	7	0,632	1,687	1,084	5336728,438	3648602,855	2945726,429
O43488	AKR7A2	Aflatoxin B1 aldehyde reductase member 2 OS=Homo sapiens OX=9606 GN=AKR7A2 PE=1 SV=3	4	0,496	2,091	1,083	3666114,624	1821512,176	1694323,629
P61204	ARF3	ADP-ribosylation factor 3 OS=Homo sapiens OX=9606 GN=ARF3 PE=1 SV=2	4	1,033	1,279	1,082	23436966,31	18126255,05	17522256,51
P61289	PSME3	Proteasome activator complex subunit 3 OS=Homo sapiens OX=9606 GN=PSME3 PE=1 SV=1	9	0,915	1,107	1,082	13273135,91	10750653,58	11228233,04
Q9NRV9	HEBP1	Heme-binding protein 1 OS=Homo sapiens OX=9606 GN=HEBP1 PE=1 SV=1	6	0,765	1,494	1,082	3569995,574	4059364,132	3052973,409
Q16643	DBN1	Drebrin OS=Homo sapiens OX=9606 GN=DBN1 PE=1 SV=4	17	0,341	3,295	1,082	88922988,8	29799651,54	25754371,18
Q16851	UGP2	UTP--glucose-1-phosphate uridylyltransferase OS=Homo sapiens OX=9606 GN=UGP2 PE=1 SV=5	13	0,528	1,904	1,079	13648106,04	7442282,464	7370796,89
Q6P1L8	MRPL14	Large ribosomal subunit protein uL14m OS=Homo sapiens OX=9606 GN=MRPL14 PE=1 SV=1	3	1,56	0,658	1,078	869348,3654	1261712,538	1279035,597
P54578	USP14	Ubiquitin carboxyl-terminal hydrolase 14 OS=Homo sapiens OX=9606 GN=USP14 PE=1 SV=3	14	0,755	1,742	1,078	11540654,99	6432328,706	6317412,909
Q9UMX0	UBQLN1	Ubiquilin-1 OS=Homo sapiens OX=9606 GN=UBQLN1 PE=1 SV=2	4	0,999	1,086	1,077	11071993,98	9676013,635	10393826,5
P51858	HDGF	Hepatoma-derived growth factor OS=Homo sapiens OX=9606 GN=HDGF PE=1 SV=1	11	0,784	1,655	1,077	22734353,11	16916576,81	13787099,44
Q8ND30	PPFIBP2	Liprin-beta-2 OS=Homo sapiens OX=9606 GN=PPFIBP2 PE=1 SV=3	10	1,612	0,643	1,076	66301,78414	106876,8149	175846,8029
P55010	EIF5	Eukaryotic translation initiation factor 5 OS=Homo sapiens OX=9606 GN=EIF5 PE=1 SV=2	10	0,573	1,816	1,076	13679308,36	6942491,426	6299087,416
Q14118	DAG1	Dystroglycan 1 OS=Homo sapiens OX=9606 GN=DAG1 PE=1 SV=2	3	1,28	0,881	1,075	1059791,749	1320868,757	1357441,34
P09382	LGALS1	Galectin-1 OS=Homo sapiens OX=9606 GN=LGALS1 PE=1 SV=2	4	36,459	0,053	1,074	6595066,946	124346214,3	128160500,6
O95169	NDUFB8	NADH dehydrogenase [ubiquinone] 1 beta subcomplex subunit 8, mitochondrial OS=Homo sapie	4	1,884	0,513	1,074	1071771,483	2149637,899	5487073,358
O43674	NDUFB5	NADH dehydrogenase [ubiquinone] 1 beta subcomplex subunit 5, mitochondrial OS=Homo sapie	5	1,46	0,95	1,074	768162,3313	1993032,896	2415080,096
O15160	POLR1C	DNA-directed RNA polymerases I and III subunit RPAC1 OS=Homo sapiens OX=9606 GN=POLR1C	5	0,746	1,587	1,074	2585836,633	1766308,912	1431477,315
O95168	NDUFB4	NADH dehydrogenase [ubiquinone] 1 beta subcomplex subunit 4 OS=Homo sapiens OX=9606 GN	5	1,738	0,504	1,073	4053829,534	6923361,599	8915925,262
Q14202	ZMYM3	Zinc finger MYM-type protein 3 OS=Homo sapiens OX=9606 GN=ZMYM3 PE=1 SV=2	5	0,681	1,774	1,073	1659293,062	1211140,559	853675,8025
P61158	ACTR3	Actin-related protein 3 OS=Homo sapiens OX=9606 GN=ACTR3 PE=1 SV=3	11	1,495	0,631	1,072	13531865,46	20890478,04	21581649,18
P11586	MTHFD1	C-1-tetrahydrofolate synthase, cytoplasmic OS=Homo sapiens OX=9606 GN=MTHFD1 PE=1 SV=4	26	0,732	1,534	1,072	45864226,56	28186759,05	30001330,7
Q9H4G0	EPB41L1	Band 4.1-like protein 1 OS=Homo sapiens OX=9606 GN=EPB41L1 PE=1 SV=2	16	2,932	0,262	1,071	839501,8663	14275688,94	14665666,33
Q9GZT8	NIF3L1	NIF3-like protein 1 OS=Homo sapiens OX=9606 GN=NIF3L1 PE=1 SV=2	3	0,32	4,726	1,071	1415170,865	309775,0273	341546,8168
O00151	PDLIM1	PDZ and LIM domain protein 1 OS=Homo sapiens OX=9606 GN=PDLIM1 PE=1 SV=4	11	4,019	0,279	1,07	10961376,61	54934853,31	52177279,41
P01111	NRAS	GTPase NRas OS=Homo sapiens OX=9606 GN=NRAS PE=1 SV=1	7	1,077	0,991	1,07	7141566,564	7197714,613	8636489,567
O43396	TXNL1	Thioredoxin-like protein 1 OS=Homo sapiens OX=9606 GN=TXNL1 PE=1 SV=3	6	0,79	1,29	1,07	5265997,938	4231819,877	3992265,779
Q9Y3A3	MOB4	MOB-like protein phocein OS=Homo sapiens OX=9606 GN=MOB4 PE=1 SV=1	2	1,02	0,909	1,067	679554,1268	801165,9688	688489,1283
Q96A57	TMEM230	Transmembrane protein 230 OS=Homo sapiens OX=9606 GN=TMEM230 PE=1 SV=1	2	3,006	0,35	1,066	1508441,533	2784860,528	2597353,367
Q96D53	COQ8B	Atypical kinase COQ8B, mitochondrial OS=Homo sapiens OX=9606 GN=COQ8B PE=1 SV=2	2	0,885	1,086	1,066	290416,2478	233250,4614	238527,259
P84090	ERH	Enhancer of rudimentary homolog OS=Homo sapiens OX=9606 GN=ERH PE=1 SV=1	5	0,801	1,301	1,066	18445550,37	14107587,75	13406055,41
P17980	PSMC3	26S proteasome regulatory subunit 6A OS=Homo sapiens OX=9606 GN=PSMC3 PE=1 SV=3	17	0,66	1,474	1,066	24374550,42	17653559,36	17656960,39
Q14151	SAFB2	Scaffold attachment factor B2 OS=Homo sapiens OX=9606 GN=SAFB2 PE=1 SV=1	7	0,605	1,563	1,066	10599819,78	5282163,247	6041232,269
Q14257	RCN2	Reticulocalbin-2 OS=Homo sapiens OX=9606 GN=RCN2 PE=1 SV=1	11	0,264	4,063	1,066	38782472,6	10035662,9	10364361,61
O95249	GOSR1	Golgi SNAP receptor complex member 1 OS=Homo sapiens OX=9606 GN=GOSR1 PE=1 SV=1	3	3,289	0,364	1,065	455277,3653	1497325,324	1234048,565
P13797	PLS3	Plastin-3 OS=Homo sapiens OX=9606 GN=PLS3 PE=1 SV=4	10	2,603	0,43	1,065	9543651,37	22777773,24	22326558,93
O95777	LSM8	U6 snRNA-associated Sm-like protein LSm8 OS=Homo sapiens OX=9606 GN=LSM8 PE=1 SV=3	5	0,586	1,831	1,065	12761252,41	7618878,274	7921353,286
Q9NUQ2	AGPAT5	1-acyl-sn-glycerol-3-phosphate acyltransferase epsilon OS=Homo sapiens OX=9606 GN=AGPAT5	3	0,807	0,5	1,064	297677,3309	361890,2968	136147,57
Q02790	FKBP4	Peptidyl-prolyl cis-trans isomerase FKBP4 OS=Homo sapiens OX=9606 GN=FKBP4 PE=1 SV=3	20	0,333	3,347	1,063	37501031,68	10537582,41	11044548,05
Q9Y2D4	EXOC6B	Exocyst complex component 6B OS=Homo sapiens OX=9606 GN=EXOC6B PE=1 SV=3	3	1,003	0,789	1,062	357934,1337	469675,5713	632365,6512
P05387	RPLP2	Large ribosomal subunit protein P2 OS=Homo sapiens OX=9606 GN=RPLP2 PE=1 SV=1	7	0,885	1,063	1,062	100192025,7	89575323,72	101750710,8
Q9BSD7	NTPCR	Cancer-related nucleoside-triphosphatase OS=Homo sapiens OX=9606 GN=NTPCR PE=1 SV=1	2	0,617	1,628	1,062	852063,9442	543702,5057	486036,8962
Q5TBB1	RNASEH2B	Ribonuclease H2 subunit B OS=Homo sapiens OX=9606 GN=RNASEH2B PE=1 SV=1	2	0,498	2,009	1,062	645683,6034	401599,3915	365087,0564
P08240	SRPRA	Signal recognition particle receptor subunit alpha OS=Homo sapiens OX=9606 GN=SRPRA PE=1 S	9	0,547	1,343	1,061	4060494,865	2398917,678	2722643,14
Q8N766	EMC1	ER membrane protein complex subunit 1 OS=Homo sapiens OX=9606 GN=EMC1 PE=1 SV=1	16	1,563	0,633	1,059	6163918,682	10933408,03	8888554,707
P06576	ATP5F1B	ATP synthase subunit beta, mitochondrial OS=Homo sapiens OX=9606 GN=ATP5F1B PE=1 SV=3	26	1,368	0,77	1,059	201001649,3	220010484,3	263698002,6
P43487	RANBP1	Ran-specific GTPase-activating protein OS=Homo sapiens OX=9606 GN=RANBP1 PE=1 SV=1	6	0,284	2,975	1,059	48529489,08	16160744,57	15517883,66
Q9H0P0	NT5C3A	Cytosolic 5'-nucleotidase 3A OS=Homo sapiens OX=9606 GN=NT5C3A PE=1 SV=3	2	1,921	0,606	1,058	630719,7006	1950267,302	1806630,455
O16718	NDUFA5	NADH dehydrogenase [ubiquinone] 1 alpha subcomplex subunit 5 OS=Homo sapiens OX=9606 G	5	1,682	0,607	1,058	3537945,283	6385924,667	6079676,445
Q8IYU8	MICU2	Calcium uptake protein 2, mitochondrial OS=Homo sapiens OX=9606 GN=MICU2 PE=1 SV=2	2	2,296	0,461	1,057	360099,1658	1336779,224	1250326,789
A8MWD9	SNRPGP15	Putative small nuclear ribonucleoprotein G-like protein 15 OS=Homo sapiens OX=9606 GN=SNRF	2	0,584	2,089	1,057	20843478,3	10833975,38	9889947,868
P62314	SNRPD1	Small nuclear ribonucleoprotein Sm D1 OS=Homo sapiens OX=9606 GN=SNRPD1 PE=1 SV=1	5	0,739	1,252	1,055	36439024,59	25375969,11	32838786,07
Q96N67	DOCK7	Dedicator of cytokinesis protein 7 OS=Homo sapiens OX=9606 GN=DOCK7 PE=1 SV=4	5	0,449	1,707	1,054	4471083,309	1740982,099	1817489,84
P12694	BCKDHA	2-oxoisovalerate dehydrogenase subunit alpha, mitochondrial OS=Homo sapiens OX=9606 GN=B	3	1,472	0,596	1,053	493947,3077	708185,2372	862216,796
Q14157	UBAP2L	Ubiquitin-associated protein 2-like OS=Homo sapiens OX=9606 GN=UBAP2L PE=1 SV=2	10	0,844	1,27	1,053	6495563,418	6510311,372	5042866,839
P60953	CDC42	Cell division control protein 42 homolog OS=Homo sapiens OX=9606 GN=CDC42 PE=1 SV=2	3	1,534	0,65	1,052	4118006,873	6495949,842	7939428,414
Q9NPH2	ISYNA1	Inositol-3-phosphate synthase 1 OS=Homo sapiens OX=9606 GN=ISYNA1 PE=1 SV=1	4	0,195	7,113	1,052	6421148,114	1037308,94	998962,3311
Q9HA77	CARS2	Probable cysteine--tRNA ligase, mitochondrial OS=Homo sapiens OX=9606 GN=CARS2 PE=1 SV=1	2	1,912	0,543	1,051	516655,4665	986939,4813	932722,3468
P36776	LONP1	Lon protease homolog, mitochondrial OS=Homo sapiens OX=9606 GN=LONP1 PE=1 SV=2	19	1,32	0,792	1,051	9831023,621	12344660,22	12777974,71
Q9H4M9	EHD1	EH domain-containing protein 1 OS=Homo sapiens OX=9606 GN=EHD1 PE=1 SV=2	15	2,783	0,403	1,05	6759455,171	23295622,35	14362302,48
O95782	AP2A1	AP-2 complex subunit alpha-1 OS=Homo sapiens OX=9606 GN=AP2A1 PE=1 SV=3	13	2,161	0,516	1,05	8738579,383	17671760,57	19464146,58
Q15006	EMC2	ER membrane protein complex subunit 2 OS=Homo sapiens OX=9606 GN=EMC2 PE=1 SV=1	6	2,084	0,514	1,05	2229617,008	7255373,675	7453144,181
Q96L21	RPL10L	Ribosomal protein uL16-like OS=Homo sapiens OX=9606 GN=RPL10L PE=1 SV=3	3	0,135	7,996	1,05	9759708,847	1067410,311	1834668,302
P56385	ATP5ME	ATP synthase subunit e, mitochondrial OS=Homo sapiens OX=9606 GN=ATP5ME PE=1 SV=2	3	1,454	0,706	1,049	6665700,504	9870909,896	10766621,76
Q14498	RBM39	RNA-binding protein 39 OS=Homo sapiens OX=9606 GN=RBM39 PE=1 SV=2	8	0,585	1,085	1,049	12280172,72	14520345,73	14262450,08
P05023	ATP1A1	Sodium/potassium-transporting ATPase subunit alpha-1 OS=Homo sapiens OX=9606 GN=ATP1A1	35	1,509	0,755	1,048	75790417,85	109236956,9	99295717,27
O95831	AIFM1	Apoptosis-inducing factor 1, mitochondrial OS=Homo sapiens OX=9606 GN=AIFM1 PE=1 SV=1	15	1,258	0,895	1,048	22701871,73	26910049,22	24882529,51
Q9H8Y8	GORASP2	Golgi reassembly-stacking protein 2 OS=Homo sapiens OX=9606 GN=GORASP2 PE=1 SV=3	5	1,033	0,96	1,048	3905902,606	3765201,278	3957613,991
Q9NPJ3	ACOT13	Acyl-coenzyme A thioesterase 13 OS=Homo sapiens OX=9606 GN=ACOT13 PE=1 SV=1	3	1,479	0,727	1,047	4631124,238	6434502,721	6338216,124
Q7L576	CYFIP1	Cytoplasmic FMR1-interacting protein 1 OS=Homo sapiens OX=9606 GN=CYFIP1 PE=1 SV=1	12	1,022	0,984	1,047	3903446,887	4074778,724	4750108,191
Q9Y221	NIP7	60S ribosome subunit biogenesis protein NIP7 homolog OS=Homo sapiens OX=9606 GN=NIP7 PE	3	0,418	2,552	1,047	1176944,219	579686,794	278666,7928
O43920	NDUFS5	NADH dehydrogenase [ubiquinone] iron-sulfur protein 5 OS=Homo sapiens OX=9606 GN=NDUFS	2	1,839	0,633	1,046	1634476,868	3088519,791	2864718,901
Q9H2M9	RAB3GAP2	Rab3 GTPase-activating protein non-catalytic subunit OS=Homo sapiens OX=9606 GN=RAB3GAP	5	0,947	1,186	1,046	1414595,882	1835400,156	1600882,857
Q96A65	EXOC4	Exocyst complex component 4 OS=Homo sapiens OX=9606 GN=EXOC4 PE=1 SV=1	3	1,406	0,764	1,045	588485,852	869523,3223	1416903,089
Q93009	USP7	Ubiquitin carboxyl-terminal hydrolase 7 OS=Homo sapiens OX=9606 GN=USP7 PE=1 SV=2	12	1,111	0,953	1,044	3813066,639	4138812,12	3881541,35
P35244	RPA3	Replication protein A 14 kDa subunit OS=Homo sapiens OX=9606 GN=RPA3 PE=1 SV=1	2	0,635	1,594	1,044	4234838,908	3112275,262	2140909,336
Q96AG4	LRRC59	Leucine-rich repeat-containing protein 59 OS=Homo sapiens OX=9606 GN=LRRC59 PE=1 SV=1	6	1,529	0,768	1,043	18621790,14	27165206,46	26176152,95
O75380	NDUFS6	NADH dehydrogenase [ubiquinone] iron-sulfur protein 6, mitochondrial OS=Homo sapiens OX=9	4	0,76	1,225	1,043	2632959,687	1676819,164	2033854,043
P41567	EIF1	Eukaryotic translation initiation factor 1 OS=Homo sapiens OX=9606 GN=EIF1 PE=1 SV=1	2	0,855	1,207	1,042	5090280,579	4275209,788	3493852,418
Q13242	SRSF9	Serine/arginine-rich splicing factor 9 OS=Homo sapiens OX=9606 GN=SRSF9 PE=1 SV=1	8	0,448	2,1	1,041	24520913,05	10185186,84	9723541,305
P45973	CBX5	Chromobox protein homolog 5 OS=Homo sapiens OX=9606 GN=CBX5 PE=1 SV=1	6	0,222	3,491	1,041	7200751,372	1705074,564	1937754,289
Q8WW59	SPRYD4	SPRY domain-containing protein 4 OS=Homo sapiens OX=9606 GN=SPRYD4 PE=1 SV=3	3	1,558	0,654	1,04	398582,2868	573484,4262	602364,4873
P53041	PPP5C	Serine/threonine-protein phosphatase 5 OS=Homo sapiens OX=9606 GN=PPP5C PE=1 SV=1	4	0,638	1,775	1,04	1615813,985	1157714,965	1159564,316
P09429	HMGB1	High mobility group protein B1 OS=Homo sapiens OX=9606 GN=HMGB1 PE=1 SV=3	8	0,404	2,567	1,04	92459254,73	32935895,37	32279655,71
O75410	TACC1	Transforming acidic coiled-coil-containing protein 1 OS=Homo sapiens OX=9606 GN=TACC1 PE=1	2	1000	0,001	1,039		214875,1857	206801,5183
O43676	NDUFB3	NADH dehydrogenase [ubiquinone] 1 beta subcomplex subunit 3 OS=Homo sapiens OX=9606 GN	3	2,021	0,458	1,039	2427474,364	5189640,012	5036878,821
P63241	EIF5A	Eukaryotic translation initiation factor 5A-1 OS=Homo sapiens OX=9606 GN=EIF5A PE=1 SV=2	9	0,27	3,008	1,039	54264099	9130148,273	23534200,54
Q16656	NRF1	Nuclear respiratory factor 1 OS=Homo sapiens OX=9606 GN=NRF1 PE=1 SV=1	2	3,159	0,726	1,038	442534,6512	530281,1314	551522,3043
P49757	NUMB	Protein numb homolog OS=Homo sapiens OX=9606 GN=NUMB PE=1 SV=2	3	1,855	0,55	1,037	460596,2937	854612,2785	821521,1106
P63010	AP2B1	AP-2 complex subunit beta OS=Homo sapiens OX=9606 GN=AP2B1 PE=1 SV=1	17	1,585	0,636	1,037	32185758,55	46427913,91	47229516,98
P11940	PABPC1	Polyadenylate-binding protein 1 OS=Homo sapiens OX=9606 GN=PABPC1 PE=1 SV=2	16	0,724	1,447	1,037	131441443,5	86091029,54	86401326,88
E9PAV3	NACA	Nascent polypeptide-associated complex subunit alpha, muscle-specific form OS=Homo sapiens	5	0,357	2,668	1,036	28071345,87	6669857,91	9722150,977
Q92599	SEPTIN8	Septin-8 OS=Homo sapiens OX=9606 GN=SEPTIN8 PE=1 SV=4	7	1,532	0,753	1,035	2681472,762	4236064,496	4687947,655
Q92888	ARHGEF1	Rho guanine nucleotide exchange factor 1 OS=Homo sapiens OX=9606 GN=ARHGEF1 PE=1 SV=2	6	1,501	0,755	1,035	1417500,055	2035116,13	985827,5074
P05388	RPLP0	Large ribosomal subunit protein uL10 OS=Homo sapiens OX=9606 GN=RPLP0 PE=1 SV=1	10	0,765	1,389	1,035	90826509,77	65026593,61	73672304,94
Q9NVZ3	NECAP2	Adaptin ear-binding coat-associated protein 2 OS=Homo sapiens OX=9606 GN=NECAP2 PE=1 SV=	2	0,599	1,729	1,035	998553,0183	624722,079	609720,051
P40227	CCT6A	T-complex protein 1 subunit zeta OS=Homo sapiens OX=9606 GN=CCT6A PE=1 SV=3	18	0,648	1,537	1,034	79033410,5	44105303,17	44876119,77
Q9P0L0	VAPA	Vesicle-associated membrane protein-associated protein A OS=Homo sapiens OX=9606 GN=VAP	7	1,798	0,729	1,033	14386241,37	17473460,75	21152386,48
O94842	TOX4	TOX high mobility group box family member 4 OS=Homo sapiens OX=9606 GN=TOX4 PE=1 SV=1	3	0,946	1,407	1,033	1641639,506	1266541,416	835816,1097
P82979	SARNP	SAP domain-containing ribonucleoprotein OS=Homo sapiens OX=9606 GN=SARNP PE=1 SV=3	7	0,749	1,375	1,033	23656704,89	18334470	20173739,86
Q9Y224	RTRAF	RNA transcription, translation and transport factor protein OS=Homo sapiens OX=9606 GN=RTRA	8	0,633	1,793	1,033	14017482,01	7913784,322	7218223,193

O15439	ABCC4	ATP-binding cassette sub-family C member 4 OS=Homo sapiens OX=9606 GN=ABCC4 PE=1 SV=3	12	2,572	0,331	1,032	147433,2785	989610,4934	905274,3937
Q7Z3C6	ATG9A	Autophagy-related protein 9A OS=Homo sapiens OX=9606 GN=ATG9A PE=1 SV=3	3	1,409	0,825	1,032	276889,6089	461798,5089	479593,8172
Q8N6T3	ARFGAP1	ADP-ribosylation factor GTPase-activating protein 1 OS=Homo sapiens OX=9606 GN=ARFGAP1 P	7	1,231	0,818	1,031	3347044,753	3892022,397	3661403,715
O14979	HNRNPDL	Heterogeneous nuclear ribonucleoprotein D-like OS=Homo sapiens OX=9606 GN=HNRNPDL PE=	6	1,131	0,874	1,03	21662169,76	28972629,69	31405619,42
Q7Z4V5	HDGFL2	Hepatoma-derived growth factor-related protein 2 OS=Homo sapiens OX=9606 GN=HDGFL2 PE=	2	1,033	1,881	1,03	4561933,497	8913832,262	6114383,952
P48634	PRRC2A	Protein PRRC2A OS=Homo sapiens OX=9606 GN=PRRC2A PE=1 SV=3	3	0,554	1,779	1,03	889600,7146	514784,4547	522030,598
Q9NW64	RBM22	Pre-mRNA-splicing factor RBM22 OS=Homo sapiens OX=9606 GN=RBM22 PE=1 SV=1	3	0,817	1,259	1,029	827633,631	228788,0866	217673,2946
P68402	PAFAH1B2	Platelet-activating factor acetylhydrolase IB subunit alpha2 OS=Homo sapiens OX=9606 GN=PAF	4	0,347	2,869	1,029	5969587,263	2356726,954	2083154,528
Q15149	PLEC	Plectin OS=Homo sapiens OX=9606 GN=PLEC PE=1 SV=3	158	3,298	0,359	1,028	47479186,36	158320669,3	165116707,3
Q9UH99	SUN2	SUN domain-containing protein 2 OS=Homo sapiens OX=9606 GN=SUN2 PE=1 SV=3	16	2,362	0,438	1,027	2460655,14	7006580,3	7114603,172
Q9UNP9	PPIE	Peptidyl-prolyl cis-trans isomerase E OS=Homo sapiens OX=9606 GN=PPIE PE=1 SV=1	4	0,949	1,258	1,027	2638968,263	1677279,457	2804651,145
Q00341	HDLBP	Vigilin OS=Homo sapiens OX=9606 GN=HDLBP PE=1 SV=3	30	0,977	1,166	1,026	27569726,67	20203267,66	20793413,15
Q5JWF2	GNAS	Guanine nucleotide-binding protein G(s) subunit alpha isoforms XLas OS=Homo sapiens OX=960	8	1,297	0,836	1,025	22818506,65	33567667,5	27107382,58
P17813	ENG	Endoglin OS=Homo sapiens OX=9606 GN=ENG PE=1 SV=2	2	2,865	0,362	1,024	595565,2044	2327094,019	1621499,412
Q9NQR4	NIT2	Omega-amidase NIT2 OS=Homo sapiens OX=9606 GN=NIT2 PE=1 SV=1	4	0,752	1,613	1,023	3685448,789	3513088,318	2276963,156
P07814	EPRS1	Bifunctional glutamate/proline--tRNA ligase OS=Homo sapiens OX=9606 GN=EPRS1 PE=1 SV=5	29	0,735	1,421	1,022	29420763,89	23622220,65	25240380,2
P53990	IST1	IST1 homolog OS=Homo sapiens OX=9606 GN=IST1 PE=1 SV=1	3	0,731	1,517	1,022	2375056,864	1579739,656	1595298,501
Q9BT78	COPS4	COP9 signalosome complex subunit 4 OS=Homo sapiens OX=9606 GN=COPS4 PE=1 SV=1	5	0,729	1,386	1,022	4741817,778	2827171,771	2740570,947
Q6UXN9	WDR82	WD repeat-containing protein 82 OS=Homo sapiens OX=9606 GN=WDR82 PE=1 SV=1	6	0,673	2,057	1,022	3718298,432	1665413,675	3259252,671
O43399	TPD52L2	Tumor protein D54 OS=Homo sapiens OX=9606 GN=TPD52L2 PE=1 SV=2	8	1,933	0,567	1,02	15212780,47	27305646,64	27353000,96
Q92905	COPS5	COP9 signalosome complex subunit 5 OS=Homo sapiens OX=9606 GN=COPS5 PE=1 SV=4	3	0,765	1,34	1,02	2070598,433	1626367,217	1520473,518
P80404	ABAT	4-aminobutyrate aminotransferase, mitochondrial OS=Homo sapiens OX=9606 GN=ABAT PE=1 S	5	0,684	0,536	1,02	1636485,164	1039495,794	974643,2441
P61086	UBE2K	Ubiquitin-conjugating enzyme E2 K OS=Homo sapiens OX=9606 GN=UBE2K PE=1 SV=3	4	0,718	1,462	1,018	7813619,13	5198710,357	5210454,226
Q9Y3E5	PTRH2	Peptidyl-tRNA hydrolase 2, mitochondrial OS=Homo sapiens OX=9606 GN=PTRH2 PE=1 SV=1	4	1,633	0,654	1,017	2407651,348	3645775,608	3864427,056
P78536	ADAM17	Disintegrin and metalloproteinase domain-containing protein 17 OS=Homo sapiens OX=9606 GN	3	1,555	0,795	1,017	377844,0254	854285,7638	827502,8734
Q96AX1	VPS33A	Vacuolar protein sorting-associated protein 33A OS=Homo sapiens OX=9606 GN=VPS33A PE=1 S	2	0,653	1,43	1,017	590110,6553	390597,0586	438759,367
Q8TDB6	DTX3L	E3 ubiquitin-protein ligase DTX3L OS=Homo sapiens OX=9606 GN=DTX3L PE=1 SV=1	6	2,793	0,613	1,016	511072,0426	1053597,903	1197296,944
P84085	ARF5	ADP-ribosylation factor 5 OS=Homo sapiens OX=9606 GN=ARF5 PE=1 SV=2	4	0,743	1,176	1,016	15387135,09	16410280,65	13394589,83
Q99747	NAPG	Gamma-soluble NSF attachment protein OS=Homo sapiens OX=9606 GN=NAPG PE=1 SV=1	4	1,072	0,767	1,015	1630168,707	1000448,664	1883364,196
P62913	RPL11	Large ribosomal subunit protein uL5 OS=Homo sapiens OX=9606 GN=RPL11 PE=1 SV=2	4	1,012	1,025	1,015	35028352,76	32808851,32	32824806,86
Q8N3C0	ASCC3	Activating signal cointegrator 1 complex subunit 3 OS=Homo sapiens OX=9606 GN=ASCC3 PE=1 S	5	0,718	0,974	1,015	553957,7266	198317,5215	632523,2035
P13693	TPT1	Translationally-controlled tumor protein OS=Homo sapiens OX=9606 GN=TPT1 PE=1 SV=1	4	0,369	2,734	1,015	22109031,17	8679746,494	8182313,71
P61254	RPL26	Large ribosomal subunit protein uL24 OS=Homo sapiens OX=9606 GN=RPL26 PE=1 SV=1	7	0,518	2,196	1,014	14482706,02	6628772,437	7411618,396
Q13045	FLII	Protein flightless-1 homolog OS=Homo sapiens OX=9606 GN=FLII PE=1 SV=2	8	1,318	0,783	1,013	4595639,619	2591910,867	2713881,787
P11234	RALB	Ras-related protein Ral-B OS=Homo sapiens OX=9606 GN=RALB PE=1 SV=1	2	1,644	0,563	1,011	3308560,767	5586885,695	5211911,012
O75489	NDUFS3	NADH dehydrogenase [ubiquinone] iron-sulfur protein 3, mitochondrial OS=Homo sapiens OX=9	10	1,516	0,648	1,011	12519448,41	19516736,38	19817801,22
Q7Z3B4	NUP54	Nucleoporin p54 OS=Homo sapiens OX=9606 GN=NUP54 PE=1 SV=2	6	1,227	0,917	1,011	3271062,935	4511656,924	5370749,49
O00159	MYO1C	Unconventional myosin-Ic OS=Homo sapiens OX=9606 GN=MYO1C PE=1 SV=4	15	4,545	0,229	1,009	2774976,678	12165706,05	12207267,86
P19404	NDUFV2	NADH dehydrogenase [ubiquinone] flavoprotein 2, mitochondrial OS=Homo sapiens OX=9606 GI	6	1,734	0,666	1,009	5286798,702	8320273,377	8589128,108
Q8IXH7	NELFCD	Negative elongation factor C/D OS=Homo sapiens OX=9606 GN=NELFCD PE=1 SV=2	2	0,793	1,196	1,009	974578,6847	822557,1049	829010,938
Q99832	CCT7	T-complex protein 1 subunit eta OS=Homo sapiens OX=9606 GN=CCT7 PE=1 SV=2	18	0,565	1,601	1,009	62518540,5	28780015,51	40167317,49
Q9Y276	BCS1L	Mitochondrial chaperone BCS1 OS=Homo sapiens OX=9606 GN=BCS1L PE=1 SV=1	2	2,624	0,384	1,007	363834,433	912366,847	988569,0239
P63220	RPS21	Small ribosomal subunit protein eS21 OS=Homo sapiens OX=9606 GN=RPS21 PE=1 SV=1	2	0,564	1,786	1,007	14079055,65	9760185,099	9538789,785
P13804	ETFA	Electron transfer flavoprotein subunit alpha, mitochondrial OS=Homo sapiens OX=9606 GN=ETF	12	3,194	0,3	1,006	14487004,53	43630729,81	38848657,47
Q969N2	PIGT	GPI transamidase component PIG-T OS=Homo sapiens OX=9606 GN=PIGT PE=1 SV=1	6	1,106	1,168	1,006	1929868,358	2246928,437	1594778,194
P31350	RRM2	Ribonucleoside-diphosphate reductase subunit M2 OS=Homo sapiens OX=9606 GN=RRM2 PE=1	8	0,453	1,809	1,006	8386514,977	2409391,958	2939579,427
P30536	TSPO	Translocator protein OS=Homo sapiens OX=9606 GN=TSPO PE=1 SV=3	2	15,409	0,09	1,004	479191,7607	7498010,877	5908214,326
Q8WVM8	SCFD1	Sec1 family domain-containing protein 1 OS=Homo sapiens OX=9606 GN=SCFD1 PE=1 SV=4	10	1,581	0,821	1,004	3503995,989	5286331,522	5142167,979
Q8TDI0	CHD5	Chromodomain-helicase-DNA-binding protein 5 OS=Homo sapiens OX=9606 GN=CHD5 PE=1 SV=	7	0,37	3,277	1,004	7817898,688	3019647,322	2634734,343
Q9UNS2	COPS3	COP9 signalosome complex subunit 3 OS=Homo sapiens OX=9606 GN=COPS3 PE=1 SV=3	4	0,448	2,551	1,003	4340733,981	1451348,79	1906988,223
Q06787	FMR1	Fragile X messenger ribonucleoprotein 1 OS=Homo sapiens OX=9606 GN=FMR1 PE=1 SV=1	5	1,139	0,913	1,002	3976026,293	3752097,244	4627633,828
Q96BW9	TAMM41	Phosphatidate cytidylyltransferase, mitochondrial OS=Homo sapiens OX=9606 GN=TAMM41 PE=	3	1,125	0,935	1,002	380302,8335	397417,4631	396775,8628
Q53GQ0	HSD17B12	Very-long-chain 3-oxoacyl-CoA reductase OS=Homo sapiens OX=9606 GN=HSD17B12 PE=1 SV=2	12	1,545	0,856	1,001	15526429,43	14002102,73	18716984,1
Q8TAA9	VANGL1	Vang-like protein 1 OS=Homo sapiens OX=9606 GN=VANGL1 PE=1 SV=1	3	2,137	0,555	0,999	207931,1631	718240,984	663523,6759
P20290	BTF3	Transcription factor BTF3 OS=Homo sapiens OX=9606 GN=BTF3 PE=1 SV=1	5	0,507	2,388	0,999	7649612,133	3397409,198	2962463,079
P49321	NASP	Nuclear autoantigenic sperm protein OS=Homo sapiens OX=9606 GN=NASP PE=1 SV=2	28	0,323	2,788	0,999	120340505	39465193,62	42417173,91
Q8WY22	BRI3BP	BRI3-binding protein OS=Homo sapiens OX=9606 GN=BRI3BP PE=1 SV=1	3	1,287	0,753	0,998	4365317,73	5686591,014	5618806,912
Q08J23	NSUN2	RNA cytosine C(5)-methyltransferase NSUN2 OS=Homo sapiens OX=9606 GN=NSUN2 PE=1 SV=2	18	0,457	2,277	0,998	21983355,14	8898255,778	9532539,618
P49643	PRIM2	DNA primase large subunit OS=Homo sapiens OX=9606 GN=PRIM2 PE=1 SV=2	2	0,432	2,147	0,997	828262,9644	367104,4112	333161,8722
P51003	PAPOLA	Poly(A) polymerase alpha OS=Homo sapiens OX=9606 GN=PAPOLA PE=1 SV=4	5	0,326	2,665	0,997	1747519,92	632692,7782	636193,0621
Q32MZ4	LRRFIP1	Leucine-rich repeat flightless-interacting protein 1 OS=Homo sapiens OX=9606 GN=LRRFIP1 PE=	7	1,497	0,595	0,996	2210011,291	4122981,031	3844539,875
Q07812	BAX	Apoptosis regulator BAX OS=Homo sapiens OX=9606 GN=BAX PE=1 SV=1	6	0,924	1,143	0,996	6101352,204	5391357,485	5516910,627
Q15435	PPP1R7	Protein phosphatase 1 regulatory subunit 7 OS=Homo sapiens OX=9606 GN=PPP1R7 PE=1 SV=1	6	0,564	1,61	0,994	3186472,664	1329682,38	2142756,773
Q8NF37	LPCAT1	Lysophosphatidylcholine acyltransferase 1 OS=Homo sapiens OX=9606 GN=LPCAT1 PE=1 SV=2	8	0,252	3,545	0,994	12710129,58	2517412,694	3570406,995
Q5H9R7	PPP6R3	Serine/threonine-protein phosphatase 6 regulatory subunit 3 OS=Homo sapiens OX=9606 GN=PI	4	0,61	2,084	0,993	1381573,897	474802,4999	1206290,189
O15173	PGRMC2	Membrane-associated progesterone receptor component 2 OS=Homo sapiens OX=9606 GN=PGF	9	2,441	0,413	0,992	7680924,96	18183611,28	19585249,81
Q8IZ81	ELMOD2	ELMO domain-containing protein 2 OS=Homo sapiens OX=9606 GN=ELMOD2 PE=1 SV=1	6	0,601	1,416	0,992	2418869,739	1397801,749	2310213,82
P31040	SDHA	Succinate dehydrogenase [ubiquinone] flavoprotein subunit, mitochondrial OS=Homo sapiens O	16	1,676	0,787	0,991	42414550,46	78262776,83	55135601,77
O00217	NDUFS8	NADH dehydrogenase [ubiquinone] iron-sulfur protein 8, mitochondrial OS=Homo sapiens OX=9	4	1,48	0,684	0,991	1618131,419	1062554,3	1353641,345
P46777	RPL5	Large ribosomal subunit protein uL18 OS=Homo sapiens OX=9606 GN=RPL5 PE=1 SV=3	9	0,678	1,451	0,991	53986862,7	36863558,22	34781617,46
P62906	RPL10A	Large ribosomal subunit protein uL1 OS=Homo sapiens OX=9606 GN=RPL10A PE=1 SV=2	7	0,951	1,311	0,99	50359776,63	36742991,63	36232660,19
P02649	APOE	Apolipoprotein E OS=Homo sapiens OX=9606 GN=APOE PE=1 SV=1	5	0,213	3,098	0,99	3811413,271	911769,846	630119,6905
O43615	TIMM44	Mitochondrial import inner membrane translocase subunit TIM44 OS=Homo sapiens OX=9606 G	9	1,208	0,84	0,989	8313506,67	9887315,779	10274407,13
Q13724	MOGS	Mannosyl-oligosaccharide glucosidase OS=Homo sapiens OX=9606 GN=MOGS PE=1 SV=5	15	1,133	0,909	0,989	12498425,24	13625526,83	13400479,24
Q3KQU3	MAP7D1	MAP7 domain-containing protein 1 OS=Homo sapiens OX=9606 GN=MAP7D1 PE=1 SV=1	3	0,744	1,397	0,989	1301416,064	883524,31	960260,2691
Q6IN85	PPP4R3A	Serine/threonine-protein phosphatase 4 regulatory subunit 3A OS=Homo sapiens OX=9606 GN=	6	0,671	1,446	0,989	1079319,465	675602,2355	755616,494
P12956	XRCC6	X-ray repair cross-complementing protein 6 OS=Homo sapiens OX=9606 GN=XRCC6 PE=1 SV=2	31	0,661	1,528	0,989	155518714,6	101480814,9	102865190,5
Q01081	U2AF1	Splicing factor U2AF 35 kDa subunit OS=Homo sapiens OX=9606 GN=U2AF1 PE=1 SV=3	4	0,538	1,573	0,989	2764248,921	1886575,917	3310554,041
Q9NP66	HMG20A	High mobility group protein 20A OS=Homo sapiens OX=9606 GN=HMG20A PE=1 SV=1	2	0,918	1,175	0,988	944034,6547	870819,5713	882138,4869
Q9Y3I0	RTCB	RNA-splicing ligase RtcB homolog OS=Homo sapiens OX=9606 GN=RTCB PE=1 SV=1	14	0,568	1,77	0,988	28383855,5	12869855,75	15484567
O43681	GET3	ATPase GET3 OS=Homo sapiens OX=9606 GN=GET3 PE=1 SV=2	7	1,144	0,875	0,986	6328222,134	6409874,865	7268791,913
Q9UL46	PSME2	Proteasome activator complex subunit 2 OS=Homo sapiens OX=9606 GN=PSME2 PE=1 SV=4	13	3,075	0,291	0,985	9026715,041	28885696,26	31554323,4
Q08211	DHX9	ATP-dependent RNA helicase A OS=Homo sapiens OX=9606 GN=DHX9 PE=1 SV=4	41	0,903	1,093	0,985	180097475,9	142866223,1	177129486,4
P62495	ETF1	Eukaryotic peptide chain release factor subunit 1 OS=Homo sapiens OX=9606 GN=ETF1 PE=1 SV=	11	0,782	1,447	0,985	16396144,01	11731064,96	11908929,5
Q9Y4F1	FARP1	FERM, ARHGEF and pleckstrin domain-containing protein 1 OS=Homo sapiens OX=9606 GN=FAR	5	0,455	1,278	0,985	1104224,673	464689,8036	743331,1797
P52294	KPNA1	Importin subunit alpha-5 OS=Homo sapiens OX=9606 GN=KPNA1 PE=1 SV=3	2	1,085	0,911	0,984	1371292,694	1397067,1	1407389,609
Q99623	PHB2	Prohibitin-2 OS=Homo sapiens OX=9606 GN=PHB2 PE=1 SV=2	16	0,934	1,204	0,984	73325554,1	67205522,54	65333430,54
Q9BY32	ITPA	Inosine triphosphate pyrophosphatase OS=Homo sapiens OX=9606 GN=ITPA PE=1 SV=2	2	0,884	1,303	0,984	1573301,981	1624972,948	1693288,231
O15294	OGT	UDP-N-acetylglucosamine--peptide N-acetylglucosaminyltransferase 110 kDa subunit OS=Homo	6	0,568	1,827	0,984	1546121,946	897701,1402	940637,3628
O75165	DNAJC13	DnaJ homolog subfamily C member 13 OS=Homo sapiens OX=9606 GN=DNAJC13 PE=1 SV=5	8	1,354	0,734	0,983	1312139,891	1345115,392	2035647,772
Q86U42	PABPN1	Polyadenylate-binding protein 2 OS=Homo sapiens OX=9606 GN=PABPN1 PE=1 SV=3	6	0,957	1,005	0,983	10511183,22	9715091,136	10284320,61
P56537	EIF6	Eukaryotic translation initiation factor 6 OS=Homo sapiens OX=9606 GN=EIF6 PE=1 SV=1	5	0,955	1,029	0,983	14480758,51	13841430,32	12030094,88
Q96RD7	PANX1	Pannexin-1 OS=Homo sapiens OX=9606 GN=PANX1 PE=1 SV=4	2	1,705	0,743	0,982	355041,4765	571550,5955	566370,1587
P49454	CENPF	Centromere protein F OS=Homo sapiens OX=9606 GN=CENPF PE=1 SV=3	4	1,525	0,802	0,982	1361733,436	2597410,298	1814047,658
Q9UPT8	ZC3H4	Zinc finger CCCH domain-containing protein 4 OS=Homo sapiens OX=9606 GN=ZC3H4 PE=1 SV=3	2	0,966	0,899	0,982	264308,3508	143756,512	288776,2022
Q9BQA1	WDR77	Methylosome protein WDR77 OS=Homo sapiens OX=9606 GN=WDR77 PE=1 SV=1	3	0,418	2,391	0,982	3120413,917	1363845,61	1470186,401
P56134	ATP5MF	ATP synthase subunit f, mitochondrial OS=Homo sapiens OX=9606 GN=ATP5MF PE=1 SV=3	2	1,088	0,876	0,98	5432089,657	5102095,616	6358238,88
Q9NTX5	ECHDC1	Ethylmalonyl-CoA decarboxylase OS=Homo sapiens OX=9606 GN=ECHDC1 PE=1 SV=2	6	0,971	0,975	0,98	3434795,679	3369002,928	2680327,663
Q9Y570	PPME1	Protein phosphatase methylesterase 1 OS=Homo sapiens OX=9606 GN=PPME1 PE=1 SV=3	6	0,711	1,725	0,98	3632589,858	2484989,256	1810383,308
Q96CW1	AP2M1	AP-2 complex subunit mu OS=Homo sapiens OX=9606 GN=AP2M1 PE=1 SV=2	10	1,792	0,548	0,979	5962685,36	10509769,01	9843811,331
P51991	HNRNPA3	Heterogeneous nuclear ribonucleoprotein A3 OS=Homo sapiens OX=9606 GN=HNRNPA3 PE=1 S	14	0,786	1,181	0,979	144956016,6	118069643,5	141087008
Q9Y5A9	YTHDF2	YTH domain-containing family protein 2 OS=Homo sapiens OX=9606 GN=YTHDF2 PE=1 SV=2	2	0,622	1,574	0,979	2881752,506	1727614,325	1208976,946
O75367	MACROH2A1	Core histone macro-H2A.1 OS=Homo sapiens OX=9606 GN=MACROH2A1 PE=1 SV=5	12	0,653	1,32	0,978	100124004	61752431,24	75155972,4
Q9Y6K1	DNMT3A	DNA (cytosine-5)-methyltransferase 3A OS=Homo sapiens OX=9606 GN=DNMT3A PE=1 SV=4	4	0,135	6,309	0,978	2400387,756	223756,7201	343325,4562
O43292	GPAA1	Glycosylphosphatidylinositol anchor attachment 1 protein OS=Homo sapiens OX=9606 GN=GPA	3	1,778	0,656	0,976	1873596,956	3066651,205	2743644,501
P31943	HNRNPH1	Heterogeneous nuclear ribonucleoprotein H OS=Homo sapiens OX=9606 GN=HNRNPH1 PE=1 SV	7	0,703	1,33	0,976	166415206,3	107703298,1	117634957,2
P45880	VDAC2	Voltage-dependent anion-selective channel protein 2 OS=Homo sapiens OX=9606 GN=VDAC2 PE	8	1,333	0,845	0,973	61838287,7	69958483,38	69022827,68
A2A3N6	PIPSL	Putative PIP5K1A and PSMD4-like protein OS=Homo sapiens OX=9606 GN=PIPSL PE=5 SV=1	2	0,889	1,098	0,973	269168,9379	251749,1496	272937,024
P78344	EIF4G2	Eukaryotic translation initiation factor 4 gamma 2 OS=Homo sapiens OX=9606 GN=EIF4G2 PE=1	25	0,587	1,526	0,973	21280989,45	11681111,14	15260863,14
Q7L2H7	EIF3M	Eukaryotic translation initiation factor 3 subunit M OS=Homo sapiens OX=9606 GN=EIF3M PE=1	5	0,494	1,481	0,973	2188443,466	2209925,733	1780702,58
Q14011	CIRBP	Cold-inducible RNA-binding protein OS=Homo sapiens OX=9606 GN=CIRBP PE=1 SV=1	4	0,775	1,151	0,972	9890568,493	7397575,716	8475834,5

Q9P2J5	LARS1	Leucine--tRNA ligase, cytoplasmic OS=Homo sapiens OX=9606 GN=LARS1 PE=1 SV=2	29	0,602	1,625	0,972	28499116,78	16083032,06	18413524,04
P12814	ACTN1	Alpha-actinin-1 OS=Homo sapiens OX=9606 GN=ACTN1 PE=1 SV=2	28	2,251	0,486	0,971	87511616,76	180645926,1	180921185,6
P58546	MTPN	Myotrophin OS=Homo sapiens OX=9606 GN=MTPN PE=1 SV=2	2	0,413	2,698	0,971	6996947,27	2759859,663	2157190,977
O95298	NDUFC2	NADH dehydrogenase [ubiquinone] 1 subunit C2 OS=Homo sapiens OX=9606 GN=NDUFC2 PE=1	4	2,138	0,49	0,97	3010766,957	6287456,832	5963849,932
Q15907	RAB11B	Ras-related protein Rab-11B OS=Homo sapiens OX=9606 GN=RAB11B PE=1 SV=4	11	1,193	0,915	0,97	50621883,14	60261957,22	62919598,2
Q8WVY7	UBLCP1	Ubiquitin-like domain-containing CTD phosphatase 1 OS=Homo sapiens OX=9606 GN=UBLCP1 PE	3	0,579	1,683	0,97	1728504,473	949634,842	1049359,849
P18621	RPL17	Large ribosomal subunit protein uL22 OS=Homo sapiens OX=9606 GN=RPL17 PE=1 SV=3	3	0,575	1,291	0,97	14985529,5	8240020,221	10165062,56
Q9UP95	SLC12A4	Solute carrier family 12 member 4 OS=Homo sapiens OX=9606 GN=SLC12A4 PE=1 SV=2	5	2,095	0,403	0,968	841919,3944	1920372,781	1996942,862
P39019	RPS19	Small ribosomal subunit protein eS19 OS=Homo sapiens OX=9606 GN=RPS19 PE=1 SV=2	8	0,806	1,263	0,968	50789485,17	36286106,68	39929551,02
Q9UBQ5	EIF3K	Eukaryotic translation initiation factor 3 subunit K OS=Homo sapiens OX=9606 GN=EIF3K PE=1 S\	3	0,702	1,292	0,968	6654024,163	4445195,596	5027149,658
Q13148	TARDBP	TAR DNA-binding protein 43 OS=Homo sapiens OX=9606 GN=TARDBP PE=1 SV=1	8	0,655	1,393	0,967	21135109,56	15586567,28	17893309,67
Q15397	PUM3	Pumilio homolog 3 OS=Homo sapiens OX=9606 GN=PUM3 PE=1 SV=3	6	1,312	0,835	0,966	1059214,546	1285849,397	1259329,501
Q96A26	FAM162A	Protein FAM162A OS=Homo sapiens OX=9606 GN=FAM162A PE=1 SV=2	6	0,91	0,824	0,966	5775186,215	2831790,129	5304835,918
Q9UBB4	ATXN10	Ataxin-10 OS=Homo sapiens OX=9606 GN=ATXN10 PE=1 SV=1	8	0,35	2,658	0,966	6747961,573	2753671,751	2996980,874
Q13751	LAMB3	Laminin subunit beta-3 OS=Homo sapiens OX=9606 GN=LAMB3 PE=1 SV=1	11	3,884	0,237	0,965	169607,7336	316608,0423	3356465,116
Q7L1Q6	BZW1	eIF5-mimic protein 2 OS=Homo sapiens OX=9606 GN=BZW1 PE=1 SV=1	9	0,585	1,691	0,965	12190841,96	7468025,828	7487777,664
Q92598	HSPH1	Heat shock protein 105 kDa OS=Homo sapiens OX=9606 GN=HSPH1 PE=1 SV=1	21	0,544	1,654	0,965	42413259,81	23835601,58	25976744,54
Q6PJG6	BRAT1	BRCA1-associated ATM activator 1 OS=Homo sapiens OX=9606 GN=BRAT1 PE=1 SV=2	2	0,463	1,793	0,964	650132,9642	210624,7981	341066,2983
Q6NSJ5	LRRC8E	Volume-regulated anion channel subunit LRRC8E OS=Homo sapiens OX=9606 GN=LRRC8E PE=1 S	2	1000	0,001	0,963		225904,3396	257971,345
Q9H201	EPN3	Epsin-3 OS=Homo sapiens OX=9606 GN=EPN3 PE=1 SV=1	2	0,077	1,323	0,963	2808065,118	1059264,128	2456931,482
P06865	HEXA	Beta-hexosaminidase subunit alpha OS=Homo sapiens OX=9606 GN=HEXA PE=1 SV=2	10	2,194	0,489	0,962	4149959,475	8050036,313	9767494,694
Q13190	STX5	Syntaxin-5 OS=Homo sapiens OX=9606 GN=STX5 PE=1 SV=2	4	2,014	0,431	0,962	1594495,771	2882923,967	3665390,608
P08754	GNAI3	Guanine nucleotide-binding protein G(i) subunit alpha-3 OS=Homo sapiens OX=9606 GN=GNAI3	4	1,105	0,874	0,962	7146327,484	7737025,623	7924192,156
Q9HB71	CACYBP	Calcyclin-binding protein OS=Homo sapiens OX=9606 GN=CACYBP PE=1 SV=2	11	0,352	2,286	0,962	16623760,99	6903627,002	7056106
Q9BXB4	OSBPL11	Oxysterol-binding protein-related protein 11 OS=Homo sapiens OX=9606 GN=OSBPL11 PE=1 SV=	2	0,679	1,478	0,961	924718,7612	633922,1121	550207,0116
Q86UV5	USP48	Ubiquitin carboxyl-terminal hydrolase 48 OS=Homo sapiens OX=9606 GN=USP48 PE=1 SV=1	3	0,196	2,328	0,961	1651714,317	359016,0624	529061,9892
O43172	PRPF4	U4/U6 small nuclear ribonucleoprotein Prp4 OS=Homo sapiens OX=9606 GN=PRPF4 PE=1 SV=2	9	1,23	0,766	0,96	4423746,921	4260281,532	6486587,091
A0A804HII9	ACTN1	Alpha-actinin-1 OS=Homo sapiens OX=9606 GN=ACTN1 PE=1 SV=1	27	1,946	0,541	0,959	84126108,19	149708697,2	154772876
P20340	RAB6A	Ras-related protein Rab-6A OS=Homo sapiens OX=9606 GN=RAB6A PE=1 SV=3	7	1,521	0,645	0,958	8033024,576	9576667,985	15404196,56
P23246	SFPQ	Splicing factor, proline- and glutamine-rich OS=Homo sapiens OX=9606 GN=SFPQ PE=1 SV=2	22	0,814	1,394	0,958	206102273,2	165080054	177468139,8
O96000	NDUFB10	NADH dehydrogenase [ubiquinone] 1 beta subcomplex subunit 10 OS=Homo sapiens OX=9606 G	4	1,541	0,595	0,957	4481728,226	6424959,527	7639704,133
Q15052	ARHGEF6	Rho guanine nucleotide exchange factor 6 OS=Homo sapiens OX=9606 GN=ARHGEF6 PE=1 SV=2	4	0,797	0,851	0,957	745325,3068	1082694,936	738102,203
Q9GZZ1	NAA50	N-alpha-acetyltransferase 50 OS=Homo sapiens OX=9606 GN=NAA50 PE=1 SV=1	3	0,535	2,153	0,956	2395185,666	1289902,724	1218561,066
Q99733	NAP1L4	Nucleosome assembly protein 1-like 4 OS=Homo sapiens OX=9606 GN=NAP1L4 PE=1 SV=1	6	0,34	1,424	0,956	8860424,903	2501531,8	4650287,841
P00338	LDHA	L-lactate dehydrogenase A chain OS=Homo sapiens OX=9606 GN=LDHA PE=1 SV=2	19	1,076	0,841	0,955	152279730,9	158658485,2	180946685,7
P38117	ETFB	Electron transfer flavoprotein subunit beta OS=Homo sapiens OX=9606 GN=ETFB PE=1 SV=3	12	3,456	0,281	0,953	11485826,23	36137687,05	42467832,53
P48444	ARCN1	Coatomer subunit delta OS=Homo sapiens OX=9606 GN=ARCN1 PE=1 SV=1	13	0,818	1,228	0,953	13135639,84	11367215,67	10814452,44
P56192	MARS1	Methionine--tRNA ligase, cytoplasmic OS=Homo sapiens OX=9606 GN=MARS1 PE=1 SV=2	17	0,665	1,46	0,953	28947003,16	17896054,41	31940186,45
J3KQL8	APOL2	Apolipoprotein L2 OS=Homo sapiens OX=9606 GN=APOL2 PE=1 SV=2	7	6,574	0,243	0,951	4678093,851	35444750,12	18500672
Q9Y277	VDAC3	Voltage-dependent anion-selective channel protein 3 OS=Homo sapiens OX=9606 GN=VDAC3 PE	7	0,852	0,814	0,951	18942568,37	17392067,83	22983979,22
O95832	CLDN1	Claudin-1 OS=Homo sapiens OX=9606 GN=CLDN1 PE=1 SV=1	2	1000	0,001	0,949		1094770,682	1287273,23
Q9BTD8	RBM42	RNA-binding protein 42 OS=Homo sapiens OX=9606 GN=RBM42 PE=1 SV=1	5	1,657	0,65	0,949	1242275,184	1467798,302	1981136,884
Q13131	PRKAA1	5'-AMP-activated protein kinase catalytic subunit alpha-1 OS=Homo sapiens OX=9606 GN=PRKA/	5	1,286	1,342	0,949	2622390,36	1991904,539	229162,2885
P62140	PPP1CB	Serine/threonine-protein phosphatase PP1-beta catalytic subunit OS=Homo sapiens OX=9606 GI	2	0,965	0,983	0,949	2448849,02	2470849,614	2508238,609
Q8IXT5	RBM12B	RNA-binding protein 12B OS=Homo sapiens OX=9606 GN=RBM12B PE=1 SV=2	7	0,346	2,248	0,948	3488894,873	976192,6189	1422651,94
P23921	RRM1	Ribonucleoside-diphosphate reductase large subunit OS=Homo sapiens OX=9606 GN=RRM1 PE=	14	0,321	3,619	0,948	14178065,61	3617274,625	3685677,842
Q13439	GOLGA4	Golgin subfamily A member 4 OS=Homo sapiens OX=9606 GN=GOLGA4 PE=1 SV=1	6	1,611	0,434	0,947	2318895,856	1925136,656	2534073,661
Q15554	TERF2	Telomeric repeat-binding factor 2 OS=Homo sapiens OX=9606 GN=TERF2 PE=1 SV=3	4	0,782	1,133	0,945	2402259,616	1939295,372	2101731,01
P28482	MAPK1	Mitogen-activated protein kinase 1 OS=Homo sapiens OX=9606 GN=MAPK1 PE=1 SV=3	9	0,753	1,236	0,945	5529863,438	3805847,706	4014848,989
P11142	HSPA8	Heat shock cognate 71 kDa protein OS=Homo sapiens OX=9606 GN=HSPA8 PE=1 SV=1	20	0,596	1,463	0,944	731925581,1	437227931,6	508086909,6
O75175	CNOT3	CCR4-NOT transcription complex subunit 3 OS=Homo sapiens OX=9606 GN=CNOT3 PE=1 SV=1	3			0,943	129201,9742	284329,5603	301601,6568
Q96QC0	PPP1R10	Serine/threonine-protein phosphatase 1 regulatory subunit 10 OS=Homo sapiens OX=9606 GN=I	5	0,494	1,659	0,943	1844061,741	588321,6567	1197073,195
P15291	B4GALT1	Beta-1,4-galactosyltransferase 1 OS=Homo sapiens OX=9606 GN=B4GALT1 PE=1 SV=5	4	5,541	0,185	0,942	452731,5718	1836097,155	2314528,387
Q00577	PURA	Transcriptional activator protein Pur-alpha OS=Homo sapiens OX=9606 GN=PURA PE=1 SV=2	6	2,753	0,266	0,941	2199628,714	6201960,677	6467932,313
P35869	AHR	Aryl hydrocarbon receptor OS=Homo sapiens OX=9606 GN=AHR PE=1 SV=2	3	0,811	1,162	0,941	135102,714	78060,77428	82675,01887
Q9UKL0	RCOR1	REST corepressor 1 OS=Homo sapiens OX=9606 GN=RCOR1 PE=1 SV=2	6	0,718	1,032	0,941	2484367,087	1032199,451	2426623,835
Q96Q11	TRNT1	CCA tRNA nucleotidyltransferase 1, mitochondrial OS=Homo sapiens OX=9606 GN=TRNT1 PE=1 S	3	1,454	0,656	0,94	961605,4154	1321379,976	1480616,15
Q8N2U0	TMEM256	Transmembrane protein 256 OS=Homo sapiens OX=9606 GN=TMEM256 PE=3 SV=1	2	0,858	1,05	0,94	692022,9698	593637,7026	631577,0752
P62263	RPS14	Small ribosomal subunit protein uS11 OS=Homo sapiens OX=9606 GN=RPS14 PE=1 SV=3	5	0,848	1,141	0,94	59141227,36	33034006,71	51755948,39
P63279	UBE2I	SUMO-conjugating enzyme UBC9 OS=Homo sapiens OX=9606 GN=UBE2I PE=1 SV=1	5	0,556	1,897	0,94	16096901,13	8821210,649	9136434,754
P24941	CDK2	Cyclin-dependent kinase 2 OS=Homo sapiens OX=9606 GN=CDK2 PE=1 SV=2	5	0,49	2,109	0,94	3629300,059	1912182,321	2278626,884
P11172	UMPS	Uridine 5'-monophosphate synthase OS=Homo sapiens OX=9606 GN=UMPS PE=1 SV=1	7	0,461	2,067	0,94	4731396,139	1740885,21	2367370,421
Q8N3U4	STAG2	Cohesin subunit SA-2 OS=Homo sapiens OX=9606 GN=STAG2 PE=1 SV=3	7	0,858	1,146	0,939	4446198,799	4406618,745	5547332,665
Q9H223	EHD4	EH domain-containing protein 4 OS=Homo sapiens OX=9606 GN=EHD4 PE=1 SV=1	14	2,436	0,421	0,938	2097145,362	6570768,058	5706123,002
O95721	SNAP29	Synaptosomal-associated protein 29 OS=Homo sapiens OX=9606 GN=SNAP29 PE=1 SV=1	2	1,293	0,723	0,938	1020527,308	1300792,17	1439514,939
Q92878	RAD50	DNA repair protein RAD50 OS=Homo sapiens OX=9606 GN=RAD50 PE=1 SV=1	18	2,46	0,367	0,937	3681569,701	8741457,928	10343057,17
P52306	RAP1GDS1	Rap1 GTPase-GDP dissociation stimulator 1 OS=Homo sapiens OX=9606 GN=RAP1GDS1 PE=1 SV=	2	0,975	0,919	0,937	2060611,079	1590505,976	2409610,918
P46937	YAP1	Transcriptional coactivator YAP1 OS=Homo sapiens OX=9606 GN=YAP1 PE=1 SV=2	4	0,663	1,514	0,937	1807518,504	1055702,245	1133249,162
O14646	CHD1	Chromodomain-helicase-DNA-binding protein 1 OS=Homo sapiens OX=9606 GN=CHD1 PE=1 SV=	2	0,39	2,279	0,937	572050,0468	234820,9555	299938,9458
Q6IAA8	LAMTOR1	Ragulator complex protein LAMTOR1 OS=Homo sapiens OX=9606 GN=LAMTOR1 PE=1 SV=2	7	1,249	0,739	0,936	3304234,974	3996461,418	4273483,753
Q86V81	ALYREF	THO complex subunit 4 OS=Homo sapiens OX=9606 GN=ALYREF PE=1 SV=3	8	1,019	0,992	0,936	36393823,31	33366627,52	37239092,88
P14406	COX7A2	Cytochrome c oxidase subunit 7A2, mitochondrial OS=Homo sapiens OX=9606 GN=COX7A2 PE=1	2	1,373	0,661	0,935	13132103,92	16789448	19006060,74
Q00839	HNRNPU	Heterogeneous nuclear ribonucleoprotein U OS=Homo sapiens OX=9606 GN=HNRNPU PE=1 SV=	17	0,68	1,409	0,935	293252350,3	190882281,7	209400953,4
P27701	CD82	CD82 antigen OS=Homo sapiens OX=9606 GN=CD82 PE=1 SV=1	4	16,716	0,062	0,934	322087,0456	6001343,318	7307544,308
Q99653	CHP1	Calcineurin B homologous protein 1 OS=Homo sapiens OX=9606 GN=CHP1 PE=1 SV=3	6	1,424	0,681	0,934	3645878,057	3662201,056	5577682,193
Q92879	CELF1	CUGBP Elav-like family member 1 OS=Homo sapiens OX=9606 GN=CELF1 PE=1 SV=2	4	0,629	1,737	0,934	3303939,438	1275124,629	1834988,146
O43760	SYNGR2	Synaptogyrin-2 OS=Homo sapiens OX=9606 GN=SYNGR2 PE=1 SV=1	3	3,845	0,233	0,933	568907,6683	4139985,132	4084727,27
Q9UMS4	PRPF19	Pre-mRNA-processing factor 19 OS=Homo sapiens OX=9606 GN=PRPF19 PE=1 SV=1	12	0,776	1,457	0,933	29188480,46	19216593,73	22213953,31
Q8TDB8	SLC2A14	Solute carrier family 2, facilitated glucose transporter member 14 OS=Homo sapiens OX=9606 GI	3	0,168	3,85	0,933	3198093,217	558863,6879	624554,3102
Q9P0J1	PDP1	[Pyruvate dehydrogenase [acetyl-transferring]]-phosphatase 1, mitochondrial OS=Homo sapiens	3	2,935	0,331	0,932	593402,4273	1691381,032	1718210,024
Q9UIA9	XPO7	Exportin-7 OS=Homo sapiens OX=9606 GN=XPO7 PE=1 SV=3	7	0,728	1,242	0,932	1435349,066	1164690,308	1589545,994
P62277	RPS13	Small ribosomal subunit protein uS15 OS=Homo sapiens OX=9606 GN=RPS13 PE=1 SV=2	5	0,646	1,218	0,932	34962539,37	20983405,48	28985578,09
Q5M775	SPECC1	Cytospin-B OS=Homo sapiens OX=9606 GN=SPECC1 PE=1 SV=1	5	2,58	0,367	0,931	2013511,071	2671424,205	3230338,739
Q9P0I2	EMC3	ER membrane protein complex subunit 3 OS=Homo sapiens OX=9606 GN=EMC3 PE=1 SV=3	5	1,958	0,533	0,931	1670201,444	2678010,319	4150358,614
Q16204	CCDC6	Coiled-coil domain-containing protein 6 OS=Homo sapiens OX=9606 GN=CCDC6 PE=1 SV=2	3	1,002	1,008	0,931	1397186,591	867576,436	1289993,925
O00170	AIP	AH receptor-interacting protein OS=Homo sapiens OX=9606 GN=AIP PE=1 SV=3	3	0,855	1,003	0,929	1981184,456	1852630,997	1913768,839
Q00169	PITPNA	Phosphatidylinositol transfer protein alpha isoform OS=Homo sapiens OX=9606 GN=PITPNA PE=	5	0,325	3,236	0,929	2955891,785	671178,7833	637245,8526
P62857	RPS28	Small ribosomal subunit protein eS28 OS=Homo sapiens OX=9606 GN=RPS28 PE=1 SV=1	2	0,778	1,164	0,928	34549252,12	26921615,11	28526084,57
Q06323	PSME1	Proteasome activator complex subunit 1 OS=Homo sapiens OX=9606 GN=PSME1 PE=1 SV=1	15	4,152	0,217	0,927	11808325,54	44414242,31	65235155,38
O15127	SCAMP2	Secretory carrier-associated membrane protein 2 OS=Homo sapiens OX=9606 GN=SCAMP2 PE=1	2	2,078	0,431	0,927	2611876,4	5258847,485	6035686,014
P20592	MX2	Interferon-induced GTP-binding protein Mx2 OS=Homo sapiens OX=9606 GN=MX2 PE=1 SV=1	19	0,985	0,466	0,927	23506962,91	40439380,17	40971836,69
O00571	DDX3X	ATP-dependent RNA helicase DDX3X OS=Homo sapiens OX=9606 GN=DDX3X PE=1 SV=3	7	0,479	1,615	0,927	49154061,66	22175194,5	28904520,1
Q9NYB0	TERF2IP	Telomeric repeat-binding factor 2-interacting protein 1 OS=Homo sapiens OX=9606 GN=TERF2IP	2	0,874	1,021	0,926	1504330,383	1281762,008	1365582,527
P20336	RAB3A	Ras-related protein Rab-3A OS=Homo sapiens OX=9606 GN=RAB3A PE=1 SV=1	3	1,205	0,907	0,925	1871584,164	1627049,381	2048029,92
O94880	PHF14	PHD finger protein 14 OS=Homo sapiens OX=9606 GN=PHF14 PE=1 SV=3	2	0,624	1,43	0,925	850110,0968	299835,6738	335717,0007
Q12824	SMARCB1	SWI/SNF-related matrix-associated actin-dependent regulator of chromatin subfamily B membe	6	0,623	1,775	0,925	6136922,031	1423371,51	1758161,555
P32119	PRDX2	Peroxiredoxin-2 OS=Homo sapiens OX=9606 GN=PRDX2 PE=1 SV=5	7	0,488	1,852	0,925	58578667,04	27776192,29	32791071,11
Q92990	GLMN	Glomulin OS=Homo sapiens OX=9606 GN=GLMN PE=1 SV=2	3	0,374	2,582	0,925	1290657,901	466099,4263	537452,5468
Q15631	TSN	Translin OS=Homo sapiens OX=9606 GN=TSN PE=1 SV=1	7	0,44	2,106	0,924	13166231,04	6659516,745	7660577,941
P35232	PHB1	Prohibitin 1 OS=Homo sapiens OX=9606 GN=PHB1 PE=1 SV=1	15	0,961	0,953	0,921	74374223,06	69746081,64	94883118,38
P19387	POLR2C	DNA-directed RNA polymerase II subunit RPB3 OS=Homo sapiens OX=9606 GN=POLR2C PE=1 SV	4	0,654	1,445	0,921	2308002,282	1573450,636	1872539,824
P25705	ATP5F1A	ATP synthase subunit alpha, mitochondrial OS=Homo sapiens OX=9606 GN=ATP5F1A PE=1 SV=1	23	1,395	0,647	0,92	173745047,6	226519148,4	253933545,3
Q9UNZ2	NSFL1C	NSFL1 cofactor p47 OS=Homo sapiens OX=9606 GN=NSFL1C PE=1 SV=2	11	0,563	1,444	0,919	20050299,96	13463058,89	13846527,76
O14981	BTAF1	TATA-binding protein-associated factor 172 OS=Homo sapiens OX=9606 GN=BTAF1 PE=1 SV=2	2	0,503	2,399	0,919	629755,4539	227730,0775	296957,4362
P22234	PAICS	Bifunctional phosphoribosylaminoimidazole carboxylase/phosphoribosylaminoimidazole succino	11	0,362	2,635	0,919	28974608,06	9048092,481	10272480,2
O14681	EI24	Etoposide-induced protein 2.4 homolog OS=Homo sapiens OX=9606 GN=EI24 PE=1 SV=4	3	0,913	1,142	0,918	370182,4929	644361,7938	923499,7463
O00303	EIF3F	Eukaryotic translation initiation factor 3 subunit F OS=Homo sapiens OX=9606 GN=EIF3F PE=1 S\	10	0,597	1,332	0,918	18216475,55	9309800,393	13815106,72
P05362	ICAM1	Intercellular adhesion molecule 1 OS=Homo sapiens OX=9606 GN=ICAM1 PE=1 SV=2	6	12,547	0,071	0,917	977860,9793	10875156,21	12611875
Q8WWY3	PRPF31	U4/U6 small nuclear ribonucleoprotein Prp31 OS=Homo sapiens OX=9606 GN=PRPF31 PE=1 SV=	9	1,009	0,848	0,917	4320369,992	4098786,765	5194013,196
Q99700	ATXN2	Ataxin-2 OS=Homo sapiens OX=9606 GN=ATXN2 PE=1 SV=2	2	0,828	1,107	0,917	368963,7762	295658,5584	335875,0727
P50914	RPL14	Large ribosomal subunit protein eL14 OS=Homo sapiens OX=9606 GN=RPL14 PE=1 SV=4	2	0,825	1,668	0,917	14068569,34	8201200,323	12117027,35

P51571	SSR4	Translocon-associated protein subunit delta OS=Homo sapiens OX=9606 GN=SSR4 PE=1 SV=1	4	1,257	0,705	0,916	14189340,67	18798091,8	20177584,07
O75694	NUP155	Nuclear pore complex protein Nup155 OS=Homo sapiens OX=9606 GN=NUP155 PE=1 SV=1	25	0,91	1,019	0,916	29893472,64	25125253,22	24339877,37
Q92499	DDX1	ATP-dependent RNA helicase DDX1 OS=Homo sapiens OX=9606 GN=DDX1 PE=1 SV=2	17	0,482	1,899	0,916	22938003,75	9171844,9	11576718,12
P30566	ADSL	Adenylosuccinate lyase OS=Homo sapiens OX=9606 GN=ADSL PE=1 SV=2	7	0,21	3,731	0,916	8917694,727	2097428,287	1835081,538
P49959	MRE11	Double-strand break repair protein MRE11 OS=Homo sapiens OX=9606 GN=MRE11 PE=1 SV=3	16	1,63	0,592	0,915	2189982,47	3131834,111	4440379,825
Q9BUL8	PDCD10	Programmed cell death protein 10 OS=Homo sapiens OX=9606 GN=PDCD10 PE=1 SV=1	6	0,786	0,816	0,915	3589830,804	2035646,18	3066375,983
P49411	TUFM	Elongation factor Tu, mitochondrial OS=Homo sapiens OX=9606 GN=TUFM PE=1 SV=3	19	1,61	0,504	0,914	76278825,38	117397451,8	145684531,4
Q9C005	DPY30	Protein dpy-30 homolog OS=Homo sapiens OX=9606 GN=DPY30 PE=1 SV=1	3	0,709	0,928	0,914	3285365,145	2309874,23	4401683,377
P09884	POLA1	DNA polymerase alpha catalytic subunit OS=Homo sapiens OX=9606 GN=POLA1 PE=1 SV=2	3	0,492	1,927	0,914	873413,626	448096,9767	841739,8114
Q96ER3	SAAL1	Protein SAAL1 OS=Homo sapiens OX=9606 GN=SAAL1 PE=1 SV=2	2	0,402	2,244	0,914	314772,2272	120467,6684	131813,53
O00186	STXBP3	Syntaxin-binding protein 3 OS=Homo sapiens OX=9606 GN=STXBP3 PE=1 SV=2	8	3,307	0,286	0,912	915738,545	4747141,466	5788204,012
Q9NX47	MARCHF5	E3 ubiquitin-protein ligase MARCHF5 OS=Homo sapiens OX=9606 GN=MARCHF5 PE=1 SV=1	2	1,238	0,73	0,912	1565219,632	455860,8152	1860177,945
P22670	RFX1	MHC class II regulatory factor RFX1 OS=Homo sapiens OX=9606 GN=RFX1 PE=1 SV=2	2	0,975	0,83	0,912	336543,8307	319088,8597	437294,6067
O14745	NHERF1	Na(+)/H(+) exchange regulatory cofactor NHE-RF1 OS=Homo sapiens OX=9606 GN=NHERF1 PE=1	7	1,813	0,512	0,911	2476941,817	4675686,221	5773706,924
P28331	NDUFS1	NADH-ubiquinone oxidoreductase 75 kDa subunit, mitochondrial OS=Homo sapiens OX=9606 GN	23	1,376	0,63	0,91	16988049,68	23362350,08	27626404,87
P10515	DLAT	Dihydrolipoyllysine-residue acetyltransferase component of pyruvate dehydrogenase complex, n	11	0,941	1,02	0,909	18641059,01	12232676,96	16267169,71
O14980	XPO1	Exportin-1 OS=Homo sapiens OX=9606 GN=XPO1 PE=1 SV=1	30	0,65	1,318	0,908	45391599,19	28055207,87	32346051,59
Q99755	PIP5K1A	Phosphatidylinositol 4-phosphate 5-kinase type-1 alpha OS=Homo sapiens OX=9606 GN=PIP5K1	2	0,807	1,124	0,907	260424,0399	210088,6306	231724,1027
Q9H6T3	RPAP3	RNA polymerase II-associated protein 3 OS=Homo sapiens OX=9606 GN=RPAP3 PE=1 SV=2	4	0,682	1,613	0,907	1281748,368	1697490,305	410549,5486
Q9ULU4	ZMYND8	MYND-type zinc finger-containing chromatin reader ZMYND8 OS=Homo sapiens OX=9606 GN=Z	3	0,456	1,666	0,907	226304,2654	139928,9186	365143,8397
Q8N183	NDUFAF2	NADH dehydrogenase [ubiquinone] 1 alpha subcomplex assembly factor 2 OS=Homo sapiens OX	3	0,712	1,098	0,906	3225174,816	2611394,787	4766912,176
Q96JM3	CHAMP1	Chromosome alignment-maintaining phosphoprotein 1 OS=Homo sapiens OX=9606 GN=CHAMP	5	0,489	1,892	0,906	1683357,159	741139,3295	991095,6025
O43707	ACTN4	Alpha-actinin-4 OS=Homo sapiens OX=9606 GN=ACTN4 PE=1 SV=2	37	2,634	0,344	0,905	180185620,9	433115610,3	499268473,5
Q15067	ACOX1	Peroxisomal acyl-coenzyme A oxidase 1 OS=Homo sapiens OX=9606 GN=ACOX1 PE=1 SV=3	16	2,157	0,432	0,905	1500912,848	3124222,939	4582026,741
Q96CS3	FAF2	FAS-associated factor 2 OS=Homo sapiens OX=9606 GN=FAF2 PE=1 SV=2	6	1,196	0,863	0,905	6253658,039	6921816,833	10110678,54
Q92688	ANP32B	Acidic leucine-rich nuclear phosphoprotein 32 family member B OS=Homo sapiens OX=9606 GN	10	0,672	1,321	0,905	47038151,78	25969166,21	30535029,6
Q9UBW7	ZMYM2	Zinc finger MYM-type protein 2 OS=Homo sapiens OX=9606 GN=ZMYM2 PE=1 SV=1	2	0,264	2,834	0,905	1930637,915	174734,305	696382,0937
Q5XKP0	MICOS13	MICOS complex subunit MIC13 OS=Homo sapiens OX=9606 GN=MICOS13 PE=1 SV=1	3	1,582	0,575	0,904	892539,7285	1561531,598	1936785,979
Q9BSC4	NOL10	Nucleolar protein 10 OS=Homo sapiens OX=9606 GN=NOL10 PE=1 SV=1	2	1,376	0,86	0,904	333170,5478	416244,4905	675318,155
Q9NX46	ADPRS	ADP-ribosylhydrolase ARH3 OS=Homo sapiens OX=9606 GN=ADPRS PE=1 SV=1	5	0,74	1,288	0,904	2611830,144	2546834,954	1985496,505
P61009	SPCS3	Signal peptidase complex subunit 3 OS=Homo sapiens OX=9606 GN=SPCS3 PE=1 SV=1	4	1,746	0,541	0,903	4832263,965	8595277,187	9540502,601
Q96SL4	GPX7	Glutathione peroxidase 7 OS=Homo sapiens OX=9606 GN=GPX7 PE=1 SV=1	2	0,164	5,386	0,903	1412697,714	296817,3732	315623,2148
Q92643	PIGK	GPI-anchor transamidase OS=Homo sapiens OX=9606 GN=PIGK PE=1 SV=2	3	1,21	0,748	0,902	2245671,505	2287666,671	2601136,858
P42126	ECI1	Enoyl-CoA delta isomerase 1, mitochondrial OS=Homo sapiens OX=9606 GN=ECI1 PE=1 SV=1	5	2,471	0,379	0,901	5556961,094	12844230,83	15231968,87
Q9BUR5	APOO	MICOS complex subunit MIC26 OS=Homo sapiens OX=9606 GN=APOO PE=1 SV=1	4	1,106	0,83	0,901	283803,8173	3168671,364	3164894,007
P61081	UBE2M	NEDD8-conjugating enzyme Ubc12 OS=Homo sapiens OX=9606 GN=UBE2M PE=1 SV=1	4	0,682	1,502	0,9	4841303,111	3277499,151	3016211,497
Q96EE3	SEH1L	Nucleoporin SEH1 OS=Homo sapiens OX=9606 GN=SEH1L PE=1 SV=3	4	0,975	0,922	0,898	2877932,863	2719773,078	4861241,634
Q99547	MPHOSPH6	M-phase phosphoprotein 6 OS=Homo sapiens OX=9606 GN=MPHOSPH6 PE=1 SV=2	2	0,895	0,907	0,898	675712,0846	570648,7818	891444,0088
Q8NC51	SERBP1	SERPINE1 mRNA-binding protein 1 OS=Homo sapiens OX=9606 GN=SERBP1 PE=1 SV=2	17	0,56	1,754	0,898	64517154,7	35318137,49	37059803,05
Q8N3R9	PALS1	Protein PALS1 OS=Homo sapiens OX=9606 GN=PALS1 PE=1 SV=3	3	1,595	0,631	0,897	342033,1914	158383,5104	1025341,193
P20674	COX5A	Cytochrome c oxidase subunit 5A, mitochondrial OS=Homo sapiens OX=9606 GN=COX5A PE=1 S	6	0,324	2,017	0,897	23929164,88	15195032,11	16955805,35
O43752	STX6	Syntaxin-6 OS=Homo sapiens OX=9606 GN=STX6 PE=1 SV=1	6	1,031	1,407	0,896	2604353,182	2324112,617	2371982,915
O43252	PAPSS1	Bifunctional 3'-phosphoadenosine 5'-phosphosulfate synthase 1 OS=Homo sapiens OX=9606 GN	7	0,365	2,302	0,896	4074021,419	1378313,385	1368248,473
P04439	HLA-A	HLA class I histocompatibility antigen, A alpha chain OS=Homo sapiens OX=9606 GN=HLA-A PE=	9	13,48	0,073	0,895	19457615,8	272296975,8	241568562,6
O00264	PGRMC1	Membrane-associated progesterone receptor component 1 OS=Homo sapiens OX=9606 GN=PGF	10	0,265	3,455	0,895	83662743,79	24378660,85	31076023,31
Q86U28	ISCA2	Iron-sulfur cluster assembly 2 homolog, mitochondrial OS=Homo sapiens OX=9606 GN=ISCA2 PE	3	1,926	0,473	0,894	673828,7927	658541,3321	1323952,729
Q96EV2	RBM33	RNA-binding protein 33 OS=Homo sapiens OX=9606 GN=RBM33 PE=1 SV=3	2	0,855	1,067	0,894	367559,447	308182,1435	344607,1126
P28288	ABCD3	ATP-binding cassette sub-family D member 3 OS=Homo sapiens OX=9606 GN=ABCD3 PE=1 SV=1	12	1,213	0,817	0,893	6731981,503	8260556,115	8192810,608
Q9Y6M9	NDUFB9	NADH dehydrogenase [ubiquinone] 1 beta subcomplex subunit 9 OS=Homo sapiens OX=9606 GN	3	1,93	0,502	0,892	1098269,814	2928303,975	3833831,507
P51608	MECP2	Methyl-CpG-binding protein 2 OS=Homo sapiens OX=9606 GN=MECP2 PE=1 SV=1	3	1,868	0,518	0,891	824220,4974	1337740,189	1489100,126
P24752	ACAT1	Acetyl-CoA acetyltransferase, mitochondrial OS=Homo sapiens OX=9606 GN=ACAT1 PE=1 SV=1	14	1,555	0,6	0,891	12342432,16	21102269,73	21585675,25
Q9H3Z4	DNAJC5	DnaJ homolog subfamily C member 5 OS=Homo sapiens OX=9606 GN=DNAJC5 PE=1 SV=1	7	1,42	0,784	0,889	2844276,85	3411273,588	5000312,957
Q15334	LLGL1	Lethal(2) giant larvae protein homolog 1 OS=Homo sapiens OX=9606 GN=LLGL1 PE=1 SV=3	2	0,322	1,955	0,889	2783620,428	624330,6311	1334555,816
P49006	MARCKSL1	MARCKS-related protein OS=Homo sapiens OX=9606 GN=MARCKSL1 PE=1 SV=2	4	0,254	3,687	0,889	36057834,9	9563328,005	10921552,23
Q9Y4K1	CRYBG1	Beta/gamma crystallin domain-containing protein 1 OS=Homo sapiens OX=9606 GN=CRYBG1 PE	20	2,383		0,888	215722,7138	5129407,862	5544996,093
Q04837	SSBP1	Single-stranded DNA-binding protein, mitochondrial OS=Homo sapiens OX=9606 GN=SSBP1 PE=	9	1,319	0,706	0,888	32801642,13	42905732,91	57283426,05
Q9Y281	CFL2	Cofilin-2 OS=Homo sapiens OX=9606 GN=CFL2 PE=1 SV=1	5	0,867	1,822	0,888	63556467,76	16443250,95	25358286,04
P06493	CDK1	Cyclin-dependent kinase 1 OS=Homo sapiens OX=9606 GN=CDK1 PE=1 SV=3	9	0,539	1,852	0,888	21199821,11	8844921,588	8276536,011
P0C0S5	H2AZ1	Histone H2A.Z OS=Homo sapiens OX=9606 GN=H2AZ1 PE=1 SV=2	3	0,904	0,984	0,887	73015643,93	66577720,82	78624907,49
Q99447	PCYT2	Ethanolamine-phosphate cytidylyltransferase OS=Homo sapiens OX=9606 GN=PCYT2 PE=1 SV=1	2	0,849	1,114	0,887	348555,5115	295790,802	333327,7666
O75608	LYPLA1	Acyl-protein thioesterase 1 OS=Homo sapiens OX=9606 GN=LYPLA1 PE=1 SV=1	2	0,743	1,208	0,887	2264096,563	1625811,464	1867362,479
O00506	STK25	Serine/threonine-protein kinase 25 OS=Homo sapiens OX=9606 GN=STK25 PE=1 SV=1	3	0,509	1,573	0,887	2694899,725	3679103,963	2138173,741
P63167	DYNLL1	Dynein light chain 1, cytoplasmic OS=Homo sapiens OX=9606 GN=DYNLL1 PE=1 SV=1	2	0,87	1,008	0,886	6496552,242	5149084,252	6135079,922
O43678	NDUFA2	NADH dehydrogenase [ubiquinone] 1 alpha subcomplex subunit 2 OS=Homo sapiens OX=9606 G	4	1,443	0,656	0,885	2999124,703	5245901,145	6593973,258
P30260	CDC27	Cell division cycle protein 27 homolog OS=Homo sapiens OX=9606 GN=CDC27 PE=1 SV=2	2	0,955	0,927	0,885	626647,1492	564612,0761	659452,2413
Q99496	RNF2	E3 ubiquitin-protein ligase RING2 OS=Homo sapiens OX=9606 GN=RNF2 PE=1 SV=1	2	0,52	1,631	0,885	1404160,813	746657,1844	508618,0468
O75369	FLNB	Filamin-B OS=Homo sapiens OX=9606 GN=FLNB PE=1 SV=2	84	4,382	0,192	0,884	110929517,7	437330487,6	480328454,1
P21980	TGM2	Protein-glutamine gamma-glutamyltransferase 2 OS=Homo sapiens OX=9606 GN=TGM2 PE=1 SV	10	3,968	0,222	0,884	1391787,355	7183656,102	9349020,27
Q9P0M9	MRPL27	Large ribosomal subunit protein bL27m OS=Homo sapiens OX=9606 GN=MRPL27 PE=1 SV=1	2	1,363	0,671	0,884	616615,0763	851162,7585	811124,5426
Q8IX12	CCAR1	Cell division cycle and apoptosis regulator protein 1 OS=Homo sapiens OX=9606 GN=CCAR1 PE=	3	0,812	1,076	0,884	2011230,96	1566865,223	1927671,954
Q15427	SF3B4	Splicing factor 3B subunit 4 OS=Homo sapiens OX=9606 GN=SF3B4 PE=1 SV=1	6	0,576	1,419	0,884	6368852,574	4607076,756	5540279,313
P41252	IARS1	Isoleucine--tRNA ligase, cytoplasmic OS=Homo sapiens OX=9606 GN=IARS1 PE=1 SV=2	24	0,817	1,108	0,883	22707170,94	14101692,15	19830932,4
Q05048	CSTF1	Cleavage stimulation factor subunit 1 OS=Homo sapiens OX=9606 GN=CSTF1 PE=1 SV=1	4	0,806	1,143	0,882	2596225,731	2077819,224	2336075,397
A0A0D9SF53	DDX3X	RNA helicase OS=Homo sapiens OX=9606 GN=DDX3X PE=1 SV=1	18	0,394	1,935	0,882	49511980,99	18923878,12	25060509,07
P23396	RPS3	Small ribosomal subunit protein uS3 OS=Homo sapiens OX=9606 GN=RPS3 PE=1 SV=2	15	0,765	1,23	0,881	130242488,4	93038557,09	115850434,6
P30519	HMOX2	Heme oxygenase 2 OS=Homo sapiens OX=9606 GN=HMOX2 PE=1 SV=2	9	2,58	0,33	0,88	2637834,495	7506163,869	8426669,037
Q93096	PTP4A1	Protein tyrosine phosphatase type IVA 1 OS=Homo sapiens OX=9606 GN=PTP4A1 PE=1 SV=2	3	1,28	1,093	0,88	1503486,073	2278880,131	1883569,706
P09972	ALDOC	Fructose-bisphosphate aldolase C OS=Homo sapiens OX=9606 GN=ALDOC PE=1 SV=2	3	0,31	1,273	0,879	1711768,055	497269,0293	366933,8252
Q9BU61	NDUFAF3	NADH dehydrogenase [ubiquinone] 1 alpha subcomplex assembly factor 3 OS=Homo sapiens OX	3	1,107	0,786	0,878	1192381,239	1364844,132	1586853,265
P61978	HNRNPK	Heterogeneous nuclear ribonucleoprotein K OS=Homo sapiens OX=9606 GN=HNRNPK PE=1 SV=	18	0,815	1,191	0,878	306054697,4	238641278,3	266418645,9
Q16576	RBBP7	Histone-binding protein RBBP7 OS=Homo sapiens OX=9606 GN=RBBP7 PE=1 SV=1	5	0,758	1,456	0,878	36075360,98	18121413,83	23809413,56
P26572	MGAT1	Alpha-1,3-mannosyl-glycoprotein 2-beta-N-acetylglucosaminyltransferase OS=Homo sapiens OX	2	2,059	0,407	0,877	497669,7254	998208,184	1068919,763
Q5T653	MRPL2	Large ribosomal subunit protein uL2m OS=Homo sapiens OX=9606 GN=MRPL2 PE=1 SV=2	2	1,278	0,608	0,877	1344112,941	1469940,97	1693025,591
P31942	HNRNPH3	Heterogeneous nuclear ribonucleoprotein H3 OS=Homo sapiens OX=9606 GN=HNRNPH3 PE=1 S	10	0,785	1,02	0,877	56802581,39	44670318,65	60665168,21
Q9BUQ8	DDX23	Probable ATP-dependent RNA helicase DDX23 OS=Homo sapiens OX=9606 GN=DDX23 PE=1 SV=	7	0,722	1,206	0,877	4270061,991	1940766,971	4074300,302
Q9NTJ5	SACM1L	Phosphatidylinositol-3-phosphatase SAC1 OS=Homo sapiens OX=9606 GN=SACM1L PE=1 SV=2	17	1,198	0,788	0,875	5340151,756	7361538,799	6982422,3
P36542	ATP5F1C	ATP synthase subunit gamma, mitochondrial OS=Homo sapiens OX=9606 GN=ATP5F1C PE=1 SV=	7	1,169	0,788	0,875	20786399,57	23783332,77	26230867
O95470	SGPL1	Sphingosine-1-phosphate lyase 1 OS=Homo sapiens OX=9606 GN=SGPL1 PE=1 SV=3	9	0,989	0,861	0,875	5579321,039	5464882,793	7145339,883
P54652	HSPA2	Heat shock-related 70 kDa protein 2 OS=Homo sapiens OX=9606 GN=HSPA2 PE=1 SV=1	13	0,629	1,464	0,874	437878023,3	287112947,1	337896580,6
Q02252	ALDH6A1	Methylmalonate-semialdehyde/malonate-semialdehyde dehydrogenase [acylating], mitochondr	4	1,748	0,494	0,872	1031135,657	1816519,285	1989399,084
P62491	RAB11A	Ras-related protein Rab-11A OS=Homo sapiens OX=9606 GN=RAB11A PE=1 SV=3	8	1,022	0,87	0,872	35375157,03	35297219,45	38380599,98
Q9UBL3	ASH2L	Set1/Ash2 histone methyltransferase complex subunit ASH2 OS=Homo sapiens OX=9606 GN=AS	4	0,861	1,032	0,872	1873825,924	1621891,109	1898425,549
Q14108	SCARB2	Lysosome membrane protein 2 OS=Homo sapiens OX=9606 GN=SCARB2 PE=1 SV=2	7	2,545	0,349	0,871	3041625,919	7619336,91	7802540,811
Q7Z5K2	WAPL	Wings apart-like protein homolog OS=Homo sapiens OX=9606 GN=WAPL PE=1 SV=1	2	1,06	0,786	0,871	155158,2032	176270,3437	202481,9841
Q9Y2Z0	SUGT1	Protein SGT1 homolog OS=Homo sapiens OX=9606 GN=SUGT1 PE=1 SV=3	9	0,349	2,265	0,871	14322900,2	4020667,941	5950142,367
P62760	VSNL1	Visinin-like protein 1 OS=Homo sapiens OX=9606 GN=VSNL1 PE=1 SV=2	2	1,952	0,446	0,87	354591,8478	1160082,418	795287,798
P55263	ADK	Adenosine kinase OS=Homo sapiens OX=9606 GN=ADK PE=1 SV=2	4	1,076	1	0,87	2101208,335	1381499,803	2253868,895
Q96DI7	SNRNP40	U5 small nuclear ribonucleoprotein 40 kDa protein OS=Homo sapiens OX=9606 GN=SNRNP40 PE	7	0,671	1,088	0,87	5248703,25	3501914,753	3672645,232
P52732	KIF11	Kinesin-like protein KIF11 OS=Homo sapiens OX=9606 GN=KIF11 PE=1 SV=2	8	0,62	1,516	0,87	4085054,27	2576077,54	2639580,962
P34932	HSPA4	Heat shock 70 kDa protein 4 OS=Homo sapiens OX=9606 GN=HSPA4 PE=1 SV=4	23	0,503	1,815	0,87	78905719,14	38502869,62	46031232,57
O43708	GSTZ1	Maleylacetoacetate isomerase OS=Homo sapiens OX=9606 GN=GSTZ1 PE=1 SV=3	2	1,277	0,82	0,869	488883,3073	473402,386	646404,5094
Q9UKR5	ERG28	Ergosterol biosynthetic protein 28 homolog OS=Homo sapiens OX=9606 GN=ERG28 PE=1 SV=1	3	0,523	1,621	0,869	2626753,69	1434464,793	1626669,365
P46776	RPL27A	Large ribosomal subunit protein uL15 OS=Homo sapiens OX=9606 GN=RPL27A PE=1 SV=2	3	0,517	1,366	0,869	6341461,344	4205083,219	7392251,867
Q6L8Q7	PDE12	2',5'-phosphodiesterase 12 OS=Homo sapiens OX=9606 GN=PDE12 PE=1 SV=2	6	0,789	1,129	0,868	2051139,429	1706718,867	1884683,883
P07738	BPGM	Bisphosphoglycerate mutase OS=Homo sapiens OX=9606 GN=BPGM PE=1 SV=2	3	0,769	1,405	0,868	320443,1057	292560,6699	120852,4707
P37198	NUP62	Nuclear pore glycoprotein p62 OS=Homo sapiens OX=9606 GN=NUP62 PE=1 SV=3	8	1,085	0,809	0,867	6122808,582	4646138,105	7152928,345
Q01085	TIAL1	Nucleolysin TIAR OS=Homo sapiens OX=9606 GN=TIAL1 PE=1 SV=1	5	0,675	1,156	0,867	8875119,718	5480966,028	7835548,372
Q9UJV9	DDX41	Probable ATP-dependent RNA helicase DDX41 OS=Homo sapiens OX=9606 GN=DDX41 PE=1 SV=	3	0,527	1,264	0,866	1314710,164	753428,2727	1129194,067
Q5T7N2	L1TD1	LINE-1 type transposase domain-containing protein 1 OS=Homo sapiens OX=9606 GN=L1TD1 PE	19	0,07	10,954	0,866	34190289,11	1194785,741	776888,5157
P38571	LIPA	Lysosomal acid lipase/cholesteryl ester hydrolase OS=Homo sapiens OX=9606 GN=LIPA PE=1 SV=	4	2,198	2,687	0,865	2192549,971	3841365,645	604927,3881
O00233	PSMD9	26S proteasome non-ATPase regulatory subunit 9 OS=Homo sapiens OX=9606 GN=PSMD9 PE=1	2	0,891	0,978	0,864	1627963,808	1469452,452	1721607,069

P62826	RAN	GTP-binding nuclear protein Ran OS=Homo sapiens OX=9606 GN=RAN PE=1 SV=3	9	0,378	2,437	0,864	139220449,8	51040331,23	56820955,93
Q14974	KPNB1	Importin subunit beta-1 OS=Homo sapiens OX=9606 GN=KPNB1 PE=1 SV=2	20	0,772	1,084	0,863	74585128,66	50457213,3	66322284,66
O95983	MBD3	Methyl-CpG-binding domain protein 3 OS=Homo sapiens OX=9606 GN=MBD3 PE=1 SV=1	4	0,332	2,539	0,863	3944468,508	1064903,21	2323066,959
Q27J81	INF2	Inverted formin-2 OS=Homo sapiens OX=9606 GN=INF2 PE=1 SV=2	22	3,65	0,227	0,862	1633325,998	8505212,609	10258632,42
Q9C0B5	ZDHHC5	Palmitoyltransferase ZDHHC5 OS=Homo sapiens OX=9606 GN=ZDHHC5 PE=1 SV=2	2	1,955	0,392	0,862	278948,0722	870730,6722	1032146,13
Q8NFH3	NUP43	Nucleoporin Nup43 OS=Homo sapiens OX=9606 GN=NUP43 PE=1 SV=1	4	1,027	0,837	0,862	3390377,706	4675734,061	4574404,329
P14868	DARS1	Aspartate--tRNA ligase, cytoplasmic OS=Homo sapiens OX=9606 GN=DARS1 PE=1 SV=2	20	0,902	0,936	0,862	24922847,86	22903183,76	29395342,51
Q9Y6D6	ARFGEF1	Brefeldin A-inhibited guanine nucleotide-exchange protein 1 OS=Homo sapiens OX=9606 GN=AR	3	0,864	1,357	0,862	416907,8377	139761,864	208534,9361
Q9Y5J9	TIMM8B	Mitochondrial import inner membrane translocase subunit Tim8 B OS=Homo sapiens OX=9606 G	2	0,863	0,992	0,862	461982,8235	409267,7947	474524,7214
P40937	RFC5	Replication factor C subunit 5 OS=Homo sapiens OX=9606 GN=RFC5 PE=1 SV=1	5	0,606	1,419	0,862	4454326,164	1363890,534	2483297,628
Q6KC79	NIPBL	Nipped-B-like protein OS=Homo sapiens OX=9606 GN=NIPBL PE=1 SV=2	5	0,57	1,524	0,862	1226647,364	644781,2154	933578,9101
P40616	ARL1	ADP-ribosylation factor-like protein 1 OS=Homo sapiens OX=9606 GN=ARL1 PE=1 SV=1	8	2,125	0,371	0,861	2568591,01	5352016,936	7208792,89
P82663	MRPS25	Small ribosomal subunit protein mS25 OS=Homo sapiens OX=9606 GN=MRPS25 PE=1 SV=1	3	1,282	0,829	0,861	430145,0143	436798,8668	774343,4234
P30825	SLC7A1	High affinity cationic amino acid transporter 1 OS=Homo sapiens OX=9606 GN=SLC7A1 PE=1 SV=	2	0,867	1,011	0,861	674109,4407	1258056,566	1509419,827
Q15056	EIF4H	Eukaryotic translation initiation factor 4H OS=Homo sapiens OX=9606 GN=EIF4H PE=1 SV=5	6	0,42	1,677	0,861	20226789,06	9718947,349	12593270,67
P16278	GLB1	Beta-galactosidase OS=Homo sapiens OX=9606 GN=GLB1 PE=1 SV=2	6	2,476	0,372	0,86	2509796,649	5432187,542	6785453,275
P51114	FXR1	RNA-binding protein FXR1 OS=Homo sapiens OX=9606 GN=FXR1 PE=1 SV=3	10	0,793	1,047	0,86	13294748,8	11544414,77	12729146,78
P15880	RPS2	Small ribosomal subunit protein uS5 OS=Homo sapiens OX=9606 GN=RPS2 PE=1 SV=2	11	0,703	1,25	0,86	64962634,92	34507811,81	51509020,21
Q96HE7	ERO1A	ERO1-like protein alpha OS=Homo sapiens OX=9606 GN=ERO1A PE=1 SV=2	12	2,995	0,289	0,858	6808837,544	19614711,92	23616363,24
P38646	HSPA9	Stress-70 protein, mitochondrial OS=Homo sapiens OX=9606 GN=HSPA9 PE=1 SV=2	27	1,113	0,742	0,858	192861432,9	219418644,5	289899086,7
Q3MHD2	LSM12	Protein LSM12 OS=Homo sapiens OX=9606 GN=LSM12 PE=1 SV=2	3	0,852	1,033	0,858	2133621,627	1775411,317	2501139,047
O75792	RNASEH2A	Ribonuclease H2 subunit A OS=Homo sapiens OX=9606 GN=RNASEH2A PE=1 SV=2	2	0,768	1,149	0,858	643204,1095	509210,0668	551421,393
Q9BQ39	DDX50	ATP-dependent RNA helicase DDX50 OS=Homo sapiens OX=9606 GN=DDX50 PE=1 SV=1	5	0,414	2,001	0,858	1472759,899	781767,9173	1000939,944
Q9BS26	ERP44	Endoplasmic reticulum resident protein 44 OS=Homo sapiens OX=9606 GN=ERP44 PE=1 SV=1	11	2,745	0,311	0,857	10482073,51	28978460,09	35961268,12
Q13155	AIMP2	Aminoacyl tRNA synthase complex-interacting multifunctional protein 2 OS=Homo sapiens OX=9	4	0,704	1,637	0,857	1453524,899	1421283,578	2202686,482
Q9H0D6	XRN2	5'-3' exoribonuclease 2 OS=Homo sapiens OX=9606 GN=XRN2 PE=1 SV=1	15	0,543	1,32	0,857	18708321,87	10499124,36	14759028,68
Q969M3	YIPF5	Protein YIPF5 OS=Homo sapiens OX=9606 GN=YIPF5 PE=1 SV=1	2	1,831	0,475	0,856	1554394,115	3382718,115	2482641,604
Q15120	PDK3	[Pyruvate dehydrogenase (acetyl-transferring)] kinase isozyme 3, mitochondrial OS=Homo sapie	3	0,333	2,095	0,856	439757,6207	118142,1325	276444,9574
Q96RQ9	IL4I1	L-amino-acid oxidase OS=Homo sapiens OX=9606 GN=IL4I1 PE=1 SV=1	3	0,394	2,598	0,854	413470,6718	162954,2463	194928,5383
Q9UKG9	CROT	Peroxisomal carnitine O-octanoyltransferase OS=Homo sapiens OX=9606 GN=CROT PE=1 SV=2	5	2,56	0,299	0,853	264241,2092	515711,0757	679819,5041
P13984	GTF2F2	General transcription factor IIF subunit 2 OS=Homo sapiens OX=9606 GN=GTF2F2 PE=1 SV=2	2	0,613	1,391	0,853	681175,2538	417486,2715	489551,5203
Q16822	PCK2	Phosphoenolpyruvate carboxykinase [GTP], mitochondrial OS=Homo sapiens OX=9606 GN=PCK2	20	3,407	0,245	0,852	7510416,826	29762336,54	35053671,31
O60287	URB1	Nucleolar pre-ribosomal-associated protein 1 OS=Homo sapiens OX=9606 GN=URB1 PE=1 SV=4	3	2,392	0,272	0,852	608743,7244	1414762,311	692222,8288
Q71RG4	TMUB2	Transmembrane and ubiquitin-like domain-containing protein 2 OS=Homo sapiens OX=9606 GN=	2	0,616	1,319	0,852	1036570,608	638313,327	1448879,504
O60256	PRPSAP2	Phosphoribosyl pyrophosphate synthase-associated protein 2 OS=Homo sapiens OX=9606 GN=P	3	0,172	4,624	0,852	1946616,803	192469,2913	233241,4071
L0R6Q1	SLC35A4	SLC35A4 upstream open reading frame protein OS=Homo sapiens OX=9606 GN=SLC35A4 PE=3 S'	7	1,501	0,587	0,851	3320470,074	7543352,414	8999387,962
O15514	POLR2D	DNA-directed RNA polymerase II subunit RPB4 OS=Homo sapiens OX=9606 GN=POLR2D PE=1 SV	3	0,734	1,206	0,851	1651773,355	1181962,541	1580854,475
O75251	NDUFS7	NADH dehydrogenase [ubiquinone] iron-sulfur protein 7, mitochondrial OS=Homo sapiens OX=9	5	1,174	0,624	0,85	1809579,416	2199172,07	3085404,204
O94992	HEXIM1	Protein HEXIM1 OS=Homo sapiens OX=9606 GN=HEXIM1 PE=1 SV=1	4	1,637	0,543	0,849	531029,5666	878038,915	967495,8114
Q7KZF4	SND1	Staphylococcal nuclease domain-containing protein 1 OS=Homo sapiens OX=9606 GN=SND1 PE=	31	0,556	1,483	0,849	75379344,8	43207072,62	52288746,29
P16298	PPP3CB	Serine/threonine-protein phosphatase 2B catalytic subunit beta isoform OS=Homo sapiens OX=9	2	1,048	0,751	0,848	2550453,919	2700886,457	3738805,122
Q9Y266	NUDC	Nuclear migration protein nudC OS=Homo sapiens OX=9606 GN=NUDC PE=1 SV=1	10	0,362	2,581	0,848	43150359,63	14352122,33	17226161,52
Q99798	ACO2	Aconitate hydratase, mitochondrial OS=Homo sapiens OX=9606 GN=ACO2 PE=1 SV=2	33	1,612	0,55	0,847	40536933,54	66495118,02	73324031,41
Q8IYB8	SUPV3L1	ATP-dependent RNA helicase SUPV3L1, mitochondrial OS=Homo sapiens OX=9606 GN=SUPV3L1	4	1,011	0,897	0,847	778954,8961	738003,9909	1683176,24
P40222	TXLNA	Alpha-taxilin OS=Homo sapiens OX=9606 GN=TXLNA PE=1 SV=3	6	0,759	1,061	0,847	4655967,225	3633561,613	4792224,198
Q96EP5	DAZAP1	DAZ-associated protein 1 OS=Homo sapiens OX=9606 GN=DAZAP1 PE=1 SV=1	2	0,724	1,078	0,847	5832396,153	5252577,39	6304201,098
P25398	RPS12	Small ribosomal subunit protein eS12 OS=Homo sapiens OX=9606 GN=RPS12 PE=1 SV=3	3	0,718	1,084	0,846	24673790,77	16658367,86	20103496,73
Q9BZZ5	API5	Apoptosis inhibitor 5 OS=Homo sapiens OX=9606 GN=API5 PE=1 SV=3	10	0,697	1,238	0,846	24603031,34	17347503,11	20629441,76
Q96FQ6	S100A16	Protein S100-A16 OS=Homo sapiens OX=9606 GN=S100A16 PE=1 SV=1	3	1000	0,001	0,845		3255055,653	3621024,121
Q6PIU2	NCEH1	Neutral cholesterol ester hydrolase 1 OS=Homo sapiens OX=9606 GN=NCEH1 PE=1 SV=3	8	7,037	0,119	0,845	983323,6418	6666772,84	7609311,679
Q6IQ22	RAB12	Ras-related protein Rab-12 OS=Homo sapiens OX=9606 GN=RAB12 PE=1 SV=3	3	1,846	0,489	0,845	892389,9871	2323481,224	1937759,855
P40926	MDH2	Malate dehydrogenase, mitochondrial OS=Homo sapiens OX=9606 GN=MDH2 PE=1 SV=3	10	1,055	0,843	0,845	155627310,6	152408954,3	177527230
Q9BYT8	NLN	Neurolysin, mitochondrial OS=Homo sapiens OX=9606 GN=NLN PE=1 SV=1	9	0,385	2,423	0,845	6446006,202	2283641,033	2571439,263
Q96G42	KLHDC7B	Kelch domain-containing protein 7B OS=Homo sapiens OX=9606 GN=KLHDC7B PE=1 SV=2	4	0,738	1,364	0,844	414659,2978	305862,9981	401376,3239
Q86X55	CARM1	Histone-arginine methyltransferase CARM1 OS=Homo sapiens OX=9606 GN=CARM1 PE=1 SV=3	3	0,528	1,458	0,844	903713,4582	521214,5312	606297,0351
Q9NZL4	HSPBP1	Hsp70-binding protein 1 OS=Homo sapiens OX=9606 GN=HSPBP1 PE=1 SV=2	2	0,988	0,866	0,843	1136003,615	1098284,732	1241507,997
Q9UKX7	NUP50	Nuclear pore complex protein Nup50 OS=Homo sapiens OX=9606 GN=NUP50 PE=1 SV=2	10	0,825	0,86	0,843	6527859,555	5673311,441	7736109,455
P61962	DCAF7	DDB1- and CUL4-associated factor 7 OS=Homo sapiens OX=9606 GN=DCAF7 PE=1 SV=1	4	0,363	2,631	0,843	4414964,239	1449701,749	1500970,78
Q9BXF6	RAB11FIP5	Rab11 family-interacting protein 5 OS=Homo sapiens OX=9606 GN=RAB11FIP5 PE=1 SV=1	2	1000	0,001	0,842		532589,4487	629081,012
Q9BYK8	HELZ2	3'-5' exoribonuclease HELZ2 OS=Homo sapiens OX=9606 GN=HELZ2 PE=1 SV=7	13	12,543	0,079	0,842	185418,3429	3696466,075	6410015,237
P09211	GSTP1	Glutathione S-transferase P OS=Homo sapiens OX=9606 GN=GSTP1 PE=1 SV=2	11	0,19	4,633	0,842	163828830,2	29594533,96	35915153,53
Q7Z2K8	GPRIN1	G protein-regulated inducer of neurite outgrowth 1 OS=Homo sapiens OX=9606 GN=GPRIN1 PE=	7	1,704	0,532	0,841	2088594,347	2971688,053	8166237,704
Q14781	CBX2	Chromobox protein homolog 2 OS=Homo sapiens OX=9606 GN=CBX2 PE=1 SV=2	2	0,503	1,624	0,841	739609,501	397077,7976	508382,0338
P22102	GART	Trifunctional purine biosynthetic protein adenosine-3 OS=Homo sapiens OX=9606 GN=GART PE=	19	0,379	2,38	0,841	18231682,22	6691432,019	7577056,022
Q9UI09	NDUFA12	NADH dehydrogenase [ubiquinone] 1 alpha subcomplex subunit 12 OS=Homo sapiens OX=9606 (	3	1,117	0,695	0,84	1371704,571	1829872,55	2412278,972
P30038	ALDH4A1	Delta-1-pyrroline-5-carboxylate dehydrogenase, mitochondrial OS=Homo sapiens OX=9606 GN=/	13	0,578	1,276	0,84	4560161,375	3082491,827	4343255,331
Q96T88	UHRF1	E3 ubiquitin-protein ligase UHRF1 OS=Homo sapiens OX=9606 GN=UHRF1 PE=1 SV=1	13	0,402	2,139	0,84	8875838,153	1979719,7	3920870,196
Q9H553	ALG2	Alpha-1,3/1,6-mannosyltransferase ALG2 OS=Homo sapiens OX=9606 GN=ALG2 PE=1 SV=1	5	4,221	0,189	0,839	789627,7341	2935531,074	4131604,484
P59998	ARPC4	Actin-related protein 2/3 complex subunit 4 OS=Homo sapiens OX=9606 GN=ARPC4 PE=1 SV=3	5	1,271	0,692	0,839	34177389,14	38130125,25	46046655,04
P00367	GLUD1	Glutamate dehydrogenase 1, mitochondrial OS=Homo sapiens OX=9606 GN=GLUD1 PE=1 SV=2	18	1,189	0,947	0,838	38244820,66	44314631,51	43493730,5
Q53EL6	PDCD4	Programmed cell death protein 4 OS=Homo sapiens OX=9606 GN=PDCD4 PE=1 SV=2	4	1,069	0,903	0,837	1939278,822	1428907,504	2085242,755
P14866	HNRNPL	Heterogeneous nuclear ribonucleoprotein L OS=Homo sapiens OX=9606 GN=HNRNPL PE=1 SV=2	19	0,97	0,901	0,837	77246117,54	66571381,83	81449834,7
Q05209	PTPN12	Tyrosine-protein phosphatase non-receptor type 12 OS=Homo sapiens OX=9606 GN=PTPN12 PE=	2	0,394	2,13	0,837	516549,6737	203404,4843	242459,7863
Q15102	PAFAH1B3	Platelet-activating factor acetylhydrolase IB subunit alpha1 OS=Homo sapiens OX=9606 GN=PAF	7	0,334	2,238	0,837	10200110,87	2923920,575	3341761,89
P80217	IFI35	Interferon-induced 35 kDa protein OS=Homo sapiens OX=9606 GN=IFI35 PE=1 SV=5	6	1000	0,001	0,836		1201848,882	2345772,341
P51531	SMARCA2	Probable global transcription activator SNF2L2 OS=Homo sapiens OX=9606 GN=SMARCA2 PE=1 S	5	0,383	2,988	0,836	6864515,975	4641465,472	2138835,326
Q13085	ACACA	Acetyl-CoA carboxylase 1 OS=Homo sapiens OX=9606 GN=ACACA PE=1 SV=2	13	0,266	4,605	0,836	12387688,22	7413360,275	2939852,107
Q8IUH4	ZDHHC13	Palmitoyltransferase ZDHHC13 OS=Homo sapiens OX=9606 GN=ZDHHC13 PE=1 SV=3	2	2,52	0,351	0,835	288760,4223	1343714,161	1826259,612
Q9Y5L4	TIMM13	Mitochondrial import inner membrane translocase subunit Tim13 OS=Homo sapiens OX=9606 G	3	0,968	0,864	0,835	5385678,803	5334140,619	6411579,26
Q13838	DDX39B	Spliceosome RNA helicase DDX39B OS=Homo sapiens OX=9606 GN=DDX39B PE=1 SV=1	8	0,755	1,226	0,835	95855652,33	65443327,62	84535186,83
Q9H2U1	DHX36	ATP-dependent DNA/RNA helicase DHX36 OS=Homo sapiens OX=9606 GN=DHX36 PE=1 SV=2	2	0,717	1,092	0,835	1617868,798	1149358,488	1644063,379
O60610	DIAPH1	Protein diaphanous homolog 1 OS=Homo sapiens OX=9606 GN=DIAPH1 PE=1 SV=2	17	0,601	1,301	0,835	10879566,95	7109117,101	8497448,928
Q86T03	PIP4P1	Type 1 phosphatidylinositol 4,5-bisphosphate 4-phosphatase OS=Homo sapiens OX=9606 GN=PI	2	0,93	0,964	0,834	567771,858	509293,4583	686576,7106
Q96RP9	GFM1	Elongation factor G, mitochondrial OS=Homo sapiens OX=9606 GN=GFM1 PE=1 SV=2	12	1,822	0,45	0,833	6204216,997	11699540,2	14093537,19
Q96QR8	PURB	Transcriptional activator protein Pur-beta OS=Homo sapiens OX=9606 GN=PURB PE=1 SV=3	3	1,615	0,495	0,833	1194150,767	1915455,43	2185349,819
O75352	MPDU1	Mannose-P-dolichol utilization defect 1 protein OS=Homo sapiens OX=9606 GN=MPDU1 PE=1 S\	3	1,246	0,729	0,832	1368431,2	2006312,969	2177781,928
Q9BW27	NUP85	Nuclear pore complex protein Nup85 OS=Homo sapiens OX=9606 GN=NUP85 PE=1 SV=1	10	0,955	0,855	0,832	6192252,016	5203683,408	7236322,319
Q9NQX7	ITM2C	Integral membrane protein 2C OS=Homo sapiens OX=9606 GN=ITM2C PE=1 SV=1	2	0,125	5,24	0,832	1271674,24	71150,96628	239038,0745
O60216	RAD21	Double-strand-break repair protein rad21 homolog OS=Homo sapiens OX=9606 GN=RAD21 PE=1	10	0,888	0,92	0,831	5798925,408	5986336,706	8337724,502
Q9P2D1	CHD7	Chromodomain-helicase-DNA-binding protein 7 OS=Homo sapiens OX=9606 GN=CHD7 PE=1 SV=	5	0,23	2,556	0,831	1161925,373	107926,6419	227484,6629
P83111	LACTB	Serine beta-lactamase-like protein LACTB, mitochondrial OS=Homo sapiens OX=9606 GN=LACTB	12	4,013	0,224	0,83	3446413,511	8076403,487	17685055
Q7RTP0	NIPA1	Magnesium transporter NIPA1 OS=Homo sapiens OX=9606 GN=NIPA1 PE=1 SV=1	2	1000	0,001	0,829		168849,253	180081,0107
P11182	DBT	Lipoamide acyltransferase component of branched-chain alpha-keto acid dehydrogenase comple	3	1,19	0,619	0,829	446327,9981	875124,461	1105204,301
Q15003	NCAPH	Condensin complex subunit 2 OS=Homo sapiens OX=9606 GN=NCAPH PE=1 SV=3	2	0,71	1,211	0,829	2395631,172	1746180,33	1915901,375
Q7Z739	YTHDF3	YTH domain-containing family protein 3 OS=Homo sapiens OX=9606 GN=YTHDF3 PE=1 SV=1	3	0,654	1,421	0,829	4999451,684	3429952,295	3689489,695
P19525	EIF2AK2	Interferon-induced, double-stranded RNA-activated protein kinase OS=Homo sapiens OX=9606 (	13	1,899	0,412	0,828	7701458,001	12340290,84	19077830,41
Q14247	CTTN	Src substrate cortactin OS=Homo sapiens OX=9606 GN=CTTN PE=1 SV=2	17	1,653	0,514	0,828	21176182,46	32799956,64	46628809,3
P54727	RAD23B	UV excision repair protein RAD23 homolog B OS=Homo sapiens OX=9606 GN=RAD23B PE=1 SV=1	11	1,013	0,791	0,828	18992672,54	19207986,32	23000124,81
Q9NSD9	FARSB	Phenylalanine--tRNA ligase beta subunit OS=Homo sapiens OX=9606 GN=FARSB PE=1 SV=3	13	0,661	1,229	0,828	12720054,12	5801879,956	10057612,03
Q00403	GTF2B	Transcription initiation factor IIB OS=Homo sapiens OX=9606 GN=GTF2B PE=1 SV=1	4	1,012	0,926	0,827	3050428,524	2195724,383	3426112,408
Q9H4L4	SENP3	Sentrin-specific protease 3 OS=Homo sapiens OX=9606 GN=SENP3 PE=1 SV=2	2	0,887	0,916	0,827	1505001,342	1461376,422	1402466,243
P33316	DUT	Deoxyuridine 5'-triphosphate nucleotidohydrolase, mitochondrial OS=Homo sapiens OX=9606 GI	7	0,625	1,125	0,827	9007927,036	5928305,972	7718683,497
Q9Y3C6	PPIL1	Peptidyl-prolyl cis-trans isomerase-like 1 OS=Homo sapiens OX=9606 GN=PPIL1 PE=1 SV=1	3	0,522	1,667	0,827	1650055,369	917919,2559	1175979,96
P10321	HLA-C	HLA class I histocompatibility antigen, C alpha chain OS=Homo sapiens OX=9606 GN=HLA-C PE=1	3	3,048	0,26	0,826	2091484,074	3331534,002	4493973,773
Q9H0E2	TOLLIP	Toll-interacting protein OS=Homo sapiens OX=9606 GN=TOLLIP PE=1 SV=1	2	2,13	0,513	0,826	825606,2979	818925,084	1653664,089
P36957	DLST	Dihydrolipoyllysine-residue succinyltransferase component of 2-oxoglutarate dehydrogenase cor	13	1,261	0,644	0,826	25303373,02	30896148,08	39968536,64
Q8IWT6	LRRC8A	Volume-regulated anion channel subunit LRRC8A OS=Homo sapiens OX=9606 GN=LRRC8A PE=1	6	1,077	0,692	0,826	1911826,889	2627780,69	2651677,101
P63173	RPL38	Large ribosomal subunit protein eL38 OS=Homo sapiens OX=9606 GN=RPL38 PE=1 SV=2	6	0,888	0,835	0,826	29047119,89	24059729,76	32953966,39
P63244	RACK1	Small ribosomal subunit protein RACK1 OS=Homo sapiens OX=9606 GN=RACK1 PE=1 SV=3	9	0,839	1,091	0,826	79084958,26	60018716,54	70570623,38
P61221	ABCE1	ATP-binding cassette sub-family E member 1 OS=Homo sapiens OX=9606 GN=ABCE1 PE=1 SV=1	10	0,608	1,234	0,826	7775622,047	5200360,483	6497887,066
Q9NR28	DIABLO	Diablo IAP-binding mitochondrial protein OS=Homo sapiens OX=9606 GN=DIABLO PE=1 SV=1	9	0,554	1,327	0,826	11128168,62	6615416,146	8648573,976

P56589	PEX3	Peroxisomal biogenesis factor 3 OS=Homo sapiens OX=9606 GN=PEX3 PE=1 SV=1	3	1,58	0,416	0,825	875771,9625	1400832,32	2075655,038
P49821	NDUFV1	NADH dehydrogenase [ubiquinone] flavoprotein 1, mitochondrial OS=Homo sapiens OX=9606 G	10	1,297	0,567	0,825	7087376,222	7420659,565	12070085,28
Q9GZP9	DERL2	Derlin-2 OS=Homo sapiens OX=9606 GN=DERL2 PE=1 SV=1	2	1,079	0,694	0,825	794504,3283	790041,3505	1418765,665
O95239	KIF4A	Chromosome-associated kinesin KIF4A OS=Homo sapiens OX=9606 GN=KIF4A PE=1 SV=3	4	0,347	1,634	0,825	2114307,437	1090258,847	1304016,603
Q96I24	FUBP3	Far upstream element-binding protein 3 OS=Homo sapiens OX=9606 GN=FUBP3 PE=1 SV=2	13	1,312	0,623	0,824	12778645,13	15120380,56	19378712,38
A0AVT1	UBA6	Ubiquitin-like modifier-activating enzyme 6 OS=Homo sapiens OX=9606 GN=UBA6 PE=1 SV=1	7	1,109	0,674	0,824	1935828,073	2221887,121	3427693,616
Q96PZ0	PUS7	Pseudouridylate synthase 7 homolog OS=Homo sapiens OX=9606 GN=PUS7 PE=1 SV=2	6	0,829	1,105	0,824	2199129,235	631463,4682	1877811,23
Q86W42	THOC6	THO complex subunit 6 homolog OS=Homo sapiens OX=9606 GN=THOC6 PE=1 SV=1	5	0,566	1,648	0,824	4331355,542	2323780,629	2247502,494
Q9H9J2	MRPL44	Large ribosomal subunit protein mL44 OS=Homo sapiens OX=9606 GN=MRPL44 PE=1 SV=1	7	1,248	0,545	0,823	1916265,909	2386263,315	3757923,394
O00203	AP3B1	AP-3 complex subunit beta-1 OS=Homo sapiens OX=9606 GN=AP3B1 PE=1 SV=3	3	1,055	1,405	0,823	862836,9951	729423,4711	814200,2225
P62241	RPS8	Small ribosomal subunit protein eS8 OS=Homo sapiens OX=9606 GN=RPS8 PE=1 SV=2	6	0,519	1,443	0,823	30151576,39	16223919,83	20104614,42
O95347	SMC2	Structural maintenance of chromosomes protein 2 OS=Homo sapiens OX=9606 GN=SMC2 PE=1 S	9	0,747	1,004	0,822	4709822,254	2439610,186	4228826,566
P46108	CRK	Adapter molecule crk OS=Homo sapiens OX=9606 GN=CRK PE=1 SV=2	8	0,527	1,715	0,822	4933003,19	1894720,282	3855573,251
Q15005	SPCS2	Signal peptidase complex subunit 2 OS=Homo sapiens OX=9606 GN=SPCS2 PE=1 SV=3	7	1,595	0,476	0,821	11191195,43	13317226,07	17240699,54
O15031	PLXNB2	Plexin-B2 OS=Homo sapiens OX=9606 GN=PLXNB2 PE=1 SV=3	20	1,935	0,467	0,82	4806226,652	11259299,17	14200175,14
P41091	EIF2S3	Eukaryotic translation initiation factor 2 subunit 3 OS=Homo sapiens OX=9606 GN=EIF2S3 PE=1 S	12	0,878	0,959	0,819	15405757,03	13142547,99	15439497,79
Q5T160	RARS2	Probable arginine--tRNA ligase, mitochondrial OS=Homo sapiens OX=9606 GN=RARS2 PE=1 SV=1	3	1,44	0,559	0,818	405777,6712	525914,1913	696536,3751
P08559	PDHA1	Pyruvate dehydrogenase E1 component subunit alpha, somatic form, mitochondrial OS=Homo s	14	0,865	1,049	0,818	11084972,31	9770009,287	10327688,65
Q8WX92	NELFB	Negative elongation factor B OS=Homo sapiens OX=9606 GN=NELFB PE=1 SV=1	6	0,853	1,009	0,818	2834553,478	932010,4011	2180235,323
Q13151	HNRNPA0	Heterogeneous nuclear ribonucleoprotein A0 OS=Homo sapiens OX=9606 GN=HNRNPA0 PE=1 S	6	0,344	1,935	0,818	70096085,28	35147534,79	38795087,45
P67812	SEC11A	Signal peptidase complex catalytic subunit SEC11A OS=Homo sapiens OX=9606 GN=SEC11A PE=1	6	1,641	0,439	0,817	5551109,582	9813972,443	10631358,58
P36578	RPL4	Large ribosomal subunit protein uL4 OS=Homo sapiens OX=9606 GN=RPL4 PE=1 SV=5	12	0,797	1,193	0,817	64554569,56	43447221,84	60460266,99
Q07020	RPL18	Large ribosomal subunit protein eL18 OS=Homo sapiens OX=9606 GN=RPL18 PE=1 SV=2	6	0,803	1,133	0,816	42229056,8	29164111,63	39037854,99
Q14008	CKAP5	Cytoskeleton-associated protein 5 OS=Homo sapiens OX=9606 GN=CKAP5 PE=1 SV=3	24	0,761	1,047	0,816	17941676,83	12510907,4	15764911,49
P82664	MRPS10	Small ribosomal subunit protein uS10m OS=Homo sapiens OX=9606 GN=MRPS10 PE=1 SV=2	2	2,14	0,274	0,815	90287,77106	720839,8145	817338,217
Q8WUM0	NUP133	Nuclear pore complex protein Nup133 OS=Homo sapiens OX=9606 GN=NUP133 PE=1 SV=2	22	0,831	0,91	0,815	13967416,95	10662313,16	16367385,17
Q02878	RPL6	Large ribosomal subunit protein eL6 OS=Homo sapiens OX=9606 GN=RPL6 PE=1 SV=3	15	0,689	1,029	0,815	70378925,98	42991779,73	63253965,44
Q7L9L4	MOB1B	MOB kinase activator 1B OS=Homo sapiens OX=9606 GN=MOB1B PE=1 SV=3	2	0,532	1,547	0,815	1705625,213	803136,5675	996332,5128
Q8WYA6	CTNNBL1	Beta-catenin-like protein 1 OS=Homo sapiens OX=9606 GN=CTNNBL1 PE=1 SV=1	7	0,68	1,171	0,814	4179069,319	2306076,9	3095385,103
A0AV96	RBM47	RNA-binding protein 47 OS=Homo sapiens OX=9606 GN=RBM47 PE=1 SV=2	5	1,094	0,474	0,813	76100,3346	643563,9298	655200,1578
Q2TAY7	SMU1	WD40 repeat-containing protein SMU1 OS=Homo sapiens OX=9606 GN=SMU1 PE=1 SV=2	10	0,712	1,226	0,813	13228148,92	9350028,28	11918429,19
P08195	SLC3A2	Amino acid transporter heavy chain SLC3A2 OS=Homo sapiens OX=9606 GN=SLC3A2 PE=1 SV=3	25	4,227	0,211	0,812	53217874,16	208684587,1	286321809,4
P19320	VCAM1	Vascular cell adhesion protein 1 OS=Homo sapiens OX=9606 GN=VCAM1 PE=1 SV=1	5	1,223	0,611	0,811	2006114,608	6020588,314	3958332,825
Q15424	SAFB	Scaffold attachment factor B1 OS=Homo sapiens OX=9606 GN=SAFB PE=1 SV=4	7	0,585	1,239	0,811	20687482,81	12148970,44	13313500,58
Q5J8M3	EMC4	ER membrane protein complex subunit 4 OS=Homo sapiens OX=9606 GN=EMC4 PE=1 SV=2	3	1000	0,001	0,81		885275,8252	1639083,614
O00255	MEN1	Menin OS=Homo sapiens OX=9606 GN=MEN1 PE=1 SV=5	2	0,78	0,985	0,81	586222,9291	481212,8134	338107,9241
Q6P1J9	CDC73	Parafibromin OS=Homo sapiens OX=9606 GN=CDC73 PE=1 SV=1	8	1,027	0,782	0,809	2011940,748	2017834,924	2400230,877
Q15392	DHCR24	Delta(24)-sterol reductase OS=Homo sapiens OX=9606 GN=DHCR24 PE=1 SV=2	5	0,578	1,445	0,809	10289853,89	6047195,263	8530385,403
Q15691	MAPRE1	Microtubule-associated protein RP/EB family member 1 OS=Homo sapiens OX=9606 GN=MAPRE	13	0,481	2,007	0,809	27870101,11	11732171,97	16712296,81
Q69YN4	VIRMA	Protein virilizer homolog OS=Homo sapiens OX=9606 GN=VIRMA PE=1 SV=2	4	0,773	1,086	0,808	811568,8302	610004,1241	752923,1058
P13861	PRKAR2A	cAMP-dependent protein kinase type II-alpha regulatory subunit OS=Homo sapiens OX=9606 GN	9	0,698	1,401	0,808	5575614,572	2886707,543	4526606,173
P62280	RPS11	Small ribosomal subunit protein uS17 OS=Homo sapiens OX=9606 GN=RPS11 PE=1 SV=3	6	0,556	1,277	0,808	31363824,06	19073653,65	21982994,56
O95716	RAB3D	Ras-related protein Rab-3D OS=Homo sapiens OX=9606 GN=RAB3D PE=1 SV=1	3	1,649	0,593	0,807	3018596,775	3969854,334	5367182,476
Q6ZXV5	TMTC3	Protein O-mannosyl-transferase TMTC3 OS=Homo sapiens OX=9606 GN=TMTC3 PE=1 SV=2	4	1,354	0,82	0,807	1607364,95	1962714,779	2023823,485
P51687	SUOX	Sulfite oxidase, mitochondrial OS=Homo sapiens OX=9606 GN=SUOX PE=1 SV=2	2	1,167	0,592	0,807	195200,1528	146259,7195	327728,9511
P18077	RPL35A	Large ribosomal subunit protein eL33 OS=Homo sapiens OX=9606 GN=RPL35A PE=1 SV=2	3	0,856	0,84	0,807	11367088,72	10411371,7	12232488,45
P20700	LMNB1	Lamin-B1 OS=Homo sapiens OX=9606 GN=LMNB1 PE=1 SV=2	33	0,606	1,355	0,807	292658660,6	173412746,3	219729695,2
Q9H1E3	NUCKS1	Nuclear ubiquitous casein and cyclin-dependent kinase substrate 1 OS=Homo sapiens OX=9606	3	0,334	2,713	0,807	2873783,419	1086610,821	1113823,389
Q15393	SF3B3	Splicing factor 3B subunit 3 OS=Homo sapiens OX=9606 GN=SF3B3 PE=1 SV=4	33	0,857	1,039	0,806	56708459,71	46958671,73	63272292,67
P38159	RBMX	RNA-binding motif protein, X chromosome OS=Homo sapiens OX=9606 GN=RBMX PE=1 SV=3	9	0,743	1,105	0,806	52059753,3	36473841,01	47845963,31
P26641	EEF1G	Elongation factor 1-gamma OS=Homo sapiens OX=9606 GN=EEF1G PE=1 SV=3	19	0,56	1,63	0,806	109335836,9	59726603,73	73207346,73
P23588	EIF4B	Eukaryotic translation initiation factor 4B OS=Homo sapiens OX=9606 GN=EIF4B PE=1 SV=2	7	0,458	2,324	0,806	6831063,812	2216476,898	3042955,819
Q9UH17	APOBEC3B	DNA dC->dU-editing enzyme APOBEC-3B OS=Homo sapiens OX=9606 GN=APOBEC3B PE=1 SV=1	4	3,912	0,281	0,805	291424,2	1701798,576	1963083,709
O95139	NDUFB6	NADH dehydrogenase [ubiquinone] 1 beta subcomplex subunit 6 OS=Homo sapiens OX=9606 GN	3	1,954	0,425	0,805	1415250,52	2825721,559	4362543,077
O15321	TM9SF1	Transmembrane 9 superfamily member 1 OS=Homo sapiens OX=9606 GN=TM9SF1 PE=2 SV=2	3	1,765	0,453	0,805	519472,7139	2277553,213	1129626,324
Q14676	MDC1	Mediator of DNA damage checkpoint protein 1 OS=Homo sapiens OX=9606 GN=MDC1 PE=1 SV=	12	0,328	2,327	0,805	7159127,035	1924409,267	2688844,421
P61006	RAB8A	Ras-related protein Rab-8A OS=Homo sapiens OX=9606 GN=RAB8A PE=1 SV=1	6	1,284	0,759	0,804	36823155,83	50824943,52	47760900,71
Q9Y285	FARSA	Phenylalanine--tRNA ligase alpha subunit OS=Homo sapiens OX=9606 GN=FARSA PE=1 SV=3	11	0,676	1,305	0,804	5243312,472	3509385,834	4725861,035
A0A994J6K9	NCKAP1	Nck-associated protein 1 OS=Homo sapiens OX=9606 GN=NCKAP1 PE=1 SV=1	3	0,978	0,879	0,802	1329182,194	1154005,977	1540975,638
Q9BSH5	HDHD3	Haloacid dehalogenase-like hydrolase domain-containing protein 3 OS=Homo sapiens OX=9606	2	3,653	0,249	0,801	176151,1902	725228,5221	866319,297
P10909	CLU	Clusterin OS=Homo sapiens OX=9606 GN=CLU PE=1 SV=1	4	1,289	1,117	0,801	570283,8136	3348969,897	205533,1087
Q8WVC6	DCAKD	Dephospho-CoA kinase domain-containing protein OS=Homo sapiens OX=9606 GN=DCAKD PE=1	2	1,014	0,812	0,801	8919746,479	6318850,455	11048863,47
Q96P16	RPRD1A	Regulation of nuclear pre-mRNA domain-containing protein 1A OS=Homo sapiens OX=9606 GN=	4	0,569	1,774	0,8	1591678,629	622038,5937	1015050,746
O14744	PRMT5	Protein arginine N-methyltransferase 5 OS=Homo sapiens OX=9606 GN=PRMT5 PE=1 SV=4	7	0,409	1,946	0,8	7720954,275	1996658,147	3507639,909
Q9H5Q4	TFB2M	Dimethyladenosine transferase 2, mitochondrial OS=Homo sapiens OX=9606 GN=TFB2M PE=1 S	2	1,833	0,439	0,799	195615,9597	689152,4624	746493,7899
Q9NXV6	CDKN2AIP	CDKN2A-interacting protein OS=Homo sapiens OX=9606 GN=CDKN2AIP PE=1 SV=3	2	1,657	0,632	0,799	320926,0101	550117,6482	580774,3681
P31930	UQCRC1	Cytochrome b-c1 complex subunit 1, mitochondrial OS=Homo sapiens OX=9606 GN=UQCRC1 PE	11	1,2	0,628	0,799	22242146,6	25860466,73	36997278,79
Q15007	WTAP	Pre-mRNA-splicing regulator WTAP OS=Homo sapiens OX=9606 GN=WTAP PE=1 SV=2	5	0,665	1,124	0,799	2829421,857	1688858,196	2822667,557
Q9Y5X1	SNX9	Sorting nexin-9 OS=Homo sapiens OX=9606 GN=SNX9 PE=1 SV=1	2	1,639	0,474	0,798	448593,599	885456,826	1024216,589
Q8WVX9	FAR1	Fatty acyl-CoA reductase 1 OS=Homo sapiens OX=9606 GN=FAR1 PE=1 SV=1	9	0,265	3,137	0,798	5118248,755	954109,8603	1576369,623
Q9BTZ2	DHRS4	Dehydrogenase/reductase SDR family member 4 OS=Homo sapiens OX=9606 GN=DHRS4 PE=1 S	3	2,835	0,275	0,797	390849,8778	1089577,154	1919737,203
Q15291	RBBP5	Retinoblastoma-binding protein 5 OS=Homo sapiens OX=9606 GN=RBBP5 PE=1 SV=2	3	0,718	0,853	0,797	778431,4476	323854,8715	950126,8735
Q9NRL3	STRN4	Striatin-4 OS=Homo sapiens OX=9606 GN=STRN4 PE=1 SV=2	3	0,648	1,251	0,797	1459589,577	1231632,633	1254317,493
O14602	EIF1AY	Eukaryotic translation initiation factor 1A, Y-chromosomal OS=Homo sapiens OX=9606 GN=EIF1A	3	0,583	2,277	0,797	6077728,076	3953972,082	3708049,317
O43808	SLC25A17	Peroxisomal membrane protein PMP34 OS=Homo sapiens OX=9606 GN=SLC25A17 PE=1 SV=1	2	0,418	1,905	0,797	146292,4357	61173,67259	76785,89108
Q5JTV8	TOR1AIP1	Torsin-1A-interacting protein 1 OS=Homo sapiens OX=9606 GN=TOR1AIP1 PE=1 SV=2	14	2,49	0,332	0,796	6617793,338	16350458,36	20186274,3
Q5VYK3	ECPAS	Proteasome adapter and scaffold protein ECM29 OS=Homo sapiens OX=9606 GN=ECPAS PE=1 S	3	1,035	1	0,796	958178,6466	827280,637	653328,4472
P09661	SNRPA1	U2 small nuclear ribonucleoprotein A' OS=Homo sapiens OX=9606 GN=SNRPA1 PE=1 SV=2	12	0,913	0,842	0,796	22519187,24	20215360,6	24989197,4
Q9NQG5	RPRD1B	Regulation of nuclear pre-mRNA domain-containing protein 1B OS=Homo sapiens OX=9606 GN=	7	0,72	0,954	0,796	8890389,613	5807739,086	9441918,194
Q8TEA8	DTD1	D-aminoacyl-tRNA deacylase 1 OS=Homo sapiens OX=9606 GN=DTD1 PE=1 SV=2	2	0,461	2,076	0,796	1643833,354	659211,4001	873018,4911
P43304	GPD2	Glycerol-3-phosphate dehydrogenase, mitochondrial OS=Homo sapiens OX=9606 GN=GPD2 PE=	22	1,319	0,626	0,794	17060715,06	27604940,64	28823563,8
Q9NQT4	EXOSC5	Exosome complex component RRP46 OS=Homo sapiens OX=9606 GN=EXOSC5 PE=1 SV=1	3	1,055	0,665	0,794	1414810,402	1545545,501	2187030,091
Q13555	CAMK2G	Calcium/calmodulin-dependent protein kinase type II subunit gamma OS=Homo sapiens OX=960	2	1,044	0,715	0,794	3117243,542	3123405,182	3865392,199
Q9Y2A7	NCKAP1	Nck-associated protein 1 OS=Homo sapiens OX=9606 GN=NCKAP1 PE=1 SV=1	9	0,975	0,846	0,794	2840194,743	2662516,848	3499866,985
Q12905	ILF2	Interleukin enhancer-binding factor 2 OS=Homo sapiens OX=9606 GN=ILF2 PE=1 SV=2	15	0,911	0,87	0,794	71710104,19	64777374,96	83349195,14
Q96CU9	FOXRED1	FAD-dependent oxidoreductase domain-containing protein 1 OS=Homo sapiens OX=9606 GN=FC	2	0,599	1,067	0,794	737661,4316	145906,049	665072,2232
O00743	PPP6C	Serine/threonine-protein phosphatase 6 catalytic subunit OS=Homo sapiens OX=9606 GN=PPP6	3	0,528	1,111	0,794	1812009,382	667433,4672	1073948,666
Q99986	VRK1	Serine/threonine-protein kinase VRK1 OS=Homo sapiens OX=9606 GN=VRK1 PE=1 SV=1	7	0,418	1,381	0,794	9738812,609	6172225,893	9790846,76
P31939	ATIC	Bifunctional purine biosynthesis protein ATIC OS=Homo sapiens OX=9606 GN=ATIC PE=1 SV=3	24	0,336	2,161	0,794	36818851,59	11125637,6	16062932,78
Q9HBR0	SLC38A10	Solute carrier family 38 member 10 OS=Homo sapiens OX=9606 GN=SLC38A10 PE=1 SV=2	2	1000	0,001	0,793		378284,0258	464657,0242
Q9Y265	RUVBL1	RuvB-like 1 OS=Homo sapiens OX=9606 GN=RUVBL1 PE=1 SV=1	14	0,782	1,015	0,793	33391457,92	24482396,11	37368988,57
O15372	EIF3H	Eukaryotic translation initiation factor 3 subunit H OS=Homo sapiens OX=9606 GN=EIF3H PE=1 S	10	0,76	1,1	0,793	11818413,35	7870311,257	18516668,23
P49750	YLPM1	YLP motif-containing protein 1 OS=Homo sapiens OX=9606 GN=YLPM1 PE=1 SV=4	12	0,681	1,11	0,793	6130277,44	5158608,92	6517498,377
O43324	EEF1E1	Eukaryotic translation elongation factor 1 epsilon-1 OS=Homo sapiens OX=9606 GN=EEF1E1 PE=	5	1,418	0,656	0,792	1506301,739	2348653,24	3004335,358
Q15437	SEC23B	Protein transport protein Sec23B OS=Homo sapiens OX=9606 GN=SEC23B PE=1 SV=2	3	1,02	0,793	0,792	811380,0818	900732,3667	1073894,184
Q9UDY8	MALT1	Mucosa-associated lymphoid tissue lymphoma translocation protein 1 OS=Homo sapiens OX=96	2	0,392	2,017	0,791	16627294,81	6462905,175	8166158,193
P49768	PSEN1	Presenilin-1 OS=Homo sapiens OX=9606 GN=PSEN1 PE=1 SV=1	2	1,784	0,379	0,79	675404,0678	1141731,179	1711531,813
P23193	TCEA1	Transcription elongation factor A protein 1 OS=Homo sapiens OX=9606 GN=TCEA1 PE=1 SV=2	8	0,475	1,63	0,79	8626016,771	4129701,928	4717836,678
P62316	SNRPD2	Small nuclear ribonucleoprotein Sm D2 OS=Homo sapiens OX=9606 GN=SNRPD2 PE=1 SV=1	6	0,784	0,945	0,789	26307120,84	21337182,34	28274104,86
Q66K14	TBC1D9B	TBC1 domain family member 9B OS=Homo sapiens OX=9606 GN=TBC1D9B PE=1 SV=3	2	0,783	0,878	0,789	266736,624	208857,6507	318248,499
Q9Y5S2	CDC42BPB	Serine/threonine-protein kinase MRCK beta OS=Homo sapiens OX=9606 GN=CDC42BPB PE=1 SV	2	0,701	1,096	0,789	383781,1712	93762,26236	118844,0075
Q9H3U1	UNC45A	Protein unc-45 homolog A OS=Homo sapiens OX=9606 GN=UNC45A PE=1 SV=1	9	1,387	0,601	0,788	9020502,566	7995413,069	8613764,774
P63208	SKP1	S-phase kinase-associated protein 1 OS=Homo sapiens OX=9606 GN=SKP1 PE=1 SV=2	6	0,656	1,216	0,788	18840177,61	12261036,2	15500820,67
Q16630	CPSF6	Cleavage and polyadenylation specificity factor subunit 6 OS=Homo sapiens OX=9606 GN=CPSF6	4	0,474	1,623	0,788	4512365,939	1106803,703	1753135,892
Q9BXJ9	NAA15	N-alpha-acetyltransferase 15, NatA auxiliary subunit OS=Homo sapiens OX=9606 GN=NAA15 PE=	2	0,456	1,827	0,788	1529597,156	399178,5792	817271,0307
Q14669	TRIP12	E3 ubiquitin-protein ligase TRIP12 OS=Homo sapiens OX=9606 GN=TRIP12 PE=1 SV=1	3	0,915	0,69	0,787	577450,2554	510950,2685	745010,0764
Q7L2E3	DHX30	ATP-dependent RNA helicase DHX30 OS=Homo sapiens OX=9606 GN=DHX30 PE=1 SV=1	19	0,699	1,177	0,787	11812067,7	6774084,498	10363257,21
P42166	TMPO	Lamina-associated polypeptide 2, isoform alpha OS=Homo sapiens OX=9606 GN=TMPO PE=1 SV	10	0,609	1,182	0,787	73957268,34	48816184,97	66408493,3
Q8N1G4	LRRC47	Leucine-rich repeat-containing protein 47 OS=Homo sapiens OX=9606 GN=LRRC47 PE=1 SV=1	11	0,413	2,025	0,787	11880434,94	4011889,768	5340853,162
Q9UJU6	DBNL	Drebrin-like protein OS=Homo sapiens OX=9606 GN=DBNL PE=1 SV=1	7	1,17	0,825	0,786	4022116,437	6336184,763	6741156,975
O15381	NVL	Nuclear valosin-containing protein-like OS=Homo sapiens OX=9606 GN=NVL PE=1 SV=1	2	0,884	0,919	0,786	672717,619	532177,6622	687889,697

P62266	RPS23	Small ribosomal subunit protein uS12 OS=Homo sapiens OX=9606 GN=RPS23 PE=1 SV=3	6	0,272	2,808	0,786	17250178,06	4672433,663	5898486,773
P35580	MYH10	Myosin-10 OS=Homo sapiens OX=9606 GN=MYH10 PE=1 SV=3	39	0,225	3,489	0,786	48463588,78	10377124,59	14150194,73
Q9NVH0	EXD2	Exonuclease 3'-5' domain-containing protein 2 OS=Homo sapiens OX=9606 GN=EXD2 PE=1 SV=2	3	1,793	0,447	0,785	354526,1795	611946,8132	804337,6078
P21912	SDHB	Succinate dehydrogenase [ubiquinone] iron-sulfur subunit, mitochondrial OS=Homo sapiens OX=	8	1,724	0,556	0,785	8277360,777	13582511,47	19931065,01
Q01082	SPTBN1	Spectrin beta chain, non-erythrocytic 1 OS=Homo sapiens OX=9606 GN=SPTBN1 PE=1 SV=2	78	1,455	0,539	0,785	87710194,12	126115936,1	164167656,7
Q15029	EFTUD2	116 kDa U5 small nuclear ribonucleoprotein component OS=Homo sapiens OX=9606 GN=EFTUD2	25	0,668	1,137	0,785	109554143,3	80305589,46	118123059,3
P83731	RPL24	Large ribosomal subunit protein eL24 OS=Homo sapiens OX=9606 GN=RPL24 PE=1 SV=1	2	0,341	1,47	0,785	2828164,787	775764,3059	1621942,82
Q9UHG3	PCYOX1	Prenylcysteine oxidase 1 OS=Homo sapiens OX=9606 GN=PCYOX1 PE=1 SV=3	14	0,882	0,849	0,784	19169099,64	14143918,79	19083217,68
P62917	RPL8	Large ribosomal subunit protein uL2 OS=Homo sapiens OX=9606 GN=RPL8 PE=1 SV=2	7	0,584	1,28	0,782	54890740,64	37991180,88	48300640,26
P07384	CAPN1	Calpain-1 catalytic subunit OS=Homo sapiens OX=9606 GN=CAPN1 PE=1 SV=1	20	1,667	0,451	0,781	6807664,158	8717951,842	11417645,68
P57740	NUP107	Nuclear pore complex protein Nup107 OS=Homo sapiens OX=9606 GN=NUP107 PE=1 SV=1	17	0,83	0,847	0,781	10539370,26	8945137,79	13107999,26
Q9BQE5	APOL2	Apolipoprotein L2 OS=Homo sapiens OX=9606 GN=APOL2 PE=1 SV=2	8	10,274	0,116	0,78	1624736,246	16778629,23	16479217,75
Q04637	EIF4G1	Eukaryotic translation initiation factor 4 gamma 1 OS=Homo sapiens OX=9606 GN=EIF4G1 PE=1 S	32	0,771	1,044	0,78	46760166,94	31667926,97	60283135,53
P28074	PSMB5	Proteasome subunit beta type-5 OS=Homo sapiens OX=9606 GN=PSMB5 PE=1 SV=3	8	0,263	2,741	0,78	23582212,66	4666065,935	8508569,155
P09110	ACAA1	3-ketoacyl-CoA thiolase, peroxisomal OS=Homo sapiens OX=9606 GN=ACAA1 PE=1 SV=2	11	2,44	0,336	0,779	1856318,531	4670953,205	5397613,764
A4D1E9	GTPBP10	GTP-binding protein 10 OS=Homo sapiens OX=9606 GN=GTPBP10 PE=1 SV=1	4	1,379	0,561	0,779	606625,4689	754780,9398	1046721,222
P82673	MRPS35	Small ribosomal subunit protein mS35 OS=Homo sapiens OX=9606 GN=MRPS35 PE=1 SV=1	5	1,061	0,737	0,779	1880154,597	1967878,504	2722064,168
P33240	CSTF2	Cleavage stimulation factor subunit 2 OS=Homo sapiens OX=9606 GN=CSTF2 PE=1 SV=1	4	1,001	0,776	0,779	9105184,619	13696323,93	12918909,1
P46100	ATRX	Transcriptional regulator ATRX OS=Homo sapiens OX=9606 GN=ATRX PE=1 SV=6	3	0,201	3,861	0,779	889714,5823	145152,6348	183180,7457
O75964	ATP5MG	ATP synthase subunit g, mitochondrial OS=Homo sapiens OX=9606 GN=ATP5MG PE=1 SV=3	4	1,065	0,795	0,778	7791273,391	10223663,45	12225158,58
O75844	ZMPSTE24	CAAX prenyl protease 1 homolog OS=Homo sapiens OX=9606 GN=ZMPSTE24 PE=1 SV=2	8	1,107	0,632	0,777	6392589,515	8043612,985	10818785,39
Q9UHY7	ENOPH1	Enolase-phosphatase E1 OS=Homo sapiens OX=9606 GN=ENOPH1 PE=1 SV=1	2	0,316	1,908	0,777	988177,7929	302970,9986	325288,5849
Q1KMD3	HNRNPUL2	Heterogeneous nuclear ribonucleoprotein U-like protein 2 OS=Homo sapiens OX=9606 GN=HNRI	15	1,103	0,716	0,776	20556362,92	20623934,84	27667374,73
O14925	TIMM23	Mitochondrial import inner membrane translocase subunit Tim23 OS=Homo sapiens OX=9606 G	2	1,085	0,685	0,776	1099594,085	1762934,841	1644816,386
Q9BZK7	TBL1XR1	F-box-like/WD repeat-containing protein TBL1XR1 OS=Homo sapiens OX=9606 GN=TBL1XR1 PE=	7	0,794	1,146	0,776	6541644,332	5717474,922	4697820,412
Q99459	CDC5L	Cell division cycle 5-like protein OS=Homo sapiens OX=9606 GN=CDC5L PE=1 SV=2	16	0,59	1,253	0,776	18268966,13	10734180,61	13481493,61
O75534	CSDE1	Cold shock domain-containing protein E1 OS=Homo sapiens OX=9606 GN=CSDE1 PE=1 SV=2	15	0,38	2,024	0,776	14896450,26	4742329,308	6545426,632
O43809	NUDT21	Cleavage and polyadenylation specificity factor subunit 5 OS=Homo sapiens OX=9606 GN=NUDT2	9	0,649	1,168	0,775	19974345,12	12028449,89	17478989,03
O75746	SLC25A12	Electrogenic aspartate/glutamate antiporter SLC25A12, mitochondrial OS=Homo sapiens OX=960	13	2,032	0,422	0,774	1875762,383	3997115,005	4978185,599
A0A1W2PNX8	UNC45A	Unc-45 myosin chaperone A OS=Homo sapiens OX=9606 GN=UNC45A PE=1 SV=1	4	1,24	0,541	0,774	1516188,788	3181304,662	3062103,57
Q9NTI5	PDS5B	Sister chromatid cohesion protein PDS5 homolog B OS=Homo sapiens OX=9606 GN=PDS5B PE=1	10	0,764	1,006	0,774	4541354,637	3072373,326	4881806,542
Q96K17	BTF3L4	Transcription factor BTF3 homolog 4 OS=Homo sapiens OX=9606 GN=BTF3L4 PE=1 SV=1	5	0,447	2,369	0,774	3977621,086	1629150,393	2273440,227
P27695	APEX1	DNA-(apurinic or apyrimidinic site) endonuclease OS=Homo sapiens OX=9606 GN=APEX1 PE=1 S	11	0,379	2,682	0,774	32442364,94	11051832,62	11979816,48
Q5JTJ3	COA6	Cytochrome c oxidase assembly factor 6 homolog OS=Homo sapiens OX=9606 GN=COA6 PE=1 SV	2	2,571	0,274	0,773	732090,2712	1620303,398	1902883,714
Q12849	GRSF1	G-rich sequence factor 1 OS=Homo sapiens OX=9606 GN=GRSF1 PE=1 SV=3	6	0,903	0,557	0,773	1458416,568	884714,0588	2666050,699
P30876	POLR2B	DNA-directed RNA polymerase II subunit RPB2 OS=Homo sapiens OX=9606 GN=POLR2B PE=1 SV	8	0,773	0,865	0,773	7766483,53	8553575,914	9509811,045
Q15459	SF3A1	Splicing factor 3A subunit 1 OS=Homo sapiens OX=9606 GN=SF3A1 PE=1 SV=1	22	0,738	1,151	0,773	33073825,33	22916033,1	33936673,07
Q8TB22	SPATA20	Spermatogenesis-associated protein 20 OS=Homo sapiens OX=9606 GN=SPATA20 PE=2 SV=3	3	0,645	1,199	0,773	207338,6465	133646,6918	172871,5594
Q15365	PCBP1	Poly(rC)-binding protein 1 OS=Homo sapiens OX=9606 GN=PCBP1 PE=1 SV=2	7	0,486	1,479	0,773	41579554,93	15571193,9	31991873,59
Q13813	SPTAN1	Spectrin alpha chain, non-erythrocytic 1 OS=Homo sapiens OX=9606 GN=SPTAN1 PE=1 SV=3	108	1,599	0,48	0,772	146564203,3	225413794,6	295407859
O95197	RTN3	Reticulon-3 OS=Homo sapiens OX=9606 GN=RTN3 PE=1 SV=2	2	1,092	0,654	0,772	14026479,04	15998063,08	18105128,52
P22059	OSBP	Oxysterol-binding protein 1 OS=Homo sapiens OX=9606 GN=OSBP PE=1 SV=1	4	0,828	0,915	0,772	852150,9546	705537,0074	1161468,349
Q9Y3D6	FIS1	Mitochondrial fission 1 protein OS=Homo sapiens OX=9606 GN=FIS1 PE=1 SV=2	2	1,477	0,509	0,771	2114504,863	3016419,6	4064888,017
Q9H936	SLC25A22	Mitochondrial glutamate carrier 1 OS=Homo sapiens OX=9606 GN=SLC25A22 PE=1 SV=1	3	1,275	0,625	0,771	1333481,795	1193522,98	2128383,364
Q9UPQ0	LIMCH1	LIM and calponin homology domains-containing protein 1 OS=Homo sapiens OX=9606 GN=LIMC	12	0,89	1,518	0,771	1334400,365	1254452,62	601319,1102
Q9UDW1	UQCR10	Cytochrome b-c1 complex subunit 9 OS=Homo sapiens OX=9606 GN=UQCR10 PE=1 SV=3	2	0,865	0,828	0,771	2084190,747	1856971,035	2204706,855
O00115	DNASE2	Deoxyribonuclease-2-alpha OS=Homo sapiens OX=9606 GN=DNASE2 PE=1 SV=2	2	4,294	0,178	0,77	606395,2045	2625037,962	3409621,434
Q9NYL9	TMOD3	Tropomodulin-3 OS=Homo sapiens OX=9606 GN=TMOD3 PE=1 SV=1	13	1,997	0,469	0,77	7138266,135	14899566,55	19317757,42
Q9NRG7	SDR39U1	Epimerase family protein SDR39U1 OS=Homo sapiens OX=9606 GN=SDR39U1 PE=1 SV=3	4	1,523	0,334	0,77	149435,8932	288952,3541	313567,1001
Q9UBQ7	GRHPR	Glyoxylate reductase/hydroxypyruvate reductase OS=Homo sapiens OX=9606 GN=GRHPR PE=1 S	6	1,34	0,402	0,77	1246940,736	784116,3936	3505978,054
P78346	RPP30	Ribonuclease P protein subunit p30 OS=Homo sapiens OX=9606 GN=RPP30 PE=1 SV=1	4	1,137	0,999	0,77	628868,5119	424389,3338	820547,6816
Q14683	SMC1A	Structural maintenance of chromosomes protein 1A OS=Homo sapiens OX=9606 GN=SMC1A PE=	30	0,724	1,032	0,769	27827144,62	19747117,15	25816836,18
P00749	PLAU	Urokinase-type plasminogen activator OS=Homo sapiens OX=9606 GN=PLAU PE=1 SV=3	2	1000	0,001	0,768		302086,2313	324870,5813
P40939	HADHA	Trifunctional enzyme subunit alpha, mitochondrial OS=Homo sapiens OX=9606 GN=HADHA PE=1	27	1,296	0,62	0,768	50450943,88	66545873,57	87879124,14
Q9Y673	ALG5	Dolichyl-phosphate beta-glucosyltransferase OS=Homo sapiens OX=9606 GN=ALG5 PE=1 SV=1	6	1,108	0,655	0,768	1848867,043	2042349,425	3448568,922
P54136	RARS1	Arginine--tRNA ligase, cytoplasmic OS=Homo sapiens OX=9606 GN=RARS1 PE=1 SV=2	24	0,751	0,961	0,768	31977681,56	23790805,38	31023465,75
Q9BTX1	NDC1	Nucleoporin NDC1 OS=Homo sapiens OX=9606 GN=NDC1 PE=1 SV=2	6	0,483	1,038	0,767	2230882,271	1060628,746	2073260,002
Q15185	PTGES3	Prostaglandin E synthase 3 OS=Homo sapiens OX=9606 GN=PTGES3 PE=1 SV=1	3	0,42	1,811	0,767	3905813,619	1274460,11	2145758,496
P49590	HARS2	Histidine--tRNA ligase, mitochondrial OS=Homo sapiens OX=9606 GN=HARS2 PE=1 SV=1	3	1,616	0,504	0,766	1215566,193	1987207,929	2270960,325
P50579	METAP2	Methionine aminopeptidase 2 OS=Homo sapiens OX=9606 GN=METAP2 PE=1 SV=1	6	0,34	2,295	0,766	3002090,873	758594,8064	1191401,603
Q8NCN5	PDPR	Pyruvate dehydrogenase phosphatase regulatory subunit, mitochondrial OS=Homo sapiens OX=9	9	1,148	0,71	0,765	4015000,168	3954258,766	5513079,396
Q9UQE7	SMC3	Structural maintenance of chromosomes protein 3 OS=Homo sapiens OX=9606 GN=SMC3 PE=1 S	30	0,728	1,075	0,765	32282810,37	22506814,38	31805205,4
P42167	TMPO	Lamina-associated polypeptide 2, isoforms beta/gamma OS=Homo sapiens OX=9606 GN=TMPO	9	0,644	1,048	0,765	37503534,1	22715372,11	29119986,57
P12004	PCNA	Proliferating cell nuclear antigen OS=Homo sapiens OX=9606 GN=PCNA PE=1 SV=1	10	0,764	1,327	0,764	51177827,14	31293083,36	46251676,68
Q99805	TM9SF2	Transmembrane 9 superfamily member 2 OS=Homo sapiens OX=9606 GN=TM9SF2 PE=1 SV=1	5	1,982	0,686	0,763	4290002,25	3578759,988	8103682,567
Q86SF2	GALNT7	N-acetylgalactosaminyltransferase 7 OS=Homo sapiens OX=9606 GN=GALNT7 PE=1 SV=1	5	1,654	0,593	0,763	2273911,249	3464802,701	4146116,312
P39023	RPL3	Large ribosomal subunit protein uL3 OS=Homo sapiens OX=9606 GN=RPL3 PE=1 SV=2	14	0,634	1,33	0,763	42852450,16	23079867,62	30454451,04
Q9HA64	FN3KRP	Ketosamine-3-kinase OS=Homo sapiens OX=9606 GN=FN3KRP PE=1 SV=2	3	0,614	1,401	0,763	662673,4065	241033,3525	483324,6382
Q9UHX1	PUF60	Poly(U)-binding-splicing factor PUF60 OS=Homo sapiens OX=9606 GN=PUF60 PE=1 SV=1	19	0,559	1,31	0,763	30775807,79	16058459	24394449,34
Q15070	OXA1L	Mitochondrial inner membrane protein OXA1L OS=Homo sapiens OX=9606 GN=OXA1L PE=1 SV=	4	1,296	0,651	0,762	1687739,775	2271033,933	3426734,574
P83916	CBX1	Chromobox protein homolog 1 OS=Homo sapiens OX=9606 GN=CBX1 PE=1 SV=1	6	0,519	1,506	0,762	19049073,5	9163142,068	9386887,472
O15020	SPTBN2	Spectrin beta chain, non-erythrocytic 2 OS=Homo sapiens OX=9606 GN=SPTBN2 PE=1 SV=3	3	0,119	3,065	0,762	1337462,532	24430,46612	279007,1788
Q9UL03	INTS6	Integrator complex subunit 6 OS=Homo sapiens OX=9606 GN=INTS6 PE=1 SV=1	2	2,363	0,327	0,761	158344,2312	354730,1824	473662,7188
Q9BWS9	CHID1	Chitinase domain-containing protein 1 OS=Homo sapiens OX=9606 GN=CHID1 PE=1 SV=1	3	1,138	0,649	0,761	1578227,242	1774801,724	2382221,873
Q15637	SF1	Splicing factor 1 OS=Homo sapiens OX=9606 GN=SF1 PE=1 SV=4	10	0,752	0,892	0,761	13847515,48	10356408,75	14798546,44
P31948	STIP1	Stress-induced-phosphoprotein 1 OS=Homo sapiens OX=9606 GN=STIP1 PE=1 SV=1	23	0,443	1,735	0,761	67864953,73	26410862,62	38252043,94
Q14696	MESD	LRP chaperone MESD OS=Homo sapiens OX=9606 GN=MESD PE=1 SV=2	5	1,236	0,626	0,76	5508279,823	6612898,903	8434611,597
Q9NNW7	TXNRD2	Thioredoxin reductase 2, mitochondrial OS=Homo sapiens OX=9606 GN=TXNRD2 PE=1 SV=3	2	0,668	1,124	0,76	5891881,293	3990266,643	5239611,436
P62714	PPP2CB	Serine/threonine-protein phosphatase 2A catalytic subunit beta isoform OS=Homo sapiens OX=9	5	0,57	1,418	0,76	9827582,481	6062510,296	6982740,463
Q9UQ16	DNM3	Dynamin-3 OS=Homo sapiens OX=9606 GN=DNM3 PE=1 SV=4	2	0,303	2,553	0,76	256811,5444	77733,96068	98989,51936
Q86V48	LUZP1	Leucine zipper protein 1 OS=Homo sapiens OX=9606 GN=LUZP1 PE=1 SV=2	12	1,295	0,59	0,759	3964245,016	6369112,493	8178800,863
P10586	PTPRF	Receptor-type tyrosine-protein phosphatase F OS=Homo sapiens OX=9606 GN=PTPRF PE=1 SV=2	12	1,114	0,627	0,759	2138983,305	1726544,58	3458758,099
Q13362	PPP2R5C	Serine/threonine-protein phosphatase 2A 56 kDa regulatory subunit gamma isoform OS=Homo s	3	0,37	1,571	0,759	582708,3155	103545,7264	376749,3371
Q9BWD1	ACAT2	Acetyl-CoA acetyltransferase, cytosolic OS=Homo sapiens OX=9606 GN=ACAT2 PE=1 SV=2	9	0,151	5,348	0,759	35037195,12	5285996,928	7482183,983
Q9NSE4	IARS2	Isoleucine--tRNA ligase, mitochondrial OS=Homo sapiens OX=9606 GN=IARS2 PE=1 SV=2	23	1,68	0,487	0,758	16575055,7	21138794,58	29312162,39
P49207	RPL34	Large ribosomal subunit protein eL34 OS=Homo sapiens OX=9606 GN=RPL34 PE=1 SV=3	2	0,66	1,09	0,758	14811523,82	11236430,55	13615843,69
Q9UGP8	SEC63	Translocation protein SEC63 homolog OS=Homo sapiens OX=9606 GN=SEC63 PE=1 SV=2	6	0,604	1,323	0,758	2878856,454	976563,873	1856299,146
Q9Y2R9	MRPS7	Small ribosomal subunit protein uS7m OS=Homo sapiens OX=9606 GN=MRPS7 PE=1 SV=2	7	1,253	0,588	0,756	3407554,176	3830586,276	6805884,445
Q16836	HADH	Hydroxyacyl-coenzyme A dehydrogenase, mitochondrial OS=Homo sapiens OX=9606 GN=HADH I	11	0,861	1,088	0,756	11610506,98	10596839,62	17443414,79
P07900	HSP90AA1	Heat shock protein HSP 90-alpha OS=Homo sapiens OX=9606 GN=HSP90AA1 PE=1 SV=5	20	0,356	1,88	0,756	188510944,7	86536903,39	101647146,1
P52434	POLR2H	DNA-directed RNA polymerases I, II, and III subunit RPABC3 OS=Homo sapiens OX=9606 GN=POL	4	0,792	1,021	0,755	1917785,569	1698471,32	2668796,8
Q12972	PPP1R8	Nuclear inhibitor of protein phosphatase 1 OS=Homo sapiens OX=9606 GN=PPP1R8 PE=1 SV=2	5	0,658	1,305	0,755	2324274,058	1621697,389	3430281,053
Q9BWF3	RBM4	RNA-binding protein 4 OS=Homo sapiens OX=9606 GN=RBM4 PE=1 SV=1	9	0,466	1,684	0,755	15079021,8	6814203,82	9516321,625
Q15785	TOMM34	Mitochondrial import receptor subunit TOM34 OS=Homo sapiens OX=9606 GN=TOMM34 PE=1 S	8	0,281	2,399	0,755	10957531,17	2545911,957	4176703,004
O00273	DFFA	DNA fragmentation factor subunit alpha OS=Homo sapiens OX=9606 GN=DFFA PE=1 SV=1	5	0,173	2,91	0,755	4557388,305	1236691,356	1826517,465
P19388	POLR2E	DNA-directed RNA polymerases I, II, and III subunit RPABC1 OS=Homo sapiens OX=9606 GN=POL	4	0,807	0,88	0,754	2283824,101	2391943,272	2652789,211
Q9NZT2	OGFR	Opioid growth factor receptor OS=Homo sapiens OX=9606 GN=OGFR PE=1 SV=3	2	1,382	0,543	0,753	596572,7989	935875,5833	1127714,432
P30049	ATP5F1D	ATP synthase subunit delta, mitochondrial OS=Homo sapiens OX=9606 GN=ATP5F1D PE=1 SV=2	2	1,532	0,483	0,752	11755742,38	20257677,07	23747382,37
Q16850	CYP51A1	Lanosterol 14-alpha demethylase OS=Homo sapiens OX=9606 GN=CYP51A1 PE=1 SV=4	15	0,203	3,368	0,752	19458055,65	5990861,14	7293002,169
P00533	EGFR	Epidermal growth factor receptor OS=Homo sapiens OX=9606 GN=EGFR PE=1 SV=2	15	2,78	0,276	0,751	7294058,759	15765373,59	29701375,22
Q96D46	NMD3	60S ribosomal export protein NMD3 OS=Homo sapiens OX=9606 GN=NMD3 PE=1 SV=1	2	0,828	0,906	0,75	258367,7018	213953,778	285260,6633
P08865	RPSA	Small ribosomal subunit protein uS2 OS=Homo sapiens OX=9606 GN=RPSA PE=1 SV=4	14	0,59	1,105	0,75	93577196,32	51344688,44	84243966,41
Q9Y672	ALG6	Dolichyl pyrophosphate Man9GlcNAc2 alpha-1,3-glucosyltransferase OS=Homo sapiens OX=9606	2	0,757	0,957	0,749	836791,8486	627131,4832	267372,449
Q9Y520	PRRC2C	Protein PRRC2C OS=Homo sapiens OX=9606 GN=PRRC2C PE=1 SV=4	11	0,684	1,158	0,749	4785260,624	3430629,681	5631605,498
P42566	EPS15	Epidermal growth factor receptor substrate 15 OS=Homo sapiens OX=9606 GN=EPS15 PE=1 SV=2	6	1,308	0,538	0,748	1042498,353	1607099,078	2346905,596
O75947	ATP5PD	ATP synthase subunit d, mitochondrial OS=Homo sapiens OX=9606 GN=ATP5PD PE=1 SV=3	12	1,273	0,694	0,748	19375772,08	24215607,35	28892839,39
Q9BR76	CORO1B	Coronin-1B OS=Homo sapiens OX=9606 GN=CORO1B PE=1 SV=1	5	1,054	0,915	0,748	2387818,577	2796611,139	3517296,929
P05109	S100A8	Protein S100-A8 OS=Homo sapiens OX=9606 GN=S100A8 PE=1 SV=1	2	5,162	0,133	0,747	99145,40646	475666,6788	611988,1358
Q12846	STX4	Syntaxin-4 OS=Homo sapiens OX=9606 GN=STX4 PE=1 SV=2	5	3,727	0,247	0,747	1664606,699	3776076,869	4796849,951
Q13868	EXOSC2	Exosome complex component RRP4 OS=Homo sapiens OX=9606 GN=EXOSC2 PE=1 SV=2	4	1,009	0,696	0,747	2386504,107	2410758,515	3616001,435
P61964	WDR5	WD repeat-containing protein 5 OS=Homo sapiens OX=9606 GN=WDR5 PE=1 SV=1	6	0,923	0,768	0,747	3715713,793	2853894,88	4609137,704

Q5RKV6	EXOSC6	Exosome complex component MTR3 OS=Homo sapiens OX=9606 GN=EXOSC6 PE=1 SV=1	5	0,865	1,079	0,747	4150319,982	3534689,233	4053480,266
Q9NRG9	AAAS	Aladin OS=Homo sapiens OX=9606 GN=AAAS PE=1 SV=1	7	0,693	1,172	0,747	5857411,761	3669757,813	5178478,806
Q12789	GTF3C1	General transcription factor 3C polypeptide 1 OS=Homo sapiens OX=9606 GN=GTF3C1 PE=1 SV=	2	0,509	1,391	0,747	346723,4911	145373,6644	249301,4524
Q9NYK5	MRPL39	Large ribosomal subunit protein mL39 OS=Homo sapiens OX=9606 GN=MRPL39 PE=1 SV=3	8	1,076	0,579	0,746	2682619,539	4679513,522	6070939,743
O00505	KPNA3	Importin subunit alpha-4 OS=Homo sapiens OX=9606 GN=KPNA3 PE=1 SV=2	6	0,581	1,46	0,746	7241585,258	5367471,926	7382966,896
Q06203	PPAT	Amidophosphoribosyltransferase OS=Homo sapiens OX=9606 GN=PPAT PE=1 SV=1	4	0,219	2,434	0,746	2711171,006	779473,9433	497865,6534
P03886	MT-ND1	NADH-ubiquinone oxidoreductase chain 1 OS=Homo sapiens OX=9606 GN=MT-ND1 PE=1 SV=1	3	1,071	0,791	0,745	666467,8209	640524,9507	783262,3749
Q9NRL2	BAZ1A	Bromodomain adjacent to zinc finger domain protein 1A OS=Homo sapiens OX=9606 GN=BAZ1A	6	0,543	1,295	0,745	2228412,282	882159,4185	1413826,384
P31949	S100A11	Protein S100-A11 OS=Homo sapiens OX=9606 GN=S100A11 PE=1 SV=2	3	2,35	0,399	0,744	10237756,22	25809850,08	26356401,41
P49406	MRPL19	Large ribosomal subunit protein bL19m OS=Homo sapiens OX=9606 GN=MRPL19 PE=1 SV=2	6	1,171	0,642	0,744	2076520,452	2352203,168	3923388,775
Q9HCU5	PREB	Prolactin regulatory element-binding protein OS=Homo sapiens OX=9606 GN=PREB PE=1 SV=2	6	1,084	0,928	0,744	2334393,841	2752244,956	3923331,054
O94826	TOMM70	Mitochondrial import receptor subunit TOM70 OS=Homo sapiens OX=9606 GN=TOMM70 PE=1 S	11	1,148	0,63	0,743	11019768,2	11324283,12	18702243,22
O43776	NARS1	Asparagine--tRNA ligase, cytoplasmic OS=Homo sapiens OX=9606 GN=NARS1 PE=1 SV=1	9	0,729	1,062	0,743	7845773,425	4987002,54	8760279,659
Q6P179	ERAP2	Endoplasmic reticulum aminopeptidase 2 OS=Homo sapiens OX=9606 GN=ERAP2 PE=1 SV=2	10			0,742	253008,2302	348944,3067	451698,5562
Q14789	GOLGB1	Golgin subfamily B member 1 OS=Homo sapiens OX=9606 GN=GOLGB1 PE=1 SV=2	30	1,653	0,471	0,742	4376238,865	7926096,272	12638987,03
Q9Y2P8	RCL1	RNA 3'-terminal phosphate cyclase-like protein OS=Homo sapiens OX=9606 GN=RCL1 PE=1 SV=3	2	1,135	0,666	0,741	335228,472	381289,2001	560993,2234
O95336	PGLS	6-phosphogluconolactonase OS=Homo sapiens OX=9606 GN=PGLS PE=1 SV=2	6	1,064	0,705	0,741	4130310,833	4380873,342	6254910,738
P85037	FOXK1	Forkhead box protein K1 OS=Homo sapiens OX=9606 GN=FOXK1 PE=1 SV=1	2	0,956	0,822	0,741	834022,2011	862463,4235	1067973,136
Q14320	FAM50A	Protein FAM50A OS=Homo sapiens OX=9606 GN=FAM50A PE=1 SV=2	5	0,627	1,115	0,741	6273468,543	3270402,756	4416883,407
O94903	PLPBP	Pyridoxal phosphate homeostasis protein OS=Homo sapiens OX=9606 GN=PLPBP PE=1 SV=1	6	0,498	1,376	0,74	18217909,74	9274433,417	24931505,31
Q9UKG1	APPL1	DCC-interacting protein 13-alpha OS=Homo sapiens OX=9606 GN=APPL1 PE=1 SV=1	3	0,145	2,263	0,74	427871,903	39701,57777	388827,8463
P56545	CTBP2	C-terminal-binding protein 2 OS=Homo sapiens OX=9606 GN=CTBP2 PE=1 SV=1	5	0,546	1,426	0,739	7018887,573	4234618,8	5340394,876
P50851	LRBA	Lipopolysaccharide-responsive and beige-like anchor protein OS=Homo sapiens OX=9606 GN=LR	6	0,885	1,057	0,738	1049560,726	358543,2142	1072856,438
Q16540	MRPL23	Large ribosomal subunit protein uL23m OS=Homo sapiens OX=9606 GN=MRPL23 PE=1 SV=1	2	1,532	0,449	0,737	1014401,152	1341641,468	2231909,248
P62993	GRB2	Growth factor receptor-bound protein 2 OS=Homo sapiens OX=9606 GN=GRB2 PE=1 SV=1	3	0,988	0,884	0,736	2336451,238	2309307,351	2824346,718
O94776	MTA2	Metastasis-associated protein MTA2 OS=Homo sapiens OX=9606 GN=MTA2 PE=1 SV=1	10	0,98	0,771	0,736	11882607,58	9289869,607	15332430,96
Q14139	UBE4A	Ubiquitin conjugation factor E4 A OS=Homo sapiens OX=9606 GN=UBE4A PE=1 SV=2	2	1,175	0,626	0,735	294524,1778	346004,6756	470519,5805
Q96TA2	YME1L1	ATP-dependent zinc metalloprotease YME1L1 OS=Homo sapiens OX=9606 GN=YME1L1 PE=1 SV=	11	0,921	0,757	0,735	3546653,252	3413102,11	4694136,811
P35269	GTF2F1	General transcription factor IIF subunit 1 OS=Homo sapiens OX=9606 GN=GTF2F1 PE=1 SV=2	3	0,634	1,034	0,735	769284,2241	354501,5201	741499,9142
P12270	TPR	Nucleoprotein TPR OS=Homo sapiens OX=9606 GN=TPR PE=1 SV=3	67	1,154	0,628	0,734	53217876,78	59437907,87	80045969,16
Q00059	TFAM	Transcription factor A, mitochondrial OS=Homo sapiens OX=9606 GN=TFAM PE=1 SV=1	10	1,127	0,793	0,734	12791704,61	10125353,33	15378954,69
Q8N1F7	NUP93	Nuclear pore complex protein Nup93 OS=Homo sapiens OX=9606 GN=NUP93 PE=1 SV=2	20	0,729	0,982	0,734	28579262,9	22482453,32	31967835,89
Q9P035	HACD3	Very-long-chain (3R)-3-hydroxyacyl-CoA dehydratase 3 OS=Homo sapiens OX=9606 GN=HACD3 F	7	0,656	1,24	0,734	21941813,36	9724794,013	21616057,97
Q6P2Q9	PRPF8	Pre-mRNA-processing-splicing factor 8 OS=Homo sapiens OX=9606 GN=PRPF8 PE=1 SV=2	56	0,635	1,147	0,734	53575974,92	30977646,06	43276724,03
Q9BUB7	TMEM70	Transmembrane protein 70, mitochondrial OS=Homo sapiens OX=9606 GN=TMEM70 PE=1 SV=2	2	1,588	0,448	0,733	1627725,024	2596716,651	3302812,105
P18433	PTPRA	Receptor-type tyrosine-protein phosphatase alpha OS=Homo sapiens OX=9606 GN=PTPRA PE=1	3	1,57	0,453	0,733	750376,8409	1175673,714	1621444,657
Q9BUN8	DERL1	Derlin-1 OS=Homo sapiens OX=9606 GN=DERL1 PE=1 SV=1	2	1,158	0,613	0,733	1608068,253	2141428,563	3001232,968
P60228	EIF3E	Eukaryotic translation initiation factor 3 subunit E OS=Homo sapiens OX=9606 GN=EIF3E PE=1 S\	17	0,661	1	0,733	19109938,72	13387132,01	17620484,32
P62854	RPS26	Small ribosomal subunit protein eS26 OS=Homo sapiens OX=9606 GN=RPS26 PE=1 SV=3	3	0,476	1,467	0,733	12969643,68	6590929,978	9760237,163
Q96DV4	MRPL38	Large ribosomal subunit protein mL38 OS=Homo sapiens OX=9606 GN=MRPL38 PE=1 SV=2	4	1,21	0,618	0,732	2050298,713	2260037,71	3765464,225
Q9NY12	GAR1	H/ACA ribonucleoprotein complex subunit 1 OS=Homo sapiens OX=9606 GN=GAR1 PE=1 SV=1	2	0,933	0,784	0,732	1470644,657	1372525,77	1875473,74
P42285	MTREX	Exosome RNA helicase MTR4 OS=Homo sapiens OX=9606 GN=MTREX PE=1 SV=3	15	0,775	0,919	0,732	9091905,585	7005305,537	10788630,11
O43719	HTATSF1	17S U2 SnRNP complex component HTATSF1 OS=Homo sapiens OX=9606 GN=HTATSF1 PE=1 SV=	9	0,453	1,877	0,732	6114605,559	2441156,198	4009974,35
P46199	MTIF2	Translation initiation factor IF-2, mitochondrial OS=Homo sapiens OX=9606 GN=MTIF2 PE=1 SV=	2	1,432	0,512	0,731	188785,8465	246630,8958	337162,7877
Q9UFW8	CGGBP1	CGG triplet repeat-binding protein 1 OS=Homo sapiens OX=9606 GN=CGGBP1 PE=1 SV=2	3	0,457	1,494	0,731	1833057,129	749324,9255	1156740,186
Q96HH9	GRAMD2B	GRAM domain-containing protein 2B OS=Homo sapiens OX=9606 GN=GRAMD2B PE=1 SV=1	3	4,235		0,73	28083,16997	333970,8676	317490,704
Q92552	MRPS27	Small ribosomal subunit protein mS27 OS=Homo sapiens OX=9606 GN=MRPS27 PE=1 SV=3	8	1,301	0,519	0,73	3294261,968	4160004,043	8040297,6
P52298	NCBP2	Nuclear cap-binding protein subunit 2 OS=Homo sapiens OX=9606 GN=NCBP2 PE=1 SV=1	3	1,176	0,753	0,73	1520329,752	1571831,253	2679053,156
Q9BYD3	MRPL4	Large ribosomal subunit protein uL4m OS=Homo sapiens OX=9606 GN=MRPL4 PE=1 SV=1	5	0,733	0,679	0,73	1529087,536	1545001,049	2460059,68
P62258	YWHAE	14-3-3 protein epsilon OS=Homo sapiens OX=9606 GN=YWHAE PE=1 SV=1	15	0,349	1,984	0,73	326847921,5	155203438,8	159681361,3
Q9Y3X0	CCDC9	Coiled-coil domain-containing protein 9 OS=Homo sapiens OX=9606 GN=CCDC9 PE=1 SV=1	2	1,748	0,484	0,728	441440,3555	716317,6447	735890,3965
Q15024	EXOSC7	Exosome complex component RRP42 OS=Homo sapiens OX=9606 GN=EXOSC7 PE=1 SV=3	4	0,906	0,763	0,728	2268486,403	2022714,907	2804687,304
Q92522	H1-10	Histone H1.10 OS=Homo sapiens OX=9606 GN=H1-10 PE=1 SV=1	6	0,629	1,103	0,728	11708485,14	7911461,064	10596289,83
Q9BRT9	GINS4	DNA replication complex GINS protein SLD5 OS=Homo sapiens OX=9606 GN=GINS4 PE=1 SV=1	2	0,472	1,501	0,728	725535,1872	297666,4511	408770,022
P61966	AP1S1	AP-1 complex subunit sigma-1A OS=Homo sapiens OX=9606 GN=AP1S1 PE=1 SV=1	2	1,911	0,383	0,727	817366,8439	1575684,82	3256910,314
Q9NPJ6	MED4	Mediator of RNA polymerase II transcription subunit 4 OS=Homo sapiens OX=9606 GN=MED4 PE	2	0,768	0,913	0,727	467945,3897	321094,2446	506706,0393
Q9Y262	EIF3L	Eukaryotic translation initiation factor 3 subunit L OS=Homo sapiens OX=9606 GN=EIF3L PE=1 S\	16	0,628	0,991	0,727	17819225,93	10984048,36	16652470,57
Q9Y3Y2	CHTOP	Chromatin target of PRMT1 protein OS=Homo sapiens OX=9606 GN=CHTOP PE=1 SV=2	6	0,491	0,935	0,727	7099011,139	3449601,162	5895216,012
Q9BYD6	MRPL1	Large ribosomal subunit protein uL1m OS=Homo sapiens OX=9606 GN=MRPL1 PE=1 SV=2	6	1,501	0,442	0,726	739951,0063	689171,7423	7345987,89
Q9P2E9	RRBP1	Ribosome-binding protein 1 OS=Homo sapiens OX=9606 GN=RRBP1 PE=1 SV=5	36	1,372	0,573	0,726	54431802,77	75581630,94	85591975,1
Q86WX3	RPS19BP1	Active regulator of SIRT1 OS=Homo sapiens OX=9606 GN=RPS19BP1 PE=1 SV=1	2	0,867	0,636	0,726	569416,1759	180727,2486	940122,2688
P67775	PPP2CA	Serine/threonine-protein phosphatase 2A catalytic subunit alpha isoform OS=Homo sapiens OX=	5	0,554	1,397	0,726	9503964,171	5695865,964	6768824,075
Q8IY21	DDX60	Probable ATP-dependent RNA helicase DDX60 OS=Homo sapiens OX=9606 GN=DDX60 PE=1 SV=	7	2,628	0,239	0,725	1370607,976	3850172,792	4262964,616
O15145	ARPC3	Actin-related protein 2/3 complex subunit 3 OS=Homo sapiens OX=9606 GN=ARPC3 PE=1 SV=3	3	1,114	0,892	0,725	4235969,261	4541050,974	6638445,339
Q9Y5S9	RBM8A	RNA-binding protein 8A OS=Homo sapiens OX=9606 GN=RBM8A PE=1 SV=1	6	0,566	1,162	0,725	20458917,26	11323362,78	16084536,35
Q14839	CHD4	Chromodomain-helicase-DNA-binding protein 4 OS=Homo sapiens OX=9606 GN=CHD4 PE=1 SV=	22	0,381	1,711	0,725	31003138,55	10029051,64	15968305,51
P08574	CYC1	Cytochrome c1, heme protein, mitochondrial OS=Homo sapiens OX=9606 GN=CYC1 PE=1 SV=3	6	0,996	0,84	0,724	7843382,969	4985641,61	10773212,05
Q5JSZ5	PRRC2B	Protein PRRC2B OS=Homo sapiens OX=9606 GN=PRRC2B PE=1 SV=2	2	0,647	1,155	0,724	307251,0938	198741,1145	265921,2463
O00178	GTPBP1	GTP-binding protein 1 OS=Homo sapiens OX=9606 GN=GTPBP1 PE=1 SV=3	2	0,397	1,806	0,723	1266828,717	515450,6299	766811,5111
Q9NRZ7	AGPAT3	1-acyl-sn-glycerol-3-phosphate acyltransferase gamma OS=Homo sapiens OX=9606 GN=AGPAT3	4	1,953	0,709	0,722	506344,5533	621217,4944	1640382,307
Q9BTU6	PI4K2A	Phosphatidylinositol 4-kinase type 2-alpha OS=Homo sapiens OX=9606 GN=PI4K2A PE=1 SV=1	11	1,321	0,684	0,722	1788923,706	3234679,334	2683010,256
O94906	PRPF6	Pre-mRNA-processing factor 6 OS=Homo sapiens OX=9606 GN=PRPF6 PE=1 SV=1	20	0,768	0,911	0,722	19024220,4	13577438,79	18617218,41
P05114	HMGN1	Non-histone chromosomal protein HMG-14 OS=Homo sapiens OX=9606 GN=HMGN1 PE=1 SV=3	3	0,657	0,76	0,722	2055893,534	2010746,485	1976879,587
Q7Z7F7	MRPL55	Large ribosomal subunit protein mL55 OS=Homo sapiens OX=9606 GN=MRPL55 PE=1 SV=1	3	1,362	0,482	0,721	789739,6735	1167810,443	1644852,835
Q14671	PUM1	Pumilio homolog 1 OS=Homo sapiens OX=9606 GN=PUM1 PE=1 SV=3	7	0,735	1,086	0,721	4816602,534	1872419,975	4159451,672
Q8TCS8	PNPT1	Polyribonucleotide nucleotidyltransferase 1, mitochondrial OS=Homo sapiens OX=9606 GN=PNP	28	1,642	0,392	0,72	7434351,356	11117993,49	25070439,28
P62312	LSM6	U6 snRNA-associated Sm-like protein LSm6 OS=Homo sapiens OX=9606 GN=LSM6 PE=1 SV=1	2	0,446	1,786	0,72	3426499,246	1871357,222	1957882,71
Q9H9P8	L2HGDH	L-2-hydroxyglutarate dehydrogenase, mitochondrial OS=Homo sapiens OX=9606 GN=L2HGDH PE	8	1,487	0,477	0,719	910043,1821	1189040,742	2061294,244
Q9HCS7	XAB2	Pre-mRNA-splicing factor SYF1 OS=Homo sapiens OX=9606 GN=XAB2 PE=1 SV=2	4	0,927	0,686	0,719	1582148,524	1227080,514	2315881,862
A6NCE7	MAP1LC3B2	Microtubule-associated proteins 1A/1B light chain 3 beta 2 OS=Homo sapiens OX=9606 GN=MAI	3	0,396	9,168	0,719	5594175,082	997103,321	588703,3268
Q7Z460	CLASP1	CLIP-associating protein 1 OS=Homo sapiens OX=9606 GN=CLASP1 PE=1 SV=1	6	0,364	2,099	0,718	2045772,778	1043568,479	720441,1201
P14314	PRKCSH	Glucosidase 2 subunit beta OS=Homo sapiens OX=9606 GN=PRKCSH PE=1 SV=2	16	1,695	0,393	0,717	93052610,57	155309981,5	251680399,4
P26440	IVD	Isovaleryl-CoA dehydrogenase, mitochondrial OS=Homo sapiens OX=9606 GN=IVD PE=1 SV=2	10	1,344	0,528	0,717	1959207,32	2261932,641	3749918,592
Q9Y3L5	RAP2C	Ras-related protein Rap-2c OS=Homo sapiens OX=9606 GN=RAP2C PE=1 SV=1	5	1,056	0,754	0,717	5013384,539	3557846,875	7077777,461
O60884	DNAJA2	DnaJ homolog subfamily A member 2 OS=Homo sapiens OX=9606 GN=DNAJA2 PE=1 SV=1	5	0,596	0,775	0,717	3217353,232	3024170,323	4671427,766
Q13188	STK3	Serine/threonine-protein kinase 3 OS=Homo sapiens OX=9606 GN=STK3 PE=1 SV=2	2	1,324	0,517	0,716	305470,9679	399696,6179	1169102,719
Q9Y376	CAB39	Calcium-binding protein 39 OS=Homo sapiens OX=9606 GN=CAB39 PE=1 SV=1	6	1,109	0,636	0,716	1708067,656	1887611,889	2994139,225
O15118	NPC1	NPC intracellular cholesterol transporter 1 OS=Homo sapiens OX=9606 GN=NPC1 PE=1 SV=2	5	1,09	0,622	0,716	487206,5942	447955,3918	1056425,857
Q10570	CPSF1	Cleavage and polyadenylation specificity factor subunit 1 OS=Homo sapiens OX=9606 GN=CPSF1	10	0,602	1,194	0,716	3797906,97	1815559,783	3418332,075
P30084	ECHS1	Enoyl-CoA hydratase, mitochondrial OS=Homo sapiens OX=9606 GN=ECHS1 PE=1 SV=4	11	1,304	0,519	0,715	14304136,68	19224187,02	25144742,66
P12268	IMPDH2	Inosine-5'-monophosphate dehydrogenase 2 OS=Homo sapiens OX=9606 GN=IMPDH2 PE=1 SV=	15	0,591	1,126	0,715	28429120,19	15542393,93	23748184,81
Q96AE4	FUBP1	Far upstream element-binding protein 1 OS=Homo sapiens OX=9606 GN=FUBP1 PE=1 SV=3	15	0,512	1,484	0,715	81588543,18	42170539,13	60861801,45
P62269	RPS18	Small ribosomal subunit protein uS13 OS=Homo sapiens OX=9606 GN=RPS18 PE=1 SV=3	13	0,387	1,491	0,714	122160427,3	53036608,02	78559925,79
P34896	SHMT1	Serine hydroxymethyltransferase, cytosolic OS=Homo sapiens OX=9606 GN=SHMT1 PE=1 SV=1	5	0,379	1,941	0,714	3780930,608	1439349,078	1988200,829
Q9UJZ1	STOML2	Stomatin-like protein 2, mitochondrial OS=Homo sapiens OX=9606 GN=STOML2 PE=1 SV=1	15	1,653	0,409	0,713	18786565,88	27131377,82	51227461,12
Q5SSJ5	HP1BP3	Heterochromatin protein 1-binding protein 3 OS=Homo sapiens OX=9606 GN=HP1BP3 PE=1 SV=	10	0,977	0,786	0,713	17482729,67	14425242,61	22453846,39
O43264	ZW10	Centromere/kinetochore protein zw10 homolog OS=Homo sapiens OX=9606 GN=ZW10 PE=1 SV=	7	0,774	1,29	0,713	1477749,386	764303,0594	986201,6522
Q9UHR4	BAIAP2L1	Brain-specific angiogenesis inhibitor 1-associated protein 2-like protein 1 OS=Homo sapiens OX=	4	1000	0,001	0,712		768203,9034	1308272,679
Q12769	NUP160	Nuclear pore complex protein Nup160 OS=Homo sapiens OX=9606 GN=NUP160 PE=1 SV=3	15	0,871	0,891	0,712	6360725,36	3507412,291	8275030,374
P36915	GNL1	Guanine nucleotide-binding protein-like 1 OS=Homo sapiens OX=9606 GN=GNL1 PE=1 SV=2	2	0,507	1,378	0,712	467149,9808	255302,9084	338887,5224
P11387	TOP1	DNA topoisomerase 1 OS=Homo sapiens OX=9606 GN=TOP1 PE=1 SV=2	14	0,434	1,657	0,712	24089886,85	10722867,99	15488968,32
P62829	RPL23	Large ribosomal subunit protein uL14 OS=Homo sapiens OX=9606 GN=RPL23 PE=1 SV=1	5	0,381	1,915	0,712	29314713,04	10481977,55	15900514,76
Q9NUD5	ZCCHC3	Zinc finger CCHC domain-containing protein 3 OS=Homo sapiens OX=9606 GN=ZCCHC3 PE=1 SV=	3	0,094	7,569	0,712	1075551,715	25962,79441	36460,85873
Q9BXS5	AP1M1	AP-1 complex subunit mu-1 OS=Homo sapiens OX=9606 GN=AP1M1 PE=1 SV=3	6	1,278	1,132	0,711	2817234,415	3274819,725	3457440,066
O75381	PEX14	Peroxisomal membrane protein PEX14 OS=Homo sapiens OX=9606 GN=PEX14 PE=1 SV=1	6	1,112	0,529	0,711	2124453,899	1531097,008	4256030,864
P26196	DDX6	Probable ATP-dependent RNA helicase DDX6 OS=Homo sapiens OX=9606 GN=DDX6 PE=1 SV=2	9	0,488	1,628	0,711	18814505,33	8195262,075	11823900,74
O15270	SPTLC2	Serine palmitoyltransferase 2 OS=Homo sapiens OX=9606 GN=SPTLC2 PE=1 SV=1	10	1,315	0,499	0,71	1793058,561	2281021,645	4722479,943
P11310	ACADM	Medium-chain specific acyl-CoA dehydrogenase, mitochondrial OS=Homo sapiens OX=9606 GN=	11	0,553	1,31	0,71	18833289,34	11368012,31	13159159,46
Q14444	CAPRIN1	Caprin-1 OS=Homo sapiens OX=9606 GN=CAPRIN1 PE=1 SV=2	12	0,592	1,117	0,709	27406962,81	18585937,77	27229642,17
Q9Y3U8	RPL36	Large ribosomal subunit protein eL36 OS=Homo sapiens OX=9606 GN=RPL36 PE=1 SV=3	2	0,574	1,194	0,709	6717006,711	3902929,193	6202795,686
P11233	RALA	Ras-related protein Ral-A OS=Homo sapiens OX=9606 GN=RALA PE=1 SV=1	2	1,68	0,417	0,707	4672134,328	8337068,77	10995247,07

P14927	UQCRB	Cytochrome b-c1 complex subunit 7 OS=Homo sapiens OX=9606 GN=UQCRB PE=1 SV=2	5	1,173	0,546	0,707	8338416,679	9985674,742	14820895,19
P62910	RPL32	Large ribosomal subunit protein eL32 OS=Homo sapiens OX=9606 GN=RPL32 PE=1 SV=2	2	0,585	1,217	0,706	2013120,789	1162364,174	1804958,656
Q13363	CTBP1	C-terminal-binding protein 1 OS=Homo sapiens OX=9606 GN=CTBP1 PE=1 SV=2	4	0,555	1,425	0,706	2564218,899	1301156,042	1696389,344
Q9Y3B7	MRPL11	Large ribosomal subunit protein uL11m OS=Homo sapiens OX=9606 GN=MRPL11 PE=1 SV=1	7	1,356	0,546	0,705	4554610,072	5447133,817	8089545,134
Q9UH65	SWAP70	Switch-associated protein 70 OS=Homo sapiens OX=9606 GN=SWAP70 PE=1 SV=1	9	0,59	1,53	0,705	3049597,555	1576676,752	2159297,241
Q96GM5	SMARCD1	SWI/SNF-related matrix-associated actin-dependent regulator of chromatin subfamily D membe	4	0,191	3,389	0,705	3006108,212	409727,8389	328200,3151
Q13309	SKP2	S-phase kinase-associated protein 2 OS=Homo sapiens OX=9606 GN=SKP2 PE=1 SV=2	5	0,114	9,283	0,705	3014892,024	359752,3656	225309,9174
Q96C36	PYCR2	Pyrroline-5-carboxylate reductase 2 OS=Homo sapiens OX=9606 GN=PYCR2 PE=1 SV=1	5	1,341	0,562	0,704	3002205,245	4026677,592	5671795,838
Q5T8P6	RBM26	RNA-binding protein 26 OS=Homo sapiens OX=9606 GN=RBM26 PE=1 SV=3	3	0,518	1,237	0,703	1043155,122	632664,014	833045,7652
O00193	SMAP	Small acidic protein OS=Homo sapiens OX=9606 GN=SMAP PE=1 SV=1	2	0,329	1,041	0,703	1513338,684	328428,2352	1447384,265
Q8WUF5	PPP1R13L	RelA-associated inhibitor OS=Homo sapiens OX=9606 GN=PPP1R13L PE=1 SV=4	10	1,712	0,744	0,701	4529665,464	9898149,747	7307569,442
Q6UB35	MTHFD1L	Monofunctional C1-tetrahydrofolate synthase, mitochondrial OS=Homo sapiens OX=9606 GN=M	11	1,593	0,482	0,701	5114753,71	9132391,942	11269534
Q13347	EIF3I	Eukaryotic translation initiation factor 3 subunit I OS=Homo sapiens OX=9606 GN=EIF3I PE=1 SV=	11	0,598	1,471	0,7	16364262,14	8426261,968	11667240,19
Q4G0N4	NADK2	NAD kinase 2, mitochondrial OS=Homo sapiens OX=9606 GN=NADK2 PE=1 SV=2	5	1,371	0,607	0,699	1628224,992	2690074,412	3282339,66
Q9UBX3	SLC25A10	Mitochondrial dicarboxylate carrier OS=Homo sapiens OX=9606 GN=SLC25A10 PE=1 SV=2	4	1,257	0,711	0,699	1173859,882	1041456,149	1691035,554
Q9UNK0	STX8	Syntaxin-8 OS=Homo sapiens OX=9606 GN=STX8 PE=1 SV=2	2	1,175	0,53	0,699	829486,6176	949483,4816	1378695,931
O60506	SYNCRIP	Heterogeneous nuclear ribonucleoprotein Q OS=Homo sapiens OX=9606 GN=SYNCRIP PE=1 SV=	20	1,037	0,724	0,699	80772064,01	74565354,09	108725728,8
Q96A33	CCDC47	PAT complex subunit CCDC47 OS=Homo sapiens OX=9606 GN=CCDC47 PE=1 SV=1	12	0,992	0,821	0,698	5596124,456	4891156,45	5240668,253
Q16595	FXN	Frataxin, mitochondrial OS=Homo sapiens OX=9606 GN=FXN PE=1 SV=2	3	1,228	0,578	0,697	545461,0106	493325,6326	910571,5106
Q7L8L6	FASTKD5	FAST kinase domain-containing protein 5, mitochondrial OS=Homo sapiens OX=9606 GN=FASTKD	3	0,978	0,748	0,697	242088,6747	216995,3942	653937,1246
Q12874	SF3A3	Splicing factor 3A subunit 3 OS=Homo sapiens OX=9606 GN=SF3A3 PE=1 SV=1	7	0,632	1,041	0,697	15494161,22	9720135,537	15628072,98
Q9P289	STK26	Serine/threonine-protein kinase 26 OS=Homo sapiens OX=9606 GN=STK26 PE=1 SV=2	4	0,466	1,582	0,696	3335271,749	1030825,769	1395217,905
Q08174	PCDH1	Protocadherin-1 OS=Homo sapiens OX=9606 GN=PCDH1 PE=1 SV=2	3	3,583	0,399	0,695	350879,7159	2163977,07	1379512,095
P43307	SSR1	Translocon-associated protein subunit alpha OS=Homo sapiens OX=9606 GN=SSR1 PE=1 SV=3	4	1,845	0,33	0,695	6257742,116	11222854,8	16490936,74
Q08257	CRYZ	Quinone oxidoreductase OS=Homo sapiens OX=9606 GN=CRYZ PE=1 SV=1	8	0,775	0,966	0,695	6240138,96	3412166,328	6710790,219
O75533	SF3B1	Splicing factor 3B subunit 1 OS=Homo sapiens OX=9606 GN=SF3B1 PE=1 SV=3	45	0,744	1,046	0,695	50154995,35	35741762,22	49475305,25
P29966	MARCKS	Myristoylated alanine-rich C-kinase substrate OS=Homo sapiens OX=9606 GN=MARCKS PE=1 SV=	12	0,7	1,183	0,695	56976574,15	44273107,53	51224305,32
Q8TEX9	IPO4	Importin-4 OS=Homo sapiens OX=9606 GN=IPO4 PE=1 SV=2	5	0,289	2,92	0,695	2270423,503	574307,8292	881757,2472
Q96HS1	PGAM5	Serine/threonine-protein phosphatase PGAM5, mitochondrial OS=Homo sapiens OX=9606 GN=P	8	0,67	0,919	0,694	15077057,44	12417283,5	15572708,38
O95573	ACSL3	Fatty acid CoA ligase Acsl3 OS=Homo sapiens OX=9606 GN=ACSL3 PE=1 SV=3	19	1,315	0,515	0,693	19004071	23975438,87	36815974,87
Q14847	LASP1	LIM and SH3 domain protein 1 OS=Homo sapiens OX=9606 GN=LASP1 PE=1 SV=2	11	0,972	0,944	0,693	21082114,12	15980561,37	19380222,3
Q9UIG0	BAZ1B	Tyrosine-protein kinase BAZ1B OS=Homo sapiens OX=9606 GN=BAZ1B PE=1 SV=2	16	0,534	1,37	0,693	13306052,11	7156489,737	10573938,47
P51116	FXR2	RNA-binding protein FXR2 OS=Homo sapiens OX=9606 GN=FXR2 PE=1 SV=2	2	0,52	1,15	0,693	883271,1345	552736,1316	889349,1209
P46783	RPS10	Small ribosomal subunit protein eS10 OS=Homo sapiens OX=9606 GN=RPS10 PE=1 SV=1	5	0,717	1,245	0,692	43781280,2	18252488,02	34329737,88
Q13685	AAMP	Angio-associated migratory cell protein OS=Homo sapiens OX=9606 GN=AAMP PE=1 SV=2	2	0,621	1,114	0,692	256440,1176	159288,5	230122,3319
Q00688	FKBP3	Peptidyl-prolyl cis-trans isomerase FKBP3 OS=Homo sapiens OX=9606 GN=FKBP3 PE=1 SV=1	7	0,421	1,562	0,692	27481346,17	12886955,28	19022163,7
Q13144	EIF2B5	Translation initiation factor eIF2B subunit epsilon OS=Homo sapiens OX=9606 GN=EIF2B5 PE=1 S	2	0,516	1,281	0,691	177657,7472	91676,53601	120007,0553
Q04323	UBXN1	UBX domain-containing protein 1 OS=Homo sapiens OX=9606 GN=UBXN1 PE=1 SV=2	4	0,513	1,417	0,691	4790999,627	1262224,497	2884414,014
Q9Y5L0	TNPO3	Transportin-3 OS=Homo sapiens OX=9606 GN=TNPO3 PE=1 SV=3	7	0,352	1,741	0,691	4246673,286	904975,2999	2225303,349
Q9UBV8	PEF1	Peflin OS=Homo sapiens OX=9606 GN=PEF1 PE=1 SV=1	2	0,284	2,47	0,691	1304591,127	391018,191	495677,6586
Q96I99	SUCLG2	Succinate--CoA ligase [GDP-forming] subunit beta, mitochondrial OS=Homo sapiens OX=9606 GN	16	2,071	0,317	0,69	5763124,285	10679461,11	18766834,98
Q9HCC0	MCCC2	Methylcrotonoyl-CoA carboxylase beta chain, mitochondrial OS=Homo sapiens OX=9606 GN=MC	15	1,878	0,4	0,69	2329015,987	5517082,677	7853570,321
P26373	RPL13	Large ribosomal subunit protein eL13 OS=Homo sapiens OX=9606 GN=RPL13 PE=1 SV=4	6	0,782	1,069	0,69	22538802,28	16591388,59	29250679,05
Q14103	HNRNPD	Heterogeneous nuclear ribonucleoprotein D0 OS=Homo sapiens OX=9606 GN=HNRNPD PE=1 SV	11	0,717	1,162	0,69	85299437,66	66846871,34	83927249,23
O14776	TCERG1	Transcription elongation regulator 1 OS=Homo sapiens OX=9606 GN=TCERG1 PE=1 SV=2	11	0,606	1,08	0,69	9538910,992	5075587,35	7583202,064
Q96G03	PGM2	Phosphopentomutase OS=Homo sapiens OX=9606 GN=PGM2 PE=1 SV=4	14	0,42	1,504	0,69	2878342,187	4807604,254	2201906,187
Q12788	TBL3	Transducin beta-like protein 3 OS=Homo sapiens OX=9606 GN=TBL3 PE=1 SV=2	5	0,889	0,89	0,688	1093538,563	863294,1763	2024480,515
P29590	PML	Protein PML OS=Homo sapiens OX=9606 GN=PML PE=1 SV=3	22	6,816	0,134	0,687	3098271,628	19588101,45	29891027,84
P55084	HADHB	Trifunctional enzyme subunit beta, mitochondrial OS=Homo sapiens OX=9606 GN=HADHB PE=1	22	1,314	0,558	0,687	36568582,34	38048567	69331810,97
A0A0B4J2D5	GATD3B	Putative glutamine amidotransferase-like class 1 domain-containing protein 3B, mitochondrial O	5	1,19	0,508	0,687	3433053,199	4013851,45	6968782,903
P82930	MRPS34	Small ribosomal subunit protein mS34 OS=Homo sapiens OX=9606 GN=MRPS34 PE=1 SV=2	4	0,968	0,561	0,687	1321713,05	1162487,637	1814821,934
Q9Y2W2	WBP11	WW domain-binding protein 11 OS=Homo sapiens OX=9606 GN=WBP11 PE=1 SV=1	5	0,479	1,379	0,687	2879199,63	1654214,444	2119839,549
G5EA30	CELF1	CUG triplet repeat, RNA binding protein 1, isoform CRA_c OS=Homo sapiens OX=9606 GN=CELF1	3	0,415	1,632	0,687	2429334,813	937413,5958	1218645,356
P24666	ACP1	Low molecular weight phosphotyrosine protein phosphatase OS=Homo sapiens OX=9606 GN=AC	5	0,252	2,901	0,687	11629585,6	898816,2282	2995590,241
Q9NPL8	TIMMDC1	Complex I assembly factor TIMMDC1, mitochondrial OS=Homo sapiens OX=9606 GN=TIMMDC1 I	6	1,438	0,442	0,686	809235,3804	799084,5523	1721394,303
P13798	APEH	Acylamino-acid-releasing enzyme OS=Homo sapiens OX=9606 GN=APEH PE=1 SV=4	12	1,125	1,176	0,686	6490243,73	3919718,236	4379681,61
O75643	SNRNP200	U5 small nuclear ribonucleoprotein 200 kDa helicase OS=Homo sapiens OX=9606 GN=SNRNP200	55	0,539	1,186	0,686	62321167,11	28247906,47	52716413,79
P62899	RPL31	Large ribosomal subunit protein eL31 OS=Homo sapiens OX=9606 GN=RPL31 PE=1 SV=1	5	0,347	2,976	0,686	28509197,86	13928641,46	18470689,5
Q14142	TRIM14	Tripartite motif-containing protein 14 OS=Homo sapiens OX=9606 GN=TRIM14 PE=1 SV=2	2	1000	0,001	0,685		636464,518	890802,0697
Q96JB5	CDK5RAP3	CDK5 regulatory subunit-associated protein 3 OS=Homo sapiens OX=9606 GN=CDK5RAP3 PE=1 S	12	1,833	0,448	0,685	3211908,227	4027655,732	8101570,203
P16220	CREB1	Cyclic AMP-responsive element-binding protein 1 OS=Homo sapiens OX=9606 GN=CREB1 PE=1 S	2	0,986	0,668	0,685	1393125,22	1385578,846	2137917,876
P62701	RPS4X	Small ribosomal subunit protein eS4, X isoform OS=Homo sapiens OX=9606 GN=RPS4X PE=1 SV=	6	0,379	1,685	0,685	76043854,92	28584523,94	45847643,61
Q9UG63	ABCF2	ATP-binding cassette sub-family F member 2 OS=Homo sapiens OX=9606 GN=ABCF2 PE=1 SV=2	5	0,868	0,849	0,684	2335571,048	1891911,349	2782895,8
O95400	CD2BP2	CD2 antigen cytoplasmic tail-binding protein 2 OS=Homo sapiens OX=9606 GN=CD2BP2 PE=1 SV	4	0,625	1,163	0,684	1961763,775	1235530,942	1786935,653
Q13057	COASY	Bifunctional coenzyme A synthase OS=Homo sapiens OX=9606 GN=COASY PE=1 SV=4	5	1,89	0,392	0,681	548441,3694	683674,683	1164230,131
Q8IXI1	RHOT2	Mitochondrial Rho GTPase 2 OS=Homo sapiens OX=9606 GN=RHOT2 PE=1 SV=2	6	1,328	0,483	0,681	3772102,48	2897890,652	6697460,534
Q14318	FKBP8	Peptidyl-prolyl cis-trans isomerase FKBP8 OS=Homo sapiens OX=9606 GN=FKBP8 PE=1 SV=2	5	1,305	0,509	0,681	3802814,114	5404636,323	7411916,892
P38919	EIF4A3	Eukaryotic initiation factor 4A-III OS=Homo sapiens OX=9606 GN=EIF4A3 PE=1 SV=4	19	0,809	0,982	0,681	70080951,65	38182647,2	62892040,36
Q9NZB2	FAM120A	Constitutive coactivator of PPAR-gamma-like protein 1 OS=Homo sapiens OX=9606 GN=FAM120	7	3,025	0,251	0,68	844376,1563	3367734,968	4709526,783
Q5HYK3	COQ5	2-methoxy-6-polyprenyl-1,4-benzoquinol methylase, mitochondrial OS=Homo sapiens OX=9606	2	1,689	0,346	0,68	320514,8043	364222,2571	930360,5571
Q15031	LARS2	Leucine--tRNA ligase, mitochondrial OS=Homo sapiens OX=9606 GN=LARS2 PE=1 SV=2	8	1,531	0,397	0,68	1559126,444	1523204,695	4777258,056
Q3ZCQ8	TIMM50	Mitochondrial import inner membrane translocase subunit TIM50 OS=Homo sapiens OX=9606 G	7	1,165	0,633	0,68	4942763,26	6745284,191	8429864,488
Q8N7H5	PAF1	RNA polymerase II-associated factor 1 homolog OS=Homo sapiens OX=9606 GN=PAF1 PE=1 SV=	2	0,665	1,008	0,68	848311,2314	560701,6393	924040,4429
Q9Y5X3	SNX5	Sorting nexin-5 OS=Homo sapiens OX=9606 GN=SNX5 PE=1 SV=1	8	0,257	2,235	0,68	5399381,737	1411920,905	1966325,805
Q53H82	LACTB2	Endoribonuclease LACTB2 OS=Homo sapiens OX=9606 GN=LACTB2 PE=1 SV=2	3	1,958	0,367	0,679	554714,2513	1001575,572	1564499,194
O94874	UFL1	E3 UFM1-protein ligase 1 OS=Homo sapiens OX=9606 GN=UFL1 PE=1 SV=2	12	1,436	0,391	0,679	2122561,209	2127639,618	4550516,292
O95674	CDS2	Phosphatidate cytidylyltransferase 2 OS=Homo sapiens OX=9606 GN=CDS2 PE=1 SV=1	4	0,742	0,746	0,679	2716907,689	2221274,989	3154132,56
Q9BPX3	NCAPG	Condensin complex subunit 3 OS=Homo sapiens OX=9606 GN=NCAPG PE=1 SV=1	4	0,74	0,922	0,679	1195127,38	195226,3963	1963956,053
P30040	ERP29	Endoplasmic reticulum resident protein 29 OS=Homo sapiens OX=9606 GN=ERP29 PE=1 SV=4	9	0,634	1,079	0,679	51177015,4	36906616,83	49765707,76
O43684	BUB3	Mitotic checkpoint protein BUB3 OS=Homo sapiens OX=9606 GN=BUB3 PE=1 SV=1	7	0,518	1,081	0,679	8177137,302	4343560,798	7692608,065
Q9Y241	HIGD1A	HIG1 domain family member 1A, mitochondrial OS=Homo sapiens OX=9606 GN=HIGD1A PE=1 SV	2	1,385	0,454	0,678	41172,71292	56945,2047	98283,91392
Q09161	NCBP1	Nuclear cap-binding protein subunit 1 OS=Homo sapiens OX=9606 GN=NCBP1 PE=1 SV=1	8	0,918	0,811	0,678	6029923,866	5039917,573	7940129,838
Q9Y3I1	FBXO7	F-box only protein 7 OS=Homo sapiens OX=9606 GN=FBXO7 PE=1 SV=1	3	0,884	0,815	0,678	847787,5825	701111,3277	683051,5051
P62847	RPS24	Small ribosomal subunit protein eS24 OS=Homo sapiens OX=9606 GN=RPS24 PE=1 SV=1	3	0,409	1,34	0,678	5594835,177	2309677,486	3626404,203
Q14728	MFSD10	Major facilitator superfamily domain-containing protein 10 OS=Homo sapiens OX=9606 GN=MFS	2	2,633	0,6	0,677	359696,4505	688075,11	1078871,893
P23229	ITGA6	Integrin alpha-6 OS=Homo sapiens OX=9606 GN=ITGA6 PE=1 SV=5	26	1,656	0,416	0,676	5345271,195	10095312,78	18448032,65
Q96S66	CLCC1	Chloride channel CLIC-like protein 1 OS=Homo sapiens OX=9606 GN=CLCC1 PE=1 SV=1	7	1,177	0,608	0,676	2010255,703	2037784,449	2827639,395
P20042	EIF2S2	Eukaryotic translation initiation factor 2 subunit 2 OS=Homo sapiens OX=9606 GN=EIF2S2 PE=1 S	14	0,851	0,749	0,676	19790986,84	11208851,82	25544485,47
A3KMH1	VWA8	von Willebrand factor A domain-containing protein 8 OS=Homo sapiens OX=9606 GN=VWA8 PE=	13	0,833	0,82	0,676	2363164,932	1716831,891	2637921,605
Q9Y2Q9	MRPS28	Small ribosomal subunit protein bS1m OS=Homo sapiens OX=9606 GN=MRPS28 PE=1 SV=1	4	0,796	0,598	0,676	1859157,778	2177253,28	4579033,888
Q9BTE6	AARSD1	Alanyl-tRNA editing protein Aarsd1 OS=Homo sapiens OX=9606 GN=AARSD1 PE=1 SV=2	3	0,289	2,435	0,676	1087123,889	233726,0475	338785,8089
Q9P258	RCC2	Protein RCC2 OS=Homo sapiens OX=9606 GN=RCC2 PE=1 SV=2	9	0,091	2,847	0,676	12777180,82	2079452,727	4695686,757
Q8IWX8	CHERP	Calcium homeostasis endoplasmic reticulum protein OS=Homo sapiens OX=9606 GN=CHERP PE=	3	1,281	0,343	0,675	1543867,148	2337664,95	3720463,65
P18615	NELFE	Negative elongation factor E OS=Homo sapiens OX=9606 GN=NELFE PE=1 SV=3	3	0,969	1,123	0,675	852867,443	650982,2112	385855,9716
O75312	ZPR1	Zinc finger protein ZPR1 OS=Homo sapiens OX=9606 GN=ZPR1 PE=1 SV=1	2	0,436	1,495	0,675	928939,8227	381863,7053	619020,4075
Q12981	BNIP1	Vesicle transport protein SEC20 OS=Homo sapiens OX=9606 GN=BNIP1 PE=1 SV=3	2	1,251	0,54	0,674	877199,2459	938343,9024	1882191,806
Q8NFH4	NUP37	Nucleoporin Nup37 OS=Homo sapiens OX=9606 GN=NUP37 PE=1 SV=1	5	0,861	0,726	0,674	2594413,17	1031109,121	3178762,091
P24534	EEF1B2	Elongation factor 1-beta OS=Homo sapiens OX=9606 GN=EEF1B2 PE=1 SV=3	6	0,591	1,218	0,674	48012290,74	22538197,64	38698919,2
P17844	DDX5	Probable ATP-dependent RNA helicase DDX5 OS=Homo sapiens OX=9606 GN=DDX5 PE=1 SV=1	18	0,5	1,305	0,674	124335207,5	45030610,09	93495162,42
O14907	TAX1BP3	Tax1-binding protein 3 OS=Homo sapiens OX=9606 GN=TAX1BP3 PE=1 SV=2	2	2,123	0,334	0,673	701584,0521	1489115,184	2211941,356
Q8IXM3	MRPL41	Large ribosomal subunit protein mL41 OS=Homo sapiens OX=9606 GN=MRPL41 PE=1 SV=1	4	0,957	0,526	0,673	1441086,866	1498918,758	3287230,378
Q9NTJ3	SMC4	Structural maintenance of chromosomes protein 4 OS=Homo sapiens OX=9606 GN=SMC4 PE=1 S	15	0,802	1,023	0,673	6882834,208	5437251,59	15565613,51
P00491	PNP	Purine nucleoside phosphorylase OS=Homo sapiens OX=9606 GN=PNP PE=1 SV=2	10	0,379	2,234	0,673	11696107,33	3052392,913	8494194,316
P60033	CD81	CD81 antigen OS=Homo sapiens OX=9606 GN=CD81 PE=1 SV=1	2	7,105	0,093	0,672	707015,8264	2963017,829	4973305,364
Q8WVB6	CHTF18	Chromosome transmission fidelity protein 18 homolog OS=Homo sapiens OX=9606 GN=CHTF18	2	0,551	1,07	0,672	196019,5534	105733,7692	495638,1356
Q6STE5	SMARCD3	SWI/SNF-related matrix-associated actin-dependent regulator of chromatin subfamily D membe	2	0,326	2,069	0,672	1514797,914	472016,3326	698351,0123
Q92615	LARP4B	La-related protein 4B OS=Homo sapiens OX=9606 GN=LARP4B PE=1 SV=3	2	1,674	0,437	0,671	1507180,181	5932446,675	4544999,743
Q86YP4	GATAD2A	Transcriptional repressor p66-alpha OS=Homo sapiens OX=9606 GN=GATAD2A PE=1 SV=1	6	0,595	1,224	0,671	5262359,112	3575013,999	4161278,197
Q9NR56	MBNL1	Muscleblind-like protein 1 OS=Homo sapiens OX=9606 GN=MBNL1 PE=1 SV=2	4	0,305	0,686	0,671	104530,655	1567803,245	256911,5815
Q9P0M6	MACROH2A2	Core histone macro-H2A.2 OS=Homo sapiens OX=9606 GN=MACROH2A2 PE=1 SV=3	9	0,296	2,218	0,671	21264290,63	5894835,178	8920105,952
Q9NUQ6	SPATS2L	SPATS2-like protein OS=Homo sapiens OX=9606 GN=SPATS2L PE=1 SV=2	2	1000	0,001	0,67		539676,7723	935344,0165

O95292	VAPB	Vesicle-associated membrane protein-associated protein B/C OS=Homo sapiens OX=9606 GN=V/	8	1,038	0,66	0,67	20327468,1	17858201,53	36698832,34
P50570	DNM2	Dynamin-2 OS=Homo sapiens OX=9606 GN=DNM2 PE=1 SV=2	11	1,034	0,613	0,67	8329766,782	6513569,754	10700652,62
Q5K651	SAMD9	Sterile alpha motif domain-containing protein 9 OS=Homo sapiens OX=9606 GN=SAMD9 PE=1 SV	7	2,934	0,075	0,669	92553,9675	2523947,488	3886812,861
Q9Y2R0	COA3	Cytochrome c oxidase assembly factor 3 homolog, mitochondrial OS=Homo sapiens OX=9606 GN	5	1,975	0,413	0,669	1998126,887	4102134,56	10045198,2
Q96A72	MAGOHB	Protein mago nashi homolog 2 OS=Homo sapiens OX=9606 GN=MAGOHB PE=1 SV=1	8	0,673	1,034	0,669	22839213,61	15286602,11	21370874,17
P22695	UQCRC2	Cytochrome b-c1 complex subunit 2, mitochondrial OS=Homo sapiens OX=9606 GN=UQCRC2 PE=	13	1,361	0,544	0,668	30248937,78	38203819,14	50416403,84
O96008	TOMM40	Mitochondrial import receptor subunit TOM40 homolog OS=Homo sapiens OX=9606 GN=TOMM	9	1,28	0,56	0,668	12613454,24	11141943,69	22462865,49
Q6NVY1	HIBCH	3-hydroxyisobutyryl-CoA hydrolase, mitochondrial OS=Homo sapiens OX=9606 GN=HIBCH PE=1 S	9	0,547	1,042	0,668	3664732,176	2114059,619	4375844,318
Q9BQ95	ECSIT	Evolutionarily conserved signaling intermediate in Toll pathway, mitochondrial OS=Homo sapien	5	1,263	0,477	0,667	810090,2529	987266,3572	2263186,463
Q96B26	EXOSC8	Exosome complex component RRP43 OS=Homo sapiens OX=9606 GN=EXOSC8 PE=1 SV=1	3	0,855	0,729	0,667	2211897,884	1979793,594	3274953,185
Q9H444	CHMP4B	Charged multivesicular body protein 4b OS=Homo sapiens OX=9606 GN=CHMP4B PE=1 SV=1	5	1,174	0,509	0,666	5850834,826	6574288,294	10388251,84
P51532	SMARCA4	Transcription activator BRG1 OS=Homo sapiens OX=9606 GN=SMARCA4 PE=1 SV=2	15	0,356	2,094	0,666	13277431,81	3536866,718	6542904,593
Q92797	SYMPK	Symplekin OS=Homo sapiens OX=9606 GN=SYMPK PE=1 SV=2	11	1,124	0,708	0,664	3127059,432	3454000,072	6718008,544
Q9BXY0	MAK16	Protein MAK16 homolog OS=Homo sapiens OX=9606 GN=MAK16 PE=1 SV=2	3	1,033	0,783	0,664	938889,2165	813783,5442	1225649,467
Q9H3K2	GHITM	Growth hormone-inducible transmembrane protein OS=Homo sapiens OX=9606 GN=GHITM PE=	3	0,866	0,775	0,664	1883754,292	1113044,973	2728986,198
Q15738	NSDHL	Sterol-4-alpha-carboxylate 3-dehydrogenase, decarboxylating OS=Homo sapiens OX=9606 GN=N	7	0,621	1,116	0,664	10477275,01	6198774,123	10135338,16
Q9BYD1	MRPL13	Large ribosomal subunit protein uL13m OS=Homo sapiens OX=9606 GN=MRPL13 PE=1 SV=1	8	0,615	0,645	0,664	2640786,038	2280713,536	3970720,459
P30419	NMT1	Glycylpeptide N-tetradecanoyltransferase 1 OS=Homo sapiens OX=9606 GN=NMT1 PE=1 SV=2	5	0,536	1,136	0,663	3998250,905	2163999,279	3526607,992
O95563	MPC2	Mitochondrial pyruvate carrier 2 OS=Homo sapiens OX=9606 GN=MPC2 PE=1 SV=1	2	1,882	0,351	0,662	319320,9995	581607,5052	880170,1864
P35658	NUP214	Nuclear pore complex protein Nup214 OS=Homo sapiens OX=9606 GN=NUP214 PE=1 SV=2	15	0,741	0,718	0,662	7138114,512	5156124,41	9186078,321
Q9Y230	RUVBL2	RuvB-like 2 OS=Homo sapiens OX=9606 GN=RUVBL2 PE=1 SV=3	17	0,822	0,874	0,661	32987986,59	25471068,48	40545931,41
P24928	POLR2A	DNA-directed RNA polymerase II subunit RPB1 OS=Homo sapiens OX=9606 GN=POLR2A PE=1 SV	17	0,762	0,928	0,661	6060207,025	4383689,054	7201311,276
P07311	ACYP1	Acylphosphatase-1 OS=Homo sapiens OX=9606 GN=ACYP1 PE=1 SV=2	3	0,387	3,396	0,661	3330059,243	1131894,579	1518749,886
Q9Y5Q8	GTF3C5	General transcription factor 3C polypeptide 5 OS=Homo sapiens OX=9606 GN=GTF3C5 PE=1 SV=	3	0,508	1,323	0,66	713799,669	375419,8601	579268,7022
P23497	SP100	Nuclear autoantigen Sp-100 OS=Homo sapiens OX=9606 GN=SP100 PE=1 SV=3	9	0,505	0,37	0,659	10925622,12	10018614,11	12225982,05
Q92621	NUP205	Nuclear pore complex protein Nup205 OS=Homo sapiens OX=9606 GN=NUP205 PE=1 SV=3	27	0,912	0,736	0,658	17787658,81	14305371,6	24058855,44
Q08380	LGALS3BP	Galectin-3-binding protein OS=Homo sapiens OX=9606 GN=LGALS3BP PE=1 SV=1	9	8,67	0,116	0,657	2236279,978	17336325,29	25281672,92
Q9UHA4	LAMTOR3	Ragulator complex protein LAMTOR3 OS=Homo sapiens OX=9606 GN=LAMTOR3 PE=1 SV=1	5	1,213	0,532	0,657	2010887,477	2220951,475	3177191,232
Q01581	HMGCS1	Hydroxymethylglutaryl-CoA synthase, cytoplasmic OS=Homo sapiens OX=9606 GN=HMGCS1 PE=	15	0,026	20,276	0,657	126970718,5	2309335,152	4645657,968
P19634	SLC9A1	Sodium/hydrogen exchanger 1 OS=Homo sapiens OX=9606 GN=SLC9A1 PE=1 SV=2	5	2,31	0,353	0,656	507669,9813	1223357,761	1545963,527
Q96PU5	NEDD4L	E3 ubiquitin-protein ligase NEDD4-like OS=Homo sapiens OX=9606 GN=NEDD4L PE=1 SV=2	2	1,096	0,593	0,656	333105,0726	194986,0913	548877,2554
Q9HD33	MRPL47	Large ribosomal subunit protein uL29m OS=Homo sapiens OX=9606 GN=MRPL47 PE=1 SV=2	4	1,078	0,644	0,656	1371433,603	1579433,462	3001094,447
Q9Y3B4	SF3B6	Splicing factor 3B subunit 6 OS=Homo sapiens OX=9606 GN=SF3B6 PE=1 SV=1	4	0,644	0,794	0,656	9603171,764	5884181,202	8421007,151
Q14197	MRPL58	Large ribosomal subunit protein mL62 OS=Homo sapiens OX=9606 GN=MRPL58 PE=1 SV=1	3	1,985	0,442	0,655	349297,359	925243,298	1430905,291
Q96DB5	RMDN1	Regulator of microtubule dynamics protein 1 OS=Homo sapiens OX=9606 GN=RMDN1 PE=1 SV=	6	1,813	0,342	0,655	1352363,008	2431276,006	4181060,386
Q9Y320	TMX2	Thioredoxin-related transmembrane protein 2 OS=Homo sapiens OX=9606 GN=TMX2 PE=1 SV=1	5	1,682	0,41	0,655	577997,8108	2387125,084	3810322,009
Q9UJX2	CDC23	Cell division cycle protein 23 homolog OS=Homo sapiens OX=9606 GN=CDC23 PE=1 SV=3	4	1,003	0,675	0,655	903579,6799	914581,5648	1463430,95
Q9UMS0	NFU1	NFU1 iron-sulfur cluster scaffold homolog, mitochondrial OS=Homo sapiens OX=9606 GN=NFU1	2	1,34	0,524	0,654	1633608,425	2133814,312	3239156,578
O95602	POLR1A	DNA-directed RNA polymerase I subunit RPA1 OS=Homo sapiens OX=9606 GN=POLR1A PE=1 SV=	13	0,612	0,83	0,654	1606798,728	1392115,929	3211689,748
P55060	CSE1L	Exportin-2 OS=Homo sapiens OX=9606 GN=CSE1L PE=1 SV=3	27	0,433	1,386	0,654	74558676,25	29667711,03	54030267,88
Q96T23	RSF1	Remodeling and spacing factor 1 OS=Homo sapiens OX=9606 GN=RSF1 PE=1 SV=2	7	0,627	1,039	0,653	1531795,917	851344,0039	1256761,135
Q9H7Z7	PTGES2	Prostaglandin E synthase 2 OS=Homo sapiens OX=9606 GN=PTGES2 PE=1 SV=1	8	1,784	0,278	0,652	2317590,023	4025858,495	10608263,07
O75616	ERAL1	GTPase Era, mitochondrial OS=Homo sapiens OX=9606 GN=ERAL1 PE=1 SV=2	3	1,001	0,679	0,652	1044195,424	746692,6423	1534166,292
P39880	CUX1	Homeobox protein cut-like 1 OS=Homo sapiens OX=9606 GN=CUX1 PE=1 SV=3	4	0,797	0,84	0,652	1230609,727	1003011,21	1510364,759
Q9Y4P3	TBL2	Transducin beta-like protein 2 OS=Homo sapiens OX=9606 GN=TBL2 PE=1 SV=1	6	1,304	0,397	0,651	2311829,399	2628835,704	6087941,169
P22626	HNRNPA2B1	Heterogeneous nuclear ribonucleoproteins A2/B1 OS=Homo sapiens OX=9606 GN=HNRNPA2B1	17	0,978	0,778	0,651	626976227,6	608815453,2	907094388,2
Q96T51	RUFY1	RUN and FYVE domain-containing protein 1 OS=Homo sapiens OX=9606 GN=RUFY1 PE=1 SV=2	5	0,767	0,848	0,65	1297734,316	726164,4264	1631459,929
Q92575	UBXN4	UBX domain-containing protein 4 OS=Homo sapiens OX=9606 GN=UBXN4 PE=1 SV=2	4	0,899	0,669	0,649	1284998,327	1353244,942	2322700,668
O60830	TIMM17B	Mitochondrial import inner membrane translocase subunit Tim17-B OS=Homo sapiens OX=9606	3	0,731	0,739	0,649	1646190,684	852402,8735	1981835,93
Q13442	PDAP1	28 kDa heat- and acid-stable phosphoprotein OS=Homo sapiens OX=9606 GN=PDAP1 PE=1 SV=1	4	0,645	1,125	0,649	3219352,669	1141644,309	2524730,813
P23526	AHCY	Adenosylhomocysteinase OS=Homo sapiens OX=9606 GN=AHCY PE=1 SV=4	15	0,311	2,05	0,649	76807263,42	20973618,51	39943770,03
O15258	RER1	Protein RER1 OS=Homo sapiens OX=9606 GN=RER1 PE=1 SV=1	3	1,403	0,465	0,648	2762904,874	3960806,396	6094268,65
Q9C0E8	LNPK	Endoplasmic reticulum junction formation protein lunapark OS=Homo sapiens OX=9606 GN=LNP	2	1,378	0,66	0,648	784812,9998	223500,4098	1070890,896
Q9P015	MRPL15	Large ribosomal subunit protein uL15m OS=Homo sapiens OX=9606 GN=MRPL15 PE=1 SV=1	9	0,978	0,469	0,648	1959824,697	4683806,551	4880091,311
Q5SWX8	ODR4	Protein odr-4 homolog OS=Homo sapiens OX=9606 GN=ODR4 PE=1 SV=1	3	0,85	0,746	0,648	411603,1237	359493,0689	867405,6941
Q13247	SRSF6	Serine/arginine-rich splicing factor 6 OS=Homo sapiens OX=9606 GN=SRSF6 PE=1 SV=2	3	0,468	1,234	0,648	9500735,147	2879318,705	5948888,133
P13796	LCP1	Plastin-2 OS=Homo sapiens OX=9606 GN=LCP1 PE=1 SV=6	19	1,161	0,312	0,647	6524011,67	12901865,81	21211258,77
Q86X10	RALGAPB	Ral GTPase-activating protein subunit beta OS=Homo sapiens OX=9606 GN=RALGAPB PE=1 SV=1	4	0,381	1,585	0,647	549786,0001	206209,4464	290805,4343
P17677	GAP43	Neuromodulin OS=Homo sapiens OX=9606 GN=GAP43 PE=1 SV=1	5	0,041	7,189	0,647	2779406,296	9844,095526	33321,40567
Q9P2I0	CPSF2	Cleavage and polyadenylation specificity factor subunit 2 OS=Homo sapiens OX=9606 GN=CPSF2	4	0,629	0,963	0,646	1482538,189	724717,622	1544596,693
Q02543	RPL18A	Large ribosomal subunit protein eL20 OS=Homo sapiens OX=9606 GN=RPL18A PE=1 SV=2	5	0,445	1,567	0,646	16536973,04	5745979,575	8583254,595
P51589	CYP2J2	Cytochrome P450 2J2 OS=Homo sapiens OX=9606 GN=CYP2J2 PE=1 SV=2	7	0,402	1,605	0,646	165765,6921	63479,12019	99085,55275
O75940	SMNDC1	Survival of motor neuron-related-splicing factor 30 OS=Homo sapiens OX=9606 GN=SMNDC1 PE=	3	0,833	0,796	0,645	3207619,944	1802749,224	4144045,101
O43913	ORC5	Origin recognition complex subunit 5 OS=Homo sapiens OX=9606 GN=ORC5 PE=1 SV=1	3	0,643	0,931	0,645	689150,4319	458759,2423	785001,3034
O00330	PDHX	Pyruvate dehydrogenase protein X component, mitochondrial OS=Homo sapiens OX=9606 GN=P	7	1,278	0,404	0,644	1218700,85	2943703,36	3381050,057
Q9NVH1	DNAJC11	DnaJ homolog subfamily C member 11 OS=Homo sapiens OX=9606 GN=DNAJC11 PE=1 SV=2	8	0,843	0,688	0,644	1478918,642	2093575,215	4134438,448
P62304	SNRPE	Small nuclear ribonucleoprotein E OS=Homo sapiens OX=9606 GN=SNRPE PE=1 SV=1	2	0,642	1,034	0,644	19949088,38	11541429,45	17098783,54
P51398	DAP3	Small ribosomal subunit protein mS29 OS=Homo sapiens OX=9606 GN=DAP3 PE=1 SV=1	11	1,206	0,538	0,643	5427722,12	9559712,905	11742409,42
Q6ZRP7	QSOX2	Sulfhydryl oxidase 2 OS=Homo sapiens OX=9606 GN=QSOX2 PE=1 SV=3	5	1,192	0,579	0,642	913696,4162	832363,8882	1781477,06
Q8N5K1	CISD2	CDGSH iron-sulfur domain-containing protein 2 OS=Homo sapiens OX=9606 GN=CISD2 PE=1 SV=	5	1,148	0,658	0,642	7863761,373	5213856,264	9986072,045
Q8IY81	FTSJ3	pre-rRNA 2'-O-ribose RNA methyltransferase FTSJ3 OS=Homo sapiens OX=9606 GN=FTSJ3 PE=1 S	9	0,508	1,322	0,642	1829487,609	1443525,137	2944033,974
P05455	SSB	Lupus La protein OS=Homo sapiens OX=9606 GN=SSB PE=1 SV=2	21	0,429	1,496	0,642	33258904,88	14085183,86	24609100,41
P54819	AK2	Adenylate kinase 2, mitochondrial OS=Homo sapiens OX=9606 GN=AK2 PE=1 SV=2	11	1,972	0,309	0,641	9843867,604	16252073,97	31547355,24
Q86TS9	MRPL52	Large ribosomal subunit protein mL52 OS=Homo sapiens OX=9606 GN=MRPL52 PE=1 SV=2	2	0,834	0,934	0,641	224299,4507	278217,9324	380065,604
P07910	HNRNPC	Heterogeneous nuclear ribonucleoproteins C1/C2 OS=Homo sapiens OX=9606 GN=HNRNPC PE=	17	1,07	0,572	0,64	213038013,2	194492684,7	300259595
Q6ZSR9		Uncharacterized protein FLJ45252 OS=Homo sapiens OX=9606 PE=2 SV=2	2	0,841	0,81	0,64	633091,8624	390932,0884	621782,2063
Q14651	PLS1	Plastin-1 OS=Homo sapiens OX=9606 GN=PLS1 PE=1 SV=2	9	0,703	1,081	0,64	1159937,896	736660,2038	1041919,358
Q05655	PRKCD	Protein kinase C delta type OS=Homo sapiens OX=9606 GN=PRKCD PE=1 SV=2	2	1,964	0,314	0,639	36281,53935	70487,9903	115702,2066
Q9UBU8	MORF4L1	Mortality factor 4-like protein 1 OS=Homo sapiens OX=9606 GN=MORF4L1 PE=1 SV=2	3	0,727	0,755	0,639	2261762,602	1363186,534	2826039,403
Q96KG9	SCYL1	N-terminal kinase-like protein OS=Homo sapiens OX=9606 GN=SCYL1 PE=1 SV=1	3	0,592	1,806	0,639	628635,8346	126800,144	399083,5408
Q9BZE1	MRPL37	Large ribosomal subunit protein mL37 OS=Homo sapiens OX=9606 GN=MRPL37 PE=1 SV=2	9	1,186	0,547	0,638	3708956,453	4479456,571	8219087,498
P68400	CSNK2A1	Casein kinase II subunit alpha OS=Homo sapiens OX=9606 GN=CSNK2A1 PE=1 SV=1	13	0,767	0,931	0,638	16502500,28	10224451,03	17346349,41
P48681	NES	Nestin OS=Homo sapiens OX=9606 GN=NES PE=1 SV=2	29	0,135	5,837	0,638	40365454,39	2250278,139	3015585,451
P42704	LRPPRC	Leucine-rich PPR motif-containing protein, mitochondrial OS=Homo sapiens OX=9606 GN=LRPPF	64	0,741	0,901	0,637	139835753,7	102622676,2	165654202
P23919	DTYMK	Thymidylate kinase OS=Homo sapiens OX=9606 GN=DTYMK PE=1 SV=4	6	0,592	1,265	0,637	4192471,234	1906694,913	3504375,16
O60832	DKC1	H/ACA ribonucleoprotein complex subunit DKC1 OS=Homo sapiens OX=9606 GN=DKC1 PE=1 SV=	12	0,58	1,127	0,637	4381727,431	2341995,237	3581975,92
Q8WXI9	GATAD2B	Transcriptional repressor p66-beta OS=Homo sapiens OX=9606 GN=GATAD2B PE=1 SV=1	3	0,56	1,064	0,637	1892669,599	649873,2605	1707376,447
Q01469	FABP5	Fatty acid-binding protein 5 OS=Homo sapiens OX=9606 GN=FABP5 PE=1 SV=3	2	0,105	5,46	0,637	6332675,462	654431,8147	1176786,189
P41219	PRPH	Peripherin OS=Homo sapiens OX=9606 GN=PRPH PE=1 SV=2	8	0,067	1,514	0,637	7881871,91	1726690,495	3351952,217
Q08722	CD47	Leukocyte surface antigen CD47 OS=Homo sapiens OX=9606 GN=CD47 PE=1 SV=1	3	2,335	0,242	0,636	1500748,613	6541684,901	10986677,17
P52948	NUP98	Nuclear pore complex protein Nup98-Nup96 OS=Homo sapiens OX=9606 GN=NUP98 PE=1 SV=4	15	0,903	0,68	0,636	9201969,195	7712637,554	12705781,41
Q86SX6	GLRX5	Glutaredoxin-related protein 5, mitochondrial OS=Homo sapiens OX=9606 GN=GLRX5 PE=1 SV=2	2	0,893	0,734	0,636	3501172,075	2935360,421	5220450,832
P49327	FASN	Fatty acid synthase OS=Homo sapiens OX=9606 GN=FASN PE=1 SV=3	72	0,096	6,476	0,636	345396010,2	50670828,92	55756686,03
P35914	HMGCL	Hydroxymethylglutaryl-CoA lyase, mitochondrial OS=Homo sapiens OX=9606 GN=HMGCL PE=1 S	2	1,277	0,515	0,635	1127704,793	1026230,438	1781169,462
O43583	DENR	Density-regulated protein OS=Homo sapiens OX=9606 GN=DENR PE=1 SV=2	2	0,637	1,216	0,635	1621396,816	1519944,029	1303902,295
P08670	VIM	Vimentin OS=Homo sapiens OX=9606 GN=VIM PE=1 SV=4	19	0,035	30,806	0,635	173263358,8	2000018,623	4122648,491
P47985	UQCRFS1	Cytochrome b-c1 complex subunit Rieske, mitochondrial OS=Homo sapiens OX=9606 GN=UQCRF	11	1,371	0,607	0,634	15054744,74	17686718,93	30889998,06
P27824	CANX	Calnexin OS=Homo sapiens OX=9606 GN=CANX PE=1 SV=2	26	0,608	0,983	0,634	298721269,8	133010621,8	313049307,4
O95372	LYPLA2	Acyl-protein thioesterase 2 OS=Homo sapiens OX=9606 GN=LYPLA2 PE=1 SV=1	3	0,275	2,815	0,634	2591958,838	893522,7012	1048682,64
Q9Y4W6	AFG3L2	AFG3-like protein 2 OS=Homo sapiens OX=9606 GN=AFG3L2 PE=1 SV=2	19	1,4	0,416	0,633	5890975,659	9908770,954	16160514,43
Q08379	GOLGA2	Golgin subfamily A member 2 OS=Homo sapiens OX=9606 GN=GOLGA2 PE=1 SV=3	7	1,289	0,547	0,633	4330268,383	6297737,676	7538809,141
O95793	STAU1	Double-stranded RNA-binding protein Staufen homolog 1 OS=Homo sapiens OX=9606 GN=STAU	11	0,689	0,924	0,633	11441179,22	6850655,036	11370501,14
P02786	TFRC	Transferrin receptor protein 1 OS=Homo sapiens OX=9606 GN=TFRC PE=1 SV=2	27	1,187	0,551	0,632	46223374,32	49133839,7	80969277,47
Q13330	MTA1	Metastasis-associated protein MTA1 OS=Homo sapiens OX=9606 GN=MTA1 PE=1 SV=2	6	0,339	1,848	0,632	4432417,79	1168279,592	2192765,263
Q9UBU9	NXF1	Nuclear RNA export factor 1 OS=Homo sapiens OX=9606 GN=NXF1 PE=1 SV=1	9	0,774	0,923	0,631	6901141,761	3571886,586	6644605,506
Q9GZR7	DDX24	ATP-dependent RNA helicase DDX24 OS=Homo sapiens OX=9606 GN=DDX24 PE=1 SV=1	4	0,591	0,963	0,631	1748728,891	791692,0206	1602119,179
O75439	PMPCB	Mitochondrial-processing peptidase subunit beta OS=Homo sapiens OX=9606 GN=PMPCB PE=1 S	8	1,031	0,637	0,63	10334270,67	10395267,23	15386461,15
P62995	TRA2B	Transformer-2 protein homolog beta OS=Homo sapiens OX=9606 GN=TRA2B PE=1 SV=1	2	0,412	1,726	0,63	7042158,904	3795665,398	7255607,071
Q86U86	PBRM1	Protein polybromo-1 OS=Homo sapiens OX=9606 GN=PBRM1 PE=1 SV=1	4	0,31	2,086	0,63	1460999,176	343800,6683	472705,5346
Q9NZT1	CALML5	Calmodulin-like protein 5 OS=Homo sapiens OX=9606 GN=CALML5 PE=1 SV=2	2	1,103	0,569	0,629	1050963,981	1158826,896	1841864,178
Q6PKG0	LARP1	La-related protein 1 OS=Homo sapiens OX=9606 GN=LARP1 PE=1 SV=2	16	0,418	1,106	0,629	9168011,706	2681940,028	6602624,514

Q99720	SIGMAR1	Sigma non-opioid intracellular receptor 1 OS=Homo sapiens OX=9606 GN=SIGMAR1 PE=1 SV=1	2	1,905	0,354	0,628	1115481,356	2124710,643	4861187,292
O95684	CEP43	Centrosomal protein 43 OS=Homo sapiens OX=9606 GN=CEP43 PE=1 SV=1	3	1,046	1,068	0,628	559554,8111	306574,0893	651249,7664
P07741	APRT	Adenine phosphoribosyltransferase OS=Homo sapiens OX=9606 GN=APRT PE=1 SV=2	5	0,943	1,155	0,628	2093905,799	1792888,201	1898937,6
O60869	EDF1	Endothelial differentiation-related factor 1 OS=Homo sapiens OX=9606 GN=EDF1 PE=1 SV=1	4	0,568	1,083	0,628	5807762,09	3519080,66	6151658,91
P17050	NAGA	Alpha-N-acetylgalactosaminidase OS=Homo sapiens OX=9606 GN=NAGA PE=1 SV=2	2	1,31	0,48	0,627	978884,7314	1298466,534	2093711,86
Q9H300	PARL	Presenilin-associated rhomboid-like protein, mitochondrial OS=Homo sapiens OX=9606 GN=PAR	2	0,813	0,829	0,627	374019,3944	275907,0654	491533,3784
P10253	GAA	Lysosomal alpha-glucosidase OS=Homo sapiens OX=9606 GN=GAA PE=1 SV=4	9	0,995	0,306	0,626	1677555,261	1496015,154	3811819,922
O00762	UBE2C	Ubiquitin-conjugating enzyme E2 C OS=Homo sapiens OX=9606 GN=UBE2C PE=1 SV=1	3	0,323	1,762	0,626	3892959,53	306915,6752	2382599,611
P09012	SNRPA	U1 small nuclear ribonucleoprotein A OS=Homo sapiens OX=9606 GN=SNRPA PE=1 SV=3	6	0,788	0,728	0,625	19413763,6	14736923,98	24892746,54
Q02880	TOP2B	DNA topoisomerase 2-beta OS=Homo sapiens OX=9606 GN=TOP2B PE=1 SV=3	18	0,283	2,149	0,625	14851776,44	4194355,427	6609797,993
Q9Y3D3	MRPS16	Small ribosomal subunit protein bS16m OS=Homo sapiens OX=9606 GN=MRPS16 PE=1 SV=1	2	1,463	0,425	0,624	780563,8011	1121850,906	1619931,839
Q92979	EMG1	Ribosomal RNA small subunit methyltransferase NEP1 OS=Homo sapiens OX=9606 GN=EMG1 PE	5	0,811	0,798	0,624	2361370,5	1981098,494	3049543,679
Q13573	SNW1	SNW domain-containing protein 1 OS=Homo sapiens OX=9606 GN=SNW1 PE=1 SV=1	8	0,631	0,993	0,624	3251528,141	2050600,571	5235421,939
P62753	RPS6	Small ribosomal subunit protein eS6 OS=Homo sapiens OX=9606 GN=RPS6 PE=1 SV=1	3	0,478	1,381	0,624	20652545,33	7558983,057	16158504,17
P24821	TNC	Tenascin OS=Homo sapiens OX=9606 GN=TNC PE=1 SV=3	2	1,332	0,464	0,623	565836,8174	711742,4895	1185712,392
Q9UBC2	EPS15L1	Epidermal growth factor receptor substrate 15-like 1 OS=Homo sapiens OX=9606 GN=EPS15L1 P	11	1,132	0,514	0,623	2381018,865	2954383,662	5802799,417
Q7Z417	NUFIP2	FMR1-interacting protein NUFIP2 OS=Homo sapiens OX=9606 GN=NUFIP2 PE=1 SV=1	6	0,834	0,731	0,623	1693068,853	1889268,284	2893257,386
P05198	EIF2S1	Eukaryotic translation initiation factor 2 subunit 1 OS=Homo sapiens OX=9606 GN=EIF2S1 PE=1 S	17	0,818	0,856	0,623	26655974,47	20443127,91	28071044,92
Q6P2E9	EDC4	Enhancer of mRNA-decapping protein 4 OS=Homo sapiens OX=9606 GN=EDC4 PE=1 SV=1	8	0,518	1,069	0,623	3747159,021	1753618,961	3186120,414
Q13445	TMED1	Transmembrane emp24 domain-containing protein 1 OS=Homo sapiens OX=9606 GN=TMED1 PE	3	1,261	0,508	0,622	1136087,208	1460862,751	2338041,131
Q99567	NUP88	Nuclear pore complex protein Nup88 OS=Homo sapiens OX=9606 GN=NUP88 PE=1 SV=2	8	0,847	0,707	0,622	4985064,374	3764648,932	7108013,732
Q7L014	DDX46	Probable ATP-dependent RNA helicase DDX46 OS=Homo sapiens OX=9606 GN=DDX46 PE=1 SV=	11	0,643	0,943	0,622	9444552,323	3332156,438	5912250,538
Q8IXI2	RHOT1	Mitochondrial Rho GTPase 1 OS=Homo sapiens OX=9606 GN=RHOT1 PE=1 SV=2	6	0,909	0,721	0,621	1795155,12	1035635,132	2562226,332
Q6UN15	FIP1L1	Pre-mRNA 3'-end-processing factor FIP1 OS=Homo sapiens OX=9606 GN=FIP1L1 PE=1 SV=1	5	0,818	0,689	0,621	2177588,029	1243967,83	3094342,939
Q9Y314	NOSIP	Nitric oxide synthase-interacting protein OS=Homo sapiens OX=9606 GN=NOSIP PE=1 SV=1	2	0,603	0,993	0,621	952802,5103	244548,1757	393658,2913
O94766	B3GAT3	Galactosylgalactosylxylosylprotein 3-beta-glucuronosyltransferase 3 OS=Homo sapiens OX=9606	2	0,378	1,643	0,621	368543,9936	139249,9702	224245,1845
P21127	CDK11B	Cyclin-dependent kinase 11B OS=Homo sapiens OX=9606 GN=CDK11B PE=1 SV=4	5	0,212	0,967	0,621	2195713,161	420527,2643	1638463,043
Q6NUQ4	TMEM214	Transmembrane protein 214 OS=Homo sapiens OX=9606 GN=TMEM214 PE=1 SV=2	5	1,326	0,443	0,62	1074057,951	3316021,941	3217078,842
Q9BTC0	DIDO1	Death-inducer obliterator 1 OS=Homo sapiens OX=9606 GN=DIDO1 PE=1 SV=5	5	1,203	0,532	0,62	616295,6917	749986,4426	1449204,73
Q12906	ILF3	Interleukin enhancer-binding factor 3 OS=Homo sapiens OX=9606 GN=ILF3 PE=1 SV=3	24	0,747	0,804	0,62	113281843	80546401,81	140894950
Q8NBM4	UBAC2	Ubiquitin-associated domain-containing protein 2 OS=Homo sapiens OX=9606 GN=UBAC2 PE=1	3	0,489	1,033	0,62	649442,4306	224779,0621	745500,8314
P0CG08	GPR89B	Golgi pH regulator B OS=Homo sapiens OX=9606 GN=GPR89B PE=1 SV=1	2	1,773	0,368	0,619	761756,3099	1378050,673	1574653,045
Q9BRJ2	MRPL45	Large ribosomal subunit protein mL45 OS=Homo sapiens OX=9606 GN=MRPL45 PE=1 SV=2	7	1,292	0,44	0,619	2800104,723	3487660,717	6344429,675
Q9Y3C1	NOP16	Nucleolar protein 16 OS=Homo sapiens OX=9606 GN=NOP16 PE=1 SV=2	5	0,84	0,727	0,619	895091,878	3526380,96	2378105,091
P46779	RPL28	Large ribosomal subunit protein eL28 OS=Homo sapiens OX=9606 GN=RPL28 PE=1 SV=3	5	0,752	0,881	0,619	14409305,73	9158794,949	17720367,92
Q9NWB6	ARGLU1	Arginine and glutamate-rich protein 1 OS=Homo sapiens OX=9606 GN=ARGLU1 PE=1 SV=1	2	0,75	0,525	0,619	389014,4176	157017,4359	783104,2026
O75569	PRKRA	Interferon-inducible double-stranded RNA-dependent protein kinase activator A OS=Homo sapie	5	0,361	1,683	0,619	3985089,27	754112,6849	1526363,831
Q8NC44	RETREG2	Reticulophagy regulator 2 OS=Homo sapiens OX=9606 GN=RETREG2 PE=1 SV=3	2	1,017	0,534	0,618	306544,7108	311786,5844	639421,6118
P54709	ATP1B3	Sodium/potassium-transporting ATPase subunit beta-3 OS=Homo sapiens OX=9606 GN=ATP1B3	6	0,913	0,707	0,618	13851088,14	11715802,13	18810559,88
P0DP25	CALM3	Calmodulin-3 OS=Homo sapiens OX=9606 GN=CALM3 PE=1 SV=1	9	0,692	0,801	0,618	70973233,64	62458434,92	93768357,25
Q99613	EIF3C	Eukaryotic translation initiation factor 3 subunit C OS=Homo sapiens OX=9606 GN=EIF3C PE=1 SV	16	0,498	1,198	0,618	16061068,05	7592804,205	15429945,51
O75356	ENTPD5	Nucleoside diphosphate phosphatase ENTPD5 OS=Homo sapiens OX=9606 GN=ENTPD5 PE=1 SV=	7	2,366	0,288	0,617	18360,96033	247201,1582	63847,56489
P18124	RPL7	Large ribosomal subunit protein uL30 OS=Homo sapiens OX=9606 GN=RPL7 PE=1 SV=1	13	0,727	0,991	0,617	81253552,85	46929181,88	79009009,03
Q6NXE6	ARMC6	Armadillo repeat-containing protein 6 OS=Homo sapiens OX=9606 GN=ARMC6 PE=1 SV=2	2	0,297	1,933	0,617	28395684,51	4530227,024	13888076,93
Q9H2W6	MRPL46	Large ribosomal subunit protein mL46 OS=Homo sapiens OX=9606 GN=MRPL46 PE=1 SV=1	6	1,592	0,369	0,616	2026387,593	3004839,944	5312016,108
Q9NV56	MRGBP	MRG/MORF4L-binding protein OS=Homo sapiens OX=9606 GN=MRGBP PE=1 SV=1	2	0,658	0,922	0,616	895763,6382	598213,3117	975362,6903
Q15020	SART3	Squamous cell carcinoma antigen recognized by T-cells 3 OS=Homo sapiens OX=9606 GN=SART3	13	0,463	1,258	0,615	6547705,031	1824042,438	3175953,523
P43246	MSH2	DNA mismatch repair protein Msh2 OS=Homo sapiens OX=9606 GN=MSH2 PE=1 SV=1	13	0,278	1,666	0,615	7548255,482	1717044,226	4732011,36
Q5UCC4	EMC10	ER membrane protein complex subunit 10 OS=Homo sapiens OX=9606 GN=EMC10 PE=1 SV=1	2	1,196	0,483	0,614	561255,8788	712498,4489	1161044,143
Q01844	EWSR1	RNA-binding protein EWS OS=Homo sapiens OX=9606 GN=EWSR1 PE=1 SV=1	6	0,58	1,504	0,614	16755184,26	9984407,931	15554602,08
Q9NTK5	OLA1	Obg-like ATPase 1 OS=Homo sapiens OX=9606 GN=OLA1 PE=1 SV=2	15	0,262	2,155	0,614	34036072,08	7869538,95	13191382,81
Q5RI15	COX20	Cytochrome c oxidase assembly protein COX20, mitochondrial OS=Homo sapiens OX=9606 GN=C	2	11,58	0,052	0,613	121840,2919	1498518,097	2359578,01
O15355	PPM1G	Protein phosphatase 1G OS=Homo sapiens OX=9606 GN=PPM1G PE=1 SV=1	9	0,285	2,153	0,613	7683708,785	2112651,702	3389764,34
Q9P287	BCCIP	BRCA2 and CDKN1A-interacting protein OS=Homo sapiens OX=9606 GN=BCCIP PE=1 SV=1	4	0,28	2,263	0,613	2886231,193	247550,7527	988048,8412
Q14376	GALE	UDP-glucose 4-epimerase OS=Homo sapiens OX=9606 GN=GALE PE=1 SV=2	2	1,23	0,472	0,612	744951,5444	899796,4829	1487573,81
P33991	MCM4	DNA replication licensing factor MCM4 OS=Homo sapiens OX=9606 GN=MCM4 PE=1 SV=5	18	0,48	1,318	0,612	12745705,43	7293980,055	16541528,87
Q96SB4	SRPK1	SRSF protein kinase 1 OS=Homo sapiens OX=9606 GN=SRPK1 PE=1 SV=2	5	0,297	2,775	0,612	2169337,157	747964,0396	1053702,111
P55769	SNU13	NHP2-like protein 1 OS=Homo sapiens OX=9606 GN=SNU13 PE=1 SV=3	3	0,862	0,684	0,61	4309257,202	4357767,036	11923529,87
Q8ND56	LSM14A	Protein LSM14 homolog A OS=Homo sapiens OX=9606 GN=LSM14A PE=1 SV=3	6	0,641	0,88	0,61	3181340,041	1612122,649	3238836,484
Q8NDH3	NPEPL1	Probable aminopeptidase NPEPL1 OS=Homo sapiens OX=9606 GN=NPEPL1 PE=1 SV=3	3	1,478	0,619	0,609	192733,0609	396090,1313	560854,5865
O43290	SART1	U4/U6.U5 tri-snRNP-associated protein 1 OS=Homo sapiens OX=9606 GN=SART1 PE=1 SV=1	17	0,673	0,853	0,609	5926707,959	3373113,758	8528285,695
Q8N5N7	MRPL50	Large ribosomal subunit protein mL50 OS=Homo sapiens OX=9606 GN=MRPL50 PE=1 SV=2	6	1,732	0,435	0,608	2086342,609	3379121,181	6005252,884
Q9P2R3	ANKFY1	Rabankyrin-5 OS=Homo sapiens OX=9606 GN=ANKFY1 PE=1 SV=2	2	1,296	0,473	0,608	152529,4052	198415,9251	322332,9083
P62805	H4C1	Histone H4 OS=Homo sapiens OX=9606 GN=H4C1 PE=1 SV=2	11	0,679	0,89	0,608	2637384665	2190162840	2942064083
Q8N2H4	SYS1	Protein SYS1 homolog OS=Homo sapiens OX=9606 GN=SYS1 PE=1 SV=1	2	1,595	0,311	0,606	268086,6478	448377,921	740893,0222
Q9NX20	MRPL16	Large ribosomal subunit protein uL16m OS=Homo sapiens OX=9606 GN=MRPL16 PE=1 SV=1	4	1,391	0,439	0,606	1955372,277	10963498,31	3322112,203
P35250	RFC2	Replication factor C subunit 2 OS=Homo sapiens OX=9606 GN=RFC2 PE=1 SV=3	7	0,65	1,105	0,606	4295088,301	2593305,013	4057908,29
P61247	RPS3A	Small ribosomal subunit protein eS1 OS=Homo sapiens OX=9606 GN=RPS3A PE=1 SV=2	11	0,609	1,095	0,606	53843875,65	28699762,93	40936064,64
O75150	RNF40	E3 ubiquitin-protein ligase BRE1B OS=Homo sapiens OX=9606 GN=RNF40 PE=1 SV=5	2	0,766	0,792	0,605	371940,5323	276868,4421	457966,2015
Q9UN86	G3BP2	Ras GTPase-activating protein-binding protein 2 OS=Homo sapiens OX=9606 GN=G3BP2 PE=1 SV	6	0,681	0,822	0,605	7328504,903	5496156,355	8175975,079
O75351	VPS4B	Vacuolar protein sorting-associated protein 4B OS=Homo sapiens OX=9606 GN=VPS4B PE=1 SV=	3	0,669	0,798	0,605	2482869,077	1600667,19	3021660,782
P62424	RPL7A	Large ribosomal subunit protein eL8 OS=Homo sapiens OX=9606 GN=RPL7A PE=1 SV=2	13	0,603	1,117	0,605	75894226,99	45941761,33	69192645,18
P35268	RPL22	Large ribosomal subunit protein eL22 OS=Homo sapiens OX=9606 GN=RPL22 PE=1 SV=2	3	0,531	1,221	0,605	42560349,94	22756589,44	39465731,75
P41208	CETN2	Centrin-2 OS=Homo sapiens OX=9606 GN=CETN2 PE=1 SV=1	2	0,338	1,634	0,605	1886469,198	724497,2589	1163151,418
Q9Y679	AUP1	Lipid droplet-regulating VLDL assembly factor AUP1 OS=Homo sapiens OX=9606 GN=AUP1 PE=1	4	1,196	0,675	0,604	1882030,413	2629564,895	4466471,349
Q4VC31	MIX23	Protein MIX23 OS=Homo sapiens OX=9606 GN=MIX23 PE=1 SV=1	4	0,607	0,717	0,604	2526095,468	1447162,892	3109763,328
Q99627	COPS8	COP9 signalosome complex subunit 8 OS=Homo sapiens OX=9606 GN=COPS8 PE=1 SV=1	3	0,558	1,526	0,604	4101940,477	2065772,489	2811975,774
Q8NHH9	ATL2	Atlastin-2 OS=Homo sapiens OX=9606 GN=ATL2 PE=1 SV=2	7	0,654	0,859	0,603	1827126,065	1298672,318	3161293,269
Q9NX14	NDUFB11	NADH dehydrogenase [ubiquinone] 1 beta subcomplex subunit 11, mitochondrial OS=Homo sapi	2	1,69	0,366	0,602	3036588,742	5100464,121	7684458,187
O43395	PRPF3	U4/U6 small nuclear ribonucleoprotein Prp3 OS=Homo sapiens OX=9606 GN=PRPF3 PE=1 SV=2	8	1,057	0,653	0,602	3869023,409	3225497,78	6310291,919
Q9NX00	TMEM160	Transmembrane protein 160 OS=Homo sapiens OX=9606 GN=TMEM160 PE=1 SV=1	2	3,767	0,146	0,601	25738,44591	472169,9697	797081,0396
P99999	CYCS	Cytochrome c OS=Homo sapiens OX=9606 GN=CYCS PE=1 SV=2	7	0,967	0,629	0,601	21273307,72	12371859,38	22036882,64
P53701	HCCS	Holocytochrome c-type synthase OS=Homo sapiens OX=9606 GN=HCCS PE=1 SV=1	4	0,949	0,608	0,601	2953399,465	2762898,538	2528555,226
Q9BY77	POLDIP3	Polymerase delta-interacting protein 3 OS=Homo sapiens OX=9606 GN=POLDIP3 PE=1 SV=2	9	0,591	0,94	0,601	5733305,708	2897303,689	6026336,809
Q9P2K5	MYEF2	Myelin expression factor 2 OS=Homo sapiens OX=9606 GN=MYEF2 PE=1 SV=3	4	0,241	2,639	0,6	5216904,613	2567956,212	2162711,923
P82650	MRPS22	Small ribosomal subunit protein mS22 OS=Homo sapiens OX=9606 GN=MRPS22 PE=1 SV=1	8	1,192	0,469	0,599	3541762,474	2972770,836	7915804,318
Q10713	PMPCA	Mitochondrial-processing peptidase subunit alpha OS=Homo sapiens OX=9606 GN=PMPCA PE=1	12	0,882	0,737	0,599	9482784,147	8369241,884	12254919,29
Q9NQH7	XPNPEP3	Xaa-Pro aminopeptidase 3 OS=Homo sapiens OX=9606 GN=XPNPEP3 PE=1 SV=1	4	0,439	1,396	0,599	1875481,133	266740,9033	1312994,095
Q9BX40	LSM14B	Protein LSM14 homolog B OS=Homo sapiens OX=9606 GN=LSM14B PE=1 SV=1	5	0,405	1,525	0,599	3169530,044	1112225,768	2138345,496
Q8IXM2	BACC1	Chromatin complexes subunit BAP18 OS=Homo sapiens OX=9606 GN=BAP18 PE=1 SV=1	2	0,39	0,726	0,599	1402275,142	467624,9308	1454053,585
Q96EY1	DNAJA3	DnaJ homolog subfamily A member 3, mitochondrial OS=Homo sapiens OX=9606 GN=DNAJA3 PE	3	2,1	0,269	0,598	1293505,237	573038,254	4531419,59
P67809	YBX1	Y-box-binding protein 1 OS=Homo sapiens OX=9606 GN=YBX1 PE=1 SV=3	8	0,629	1,023	0,598	62981450	42829622,46	59890837,59
O43447	PPIH	Peptidyl-prolyl cis-trans isomerase H OS=Homo sapiens OX=9606 GN=PPIH PE=1 SV=1	5	0,705	1,218	0,597	2959388,748	1399177,143	2999868,523
Q969Z0	TBRG4	FAST kinase domain-containing protein 4 OS=Homo sapiens OX=9606 GN=TBRG4 PE=1 SV=1	7	0,574	1,042	0,597	2793799,212	1142379,329	2898985,99
Q8TCC3	MRPL30	Large ribosomal subunit protein uL30m OS=Homo sapiens OX=9606 GN=MRPL30 PE=1 SV=1	2	0,96	0,697	0,596	760234,9941	756740,9718	1089964,409
P06730	EIF4E	Eukaryotic translation initiation factor 4E OS=Homo sapiens OX=9606 GN=EIF4E PE=1 SV=2	2	0,62	1,066	0,596	1653661,361	1000729,624	1416983,272
Q9H6Z4	RANBP3	Ran-binding protein 3 OS=Homo sapiens OX=9606 GN=RANBP3 PE=1 SV=1	2	0,617	1,004	0,596	708236,4012	420299,558	705627,7909
O15371	EIF3D	Eukaryotic translation initiation factor 3 subunit D OS=Homo sapiens OX=9606 GN=EIF3D PE=1 S	10	0,563	1,115	0,596	9611737,566	5082511,422	8759024,351
Q13283	G3BP1	Ras GTPase-activating protein-binding protein 1 OS=Homo sapiens OX=9606 GN=G3BP1 PE=1 SV	15	0,503	1,283	0,596	40210166,53	17060274,29	45392787,87
Q15428	SF3A2	Splicing factor 3A subunit 2 OS=Homo sapiens OX=9606 GN=SF3A2 PE=1 SV=2	3	0,956	0,631	0,595	2326553,089	2380597,835	2698789,575
Q68E01	INTS3	Integrator complex subunit 3 OS=Homo sapiens OX=9606 GN=INTS3 PE=1 SV=1	4	0,7	1,052	0,595	493031,5434	246767,3487	532463,2272
P48729	CSNK1A1	Casein kinase I isoform alpha OS=Homo sapiens OX=9606 GN=CSNK1A1 PE=1 SV=2	4	1,508	0,343	0,594	1231590,224	1471851,443	2849524,779
Q9NP92	MRPS30	Large ribosomal subunit protein mL65 OS=Homo sapiens OX=9606 GN=MRPS30 PE=1 SV=2	2	1,296	0,474	0,594	1090340,201	1381886,465	2384274,78
Q4G0J3	LARP7	La-related protein 7 OS=Homo sapiens OX=9606 GN=LARP7 PE=1 SV=1	2	0,799	0,904	0,594	456442,5692	372241,7701	626430,3937
O75821	EIF3G	Eukaryotic translation initiation factor 3 subunit G OS=Homo sapiens OX=9606 GN=EIF3G PE=1 S	6	0,552	1,206	0,594	9720693,64	4458753,553	8201146,411
Q53GS9	USP39	Ubiquitin carboxyl-terminal hydrolase 39 OS=Homo sapiens OX=9606 GN=USP39 PE=1 SV=2	9	0,45	1,227	0,594	5122879,762	1980303,745	4520238,492
P62750	RPL23A	Large ribosomal subunit protein uL23 OS=Homo sapiens OX=9606 GN=RPL23A PE=1 SV=1	8	0,357	1,691	0,594	28750792,27	10184205,49	21149969,77
O75323	NIPSNAP2	Protein NipSnap homolog 2 OS=Homo sapiens OX=9606 GN=NIPSNAP2 PE=1 SV=1	3	1,734	0,312	0,592	2087079,33	3321868,485	6217262,51
Q49A26	GLYR1	Cytokine-like nuclear factor N-PAC OS=Homo sapiens OX=9606 GN=GLYR1 PE=1 SV=4	3	0,788	0,751	0,592	1249343,395	942114,9955	1632822,379
P49792	RANBP2	E3 SUMO-protein ligase RanBP2 OS=Homo sapiens OX=9606 GN=RANBP2 PE=1 SV=2	46	0,721	0,79	0,592	28886239,14	19410335,72	35267663,3
P33993	MCM7	DNA replication licensing factor MCM7 OS=Homo sapiens OX=9606 GN=MCM7 PE=1 SV=4	19	0,533	1,195	0,592	24056324,59	8255749,074	20464418,42

P62249	RPS16	Small ribosomal subunit protein uS9 OS=Homo sapiens OX=9606 GN=RPS16 PE=1 SV=2	9	0,513	1,273	0,592	55764284,36	26105587,88	42737727,37
Q9NQT5	EXOSC3	Exosome complex component RRP40 OS=Homo sapiens OX=9606 GN=EXOSC3 PE=1 SV=3	2	1,028	0,567	0,591	690407,7077	719228,1873	1217833,941
Q9H0S4	DDX47	Probable ATP-dependent RNA helicase DDX47 OS=Homo sapiens OX=9606 GN=DDX47 PE=1 SV=	6	0,5	1,029	0,591	2840985,72	1466441,074	2733361,148
Q9GZL7	WDR12	Ribosome biogenesis protein WDR12 OS=Homo sapiens OX=9606 GN=WDR12 PE=1 SV=2	7	0,484	1,203	0,591	5362669,731	2621299,623	3644557,153
P25325	MPST	3-mercaptopyruvate sulfurtransferase OS=Homo sapiens OX=9606 GN=MPST PE=1 SV=3	7	0,624	0,984	0,59	4648429,024	8284884,712	5924776,918
Q99729	HNRNPAB	Heterogeneous nuclear ribonucleoprotein A/B OS=Homo sapiens OX=9606 GN=HNRNPAB PE=1	8	0,576	1,183	0,59	34874132,14	18013038,28	34318593,99
Q8WVJ2	NUDCD2	NudC domain-containing protein 2 OS=Homo sapiens OX=9606 GN=NUDCD2 PE=1 SV=1	2	0,26	2,347	0,59	1894250,358	473575,6176	814932,3802
Q6UW68	TMEM205	Transmembrane protein 205 OS=Homo sapiens OX=9606 GN=TMEM205 PE=1 SV=1	2	5,278	0,109	0,589	2235030,733	12216880,48	21118167,72
Q5VTR2	RNF20	E3 ubiquitin-protein ligase BRE1A OS=Homo sapiens OX=9606 GN=RNF20 PE=1 SV=2	4	0,835	0,73	0,589	1445898,861	1067432,945	2106131,238
Q12996	CSTF3	Cleavage stimulation factor subunit 3 OS=Homo sapiens OX=9606 GN=CSTF3 PE=1 SV=1	12	0,833	0,689	0,589	3905711,763	3332810,504	6887833,391
P46781	RPS9	Small ribosomal subunit protein uS4 OS=Homo sapiens OX=9606 GN=RPS9 PE=1 SV=3	12	0,452	1,236	0,589	53768914,97	24724544,53	42072727,96
Q8TDN6	BRIX1	Ribosome biogenesis protein BRX1 homolog OS=Homo sapiens OX=9606 GN=BRIX1 PE=1 SV=2	5	0,413	0,799	0,588	1628830,09	836706,2905	1177256,298
P30626	SRI	Sorcin OS=Homo sapiens OX=9606 GN=SRI PE=1 SV=1	3	0,37	1,685	0,588	6808301,974	4730965,918	4369709,802
P49916	LIG3	DNA ligase 3 OS=Homo sapiens OX=9606 GN=LIG3 PE=1 SV=2	10	0,239	2,131	0,588	4532792,642	799359,1418	2104067,81
Q96KR1	ZFR	Zinc finger RNA-binding protein OS=Homo sapiens OX=9606 GN=ZFR PE=1 SV=2	9	0,637	1,033	0,587	6505281,776	4109964,782	6663812,311
P53367	ARFIP1	Arfaptin-1 OS=Homo sapiens OX=9606 GN=ARFIP1 PE=1 SV=2	3	1,548	0,394	0,586	1038035,447	1658540,685	2985622,094
O15269	SPTLC1	Serine palmitoyltransferase 1 OS=Homo sapiens OX=9606 GN=SPTLC1 PE=1 SV=1	4	1,537	0,301	0,586	1792051,98	3482257,458	5703081,36
Q14152	EIF3A	Eukaryotic translation initiation factor 3 subunit A OS=Homo sapiens OX=9606 GN=EIF3A PE=1 S	35	0,403	1,428	0,586	37866681,77	14534201,59	23780783,55
Q99797	MIPEP	Mitochondrial intermediate peptidase OS=Homo sapiens OX=9606 GN=MIPEP PE=1 SV=2	4	1,096	0,526	0,585	1155447,264	1251955,799	2269609,087
P53597	SUCLG1	Succinate--CoA ligase [ADP/GDP-forming] subunit alpha, mitochondrial OS=Homo sapiens OX=9	5	0,78	0,691	0,585	5021428,32	5320810,558	7388256,156
Q92925	SMARCD2	SWI/SNF-related matrix-associated actin-dependent regulator of chromatin subfamily D membe	4	0,762	1,54	0,585	2240728,705	1088106,309	1845633,548
P0DP23	CALM1	Calmodulin-1 OS=Homo sapiens OX=9606 GN=CALM1 PE=1 SV=1	9	0,655	0,78	0,585	69794350,73	58987596,51	91962769,1
Q9NRY4	ARHGAP35	Rho GTPase-activating protein 35 OS=Homo sapiens OX=9606 GN=ARHGAP35 PE=1 SV=3	4			0,584	195448,722	35031,2494	59960,49524
O15511	ARPC5	Actin-related protein 2/3 complex subunit 5 OS=Homo sapiens OX=9606 GN=ARPC5 PE=1 SV=3	4	1,728	0,532	0,584	658611,7556	1537330,06	2498848,46
Q8WU76	SCFD2	Sec1 family domain-containing protein 2 OS=Homo sapiens OX=9606 GN=SCFD2 PE=1 SV=2	4	0,766	0,581	0,584	258326,459	193306,1471	479719,4951
Q8NG11	TSPAN14	Tetraspanin-14 OS=Homo sapiens OX=9606 GN=TSPAN14 PE=1 SV=1	2	1,743	0,326	0,583	192130,4846	334815,3356	554636,8006
P00966	ASS1	Argininosuccinate synthase OS=Homo sapiens OX=9606 GN=ASS1 PE=1 SV=2	10	7,003	0,09	0,582	1117893,175	9156293,399	17896902,66
Q7L0Y3	TRMT10C	tRNA methyltransferase 10 homolog C OS=Homo sapiens OX=9606 GN=TRMT10C PE=1 SV=2	11	0,903	0,69	0,582	6141135,797	4503082,849	9377947,516
Q9UHB9	SRP68	Signal recognition particle subunit SRP68 OS=Homo sapiens OX=9606 GN=SRP68 PE=1 SV=2	10	0,87	0,832	0,582	5854476,374	4041399,98	7230766,509
Q14694	USP10	Ubiquitin carboxyl-terminal hydrolase 10 OS=Homo sapiens OX=9606 GN=USP10 PE=1 SV=2	9	0,704	0,8	0,582	5952774,002	3806608,93	7515549,09
Q9Y6I9	TEX264	Testis-expressed protein 264 OS=Homo sapiens OX=9606 GN=TEX264 PE=1 SV=1	3	2,726	0,342	0,581	331817,555	644215,1277	2236269,407
Q96HY6	DDRGK1	DDRGK domain-containing protein 1 OS=Homo sapiens OX=9606 GN=DDRGK1 PE=1 SV=2	7	1,171	0,468	0,581	2624495,437	4220753,358	7154842,141
Q96KB5	PBK	Lymphokine-activated killer T-cell-originated protein kinase OS=Homo sapiens OX=9606 GN=PBK	2	0,514	1,152	0,581	1152746,68	595826,9938	998298,5474
P67870	CSNK2B	Casein kinase II subunit beta OS=Homo sapiens OX=9606 GN=CSNK2B PE=1 SV=1	2	0,318	1,577	0,581	5152920,037	2091804,964	3260639,247
Q9Y383	LUC7L2	Putative RNA-binding protein Luc7-like 2 OS=Homo sapiens OX=9606 GN=LUC7L2 PE=1 SV=2	7	0,238	2,054	0,581	10894061,94	2960743,901	6319392,042
Q9UBB5	MBD2	Methyl-CpG-binding domain protein 2 OS=Homo sapiens OX=9606 GN=MBD2 PE=1 SV=1	2	4,472	0,129	0,58	122689,7999	548634,4059	1648836,616
Q96J02	ITCH	E3 ubiquitin-protein ligase Itchy homolog OS=Homo sapiens OX=9606 GN=ITCH PE=1 SV=2	4	1,496	0,461	0,579	539198,0308	758534,2962	1129083,436
Q8N4H5	TOMM5	Mitochondrial import receptor subunit TOM5 homolog OS=Homo sapiens OX=9606 GN=TOMM5	4	1,327	0,646	0,579	3234697,981	4405816,943	7000349,594
P35270	SPR	Sepiapterin reductase OS=Homo sapiens OX=9606 GN=SPR PE=1 SV=1	8	1	0,13	0,579	61171,40543	889339,2645	2064771,43
Q99543	DNAJC2	DnaJ homolog subfamily C member 2 OS=Homo sapiens OX=9606 GN=DNAJC2 PE=1 SV=4	2	0,593	0,95	0,579	459456,0244	269984,8726	523016,6705
Q96PK6	RBM14	RNA-binding protein 14 OS=Homo sapiens OX=9606 GN=RBM14 PE=1 SV=2	16	0,751	0,829	0,578	30167609,12	22212475,61	32465929,9
P35249	RFC4	Replication factor C subunit 4 OS=Homo sapiens OX=9606 GN=RFC4 PE=1 SV=2	8	0,63	0,941	0,578	8875825,521	4334034,352	9645043,374
P06454	PTMA	Prothymosin alpha OS=Homo sapiens OX=9606 GN=PTMA PE=1 SV=2	3	1000	0,001	0,577		951517,3085	1650427,456
P55884	EIF3B	Eukaryotic translation initiation factor 3 subunit B OS=Homo sapiens OX=9606 GN=EIF3B PE=1 S	15	0,501	1,127	0,577	23917634,73	12085692,18	21072230,27
Q92922	SMARCC1	SWI/SNF complex subunit SMARCC1 OS=Homo sapiens OX=9606 GN=SMARCC1 PE=1 SV=3	8	0,204	2,256	0,576	13438956,24	2781247,052	4443434,884
P18859	ATP5PF	ATP synthase-coupling factor 6, mitochondrial OS=Homo sapiens OX=9606 GN=ATP5PF PE=1 SV=	5	1,433	0,41	0,575	6298331,432	8632258,36	12669860,8
Q9Y6X3	MAU2	MAU2 chromatid cohesion factor homolog OS=Homo sapiens OX=9606 GN=MAU2 PE=1 SV=2	2	0,752	0,898	0,575	420727,1703	288206,0393	503191,6173
Q16543	CDC37	Hsp90 co-chaperone Cdc37 OS=Homo sapiens OX=9606 GN=CDC37 PE=1 SV=1	6	0,916	0,626	0,574	11085626,7	9901040,707	17381921,3
P46060	RANGAP1	Ran GTPase-activating protein 1 OS=Homo sapiens OX=9606 GN=RANGAP1 PE=1 SV=1	15	0,75	0,832	0,574	16928434,05	8770837,622	19910366,02
Q9BRX5	GINS3	DNA replication complex GINS protein PSF3 OS=Homo sapiens OX=9606 GN=GINS3 PE=1 SV=1	3	0,368	1,559	0,574	918025,2619	311728,8266	615117,2417
P68366	TUBA4A	Tubulin alpha-4A chain OS=Homo sapiens OX=9606 GN=TUBA4A PE=1 SV=1	3	7,712	0,074	0,573	31173176,76	20231541,73	22094492,23
Q6WCQ1	MPRIP	Myosin phosphatase Rho-interacting protein OS=Homo sapiens OX=9606 GN=MPRIP PE=1 SV=3	15	3,165	0,188	0,573	2037646,271	5417089,056	10684071,26
O43837	IDH3B	Isocitrate dehydrogenase [NAD] subunit beta, mitochondrial OS=Homo sapiens OX=9606 GN=IDI	13	2,026	0,315	0,573	4345113,574	7149805,457	12578957,37
O60763	USO1	General vesicular transport factor p115 OS=Homo sapiens OX=9606 GN=USO1 PE=1 SV=2	13	1,12	0,527	0,573	6285268,503	6667995,531	12003800,35
P82914	MRPS15	Small ribosomal subunit protein uS15m OS=Homo sapiens OX=9606 GN=MRPS15 PE=1 SV=1	3	1,064	0,448	0,573	1096619,823	1446119,779	2279886,157
Q08AF3	SLFN5	Schlafen family member 5 OS=Homo sapiens OX=9606 GN=SLFN5 PE=1 SV=1	2	1,042	0,561	0,572	844428,494	905730,6754	1504295,172
P50897	PPT1	Palmitoyl-protein thioesterase 1 OS=Homo sapiens OX=9606 GN=PPT1 PE=1 SV=1	5	0,769	0,795	0,572	5793676,737	3506257,401	6184952,839
P46087	NOP2	Probable 28S rRNA (cytosine(4447)-C(5))-methyltransferase OS=Homo sapiens OX=9606 GN=NO	11	0,581	1,135	0,572	6167408,179	3315965,141	7002005,219
Q13257	MAD2L1	Mitotic spindle assembly checkpoint protein MAD2A OS=Homo sapiens OX=9606 GN=MAD2L1 P	3	0,368	1,727	0,572	1672348,14	513176,7706	788877,4765
Q7Z3D6	DGLUCY	D-glutamate cyclase, mitochondrial OS=Homo sapiens OX=9606 GN=DGLUCY PE=1 SV=2	4	1,595	0,288	0,57	165711,7422	117092,1926	587246,6538
P82921	MRPS21	Small ribosomal subunit protein bS21m OS=Homo sapiens OX=9606 GN=MRPS21 PE=1 SV=3	2	1,412	0,629	0,57	358424,6487	683887,3494	1106667,594
O60437	PPL	Periplakin OS=Homo sapiens OX=9606 GN=PPL PE=1 SV=4	13	0,938	0,279	0,57	114837,4941	4458346,811	8009166,011
P48730	CSNK1D	Casein kinase I isoform delta OS=Homo sapiens OX=9606 GN=CSNK1D PE=1 SV=2	3	0,811	0,748	0,57	805162,3691	618579,9948	1092331,646
O75475	PSIP1	PC4 and SFRS1-interacting protein OS=Homo sapiens OX=9606 GN=PSIP1 PE=1 SV=1	11	0,587	0,983	0,569	20422614,32	11542187,42	19180306,23
Q00325	SLC25A3	Solute carrier family 25 member 3 OS=Homo sapiens OX=9606 GN=SLC25A3 PE=1 SV=2	12	0,982	0,722	0,568	50952248,92	46111188,56	85955315,12
P10606	COX5B	Cytochrome c oxidase subunit 5B, mitochondrial OS=Homo sapiens OX=9606 GN=COX5B PE=1 S\	7	1,109	0,552	0,567	19240297,72	22206727,18	34153674,44
P78406	RAE1	mRNA export factor RAE1 OS=Homo sapiens OX=9606 GN=RAE1 PE=1 SV=1	5	0,528	1,263	0,567	8839728,305	4171883,384	7410029,816
P31689	DNAJA1	DnaJ homolog subfamily A member 1 OS=Homo sapiens OX=9606 GN=DNAJA1 PE=1 SV=2	10	0,504	0,999	0,567	10618040,28	4923458,948	11426010,53
P28340	POLD1	DNA polymerase delta catalytic subunit OS=Homo sapiens OX=9606 GN=POLD1 PE=1 SV=2	2	1,911	0,382	0,566	377820,4323	592457,5117	1098226,146
P43003	SLC1A3	Excitatory amino acid transporter 1 OS=Homo sapiens OX=9606 GN=SLC1A3 PE=1 SV=1	4	1,043	0,705	0,566	959442,7173	785429,8045	1193330,142
Q9H490	PIGU	Phosphatidylinositol glycan anchor biosynthesis class U protein OS=Homo sapiens OX=9606 GN=	4	0,977	0,593	0,566	2231417,385	2116685,435	3808230,425
P12931	SRC	Proto-oncogene tyrosine-protein kinase Src OS=Homo sapiens OX=9606 GN=SRC PE=1 SV=3	7	0,563	1,239	0,566	4044386,962	2905042,509	3668359,534
Q9NX40	OCIAD1	OCIA domain-containing protein 1 OS=Homo sapiens OX=9606 GN=OCIAD1 PE=1 SV=1	7	1,555	0,356	0,565	4775010,529	7359820,04	11461035,35
P08708	RPS17	Small ribosomal subunit protein eS17 OS=Homo sapiens OX=9606 GN=RPS17 PE=1 SV=2	6	0,454	1,767	0,565	19044969,51	4787973,399	8198057,41
Q8WUK0	PTPMT1	Phosphatidylglycerophosphatase and protein-tyrosine phosphatase 1 OS=Homo sapiens OX=960	2	1,406	0,419	0,564	161902,4132	193154,0878	386273,1102
Q8TED0	UTP15	U3 small nucleolar RNA-associated protein 15 homolog OS=Homo sapiens OX=9606 GN=UTP15 F	2	0,713	0,671	0,564	579211,1371	177866,5374	740354,3947
Q5BKZ1	ZNF326	DBIRD complex subunit ZNF326 OS=Homo sapiens OX=9606 GN=ZNF326 PE=1 SV=2	7	0,614	0,955	0,564	6599241,298	4109484,142	7073954,329
Q9BZF9	UACA	Uveal autoantigen with coiled-coil domains and ankyrin repeats OS=Homo sapiens OX=9606 GN=	2	2,549	0,233	0,563	85833,83676	176947,4424	324901,8624
Q96AA3	RFT1	Protein RFT1 homolog OS=Homo sapiens OX=9606 GN=RFT1 PE=1 SV=1	3	1,356	0,477	0,563	754889,0901	1005216,902	1501492,398
Q8N4Q1	CHCHD4	Mitochondrial intermembrane space import and assembly protein 40 OS=Homo sapiens OX=960	2	1,082	0,589	0,563	637190,6031	906524,8154	1975359,911
P23786	CPT2	Carnitine O-palmitoyltransferase 2, mitochondrial OS=Homo sapiens OX=9606 GN=CPT2 PE=1 SV	13	1,106	0,423	0,562	5801399,787	5568022,925	10937313,46
Q969X5	ERGIC1	Endoplasmic reticulum-Golgi intermediate compartment protein 1 OS=Homo sapiens OX=9606 G	7	0,52	1,137	0,561	9535756,712	4016708,721	8755565,582
Q96FF9	CDCA5	Sororin OS=Homo sapiens OX=9606 GN=CDCA5 PE=1 SV=1	2	0,969	0,591	0,56	763286,5419	733930,0405	1491987,837
Q96A08	H2BC1	Histone H2B type 1-A OS=Homo sapiens OX=9606 GN=H2BC1 PE=1 SV=3	2	0,74	0,579	0,56	730929668,6	542960640,6	732987637
O60231	DHX16	Pre-mRNA-splicing factor ATP-dependent RNA helicase DHX16 OS=Homo sapiens OX=9606 GN=[	3	0,203	1,543	0,56	1103514,942	50283,14934	723823,1718
Q6YP21	KYAT3	Kynurenine--oxoglutarate transaminase 3 OS=Homo sapiens OX=9606 GN=KYAT3 PE=1 SV=1	5	0,764	0,731	0,559	1197236,011	398093,4657	1894786,402
Q8TC12	RDH11	Retinol dehydrogenase 11 OS=Homo sapiens OX=9606 GN=RDH11 PE=1 SV=2	7	0,664	1,125	0,559	8228983,903	6501088,877	10182744,13
P30511	HLA-F	HLA class I histocompatibility antigen, alpha chain F OS=Homo sapiens OX=9606 GN=HLA-F PE=1	7	1,514	0,348	0,557	1063246,256	1721625,544	6561777,511
Q29RF7	PDS5A	Sister chromatid cohesion protein PDS5 homolog A OS=Homo sapiens OX=9606 GN=PDS5A PE=1	14	0,817	0,692	0,556	6720685,068	5547605,006	10681355,95
Q9HBH5	RDH14	Retinol dehydrogenase 14 OS=Homo sapiens OX=9606 GN=RDH14 PE=1 SV=1	3	2,442	0,251	0,555	289855,1239	878268,9204	1609002,106
Q9Y666	SLC12A7	Solute carrier family 12 member 7 OS=Homo sapiens OX=9606 GN=SLC12A7 PE=1 SV=3	8	0,344	1,527	0,555	1706160,538	463977,6788	1317690,396
Q99829	CPNE1	Copine-1 OS=Homo sapiens OX=9606 GN=CPNE1 PE=1 SV=1	5	0,493	1,115	0,554	7448861,296	3562216,861	6489636,536
Q12965	MYO1E	Unconventional myosin-Ie OS=Homo sapiens OX=9606 GN=MYO1E PE=1 SV=2	4	3,333	0,156	0,553	117177,0911	352219,2858	1405263,976
Q9H845	ACAD9	Complex I assembly factor ACAD9, mitochondrial OS=Homo sapiens OX=9606 GN=ACAD9 PE=1 S	12	1,381	0,423	0,553	6506552,449	8647646,427	13850506,57
Q5SRE5	NUP188	Nucleoporin NUP188 OS=Homo sapiens OX=9606 GN=NUP188 PE=1 SV=1	6	1,065	0,417	0,553	1780015,066	1636163,874	3437321,351
P84098	RPL19	Large ribosomal subunit protein eL19 OS=Homo sapiens OX=9606 GN=RPL19 PE=1 SV=1	3	0,96	0,585	0,553	7828456,401	7609969,128	13592502,72
P56182	RRP1	Ribosomal RNA processing protein 1 homolog A OS=Homo sapiens OX=9606 GN=RRP1 PE=1 SV=	2	0,746	0,722	0,553	570445,246	254111,5861	794828,7607
O94919	ENDOD1	Endonuclease domain-containing 1 protein OS=Homo sapiens OX=9606 GN=ENDOD1 PE=1 SV=2	3	0,707	0,669	0,553	595560,7222	661898,5951	1279508,543
Q92785	DPF2	Zinc finger protein ubi-d4 OS=Homo sapiens OX=9606 GN=DPF2 PE=1 SV=2	4	0,24	2,42	0,553	2794945,084	426696,6598	1159042,076
O94888	UBXN7	UBX domain-containing protein 7 OS=Homo sapiens OX=9606 GN=UBXN7 PE=1 SV=2	3	0,356	1,455	0,552	1028196,071	357407,1127	682963,2261
Q86Y56	DNAAF5	Dynein axonemal assembly factor 5 OS=Homo sapiens OX=9606 GN=DNAAF5 PE=1 SV=4	3	0,378	1,578	0,551	1240535,893	460021,8141	795526,3998
Q92597	NDRG1	Protein NDRG1 OS=Homo sapiens OX=9606 GN=NDRG1 PE=1 SV=1	7	0,213	1,668	0,551	306897,7714	365758,0126	810651,4401
P40429	RPL13A	Large ribosomal subunit protein uL13 OS=Homo sapiens OX=9606 GN=RPL13A PE=1 SV=2	8	0,4	0,937	0,55	33801683,25	16359750,47	29437823,97
Q8N163	CCAR2	Cell cycle and apoptosis regulator protein 2 OS=Homo sapiens OX=9606 GN=CCAR2 PE=1 SV=2	19	0,964	0,65	0,549	20050060,85	13914334,02	28856094,45
P30154	PPP2R1B	Serine/threonine-protein phosphatase 2A 65 kDa regulatory subunit A beta isoform OS=Homo sa	2	0,418	1,482	0,549	1361126,834	469342,2653	865385,0465
Q99417	MYCBP	c-Myc-binding protein OS=Homo sapiens OX=9606 GN=MYCBP PE=1 SV=3	2	0,899	0,644	0,548	1735822,773	1623941,931	2634829,421
P40938	RFC3	Replication factor C subunit 3 OS=Homo sapiens OX=9606 GN=RFC3 PE=1 SV=2	3	0,817	0,713	0,548	1397363,017	1147640,549	1946510,74
Q92665	MRPS31	Small ribosomal subunit protein mS31 OS=Homo sapiens OX=9606 GN=MRPS31 PE=1 SV=3	6	0,828	0,684	0,547	2653451,758	2223535,858	4079471,996
O15091	PRORP	Mitochondrial ribonuclease P catalytic subunit OS=Homo sapiens OX=9606 GN=PRORP PE=1 SV=	2	0,54	1,213	0,547	412834,8432	211486,9633	379745,1735
P78347	GTF2I	General transcription factor II-I OS=Homo sapiens OX=9606 GN=GTF2I PE=1 SV=2	35	0,539	1,209	0,547	56499448,15	20491363,88	45944480,54
Q8WXF1	PSPC1	Paraspeckle component 1 OS=Homo sapiens OX=9606 GN=PSPC1 PE=1 SV=1	11	0,547	1,105	0,546	12122804,32	6645406,989	12361105,07

P17096	HMGA1	High mobility group protein HMG-I/HMG-Y OS=Homo sapiens OX=9606 GN=HMGA1 PE=1 SV=3	4	0,16	0,308	0,546	20028058,54	2976582,968	7930252,133
Q9ULV4	CORO1C	Coronin-1C OS=Homo sapiens OX=9606 GN=CORO1C PE=1 SV=1	8	1,224	0,577	0,544	4707529,047	4658030,927	8192319,05
Q9NP79	VTA1	Vacuolar protein sorting-associated protein VTA1 homolog OS=Homo sapiens OX=9606 GN=VTA	2	0,813	0,681	0,544	1054994,162	858071,5654	1576421,022
Q8WXH0	SYNE2	Nesprin-2 OS=Homo sapiens OX=9606 GN=SYNE2 PE=1 SV=3	28	0,687	0,693	0,544	5280727,651	4111524,813	9221752,294
P98175	RBM10	RNA-binding protein 10 OS=Homo sapiens OX=9606 GN=RBM10 PE=1 SV=3	7	0,618	0,922	0,543	3305841,758	1127517,214	5215438,449
Q96J01	THOC3	THO complex subunit 3 OS=Homo sapiens OX=9606 GN=THOC3 PE=1 SV=1	3	0,501	1,137	0,543	1878875,779	632600,7529	1598792,375
O60716	CTNND1	Catenin delta-1 OS=Homo sapiens OX=9606 GN=CTNND1 PE=1 SV=1	29	2,703	0,19	0,542	12588413,16	35954459	77629424,53
Q9NWU5	MRPL22	Large ribosomal subunit protein uL22m OS=Homo sapiens OX=9606 GN=MRPL22 PE=1 SV=1	5	1,297	0,362	0,542	2259256,43	2145556,99	6062425,474
Q9NPD3	EXOSC4	Exosome complex component RRP41 OS=Homo sapiens OX=9606 GN=EXOSC4 PE=1 SV=3	3	0,691	0,852	0,542	2691042,472	1605371,419	3041340,59
Q13610	PWP1	Periodic tryptophan protein 1 homolog OS=Homo sapiens OX=9606 GN=PWP1 PE=1 SV=1	4	0,752	0,757	0,541	1463758,424	1291853,131	2036591,382
Q9ULX6	AKAP8L	A-kinase anchor protein 8-like OS=Homo sapiens OX=9606 GN=AKAP8L PE=1 SV=4	3	0,7	0,808	0,54	388893,3332	242039,7814	448021,7154
Q7KZ85	SUPT6H	Transcription elongation factor SPT6 OS=Homo sapiens OX=9606 GN=SUPT6H PE=1 SV=2	9	0,469	1,461	0,54	1715605,894	739660,0366	2465294,278
Q14566	MCM6	DNA replication licensing factor MCM6 OS=Homo sapiens OX=9606 GN=MCM6 PE=1 SV=1	18	0,514	1,14	0,539	58907396,64	44932558,89	26792823,13
Q14C86	GAPVD1	GTPase-activating protein and VPS9 domain-containing protein 1 OS=Homo sapiens OX=9606 GN	2	1,042	0,555	0,538	491074,1159	460216,3646	865562,9188
Q13185	CBX3	Chromobox protein homolog 3 OS=Homo sapiens OX=9606 GN=CBX3 PE=1 SV=4	4	0,674	0,793	0,538	23317607,22	15325640,36	26724484,08
Q08170	SRSF4	Serine/arginine-rich splicing factor 4 OS=Homo sapiens OX=9606 GN=SRSF4 PE=1 SV=2	3	0,756	0,705	0,537	3696896,307	2870755,084	6512707,426
Q9BYN8	MRPS26	Small ribosomal subunit protein mS26 OS=Homo sapiens OX=9606 GN=MRPS26 PE=1 SV=1	5	1,653	0,377	0,536	1844596,215	2330841,565	5279534,466
O43347	MSI1	RNA-binding protein Musashi homolog 1 OS=Homo sapiens OX=9606 GN=MSI1 PE=1 SV=1	8	0,35	7,442	0,536	13356251,58	491381,6792	1389144,576
O43813	LANCL1	Glutathione S-transferase LANCL1 OS=Homo sapiens OX=9606 GN=LANCL1 PE=1 SV=1	2	0,481	1,126	0,535	1106068,104	536719,086	1004018,095
O15400	STX7	Syntaxin-7 OS=Homo sapiens OX=9606 GN=STX7 PE=1 SV=4	7	1,136	0,373	0,534	1995925,767	4202645,43	6071163,472
Q9NS69	TOMM22	Mitochondrial import receptor subunit TOM22 homolog OS=Homo sapiens OX=9606 GN=TOMM	6	0,987	0,624	0,534	7653101,785	7036772,974	11620744,2
Q96EY7	PTCD3	Small ribosomal subunit protein mS39 OS=Homo sapiens OX=9606 GN=PTCD3 PE=1 SV=3	10	0,846	0,605	0,534	4474764,581	1635992,413	5950928,691
Q9Y5Q9	GTF3C3	General transcription factor 3C polypeptide 3 OS=Homo sapiens OX=9606 GN=GTF3C3 PE=1 SV=	4	0,464	1	0,534	11978760,7	3020804,702	1350083,642
Q9BPW8	NIPSNAP1	Protein NipSnap homolog 1 OS=Homo sapiens OX=9606 GN=NIPSNAP1 PE=1 SV=1	4	0,46	1,161	0,534	6041287,546	2538531,098	5143081,362
Q9UKF6	CPSF3	Cleavage and polyadenylation specificity factor subunit 3 OS=Homo sapiens OX=9606 GN=CPSF3	7	0,628	0,899	0,533	2826655,293	1602777,18	2978411,33
Q9UL15	BAG5	BAG family molecular chaperone regulator 5 OS=Homo sapiens OX=9606 GN=BAG5 PE=1 SV=1	4	0,765	1,18	0,532	1038457,13	586704,3817	839476,4829
Q96SZ6	CDK5RAP1	Mitochondrial tRNA methylthiotransferase CDK5RAP1 OS=Homo sapiens OX=9606 GN=CDK5RAP	2	0,765	0,663	0,532	444015,6478	352980,9925	666239,6127
O00422	SAP18	Histone deacetylase complex subunit SAP18 OS=Homo sapiens OX=9606 GN=SAP18 PE=1 SV=1	3	0,456	0,746	0,532	961828,6749	830759,4171	1432120,53
P52701	MSH6	DNA mismatch repair protein Msh6 OS=Homo sapiens OX=9606 GN=MSH6 PE=1 SV=2	9	0,265	2,247	0,532	2617691,197	323413,4143	1128516,519
Q13405	MRPL49	Large ribosomal subunit protein mL49 OS=Homo sapiens OX=9606 GN=MRPL49 PE=1 SV=1	4	0,879	0,489	0,531	3980823,305	3451705,259	7728816,369
Q8WVM7	STAG1	Cohesin subunit SA-1 OS=Homo sapiens OX=9606 GN=STAG1 PE=1 SV=3	2	0,857	0,612	0,531	279302,8291	239422,9621	450704,5721
P41223	BUD31	Protein BUD31 homolog OS=Homo sapiens OX=9606 GN=BUD31 PE=1 SV=2	4	0,727	0,739	0,531	1113181,598	453858,0214	1465133,738
O76094	SRP72	Signal recognition particle subunit SRP72 OS=Homo sapiens OX=9606 GN=SRP72 PE=1 SV=3	7	0,549	1,033	0,531	3341425,679	1365153,212	2532147,146
Q9NW13	RBM28	RNA-binding protein 28 OS=Homo sapiens OX=9606 GN=RBM28 PE=1 SV=3	6	0,649	0,654	0,53	1114683,913	1054225,182	2080168,66
Q13619	CUL4A	Cullin-4A OS=Homo sapiens OX=9606 GN=CUL4A PE=1 SV=3	3	0,474	1,079	0,53	758612,8972	341017,0579	630911,1054
Q8WXA9	SREK1	Splicing regulatory glutamine/lysine-rich protein 1 OS=Homo sapiens OX=9606 GN=SREK1 PE=1 S	3	0,421	1,124	0,53	800324,7834	260902,0192	861856,9336
Q01650	SLC7A5	Large neutral amino acids transporter small subunit 1 OS=Homo sapiens OX=9606 GN=SLC7A5 PI	6	5,685	0,104	0,529	6666074,32	38162567,79	63248594,93
Q8TBA6	GOLGA5	Golgin subfamily A member 5 OS=Homo sapiens OX=9606 GN=GOLGA5 PE=1 SV=3	5	1,836	0,37	0,529	417372,7224	1057682,574	1870107,935
Q9BQS8	FYCO1	FYVE and coiled-coil domain-containing protein 1 OS=Homo sapiens OX=9606 GN=FYCO1 PE=1 S	6	1,33	0,382	0,529	316299,2589	416418,9537	682430,2953
P49748	ACADVL	Very long-chain specific acyl-CoA dehydrogenase, mitochondrial OS=Homo sapiens OX=9606 GN	23	2,4	0,214	0,528	13535169,86	30615994,63	72225241
Q96HV5	TMEM41A	Transmembrane protein 41A OS=Homo sapiens OX=9606 GN=TMEM41A PE=1 SV=1	2	1,222	0,447	0,528	498986,7073	555005,6857	1094396,374
Q02978	SLC25A11	Mitochondrial 2-oxoglutarate/malate carrier protein OS=Homo sapiens OX=9606 GN=SLC25A11 I	11	1,004	0,585	0,528	5312028,122	4501169,838	8960795,965
Q9BTC8	MTA3	Metastasis-associated protein MTA3 OS=Homo sapiens OX=9606 GN=MTA3 PE=1 SV=2	3	0,799	0,778	0,528	1619995,897	1741091,103	3173560,794
P45954	ACADSB	Short/branched chain specific acyl-CoA dehydrogenase, mitochondrial OS=Homo sapiens OX=96	8	0,684	0,626	0,528	906745,6025	757436,5877	2199150,238
Q05519	SRSF11	Serine/arginine-rich splicing factor 11 OS=Homo sapiens OX=9606 GN=SRSF11 PE=1 SV=1	5	0,465	1,228	0,528	5431965,58	2669500,428	4976016,608
Q6DKI1	RPL7L1	Ribosomal protein uL30-like OS=Homo sapiens OX=9606 GN=RPL7L1 PE=1 SV=2	3	0,452	0,542	0,528	631300,3653	722653,4105	1379386,842
O96011	PEX11B	Peroxisomal membrane protein 11B OS=Homo sapiens OX=9606 GN=PEX11B PE=1 SV=1	2	3,55	0,197	0,527	93352,39617	1232816,359	2377972,325
Q92882	OSTF1	Osteoclast-stimulating factor 1 OS=Homo sapiens OX=9606 GN=OSTF1 PE=1 SV=2	5	2,803	0,21	0,527	101570,7741	835686,4253	1830104,447
Q96A35	MRPL24	Large ribosomal subunit protein uL24m OS=Homo sapiens OX=9606 GN=MRPL24 PE=1 SV=1	3	0,846	0,457	0,527	1129733,688	3862380,338	4834116,163
O00267	SUPT5H	Transcription elongation factor SPT5 OS=Homo sapiens OX=9606 GN=SUPT5H PE=1 SV=1	9	0,723	0,692	0,525	2860489,61	2233107,046	4654024,681
P11166	SLC2A1	Solute carrier family 2, facilitated glucose transporter member 1 OS=Homo sapiens OX=9606 GN	5	1,615	0,324	0,524	4035009,867	10602845,52	12507798,54
P33121	ACSL1	Long-chain-fatty-acid--CoA ligase 1 OS=Homo sapiens OX=9606 GN=ACSL1 PE=1 SV=1	16	1,083	0,364	0,524	1412845,966	2255239,61	5100633,026
P35222	CTNNB1	Catenin beta-1 OS=Homo sapiens OX=9606 GN=CTNNB1 PE=1 SV=1	21	1,038	0,466	0,524	15756033,17	16757091,93	36131193,47
P42696	RBM34	RNA-binding protein 34 OS=Homo sapiens OX=9606 GN=RBM34 PE=1 SV=2	3	0,969	0,666	0,524	612334,4991	150802,859	816049,2302
Q9C0J8	WDR33	pre-mRNA 3' end processing protein WDR33 OS=Homo sapiens OX=9606 GN=WDR33 PE=1 SV=2	4	0,622	0,965	0,523	1809391,924	852998,618	2017427,897
Q9Y5B9	SUPT16H	FACT complex subunit SPT16 OS=Homo sapiens OX=9606 GN=SUPT16H PE=1 SV=1	19	0,508	1,144	0,523	27303722,69	9155448,383	25915275,97
Q14134	TRIM29	Tripartite motif-containing protein 29 OS=Homo sapiens OX=9606 GN=TRIM29 PE=1 SV=2	20	3,816	1,67	0,522	1664873,222	3105149,713	2320609,134
O75431	MTX2	Metaxin-2 OS=Homo sapiens OX=9606 GN=MTX2 PE=1 SV=1	4	0,765	0,774	0,522	1656946,357	1586040,799	2943235,318
Q14533	KRT81	Keratin, type II cuticular Hb1 OS=Homo sapiens OX=9606 GN=KRT81 PE=1 SV=3	3	2,746	0,093	0,521	245247,7083	2562450,216	4925861,465
P49419	ALDH7A1	Alpha-aminoadipic semialdehyde dehydrogenase OS=Homo sapiens OX=9606 GN=ALDH7A1 PE=	17	1,249	0,184	0,521	3965133,252	1105386,107	26712866,55
P22061	PCMT1	Protein-L-isoaspartate(D-aspartate) O-methyltransferase OS=Homo sapiens OX=9606 GN=PCMT1	7	0,844	0,585	0,521	5716016,147	5064105,231	7571813,581
P52815	MRPL12	Large ribosomal subunit protein bL12m OS=Homo sapiens OX=9606 GN=MRPL12 PE=1 SV=2	5	1,221	0,508	0,52	8518864,887	9598380,194	17660394,12
Q8N684	CPSF7	Cleavage and polyadenylation specificity factor subunit 7 OS=Homo sapiens OX=9606 GN=CPSF7	7	0,734	0,69	0,519	2794300,974	2078317,769	3787862,129
Q02127	DHODH	Dihydroorotate dehydrogenase (quinone), mitochondrial OS=Homo sapiens OX=9606 GN=DHOD	2	0,727	0,731	0,519	351132,7439	611532,7591	1132818,01
O15042	U2SURP	U2 snRNP-associated SURP motif-containing protein OS=Homo sapiens OX=9606 GN=U2SURP PE	8	0,7	0,79	0,519	4702436,948	2549891,408	6317491,787
P50502	ST13	Hsc70-interacting protein OS=Homo sapiens OX=9606 GN=ST13 PE=1 SV=2	8	0,199	3,021	0,519	30523604,86	6591305,947	13133299,78
P49756	RBM25	RNA-binding protein 25 OS=Homo sapiens OX=9606 GN=RBM25 PE=1 SV=3	15	0,717	0,782	0,518	10367139,6	11467958,86	12856302,5
Q9BXP5	SRRT	Serrate RNA effector molecule homolog OS=Homo sapiens OX=9606 GN=SRRT PE=1 SV=1	14	0,455	1,163	0,518	10137542,95	4771692,063	8031042,098
G5E9E7	TJP1	Tight junction protein 1 OS=Homo sapiens OX=9606 GN=TJP1 PE=1 SV=1	15	0,417	1,149	0,518	6382885,672	12386040,67	6688066,057
Q9H9B4	SFXN1	Sideroflexin-1 OS=Homo sapiens OX=9606 GN=SFXN1 PE=1 SV=4	12	0,626	0,759	0,517	34526057,16	23590040,22	55269159,72
P33992	MCM5	DNA replication licensing factor MCM5 OS=Homo sapiens OX=9606 GN=MCM5 PE=1 SV=5	16	0,546	0,914	0,517	18531827,95	7233893,561	18859232,5
O43709	BUD23	Probable 18S rRNA (guanine-N(7))-methyltransferase OS=Homo sapiens OX=9606 GN=BUD23 PE	3	0,493	1,189	0,517	1608856,361	604203,4758	1267683,715
Q3MIR4	TMEM30B	Cell cycle control protein 50B OS=Homo sapiens OX=9606 GN=TMEM30B PE=1 SV=1	3	1000	0,001	0,516		47509,07026	446650,453
P35221	CTNNA1	Catenin alpha-1 OS=Homo sapiens OX=9606 GN=CTNNA1 PE=1 SV=1	40	1,678	0,339	0,516	47043219,42	65294420,32	142655607,7
Q9Y697	NFS1	Cysteine desulfurase OS=Homo sapiens OX=9606 GN=NFS1 PE=1 SV=3	6	1,138	0,568	0,516	2673808,458	3026921,782	3835753,767
Q9NR77	PXMP2	Peroxisomal membrane protein 2 OS=Homo sapiens OX=9606 GN=PXMP2 PE=1 SV=3	2	0,938	0,641	0,516	816046,2853	841937,7332	1393412,546
O60313	OPA1	Dynamin-like 120 kDa protein, mitochondrial OS=Homo sapiens OX=9606 GN=OPA1 PE=1 SV=3	25	0,788	0,689	0,516	12468768,34	9433172,544	16652194,91
O43464	HTRA2	Serine protease HTRA2, mitochondrial OS=Homo sapiens OX=9606 GN=HTRA2 PE=1 SV=2	5	0,741	0,562	0,516	1103200,779	1384637,946	2604952,422
Q9HC38	GLOD4	Glyoxalase domain-containing protein 4 OS=Homo sapiens OX=9606 GN=GLOD4 PE=1 SV=1	7	0,303	1,667	0,515	7313014,73	1691258,612	3954940,717
Q969P0	IGSF8	Immunoglobulin superfamily member 8 OS=Homo sapiens OX=9606 GN=IGSF8 PE=1 SV=1	2	3,238	0,257	0,514	649460,7049	479680,062	2256605,908
Q96DH6	MSI2	RNA-binding protein Musashi homolog 2 OS=Homo sapiens OX=9606 GN=MSI2 PE=1 SV=1	7	0,33	1,394	0,514	6580487,516	2036025,113	4133571,334
Q15021	NCAPD2	Condensin complex subunit 1 OS=Homo sapiens OX=9606 GN=NCAPD2 PE=1 SV=3	5	0,277	1,479	0,514	1655811,876	342023,5227	840464,3797
Q13451	FKBP5	Peptidyl-prolyl cis-trans isomerase FKBP5 OS=Homo sapiens OX=9606 GN=FKBP5 PE=1 SV=2	7	0,067	5,89	0,514	5386550,461	390848,7013	751587,3376
Q99873	PRMT1	Protein arginine N-methyltransferase 1 OS=Homo sapiens OX=9606 GN=PRMT1 PE=1 SV=3	12	0,639	0,848	0,513	23027251,41	14078283,89	29099113,13
Q15118	PDK1	[Pyruvate dehydrogenase (acetyl-transferring)] kinase isozyme 1, mitochondrial OS=Homo sapie	2	0,379	1,352	0,512	516795,7279	104454,2163	204138,9073
Q14980	NUMA1	Nuclear mitotic apparatus protein 1 OS=Homo sapiens OX=9606 GN=NUMA1 PE=1 SV=2	56	0,857	0,564	0,511	56178543,17	48345754,38	100141030,2
P50213	IDH3A	Isocitrate dehydrogenase [NAD] subunit alpha, mitochondrial OS=Homo sapiens OX=9606 GN=ID	9	1,48	0,307	0,51	5973554,571	6592151,991	20535848,73
P61960	UFM1	Ubiquitin-fold modifier 1 OS=Homo sapiens OX=9606 GN=UFM1 PE=1 SV=1	4	0,563	1,737	0,51	6275630,108	2630203,149	3595689,806
Q13435	SF3B2	Splicing factor 3B subunit 2 OS=Homo sapiens OX=9606 GN=SF3B2 PE=1 SV=2	23	0,509	0,86	0,51	36371588,07	16395711,66	39898845,6
O43617	TRAPPC3	Trafficking protein particle complex subunit 3 OS=Homo sapiens OX=9606 GN=TRAPPC3 PE=1 SV	2	0,434	1,24	0,51	1033680,27	523414,5618	880823,4515
Q8NE71	ABCF1	ATP-binding cassette sub-family F member 1 OS=Homo sapiens OX=9606 GN=ABCF1 PE=1 SV=2	10	0,341	1,559	0,51	8116386,448	2150874,079	5185364,405
P49736	MCM2	DNA replication licensing factor MCM2 OS=Homo sapiens OX=9606 GN=MCM2 PE=1 SV=4	23	0,503	0,967	0,508	23794207,23	10294316,1	31926000,79
P57729	RAB38	Ras-related protein Rab-38 OS=Homo sapiens OX=9606 GN=RAB38 PE=1 SV=1	4	1,542	0,422	0,507	695230,6706	1013696,74	2057312,384
O00519	FAAH	Fatty-acid amide hydrolase 1 OS=Homo sapiens OX=9606 GN=FAAH PE=1 SV=2	6	1,5	0,345	0,507	190396,1626	295267,6938	1523305,826
Q96T60	PNKP	Bifunctional polynucleotide phosphatase/kinase OS=Homo sapiens OX=9606 GN=PNKP PE=1 SV=	4	0,727	0,692	0,507	322354,7415	248653,8419	1133552,599
Q9Y3T9	NOC2L	Nucleolar complex protein 2 homolog OS=Homo sapiens OX=9606 GN=NOC2L PE=1 SV=4	5	0,516	0,942	0,507	3538721,991	1914372,717	3660325,008
Q14CX7	NAA25	N-alpha-acetyltransferase 25, NatB auxiliary subunit OS=Homo sapiens OX=9606 GN=NAA25 PE=	4	0,465	1,119	0,507	1286876,227	676063,6843	1302757,619
Q16762	TST	Thiosulfate sulfurtransferase OS=Homo sapiens OX=9606 GN=TST PE=1 SV=4	8	2,897	0,186	0,506	2191566,207	5431586,029	11783887,25
Q96I25	RBM17	Splicing factor 45 OS=Homo sapiens OX=9606 GN=RBM17 PE=1 SV=1	6	0,815	0,576	0,505	2974277,27	2534799,761	5937171,017
Q9Y4W2	LAS1L	Ribosomal biogenesis protein LAS1L OS=Homo sapiens OX=9606 GN=LAS1L PE=1 SV=2	3	0,729	0,7	0,505	1235690,362	950953,6098	1740989,997
P52292	KPNA2	Importin subunit alpha-1 OS=Homo sapiens OX=9606 GN=KPNA2 PE=1 SV=1	11	0,43	1,163	0,505	29910215,01	13137674,73	26097511,21
P07339	CTSD	Cathepsin D OS=Homo sapiens OX=9606 GN=CTSD PE=1 SV=1	12	1,784	0,275	0,504	26538142,03	44915859,9	95223282,99
Q8WUY1	THEM6	Protein THEM6 OS=Homo sapiens OX=9606 GN=THEM6 PE=1 SV=2	8	0,967	0,72	0,504	7027065,252	4726725,219	7362820,105
Q5SY16	NOL9	Polynucleotide 5'-hydroxyl-kinase NOL9 OS=Homo sapiens OX=9606 GN=NOL9 PE=1 SV=1	3	0,734	0,897	0,504	1225071,593	615328,7139	1436537,033
J3KS93	EIF4A1	RNA helicase (Fragment) OS=Homo sapiens OX=9606 GN=EIF4A1 PE=1 SV=1	2	0,235	2,128	0,504	40338328,35	10946568,71	18830279,49
P63027	VAMP2	Vesicle-associated membrane protein 2 OS=Homo sapiens OX=9606 GN=VAMP2 PE=1 SV=3	2	1,293	0,387	0,503	5890334,301	7837979,749	14210782,52
Q9NR30	DDX21	Nucleolar RNA helicase 2 OS=Homo sapiens OX=9606 GN=DDX21 PE=1 SV=5	23	0,591	0,862	0,503	27812808,4	14649796,8	32608450,84
Q9NS86	LANCL2	LanC-like protein 2 OS=Homo sapiens OX=9606 GN=LANCL2 PE=1 SV=1	2	0,206	2,087	0,503	461120,2911	149913,1707	338229,6854
Q8WYP5	AHCTF1	Protein ELYS OS=Homo sapiens OX=9606 GN=AHCTF1 PE=1 SV=3	13	0,863	0,597	0,502	3988742,391	3144578,553	8101527,085
P53582	METAP1	Methionine aminopeptidase 1 OS=Homo sapiens OX=9606 GN=METAP1 PE=1 SV=2	3	0,554	0,904	0,501	929119,961	514590,9332	1230625,003
Q96ST2	IWS1	Protein IWS1 homolog OS=Homo sapiens OX=9606 GN=IWS1 PE=1 SV=2	3	0,5	0,846	0,501	850145,2376	421603,5449	1319409,16

P26583	HMGB2	High mobility group protein B2 OS=Homo sapiens OX=9606 GN=HMGB2 PE=1 SV=2	6	0,217	1,848	0,501	36608396,59	9303595,372	20162691,35
Q07817	BCL2L1	Bcl-2-like protein 1 OS=Homo sapiens OX=9606 GN=BCL2L1 PE=1 SV=1	2	4,324	0,116	0,5	162755,9161	725608,1068	1384529,326
A1L0T0	ILVBL	2-hydroxyacyl-CoA lyase 2 OS=Homo sapiens OX=9606 GN=ILVBL PE=1 SV=2	9	1,67	0,306	0,5	1470412,911	2794225,983	5815279,796
Q8N5A5	ZGPAT	Zinc finger CCCH-type with G patch domain-containing protein OS=Homo sapiens OX=9606 GN=Z	2	0,736	0,633	0,5	4164807,611	5053140,398	7001555,812
Q07157	TJP1	Tight junction protein ZO-1 OS=Homo sapiens OX=9606 GN=TJP1 PE=1 SV=3	17	0,447	1,11	0,5	5544659,458	2837074,887	5556078,474
Q8IV08	PLD3	5'-3' exonuclease PLD3 OS=Homo sapiens OX=9606 GN=PLD3 PE=1 SV=1	3	0,32	1,442	0,5	3897852,988	1314982,478	2547095,489
Q7Z5G4	GOLGA7	Golgin subfamily A member 7 OS=Homo sapiens OX=9606 GN=GOLGA7 PE=1 SV=2	2	0,978	0,533	0,499	557442,7311	516296,8932	993276,8932
O60814	H2BC12	Histone H2B type 1-K OS=Homo sapiens OX=9606 GN=H2BC12 PE=1 SV=3	5	0,488	0,99	0,499	1161541105	815955122,3	1210081630
Q9BY44	EIF2A	Eukaryotic translation initiation factor 2A OS=Homo sapiens OX=9606 GN=EIF2A PE=1 SV=3	6	0,455	1,413	0,499	1415789,573	722532,8133	604247,3307
Q9HD34	LYRM4	LYR motif-containing protein 4 OS=Homo sapiens OX=9606 GN=LYRM4 PE=1 SV=1	2	1,766	0,254	0,498	380591,1115	657703,606	1850596,814
O14908	GIPC1	PDZ domain-containing protein GIPC1 OS=Homo sapiens OX=9606 GN=GIPC1 PE=1 SV=2	8	1,64	0,284	0,498	2611553,84	3627364,275	9365170,526
P15924	DSP	Desmoplakin OS=Homo sapiens OX=9606 GN=DSP PE=1 SV=3	23	1,216	0,382	0,497	3591245,808	3938838,135	10868638,74
Q15398	DLGAP5	Disks large-associated protein 5 OS=Homo sapiens OX=9606 GN=DLGAP5 PE=1 SV=2	2	0,449	1,477	0,497	866671,7838	342539,5183	441327,5855
P54725	RAD23A	UV excision repair protein RAD23 homolog A OS=Homo sapiens OX=9606 GN=RAD23A PE=1 SV=	5	1,821	0,302	0,496	974637,4725	1959950,21	4019715,877
P61225	RAP2B	Ras-related protein Rap-2b OS=Homo sapiens OX=9606 GN=RAP2B PE=1 SV=1	2	1,664	0,3	0,496	844032,6119	1395603,742	2993438,79
Q92667	AKAP1	A-kinase anchor protein 1, mitochondrial OS=Homo sapiens OX=9606 GN=AKAP1 PE=1 SV=1	2	0,969	0,471	0,496	196770,8141	190612,6366	667751,8835
Q6PJT7	ZC3H14	Zinc finger CCCH domain-containing protein 14 OS=Homo sapiens OX=9606 GN=ZC3H14 PE=1 SV	6	0,809	0,586	0,496	1567965,285	2983012,054	4113135,342
Q9UKM9	RALY	RNA-binding protein Raly OS=Homo sapiens OX=9606 GN=RALY PE=1 SV=1	14	0,811	0,589	0,495	51881067,2	38184204,38	71648120,96
Q9BQ48	MRPL34	Large ribosomal subunit protein bL34m OS=Homo sapiens OX=9606 GN=MRPL34 PE=1 SV=1	2	0,768	0,577	0,495	549327,2192	450485,5861	1053906,075
Q9C0C9	UBE2O	(E3-independent) E2 ubiquitin-conjugating enzyme OS=Homo sapiens OX=9606 GN=UBE2O PE=1	2	0,314	1,7	0,494	730102,7662	239167,7849	390405,6506
Q9BPX5	ARPC5L	Actin-related protein 2/3 complex subunit 5-like protein OS=Homo sapiens OX=9606 GN=ARPC5	4	0,615	0,567	0,493	7772700,905	4698738,456	10367587,06
P26368	U2AF2	Splicing factor U2AF 65 kDa subunit OS=Homo sapiens OX=9606 GN=U2AF2 PE=1 SV=4	8	0,601	0,851	0,493	16193774,14	8782195,113	18490956,12
Q8N7R7	CCNYL1	Cyclin-Y-like protein 1 OS=Homo sapiens OX=9606 GN=CCNYL1 PE=1 SV=2	2	0,519	0,917	0,493	220804,7975	114571,2392	1139614,783
O96005	CLPTM1	Putative lipid scramblase CLPTM1 OS=Homo sapiens OX=9606 GN=CLPTM1 PE=1 SV=1	11	1,713	0,275	0,492	2138531,508	4125932,236	9866347,846
Q8WWK9	CKAP2	Cytoskeleton-associated protein 2 OS=Homo sapiens OX=9606 GN=CKAP2 PE=1 SV=1	3	0,513	0,909	0,492	1082196,955	547328,2263	1038227,963
Q9Y3L3	SH3BP1	SH3 domain-binding protein 1 OS=Homo sapiens OX=9606 GN=SH3BP1 PE=1 SV=3	5	0,527	0,953	0,49	1916325,12	621156,6364	1897939,019
P05141	SLC25A5	ADP/ATP translocase 2 OS=Homo sapiens OX=9606 GN=SLC25A5 PE=1 SV=7	10	0,816	0,672	0,489	139777690,4	113367502,7	232072410,7
O95235	KIF20A	Kinesin-like protein KIF20A OS=Homo sapiens OX=9606 GN=KIF20A PE=1 SV=1	4	0,731	0,571	0,489	875781,6724	371501,0042	2218019,77
P05386	RPLP1	Large ribosomal subunit protein P1 OS=Homo sapiens OX=9606 GN=RPLP1 PE=1 SV=1	2	0,467	0,996	0,489	27549488,7	10054635,7	26047576,9
Q7Z2W9	MRPL21	Large ribosomal subunit protein bL21m OS=Homo sapiens OX=9606 GN=MRPL21 PE=1 SV=2	3	0,862	0,583	0,488	948539,4208	846551,4558	1851805,009
Q99615	DNAJC7	DnaJ homolog subfamily C member 7 OS=Homo sapiens OX=9606 GN=DNAJC7 PE=1 SV=2	3	0,323	1,798	0,488	4069114,048	769161,7649	2500172,946
Q8WTT2	NOC3L	Nucleolar complex protein 3 homolog OS=Homo sapiens OX=9606 GN=NOC3L PE=1 SV=1	2	0,31	1,53	0,488	630327,5061	175686,9704	574777,7546
Q9H773	DCTPP1	dCTP pyrophosphatase 1 OS=Homo sapiens OX=9606 GN=DCTPP1 PE=1 SV=1	7	0,844	0,715	0,487	3654237,162	2779217,652	5583459,987
Q9P2W9	STX18	Syntaxin-18 OS=Homo sapiens OX=9606 GN=STX18 PE=1 SV=1	2	0,772	0,606	0,487	209171,6601	138265,2402	317459,8594
O00194	RAB27B	Ras-related protein Rab-27B OS=Homo sapiens OX=9606 GN=RAB27B PE=1 SV=4	2	5,13	0,095	0,486	23861,28095	122402,2011	251690,5671
Q9NXG6	P4HTM	Transmembrane prolyl 4-hydroxylase OS=Homo sapiens OX=9606 GN=P4HTM PE=1 SV=2	2	2,704	0,168	0,486	88682,05363	372925,8672	775355,5249
Q96ST3	SIN3A	Paired amphipathic helix protein Sin3a OS=Homo sapiens OX=9606 GN=SIN3A PE=1 SV=2	14	0,53	0,964	0,486	6999171,869	3882264,693	7247381,268
Q9BXS6	NUSAP1	Nucleolar and spindle-associated protein 1 OS=Homo sapiens OX=9606 GN=NUSAP1 PE=1 SV=1	2	0,295	1,433	0,486	1675761,222	404988,7768	1114365,592
P12429	ANXA3	Annexin A3 OS=Homo sapiens OX=9606 GN=ANXA3 PE=1 SV=3	10	4,989	0,118	0,485	441377,6522	13461969,01	27616654,08
O95425	SVIL	Supervillin OS=Homo sapiens OX=9606 GN=SVIL PE=1 SV=2	10	2,74	0,177	0,485	942641,5442	2801759,481	5594374,551
Q99614	TTC1	Tetratricopeptide repeat protein 1 OS=Homo sapiens OX=9606 GN=TTC1 PE=1 SV=1	8	0,677	0,745	0,485	5872816,192	3594130,001	8088095,311
P55957	BID	BH3-interacting domain death agonist OS=Homo sapiens OX=9606 GN=BID PE=1 SV=1	6	0,611	0,6	0,485	2574854,005	1338289,753	4384927,073
Q13310	PABPC4	Polyadenylate-binding protein 4 OS=Homo sapiens OX=9606 GN=PABPC4 PE=1 SV=1	13	0,577	0,93	0,485	13029557,32	6571325,025	14618465,36
P48047	ATP5PO	ATP synthase subunit O, mitochondrial OS=Homo sapiens OX=9606 GN=ATP5PO PE=1 SV=1	11	0,78	0,73	0,484	23298783,47	22294464,1	35114127,38
P27694	RPA1	Replication protein A 70 kDa DNA-binding subunit OS=Homo sapiens OX=9606 GN=RPA1 PE=1 SV	12	0,397	1,248	0,484	13572498,88	5113419,416	10359584,48
Q15269	PWP2	Periodic tryptophan protein 2 homolog OS=Homo sapiens OX=9606 GN=PWP2 PE=1 SV=2	8	0,68	0,625	0,483	2941010,099	1682320,614	4048741,584
P11137	MAP2	Microtubule-associated protein 2 OS=Homo sapiens OX=9606 GN=MAP2 PE=1 SV=4	11	0,191	6,17	0,481	6822140,063	392893,1159	606360,9785
Q01970	PLCB3	1-phosphatidylinositol 4,5-bisphosphate phosphodiesterase beta-3 OS=Homo sapiens OX=9606	3	0,972	0,522	0,48	812604,8726	640326,4467	1496300,386
O60264	SMARCA5	SWI/SNF-related matrix-associated actin-dependent regulator of chromatin subfamily A membe	20	0,448	1,168	0,48	43312989,9	29468205,69	30366184,12
O60841	EIF5B	Eukaryotic translation initiation factor 5B OS=Homo sapiens OX=9606 GN=EIF5B PE=1 SV=4	7	0,337	1,179	0,48	3060689,982	965664,3084	2144922,445
Q9H2U2	PPA2	Inorganic pyrophosphatase 2, mitochondrial OS=Homo sapiens OX=9606 GN=PPA2 PE=1 SV=2	14	0,943	0,447	0,479	7584028,422	6487580,548	13754252,18
O43815	STRN	Striatin OS=Homo sapiens OX=9606 GN=STRN PE=1 SV=4	2	0,939	0,504	0,478	428028,7816	647155,5463	1359406,736
P82933	MRPS9	Small ribosomal subunit protein uS9m OS=Homo sapiens OX=9606 GN=MRPS9 PE=1 SV=2	7	0,909	0,73	0,478	3313486,842	3049622,401	5233133,214
Q8WWC4	MAIP1	m-AAA protease-interacting protein 1, mitochondrial OS=Homo sapiens OX=9606 GN=MAIP1 PE	3	0,672	0,828	0,478	1115197,189	773233,5761	1361389,793
Q9BVG4	PBDC1	Protein PBDC1 OS=Homo sapiens OX=9606 GN=PBDC1 PE=1 SV=1	3	0,65	0,808	0,478	2108118,266	1284114,064	2617049,556
Q96CT7	CCDC124	Coiled-coil domain-containing protein 124 OS=Homo sapiens OX=9606 GN=CCDC124 PE=1 SV=1	4	0,546	0,929	0,478	3558650,387	2469663,95	4058032,479
P63165	SUMO1	Small ubiquitin-related modifier 1 OS=Homo sapiens OX=9606 GN=SUMO1 PE=1 SV=1	3	0,474	1,074	0,478	6347378,984	3010741,386	5912757,372
Q9BQ67	GRWD1	Glutamate-rich WD repeat-containing protein 1 OS=Homo sapiens OX=9606 GN=GRWD1 PE=1 S	4	0,628	0,742	0,477	2054484,348	1186809,472	2723967,94
O75691	UTP20	Small subunit processome component 20 homolog OS=Homo sapiens OX=9606 GN=UTP20 PE=1	9	0,647	0,747	0,476	1590337,699	857073,8567	2215511,638
Q9NVT9	ARMC1	Armadillo repeat-containing protein 1 OS=Homo sapiens OX=9606 GN=ARMC1 PE=1 SV=1	2	0,903	0,395	0,475	254042,5223	424847,9197	1017862,727
Q5T440	IBA57	Putative transferase CAF17, mitochondrial OS=Homo sapiens OX=9606 GN=IBA57 PE=1 SV=1	3	1000	0,001	0,474		122577,2092	365814,4066
O43520	ATP8B1	Phospholipid-transporting ATPase IC OS=Homo sapiens OX=9606 GN=ATP8B1 PE=1 SV=3	17	1,866	0,617	0,474	424673,9869	1052954,022	741046,1465
P16219	ACADS	Short-chain specific acyl-CoA dehydrogenase, mitochondrial OS=Homo sapiens OX=9606 GN=AC	6	1000	0,001	0,473		268666,7168	2407877,058
Q9P206	NHSL3	NHS-like protein 3 OS=Homo sapiens OX=9606 GN=NHSL3 PE=1 SV=2	7	4,114	0,116	0,472	141232,9887	904472,6721	3161534,835
P10696	ALPG	Alkaline phosphatase, germ cell type OS=Homo sapiens OX=9606 GN=ALPG PE=1 SV=4	3	0,824	0,573	0,472	766006,6404	657046,641	3168828,254
Q96KP4	CNDP2	Cytosolic non-specific dipeptidase OS=Homo sapiens OX=9606 GN=CNDP2 PE=1 SV=2	18	0,585	0,841	0,471	10799851,28	6146625,236	13380122,64
Q8TB37	NUBPL	Iron-sulfur cluster transfer protein NUBPL OS=Homo sapiens OX=9606 GN=NUBPL PE=1 SV=3	2	1,141	0,356	0,47	321783,2755	442371,7717	894608,6825
Q9BW92	TARS2	Threonine--tRNA ligase, mitochondrial OS=Homo sapiens OX=9606 GN=TARS2 PE=1 SV=1	7	0,899	0,507	0,47	1765126,118	1562315,421	3519519,385
O75152	ZC3H11A	Zinc finger CCCH domain-containing protein 11A OS=Homo sapiens OX=9606 GN=ZC3H11A PE=1	6	0,861	0,563	0,47	3120074,24	2566106,048	4988820,526
Q8NFH5	NUP35	Nucleoporin NUP35 OS=Homo sapiens OX=9606 GN=NUP35 PE=1 SV=1	4	0,841	0,774	0,469	2872277,19	3541406,953	3580236,498
Q9H078	CLPB	Mitochondrial disaggregase OS=Homo sapiens OX=9606 GN=CLPB PE=1 SV=1	4	0,732	0,48	0,469	1587396,061	688652,7377	3065263,227
P31483	TIA1	Cytotoxic granule associated RNA binding protein TIA1 OS=Homo sapiens OX=9606 GN=TIA1 PE=	2	0,143	3,272	0,469	1870504,842	494297,0069	632785,78
O75494	SRSF10	Serine/arginine-rich splicing factor 10 OS=Homo sapiens OX=9606 GN=SRSF10 PE=1 SV=1	5	1,05	0,714	0,468	7363239,456	7723779	12205917,09
O76031	CLPX	ATP-dependent Clp protease ATP-binding subunit clpX-like, mitochondrial OS=Homo sapiens OX	8	0,841	0,601	0,467	2896744,984	2899016,982	6423383,54
P22830	FECH	Ferrochelatase, mitochondrial OS=Homo sapiens OX=9606 GN=FECH PE=1 SV=2	3	1000	0,001	0,466		582675,4996	464596,4097
P09874	PARP1	Poly [ADP-ribose] polymerase 1 OS=Homo sapiens OX=9606 GN=PARP1 PE=1 SV=4	36	0,171	2,555	0,466	85589642,72	12847953,37	31596346,73
Q96N66	MBOAT7	Lysophospholipid acyltransferase 7 OS=Homo sapiens OX=9606 GN=MBOAT7 PE=1 SV=2	5	3,904	0,13	0,465	1165971,872	4871693,547	11106816,26
Q5VWZ2	LYPLAL1	Lysophospholipase-like protein 1 OS=Homo sapiens OX=9606 GN=LYPLAL1 PE=1 SV=3	2	1,306	0,362	0,465	45942205,03	59472423,4	103926083,6
O75153	CLUH	Clustered mitochondria protein homolog OS=Homo sapiens OX=9606 GN=CLUH PE=1 SV=2	3	0,64	1,021	0,464	1459329,771	749451,5601	1954743,892
Q9H0A0	NAT10	RNA cytidine acetyltransferase OS=Homo sapiens OX=9606 GN=NAT10 PE=1 SV=2	14	0,507	0,911	0,463	4439456,848	2122233,285	5345310,731
A0A669KB77	MAP2	Microtubule-associated protein OS=Homo sapiens OX=9606 GN=MAP2 PE=1 SV=1	9	0,338	1,719	0,463	8159750,07	1216680,768	4635054,44
O15027	SEC16A	Protein transport protein Sec16A OS=Homo sapiens OX=9606 GN=SEC16A PE=1 SV=4	9	1,143	0,504	0,462	1233159,427	1882438,363	5961272,419
Q9UNX4	WDR3	WD repeat-containing protein 3 OS=Homo sapiens OX=9606 GN=WDR3 PE=1 SV=1	5	0,525	0,71	0,462	830140,4426	75777,07616	1356274,381
Q6P161	MRPL54	Large ribosomal subunit protein mL54 OS=Homo sapiens OX=9606 GN=MRPL54 PE=1 SV=1	2	1,66	0,266	0,46	296879,0603	492898,8267	1071289,101
Q9ULA0	DNPEP	Aspartyl aminopeptidase OS=Homo sapiens OX=9606 GN=DNPEP PE=1 SV=2	3	1,234	0,398	0,46	935147,2814	995167,7292	2712638,306
Q86U38	NOP9	Nucleolar protein 9 OS=Homo sapiens OX=9606 GN=NOP9 PE=1 SV=1	6	0,551	0,802	0,46	3115440,982	1661808,005	3791998,595
Q9H8H3	TMT1A	Thiol S-methyltransferase TMT1A OS=Homo sapiens OX=9606 GN=TMT1A PE=1 SV=1	4	0,335	1,367	0,46	4387301,659	1534997,545	468093,4278
O00425	IGF2BP3	Insulin-like growth factor 2 mRNA-binding protein 3 OS=Homo sapiens OX=9606 GN=IGF2BP3 PE	14	0,301	1,822	0,46	34779022,88	7167353,995	16598464,76
P30837	ALDH1B1	Aldehyde dehydrogenase X, mitochondrial OS=Homo sapiens OX=9606 GN=ALDH1B1 PE=1 SV=4	14	1,395	0,336	0,459	6259444,481	10552498,31	20421706,96
Q9Y3D9	MRPS23	Small ribosomal subunit protein mS23 OS=Homo sapiens OX=9606 GN=MRPS23 PE=1 SV=2	6	0,712	0,601	0,459	2998893,874	2158484,183	6479776,699
Q9BVG9	PTDSS2	Phosphatidylserine synthase 2 OS=Homo sapiens OX=9606 GN=PTDSS2 PE=1 SV=1	3	0,635	0,755	0,458	1171396,571	773822,8501	1513846,898
Q9NPI6	DCP1A	mRNA-decapping enzyme 1A OS=Homo sapiens OX=9606 GN=DCP1A PE=1 SV=3	2	0,418	1,129	0,458	513052,3107	227509,4531	388877,9858
P09001	MRPL3	Large ribosomal subunit protein uL3m OS=Homo sapiens OX=9606 GN=MRPL3 PE=1 SV=1	4	1,072	0,575	0,457	1348073,405	1061365,14	2423199,679
P57088	TMEM33	Transmembrane protein 33 OS=Homo sapiens OX=9606 GN=TMEM33 PE=1 SV=2	6	0,829	0,679	0,457	6200135,319	4916019,613	11743434,67
Q63HN8	RNF213	E3 ubiquitin-protein ligase RNF213 OS=Homo sapiens OX=9606 GN=RNF213 PE=1 SV=3	58	1,891	0,203	0,456	2414568,167	8705565,52	23337603,32
Q96GC5	MRPL48	Large ribosomal subunit protein mL48 OS=Homo sapiens OX=9606 GN=MRPL48 PE=1 SV=2	2	1,295	0,349	0,456	694840,7865	1084392,109	1974471,44
Q01780	EXOSC10	Exosome complex component 10 OS=Homo sapiens OX=9606 GN=EXOSC10 PE=1 SV=2	12	0,636	0,724	0,456	3509455,41	2102195,01	7909523,821
P12236	SLC25A6	ADP/ATP translocase 3 OS=Homo sapiens OX=9606 GN=SLC25A6 PE=1 SV=4	2	0,937	0,443	0,455	6472146,122	6044300,791	15133506,43
O76021	RSL1D1	Ribosomal L1 domain-containing protein 1 OS=Homo sapiens OX=9606 GN=RSL1D1 PE=1 SV=3	9	0,791	0,645	0,455	4579319,797	4466353,506	7526104,403
Q9BWU0	SLC4A1AP	Kanadaptin OS=Homo sapiens OX=9606 GN=SLC4A1AP PE=1 SV=2	3	0,229	2,182	0,455	338063,9866	88291,69968	208991,9941
P02751	FN1	Fibronectin OS=Homo sapiens OX=9606 GN=FN1 PE=1 SV=5	26	2,768	0,162	0,453	7481903,838	23087350,46	53110092,29
Q15287	RNPS1	RNA-binding protein with serine-rich domain 1 OS=Homo sapiens OX=9606 GN=RNPS1 PE=1 SV=	3	0,74	0,612	0,453	2873893,454	2087477,62	4111028,728
P25205	MCM3	DNA replication licensing factor MCM3 OS=Homo sapiens OX=9606 GN=MCM3 PE=1 SV=3	28	0,434	1,091	0,453	28145866,84	12604860,51	31625858,01
Q13616	CUL1	Cullin-1 OS=Homo sapiens OX=9606 GN=CUL1 PE=1 SV=2	4	0,507	1,182	0,452	2188997,966	338827,6941	1887720,621
Q96E11	MRRF	Ribosome-recycling factor, mitochondrial OS=Homo sapiens OX=9606 GN=MRRF PE=1 SV=1	3	1,211	0,26	0,451	362737,9711	674357,9496	2266524,092
P42765	ACAA2	3-ketoacyl-CoA thiolase, mitochondrial OS=Homo sapiens OX=9606 GN=ACAA2 PE=1 SV=2	12	0,992	0,446	0,451	20685464	17029536,2	46861692,58
Q9H1I8	ASCC2	Activating signal cointegrator 1 complex subunit 2 OS=Homo sapiens OX=9606 GN=ASCC2 PE=1 S	2	0,494	0,887	0,451	446256,5668	230820,8413	515947,4492
P22087	FBL	rRNA 2'-O-methyltransferase fibrillarin OS=Homo sapiens OX=9606 GN=FBL PE=1 SV=2	11	0,888	0,701	0,45	11114745,58	9583286,07	29473295,94
P61457	PCBD1	Pterin-4-alpha-carbinolamine dehydratase OS=Homo sapiens OX=9606 GN=PCBD1 PE=1 SV=2	3	0,656	0,666	0,45	324294,0163	204981,9618	487005,6664
Q9BYG3	NIFK	MKI67 FHA domain-interacting nucleolar phosphoprotein OS=Homo sapiens OX=9606 GN=NIFK I	8	0,51	0,652	0,45	1650848,774	1159468,248	3192817,572
Q06481	APLP2	Amyloid beta precursor like protein 2 OS=Homo sapiens OX=9606 GN=APLP2 PE=1 SV=2	3	0,932	0,615	0,449	1242633,333	1164279,857	1901771,268

Q16629	SRSF7	Serine/arginine-rich splicing factor 7 OS=Homo sapiens OX=9606 GN=SRSF7 PE=1 SV=1	3	0,535	0,726	0,449	9152684,809	4211383,266	10269407,07
Q16363	LAMA4	Laminin subunit alpha-4 OS=Homo sapiens OX=9606 GN=LAMA4 PE=1 SV=4	6	7,991	0,783	0,448	8469562,196	6053796,712	10724770,13
P62253	UBE2G1	Ubiquitin-conjugating enzyme E2 G1 OS=Homo sapiens OX=9606 GN=UBE2G1 PE=1 SV=3	3	0,242	3,141	0,448	1026866,649	167295,0163	399868,3704
P62851	RPS25	Small ribosomal subunit protein eS25 OS=Homo sapiens OX=9606 GN=RPS25 PE=1 SV=1	5	0,382	1,272	0,447	44008285,28	11714945,04	35871069,02
Q9NQW6	ANLN	Anillin OS=Homo sapiens OX=9606 GN=ANLN PE=1 SV=2	3	0,985	0,435	0,446	246284,0902	245255,3403	516354,3428
Q6BCY4	CYB5R2	NADH-cytochrome b5 reductase 2 OS=Homo sapiens OX=9606 GN=CYB5R2 PE=1 SV=1	3	0,096	3,203	0,446	1176255,461	195989,8573	288608,7587
Q8IZL8	PELP1	Proline-, glutamic acid- and leucine-rich protein 1 OS=Homo sapiens OX=9606 GN=PELP1 PE=1 S	9	0,702	0,655	0,445	3246590,75	1331833,923	5155758,396
Q7LBR1	CHMP1B	Charged multivesicular body protein 1b OS=Homo sapiens OX=9606 GN=CHMP1B PE=1 SV=1	2	0,39	1,115	0,445	1188209,732	467196,8951	721164,8471
O60885	BRD4	Bromodomain-containing protein 4 OS=Homo sapiens OX=9606 GN=BRD4 PE=1 SV=2	5	0,691	0,562	0,444	2095504,373	1562642,068	3866291,998
Q9NZL9	MAT2B	Methionine adenosyltransferase 2 subunit beta OS=Homo sapiens OX=9606 GN=MAT2B PE=1 SV	8	0,27	1,803	0,444	12934255,37	2078574,062	7090818,702
Q9ULZ3	PYCARD	Apoptosis-associated speck-like protein containing a CARD OS=Homo sapiens OX=9606 GN=PYC	5	3,982	0,072	0,442	179844,5397	989690,6928	2336070,983
Q9H6E4	CCDC134	Coiled-coil domain-containing protein 134 OS=Homo sapiens OX=9606 GN=CCDC134 PE=1 SV=1	5	0,435	0,769	0,441	1896607,984	304750,8418	2357242,851
P15408	FOSL2	Fos-related antigen 2 OS=Homo sapiens OX=9606 GN=FOSL2 PE=1 SV=1	2	1000	0,001	0,44		529034,1224	1884430,765
Q15050	RRS1	Ribosome biogenesis regulatory protein homolog OS=Homo sapiens OX=9606 GN=RRS1 PE=1 SV	7	0,723	0,578	0,44	3030452,912	1683400,721	5114591,59
Q96FZ7	CHMP6	Charged multivesicular body protein 6 OS=Homo sapiens OX=9606 GN=CHMP6 PE=1 SV=3	2	1,992	0,22	0,439	387840,9489	816849,4415	1695568,35
Q9NPF5	DMAP1	DNA methyltransferase 1-associated protein 1 OS=Homo sapiens OX=9606 GN=DMAP1 PE=1 SV=	3	0,774	0,815	0,438	1181675,823	600072,3444	837137,5733
Q9UIW2	PLXNA1	Plexin-A1 OS=Homo sapiens OX=9606 GN=PLXNA1 PE=1 SV=3	3	0,424	0,796	0,438	476473,5357	609375,0846	1055350,627
P78324	SIRPA	Tyrosine-protein phosphatase non-receptor type substrate 1 OS=Homo sapiens OX=9606 GN=SII	2		0,294	0,437	188048,2157	156951,5918	1055156,999
P62306	SNRPF	Small nuclear ribonucleoprotein F OS=Homo sapiens OX=9606 GN=SNRPF PE=1 SV=1	3	0,604	1,76	0,437	7169811,265	4405335,4	4187348,493
O43143	DHX15	ATP-dependent RNA helicase DHX15 OS=Homo sapiens OX=9606 GN=DHX15 PE=1 SV=2	18	0,38	1,082	0,437	30154620,28	9977763,292	24585105,01
Q9HD23	MRS2	Magnesium transporter MRS2 homolog, mitochondrial OS=Homo sapiens OX=9606 GN=MRS2 PE	2	0,745	0,61	0,436	587839,6347	432228,8486	920005,8691
Q9BVP2	GNL3	Guanine nucleotide-binding protein-like 3 OS=Homo sapiens OX=9606 GN=GNL3 PE=1 SV=2	12	0,633	0,641	0,436	4758495,943	2420245,985	7414318,09
Q9NVI1	FANCI	Fanconi anemia group I protein OS=Homo sapiens OX=9606 GN=FANCI PE=1 SV=4	6	0,359	1,103	0,436	1243428,49	367144,0859	1440359,373
Q9Y676	MRPS18B	Small ribosomal subunit protein mS40 OS=Homo sapiens OX=9606 GN=MRPS18B PE=1 SV=1	4	1,304	0,435	0,435	1923477,431	1812541,821	4162433,585
Q99848	EBNA1BP2	Probable rRNA-processing protein EBP2 OS=Homo sapiens OX=9606 GN=EBNA1BP2 PE=1 SV=2	9	0,614	0,686	0,435	5717499,244	4848588,725	11706407,1
Q12931	TRAP1	Heat shock protein 75 kDa, mitochondrial OS=Homo sapiens OX=9606 GN=TRAP1 PE=1 SV=3	19	1,074	0,386	0,434	23333054,53	24667029,36	62971614,88
Q9H7B2	RPF2	Ribosome production factor 2 homolog OS=Homo sapiens OX=9606 GN=RPF2 PE=1 SV=2	6	0,594	0,705	0,434	1779384,426	1334370,454	2851846,875
Q9UJS0	SLC25A13	Electrogenic aspartate/glutamate antiporter SLC25A13, mitochondrial OS=Homo sapiens OX=96	18	0,42	1,052	0,434	45784594,11	62243856,34	66258678,36
P28838	LAP3	Cytosol aminopeptidase OS=Homo sapiens OX=9606 GN=LAP3 PE=1 SV=3	15	2,424	0,175	0,433	33982978,43	72637479,18	53172588,75
Q86VR2	RETREG3	Reticulophagy regulator 3 OS=Homo sapiens OX=9606 GN=RETREG3 PE=1 SV=1	2	0,661	0,607	0,433	761899,5695	741226,9176	1238886,671
Q07021	C1QBP	Complement component 1 Q subcomponent-binding protein, mitochondrial OS=Homo sapiens (	6	0,619	0,74	0,433	44587146,51	25984423,57	58930651,6
Q08945	SSRP1	FACT complex subunit SSRP1 OS=Homo sapiens OX=9606 GN=SSRP1 PE=1 SV=1	11	0,336	1,349	0,433	20020668,41	5784895,341	15632037,92
O95232	LUC7L3	Luc7-like protein 3 OS=Homo sapiens OX=9606 GN=LUC7L3 PE=1 SV=2	6	0,463	0,985	0,432	5430157,112	2257074,721	5764831,929
Q9NQ50	MRPL40	Large ribosomal subunit protein mL40 OS=Homo sapiens OX=9606 GN=MRPL40 PE=1 SV=1	3	1,11	0,35	0,431	775737,983	925658,4637	2138655,004
Q92804	TAF15	TATA-binding protein-associated factor 2N OS=Homo sapiens OX=9606 GN=TAF15 PE=1 SV=1	4	0,981	0,647	0,431	6570713,432	4001410,024	6309992,625
A2RRP1	NBAS	NBAS subunit of NRZ tethering complex OS=Homo sapiens OX=9606 GN=NBAS PE=1 SV=2	4	0,711	0,711	0,431	1155537,653	991571,8088	981298,7858
Q8NI36	WDR36	WD repeat-containing protein 36 OS=Homo sapiens OX=9606 GN=WDR36 PE=1 SV=1	7	0,632	0,762	0,431	1708072,148	1254482,116	3274233,429
Q14978	NOLC1	Nucleolar and coiled-body phosphoprotein 1 OS=Homo sapiens OX=9606 GN=NOLC1 PE=1 SV=2	3	0,522	0,762	0,431	1378393,891	1542147,622	3726219,689
Q96CM8	ACSF2	Medium-chain acyl-CoA ligase ACSF2, mitochondrial OS=Homo sapiens OX=9606 GN=ACSF2 PE=1	13	1,784	0,213	0,43	1439949,925	2498576,198	8893300,828
Q96GQ7	DDX27	Probable ATP-dependent RNA helicase DDX27 OS=Homo sapiens OX=9606 GN=DDX27 PE=1 SV=	9	0,87	0,627	0,43	3202317,371	4450292,326	5579483,379
Q9BZJ0	CRNKL1	Crooked neck-like protein 1 OS=Homo sapiens OX=9606 GN=CRNKL1 PE=1 SV=4	7	0,56	0,758	0,43	2678813,509	828455,3332	3480718,637
Q9UI10	EIF2B4	Translation initiation factor eIF2B subunit delta OS=Homo sapiens OX=9606 GN=EIF2B4 PE=1 SV	3	0,321	1,342	0,43	2785722,214	91023,83564	211476,3844
O43148	RNMT	mRNA cap guanine-N7 methyltransferase OS=Homo sapiens OX=9606 GN=RNMT PE=1 SV=1	5	0,625	0,934	0,429	2464705,659	2028601,688	2297585,431
P50148	GNAQ	Guanine nucleotide-binding protein G(q) subunit alpha OS=Homo sapiens OX=9606 GN=GNAQ P	4	1,799	0,727	0,428	395405,2759	723972,4388	1600755,427
Q9NZ01	TECR	Very-long-chain enoyl-CoA reductase OS=Homo sapiens OX=9606 GN=TECR PE=1 SV=1	10	1,259	0,383	0,428	22299979,94	27295740,39	60572641,64
Q01105	SET	Protein SET OS=Homo sapiens OX=9606 GN=SET PE=1 SV=3	9	0,53	0,784	0,428	127888639	64445900,28	164475936,9
P0DME0	SETSIP	Protein SETSIP OS=Homo sapiens OX=9606 GN=SETSIP PE=1 SV=2	4	0,467	0,97	0,428	93705671,27	43224833,33	98878175,33
P11169	SLC2A3	Solute carrier family 2, facilitated glucose transporter member 3 OS=Homo sapiens OX=9606 GN	4	0,075	3,696	0,428	3548453,19	243723,8981	902272,362
Q9BV38	WDR18	WD repeat-containing protein 18 OS=Homo sapiens OX=9606 GN=WDR18 PE=1 SV=2	6	0,899	0,462	0,427	3251564,924	3250126,078	7562375,324
Q9NVI7	ATAD3A	ATPase family AAA domain-containing protein 3A OS=Homo sapiens OX=9606 GN=ATAD3A PE=1	7	0,707	0,606	0,427	23631397,58	14212372,09	39980216,18
Q9H6W3	RIOX1	Ribosomal oxygenase 1 OS=Homo sapiens OX=9606 GN=RIOX1 PE=1 SV=2	5	0,799	0,561	0,426	820972,1737	391138,7945	1561668,191
Q5JTH9	RRP12	RRP12-like protein OS=Homo sapiens OX=9606 GN=RRP12 PE=1 SV=2	8	0,645	0,668	0,426	1799750,233	1027727,753	3660425,9
Q9P2N5	RBM27	RNA-binding protein 27 OS=Homo sapiens OX=9606 GN=RBM27 PE=1 SV=2	4	0,638	0,702	0,424	1482395,239	996629,8655	2692914,671
Q99442	SEC62	Translocation protein SEC62 OS=Homo sapiens OX=9606 GN=SEC62 PE=1 SV=1	3	0,503	1,072	0,423	2287228,036	1248024,461	2342143,094
Q9UHL4	DPP7	Dipeptidyl peptidase 2 OS=Homo sapiens OX=9606 GN=DPP7 PE=1 SV=3	3	4,096	0,108	0,422	333029,3805	1027347,367	2450418,852
P18754	RCC1	Regulator of chromosome condensation OS=Homo sapiens OX=9606 GN=RCC1 PE=1 SV=1	10	0,743	0,57	0,422	20053088,38	10573295,96	36616684,02
P52435	POLR2J	DNA-directed RNA polymerase II subunit RPB11-a OS=Homo sapiens OX=9606 GN=POLR2J PE=1	2	0,355	1,201	0,422	861935,3268	236348,2311	722293,8645
Q9H6R4	NOL6	Nucleolar protein 6 OS=Homo sapiens OX=9606 GN=NOL6 PE=1 SV=2	6	0,829	0,508	0,421	2504041,516	34082111,67	6352426,683
Q8TAE8	GADD45GIP1	Large ribosomal subunit protein mL64 OS=Homo sapiens OX=9606 GN=GADD45GIP1 PE=1 SV=1	5	1,106	0,377	0,419	2227177,668	1955206,984	5906206,995
Q8N3X1	FNBP4	Formin-binding protein 4 OS=Homo sapiens OX=9606 GN=FNBP4 PE=1 SV=3	2	0,693	0,737	0,419	174656,1269	246530,4359	644247,2972
Q92841	DDX17	Probable ATP-dependent RNA helicase DDX17 OS=Homo sapiens OX=9606 GN=DDX17 PE=1 SV=	17	0,675	0,633	0,419	43306808,65	26622720,41	69585605,23
Q14160	SCRIB	Protein scribble homolog OS=Homo sapiens OX=9606 GN=SCRIB PE=1 SV=5	17	0,842	0,536	0,418	5001611,708	3581348,28	10900830,69
Q9UKD2	MRTO4	mRNA turnover protein 4 homolog OS=Homo sapiens OX=9606 GN=MRTO4 PE=1 SV=2	5	0,508	0,877	0,418	3756074,895	988616,045	4067529,107
Q96FX7	TRMT61A	tRNA (adenine(58)-N(1))-methyltransferase catalytic subunit TRMT61A OS=Homo sapiens OX=96	2	0,34	1,228	0,418	432006,7482	146833,392	351676,8492
Q5JRX3	PITRM1	Presequence protease, mitochondrial OS=Homo sapiens OX=9606 GN=PITRM1 PE=1 SV=3	16	1,948	0,216	0,417	2480293,598	4956785,272	15893805,96
Q96SI9	STRBP	Spermatid perinuclear RNA-binding protein OS=Homo sapiens OX=9606 GN=STRBP PE=1 SV=1	7	0,386	1,042	0,417	20283711,82	10995334,07	19362265,72
P16989	YBX3	Y-box-binding protein 3 OS=Homo sapiens OX=9606 GN=YBX3 PE=1 SV=4	7	0,794	0,479	0,416	3200227,485	2373692,771	6577464,194
P07947	YES1	Tyrosine-protein kinase Yes OS=Homo sapiens OX=9606 GN=YES1 PE=1 SV=3	3	0,682	0,61	0,416	364435,8632	248592,8722	1196279,065
Q01130	SRSF2	Serine/arginine-rich splicing factor 2 OS=Homo sapiens OX=9606 GN=SRSF2 PE=1 SV=4	4	0,424	0,91	0,416	15723057,39	7157582,897	15732478,35
Q13907	IDI1	Isopentenyl-diphosphate Delta-isomerase 1 OS=Homo sapiens OX=9606 GN=IDI1 PE=1 SV=2	3	0,177	2,311	0,414	2109150,168	364617,5263	904079,3065
Q13637	RAB32	Ras-related protein Rab-32 OS=Homo sapiens OX=9606 GN=RAB32 PE=1 SV=3	4	2,146	0,218	0,413	421362,989	951908,8423	1939863,95
Q9HBM1	SPC25	Kinetochore protein Spc25 OS=Homo sapiens OX=9606 GN=SPC25 PE=1 SV=1	2	0,737	0,588	0,413	641921,1256	426356,1989	807877,6749
Q5T6F2	UBAP2	Ubiquitin-associated protein 2 OS=Homo sapiens OX=9606 GN=UBAP2 PE=1 SV=1	2	0,644	1,047	0,413	726220,1035	245504,3087	785040,3137
Q96RQ3	MCCC1	Methylcrotonoyl-CoA carboxylase subunit alpha, mitochondrial OS=Homo sapiens OX=9606 GN=	11	1,513	0,294	0,412	1377914,047	1621022,42	5779506,816
Q9P2R7	SUCLA2	Succinate--CoA ligase [ADP-forming] subunit beta, mitochondrial OS=Homo sapiens OX=9606 GN	11	0,908	0,492	0,412	6254596,192	6029057,742	13885766,06
Q9NZM5	NOP53	Ribosome biogenesis protein NOP53 OS=Homo sapiens OX=9606 GN=NOP53 PE=1 SV=2	3	0,878	0,578	0,412	373644,444	298379,109	1196366,144
Q96D15	RCN3	Reticulocalbin-3 OS=Homo sapiens OX=9606 GN=RCN3 PE=1 SV=1	3	0,325	4,219	0,412	1612392,778	180102,2356	471658,9151
Q9Y512	SAMM50	Sorting and assembly machinery component 50 homolog OS=Homo sapiens OX=9606 GN=SAMM	11	0,846	0,533	0,411	7864943,74	7477552,442	12398314,56
Q14807	KIF22	Kinesin-like protein KIF22 OS=Homo sapiens OX=9606 GN=KIF22 PE=1 SV=5	5	0,837	0,515	0,411	920709,2598	752234,841	1848854,539
Q8NEG4	FAM83F	Protein FAM83F OS=Homo sapiens OX=9606 GN=FAM83F PE=1 SV=1	4	1000	0,001	0,41		105150,1354	498690,3615
P00505	GOT2	Aspartate aminotransferase, mitochondrial OS=Homo sapiens OX=9606 GN=GOT2 PE=1 SV=3	18	0,92	0,498	0,41	48906247,82	43417130,54	113570217,4
Q96P11	NSUN5	28S rRNA (cytosine-C(5))-methyltransferase OS=Homo sapiens OX=9606 GN=NSUN5 PE=1 SV=2	3	0,746	0,565	0,41	1325505,824	800961,5185	2147262,972
O75190	DNAJB6	DnaJ homolog subfamily B member 6 OS=Homo sapiens OX=9606 GN=DNAJB6 PE=1 SV=2	2	0,636	0,663	0,409	351217,3014	215127,4174	529802,9902
Q14966	ZNF638	Zinc finger protein 638 OS=Homo sapiens OX=9606 GN=ZNF638 PE=1 SV=2	6	0,373	0,85	0,409	2112941,914	508892,9852	2320813,731
Q14137	BOP1	Ribosome biogenesis protein BOP1 OS=Homo sapiens OX=9606 GN=BOP1 PE=1 SV=2	5	0,355	1,145	0,408	2281059,293	802225,1027	2422177,815
O75400	PRPF40A	Pre-mRNA-processing factor 40 homolog A OS=Homo sapiens OX=9606 GN=PRPF40A PE=1 SV=2	10	0,771	0,711	0,407	8173253,201	4720450,507	11892469,09
Q9UFC0	LRWD1	Leucine-rich repeat and WD repeat-containing protein 1 OS=Homo sapiens OX=9606 GN=LRWD1	3	0,58	0,931	0,407	842971,2081	388258,3841	845653,6094
Q9H6K4	OPA3	Optic atrophy 3 protein OS=Homo sapiens OX=9606 GN=OPA3 PE=1 SV=1	2	1,701	0,292	0,406	73667,38233	126542,9775	350345,2602
P83881	RPL36A	Large ribosomal subunit protein eL42 OS=Homo sapiens OX=9606 GN=RPL36A PE=1 SV=2	2	1,429	2,262	0,406	505740,8298	939571,4853	1373114,499
Q96IR2	ZNF845	Zinc finger protein 845 OS=Homo sapiens OX=9606 GN=ZNF845 PE=1 SV=3	2	1,257	0,325	0,406	50114,30285	63012,54257	156632,4426
Q9UQ35	SRRM2	Serine/arginine repetitive matrix protein 2 OS=Homo sapiens OX=9606 GN=SRRM2 PE=1 SV=2	26	0,793	0,551	0,406	18962556,09	11323652,69	34205237,1
Q99595	TIMM17A	Mitochondrial import inner membrane translocase subunit Tim17-A OS=Homo sapiens OX=9606	2	0,612	0,532	0,405	206802,6212	138408,5931	406456,9261
P35637	FUS	RNA-binding protein FUS OS=Homo sapiens OX=9606 GN=FUS PE=1 SV=1	7	0,668	0,986	0,404	55952768,57	31350608,08	69833218,31
Q9HAV7	GRPEL1	GrpE protein homolog 1, mitochondrial OS=Homo sapiens OX=9606 GN=GRPEL1 PE=1 SV=2	7	1,311	0,321	0,403	8003677,423	9472371,346	26868760,92
Q9UII2	ATP5IF1	ATPase inhibitor, mitochondrial OS=Homo sapiens OX=9606 GN=ATP5IF1 PE=1 SV=1	2	1,625	0,422	0,402	4778536,648	7904242,607	13383174,44
O43795	MYO1B	Unconventional myosin-Ib OS=Homo sapiens OX=9606 GN=MYO1B PE=1 SV=3	28	1,004	0,365	0,402	10167420,08	10162096,9	31708563,87
Q9BYV8	CEP41	Centrosomal protein of 41 kDa OS=Homo sapiens OX=9606 GN=CEP41 PE=1 SV=1	2	0,142	2,683	0,401	486894,5122	30294,78017	79880,55995
Q96MU7	YTHDC1	YTH domain-containing protein 1 OS=Homo sapiens OX=9606 GN=YTHDC1 PE=1 SV=3	2	0,759	0,575	0,4	209497,3767	105992,6658	356942,4629
P13726	F3	Tissue factor OS=Homo sapiens OX=9606 GN=F3 PE=1 SV=1	4	1000	0,001	0,397		2302063,746	5509814,362
Q8IWZ8	SUGP1	SURP and G-patch domain-containing protein 1 OS=Homo sapiens OX=9606 GN=SUGP1 PE=1 SV	2	0,905	0,524	0,396	420306,7475	226388,0765	813358,9376
O95208	EPN2	Epsin-2 OS=Homo sapiens OX=9606 GN=EPN2 PE=1 SV=3	2	0,71	0,573	0,396	289916,5039	200194,9593	1163536,185
O00541	PES1	Pescadillo homolog OS=Homo sapiens OX=9606 GN=PES1 PE=1 SV=1	5	0,375	1,245	0,393	3621449,548	1898943,271	3661855,103
Q6P1N0	CC2D1A	Coiled-coil and C2 domain-containing protein 1A OS=Homo sapiens OX=9606 GN=CC2D1A PE=1 S	3	1000	0,001	0,392		266948,5303	1418562,313
Q99959	PKP2	Plakophilin-2 OS=Homo sapiens OX=9606 GN=PKP2 PE=1 SV=2	6	2,194	0,184	0,392	173754,5371	364459,8197	2472507,436
O43823	AKAP8	A-kinase anchor protein 8 OS=Homo sapiens OX=9606 GN=AKAP8 PE=1 SV=1	7	0,985	0,362	0,391	1237230,285	1266627,897	4411905,048
Q9Y2S7	POLDIP2	Polymerase delta-interacting protein 2 OS=Homo sapiens OX=9606 GN=POLDIP2 PE=1 SV=1	7	0,796	0,474	0,391	3683013,171	2855261,914	7531392,188
Q13595	TRA2A	Transformer-2 protein homolog alpha OS=Homo sapiens OX=9606 GN=TRA2A PE=1 SV=1	3	0,692	0,639	0,391	3614446,444	2563828,158	6614909,525
Q13618	CUL3	Cullin-3 OS=Homo sapiens OX=9606 GN=CUL3 PE=1 SV=2	9	0,264	1,828	0,391	2697823,275	129632,4308	1324041,858
P08621	SNRNP70	U1 small nuclear ribonucleoprotein 70 kDa OS=Homo sapiens OX=9606 GN=SNRNP70 PE=1 SV=2	11	0,682	0,586	0,39	18450905,46	11491712,26	31739773,34
P82675	MRPS5	Small ribosomal subunit protein uS5m OS=Homo sapiens OX=9606 GN=MRPS5 PE=1 SV=2	7	0,753	1,056	0,389	1405012,833	3776465,664	1997583,207
Q8IWA0	WDR75	WD repeat-containing protein 75 OS=Homo sapiens OX=9606 GN=WDR75 PE=1 SV=1	3	0,612	0,646	0,389	394264,2732	55001,91804	548177,2823

O14548	COX7A2L	Cytochrome c oxidase subunit 7A-related protein, mitochondrial OS=Homo sapiens OX=9606 GN	3	0,571	0,565	0,389	3735920,079	1793668,384	4751445,972
Q7LBC6	KDM3B	Lysine-specific demethylase 3B OS=Homo sapiens OX=9606 GN=KDM3B PE=1 SV=2	4	0,233	2,096	0,388	2318347,321	454048,5136	2592568,185
Q9UKV3	ACIN1	Apoptotic chromatin condensation inducer in the nucleus OS=Homo sapiens OX=9606 GN=ACIN1	13	0,566	0,699	0,387	8360068,934	4155462,27	11745073,54
P25490	YY1	Transcriptional repressor protein YY1 OS=Homo sapiens OX=9606 GN=YY1 PE=1 SV=2	4	0,556	0,832	0,387	1248006,065	633118,598	1699854,585
Q86YZ3	HRNR	Hornerin OS=Homo sapiens OX=9606 GN=HRNR PE=1 SV=2	2	0,112	3,276	0,387	1360413,013	132793,4562	435104,3597
Q14677	CLINT1	Clathrin interactor 1 OS=Homo sapiens OX=9606 GN=CLINT1 PE=1 SV=1	10	0,919	0,523	0,386	4376743,582	3946843,031	8413134,305
Q9HAB8	PPCS	Phosphopantothenate--cysteine ligase OS=Homo sapiens OX=9606 GN=PPCS PE=1 SV=2	3	0,296	0,898	0,386	544811,6939	500683,8161	1132283,286
Q99661	KIF2C	Kinesin-like protein KIF2C OS=Homo sapiens OX=9606 GN=KIF2C PE=1 SV=2	5	0,228	1,569	0,385	2686974,617	291377,8855	1311198,779
Q5UIP0	RIF1	Telomere-associated protein RIF1 OS=Homo sapiens OX=9606 GN=RIF1 PE=1 SV=2	14	0,649	0,636	0,384	4239827,117	1529642,608	8431852,837
Q15800	MSMO1	Methylsterol monooxygenase 1 OS=Homo sapiens OX=9606 GN=MSMO1 PE=1 SV=1	3	0,247	1,455	0,384	2870806,399	539312,9021	2106535,464
Q16891	IMMT	MICOS complex subunit MIC60 OS=Homo sapiens OX=9606 GN=IMMT PE=1 SV=1	33	0,749	0,502	0,383	41315414,74	40800019,56	103636340,3
Q9UDX5	MTFP1	Mitochondrial fission process protein 1 OS=Homo sapiens OX=9606 GN=MTFP1 PE=1 SV=1	2	0,716	0,529	0,383	975147,3101	681110,4853	1827972,979
Q9NYF8	BCLAF1	Bcl-2-associated transcription factor 1 OS=Homo sapiens OX=9606 GN=BCLAF1 PE=1 SV=2	15	0,604	0,619	0,383	7410190,965	3601606,2	11588566,89
Q14534	SQLE	Squalene monooxygenase OS=Homo sapiens OX=9606 GN=SQLE PE=1 SV=3	6	0,393	1,063	0,382	2325276,036	1213382,208	2191193,966
Q6P1M0	SLC27A4	Long-chain fatty acid transport protein 4 OS=Homo sapiens OX=9606 GN=SLC27A4 PE=1 SV=1	8	1,37	0,322	0,381	1120425,949	1743394,083	4759451,8
O95167	NDUFA3	NADH dehydrogenase [ubiquinone] 1 alpha subcomplex subunit 3 OS=Homo sapiens OX=9606 G	3	1,999	0,187	0,38	237035,0539	473834,8384	1266309,861
Q53F19	NCBP3	Nuclear cap-binding protein subunit 3 OS=Homo sapiens OX=9606 GN=NCBP3 PE=1 SV=2	3	0,444	0,743	0,38	1140004,891	396055,9022	1537432,588
O00244	ATOX1	Copper transport protein ATOX1 OS=Homo sapiens OX=9606 GN=ATOX1 PE=1 SV=1	2	0,527	0,732	0,379	2172885,137	1211025,505	2978205,514
Q13123	IK	Protein Red OS=Homo sapiens OX=9606 GN=IK PE=1 SV=3	7	0,569	0,803	0,378	3721725,241	3168286,49	4862030,592
Q9BRJ6	C7orf50	Uncharacterized protein C7orf50 OS=Homo sapiens OX=9606 GN=C7orf50 PE=1 SV=1	4	0,411	0,986	0,378	4724828,055	1823435,755	5935542,07
O75937	DNAJC8	DnaJ homolog subfamily C member 8 OS=Homo sapiens OX=9606 GN=DNAJC8 PE=1 SV=2	6	0,295	1,491	0,378	6513176,564	2376944,766	4262714,603
Q13084	MRPL28	Large ribosomal subunit protein bL28m OS=Homo sapiens OX=9606 GN=MRPL28 PE=1 SV=4	9	1,374	0,453	0,377	831690,6217	494590,898	2199159,668
O95202	LETM1	Mitochondrial proton/calcium exchanger protein OS=Homo sapiens OX=9606 GN=LETM1 PE=1 S	18	1,288	0,309	0,376	8782290,793	10421495,64	31277934,41
Q96ER9	CCDC51	Mitochondrial potassium channel OS=Homo sapiens OX=9606 GN=CCDC51 PE=1 SV=2	3	0,92	0,345	0,376	213909,2178	196732,0023	637163,2214
Q9NUQ7	UFSP2	Ufm1-specific protease 2 OS=Homo sapiens OX=9606 GN=UFSP2 PE=1 SV=3	2	0,732	0,52	0,376	426816,931	351604,2785	816245,6103
Q05397	PTK2	Focal adhesion kinase 1 OS=Homo sapiens OX=9606 GN=PTK2 PE=1 SV=2	4	0,232	1,641	0,375	1069138,608	249670,7285	559207,8003
Q8N8A6	DDX51	ATP-dependent RNA helicase DDX51 OS=Homo sapiens OX=9606 GN=DDX51 PE=1 SV=3	2	0,423	0,885	0,374	229771,6081	97085,84876	259613,4512
Q6RW13	AGTRAP	Type-1 angiotensin II receptor-associated protein OS=Homo sapiens OX=9606 GN=AGTRAP PE=1	2	0,845	0,441	0,373	886548,2929	749398,3351	3282732,995
Q86YS6	RAB43	Ras-related protein Rab-43 OS=Homo sapiens OX=9606 GN=RAB43 PE=1 SV=1	2	0,58	0,561	0,373	92691,42598	53769,12687	169029,7528
Q9BVJ6	UTP14A	U3 small nucleolar RNA-associated protein 14 homolog A OS=Homo sapiens OX=9606 GN=UTP14	5	0,5	0,693	0,373	1059681,388	595728,9912	1850796,337
Q13523	PRP4K	Serine/threonine-protein kinase PRP4 homolog OS=Homo sapiens OX=9606 GN=PRPF4B PE=1 SV	7	0,982	0,419	0,372	1112419,159	479271,6643	2484659,138
P23368	ME2	NAD-dependent malic enzyme, mitochondrial OS=Homo sapiens OX=9606 GN=ME2 PE=1 SV=1	8	0,802	0,458	0,372	5284261,476	4582480,025	10917080,39
Q9UPN4	CEP131	Centrosomal protein of 131 kDa OS=Homo sapiens OX=9606 GN=CEP131 PE=1 SV=3	2	0,486	0,756	0,372	651554,4845	312073,0424	861929,325
Q15014	MORF4L2	Mortality factor 4-like protein 2 OS=Homo sapiens OX=9606 GN=MORF4L2 PE=1 SV=1	2	0,429	0,83	0,372	960645,1127	477847,8569	1149162,842
Q12873	CHD3	Chromodomain-helicase-DNA-binding protein 3 OS=Homo sapiens OX=9606 GN=CHD3 PE=1 SV=	2	0,878	0,451	0,371	2650376,457	3441090,193	4028922,785
Q9NP81	SARS2	Serine--tRNA ligase, mitochondrial OS=Homo sapiens OX=9606 GN=SARS2 PE=1 SV=1	10	0,683	0,664	0,371	2839323,617	1482610,179	5138055,856
Q9H583	HEATR1	HEAT repeat-containing protein 1 OS=Homo sapiens OX=9606 GN=HEATR1 PE=1 SV=3	15	0,55	0,682	0,371	6521531,612	3979885,191	8580425,449
Q9NR46	SH3GLB2	Endophilin-B2 OS=Homo sapiens OX=9606 GN=SH3GLB2 PE=1 SV=1	5	1,732	0,242	0,37	660812,7683	1095343,297	3939893,39
P53007	SLC25A1	Tricarboxylate transport protein, mitochondrial OS=Homo sapiens OX=9606 GN=SLC25A1 PE=1 S	8	0,706	0,747	0,37	8642045,723	6142912,555	12872904,46
Q9GZT3	SLIRP	SRA stem-loop-interacting RNA-binding protein, mitochondrial OS=Homo sapiens OX=9606 GN=S	7	0,385	0,857	0,369	24573086,73	8813492,002	26049588,29
Q8TCT9	HM13	Minor histocompatibility antigen H13 OS=Homo sapiens OX=9606 GN=HM13 PE=1 SV=1	9	1,315	0,285	0,368	13244051,03	16488248,91	44108420,67
Q92900	UPF1	Regulator of nonsense transcripts 1 OS=Homo sapiens OX=9606 GN=UPF1 PE=1 SV=2	17	0,688	0,618	0,368	11064787,25	6201355,174	17163602,82
Q13740	ALCAM	CD166 antigen OS=Homo sapiens OX=9606 GN=ALCAM PE=1 SV=2	7	4,373	0,095	0,367	1230842,441	8048285,658	21965898,07
Q9BYD2	MRPL9	Large ribosomal subunit protein bL9m OS=Homo sapiens OX=9606 GN=MRPL9 PE=1 SV=2	3	1,267	0,457	0,367	979095,4616	1231924,225	3448962,339
P61604	HSPE1	10 kDa heat shock protein, mitochondrial OS=Homo sapiens OX=9606 GN=HSPE1 PE=1 SV=2	9	0,634	0,574	0,367	115921381	75100193,59	211211949,3
Q8WU90	ZC3H15	Zinc finger CCCH domain-containing protein 15 OS=Homo sapiens OX=9606 GN=ZC3H15 PE=1 SV	4	0,29	1,385	0,367	1681901,755	316068,4177	1168961,111
Q9UFG5	C19orf25	UPF0449 protein C19orf25 OS=Homo sapiens OX=9606 GN=C19orf25 PE=1 SV=2	2	0,651	0,767	0,366	471577,1173	272640,7535	568046,4921
P38432	COIL	Coilin OS=Homo sapiens OX=9606 GN=COIL PE=1 SV=1	3	0,441	0,974	0,364	250591,6439	68814,47012	279001,0469
Q9Y6M1	IGF2BP2	Insulin-like growth factor 2 mRNA-binding protein 2 OS=Homo sapiens OX=9606 GN=IGF2BP2 PE	8	0,266	1,682	0,364	11125609,75	9599384,941	7411650,255
P13647	KRT5	Keratin, type II cytoskeletal 5 OS=Homo sapiens OX=9606 GN=KRT5 PE=1 SV=3	17	2,181	0,192	0,363	17280972,56	44207050,76	127699987,9
Q13823	GNL2	Nucleolar GTP-binding protein 2 OS=Homo sapiens OX=9606 GN=GNL2 PE=1 SV=1	6	0,524	0,627	0,363	1917860,225	534060,5148	3485294,968
P07305	H1-0	Histone H1.0 OS=Homo sapiens OX=9606 GN=H1-0 PE=1 SV=3	2	1000	0,001	0,361		4764220,044	18673309,69
O95453	PARN	Poly(A)-specific ribonuclease PARN OS=Homo sapiens OX=9606 GN=PARN PE=1 SV=1	3	0,993	0,361	0,361	155344,7661	149994,9776	1924281,129
Q8IX01	SUGP2	SURP and G-patch domain-containing protein 2 OS=Homo sapiens OX=9606 GN=SUGP2 PE=1 SV=	3	0,573	0,604	0,361	638385,7332	328603,2163	1293697,667
Q6P1M3	LLGL2	LLGL scribble cell polarity complex component 2 OS=Homo sapiens OX=9606 GN=LLGL2 PE=1 SV=	9			0,36	224764,2584	48873,69713	1841757,912
Q9NX63	CHCHD3	MICOS complex subunit MIC19 OS=Homo sapiens OX=9606 GN=CHCHD3 PE=1 SV=1	5	0,897	0,386	0,36	8710048,4	9514437,086	27168953,77
Q6RFH5	WDR74	WD repeat-containing protein 74 OS=Homo sapiens OX=9606 GN=WDR74 PE=1 SV=1	2	0,723	0,592	0,36	369446,931	75876,74457	650833,9191
P47914	RPL29	Large ribosomal subunit protein eL29 OS=Homo sapiens OX=9606 GN=RPL29 PE=1 SV=2	2	0,451	1,118	0,359	12642294,63	4114590,469	13159638,51
Q56VL3	OCIAD2	OCIA domain-containing protein 2 OS=Homo sapiens OX=9606 GN=OCIAD2 PE=1 SV=1	3	0,341	0,661	0,359	2666196,733	1140366,128	4218560,253
Q6DN03	H2BC20P	Putative histone H2B type 2-C OS=Homo sapiens OX=9606 GN=H2BC20P PE=5 SV=3	3	0,471	0,77	0,358	110648166,7	62650037,52	143179204,8
P48449	LSS	Lanosterol synthase OS=Homo sapiens OX=9606 GN=LSS PE=1 SV=1	14	0,237	1,633	0,358	15907640,48	1472036,312	6511215,602
Q6PI48	DARS2	Aspartate--tRNA ligase, mitochondrial OS=Homo sapiens OX=9606 GN=DARS2 PE=1 SV=1	13	1,518	0,229	0,356	2746820,991	5100668,752	11855582,18
Q13505	MTX1	Metaxin-1 OS=Homo sapiens OX=9606 GN=MTX1 PE=1 SV=3	10	0,88	0,422	0,353	3190997,149	2923225,005	6264124,795
Q86TI2	DPP9	Dipeptidyl peptidase 9 OS=Homo sapiens OX=9606 GN=DPP9 PE=1 SV=3	2			0,352	179338,9058	210500,1211	576223,2225
Q9UI14	RABAC1	Prenylated Rab acceptor protein 1 OS=Homo sapiens OX=9606 GN=RABAC1 PE=1 SV=1	2	1,396	0,258	0,352	659003,4278	1595336,984	2538898,549
Q14684	RRP1B	Ribosomal RNA processing protein 1 homolog B OS=Homo sapiens OX=9606 GN=RRP1B PE=1 SV	5	0,197	1,387	0,352	4015489,196	1498175,296	2850511,294
Q9H788	SH2D4A	SH2 domain-containing protein 4A OS=Homo sapiens OX=9606 GN=SH2D4A PE=1 SV=1	2	0,95	0,29	0,351	106956,5078	355465,9083	700480,0801
P09497	CLTB	Clathrin light chain B OS=Homo sapiens OX=9606 GN=CLTB PE=1 SV=1	6	2,245	0,151	0,35	1438624,015	4751917,218	12294822,76
Q9NXF1	TEX10	Testis-expressed protein 10 OS=Homo sapiens OX=9606 GN=TEX10 PE=1 SV=2	4	0,549	0,632	0,35	619867,7027	113946,3743	912636,8263
O00567	NOP56	Nucleolar protein 56 OS=Homo sapiens OX=9606 GN=NOP56 PE=1 SV=4	15	0,632	0,568	0,349	7872300,814	4174182,777	14077925,66
Q9BVI4	NOC4L	Nucleolar complex protein 4 homolog OS=Homo sapiens OX=9606 GN=NOC4L PE=1 SV=1	3	0,534	0,803	0,349	1359536,779	684361,0756	1993096,996
Q13243	SRSF5	Serine/arginine-rich splicing factor 5 OS=Homo sapiens OX=9606 GN=SRSF5 PE=1 SV=1	5	0,494	0,739	0,348	5311059,537	1928860,466	7978222,328
Q9UPN9	TRIM33	E3 ubiquitin-protein ligase TRIM33 OS=Homo sapiens OX=9606 GN=TRIM33 PE=1 SV=3	2	0,281	2,192	0,348	202759,327	499775,2036	1525899,179
Q9ULW0	TPX2	Targeting protein for Xklp2 OS=Homo sapiens OX=9606 GN=TPX2 PE=1 SV=2	9	0,592	0,609	0,347	4600641,537	2342660,899	7461548,98
O00515	LAD1	Ladinin-1 OS=Homo sapiens OX=9606 GN=LAD1 PE=1 SV=2	17	1,679	0,201	0,345	370866,1891	769397,6083	13633109,36
Q9BZE4	GTPBP4	GTP-binding protein 4 OS=Homo sapiens OX=9606 GN=GTPBP4 PE=1 SV=3	13	0,557	0,61	0,345	5362637,38	3004490,326	8541858,583
P62318	SNRPD3	Small nuclear ribonucleoprotein Sm D3 OS=Homo sapiens OX=9606 GN=SNRPD3 PE=1 SV=1	2	0,37	0,916	0,345	23464874,83	10322510,09	25039403,25
Q9Y2X3	NOP58	Nucleolar protein 58 OS=Homo sapiens OX=9606 GN=NOP58 PE=1 SV=1	12	0,594	0,615	0,343	5469124,154	3009067,066	9901423,787
Q07955	SRSF1	Serine/arginine-rich splicing factor 1 OS=Homo sapiens OX=9606 GN=SRSF1 PE=1 SV=2	11	0,666	0,572	0,342	32543694,23	20258928,98	56360923,13
P18858	LIG1	DNA ligase 1 OS=Homo sapiens OX=9606 GN=LIG1 PE=1 SV=1	5	0,351	0,533	0,342	907475,2276	204860,7152	1971780,968
Q9Y5J7	TIMM9	Mitochondrial import inner membrane translocase subunit Tim9 OS=Homo sapiens OX=9606 GN	3	1,078	0,342	0,341	1363870,52	1357516,671	4044937,57
P13051	UNG	Uracil-DNA glycosylase OS=Homo sapiens OX=9606 GN=UNG PE=1 SV=2	2	0,79	0,43	0,341	577813,684	459511,4025	1244424,656
Q99640	PKMYT1	Membrane-associated tyrosine- and threonine-specific cdc2-inhibitory kinase OS=Homo sapiens	4	2,456	0,092	0,34	81545,00328	233380,368	1190596,552
Q9H089	LSG1	Large subunit GTPase 1 homolog OS=Homo sapiens OX=9606 GN=LSG1 PE=1 SV=2	3	0,724	0,451	0,34	157319,6703	113374,9513	552168,1516
O15498	YKT6	Synaptobrevin homolog YKT6 OS=Homo sapiens OX=9606 GN=YKT6 PE=1 SV=1	5	0,605	1,395	0,34	3335105,06	949536,9173	2978877,38
P06899	H2BC11	Histone H2B type 1-J OS=Homo sapiens OX=9606 GN=H2BC11 PE=1 SV=3	2	0,456	0,757	0,34	107004567,4	58552325,36	140649274,6
Q14244	MAP7	Ensconsin OS=Homo sapiens OX=9606 GN=MAP7 PE=1 SV=1	4	1000	0,001	0,339		218157,039	600781,1651
P10809	HSPD1	60 kDa heat shock protein, mitochondrial OS=Homo sapiens OX=9606 GN=HSPD1 PE=1 SV=2	38	0,456	0,948	0,339	402171966,7	170378697,8	582319217,3
Q9BQ52	ELAC2	Zinc phosphodiesterase ELAC protein 2 OS=Homo sapiens OX=9606 GN=ELAC2 PE=1 SV=2	3	0,413	0,795	0,339	1250522,835	619350,9562	1554029,641
Q9Y295	DRG1	Developmentally-regulated GTP-binding protein 1 OS=Homo sapiens OX=9606 GN=DRG1 PE=1 S	4	0,211	1,425	0,339	4817522,367	832088,0105	3295447,061
Q9Y653	ADGRG1	Adhesion G-protein coupled receptor G1 OS=Homo sapiens OX=9606 GN=ADGRG1 PE=1 SV=2	2	1000	0,001	0,338		45424,36368	277351,1183
Q969X6	UTP4	U3 small nucleolar RNA-associated protein 4 homolog OS=Homo sapiens OX=9606 GN=UTP4 PE=	4	0,465	0,592	0,338	575433,0596	544739,9321	1522497,239
Q9Y2R4	DDX52	Probable ATP-dependent RNA helicase DDX52 OS=Homo sapiens OX=9606 GN=DDX52 PE=1 SV=4	2	0,276	0,971	0,337	534618,1976	96539,79803	578554,0329
Q8NEJ9	NGDN	Neuroguidin OS=Homo sapiens OX=9606 GN=NGDN PE=1 SV=1	4	0,627	0,575	0,336	628901,6398	323845,1059	1197554,405
P68431	H3C1	Histone H3.1 OS=Homo sapiens OX=9606 GN=H3C1 PE=1 SV=2	10	0,464	0,928	0,333	1342619861	671254869,9	1414963033
Q96C19	EFHD2	EF-hand domain-containing protein D2 OS=Homo sapiens OX=9606 GN=EFHD2 PE=1 SV=1	6	1,216	0,265	0,332	5116719,56	5933567,074	18460671,57
Q6PD62	CTR9	RNA polymerase-associated protein CTR9 homolog OS=Homo sapiens OX=9606 GN=CTR9 PE=1 S	3	0,254	0,806	0,332	343979,362	31332,06737	479626,9764
Q9H0H5	RACGAP1	Rac GTPase-activating protein 1 OS=Homo sapiens OX=9606 GN=RACGAP1 PE=1 SV=1	5	1,018	0,345	0,331	1185587,036	1078058,267	3509587,566
O15446	POLR1G	DNA-directed RNA polymerase I subunit RPA34 OS=Homo sapiens OX=9606 GN=POLR1G PE=1 SV	2	0,491	0,663	0,331	277581,2572	136185,6431	538856,3985
P09622	DLD	Dihydrolipoyl dehydrogenase, mitochondrial OS=Homo sapiens OX=9606 GN=DLD PE=1 SV=2	11	0,826	0,595	0,329	23399768,2	19356545,19	46518527,94
P49711	CTCF	Transcriptional repressor CTCF OS=Homo sapiens OX=9606 GN=CTCF PE=1 SV=1	2	0,071	2,964	0,329	1264014,208	39434,21938	349193,8895
Q9UFN0	NIPSNAP3A	Protein NipSnap homolog 3A OS=Homo sapiens OX=9606 GN=NIPSNAP3A PE=1 SV=2	3	1000	0,001	0,328		642844,8397	2159580,759
O43819	SCO2	Protein SCO2 homolog, mitochondrial OS=Homo sapiens OX=9606 GN=SCO2 PE=1 SV=3	3	0,26	1,446	0,328	1152374,779	455108,1199	1379770,99
Q9Y2W1	THRAP3	Thyroid hormone receptor-associated protein 3 OS=Homo sapiens OX=9606 GN=THRAP3 PE=1 S	14	0,556	0,636	0,327	13147434,75	5910576,77	18113302,94
P37268	FDFT1	Squalene synthase OS=Homo sapiens OX=9606 GN=FDFT1 PE=1 SV=1	15	0,106	3,622	0,326	26006159,84	3030791,068	8001585,782
Q9HB09	BCL2L12	Bcl-2-like protein 12 OS=Homo sapiens OX=9606 GN=BCL2L12 PE=1 SV=1	2	1,593	0,276	0,325	146324,5403	343939,7972	1117708,582
Q96D05	FAM241B	Protein FAM241B OS=Homo sapiens OX=9606 GN=FAM241B PE=1 SV=1	2	0,616	0,513	0,325	633608,1867	390503,3564	1607943,84
Q13268	DHRS2	Dehydrogenase/reductase SDR family member 2, mitochondrial OS=Homo sapiens OX=9606 GN=	9	1,837	0,569	0,324	491798,636	833069,4652	1461061,03
Q9H330	TMEM245	Transmembrane protein 245 OS=Homo sapiens OX=9606 GN=TMEM245 PE=1 SV=3	3	1000	0,001	0,323		334808,7738	1276350,796
Q9H307	PNN	Pinin OS=Homo sapiens OX=9606 GN=PNN PE=1 SV=5	11	0,564	0,558	0,321	8703770,407	4292946,047	15974340,71
P35251	RFC1	Replication factor C subunit 1 OS=Homo sapiens OX=9606 GN=RFC1 PE=1 SV=4	3	0,589	0,539	0,32	741201,6935	416993,5297	1325694,894

Q14690	PDCD11	Protein RRP5 homolog OS=Homo sapiens OX=9606 GN=PDCD11 PE=1 SV=3	10	0,362	0,788	0,32	2311625,428	545659,4261	2933629,553
Q16637	SMN1	Survival motor neuron protein OS=Homo sapiens OX=9606 GN=SMN1 PE=1 SV=1	3	0,475	1,018	0,319	960195,6077	393025,3853	698674,1977
Q92747	ARPC1A	Actin-related protein 2/3 complex subunit 1A OS=Homo sapiens OX=9606 GN=ARPC1A PE=1 SV=	7	0,462	0,705	0,319	3697417,058	1650594,681	8774301,03
P39748	FEN1	Flap endonuclease 1 OS=Homo sapiens OX=9606 GN=FEN1 PE=1 SV=1	8	0,394	0,853	0,319	14917732,54	7208341,469	18674652,32
P17275	JUNB	Transcription factor JunB OS=Homo sapiens OX=9606 GN=JUNB PE=1 SV=1	5	2,101	0,115	0,318	51272,14924	1109554,765	3525987,184
Q03701	CEBPZ	CCAAT/enhancer-binding protein zeta OS=Homo sapiens OX=9606 GN=CEBPZ PE=1 SV=3	6	0,357	0,989	0,318	970084,7398	223402,9623	1222628,543
Q9BQG0	MYBBP1A	Myb-binding protein 1A OS=Homo sapiens OX=9606 GN=MYBBP1A PE=1 SV=2	23	0,476	0,646	0,317	12953613,86	5561965,721	20606973,44
Q9BXW7	HDHD5	Haloacid dehalogenase-like hydrolase domain-containing 5 OS=Homo sapiens OX=9606 GN=HDH	9	0,425	1,029	0,317	5063106,768	2038025,201	6526528,246
Q8NBJ4	GOLM1	Golgi membrane protein 1 OS=Homo sapiens OX=9606 GN=GOLM1 PE=1 SV=1	3	0,495	0,529	0,316	824884,0452	488688,3394	2211152,384
O75144	ICOSLG	ICOS ligand OS=Homo sapiens OX=9606 GN=ICOSLG PE=1 SV=2	2	1000	0,001	0,315		41214,61612	246121,7796
Q9NYY8	FASTKD2	FAST kinase domain-containing protein 2, mitochondrial OS=Homo sapiens OX=9606 GN=FASTKD	2	0,651	0,492	0,315	440975,1487	284691,5619	912133,0707
Q7KZI7	MARK2	Serine/threonine-protein kinase MARK2 OS=Homo sapiens OX=9606 GN=MARK2 PE=1 SV=2	3	0,724	0,498	0,313	619259,3396	340082,0062	1193510,855
Q9NV31	IMP3	U3 small nucleolar ribonucleoprotein protein IMP3 OS=Homo sapiens OX=9606 GN=IMP3 PE=1 S	3	0,505	0,679	0,313	1049524,653	485115,2555	1516480,428
Q00796	SORD	Sorbitol dehydrogenase OS=Homo sapiens OX=9606 GN=SORD PE=1 SV=4	9	0,259	1,458	0,312	6995277,664	1745236,004	4803772,642
Q8IZP0	ABI1	Abl interactor 1 OS=Homo sapiens OX=9606 GN=ABI1 PE=1 SV=4	2	1,666	0,222	0,31	681361,2304	596802,5916	1655040,598
P14923	JUP	Junction plakoglobin OS=Homo sapiens OX=9606 GN=JUP PE=1 SV=3	20	1,971	0,159	0,308	7215366,655	14534506,04	42856408,63
A0A2R8YDH4	ZO-2	Tight junction protein ZO-2 OS=Homo sapiens OX=9606 PE=1 SV=1	8	0,815	0,275	0,307	584353,9382	498758,1785	1855271,82
Q9GZY8	MFF	Mitochondrial fission factor OS=Homo sapiens OX=9606 GN=MFF PE=1 SV=1	3	0,803	0,593	0,306	2338691,516	1226711,917	4345072,54
P61313	RPL15	Large ribosomal subunit protein eL15 OS=Homo sapiens OX=9606 GN=RPL15 PE=1 SV=2	8	0,575	0,862	0,306	32441974,65	19388490,07	35652089,04
O95833	CLIC3	Chloride intracellular channel protein 3 OS=Homo sapiens OX=9606 GN=CLIC3 PE=1 SV=2	5	1000	0,001	0,305		1420225,385	6483545,847
Q9UKS6	PACSIN3	Protein kinase C and casein kinase substrate in neurons protein 3 OS=Homo sapiens OX=9606 GI	6	1,426	0,221	0,305	954025,8206	1424959,48	4053000,67
Q9NWH9	SLTM	SAFB-like transcription modulator OS=Homo sapiens OX=9606 GN=SLTM PE=1 SV=2	5	0,431	0,575	0,305	725131,2283	370069,6	1298400,01
Q9HB58	SP110	Sp110 nuclear body protein OS=Homo sapiens OX=9606 GN=SP110 PE=1 SV=5	2	1000	0,001	0,304		152405,0348	1360484,614
Q9BUP0	EFHD1	EF-hand domain-containing protein D1 OS=Homo sapiens OX=9606 GN=EFHD1 PE=1 SV=1	3	0,843	0,398	0,304	1242682,481	947428,1742	3292428,977
O43278	SPINT1	Kunitz-type protease inhibitor 1 OS=Homo sapiens OX=9606 GN=SPINT1 PE=1 SV=2	8	2,596	0,025	0,303	22752,10719	2185317,972	7010260,846
P06748	NPM1	Nucleophosmin OS=Homo sapiens OX=9606 GN=NPM1 PE=1 SV=2	9	0,371	0,854	0,302	424946902,8	139010979,6	492378607,7
Q5T280	SPOUT1	Putative methyltransferase C9orf114 OS=Homo sapiens OX=9606 GN=SPOUT1 PE=1 SV=3	2	0,727	0,432	0,301	710978,9158	504855,8683	1680938,254
Q8N1F8	STK11IP	Serine/threonine-protein kinase 11-interacting protein OS=Homo sapiens OX=9606 GN=STK11IP	2	1000	0,001	0,299		11206,46351	37535,88483
Q86XP3	DDX42	ATP-dependent RNA helicase DDX42 OS=Homo sapiens OX=9606 GN=DDX42 PE=1 SV=1	18	0,508	0,618	0,297	8059391,384	3305045,786	12637887,05
Q9NX58	LYAR	Cell growth-regulating nucleolar protein OS=Homo sapiens OX=9606 GN=LYAR PE=1 SV=3	4	0,477	0,61	0,297	1230660,665	512206,4095	2336683,523
Q5T9A4	ATAD3B	ATPase family AAA domain-containing protein 3B OS=Homo sapiens OX=9606 GN=ATAD3B PE=1	5	0,678	0,475	0,295	4699275,053	3690011,606	8364907,944
Q14146	URB2	Unhealthy ribosome biogenesis protein 2 homolog OS=Homo sapiens OX=9606 GN=URB2 PE=1 S	3	0,589	0,425	0,293	420592,3269	346463,787	1230325,089
Q8N3F8	MICALL1	MICAL-like protein 1 OS=Homo sapiens OX=9606 GN=MICALL1 PE=1 SV=2	3	0,779	0,555	0,292	181137,9461	18603,51676	660971,3897
Q96AQ8	MCUR1	Mitochondrial calcium uniporter regulator 1 OS=Homo sapiens OX=9606 GN=MCUR1 PE=1 SV=1	2	1,057	0,285	0,289	439600,0479	466538,5157	1584081,25
Q15154	PCM1	Pericentriolar material 1 protein OS=Homo sapiens OX=9606 GN=PCM1 PE=1 SV=6	7	0,957	0,995	0,289	2396812,684	4153957,324	2612526,461
Q7Z4W1	DCXR	L-xylulose reductase OS=Homo sapiens OX=9606 GN=DCXR PE=1 SV=2	5	0,933	0,177	0,289	663309,4459	513992,8576	2321045,92
Q9NYH9	UTP6	U3 small nucleolar RNA-associated protein 6 homolog OS=Homo sapiens OX=9606 GN=UTP6 PE=	2	0,436	0,453	0,288	424903,7777	92894,80406	773882,622
Q9UBR2	CTSZ	Cathepsin Z OS=Homo sapiens OX=9606 GN=CTSZ PE=1 SV=1	3	6,601	0,048	0,287	280857,5544	2712425,263	17355655,71
P18583	SON	Protein SON OS=Homo sapiens OX=9606 GN=SON PE=1 SV=4	12	0,5	0,612	0,287	6119416,095	2426701,654	8530451,979
P19338	NCL	Nucleolin OS=Homo sapiens OX=9606 GN=NCL PE=1 SV=3	24	0,315	0,966	0,287	227702695,6	70086825,13	256741408,9
Q9NUT2	ABCB8	Mitochondrial potassium channel ATP-binding subunit OS=Homo sapiens OX=9606 GN=ABCB8 P	2	1000	0,001	0,284		160088,1948	754619,5367
Q8N983	MRPL43	Large ribosomal subunit protein mL43 OS=Homo sapiens OX=9606 GN=MRPL43 PE=1 SV=1	4	0,484	0,774	0,284	718383,9704	292750,7933	883123,2163
Q969Y2	GTPBP3	tRNA modification GTPase GTPBP3, mitochondrial OS=Homo sapiens OX=9606 GN=GTPBP3 PE=1	3	0,956	0,305	0,283	246465,9997	273326,5129	856188,8595
Q86VM9	ZC3H18	Zinc finger CCCH domain-containing protein 18 OS=Homo sapiens OX=9606 GN=ZC3H18 PE=1 SV	5	0,799	0,626	0,283	1113985,792	770126,8359	2371678,729
Q14192	FHL2	Four and a half LIM domains protein 2 OS=Homo sapiens OX=9606 GN=FHL2 PE=1 SV=3	3	2,648	0,137	0,282	536989,9433	1561467,851	4879259,771
P53350	PLK1	Serine/threonine-protein kinase PLK1 OS=Homo sapiens OX=9606 GN=PLK1 PE=1 SV=1	2	0,798	0,366	0,28	408251,3063	327795,2691	1131378,347
Q9BQP7	MGME1	Mitochondrial genome maintenance exonuclease 1 OS=Homo sapiens OX=9606 GN=MGME1 PE=	5	0,632	0,422	0,28	1509044,61	901004,6152	3551042,123
Q86WA6	BPHL	Valacyclovir hydrolase OS=Homo sapiens OX=9606 GN=BPHL PE=1 SV=1	4		0,362	0,278	376120,839	45704,61305	1347714,946
Q9P0V3	SH3BP4	SH3 domain-binding protein 4 OS=Homo sapiens OX=9606 GN=SH3BP4 PE=1 SV=1	2	0,935	0,297	0,278	162811,5151	152246,9599	548064,3159
Q8WXX5	DNAJC9	DnaJ homolog subfamily C member 9 OS=Homo sapiens OX=9606 GN=DNAJC9 PE=1 SV=1	8	0,728	0,399	0,278	6891184,654	4835741,264	16955656,8
Q9Y399	MRPS2	Small ribosomal subunit protein uS2m OS=Homo sapiens OX=9606 GN=MRPS2 PE=1 SV=1	5	1,191	0,578	0,277	3784014,76	4756296,876	7497889,103
Q8NBT2	SPC24	Kinetochore protein Spc24 OS=Homo sapiens OX=9606 GN=SPC24 PE=1 SV=3	3	0,508	0,893	0,277	1158177,632	515875,0354	468155,1812
P46782	RPS5	Small ribosomal subunit protein uS7 OS=Homo sapiens OX=9606 GN=RPS5 PE=1 SV=4	5	0,42	0,62	0,277	7669114,7	3225507,158	13786378,43
P52566	ARHGDIB	Rho GDP-dissociation inhibitor 2 OS=Homo sapiens OX=9606 GN=ARHGDIB PE=1 SV=3	6	0,655	0,152	0,275	736347,1811	1062360,541	4412851,475
Q9NVP1	DDX18	ATP-dependent RNA helicase DDX18 OS=Homo sapiens OX=9606 GN=DDX18 PE=1 SV=2	9	0,296	0,89	0,275	3505466,635	988946,3447	4667663,35
Q9H1C4	UNC93B1	Protein unc-93 homolog B1 OS=Homo sapiens OX=9606 GN=UNC93B1 PE=1 SV=2	3	8,391	0,022	0,274	6675,133132	531631,5298	1276999,275
Q8TEQ6	GEMIN5	Gem-associated protein 5 OS=Homo sapiens OX=9606 GN=GEMIN5 PE=1 SV=3	7	0,333	0,752	0,273	3905739,688	1507845,993	4546553,399
P16401	H1-5	Histone H1.5 OS=Homo sapiens OX=9606 GN=H1-5 PE=1 SV=3	9	0,303	0,872	0,273	333974892,9	119675559,4	372919385,7
Q9GZS1	POLR1E	DNA-directed RNA polymerase I subunit RPA49 OS=Homo sapiens OX=9606 GN=POLR1E PE=1 SV	2	1,239	0,292	0,272	294742,8617	296902,0231	1124541,095
Q9UM00	TMCO1	Calcium load-activated calcium channel OS=Homo sapiens OX=9606 GN=TMCO1 PE=1 SV=2	2	2,204	0,396	0,269	1807531,319	643105,4767	3390622,444
Q7Z434	MAVS	Mitochondrial antiviral-signaling protein OS=Homo sapiens OX=9606 GN=MAVS PE=1 SV=2	9	1,29	0,229	0,269	4388198,948	3814529,248	21527195,06
P43362	MAGEA9	Melanoma-associated antigen 9 OS=Homo sapiens OX=9606 GN=MAGEA9 PE=1 SV=1	3	0,803	0,332	0,269	66006,43054	47423,19387	198917,6964
Q6PL18	ATAD2	ATPase family AAA domain-containing protein 2 OS=Homo sapiens OX=9606 GN=ATAD2 PE=1 SV	3	0,731	0,328	0,269	317459,606	401606,6123	975407,9935
Q99569	PKP4	Plakophilin-4 OS=Homo sapiens OX=9606 GN=PKP4 PE=1 SV=2	2	0,979	0,446	0,268	310589,3964	28913,37838	640972,4172
Q8NFV4	ABHD11	sn-1-specific diacylglycerol lipase ABHD11 OS=Homo sapiens OX=9606 GN=ABHD11 PE=1 SV=2	5	1,457	0,148	0,266	410010,0051	686722,8457	2681613,203
O00148	DDX39A	ATP-dependent RNA helicase DDX39A OS=Homo sapiens OX=9606 GN=DDX39A PE=1 SV=2	5	0,676	0,42	0,266	2578092,327	1513936,007	5515777,043
Q02952	AKAP12	A-kinase anchor protein 12 OS=Homo sapiens OX=9606 GN=AKAP12 PE=1 SV=4	14	0,143	1,942	0,265	11690138,05	1335161,756	5394271,272
Q6WKZ4	RAB11FIP1	Rab11 family-interacting protein 1 OS=Homo sapiens OX=9606 GN=RAB11FIP1 PE=1 SV=3	4	0,606	0,522	0,263	2431065,77	1048217,645	3945857,047
P11388	TOP2A	DNA topoisomerase 2-alpha OS=Homo sapiens OX=9606 GN=TOP2A PE=1 SV=3	28	0,317	0,756	0,263	33630077,18	11155986,4	40613407,08
P36952	SERPINB5	Serpin B5 OS=Homo sapiens OX=9606 GN=SERPINB5 PE=1 SV=2	9	2,286	0,083	0,262	312466,3624	4429045,681	18441811,63
P55327	TPD52	Tumor protein D52 OS=Homo sapiens OX=9606 GN=TPD52 PE=1 SV=2	6	0,776	0,316	0,261	4796938,52	3823653,139	13165532,91
Q8TDD1	DDX54	ATP-dependent RNA helicase DDX54 OS=Homo sapiens OX=9606 GN=DDX54 PE=1 SV=2	4	0,329	1,04	0,259	991768,1956	324310,7298	1146752,15
O75521	ECI2	Enoyl-CoA delta isomerase 2 OS=Homo sapiens OX=9606 GN=ECI2 PE=1 SV=4	5	0,747	0,316	0,257	1149263,61	730923,0942	5763729,945
O43818	RRP9	U3 small nucleolar RNA-interacting protein 2 OS=Homo sapiens OX=9606 GN=RRP9 PE=1 SV=1	6	0,547	0,438	0,257	2495803,583	743254,6787	4549155,739
E9PDI4	LAD1	Ladinin-1 OS=Homo sapiens OX=9606 GN=LAD1 PE=1 SV=1	9	1,252	0,188	0,256	51974938,43	19803004,6	34294194,55
O94832	MYO1D	Unconventional myosin-Id OS=Homo sapiens OX=9606 GN=MYO1D PE=1 SV=2	20	0,765	0,283	0,256	2200860,348	449816,1628	11779421,88
P28072	PSMB6	Proteasome subunit beta type-6 OS=Homo sapiens OX=9606 GN=PSMB6 PE=1 SV=4	3	0,123	1,632	0,254	14004888,28	3379450,042	9428437,315
Q8NBN7	RDH13	Retinol dehydrogenase 13 OS=Homo sapiens OX=9606 GN=RDH13 PE=1 SV=2	5	0,721	0,279	0,253	975638,9183	811585,1534	3933145,963
P35749	MYH11	Myosin-11 OS=Homo sapiens OX=9606 GN=MYH11 PE=1 SV=3	4	0,086	4,561	0,253	5019557,281	332295,9634	1309643,514
Q96EY8	MMAB	Corrinoid adenosyltransferase MMAB OS=Homo sapiens OX=9606 GN=MMAB PE=1 SV=1	4	0,42	0,685	0,252	1179121,327	322729,6637	1827379,421
Q15223	NECTIN1	Nectin-1 OS=Homo sapiens OX=9606 GN=NECTIN1 PE=1 SV=3	7	0,24	0,695	0,252	903780,0338	175612,8272	1364253,943
O75094	SLIT3	Slit homolog 3 protein OS=Homo sapiens OX=9606 GN=SLIT3 PE=2 SV=3	3	2,072	0,108	0,251	109481,4228	141568,6241	537801,9846
P49790	NUP153	Nuclear pore complex protein Nup153 OS=Homo sapiens OX=9606 GN=NUP153 PE=1 SV=2	20	1,167	0,213	0,251	5355800,897	6061388,281	28561885,38
Q16625	OCLN	Occludin OS=Homo sapiens OX=9606 GN=OCLN PE=1 SV=1	5	1,115	0,224	0,25	353366,3808	380644,163	5091796,548
Q96LJ7	DHRS1	Dehydrogenase/reductase SDR family member 1 OS=Homo sapiens OX=9606 GN=DHRS1 PE=1 SV	4	1,818	0,16	0,248	483809,7933	1129507,593	6211000,648
Q96GD4	AURKB	Aurora kinase B OS=Homo sapiens OX=9606 GN=AURKB PE=1 SV=3	2	0,285	1,225	0,248	945007,6475	157793,8528	907475,1453
Q16787	LAMA3	Laminin subunit alpha-3 OS=Homo sapiens OX=9606 GN=LAMA3 PE=1 SV=3	14	1,939	0,211	0,247	1973216,035	3179458,234	13610607,63
P78310	CXADR	Coxsackievirus and adenovirus receptor OS=Homo sapiens OX=9606 GN=CXADR PE=1 SV=1	7	0,376	4,341	0,247	3073659,669	146403,5608	1041090,284
Q5BJF2	TMEM97	Sigma intracellular receptor 2 OS=Homo sapiens OX=9606 GN=TMEM97 PE=1 SV=1	2	0,215	1,377	0,244	4573245,42	1005317,828	3177902,871
P16144	ITGB4	Integrin beta-4 OS=Homo sapiens OX=9606 GN=ITGB4 PE=1 SV=5	41	1,482	0,214	0,242	2420326,69	8363459,232	43934958,58
Q969L2	MAL2	Protein MAL2 OS=Homo sapiens OX=9606 GN=MAL2 PE=1 SV=1	2	21,91	0,01	0,238	89711,79892	1899306,758	8774915,844
O14787	TNPO2	Transportin-2 OS=Homo sapiens OX=9606 GN=TNPO2 PE=1 SV=3	5	1,095	0,286	0,236	841314,259	494029,2627	2565287,17
P48651	PTDSS1	Phosphatidylserine synthase 1 OS=Homo sapiens OX=9606 GN=PTDSS1 PE=1 SV=1	2	0,438	0,639	0,233	426208,55	52387,15009	614507,591
Q9NRX1	PNO1	RNA-binding protein PNO1 OS=Homo sapiens OX=9606 GN=PNO1 PE=1 SV=1	3	0,486	0,782	0,23	580126,6585	292600,4888	1031335,659
Q8IYB3	SRRM1	Serine/arginine repetitive matrix protein 1 OS=Homo sapiens OX=9606 GN=SRRM1 PE=1 SV=2	7	0,437	0,743	0,23	4449270,72	1153599,63	6574644,268
Q13112	CHAF1B	Chromatin assembly factor 1 subunit B OS=Homo sapiens OX=9606 GN=CHAF1B PE=1 SV=1	3	0,149	1,561	0,23	1507001,847	88991,97097	938744,4534
P25685	DNAJB1	DnaJ homolog subfamily B member 1 OS=Homo sapiens OX=9606 GN=DNAJB1 PE=1 SV=4	8	1,037	0,306	0,229	3667711,317	3204867,886	13020546,01
P18887	XRCC1	DNA repair protein XRCC1 OS=Homo sapiens OX=9606 GN=XRCC1 PE=1 SV=3	2	0,212	1,068	0,227	743119,6471	157788,5049	695617,1692
O15254	ACOX3	Peroxisomal acyl-coenzyme A oxidase 3 OS=Homo sapiens OX=9606 GN=ACOX3 PE=1 SV=2	6	0,999	0,263	0,225	147210,7899	284507,2019	1578769,592
Q16647	PTGIS	Prostacyclin synthase OS=Homo sapiens OX=9606 GN=PTGIS PE=1 SV=1	12	0,545	0,037	0,224	498354,0519	389107,7407	42560990,14
Q05639	EEF1A2	Elongation factor 1-alpha 2 OS=Homo sapiens OX=9606 GN=EEF1A2 PE=1 SV=1	6	0,654	0,578	0,222	457508291,6	242644641,2	289617298,3
P42766	RPL35	Large ribosomal subunit protein uL29 OS=Homo sapiens OX=9606 GN=RPL35 PE=1 SV=2	4	0,376	0,672	0,222	13311734,55	5682717,674	17910891,34
Q9Y5Y6	ST14	Suppressor of tumorigenicity 14 protein OS=Homo sapiens OX=9606 GN=ST14 PE=1 SV=2	4	1000	0,001	0,22		388716,2593	3323637,441
Q9Y289	SLC5A6	Sodium-dependent multivitamin transporter OS=Homo sapiens OX=9606 GN=SLC5A6 PE=1 SV=2	2	0,443	0,524	0,22	568583,3936	156117,4851	1037002,669
Q92805	GOLGA1	Golgin subfamily A member 1 OS=Homo sapiens OX=9606 GN=GOLGA1 PE=1 SV=3	4	1000	0,001	0,218		379247,6986	1602675,827
Q6JQN1	ACAD10	Acyl-CoA dehydrogenase family member 10 OS=Homo sapiens OX=9606 GN=ACAD10 PE=1 SV=1	2	0,517	0,432	0,218	56060,77784	258918,9657	129715,2446
Q9UIS9	MBD1	Methyl-CpG-binding domain protein 1 OS=Homo sapiens OX=9606 GN=MBD1 PE=1 SV=2	2		0,359	0,217	89160,87544	57562,57125	501055,7892
P12235	SLC25A4	ADP/ATP translocase 1 OS=Homo sapiens OX=9606 GN=SLC25A4 PE=1 SV=4	4	0,861	0,362	0,216	3307537,002	2681991,671	12486620,62
Q9BY89	KIAA1671	Uncharacterized protein KIAA1671 OS=Homo sapiens OX=9606 GN=KIAA1671 PE=1 SV=2	4	1000	0,001	0,215		116648,8986	1294008,863
O14495	PLPP3	Phospholipid phosphatase 3 OS=Homo sapiens OX=9606 GN=PLPP3 PE=1 SV=1	2	0,138	3,57	0,215	565083,8701	48909,70736	279694,3105
Q96KQ7	EHMT2	Histone-lysine N-methyltransferase EHMT2 OS=Homo sapiens OX=9606 GN=EHMT2 PE=1 SV=3	2	0,111	1,731	0,215	252250,0514	28077,5624	145736,3076

Q6IQ23	PLEKHA7	Pleckstrin homology domain-containing family A member 7 OS=Homo sapiens OX=9606 GN=PLE	3	1000	0,001	0,213		77160,82989	282143,3008
P51649	ALDH5A1	Succinate-semialdehyde dehydrogenase, mitochondrial OS=Homo sapiens OX=9606 GN=ALDH5A	6	0,485	0,614	0,213	1547815,986	154948,5868	3097565,883
Q96HY7	DHTKD1	2-oxoadipate dehydrogenase complex component E1 OS=Homo sapiens OX=9606 GN=DHTKD1 P	7	0,799	0,268	0,21	954201,6779	297941,2223	5101888,523
Q14919	DRAP1	Dr1-associated corepressor OS=Homo sapiens OX=9606 GN=DRAP1 PE=1 SV=3	2	0,107	1,893	0,205	1839613,668	254957,4021	918394,772
Q8WXI7	MUC16	Mucin-16 OS=Homo sapiens OX=9606 GN=MUC16 PE=1 SV=3	2	1000	0,001	0,204		198266,2883	950421,4878
Q9UHQ9	CYB5R1	NADH-cytochrome b5 reductase 1 OS=Homo sapiens OX=9606 GN=CYB5R1 PE=1 SV=1	8	2,471	0,07	0,204	1554503,3	3915691,153	25624248,4
P09668	CTSH	Pro-cathepsin H OS=Homo sapiens OX=9606 GN=CTSH PE=1 SV=4	7	1,486	0,092	0,204	202234,5494	451779,4516	3058193,822
Q9H8H0	NOL11	Nucleolar protein 11 OS=Homo sapiens OX=9606 GN=NOL11 PE=1 SV=1	3	0,54	0,557	0,204	636554,3032	144537,5105	1137678,582
P15311	EZR	Ezrin OS=Homo sapiens OX=9606 GN=EZR PE=1 SV=4	12	0,348	0,548	0,204	28442149,72	10769491,94	51582124,06
P46013	MKI67	Proliferation marker protein Ki-67 OS=Homo sapiens OX=9606 GN=MKI67 PE=1 SV=2	84	0,644	0,311	0,201	55314694,23	34640720,67	184740550,7
P52594	AGFG1	Arf-GAP domain and FG repeat-containing protein 1 OS=Homo sapiens OX=9606 GN=AGFG1 PE=	3	0,34	0,591	0,201	625444,0096	217490,6511	2098953,169
Q9UQB8	BAIAP2	Brain-specific angiogenesis inhibitor 1-associated protein 2 OS=Homo sapiens OX=9606 GN=BAI/	9	1,565	0,15	0,2	841318,2017	683111,7427	4971254,222
P84243	H3-3A	Histone H3.3 OS=Homo sapiens OX=9606 GN=H3-3A PE=1 SV=2	7	0,283	1,075	0,2	1087735177	413907874,1	941363780,4
Q6IAN0	DHRS7B	Dehydrogenase/reductase SDR family member 7B OS=Homo sapiens OX=9606 GN=DHRS7B PE=1	4	0,556	0,356	0,195	542187,6578	219727,6853	2555928,149
Q9UDY2	TJP2	Tight junction protein ZO-2 OS=Homo sapiens OX=9606 GN=TJP2 PE=1 SV=2	26	0,955	0,171	0,193	1778938,21	740342,8783	7373264,675
Q9NY93	DDX56	Probable ATP-dependent RNA helicase DDX56 OS=Homo sapiens OX=9606 GN=DDX56 PE=1 SV=:	2	0,411	0,805	0,191	784369,6149	185680,8334	1243030,825
Q9UGT4	SUSD2	Sushi domain-containing protein 2 OS=Homo sapiens OX=9606 GN=SUSD2 PE=1 SV=1	6		0,081	0,19	69049,25214	95422,98244	5071908,51
Q04912	MST1R	Macrophage-stimulating protein receptor OS=Homo sapiens OX=9606 GN=MST1R PE=1 SV=3	6	1,617	0,116	0,187	49607,69988	200323,514	1864588,574
Q5QJE6	DNTTIP2	Deoxynucleotidyltransferase terminal-interacting protein 2 OS=Homo sapiens OX=9606 GN=DNT	2	0,329	0,378	0,186	334498,0405	70257,27368	755254,78
Q9Y2S6	TMA7	Translation machinery-associated protein 7 OS=Homo sapiens OX=9606 GN=TMA7 PE=1 SV=1	2	0,019	9,483	0,177	7110263,482	393833,5314	744825,6905
Q08357	SLC20A2	Sodium-dependent phosphate transporter 2 OS=Homo sapiens OX=9606 GN=SLC20A2 PE=1 SV=:	2	0,496	0,434	0,175	942212,715	363517,8078	2414225,669
Q9UBM7	DHCR7	7-dehydrocholesterol reductase OS=Homo sapiens OX=9606 GN=DHCR7 PE=1 SV=1	6	0,181	0,867	0,173	5990780,347	1116370,077	9743300,358
Q53HL2	CDCA8	Borealin OS=Homo sapiens OX=9606 GN=CDCA8 PE=1 SV=2	2	0,215	0,727	0,172	129656,2793	27844,8142	463092,7473
P0DJI9	SAA2	Serum amyloid A-2 protein OS=Homo sapiens OX=9606 GN=SAA2 PE=1 SV=1	2	1000	0,001	0,171		38302,58158	211559,1838
Q9Y624	F11R	Junctional adhesion molecule A OS=Homo sapiens OX=9606 GN=F11R PE=1 SV=1	7	3,526	0,082	0,17	622551,2119	1598641,509	10486973,71
O43491	EPB41L2	Band 4.1-like protein 2 OS=Homo sapiens OX=9606 GN=EPB41L2 PE=1 SV=1	15	0,206	0,658	0,169	6396830,249	540072,0203	9996888,014
Q9H6F5	CCDC86	Coiled-coil domain-containing protein 86 OS=Homo sapiens OX=9606 GN=CCDC86 PE=1 SV=1	6	0,778	0,198	0,168	890965,4042	551113,0219	5186593,464
Q02241	KIF23	Kinesin-like protein KIF23 OS=Homo sapiens OX=9606 GN=KIF23 PE=1 SV=3	11	0,569	0,266	0,168	1398578,193	620479,6329	6306819,834
Q5HYI8	RABL3	Rab-like protein 3 OS=Homo sapiens OX=9606 GN=RABL3 PE=1 SV=1	2	0,249	0,674	0,168	333354,9923	83073,04227	494570,9959
Q6P4Q7	CNNM4	Metal transporter CNNM4 OS=Homo sapiens OX=9606 GN=CNNM4 PE=1 SV=3	2	0,277	0,678	0,167	417832,0435	115040,3832	1492495,118
P50443	SLC26A2	Sulfate transporter OS=Homo sapiens OX=9606 GN=SLC26A2 PE=1 SV=2	3	1,525	0,101	0,166	146185,3616	168038,515	1571158,234
Q8IXK2	GALNT12	Polypeptide N-acetylgalactosaminyltransferase 12 OS=Homo sapiens OX=9606 GN=GALNT12 PE=	4	1,958	0,559	0,162	517956,2729	227667,598	2333345,604
Q9H6S3	EPS8L2	Epidermal growth factor receptor kinase substrate 8-like protein 2 OS=Homo sapiens OX=9606 G	8	1000	0,001	0,16		1144475,744	11793424,94
O75146	HIP1R	Huntingtin-interacting protein 1-related protein OS=Homo sapiens OX=9606 GN=HIP1R PE=1 SV:	2	0,179	0,934	0,154	636581,1203	38913,10832	646436,2026
P26038	MSN	Moesin OS=Homo sapiens OX=9606 GN=MSN PE=1 SV=3	9	0,095	1,606	0,152	8537110,364	258046,3665	5198536,138
Q9NXA8	SIRT5	NAD-dependent protein deacylase sirtuin-5, mitochondrial OS=Homo sapiens OX=9606 GN=SIRT	2	1,235	0,124	0,151	161759,4561	179198,6997	2212995,958
P29034	S100A2	Protein S100-A2 OS=Homo sapiens OX=9606 GN=S100A2 PE=1 SV=3	3	3,602	0,132	0,149	254713,5609	570156,6949	4070741,153
Q9UMY1	NOL7	Nucleolar protein 7 OS=Homo sapiens OX=9606 GN=NOL7 PE=1 SV=2	2	1000	0,001	0,148		305269,4441	2048739,867
Q9Y2H5	PLEKHA6	Pleckstrin homology domain-containing family A member 6 OS=Homo sapiens OX=9606 GN=PLE	4	2,126	0,112	0,148	169127,0919	316958,8999	2859694,726
Q6ZRV2	FAM83H	Protein FAM83H OS=Homo sapiens OX=9606 GN=FAM83H PE=1 SV=3	12	1,008	0,156	0,147	228963,0305	808078,1334	9502588,672
Q9BRQ6	CHCHD6	MICOS complex subunit MIC25 OS=Homo sapiens OX=9606 GN=CHCHD6 PE=1 SV=1	3		0,191	0,143	13212,19484	74215,01428	1298672,406
Q7Z406	MYH14	Myosin-14 OS=Homo sapiens OX=9606 GN=MYH14 PE=1 SV=2	28	0,853	0,196	0,141	683485,6194	932615,8222	22415025,39
P22570	FDXR	NADPH:adrenodoxin oxidoreductase, mitochondrial OS=Homo sapiens OX=9606 GN=FDXR PE=1	9	1,003	0,168	0,139	804004,6426	956080,3058	9409398,358
P43155	CRAT	Carnitine O-acetyltransferase OS=Homo sapiens OX=9606 GN=CRAT PE=1 SV=5	3		0,079	0,138	73704,10702	95500,42971	2554001,899
P05187	ALPP	Alkaline phosphatase, placental type OS=Homo sapiens OX=9606 GN=ALPP PE=1 SV=2	3	1000	0,001	0,138		107407,3624	2514550,545
Q9Y4C8	RBM19	Probable RNA-binding protein 19 OS=Homo sapiens OX=9606 GN=RBM19 PE=1 SV=3	2	0,125	0,867	0,136	442039,46	26319,60935	481188,3126
Q15847	ADIRF	Adipogenesis regulatory factor OS=Homo sapiens OX=9606 GN=ADIRF PE=1 SV=1	2	1000	0,001	0,135		596086,7084	6733386,363
Q13428	TCOF1	Treacle protein OS=Homo sapiens OX=9606 GN=TCOF1 PE=1 SV=3	19	0,317	0,448	0,135	16565984,16	5166750,435	36545133,22
Q9BZG1	RAB34	Ras-related protein Rab-34 OS=Homo sapiens OX=9606 GN=RAB34 PE=1 SV=1	6	0,743	0,339	0,134	1057396,465	330531,2967	3598347,331
Q86SK9	SCD5	Stearoyl-CoA desaturase 5 OS=Homo sapiens OX=9606 GN=SCD5 PE=1 SV=2	2	0,273	0,497	0,134	516970,2994	148794,5748	866322,9544
Q9Y291	MRPS33	Small ribosomal subunit protein mS33 OS=Homo sapiens OX=9606 GN=MRPS33 PE=1 SV=1	2	1000	0,001	0,13		242521,7181	1838254,332
Q9UM54	MYO6	Unconventional myosin-VI OS=Homo sapiens OX=9606 GN=MYO6 PE=1 SV=4	8	0,378	0,315	0,129	680351,9487	96253,3324	3002541,86
P55196	AFDN	Afadin OS=Homo sapiens OX=9606 GN=AFDN PE=1 SV=3	4	0,171	0,556	0,127	1077580,643	59144,88879	1977811,978
P35659	DEK	Protein DEK OS=Homo sapiens OX=9606 GN=DEK PE=1 SV=1	12	0,465	0,272	0,124	10863799,76	3772746,624	38829798,44
P55290	CDH13	Cadherin-13 OS=Homo sapiens OX=9606 GN=CDH13 PE=1 SV=1	2	0,244	0,512	0,124	529132,022	129274,3566	1806178,052
P22223	CDH3	Cadherin-3 OS=Homo sapiens OX=9606 GN=CDH3 PE=1 SV=2	8	4,807	0,051	0,123	136700,7723	337162,9217	6566083,753
P18564	ITGB6	Integrin beta-6 OS=Homo sapiens OX=9606 GN=ITGB6 PE=1 SV=2	2	1000	0,001	0,121		170829,6257	2133357,817
Q9Y6Q5	AP1M2	AP-1 complex subunit mu-2 OS=Homo sapiens OX=9606 GN=AP1M2 PE=1 SV=4	4		0,329	0,12	124602,4505	152924,6941	1670884,212
P04233	CD74	HLA class II histocompatibility antigen gamma chain OS=Homo sapiens OX=9606 GN=CD74 PE=1	7	2,462	0,039	0,12	221942,3551	889155,2435	12640627,81
Q02338	BDH1	D-beta-hydroxybutyrate dehydrogenase, mitochondrial OS=Homo sapiens OX=9606 GN=BDH1 PI	2	0,896	0,133	0,119	40204,66168	36022,48768	569720,0756
P22492	H1-6	Histone H1t OS=Homo sapiens OX=9606 GN=H1-6 PE=2 SV=4	3	0,148	1,476	0,117	215013847,9	126682159,8	141236145,2
Q92947	GCDH	Glutaryl-CoA dehydrogenase, mitochondrial OS=Homo sapiens OX=9606 GN=GCDH PE=1 SV=1	4	0,536	0,183	0,115	365470,4568	410696,002	3961397,672
P31327	CPS1	Carbamoyl-phosphate synthase [ammonia], mitochondrial OS=Homo sapiens OX=9606 GN=CPS1	2	1000	0,001	0,114		67136,12997	963782,3861
Q9BUF5	TUBB6	Tubulin beta-6 chain OS=Homo sapiens OX=9606 GN=TUBB6 PE=1 SV=1	3	0,301	0,605	0,114	487936,9794	63256,64872	722974,1369
Q9HBI6	CYP4F11	Cytochrome P450 4F11 OS=Homo sapiens OX=9606 GN=CYP4F11 PE=1 SV=3	2	1000	0,001	0,113		118022,5125	1441286,08
A0A1Y0BRN3	MYO6	Unconventional myosin-VI OS=Homo sapiens OX=9606 GN=MYO6 PE=1 SV=1	5	0,322	0,374	0,111	539205,0526	80535,99113	2049072,467
P13498	CYBA	Cytochrome b-245 light chain OS=Homo sapiens OX=9606 GN=CYBA PE=1 SV=3	2	2,471	0,043	0,109	165631,5506	434786,7466	4487464,988
P05783	KRT18	Keratin, type I cytoskeletal 18 OS=Homo sapiens OX=9606 GN=KRT18 PE=1 SV=2	31	0,972	0,112	0,109	56296590,43	52119056,65	560566163,2
Q86SQ0	PHLDB2	Pleckstrin homology-like domain family B member 2 OS=Homo sapiens OX=9606 GN=PHLDB2 PE	7	0,543	0,181	0,108	471094,9129	52601,77299	4308581,062
P50895	BCAM	Basal cell adhesion molecule OS=Homo sapiens OX=9606 GN=BCAM PE=1 SV=2	13	4,096	0,026	0,1	538808,3708	5971836,52	74481011,36
Q9BQ69	MACROD1	ADP-ribose glycohydrolase MACROD1 OS=Homo sapiens OX=9606 GN=MACROD1 PE=1 SV=2	2	0,769	0,139	0,094	487540,9968	172585,9173	3344244,482
Q9Y446	PKP3	Plakophilin-3 OS=Homo sapiens OX=9606 GN=PKP3 PE=1 SV=1	9		0,089	0,088	230862,2141	940832,1905	16140341,96
Q14435	GALNT3	Polypeptide N-acetylgalactosaminyltransferase 3 OS=Homo sapiens OX=9606 GN=GALNT3 PE=1	4	1	0,313	0,087	500058,0676	626459,4577	5000830,438
P04637	TP53	Cellular tumor antigen p53 OS=Homo sapiens OX=9606 GN=TP53 PE=1 SV=4	6	1,163	0,072	0,083	80369,69495	93430,46572	11850445,5
P05549	TFAP2A	Transcription factor AP-2-alpha OS=Homo sapiens OX=9606 GN=TFAP2A PE=1 SV=1	5	0,605	0,124	0,079	954077,6094	611561,407	10684468,56
P27144	AK4	Adenylate kinase 4, mitochondrial OS=Homo sapiens OX=9606 GN=AK4 PE=1 SV=1	9	0,373	0,131	0,079	1716130,861	370485,6876	13154989,81
Q86X29	LSR	Lipolysis-stimulated lipoprotein receptor OS=Homo sapiens OX=9606 GN=LSR PE=1 SV=4	9	0,606	0,141	0,075	516757,7546	312363,5244	7054026,036
P31947	SFN	14-3-3 protein sigma OS=Homo sapiens OX=9606 GN=SFN PE=1 SV=1	10	0,825	0,115	0,072	10817886,04	8634720,376	75250819,69
P13928	ANXA8	Annexin A8 OS=Homo sapiens OX=9606 GN=ANXA8 PE=1 SV=3	2	1,451	0,066	0,068	22068,85208	1033553,629	19009323,52
Q92481	TFAP2B	Transcription factor AP-2-beta OS=Homo sapiens OX=9606 GN=TFAP2B PE=1 SV=2	4	0,426	0,148	0,061	733361,5469	241876,289	6160423,384
P32004	L1CAM	Neural cell adhesion molecule L1 OS=Homo sapiens OX=9606 GN=L1CAM PE=1 SV=2	14	0,751	0,074	0,059	2172256,563	1754096,694	31782149,61
Q969X1	TMBIM1	Protein lifeguard 3 OS=Homo sapiens OX=9606 GN=TMBIM1 PE=1 SV=2	3	1000	0,001	0,057		113172,4454	1941176,329
P05787	KRT8	Keratin, type II cytoskeletal 8 OS=Homo sapiens OX=9606 GN=KRT8 PE=1 SV=7	33	0,648	0,075	0,052	139645237,6	129130910,5	1554225670
Q8TEM1	NUP210	Nuclear pore membrane glycoprotein 210 OS=Homo sapiens OX=9606 GN=NUP210 PE=1 SV=3	19	0,068	0,863	0,05	13830598,72	315867,2326	18935032,95
Q04695	KRT17	Keratin, type I cytoskeletal 17 OS=Homo sapiens OX=9606 GN=KRT17 PE=1 SV=2	19	3,687	0,021	0,049	17442744,75	56311267,12	648505851,9
Q9BV40	VAMP8	Vesicle-associated membrane protein 8 OS=Homo sapiens OX=9606 GN=VAMP8 PE=1 SV=1	5	3,904	0,014	0,048	51977,71921	304204,9395	14600751,45
P04181	OAT	Ornithine aminotransferase, mitochondrial OS=Homo sapiens OX=9606 GN=OAT PE=1 SV=1	13	0,524	0,099	0,045	4574909,441	581829,9925	41768008,03
Q9BY67	CADM1	Cell adhesion molecule 1 OS=Homo sapiens OX=9606 GN=CADM1 PE=1 SV=2	2	0,023	1,337	0,043	1286677,76	21514,5074	864502,4345
Q14126	DSG2	Desmoglein-2 OS=Homo sapiens OX=9606 GN=DSG2 PE=1 SV=2	16	0,897	0,304	0,042	2232672,373	201308,5644	16811050,04
Q9BRX8	PRXL2A	Peroxiredoxin-like 2A OS=Homo sapiens OX=9606 GN=PRXL2A PE=1 SV=3	8	0,007	4,622	0,041	17584926,75	39314,83949	3475136,207
P47895	ALDH1A3	Retinaldehyde dehydrogenase 3 OS=Homo sapiens OX=9606 GN=ALDH1A3 PE=1 SV=2	13	1,871	0,017	0,037	154885,7264	907468,0406	39163212,5
Q5VT79	ANXA8L1	Annexin A8-like protein 1 OS=Homo sapiens OX=9606 GN=ANXA8L1 PE=2 SV=2	2	1000	0,001	0,035		182438,8578	3365733,492
P13646	KRT13	Keratin, type I cytoskeletal 13 OS=Homo sapiens OX=9606 GN=KRT13 PE=1 SV=4	8	1000	0,001	0,034		222484,922	17639433,02
P12830	CDH1	Cadherin-1 OS=Homo sapiens OX=9606 GN=CDH1 PE=1 SV=3	10	1,109	0,03	0,032	903230,0549	239409,0215	33120202,39
P25815	S100P	Protein S100-P OS=Homo sapiens OX=9606 GN=S100P PE=1 SV=2	3	1000	0,001	0,031		534479,1073	18551293,87
P63218	GNG5	Guanine nucleotide-binding protein G(I)/G(S)/G(O) subunit gamma-5 OS=Homo sapiens OX=960	2	1000	0,001	0,031		23813,11604	773887,264
P50479	PDLIM4	PDZ and LIM domain protein 4 OS=Homo sapiens OX=9606 GN=PDLIM4 PE=1 SV=2	2	1000	0,001	0,028		30602,84161	1501808,626
P09758	TACSTD2	Tumor-associated calcium signal transducer 2 OS=Homo sapiens OX=9606 GN=TACSTD2 PE=1 SV	10	3,419	0,019	0,024	339007,1363	2265304,869	96757697,39
P08727	KRT19	Keratin, type I cytoskeletal 19 OS=Homo sapiens OX=9606 GN=KRT19 PE=1 SV=4	25	2,536	0,019	0,022	37897109,16	70267991,89	1857705484
Q02539	H1-1	Histone H1.1 OS=Homo sapiens OX=9606 GN=H1-1 PE=1 SV=3	3	1000	0,001	0,019		56635,60797	4762702,655
P16422	EPCAM	Epithelial cell adhesion molecule OS=Homo sapiens OX=9606 GN=EPCAM PE=1 SV=2	2		0,028	0,014	141334,3613	52504,16762	8510464,79
O14684	PTGES	Prostaglandin E synthase OS=Homo sapiens OX=9606 GN=PTGES PE=1 SV=2	3	2,615	0,002	0,006	72829,08565	190438,5138	44919030,76
P08729	KRT7	Keratin, type II cytoskeletal 7 OS=Homo sapiens OX=9606 GN=KRT7 PE=1 SV=5	29	1,452	0,004	0,006	8432523,23	26785205,18	1871099709
O15231	ZNF185	Zinc finger protein 185 OS=Homo sapiens OX=9606 GN=ZNF185 PE=1 SV=3	6		0,001	0,001			5050548,036
Q8WWA1	TMEM40	Transmembrane protein 40 OS=Homo sapiens OX=9606 GN=TMEM40 PE=1 SV=2	5		0,001	0,001			4999797,251
Q9C002	NMES1	Normal mucosa of esophagus-specific gene 1 protein OS=Homo sapiens OX=9606 GN=NMES1 PE	5		0,001	0,001			16984206,06
O60603	TLR2	Toll-like receptor 2 OS=Homo sapiens OX=9606 GN=TLR2 PE=1 SV=1	5		0,001	0,001			236065,8549
Q9BW04	SARG	Specifically androgen-regulated gene protein OS=Homo sapiens OX=9606 GN=SARG PE=1 SV=2	4		0,001	0,001			2602370,872
Q11201	ST3GAL1	CMP-N-acetylneuraminate-beta-galactosamide-alpha-2,3-sialyltransferase 1 OS=Homo sapiens C	4		0,001	0,001			36788,341
Q96KN1	LRATD2	Protein LRATD2 OS=Homo sapiens OX=9606 GN=LRATD2 PE=1 SV=1	4		0,001	0,001			892865,1665
O60449	LY75	Lymphocyte antigen 75 OS=Homo sapiens OX=9606 GN=LY75 PE=1 SV=3	4		0,001	0,001			435644,3667

P07204	THBD	Thrombomodulin OS=Homo sapiens OX=9606 GN=THBD PE=1 SV=2	3		0,001	0,001			311782,4796
Q9UKU7	ACAD8	Isobutyryl-CoA dehydrogenase, mitochondrial OS=Homo sapiens OX=9606 GN=ACAD8 PE=1 SV=	3		0,001	0,001			1408669,366
Q9BXR0	QTRT1	Queuine tRNA-ribosyltransferase catalytic subunit 1 OS=Homo sapiens OX=9606 GN=QTRT1 PE=	3		0,001	0,001			32314,14129
Q8N271	PROM2	Prominin-2 OS=Homo sapiens OX=9606 GN=PROM2 PE=1 SV=1	3		0,001	0,001			81755,95149
Q8N556	AFAP1	Actin filament-associated protein 1 OS=Homo sapiens OX=9606 GN=AFAP1 PE=1 SV=2	3		0,001	0,001			1745658,76
Q7Z7K6	CENPV	Centromere protein V OS=Homo sapiens OX=9606 GN=CENPV PE=1 SV=1	3		0,001	0,001			164968,1843
Q92876	KLK6	Kallikrein-6 OS=Homo sapiens OX=9606 GN=KLK6 PE=1 SV=1	2		0,001	0,001			3471270,875
Q9ULV3	CIZ1	Cip1-interacting zinc finger protein OS=Homo sapiens OX=9606 GN=CIZ1 PE=1 SV=2	2		0,001	0,001			636888,3057
Q9NSC5	HOMER3	Homer protein homolog 3 OS=Homo sapiens OX=9606 GN=HOMER3 PE=1 SV=2	2		0,001	0,001			1120142,043
Q9Y375	NDUFAF1	Complex I intermediate-associated protein 30, mitochondrial OS=Homo sapiens OX=9606 GN=NI	2		0,001	0,001			210278,2638
Q8N1S5	SLC39A11	Zinc transporter ZIP11 OS=Homo sapiens OX=9606 GN=SLC39A11 PE=2 SV=3	2		0,001	0,001			957295,4215
Q9HCY8	S100A14	Protein S100-A14 OS=Homo sapiens OX=9606 GN=S100A14 PE=1 SV=1	2		0,001	0,001			273062,6368
O43166	SIPA1L1	Signal-induced proliferation-associated 1-like protein 1 OS=Homo sapiens OX=9606 GN=SIPA1L1	2		0,001	0,001			210160,6567
Q96NY8	NECTIN4	Nectin-4 OS=Homo sapiens OX=9606 GN=NECTIN4 PE=1 SV=1	2		0,001	0,001			297615,9221
Q92485	SMPDL3B	Acid sphingomyelinase-like phosphodiesterase 3b OS=Homo sapiens OX=9606 GN=SMPDL3B PE=	2		0,001	0,001			1065868,815
Q3ZCQ3	FAM174B	Membrane protein FAM174B OS=Homo sapiens OX=9606 GN=FAM174B PE=2 SV=2	2		0,001	0,001			476265,0109
P04626	ERBB2	Receptor tyrosine-protein kinase erbB-2 OS=Homo sapiens OX=9606 GN=ERBB2 PE=1 SV=1	2		0,001	0,001			543640,2071
P21709	EPHA1	Ephrin type-A receptor 1 OS=Homo sapiens OX=9606 GN=EPHA1 PE=1 SV=4	2		0,001	0,001			590919,8427
Q6UXV4	APOOL	MICOS complex subunit MIC27 OS=Homo sapiens OX=9606 GN=APOOL PE=1 SV=1	2		0,001	0,001			1253577,269
Q96K37	SLC35E1	Solute carrier family 35 member E1 OS=Homo sapiens OX=9606 GN=SLC35E1 PE=1 SV=2	2		0,001	0,001			709567,6297
Q6I9Y2	THOC7	THO complex subunit 7 homolog OS=Homo sapiens OX=9606 GN=THOC7 PE=1 SV=3	2		0,001	0,001			291258,2903
Q9NWU2	GID8	Glucose-induced degradation protein 8 homolog OS=Homo sapiens OX=9606 GN=GID8 PE=1 SV=	2		0,001	0,001			277341,4816
Q6ISB3	GRHL2	Grainyhead-like protein 2 homolog OS=Homo sapiens OX=9606 GN=GRHL2 PE=1 SV=1	2		0,001	0,001			642340,304
Q9BQL6	FERMT1	Fermitin family homolog 1 OS=Homo sapiens OX=9606 GN=FERMT1 PE=1 SV=1	2		0,001	0,001			226747,6788
O60476	MAN1A2	Mannosyl-oligosaccharide 1,2-alpha-mannosidase IB OS=Homo sapiens OX=9606 GN=MAN1A2 P	2		0,001	0,001			26795,19544
Q6NXG1	ESRP1	Epithelial splicing regulatory protein 1 OS=Homo sapiens OX=9606 GN=ESRP1 PE=1 SV=2	2		0,001	0,001			326696,7777
Q96JQ2	CLMN	Calmin OS=Homo sapiens OX=9606 GN=CLMN PE=1 SV=1	2		0,001	0,001			490872,8863
O60487	MPZL2	Myelin protein zero-like protein 2 OS=Homo sapiens OX=9606 GN=MPZL2 PE=1 SV=1	2		0,001	0,001			351413,8504
P50440	GATM	Glycine amidinotransferase, mitochondrial OS=Homo sapiens OX=9606 GN=GATM PE=1 SV=1	6	0,001	0,646	0,001	276626,0408		1046807,737
Q16352	INA	Alpha-internexin OS=Homo sapiens OX=9606 GN=INA PE=1 SV=2	6	0,001	1,873	0,001	4674126,341		2595568,184
P57735	RAB25	Ras-related protein Rab-25 OS=Homo sapiens OX=9606 GN=RAB25 PE=1 SV=2	6	0,001	0,103	0,001	124602,0494		4689322,846
O95864	FADS2	Acyl-CoA 6-desaturase OS=Homo sapiens OX=9606 GN=FADS2 PE=1 SV=1	6	0,001	9,728	0,001	15851829,32		470998,7722
Q99988	GDF15	Growth/differentiation factor 15 OS=Homo sapiens OX=9606 GN=GDF15 PE=1 SV=3	5	0,001	0,897	0,001	267304,1715		367776,2389
Q9NU22	MDN1	Midasin OS=Homo sapiens OX=9606 GN=MDN1 PE=1 SV=2	5	0,001	1,896	0,001	1414904,256		403273,2897
Q8IVT2	MISP	Mitotic interactor and substrate of PLK1 OS=Homo sapiens OX=9606 GN=MISP PE=1 SV=1	5	0,001	0,296	0,001	104382,5394		1954650,636
Q9BS40	LXN	Latexin OS=Homo sapiens OX=9606 GN=LXN PE=1 SV=2	4	0,001		0,001	277182,3609		540546,2971
A8MXV4	NUDT19	Acyl-coenzyme A diphosphatase NUDT19 OS=Homo sapiens OX=9606 GN=NUDT19 PE=1 SV=1	4	0,001	0,603	0,001	77770,91964		128952,2773
Q9H974	QTRT2	Queuine tRNA-ribosyltransferase accessory subunit 2 OS=Homo sapiens OX=9606 GN=QTRT2 PE	4	0,001	1,123	0,001	533253,1548		478806,8835
Q6DKK2	TTC19	Tetratricopeptide repeat protein 19, mitochondrial OS=Homo sapiens OX=9606 GN=TTC19 PE=1	4	0,001	0,589	0,001	1047095,864		3511281
Q2M2I8	AAK1	AP2-associated protein kinase 1 OS=Homo sapiens OX=9606 GN=AAK1 PE=1 SV=3	4	0,001	0,934	0,001	566550,99		128757,5117
Q9Y6X4	FAM169A	Soluble lamin-associated protein of 75 kDa OS=Homo sapiens OX=9606 GN=FAM169A PE=1 SV=2	3	0,001	1,155	0,001	208814,6061		519604,8659
Q9H3G5	CPVL	Probable serine carboxypeptidase CPVL OS=Homo sapiens OX=9606 GN=CPVL PE=1 SV=2	3	0,001	0,57	0,001	651688,3644		639284,1389
Q9Y4A5	TRRAP	Transformation/transcription domain-associated protein OS=Homo sapiens OX=9606 GN=TRRAP	3	0,001	0,734	0,001	127063,2714		177475,0331
P78316	NOP14	Nucleolar protein 14 OS=Homo sapiens OX=9606 GN=NOP14 PE=1 SV=3	3	0,001	0,636	0,001	476414,552		945104,7941
Q9BX66	SORBS1	Sorbin and SH3 domain-containing protein 1 OS=Homo sapiens OX=9606 GN=SORBS1 PE=1 SV=3	3	0,001	1,756	0,001	343975,7054		3478236,602
Q9H9Q2	COPS7B	COP9 signalosome complex subunit 7b OS=Homo sapiens OX=9606 GN=COPS7B PE=1 SV=1	3	0,001	1,302	0,001	676764,5389		519636,5618
P55081	MFAP1	Microfibrillar-associated protein 1 OS=Homo sapiens OX=9606 GN=MFAP1 PE=1 SV=2	3	0,001	0,136	0,001	95523,79787		1048319,273
Q969G5	CAVIN3	Caveolae-associated protein 3 OS=Homo sapiens OX=9606 GN=CAVIN3 PE=1 SV=3	3	0,001	0,106	0,001	92776,16672		2368678,958
Q13753	LAMC2	Laminin subunit gamma-2 OS=Homo sapiens OX=9606 GN=LAMC2 PE=1 SV=2	3	0,001	0,427	0,001	442775,1246		1117199,079
Q15059	BRD3	Bromodomain-containing protein 3 OS=Homo sapiens OX=9606 GN=BRD3 PE=1 SV=1	3	0,001	0,903	0,001	387712,2713		1096069,843
O75054	IGSF3	Immunoglobulin superfamily member 3 OS=Homo sapiens OX=9606 GN=IGSF3 PE=2 SV=3	3	0,001	1,941	0,001	958366,7452		248654,0169
P29762	CRABP1	Cellular retinoic acid-binding protein 1 OS=Homo sapiens OX=9606 GN=CRABP1 PE=1 SV=2	3	0,001	50,71	0,001	11980696,17		95048,24955
Q9P2M7	CGN	Cingulin OS=Homo sapiens OX=9606 GN=CGN PE=1 SV=3	3	0,001	0,357	0,001	159807,7737		649210,0981
Q9Y287	ITM2B	Integral membrane protein 2B OS=Homo sapiens OX=9606 GN=ITM2B PE=1 SV=1	3	0,001	0,98	0,001	140922,0892		607730,0066
Q99828	CIB1	Calcium and integrin-binding protein 1 OS=Homo sapiens OX=9606 GN=CIB1 PE=1 SV=4	3	0,001	1,027	0,001	332020,2518		177744,4544
Q15061	WDR43	WD repeat-containing protein 43 OS=Homo sapiens OX=9606 GN=WDR43 PE=1 SV=3	3	0,001	0,433	0,001	681267,1142		1317925,269
Q9NUM4	TMEM106B	Transmembrane protein 106B OS=Homo sapiens OX=9606 GN=TMEM106B PE=1 SV=2	2	0,001	3,316	0,001	1588732,076		479100,9132
Q9BYC9	MRPL20	Large ribosomal subunit protein bL20m OS=Homo sapiens OX=9606 GN=MRPL20 PE=1 SV=1	2	0,001	0,617	0,001	327400,0555		525107,1108
Q9NUB1	ACSS1	Acetyl-coenzyme A synthetase 2-like, mitochondrial OS=Homo sapiens OX=9606 GN=ACSS1 PE=1	2	0,001	0,824	0,001	468680,2549		663783,2189
P13747	HLA-E	HLA class I histocompatibility antigen, alpha chain E OS=Homo sapiens OX=9606 GN=HLA-E PE=1	2	0,001	0,226	0,001	176474,5295		1076221,259
O43291	SPINT2	Kunitz-type protease inhibitor 2 OS=Homo sapiens OX=9606 GN=SPINT2 PE=1 SV=2	2	0,001	0,162	0,001	166816,1914		1034349,997
Q69YQ0	SPECC1L	Cytospin-A OS=Homo sapiens OX=9606 GN=SPECC1L PE=1 SV=3	2	0,001	0,366	0,001	127684,3771		876128,0208
P29033	GJB2	Gap junction beta-2 protein OS=Homo sapiens OX=9606 GN=GJB2 PE=1 SV=3	2	0,001	1,088	0,001	153084,6294		140660,0188
Q9H4A3	WNK1	Serine/threonine-protein kinase WNK1 OS=Homo sapiens OX=9606 GN=WNK1 PE=1 SV=2	2	0,001	2,496	0,001	296338,1901		123459,8485
P52756	RBM5	RNA-binding protein 5 OS=Homo sapiens OX=9606 GN=RBM5 PE=1 SV=2	2	0,001	0,192	0,001	54661,09495		564493,1319
P04350	TUBB4A	Tubulin beta-4A chain OS=Homo sapiens OX=9606 GN=TUBB4A PE=1 SV=2	2	0,001	1,997	0,001	1012717,222		166582,1704
Q9Y4C2	TCAF1	TRPM8 channel-associated factor 1 OS=Homo sapiens OX=9606 GN=TCAF1 PE=1 SV=3	2	0,001	1,123	0,001	851979,5142		805878,6106
P14859	POU2F1	POU domain, class 2, transcription factor 1 OS=Homo sapiens OX=9606 GN=POU2F1 PE=1 SV=2	2	0,001	2,152	0,001	1096792,048		270666,5479
O94760	DDAH1	N(G),N(G)-dimethylarginine dimethylaminohydrolase 1 OS=Homo sapiens OX=9606 GN=DDAH1 I	2	0,001	2,278	0,001	697959,7696		306398,5564
Q96BN2	TADA1	Transcriptional adapter 1 OS=Homo sapiens OX=9606 GN=TADA1 PE=1 SV=1	2	0,001	0,791	0,001	99980,7227		126472,52
Q92626	PXDN	Peroxidasin homolog OS=Homo sapiens OX=9606 GN=PXDN PE=1 SV=2	2	0,001	0,045	0,001	20395,42982		457252,7268
Q9Y487	ATP6V0A2	V-type proton ATPase 116 kDa subunit a 2 OS=Homo sapiens OX=9606 GN=ATP6V0A2 PE=1 SV=2	2	0,001	1,678	0,001	251771,8957		150085,0524
Q96GW9	MARS2	Methionine--tRNA ligase, mitochondrial OS=Homo sapiens OX=9606 GN=MARS2 PE=1 SV=2	2	0,001	1,081	0,001	177901,1299		155487,898
P35219	CA8	Carbonic anhydrase-related protein OS=Homo sapiens OX=9606 GN=CA8 PE=1 SV=3	2	0,001	0,616	0,001	672302,2492		634904,1909
Q9HAF1	MEAF6	Chromatin modification-related protein MEAF6 OS=Homo sapiens OX=9606 GN=MEAF6 PE=1 SV	2	0,001	1,049	0,001	617856,8904		530991,3835
O95363	FARS2	Phenylalanine--tRNA ligase, mitochondrial OS=Homo sapiens OX=9606 GN=FARS2 PE=1 SV=1	2	0,001	0,61	0,001	72273,88835		586551,187
Q9BZE9	ASPSCR1	Tether containing UBX domain for GLUT4 OS=Homo sapiens OX=9606 GN=ASPSCR1 PE=1 SV=1	2	0,001	1,984	0,001	857367,7682		432082,6371
Q92614	MYO18A	Unconventional myosin-XVIIIa OS=Homo sapiens OX=9606 GN=MYO18A PE=1 SV=3	2	0,001	0,39	0,001	262500,0778		653475,0726
Q9NUL3	STAU2	Double-stranded RNA-binding protein Staufen homolog 2 OS=Homo sapiens OX=9606 GN=STAU	2	0,001	1,349	0,001	729798,9896		850295,5369
P51654	GPC3	Glypican-3 OS=Homo sapiens OX=9606 GN=GPC3 PE=1 SV=1	2	0,001	2,849	0,001	579917,053		135744,1823
P78345	RPP38	Ribonuclease P protein subunit p38 OS=Homo sapiens OX=9606 GN=RPP38 PE=1 SV=2	2	0,001	0,743	0,001	226750,1477		308323,0145
P53602	MVD	Diphosphomevalonate decarboxylase OS=Homo sapiens OX=9606 GN=MVD PE=1 SV=1	2	0,001	1,647	0,001	319747,5758		198737,2992
Q9UGV2	NDRG3	Protein NDRG3 OS=Homo sapiens OX=9606 GN=NDRG3 PE=1 SV=2	2	0,001	2,027	0,001	443571,7858		215951,9794
Q6IN84	MRM1	rRNA methyltransferase 1, mitochondrial OS=Homo sapiens OX=9606 GN=MRM1 PE=1 SV=1	2	0,001	0,166	0,001	21478,78528		134646,3011
P46734	MAP2K3	Dual specificity mitogen-activated protein kinase kinase 3 OS=Homo sapiens OX=9606 GN=MAP2	2	0,001	0,424	0,001	33035,04705		212730,384
Q9BQ61	TRIR	Telomerase RNA component interacting RNase OS=Homo sapiens OX=9606 GN=TRIR PE=1 SV=1	2	0,001	0,588	0,001	282235,8706		622532,8841
O60499	STX10	Syntaxin-10 OS=Homo sapiens OX=9606 GN=STX10 PE=1 SV=1	2	0,001	0,259	0,001	270762,0869		1044481,187
H7C1W1	PXDN	Peroxidasin (Fragment) OS=Homo sapiens OX=9606 GN=PXDN PE=1 SV=1	2	0,001	0,051	0,001	20560,95782		391835,9838
P14317	HCLS1	Hematopoietic lineage cell-specific protein OS=Homo sapiens OX=9606 GN=HCLS1 PE=1 SV=3	4						
Q9NQ84	GPRC5C	G-protein coupled receptor family C group 5 member C OS=Homo sapiens OX=9606 GN=GPRC5C	3						
P01903	HLA-DRA	HLA class II histocompatibility antigen, DR alpha chain OS=Homo sapiens OX=9606 GN=HLA-DRA	3						
Q8TEQ8	PIGO	GPI ethanolamine phosphate transferase 3 OS=Homo sapiens OX=9606 GN=PIGO PE=1 SV=3	3						
P55317	FOXA1	Hepatocyte nuclear factor 3-alpha OS=Homo sapiens OX=9606 GN=FOXA1 PE=1 SV=2	3						
Q8N4A0	GALNT4	Polypeptide N-acetylgalactosaminyltransferase 4 OS=Homo sapiens OX=9606 GN=GALNT4 PE=1	2						
O95361	TRIM16	Tripartite motif-containing protein 16 OS=Homo sapiens OX=9606 GN=TRIM16 PE=1 SV=3	2						
O14498	ISLR	Immunoglobulin superfamily containing leucine-rich repeat protein OS=Homo sapiens OX=9606	2						
Q5VYY1	ANKRD22	Ankyrin repeat domain-containing protein 22 OS=Homo sapiens OX=9606 GN=ANKRD22 PE=2 SV	2						
O60518	RANBP6	Ran-binding protein 6 OS=Homo sapiens OX=9606 GN=RANBP6 PE=1 SV=2	2						
Q14562	DHX8	ATP-dependent RNA helicase DHX8 OS=Homo sapiens OX=9606 GN=DHX8 PE=1 SV=1	2						
Q5VZ89	DENND4C	DENN domain-containing protein 4C OS=Homo sapiens OX=9606 GN=DENND4C PE=1 SV=3	2						
Q53SF7	COBLL1	Cordon-bleu protein-like 1 OS=Homo sapiens OX=9606 GN=COBLL1 PE=1 SV=3	2						
Q96A54	ADIPOR1	Adiponectin receptor protein 1 OS=Homo sapiens OX=9606 GN=ADIPOR1 PE=1 SV=1	2						
P0CG35	TMSB15B	Thymosin beta-15B OS=Homo sapiens OX=9606 GN=TMSB15B PE=1 SV=1	2						
Q15650	TRIP4	Activating signal cointegrator 1 OS=Homo sapiens OX=9606 GN=TRIP4 PE=1 SV=4	2						
O95678	KRT75	Keratin, type II cytoskeletal 75 OS=Homo sapiens OX=9606 GN=KRT75 PE=1 SV=2	2						

Proteins identified with unique peptide number equal to or more than 5

Accession	Description	Abundance Ratio: (HTB-5 PR) / (HTB-5)	Abundance Ratio: (HTB-5 PR) / (SV-HUC-1)	Abundance Ratio: (HTB-5) / (SV-HUC-1)
Q9Y4K1	Beta/gamma crystallin domain-containing protein 1 OS=Homo sapiens OX=9606 GN=CRYBG1 PE=1 SV=3	0,41963911		0,888
Q03169	Tumor necrosis factor alpha-induced protein 2 OS=Homo sapiens OX=9606 GN=TNFAIP2 PE=1 SV=2	0,254906959		3,124
Q9UKX5	Integrin alpha-11 OS=Homo sapiens OX=9606 GN=ITGA11 PE=1 SV=2	0,13559322		8,385
P51884	Lumican OS=Homo sapiens OX=9606 GN=LUM PE=1 SV=2	0,001		1000
Q14CN2	Calcium-activated chloride channel regulator 4 OS=Homo sapiens OX=9606 GN=CLCA4 PE=1 SV=2			1,187
Q6P179	Endoplasmic reticulum aminopeptidase 2 OS=Homo sapiens OX=9606 GN=ERAP2 PE=1 SV=2			0,742
Q6P1M3	LLGL scribble cell polarity complex component 2 OS=Homo sapiens OX=9606 GN=LLGL2 PE=1 SV=2			0,36
O75363	Breast carcinoma-amplified sequence 1 OS=Homo sapiens OX=9606 GN=BCAS1 PE=1 SV=2	1000	1000	
Q9BPU6	Dihydropyrimidinase-related protein 5 OS=Homo sapiens OX=9606 GN=DPYSL5 PE=1 SV=1	40	1000	1000
O60245	Protocadherin-7 OS=Homo sapiens OX=9606 GN=PCDH7 PE=1 SV=2	2,5	1000	1000
Q92673	Sortilin-related receptor OS=Homo sapiens OX=9606 GN=SORL1 PE=1 SV=2	1,27388535	1000	1000
Q10472	Polypeptide N-acetylgalactosaminyltransferase 1 OS=Homo sapiens OX=9606 GN=GALNT1 PE=1 SV=1	1,069518717	1000	1000
P54868	Hydroxymethylglutaryl-CoA synthase, mitochondrial OS=Homo sapiens OX=9606 GN=HMGCS2 PE=1 SV=1	0,973709834	1000	1000
P15428	15-hydroxyprostaglandin dehydrogenase [NAD(+)] OS=Homo sapiens OX=9606 GN=HPGD PE=1 SV=1	0,962463908	1000	1000
P52895	Aldo-keto reductase family 1 member C2 OS=Homo sapiens OX=9606 GN=AKR1C2 PE=1 SV=3	0,580720093	1000	1000
Q99985	Semaphorin-3C OS=Homo sapiens OX=9606 GN=SEMA3C PE=2 SV=2	0,354735722	1000	1000
P26022	Pentraxin-related protein PTX3 OS=Homo sapiens OX=9606 GN=PTX3 PE=1 SV=3	0,315656566	1000	1000
Q9UDR5	Alpha-aminoadipic semialdehyde synthase, mitochondrial OS=Homo sapiens OX=9606 GN=AASS PE=1 SV=1		1000	1000
P07196	Neurofilament light polypeptide OS=Homo sapiens OX=9606 GN=NEFL PE=1 SV=3	7,874015748	180,587	
P12277	Creatine kinase B-type OS=Homo sapiens OX=9606 GN=CKB PE=1 SV=1	4,366812227	104,267	26,258
P52926	High mobility group protein HMGI-C OS=Homo sapiens OX=9606 GN=HMGA2 PE=1 SV=1	52,63157895	68,759	1,398
P08133	Annexin A6 OS=Homo sapiens OX=9606 GN=ANXA6 PE=1 SV=3	1,492537313	63,943	45,162
Q01581	Hydroxymethylglutaryl-CoA synthase, cytoplasmic OS=Homo sapiens OX=9606 GN=HMGCS1 PE=1 SV=2	38,46153846	20,276	0,657
Q13509	Tubulin beta-3 chain OS=Homo sapiens OX=9606 GN=TUBB3 PE=1 SV=2	6,369426752	28,988	4,788
O95865	Putative hydrolase DDAH2 OS=Homo sapiens OX=9606 GN=DDAH2 PE=1 SV=1	3,436426117	25,508	7,11
O75368	Adapter SH3BGRL OS=Homo sapiens OX=9606 GN=SH3BGRL PE=1 SV=1	1,383125864	23,356	16,746
Q9NZI8	Insulin-like growth factor 2 mRNA-binding protein 1 OS=Homo sapiens OX=9606 GN=IGF2BP1 PE=1 SV=2	8,928571429	22,163	2,413
Q16658	Fascin OS=Homo sapiens OX=9606 GN=FSCN1 PE=1 SV=3	2,631578947	20,954	8,108
P08670	Vimentin OS=Homo sapiens OX=9606 GN=VIM PE=1 SV=4	28,57142857	30,806	0,635
P17677	Neuromodulin OS=Homo sapiens OX=9606 GN=GAP43 PE=1 SV=1	24,3902439	7,189	0,647
A0A1C7CYX9	Dihydropyrimidinase-related protein 2 OS=Homo sapiens OX=9606 GN=DPYSL2 PE=1 SV=1	3,164556962	19,439	5,612
P46821	Microtubule-associated protein 1B OS=Homo sapiens OX=9606 GN=MAP1B PE=1 SV=2	3,921568627	17,908	6,686
Q16555	Dihydropyrimidinase-related protein 2 OS=Homo sapiens OX=9606 GN=DPYSL2 PE=1 SV=1	2,702702703	17,36	5,877
Q9UNF1	Melanoma-associated antigen D2 OS=Homo sapiens OX=9606 GN=MAGED2 PE=1 SV=2	4,149377593	17,202	5,229
Q6DKJ4	Nucleoredoxin OS=Homo sapiens OX=9606 GN=NXN PE=1 SV=2	2,638522427	15,184	5,997
P07437	Tubulin beta chain OS=Homo sapiens OX=9606 GN=TUBB PE=1 SV=2	3,378378378	15,06	4,527
P07197	Neurofilament medium polypeptide OS=Homo sapiens OX=9606 GN=NEFM PE=1 SV=3	18,86792453	19,872	1,805
Q14194	Dihydropyrimidinase-related protein 1 OS=Homo sapiens OX=9606 GN=CRMP1 PE=1 SV=1	1,76056338	14,76	16,085
P98082	Disabled homolog 2 OS=Homo sapiens OX=9606 GN=DAB2 PE=1 SV=3	0,263991552	14,418	21,621
O15067	Phosphoribosylformylglycinamidine synthase OS=Homo sapiens OX=9606 GN=PFAS PE=1 SV=4	3,215434084	14,321	3,725
Q8NBQ5	Estradiol 17-beta-dehydrogenase 11 OS=Homo sapiens OX=9606 GN=HSD17B11 PE=1 SV=4	0,568828214	14,022	28,187
P09936	Ubiquitin carboxyl-terminal hydrolase isozyme L1 OS=Homo sapiens OX=9606 GN=UCHL1 PE=1 SV=2	2,183406114	12,322	4,662
Q04760	Lactoylglutathione lyase OS=Homo sapiens OX=9606 GN=GLO1 PE=1 SV=4	3,194888179	11,785	2,674
P09104	Gamma-enolase OS=Homo sapiens OX=9606 GN=ENO2 PE=1 SV=3	1,240694789	11,044	3,809
Q15417	Calponin-3 OS=Homo sapiens OX=9606 GN=CNN3 PE=1 SV=1	2,688172043	10,989	4,484
Q9UKA9	Polypyrimidine tract-binding protein 2 OS=Homo sapiens OX=9606 GN=PTBP2 PE=1 SV=1	16,66666667	14,792	1,089
Q16527	Cysteine and glycine-rich protein 2 OS=Homo sapiens OX=9606 GN=CSRP2 PE=1 SV=3	6,369426752	10,63	2,856
P21266	Glutathione S-transferase Mu 3 OS=Homo sapiens OX=9606 GN=GSTM3 PE=1 SV=3	1,754385965	10,182	6,463
Q13451	Peptidyl-prolyl cis-trans isomerase FKBP5 OS=Homo sapiens OX=9606 GN=FKBP5 PE=1 SV=2	14,92537313	5,89	0,514
O95864	Acyl-CoA 6-desaturase OS=Homo sapiens OX=9606 GN=FADS2 PE=1 SV=1	1000	9,728	0,001
Q5T7N2	LINE-1 type transposase domain-containing protein 1 OS=Homo sapiens OX=9606 GN=L1TD1 PE=1 SV=1	14,28571429	10,954	0,866
P07195	L-lactate dehydrogenase B chain OS=Homo sapiens OX=9606 GN=LDHB PE=1 SV=2	3,300330033	9,268	3,396
P15090	Fatty acid-binding protein, adipocyte OS=Homo sapiens OX=9606 GN=FABP4 PE=1 SV=3	0,796178344	8,735	6,075
Q16881	Thioredoxin reductase 1, cytoplasmic OS=Homo sapiens OX=9606 GN=TXNRD1 PE=1 SV=3	0,693481276	8,604	10,196
P55209	Nucleosome assembly protein 1-like 1 OS=Homo sapiens OX=9606 GN=NAP1L1 PE=1 SV=1	3,25732899	8,387	2,806
P26640	Valine--tRNA ligase OS=Homo sapiens OX=9606 GN=VARS1 PE=1 SV=4	2,277904328	8,117	3,819
Q5VV42	Threonylcarbamoyladenosine tRNA methylthiotransferase OS=Homo sapiens OX=9606 GN=CDKAL1 PE=1 SV=1	0,914076782	8,077	6,282
Q9P258	Protein RCC2 OS=Homo sapiens OX=9606 GN=RCC2 PE=1 SV=2	10,98901099	2,847	0,676
Q14738	Serine/threonine-protein phosphatase 2A 56 kDa regulatory subunit delta isoform OS=Homo sapiens OX=9606 GN=PPP2R5I	1,976284585	7,85	2,61
P49327	Fatty acid synthase OS=Homo sapiens OX=9606 GN=FASN PE=1 SV=3	10,41666667	6,476	0,636
P11802	Cyclin-dependent kinase 4 OS=Homo sapiens OX=9606 GN=CDK4 PE=1 SV=2	2,69541779	7,389	3,576
Q13309	S-phase kinase-associated protein 2 OS=Homo sapiens OX=9606 GN=SKP2 PE=1 SV=2	8,771929825	9,283	0,705
O43175	D-3-phosphoglycerate dehydrogenase OS=Homo sapiens OX=9606 GN=PHGDH PE=1 SV=4	2,352941176	7,112	3,188
P48681	Nestin OS=Homo sapiens OX=9606 GN=NES PE=1 SV=2	7,407407407	5,837	0,638
Q71U36	Tubulin alpha-1A chain OS=Homo sapiens OX=9606 GN=TUBA1A PE=1 SV=1	2,985074627	6,994	2,127
P48506	Glutamate--cysteine ligase catalytic subunit OS=Homo sapiens OX=9606 GN=GCLC PE=1 SV=2	1,39275766	6,77	2,895
O75891	Cytosolic 10-formyltetrahydrofolate dehydrogenase OS=Homo sapiens OX=9606 GN=ALDH1L1 PE=1 SV=2	0,766283525	6,744	8,354
Q9BWD1	Acetyl-CoA acetyltransferase, cytosolic OS=Homo sapiens OX=9606 GN=ACAT2 PE=1 SV=2	6,622516556	5,348	0,759
P52209	6-phosphogluconate dehydrogenase, decarboxylating OS=Homo sapiens OX=9606 GN=PGD PE=1 SV=3	6,578947368	7,988	1,211
Q14554	Protein disulfide-isomerase A5 OS=Homo sapiens OX=9606 GN=PDIA5 PE=1 SV=1	0,550660793	6,327	11,155
P68104	Elongation factor 1-alpha 1 OS=Homo sapiens OX=9606 GN=EEF1A1 PE=1 SV=1	1,492537313	6,263	1,536
O75874	Isocitrate dehydrogenase [NADP] cytoplasmic OS=Homo sapiens OX=9606 GN=IDH1 PE=1 SV=2	2,331002331	6,203	2,467
P11137	Microtubule-associated protein 2 OS=Homo sapiens OX=9606 GN=MAP2 PE=1 SV=4	5,235602094	6,17	0,481
P11171	Protein 4.1 OS=Homo sapiens OX=9606 GN=EPB41 PE=1 SV=4	2,398081535	6,053	2,822
P13591	Neural cell adhesion molecule 1 OS=Homo sapiens OX=9606 GN=NCAM1 PE=1 SV=3	6,369426752	4,503	1,417
O00273	DNA fragmentation factor subunit alpha OS=Homo sapiens OX=9606 GN=DFFA PE=1 SV=1	5,780346821	2,91	0,755
Q9H857	5'-nucleotidase domain-containing protein 2 OS=Homo sapiens OX=9606 GN=NT5DC2 PE=1 SV=1	5,434782609	5,426	1,379
Q99471	Prefoldin subunit 5 OS=Homo sapiens OX=9606 GN=PFDN5 PE=1 SV=2	2,325581395	5,899	3,058
P04818	Thymidylate synthase OS=Homo sapiens OX=9606 GN=TYMS PE=1 SV=3	5,376344086	10,026	1,648
P09211	Glutathione S-transferase P OS=Homo sapiens OX=9606 GN=GSTP1 PE=1 SV=2	5,263157895	4,633	0,842

P50502	Hsc70-interacting protein OS=Homo sapiens OX=9606 GN=ST13 PE=1 SV=2	5,025125628	3,021	0,519
P08473	Neprilysin OS=Homo sapiens OX=9606 GN=MME PE=1 SV=2	0,155738981	5,829	17,823
O95340	Bifunctional 3'-phosphoadenosine 5'-phosphosulfate synthase 2 OS=Homo sapiens OX=9606 GN=PAPSS2 PE=1 SV=2	0,249625562	5,778	27,152
P31150	Rab GDP dissociation inhibitor alpha OS=Homo sapiens OX=9606 GN=GDI1 PE=1 SV=2	1,589825119	5,764	2,958
Q16850	Lanosterol 14-alpha demethylase OS=Homo sapiens OX=9606 GN=CYP51A1 PE=1 SV=4	4,926108374	3,368	0,752
Q92922	SWI/SNF complex subunit SMARCC1 OS=Homo sapiens OX=9606 GN=SMARCC1 PE=1 SV=3	4,901960784	2,256	0,576
P30566	Adenylosuccinate lyase OS=Homo sapiens OX=9606 GN=ADSL PE=1 SV=2	4,761904762	3,731	0,916
P15586	N-acetylglucosamine-6-sulfatase OS=Homo sapiens OX=9606 GN=GNS PE=1 SV=3	1,2300123	5,593	4,344
A0A0U1RRM6	ENAH actin regulator OS=Homo sapiens OX=9606 GN=ENAH PE=1 SV=1	2,257336343	5,557	3,179
P08238	Heat shock protein HSP 90-beta OS=Homo sapiens OX=9606 GN=HSP90AB1 PE=1 SV=4	2,481389578	5,484	2,104
Q32P28	Prolyl 3-hydroxylase 1 OS=Homo sapiens OX=9606 GN=P3H1 PE=1 SV=2	0,716845878	5,484	6,743
P02649	Apolipoprotein E OS=Homo sapiens OX=9606 GN=APOE PE=1 SV=1	4,694835681	3,098	0,99
O43847	Nardilysin OS=Homo sapiens OX=9606 GN=NRDC PE=1 SV=3	1,801801802	5,424	3,824
Q9BTW9	Tubulin-specific chaperone D OS=Homo sapiens OX=9606 GN=TBCD PE=1 SV=2	1,992031873	5,372	3,068
P55285	Cadherin-6 OS=Homo sapiens OX=9606 GN=CDH6 PE=1 SV=1	4,62962963	5,604	1,453
Q8IVL6	Prolyl 3-hydroxylase 3 OS=Homo sapiens OX=9606 GN=P3H3 PE=1 SV=1	0,452898551	5,284	4,932
O00299	Chloride intracellular channel protein 1 OS=Homo sapiens OX=9606 GN=CLIC1 PE=1 SV=4	1,18623962	5,251	4,378
P45973	Chromobox protein homolog 5 OS=Homo sapiens OX=9606 GN=CBX5 PE=1 SV=1	4,504504505	3,491	1,041
P11766	Alcohol dehydrogenase class-3 OS=Homo sapiens OX=9606 GN=ADH5 PE=1 SV=4	2,352941176	5,136	2,187
P35580	Myosin-10 OS=Homo sapiens OX=9606 GN=MYH10 PE=1 SV=3	4,444444444	3,489	0,786
P04406	Glyceraldehyde-3-phosphate dehydrogenase OS=Homo sapiens OX=9606 GN=GAPDH PE=1 SV=3	1,138952164	5,057	6,508
Q9P2D1	Chromodomain-helicase-DNA-binding protein 7 OS=Homo sapiens OX=9606 GN=CHD7 PE=1 SV=3	4,347826087	2,556	0,831
Q9HC35	Echinoderm microtubule-associated protein-like 4 OS=Homo sapiens OX=9606 GN=EML4 PE=1 SV=3	1,883239171	4,956	2,628
Q9BVA1	Tubulin beta-2B chain OS=Homo sapiens OX=9606 GN=TUBB2B PE=1 SV=1	4,201680672	6,357	1,614
Q9Y383	Putative RNA-binding protein Luc7-like 2 OS=Homo sapiens OX=9606 GN=LUC7L2 PE=1 SV=2	4,201680672	2,054	0,581
P49916	DNA ligase 3 OS=Homo sapiens OX=9606 GN=LIG3 PE=1 SV=2	4,184100418	2,131	0,588
Q8NF37	Lysophosphatidylcholine acyltransferase 1 OS=Homo sapiens OX=9606 GN=LPCAT1 PE=1 SV=2	3,968253968	3,545	0,994
Q9BRX8	Peroxiredoxin-like 2A OS=Homo sapiens OX=9606 GN=PRXL2A PE=1 SV=3	142,8571429	4,622	0,041
P24666	Low molecular weight phosphotyrosine protein phosphatase OS=Homo sapiens OX=9606 GN=ACP1 PE=1 SV=3	3,968253968	2,901	0,687
O15347	High mobility group protein B3 OS=Homo sapiens OX=9606 GN=HMGB3 PE=1 SV=4	3,937007874	5,672	1,344
Q9Y5X3	Sorting nexin-5 OS=Homo sapiens OX=9606 GN=SNX5 PE=1 SV=1	3,891050584	2,235	0,68
P00390	Glutathione reductase, mitochondrial OS=Homo sapiens OX=9606 GN=GSR PE=1 SV=2	2,242152466	4,372	2,119
P78310	Coxsackievirus and adenovirus receptor OS=Homo sapiens OX=9606 GN=CXADR PE=1 SV=1	2,659574468	4,341	0,247
P10155	RNA-binding protein RO60 OS=Homo sapiens OX=9606 GN=RO60 PE=1 SV=2	1,930501931	4,314	2,357
Q9NTK5	Obg-like ATPase 1 OS=Homo sapiens OX=9606 GN=OLA1 PE=1 SV=2	3,816793893	2,155	0,614
Q01433	AMP deaminase 2 OS=Homo sapiens OX=9606 GN=AMPD2 PE=1 SV=3	1,85528757	4,16	1,157
P28074	Proteasome subunit beta type-5 OS=Homo sapiens OX=9606 GN=PSMB5 PE=1 SV=3	3,802281369	2,741	0,78
Q14195	Dihydropyrimidinase-related protein 3 OS=Homo sapiens OX=9606 GN=DPYSL3 PE=1 SV=1	0,229568411	4,095	16,599
P09467	Fructose-1,6-bisphosphatase 1 OS=Homo sapiens OX=9606 GN=FBP1 PE=1 SV=5	0,44603033	4,091	17,006
P07108	Acyl-CoA-binding protein OS=Homo sapiens OX=9606 GN=DBI PE=1 SV=2	1,926782274	4,073	3,601
Q14257	Reticulocalbin-2 OS=Homo sapiens OX=9606 GN=RCN2 PE=1 SV=1	3,787878788	4,063	1,066
O00264	Membrane-associated progesterone receptor component 1 OS=Homo sapiens OX=9606 GN=PGRMC1 PE=1 SV=3	3,773584906	3,455	0,895
Q9H1B7	Probable E3 ubiquitin-protein ligase IRF2BPL OS=Homo sapiens OX=9606 GN=IRF2BPL PE=1 SV=1	1,824817518	3,995	2,212
Q8WVX9	Fatty acyl-CoA reductase 1 OS=Homo sapiens OX=9606 GN=FAR1 PE=1 SV=1	3,773584906	3,137	0,798
Q07954	Prolow-density lipoprotein receptor-related protein 1 OS=Homo sapiens OX=9606 GN=LRP1 PE=1 SV=2	0,391696044	3,941	8,319
A1X283	SH3 and PX domain-containing protein 2B OS=Homo sapiens OX=9606 GN=SH3PXD2B PE=1 SV=3	1,908396947	3,923	1,684
P61163	Alpha-centractin OS=Homo sapiens OX=9606 GN=ACTR1A PE=1 SV=1	1,082251082	3,9	3,765
P52701	DNA mismatch repair protein Msh6 OS=Homo sapiens OX=9606 GN=MSH6 PE=1 SV=2	3,773584906	2,247	0,532
P13639	Elongation factor 2 OS=Homo sapiens OX=9606 GN=EEF2 PE=1 SV=4	1,550387597	3,803	2,313
P11717	Cation-independent mannose-6-phosphate receptor OS=Homo sapiens OX=9606 GN=IGF2R PE=1 SV=3	0,664010624	3,781	6,052
P20020	Plasma membrane calcium-transporting ATPase 1 OS=Homo sapiens OX=9606 GN=ATP2B1 PE=1 SV=4	0,64977258	3,758	6,154
Q13085	Acetyl-CoA carboxylase 1 OS=Homo sapiens OX=9606 GN=ACACA PE=1 SV=2	3,759398496	4,605	0,836
P63241	Eukaryotic translation initiation factor 5A-1 OS=Homo sapiens OX=9606 GN=EIF5A PE=1 SV=2	3,703703704	3,008	1,039
P62266	Small ribosomal subunit protein uS12 OS=Homo sapiens OX=9606 GN=RPS23 PE=1 SV=3	3,676470588	2,808	0,786
P45974	Ubiquitin carboxyl-terminal hydrolase 5 OS=Homo sapiens OX=9606 GN=USP5 PE=1 SV=2	1,811594203	3,702	1,804
P53634	Dipeptidyl peptidase 1 OS=Homo sapiens OX=9606 GN=CTSC PE=1 SV=2	3,649635036	4,167	1,971
Q8WZA9	Immunity-related GTPase family Q protein OS=Homo sapiens OX=9606 GN=IRGQ PE=1 SV=1	1,751313485	3,695	1,966
P14324	Farnesyl pyrophosphate synthase OS=Homo sapiens OX=9606 GN=FDPS PE=1 SV=4	3,623188406	4,836	1,363
P53999	Activated RNA polymerase II transcriptional coactivator p15 OS=Homo sapiens OX=9606 GN=SUB1 PE=1 SV=3	1,572327044	3,681	2,431
P60174	Triosephosphate isomerase OS=Homo sapiens OX=9606 GN=TPI1 PE=1 SV=4	1,414427157	3,679	2,756
Q86VP6	Cullin-associated NEDD8-dissociated protein 1 OS=Homo sapiens OX=9606 GN=CAND1 PE=1 SV=2	1,818181818	3,661	2,326
Q15813	Tubulin-specific chaperone E OS=Homo sapiens OX=9606 GN=TBCE PE=1 SV=1	2,680965147	3,654	
P14735	Insulin-degrading enzyme OS=Homo sapiens OX=9606 GN=IDE PE=1 SV=4	0,991080278	3,651	3,431
Q96AC1	Fermitin family homolog 2 OS=Homo sapiens OX=9606 GN=FERMT2 PE=1 SV=1	0,907441016	3,649	3,928
P37268	Squalene synthase OS=Homo sapiens OX=9606 GN=FDFT1 PE=1 SV=1	9,433962264	3,622	0,326
Q15582	Transforming growth factor-beta-induced protein ig-h3 OS=Homo sapiens OX=9606 GN=TGFBI PE=1 SV=1	3,610108303	5,99	1,565
Q15785	Mitochondrial import receptor subunit TOM34 OS=Homo sapiens OX=9606 GN=TOMM34 PE=1 SV=2	3,558718861	2,399	0,755
Q9BRP8	Partner of Y14 and mago OS=Homo sapiens OX=9606 GN=PYM1 PE=1 SV=1	1,686340641	3,593	2,04
Q02880	DNA topoisomerase 2-beta OS=Homo sapiens OX=9606 GN=TOP2B PE=1 SV=3	3,533568905	2,149	0,625
Q9BZF1	Oxysterol-binding protein-related protein 8 OS=Homo sapiens OX=9606 GN=OSBPL8 PE=1 SV=3	1,154734411	3,582	3,257
P22314	Ubiquitin-like modifier-activating enzyme 1 OS=Homo sapiens OX=9606 GN=UBA1 PE=1 SV=3	1,494768311	3,562	2,398
P43487	Ran-specific GTPase-activating protein OS=Homo sapiens OX=9606 GN=RANBP1 PE=1 SV=1	3,521126761	2,975	1,059
P18669	Phosphoglycerate mutase 1 OS=Homo sapiens OX=9606 GN=PGAM1 PE=1 SV=2	1,207729469	3,52	3,175
O15355	Protein phosphatase 1G OS=Homo sapiens OX=9606 GN=PPM1G PE=1 SV=1	3,50877193	2,153	0,613
P16949	Stathmin OS=Homo sapiens OX=9606 GN=STMN1 PE=1 SV=3	3,484320557	4,946	1,388
Q9Y617	Phosphoserine aminotransferase OS=Homo sapiens OX=9606 GN=PSAT1 PE=1 SV=2	0,941619586	3,486	3,336
P98172	Ephrin-B1 OS=Homo sapiens OX=9606 GN=EFNB1 PE=1 SV=1	3,484320557	2,495	1,749
Q9HAV4	Exportin-5 OS=Homo sapiens OX=9606 GN=XPO5 PE=1 SV=1	3,472222222	6,008	1,6
Q8TEX9	Importin-4 OS=Homo sapiens OX=9606 GN=IPO4 PE=1 SV=2	3,460207612	2,92	0,695
O00410	Importin-5 OS=Homo sapiens OX=9606 GN=IPO5 PE=1 SV=4	1,945525292	3,429	1,534
Q13418	Integrin-linked protein kinase OS=Homo sapiens OX=9606 GN=ILK PE=1 SV=2	0,629326621	3,425	5,766
Q3LXA3	Triokinase/FMN cyclase OS=Homo sapiens OX=9606 GN=TKFC PE=1 SV=2	1,976284585	3,418	2,323
P10768	S-formylglutathione hydrolase OS=Homo sapiens OX=9606 GN=ESD PE=1 SV=2	1,27388535	3,385	1,978

Q9P0M6	Core histone macro-H2A.2 OS=Homo sapiens OX=9606 GN=MACROH2A2 PE=1 SV=3	3,378378378	2,218	0,671
Q07065	Cytoskeleton-associated protein 4 OS=Homo sapiens OX=9606 GN=CKAP4 PE=1 SV=2	0,469483568	3,353	6,667
Q96SB4	SRSF protein kinase 1 OS=Homo sapiens OX=9606 GN=SRPK1 PE=1 SV=2	3,367003367	2,775	0,612
O60341	Lysine-specific histone demethylase 1A OS=Homo sapiens OX=9606 GN=KDM1A PE=1 SV=2	3,25732899	3,614	1,256
O75347	Tubulin-specific chaperone A OS=Homo sapiens OX=9606 GN=TBCA PE=1 SV=3	3,236245955	5,711	1,741
Q96P70	Importin-9 OS=Homo sapiens OX=9606 GN=IPO9 PE=1 SV=3	1,468428781	3,326	2,14
P23526	Adenosylhomocysteinase OS=Homo sapiens OX=9606 GN=AHCY PE=1 SV=4	3,215434084	2,05	0,649
Q9H074	Polyadenylate-binding protein-interacting protein 1 OS=Homo sapiens OX=9606 GN=PAIP1 PE=1 SV=1	1,569858713	3,293	1,817
P30086	Phosphatidylethanolamine-binding protein 1 OS=Homo sapiens OX=9606 GN=PEBP1 PE=1 SV=3	3,144654088	3,688	1,238
P23921	Ribonucleoside-diphosphate reductase large subunit OS=Homo sapiens OX=9606 GN=RRM1 PE=1 SV=1	3,115264798	3,619	0,948
P49321	Nuclear autoantigenic sperm protein OS=Homo sapiens OX=9606 GN=NASP PE=1 SV=2	3,095975232	2,788	0,999
O00429	Dynamin-1-like protein OS=Homo sapiens OX=9606 GN=DNM1L PE=1 SV=2	1,272264631	3,213	2,778
P08243	Asparagine synthetase [glutamine-hydrolyzing] OS=Homo sapiens OX=9606 GN=ASNS PE=1 SV=4	1,19047619	3,17	2,72
Q8N8S7	Protein enabled homolog OS=Homo sapiens OX=9606 GN=ENAH PE=1 SV=2	0,927643785	3,158	3,464
Q14204	Cytoplasmic dynein 1 heavy chain 1 OS=Homo sapiens OX=9606 GN=DYNC1H1 PE=1 SV=5	1,736111111	3,143	1,892
P11413	Glucose-6-phosphate 1-dehydrogenase OS=Homo sapiens OX=9606 GN=G6PD PE=1 SV=4	0,548546352	3,141	4,715
P20674	Cytochrome c oxidase subunit 5A, mitochondrial OS=Homo sapiens OX=9606 GN=COX5A PE=1 SV=2	3,086419753	2,017	0,897
Q9Y4L1	Hypoxia up-regulated protein 1 OS=Homo sapiens OX=9606 GN=HYOU1 PE=1 SV=1	1,248439451	3,135	2,331
O60701	UDP-glucose 6-dehydrogenase OS=Homo sapiens OX=9606 GN=UGDH PE=1 SV=1	1,430615165	3,129	2,275
Q00169	Phosphatidylinositol transfer protein alpha isoform OS=Homo sapiens OX=9606 GN=PITPNA PE=1 SV=2	3,076923077	3,236	0,929
P51003	Poly(A) polymerase alpha OS=Homo sapiens OX=9606 GN=PAPOLA PE=1 SV=4	3,067484663	2,665	0,997
Q15181	Inorganic pyrophosphatase OS=Homo sapiens OX=9606 GN=PPA1 PE=1 SV=2	1,956947162	3,092	1,514
P46926	Glucosamine-6-phosphate isomerase 1 OS=Homo sapiens OX=9606 GN=GNPDA1 PE=1 SV=1	1,795332136	3,085	1,288
P54577	Tyrosine--tRNA ligase, cytoplasmic OS=Homo sapiens OX=9606 GN=YARS1 PE=1 SV=4	1,216545012	3,067	2,342
Q14676	Mediator of DNA damage checkpoint protein 1 OS=Homo sapiens OX=9606 GN=MDC1 PE=1 SV=3	3,048780488	2,327	0,805
Q9UPN3	Microtubule-actin cross-linking factor 1, isoforms 1/2/3/4/5 OS=Homo sapiens OX=9606 GN=MACF1 PE=1 SV=4	1,577287066	3,055	1,882
P46379	Large proline-rich protein BAG6 OS=Homo sapiens OX=9606 GN=BAG6 PE=1 SV=2	1,801801802	3,046	1,666
O95870	Phosphatidylserine lipase ABHD16A OS=Homo sapiens OX=9606 GN=ABHD16A PE=1 SV=3	0,819672131	3,025	3,684
P62736	Actin, aortic smooth muscle OS=Homo sapiens OX=9606 GN=ACTA2 PE=1 SV=1	0,295159386	3,025	11,967
P53396	ATP-citrate synthase OS=Homo sapiens OX=9606 GN=ACLY PE=1 SV=3	3,021148036	4,718	1,641
Q02790	Peptidyl-prolyl cis-trans isomerase FKBP4 OS=Homo sapiens OX=9606 GN=FKBP4 PE=1 SV=3	3,003003003	3,347	1,063
Q15366	Poly(rC)-binding protein 2 OS=Homo sapiens OX=9606 GN=PCBP2 PE=1 SV=1	1,984126984	3,019	1,543
P06733	Alpha-enolase OS=Homo sapiens OX=9606 GN=ENO1 PE=1 SV=2	1,38121547	3,015	2,036
Q8N0X7	Spartin OS=Homo sapiens OX=9606 GN=SPART PE=1 SV=1	0,704225352	3,01	4,641
Q15102	Platelet-activating factor acetylhydrolase IB subunit alpha1 OS=Homo sapiens OX=9606 GN=PAFAH1B3 PE=1 SV=1	2,994011976	2,238	0,837
P31939	Bifunctional purine biosynthesis protein ATIC OS=Homo sapiens OX=9606 GN=ATIC PE=1 SV=3	2,976190476	2,161	0,794
P08648	Integrin alpha-5 OS=Homo sapiens OX=9606 GN=ITGA5 PE=1 SV=2	0,084260195	2,979	44,474
P50579	Methionine aminopeptidase 2 OS=Homo sapiens OX=9606 GN=METAP2 PE=1 SV=1	2,941176471	2,295	0,766
Q16643	Drebrin OS=Homo sapiens OX=9606 GN=DBN1 PE=1 SV=4	2,93255132	3,295	1,082
Q13126	S-methyl-5'-thioadenosine phosphorylase OS=Homo sapiens OX=9606 GN=MTAP PE=1 SV=2	0,989119683	2,938	2,82
O76003	Glutaredoxin-3 OS=Homo sapiens OX=9606 GN=GLRX3 PE=1 SV=2	1,602564103	2,937	1,485
Q14232	Translation initiation factor eIF2B subunit alpha OS=Homo sapiens OX=9606 GN=EIF2B1 PE=1 SV=1	1,356852103	2,929	1,522
P19174	1-phosphatidylinositol 4,5-bisphosphate phosphodiesterase gamma-1 OS=Homo sapiens OX=9606 GN=PLCG1 PE=1 SV=1	2,906976744	3,286	1,266
Q8IXT5	RNA-binding protein 12B OS=Homo sapiens OX=9606 GN=RBM12B PE=1 SV=2	2,89017341	2,248	0,948
P62899	Large ribosomal subunit protein eL31 OS=Homo sapiens OX=9606 GN=RPL31 PE=1 SV=1	2,88184438	2,976	0,686
P26358	DNA (cytosine-5)-methyltransferase 1 OS=Homo sapiens OX=9606 GN=DNMT1 PE=1 SV=2	2,873563218	2,379	1,354
Q9Y2Z0	Protein SGT1 homolog OS=Homo sapiens OX=9606 GN=SUGT1 PE=1 SV=3	2,865329513	2,265	0,871
O43347	RNA-binding protein Musashi homolog 1 OS=Homo sapiens OX=9606 GN=MSI1 PE=1 SV=1	2,857142857	7,442	0,536
Q13576	Ras GTPase-activating-like protein IQGAP2 OS=Homo sapiens OX=9606 GN=IQGAP2 PE=1 SV=4	1,960784314	2,883	1,338
Q96KA5	Lipid scramblase CLPTM1L OS=Homo sapiens OX=9606 GN=CLPTM1L PE=1 SV=1	1,027749229	2,879	2,39
Q9UBB4	Ataxin-10 OS=Homo sapiens OX=9606 GN=ATXN10 PE=1 SV=1	2,857142857	2,658	0,966
P52272	Heterogeneous nuclear ribonucleoprotein M OS=Homo sapiens OX=9606 GN=HNRNPM PE=1 SV=3	1,168224299	2,875	2,229
P17812	CTP synthase 1 OS=Homo sapiens OX=9606 GN=CTPS1 PE=1 SV=2	2,849002849	3,701	1,285
Q9UK76	Jupiter microtubule associated homolog 1 OS=Homo sapiens OX=9606 GN=JPT1 PE=1 SV=3	1,531393568	2,826	1,369
P21333	Filamin-A OS=Homo sapiens OX=9606 GN=FLNA PE=1 SV=4	0,62774639	2,822	4,008
P13667	Protein disulfide-isomerase A4 OS=Homo sapiens OX=9606 GN=PDIA4 PE=1 SV=2	1,408450704	2,818	1,847
Q9HB71	Calcyclin-binding protein OS=Homo sapiens OX=9606 GN=CACYBP PE=1 SV=2	2,840909091	2,286	0,962
Q13867	Bleomycin hydrolase OS=Homo sapiens OX=9606 GN=BLMH PE=1 SV=1	2,816901408	2,911	1,259
P51532	Transcription activator BRG1 OS=Homo sapiens OX=9606 GN=SMARCA4 PE=1 SV=2	2,808988764	2,094	0,666
E9PAV3	Nascent polypeptide-associated complex subunit alpha, muscle-specific form OS=Homo sapiens OX=9606 GN=NACA PE=1 S	2,801120448	2,668	1,036
P49591	Serine--tRNA ligase, cytoplasmic OS=Homo sapiens OX=9606 GN=SARS1 PE=1 SV=3	2,770083102	5,134	1,833
Q13177	Serine/threonine-protein kinase PAK 2 OS=Homo sapiens OX=9606 GN=PAK2 PE=1 SV=3	1,543209877	2,806	1,53
P48723	Heat shock 70 kDa protein 13 OS=Homo sapiens OX=9606 GN=HSPA13 PE=1 SV=1	1,335113485	2,803	2,713
Q14203	Dynactin subunit 1 OS=Homo sapiens OX=9606 GN=DCTN1 PE=1 SV=3	1,168224299	2,79	2,187
P22234	Bifunctional phosphoribosylaminoimidazole carboxylase/phosphoribosylaminoimidazole succinocarboxamide synthetase O	2,762430939	2,635	0,919
Q9Y266	Nuclear migration protein nudC OS=Homo sapiens OX=9606 GN=NUDC PE=1 SV=1	2,762430939	2,581	0,848
P61088	Ubiquitin-conjugating enzyme E2 N OS=Homo sapiens OX=9606 GN=UBE2N PE=1 SV=1	1,858736059	2,748	1,453
Q06210	Glutamine--fructose-6-phosphate aminotransferase [isomerizing] 1 OS=Homo sapiens OX=9606 GN=GFPT1 PE=1 SV=3	1,838235294	2,744	1,435
Q14141	Septin-6 OS=Homo sapiens OX=9606 GN=SEPTIN6 PE=1 SV=4	2,747252747	5,865	1,486
P08758	Annexin A5 OS=Homo sapiens OX=9606 GN=ANXA5 PE=1 SV=2	0,837520938	2,732	2,884
Q8IWZ3	Ankyrin repeat and KH domain-containing protein 1 OS=Homo sapiens OX=9606 GN=ANKHD1 PE=1 SV=1	1,757469244	2,722	1,288
P11047	Laminin subunit gamma-1 OS=Homo sapiens OX=9606 GN=LAMC1 PE=1 SV=3	0,851788756	2,708	3,05
A0A669KBH5	Alpha-synuclein OS=Homo sapiens OX=9606 GN=SNCA PE=1 SV=1	0,27578599	2,696	2,975
Q13409	Cytoplasmic dynein 1 intermediate chain 2 OS=Homo sapiens OX=9606 GN=DYNC1I2 PE=1 SV=3	1,655629139	2,689	1,403
Q7Z460	CLIP-associating protein 1 OS=Homo sapiens OX=9606 GN=CLASP1 PE=1 SV=1	2,747252747	2,099	0,718
O43252	Bifunctional 3'-phosphoadenosine 5'-phosphosulfate synthase 1 OS=Homo sapiens OX=9606 GN=PAPSS1 PE=1 SV=2	2,739726027	2,302	0,896
P42330	Aldo-keto reductase family 1 member C3 OS=Homo sapiens OX=9606 GN=AKR1C3 PE=1 SV=4	0,384024578	2,68	2,949
P23634	Plasma membrane calcium-transporting ATPase 4 OS=Homo sapiens OX=9606 GN=ATP2B4 PE=1 SV=2	0,242777373	2,68	10,937
Q8NBF2	NHL repeat-containing protein 2 OS=Homo sapiens OX=9606 GN=NHLRC2 PE=1 SV=1	2,72479564	7,052	1,795
Q8TDI0	Chromodomain-helicase-DNA-binding protein 5 OS=Homo sapiens OX=9606 GN=CHD5 PE=1 SV=1	2,702702703	3,277	1,004
Q9H2P0	Activity-dependent neuroprotector homeobox protein OS=Homo sapiens OX=9606 GN=ADNP PE=1 SV=1	2,666666667	2,91	1,163
O75962	Triple functional domain protein OS=Homo sapiens OX=9606 GN=TRIO PE=1 SV=2	0,522739153	2,653	5,152
Q9UI12	V-type proton ATPase subunit H OS=Homo sapiens OX=9606 GN=ATP6V1H PE=1 SV=1	1,133786848	2,648	2,12

A0A6Q8PFU3	Platelet-activating factor acetylhydrolase IB subunit alpha OS=Homo sapiens OX=9606 GN=PAFAH1B1 PE=1 SV=1	1,964636542	2,645	1,243
P35052	Glypican-1 OS=Homo sapiens OX=9606 GN=GPC1 PE=1 SV=2	0,147579693	2,642	16,466
P62826	GTP-binding nuclear protein Ran OS=Homo sapiens OX=9606 GN=RAN PE=1 SV=3	2,645502646	2,437	0,864
Q14165	Malectin OS=Homo sapiens OX=9606 GN=MLEC PE=1 SV=1	1,236093943	2,631	2,167
P11441	Ubiquitin-like protein 4A OS=Homo sapiens OX=9606 GN=UBL4A PE=1 SV=1	1,335113485	2,624	1,614
P27695	DNA-(apurinic or apyrimidinic site) endonuclease OS=Homo sapiens OX=9606 GN=APEX1 PE=1 SV=2	2,638522427	2,682	0,774
Q7Z6Z7	E3 ubiquitin-protein ligase HUWE1 OS=Homo sapiens OX=9606 GN=HUWE1 PE=1 SV=3	1,968503937	2,608	1,359
P07737	Profilin-1 OS=Homo sapiens OX=9606 GN=PFN1 PE=1 SV=2	1,300390117	2,607	1,979
P22102	Trifunctional purine biosynthetic protein adenosine-3 OS=Homo sapiens OX=9606 GN=GART PE=1 SV=1	2,638522427	2,38	0,841
P00491	Purine nucleoside phosphorylase OS=Homo sapiens OX=9606 GN=PNP PE=1 SV=2	2,638522427	2,234	0,673
O75534	Cold shock domain-containing protein E1 OS=Homo sapiens OX=9606 GN=CSDE1 PE=1 SV=2	2,631578947	2,024	0,776
P09874	Poly [ADP-ribose] polymerase 1 OS=Homo sapiens OX=9606 GN=PARP1 PE=1 SV=4	5,847953216	2,555	0,466
P50395	Rab GDP dissociation inhibitor beta OS=Homo sapiens OX=9606 GN=GDI2 PE=1 SV=2	1,941747573	2,551	1,276
P51531	Probable global transcription activator SNF2L2 OS=Homo sapiens OX=9606 GN=SMARCA2 PE=1 SV=2	2,610966057	2,988	0,836
Q8WW12	PEST proteolytic signal-containing nuclear protein OS=Homo sapiens OX=9606 GN=PCNP PE=1 SV=2	1,721170396	2,523	1,471
P38606	V-type proton ATPase catalytic subunit A OS=Homo sapiens OX=9606 GN=ATP6V1A PE=1 SV=2	1,501501502	2,497	1,645
Q13308	Inactive tyrosine-protein kinase 7 OS=Homo sapiens OX=9606 GN=PTK7 PE=1 SV=2	0,676589986	2,497	4,082
O95433	Activator of 90 kDa heat shock protein ATPase homolog 1 OS=Homo sapiens OX=9606 GN=AHSA1 PE=1 SV=1	2,610966057	2,685	1,264
Q9BYT8	Neurolysin, mitochondrial OS=Homo sapiens OX=9606 GN=NLN PE=1 SV=1	2,597402597	2,423	0,845
P35527	Keratin, type I cytoskeletal 9 OS=Homo sapiens OX=9606 GN=KRT9 PE=1 SV=3	0,900900901	2,482	2,729
Q9BW19	Kinesin-like protein KIFC1 OS=Homo sapiens OX=9606 GN=KIFC1 PE=1 SV=2	1,254705144	2,48	2,23
P43034	Platelet-activating factor acetylhydrolase IB subunit beta OS=Homo sapiens OX=9606 GN=PAFAH1B1 PE=1 SV=2	1,923076923	2,474	1,289
P27348	14-3-3 protein theta OS=Homo sapiens OX=9606 GN=YWHAQ PE=1 SV=1	1,865671642	2,45	1,288
Q06830	Peroxiredoxin-1 OS=Homo sapiens OX=9606 GN=PRDX1 PE=1 SV=1	2,577319588	3,022	1,22
Q93052	Lipoma-preferred partner OS=Homo sapiens OX=9606 GN=LPP PE=1 SV=1	0,532481363	2,441	4,195
B7ZLQ5	SMARCA1 protein OS=Homo sapiens OX=9606 GN=SMARCA1 PE=1 SV=1	2,570694087	4,033	1,513
Q13162	Peroxiredoxin-4 OS=Homo sapiens OX=9606 GN=PRDX4 PE=1 SV=1	0,861326443	2,436	2,212
Q14315	Filamin-C OS=Homo sapiens OX=9606 GN=FLNC PE=1 SV=3	1,526717557	2,426	1,565
P27708	Multifunctional protein CAD OS=Homo sapiens OX=9606 GN=CAD PE=1 SV=3	2,557544757	5,145	1,942
Q9Y696	Chloride intracellular channel protein 4 OS=Homo sapiens OX=9606 GN=CLIC4 PE=1 SV=4	2,53164557	2,809	1,239
P63104	14-3-3 protein zeta/delta OS=Homo sapiens OX=9606 GN=YWHAZ PE=1 SV=1	1,179245283	2,402	1,988
Q9Y3F4	Serine-threonine kinase receptor-associated protein OS=Homo sapiens OX=9606 GN=STRAP PE=1 SV=1	2,506265664	3,063	1,329
Q99497	Parkinson disease protein 7 OS=Homo sapiens OX=9606 GN=PARK7 PE=1 SV=2	1,930501931	2,396	1,207
Q05682	Caldesmon OS=Homo sapiens OX=9606 GN=CALD1 PE=1 SV=3	0,218531469	2,396	10,126
P30101	Protein disulfide-isomerase A3 OS=Homo sapiens OX=9606 GN=PDIA3 PE=1 SV=4	0,726216412	2,39	3,539
P20290	Transcription factor BTF3 OS=Homo sapiens OX=9606 GN=BTF3 PE=1 SV=1	1,972386588	2,388	0,999
P07942	Laminin subunit beta-1 OS=Homo sapiens OX=9606 GN=LAMB1 PE=1 SV=2	0,959692898	2,388	2,547
P52788	Spermine synthase OS=Homo sapiens OX=9606 GN=SMS PE=1 SV=2	1,179245283	2,386	2,153
P31153	S-adenosylmethionine synthase isoform type-2 OS=Homo sapiens OX=9606 GN=MAT2A PE=1 SV=1	1,972386588	2,384	1,115
E7EPK1	Septin OS=Homo sapiens OX=9606 GN=SEPTIN7 PE=1 SV=2	2,493765586	3,588	1,167
Q96T88	E3 ubiquitin-protein ligase UHRF1 OS=Homo sapiens OX=9606 GN=UHRF1 PE=1 SV=1	2,487562189	2,139	0,84
P09429	High mobility group protein B1 OS=Homo sapiens OX=9606 GN=HMGB1 PE=1 SV=3	2,475247525	2,567	1,04
Q01518	Adenylyl cyclase-associated protein 1 OS=Homo sapiens OX=9606 GN=CAP1 PE=1 SV=5	0,782472613	2,378	2,634
P12955	Xaa-Pro dipeptidase OS=Homo sapiens OX=9606 GN=PEPD PE=1 SV=3	1,410437236	2,374	1,092
P49902	Cytosolic purine 5'-nucleotidase OS=Homo sapiens OX=9606 GN=NT5C2 PE=1 SV=1	1,009081736	2,372	1,941
P80303	Nucleobindin-2 OS=Homo sapiens OX=9606 GN=NUCB2 PE=1 SV=3	0,366703337	2,372	7,102
Q5SW79	Centrosomal protein of 170 kDa OS=Homo sapiens OX=9606 GN=CEP170 PE=1 SV=1	1,248439451	2,371	1,723
P52565	Rho GDP-dissociation inhibitor 1 OS=Homo sapiens OX=9606 GN=ARHGDIA PE=1 SV=3	1,096491228	2,37	2,108
Q09028	Histone-binding protein RBBP4 OS=Homo sapiens OX=9606 GN=RBBP4 PE=1 SV=3	2,433090024	5,9	1,613
Q13404	Ubiquitin-conjugating enzyme E2 variant 1 OS=Homo sapiens OX=9606 GN=UBE2V1 PE=1 SV=2	1,776198934	2,363	1,328
Q96AY3	Peptidyl-prolyl cis-trans isomerase FKBP10 OS=Homo sapiens OX=9606 GN=FKBP10 PE=1 SV=1	0,488758553	2,363	4,491
P19022	Cadherin-2 OS=Homo sapiens OX=9606 GN=CDH2 PE=1 SV=4	0,379650721	2,363	11,598
O75955	Flotillin-1 OS=Homo sapiens OX=9606 GN=FLOT1 PE=1 SV=3	0,693962526	2,361	3,647
Q15363	Transmembrane emp24 domain-containing protein 2 OS=Homo sapiens OX=9606 GN=TMED2 PE=1 SV=1	0,803858521	2,359	2,878
O00461	Golgi integral membrane protein 4 OS=Homo sapiens OX=9606 GN=GOLIM4 PE=1 SV=1	0,910746812	2,358	2,378
O60271	C-Jun-amino-terminal kinase-interacting protein 4 OS=Homo sapiens OX=9606 GN=SPAG9 PE=1 SV=4	0,715819613	2,357	3,267
Q9UNH7	Sorting nexin-6 OS=Homo sapiens OX=9606 GN=SNX6 PE=1 SV=1	0,825082508	2,337	3,075
P41250	Glycine--tRNA ligase OS=Homo sapiens OX=9606 GN=GARS1 PE=1 SV=3	0,897666068	2,335	2,327
Q13561	Dynactin subunit 2 OS=Homo sapiens OX=9606 GN=DCTN2 PE=1 SV=4	1,088139282	2,329	2,26
Q8N1G4	Leucine-rich repeat-containing protein 47 OS=Homo sapiens OX=9606 GN=LRRC47 PE=1 SV=1	2,421307506	2,025	0,787
Q9BQ39	ATP-dependent RNA helicase DDX50 OS=Homo sapiens OX=9606 GN=DDX50 PE=1 SV=1	2,415458937	2,001	0,858
Q9UBF2	Coatomer subunit gamma-2 OS=Homo sapiens OX=9606 GN=COPG2 PE=1 SV=1	1,677852349	2,32	1,374
O00625	Pirin OS=Homo sapiens OX=9606 GN=PIR PE=1 SV=1	2,403846154	5,021	1,958
Q9BTT0	Acidic leucine-rich nuclear phosphoprotein 32 family member E OS=Homo sapiens OX=9606 GN=ANP32E PE=1 SV=1	2,392344498	2,491	1,119
Q96FW1	Ubiquitin thioesterase OTUB1 OS=Homo sapiens OX=9606 GN=OTUB1 PE=1 SV=2	2,364066194	2,875	1,152
Q9Y625	Glypican-6 OS=Homo sapiens OX=9606 GN=GPC6 PE=1 SV=1	0,539083558	2,285	4,722
P15170	Eukaryotic peptide chain release factor GTP-binding subunit ERF3A OS=Homo sapiens OX=9606 GN=GSPT1 PE=1 SV=1	1,179245283	2,28	2,405
Q15645	Pachytene checkpoint protein 2 homolog OS=Homo sapiens OX=9606 GN=TRIP13 PE=1 SV=2	2,34741784	3,34	1,512
Q13895	Bystin OS=Homo sapiens OX=9606 GN=BYSL PE=1 SV=3	2,341920375	2,265	1,327
Q9H9A6	Leucine-rich repeat-containing protein 40 OS=Homo sapiens OX=9606 GN=LRRC40 PE=1 SV=1	2,314814815	3,703	1,657
O14497	AT-rich interactive domain-containing protein 1A OS=Homo sapiens OX=9606 GN=ARID1A PE=1 SV=3	2,288329519	2,38	1,155
Q9P032	NADH dehydrogenase [ubiquinone] 1 alpha subcomplex assembly factor 4 OS=Homo sapiens OX=9606 GN=NDUFAF4 PE=1 S	1,169590643	2,254	1,113
P41240	Tyrosine-protein kinase CSK OS=Homo sapiens OX=9606 GN=CSK PE=1 SV=1	2,272727273	3,833	1,242
Q15631	Translin OS=Homo sapiens OX=9606 GN=TSN PE=1 SV=1	2,272727273	2,106	0,924
Q15019	Septin-2 OS=Homo sapiens OX=9606 GN=SEPTIN2 PE=1 SV=1	1,416430595	2,245	1,631
Q96K17	Transcription factor BTF3 homolog 4 OS=Homo sapiens OX=9606 GN=BTF3L4 PE=1 SV=1	2,237136465	2,369	0,774
Q13242	Serine/arginine-rich splicing factor 9 OS=Homo sapiens OX=9606 GN=SRSF9 PE=1 SV=1	2,232142857	2,1	1,041
Q9Y6G9	Cytoplasmic dynein 1 light intermediate chain 1 OS=Homo sapiens OX=9606 GN=DYNC1LI1 PE=1 SV=3	2,227171492	3,753	1,945
Q99816	Tumor susceptibility gene 101 protein OS=Homo sapiens OX=9606 GN=TSG101 PE=1 SV=2	1,215066829	2,23	2,182
P78371	T-complex protein 1 subunit beta OS=Homo sapiens OX=9606 GN=CCT2 PE=1 SV=4	1,620745543	2,223	1,193
P80723	Brain acid soluble protein 1 OS=Homo sapiens OX=9606 GN=BASP1 PE=1 SV=2	0,559284116	2,223	3,586
P62937	Peptidyl-prolyl cis-trans isomerase A OS=Homo sapiens OX=9606 GN=PPIA PE=1 SV=2	1,792114695	2,222	1,418
Q969H8	Myeloid-derived growth factor OS=Homo sapiens OX=9606 GN=MYDGF PE=1 SV=1	0,362976407	2,222	6,364

Q9Y6E2	eIF5-mimic protein 1 OS=Homo sapiens OX=9606 GN=BZW2 PE=1 SV=1	2,197802198	4,131	1,745
Q9NTZ6	RNA-binding protein 12 OS=Homo sapiens OX=9606 GN=RBM12 PE=1 SV=1	1,84501845	2,215	1,142
Q709C8	Intermembrane lipid transfer protein VPS13C OS=Homo sapiens OX=9606 GN=VPS13C PE=1 SV=1	1,838235294	2,215	3,041
O95373	Importin-7 OS=Homo sapiens OX=9606 GN=IPO7 PE=1 SV=1	1,675041876	2,205	1,326
P61254	Large ribosomal subunit protein uL24 OS=Homo sapiens OX=9606 GN=RPL26 PE=1 SV=1	1,930501931	2,196	1,014
O43852	Calumenin OS=Homo sapiens OX=9606 GN=CALU PE=1 SV=2	0,471920717	2,193	4,432
O14617	AP-3 complex subunit delta-1 OS=Homo sapiens OX=9606 GN=AP3D1 PE=1 SV=1	1,602564103	2,19	1,434
P06703	Protein S100-A6 OS=Homo sapiens OX=9606 GN=S100A6 PE=1 SV=1	0,078883016	2,181	27,387
Q15165	Serum paraoxonase/arylesterase 2 OS=Homo sapiens OX=9606 GN=PON2 PE=1 SV=4	0,644745326	2,177	3,384
O75718	Cartilage-associated protein OS=Homo sapiens OX=9606 GN=CRTAP PE=1 SV=1	0,503018109	2,177	5,752
P41227	N-alpha-acetyltransferase 10 OS=Homo sapiens OX=9606 GN=NAA10 PE=1 SV=1	1,550387597	2,165	1,316
Q16537	Serine/threonine-protein phosphatase 2A 56 kDa regulatory subunit epsilon isoform OS=Homo sapiens OX=9606 GN=PPP2F	2,188183807	2,543	1,185
P23528	Cofilin-1 OS=Homo sapiens OX=9606 GN=CFL1 PE=1 SV=3	1,481481481	2,161	1,235
P49915	GMP synthase [glutamine-hydrolyzing] OS=Homo sapiens OX=9606 GN=GMPS PE=1 SV=1	1,675041876	2,16	1,523
Q08J23	RNA cytosine C(5)-methyltransferase NSUN2 OS=Homo sapiens OX=9606 GN=NSUN2 PE=1 SV=2	2,188183807	2,277	0,998
P23588	Eukaryotic translation initiation factor 4B OS=Homo sapiens OX=9606 GN=EIF4B PE=1 SV=2	2,183406114	2,324	0,806
P15531	Nucleoside diphosphate kinase A OS=Homo sapiens OX=9606 GN=NME1 PE=1 SV=1	1,715265866	2,152	1,504
P33176	Kinesin-1 heavy chain OS=Homo sapiens OX=9606 GN=KIF5B PE=1 SV=1	1,216545012	2,15	1,724
P11172	Uridine 5'-monophosphate synthase OS=Homo sapiens OX=9606 GN=UMPS PE=1 SV=1	2,169197397	2,067	0,94
Q9UMR2	ATP-dependent RNA helicase DDX19B OS=Homo sapiens OX=9606 GN=DDX19B PE=1 SV=1	1,402524544	2,148	1,582
Q16181	Septin-7 OS=Homo sapiens OX=9606 GN=SEPTIN7 PE=1 SV=2	2,164502165	2,815	1,235
Q7Z4H8	Protein O-glucosyltransferase 3 OS=Homo sapiens OX=9606 GN=POGLUT3 PE=1 SV=2	0,490677134	2,138	4,817
Q9UQ80	Proliferation-associated protein 2G4 OS=Homo sapiens OX=9606 GN=PA2G4 PE=1 SV=3	1,727115717	2,134	1,137
O14929	Histone acetyltransferase type B catalytic subunit OS=Homo sapiens OX=9606 GN=HAT1 PE=1 SV=2	2,150537634	2,402	1,256
P63151	Serine/threonine-protein phosphatase 2A 55 kDa regulatory subunit B alpha isoform OS=Homo sapiens OX=9606 GN=PPP2F	1,912045889	2,121	1,737
Q6FI81	Anamorsin OS=Homo sapiens OX=9606 GN=CIAPIN1 PE=1 SV=2	2,132196162	3,448	1,111
Q16513	Serine/threonine-protein kinase N2 OS=Homo sapiens OX=9606 GN=PKN2 PE=1 SV=1	2,114164905	2,914	1,134
Q14739	Delta(14)-sterol reductase LBR OS=Homo sapiens OX=9606 GN=LBR PE=1 SV=2	1,385041551	2,106	1,525
Q9HB40	Retinoid-inducible serine carboxypeptidase OS=Homo sapiens OX=9606 GN=SCPEP1 PE=1 SV=1	0,661375661	2,106	3,435
P14550	Aldo-keto reductase family 1 member A1 OS=Homo sapiens OX=9606 GN=AKR1A1 PE=1 SV=3	2,105263158	4,519	1,657
Q9NVA2	Septin-11 OS=Homo sapiens OX=9606 GN=SEPTIN11 PE=1 SV=3	2,092050209	3,124	1,551
P40925	Malate dehydrogenase, cytoplasmic OS=Homo sapiens OX=9606 GN=MDH1 PE=1 SV=4	1,968503937	2,096	1,179
A0A7I2V508	Large proline-rich protein BAG6 OS=Homo sapiens OX=9606 GN=BAG6 PE=1 SV=1	2,083333333	3,439	1,637
Q9NY33	Dipeptidyl peptidase 3 OS=Homo sapiens OX=9606 GN=DPP3 PE=1 SV=2	1,490312966	2,092	1,476
P50281	Matrix metalloproteinase-14 OS=Homo sapiens OX=9606 GN=MMP14 PE=1 SV=3	0,392772977	2,091	4,725
P62244	Small ribosomal subunit protein uS8 OS=Homo sapiens OX=9606 GN=RPS15A PE=1 SV=2	1,162790698	2,076	1,696
P43686	26S proteasome regulatory subunit 6B OS=Homo sapiens OX=9606 GN=PSMC4 PE=1 SV=2	1,340482574	2,075	1,28
P55011	Solute carrier family 12 member 2 OS=Homo sapiens OX=9606 GN=SLC12A2 PE=1 SV=1	0,773395205	2,075	2,815
Q9Y6Y8	SEC23-interacting protein OS=Homo sapiens OX=9606 GN=SEC23IP PE=1 SV=1	0,742390497	2,074	2,638
O00139	Kinesin-like protein KIF2A OS=Homo sapiens OX=9606 GN=KIF2A PE=1 SV=3	1,886792453	2,069	1,141
Q15691	Microtubule-associated protein RP/EB family member 1 OS=Homo sapiens OX=9606 GN=MAPRE1 PE=1 SV=3	2,079002079	2,007	0,809
Q9NXU5	ADP-ribosylation factor-like protein 15 OS=Homo sapiens OX=9606 GN=ARL15 PE=1 SV=1	1,285347044	2,063	1,639
Q96QK1	Vacuolar protein sorting-associated protein 35 OS=Homo sapiens OX=9606 GN=VPS35 PE=1 SV=2	1,283697047	2,06	1,348
Q92616	Stalled ribosome sensor GCN1 OS=Homo sapiens OX=9606 GN=GCN1 PE=1 SV=7	1,237623762	2,06	1,756
Q6UXN9	WD repeat-containing protein 82 OS=Homo sapiens OX=9606 GN=WDR82 PE=1 SV=1	1,485884101	2,057	1,022
O60502	Protein O-GlcNAcase OS=Homo sapiens OX=9606 GN=OGA PE=1 SV=2	0,815660685	2,056	2,395
O60749	Sorting nexin-2 OS=Homo sapiens OX=9606 GN=SNX2 PE=1 SV=2	2,074688797	2,809	1,769
P30085	UMP-CMP kinase OS=Homo sapiens OX=9606 GN=CMPK1 PE=1 SV=3	1,400560224	2,052	1,761
P27635	Large ribosomal subunit protein uL16 OS=Homo sapiens OX=9606 GN=RPL10 PE=1 SV=5	2,049180328	3,342	1,102
P07858	Cathepsin B OS=Homo sapiens OX=9606 GN=CTSB PE=1 SV=3	0,73800738	2,046	2,539
P62333	26S proteasome regulatory subunit 10B OS=Homo sapiens OX=9606 GN=PSMC6 PE=1 SV=1	1,492537313	2,039	1,327
Q15233	Non-POU domain-containing octamer-binding protein OS=Homo sapiens OX=9606 GN=NONO PE=1 SV=4	1,672240803	2,034	1,116
Q8NHP8	Putative phospholipase B-like 2 OS=Homo sapiens OX=9606 GN=PLBD2 PE=1 SV=2	0,908265213	2,031	2,436
P68036	Ubiquitin-conjugating enzyme E2 L3 OS=Homo sapiens OX=9606 GN=UBE2L3 PE=1 SV=1	1,76366843	2,03	1,148
P49588	Alanine--tRNA ligase, cytoplasmic OS=Homo sapiens OX=9606 GN=AARS1 PE=1 SV=2	0,618046972	2,03	3,094
P26639	Threonine--tRNA ligase 1, cytoplasmic OS=Homo sapiens OX=9606 GN=TARS1 PE=1 SV=3	2,040816327	3,958	1,761
P34897	Serine hydroxymethyltransferase, mitochondrial OS=Homo sapiens OX=9606 GN=SHMT2 PE=1 SV=3	1,349527665	2,025	1,758
P24941	Cyclin-dependent kinase 2 OS=Homo sapiens OX=9606 GN=CDK2 PE=1 SV=2	2,040816327	2,109	0,94
P09651	Heterogeneous nuclear ribonucleoprotein A1 OS=Homo sapiens OX=9606 GN=HNRNPA1 PE=1 SV=5	1,290322581	2,024	1,542
P48643	T-complex protein 1 subunit epsilon OS=Homo sapiens OX=9606 GN=CCT5 PE=1 SV=1	1,650165017	2,021	1,152
P17174	Aspartate aminotransferase, cytoplasmic OS=Homo sapiens OX=9606 GN=GOT1 PE=1 SV=3	2,028397566	2,616	1,509
P21281	V-type proton ATPase subunit B, brain isoform OS=Homo sapiens OX=9606 GN=ATP6V1B2 PE=1 SV=3	1,088139282	2,017	1,909
Q08378	Golgin subfamily A member 3 OS=Homo sapiens OX=9606 GN=GOLGA3 PE=1 SV=2	0,705716302	2,012	2,596
Q93008	Probable ubiquitin carboxyl-terminal hydrolase FAF-X OS=Homo sapiens OX=9606 GN=USP9X PE=1 SV=4	1,564945227	2,009	1,62
Q99541	Perilipin-2 OS=Homo sapiens OX=9606 GN=PLIN2 PE=1 SV=2	0,823045267	2,008	2,07
P60842	Eukaryotic initiation factor 4A-I OS=Homo sapiens OX=9606 GN=EIF4A1 PE=1 SV=1	2,02020202	2,812	1,438
Q12765	Secernin-1 OS=Homo sapiens OX=9606 GN=SCRN1 PE=1 SV=2	2	2,448	1,214
Q99460	26S proteasome non-ATPase regulatory subunit 1 OS=Homo sapiens OX=9606 GN=PSMD1 PE=1 SV=2	1,272264631	1,999	1,559
P09960	Leukotriene A-4 hydrolase OS=Homo sapiens OX=9606 GN=LTA4H PE=1 SV=2	1,298701299	1,998	1,593
O00487	26S proteasome non-ATPase regulatory subunit 14 OS=Homo sapiens OX=9606 GN=PSMD14 PE=1 SV=1	1,166861144	1,994	1,663
P25787	Proteasome subunit alpha type-2 OS=Homo sapiens OX=9606 GN=PSMA2 PE=1 SV=2	1,207729469	1,987	1,286
P62258	14-3-3 protein epsilon OS=Homo sapiens OX=9606 GN=YWHAE PE=1 SV=1	2,865329513	1,984	0,73
Q96CX2	BTB/POZ domain-containing protein KCTD12 OS=Homo sapiens OX=9606 GN=KCTD12 PE=1 SV=1	0,603500302	1,976	3,002
P50750	Cyclin-dependent kinase 9 OS=Homo sapiens OX=9606 GN=CDK9 PE=1 SV=3	1,62601626	1,975	1,086
P49589	Cysteine--tRNA ligase, cytoplasmic OS=Homo sapiens OX=9606 GN=CARS1 PE=1 SV=3	0,941619586	1,974	2,162
P61201	COP9 signalosome complex subunit 2 OS=Homo sapiens OX=9606 GN=COPS2 PE=1 SV=1	1,782531194	1,967	1,271
P51665	26S proteasome non-ATPase regulatory subunit 7 OS=Homo sapiens OX=9606 GN=PSMD7 PE=1 SV=2	1,379310345	1,967	1,508
P30153	Serine/threonine-protein phosphatase 2A 65 kDa regulatory subunit A alpha isoform OS=Homo sapiens OX=9606 GN=PPP2F	1,379310345	1,954	1,395
O14744	Protein arginine N-methyltransferase 5 OS=Homo sapiens OX=9606 GN=PRMT5 PE=1 SV=4	2,444987775	1,946	0,8
Q92945	Far upstream element-binding protein 2 OS=Homo sapiens OX=9606 GN=KHSRP PE=1 SV=4	1,086956522	1,944	1,607
Q02952	A-kinase anchor protein 12 OS=Homo sapiens OX=9606 GN=AKAP12 PE=1 SV=4	6,993006993	1,942	0,265
P34896	Serine hydroxymethyltransferase, cytosolic OS=Homo sapiens OX=9606 GN=SHMT1 PE=1 SV=1	2,638522427	1,941	0,714
Q7Z7H5	Transmembrane emp24 domain-containing protein 4 OS=Homo sapiens OX=9606 GN=TMED4 PE=1 SV=1	0,85106383	1,937	1,517

Q13151	Heterogeneous nuclear ribonucleoprotein A0 OS=Homo sapiens OX=9606 GN=HNRNPA0 PE=1 SV=1	2,906976744	1,935	0,818
A0A0D9SF53	RNA helicase OS=Homo sapiens OX=9606 GN=DDX3X PE=1 SV=1	2,538071066	1,935	0,882
Q07666	KH domain-containing, RNA-binding, signal transduction-associated protein 1 OS=Homo sapiens OX=9606 GN=KHDRBS1 PE=	1,257861635	1,935	1,576
P29401	Transketolase OS=Homo sapiens OX=9606 GN=TKT PE=1 SV=3	1,033057851	1,935	1,957
P78417	Glutathione S-transferase omega-1 OS=Homo sapiens OX=9606 GN=GSTO1 PE=1 SV=2	1	1,927	1,766
Q9UKK9	ADP-sugar pyrophosphatase OS=Homo sapiens OX=9606 GN=NUDT5 PE=1 SV=1	2,032520325	1,926	1,659
P16152	Carbonyl reductase [NADPH] 1 OS=Homo sapiens OX=9606 GN=CBR1 PE=1 SV=3	1,408450704	1,925	1,362
P49368	T-complex protein 1 subunit gamma OS=Homo sapiens OX=9606 GN=CCT3 PE=1 SV=4	1,410437236	1,921	1,099
Q9Y2U8	Inner nuclear membrane protein Man1 OS=Homo sapiens OX=9606 GN=LEMD3 PE=1 SV=2	0,892857143	1,919	1,913
P62829	Large ribosomal subunit protein uL14 OS=Homo sapiens OX=9606 GN=RPL23 PE=1 SV=1	2,624671916	1,915	0,712
P50990	T-complex protein 1 subunit theta OS=Homo sapiens OX=9606 GN=CCT8 PE=1 SV=4	1,712328767	1,914	1,106
Q16851	UTP--glucose-1-phosphate uridylyltransferase OS=Homo sapiens OX=9606 GN=UGP2 PE=1 SV=5	1,893939394	1,904	1,079
Q92499	ATP-dependent RNA helicase DDX1 OS=Homo sapiens OX=9606 GN=DDX1 PE=1 SV=2	2,074688797	1,899	0,916
P63279	SUMO-conjugating enzyme UBC9 OS=Homo sapiens OX=9606 GN=UBE2I PE=1 SV=1	1,798561151	1,897	0,94
Q9NU22	Midasin OS=Homo sapiens OX=9606 GN=MDN1 PE=1 SV=2	1000	1,896	0,001
P61981	14-3-3 protein gamma OS=Homo sapiens OX=9606 GN=YWHAG PE=1 SV=2	1,262626263	1,894	1,681
Q7Z3K3	Pogo transposable element with ZNF domain OS=Homo sapiens OX=9606 GN=POGZ PE=1 SV=2	1,821493625	1,893	1,105
Q96JM3	Chromosome alignment-maintaining phosphoprotein 1 OS=Homo sapiens OX=9606 GN=CHAMP1 PE=1 SV=2	2,044989775	1,892	0,906
P09496	Clathrin light chain A OS=Homo sapiens OX=9606 GN=CLTA PE=1 SV=1	0,628930818	1,89	3,049
Q8IXB1	DnaJ homolog subfamily C member 10 OS=Homo sapiens OX=9606 GN=DNAJC10 PE=1 SV=2	1,406469761	1,886	1,249
P48147	Prolyl endopeptidase OS=Homo sapiens OX=9606 GN=PREP PE=1 SV=2	2,016129032	1,885	1,138
Q07866	Kinesin light chain 1 OS=Homo sapiens OX=9606 GN=KLC1 PE=1 SV=2	1,072961373	1,884	1,893
P07900	Heat shock protein HSP 90-alpha OS=Homo sapiens OX=9606 GN=HSP90AA1 PE=1 SV=5	2,808988764	1,88	0,756
O43719	17S U2 SnRNP complex component HTATSF1 OS=Homo sapiens OX=9606 GN=HTATSF1 PE=1 SV=1	2,207505519	1,877	0,732
Q99439	Calponin-2 OS=Homo sapiens OX=9606 GN=CNN2 PE=1 SV=4	0,582750583	1,877	3,33
Q16352	Alpha-internexin OS=Homo sapiens OX=9606 GN=INA PE=1 SV=2	1000	1,873	0,001
P39060	Collagen alpha-1(XVIII) chain OS=Homo sapiens OX=9606 GN=COL18A1 PE=1 SV=5	0,20173492	1,867	7,517
P23219	Prostaglandin G/H synthase 1 OS=Homo sapiens OX=9606 GN=PTGS1 PE=1 SV=2	0,03681614	1,863	32,559
Q5JTZ9	Alanine--tRNA ligase, mitochondrial OS=Homo sapiens OX=9606 GN=AARS2 PE=1 SV=1	0,894454383	1,862	1,501
P26599	Polypyrimidine tract-binding protein 1 OS=Homo sapiens OX=9606 GN=PTBP1 PE=1 SV=2	1,177856302	1,854	1,618
Q969G3	SWI/SNF-related matrix-associated actin-dependent regulator of chromatin subfamily E member 1 OS=Homo sapiens OX=96	1,779359431	1,853	1,101
P32119	Peroxiredoxin-2 OS=Homo sapiens OX=9606 GN=PRDX2 PE=1 SV=5	2,049180328	1,852	0,925
P06493	Cyclin-dependent kinase 1 OS=Homo sapiens OX=9606 GN=CDK1 PE=1 SV=3	1,85528757	1,852	0,888
P26583	High mobility group protein B2 OS=Homo sapiens OX=9606 GN=HMGB2 PE=1 SV=2	4,608294931	1,848	0,501
Q13330	Metastasis-associated protein MTA1 OS=Homo sapiens OX=9606 GN=MTA1 PE=1 SV=2	2,949852507	1,848	0,632
P14625	Endoplasmin OS=Homo sapiens OX=9606 GN=HSP90B1 PE=1 SV=1	1,071811361	1,841	1,724
Q9Y2B0	Protein canopy homolog 2 OS=Homo sapiens OX=9606 GN=CNPY2 PE=1 SV=1	0,666666667	1,84	2,784
O00154	Cytosolic acyl coenzyme A thioester hydrolase OS=Homo sapiens OX=9606 GN=ACOT7 PE=1 SV=3	1,519756839	1,838	1,145
P49721	Proteasome subunit beta type-2 OS=Homo sapiens OX=9606 GN=PSMB2 PE=1 SV=1	0,574052813	1,835	2,924
O95777	U6 snRNA-associated Sm-like protein LSm8 OS=Homo sapiens OX=9606 GN=LSM8 PE=1 SV=3	1,706484642	1,831	1,065
P49720	Proteasome subunit beta type-3 OS=Homo sapiens OX=9606 GN=PSMB3 PE=1 SV=2	0,745156483	1,829	2,149
Q13618	Cullin-3 OS=Homo sapiens OX=9606 GN=CUL3 PE=1 SV=2	3,787878788	1,828	0,391
P50991	T-complex protein 1 subunit delta OS=Homo sapiens OX=9606 GN=CCT4 PE=1 SV=4	1,474926254	1,828	1,241
O15294	UDP-N-acetylglucosamine--peptide N-acetylglucosaminyltransferase 110 kDa subunit OS=Homo sapiens OX=9606 GN=OGT	1,76056338	1,827	0,984
O00291	Huntingtin-interacting protein 1 OS=Homo sapiens OX=9606 GN=HIP1 PE=1 SV=5	0,853970965	1,827	2,699
O75976	Carboxypeptidase D OS=Homo sapiens OX=9606 GN=CPD PE=1 SV=2	1,043841336	1,824	1,754
O60664	Perilipin-3 OS=Homo sapiens OX=9606 GN=PLIN3 PE=1 SV=3	0,449640288	1,823	4,248
O00425	Insulin-like growth factor 2 mRNA-binding protein 3 OS=Homo sapiens OX=9606 GN=IGF2BP3 PE=1 SV=2	3,322259136	1,822	0,46
Q9Y281	Cofilin-2 OS=Homo sapiens OX=9606 GN=CFL2 PE=1 SV=1	1,153402537	1,822	0,888
P55010	Eukaryotic translation initiation factor 5 OS=Homo sapiens OX=9606 GN=EIF5 PE=1 SV=2	1,745200698	1,816	1,076
P34932	Heat shock 70 kDa protein 4 OS=Homo sapiens OX=9606 GN=HSPA4 PE=1 SV=4	1,988071571	1,815	0,87
P31350	Ribonucleoside-diphosphate reductase subunit M2 OS=Homo sapiens OX=9606 GN=RRM2 PE=1 SV=1	2,207505519	1,809	1,006
Q9UKY7	Protein CDV3 homolog OS=Homo sapiens OX=9606 GN=CDV3 PE=1 SV=1	1,254705144	1,808	1,472
P06396	Gelsolin OS=Homo sapiens OX=9606 GN=GSN PE=1 SV=1	1,124859393	1,806	1,852
Q9NZL9	Methionine adenosyltransferase 2 subunit beta OS=Homo sapiens OX=9606 GN=MAT2B PE=1 SV=1	3,703703704	1,803	0,444
Q92820	Gamma-glutamyl hydrolase OS=Homo sapiens OX=9606 GN=GGH PE=1 SV=2	1,014198783	1,797	2,098
Q9Y224	RNA transcription, translation and transport factor protein OS=Homo sapiens OX=9606 GN=RTRAF PE=1 SV=1	1,579778831	1,793	1,033
Q01995	Transgelin OS=Homo sapiens OX=9606 GN=TAGLN PE=1 SV=4	0,191644308	1,79	8,959
O75027	Iron-sulfur clusters transporter ABCB7, mitochondrial OS=Homo sapiens OX=9606 GN=ABCB7 PE=1 SV=2	1,328021248	1,784	1,857
P00492	Hypoxanthine-guanine phosphoribosyltransferase OS=Homo sapiens OX=9606 GN=HPRT1 PE=1 SV=2	0,668449198	1,783	2,242
Q12824	SWI/SNF-related matrix-associated actin-dependent regulator of chromatin subfamily B member 1 OS=Homo sapiens OX=9	1,605136437	1,775	0,925
P21399	Cytoplasmic aconitate hydratase OS=Homo sapiens OX=9606 GN=ACO1 PE=1 SV=3	0,816326531	1,775	1,542
Q14202	Zinc finger MYM-type protein 3 OS=Homo sapiens OX=9606 GN=ZMYM3 PE=1 SV=2	1,468428781	1,774	1,073
Q15904	V-type proton ATPase subunit S1 OS=Homo sapiens OX=9606 GN=ATP6AP1 PE=1 SV=2	1,089324619	1,771	1,506
Q9Y3I0	RNA-splicing ligase RtcB homolog OS=Homo sapiens OX=9606 GN=RTCB PE=1 SV=1	1,76056338	1,77	0,988
P13010	X-ray repair cross-complementing protein 5 OS=Homo sapiens OX=9606 GN=XRCC5 PE=1 SV=3	1,519756839	1,769	1,185
P08708	Small ribosomal subunit protein eS17 OS=Homo sapiens OX=9606 GN=RPS17 PE=1 SV=2	2,202643172	1,767	0,565
P18085	ADP-ribosylation factor 4 OS=Homo sapiens OX=9606 GN=ARF4 PE=1 SV=3	0,695410292	1,767	3,387
Q13596	Sorting nexin-1 OS=Homo sapiens OX=9606 GN=SNX1 PE=1 SV=3	1,587301587	1,759	1,197
Q9HAU0	Pleckstrin homology domain-containing family A member 5 OS=Homo sapiens OX=9606 GN=PLEKHA5 PE=1 SV=1	0,482392668	1,757	3,345
O95881	Thioredoxin domain-containing protein 12 OS=Homo sapiens OX=9606 GN=TXNDC12 PE=1 SV=1	0,92936803	1,756	1,392
Q8NC51	SERPINE1 mRNA-binding protein 1 OS=Homo sapiens OX=9606 GN=SERBP1 PE=1 SV=2	1,785714286	1,754	0,898
P05165	Propionyl-CoA carboxylase alpha chain, mitochondrial OS=Homo sapiens OX=9606 GN=PCCA PE=1 SV=4	1,592356688	1,754	1,353
Q9UHD8	Septin-9 OS=Homo sapiens OX=9606 GN=SEPTIN9 PE=1 SV=2	1,18623962	1,754	1,338
Q92973	Transportin-1 OS=Homo sapiens OX=9606 GN=TNPO1 PE=1 SV=2	1,113585746	1,754	1,579
P55145	Mesencephalic astrocyte-derived neurotrophic factor OS=Homo sapiens OX=9606 GN=MANF PE=1 SV=3	0,849617672	1,754	2,16
P36873	Serine/threonine-protein phosphatase PP1-gamma catalytic subunit OS=Homo sapiens OX=9606 GN=PPP1CC PE=1 SV=1	1,338688086	1,745	1,265
O75822	Eukaryotic translation initiation factor 3 subunit J OS=Homo sapiens OX=9606 GN=EIF3J PE=1 SV=2	1,416430595	1,743	1,147
P54578	Ubiquitin carboxyl-terminal hydrolase 14 OS=Homo sapiens OX=9606 GN=USP14 PE=1 SV=3	1,324503311	1,742	1,078
Q9Y5L0	Transportin-3 OS=Homo sapiens OX=9606 GN=TNPO3 PE=1 SV=3	2,840909091	1,741	0,691
P31948	Stress-induced-phosphoprotein 1 OS=Homo sapiens OX=9606 GN=STIP1 PE=1 SV=1	2,257336343	1,735	0,761
Q15717	ELAV-like protein 1 OS=Homo sapiens OX=9606 GN=ELAVL1 PE=1 SV=2	1,092896175	1,731	1,653
Q6UW02	Cytochrome P450 20A1 OS=Homo sapiens OX=9606 GN=CYP20A1 PE=1 SV=1	0,687285223	1,731	3,037

Q9Y570	Protein phosphatase methylesterase 1 OS=Homo sapiens OX=9606 GN=PPME1 PE=1 SV=3	1,406469761	1,725	0,98
A0A669KB77	Microtubule-associated protein OS=Homo sapiens OX=9606 GN=MAP2 PE=1 SV=1	2,958579882	1,719	0,463
P62191	26S proteasome regulatory subunit 4 OS=Homo sapiens OX=9606 GN=PSMC1 PE=1 SV=1	1,283697047	1,719	1,377
Q8TAQ2	SWI/SNF complex subunit SMARCC2 OS=Homo sapiens OX=9606 GN=SMARCC2 PE=1 SV=1	1,321003963	1,716	1,324
P51610	Host cell factor 1 OS=Homo sapiens OX=9606 GN=HCFC1 PE=1 SV=2	1,253132832	1,716	1,284
P46108	Adapter molecule crk OS=Homo sapiens OX=9606 GN=CRK PE=1 SV=2	1,897533207	1,715	0,822
Q15084	Protein disulfide-isomerase A6 OS=Homo sapiens OX=9606 GN=PDIA6 PE=1 SV=1	1,522070015	1,714	1,095
O43570	Carbonic anhydrase 12 OS=Homo sapiens OX=9606 GN=CA12 PE=1 SV=1	1,066098081	1,714	1,948
P00558	Phosphoglycerate kinase 1 OS=Homo sapiens OX=9606 GN=PGK1 PE=1 SV=3	0,954198473	1,712	1,862
Q14839	Chromodomain-helicase-DNA-binding protein 4 OS=Homo sapiens OX=9606 GN=CHD4 PE=1 SV=2	2,624671916	1,711	0,725
Q13200	26S proteasome non-ATPase regulatory subunit 2 OS=Homo sapiens OX=9606 GN=PSMD2 PE=1 SV=3	1,552795031	1,71	1,134
Q96N67	Dedicator of cytokinesis protein 7 OS=Homo sapiens OX=9606 GN=DOCK7 PE=1 SV=4	2,227171492	1,707	1,054
Q9UBE0	SUMO-activating enzyme subunit 1 OS=Homo sapiens OX=9606 GN=SAE1 PE=1 SV=1	1,156069364	1,698	1,685
P62750	Large ribosomal subunit protein uL23 OS=Homo sapiens OX=9606 GN=RPL23A PE=1 SV=1	2,801120448	1,691	0,594
Q7L1Q6	eIF5-mimic protein 2 OS=Homo sapiens OX=9606 GN=BZW1 PE=1 SV=1	1,709401709	1,691	0,965
Q14254	Flotillin-2 OS=Homo sapiens OX=9606 GN=FLOT2 PE=1 SV=2	0,658761528	1,689	2,296
Q13098	COP9 signalosome complex subunit 1 OS=Homo sapiens OX=9606 GN=GPS1 PE=1 SV=4	1,582278481	1,687	1,084
P62701	Small ribosomal subunit protein eS4, X isoform OS=Homo sapiens OX=9606 GN=RPS4X PE=1 SV=2	2,638522427	1,685	0,685
Q9BWF3	RNA-binding protein 4 OS=Homo sapiens OX=9606 GN=RBM4 PE=1 SV=1	2,145922747	1,684	0,755
O75569	Interferon-inducible double-stranded RNA-dependent protein kinase activator A OS=Homo sapiens OX=9606 GN=PRKRA PE=	2,770083102	1,683	0,619
Q99436	Proteasome subunit beta type-7 OS=Homo sapiens OX=9606 GN=PSMB7 PE=1 SV=1	1,303780965	1,683	1,446
Q9Y6M1	Insulin-like growth factor 2 mRNA-binding protein 2 OS=Homo sapiens OX=9606 GN=IGF2BP2 PE=1 SV=2	3,759398496	1,682	0,364
Q15056	Eukaryotic translation initiation factor 4H OS=Homo sapiens OX=9606 GN=EIF4H PE=1 SV=5	2,380952381	1,677	0,861
Q02218	2-oxoglutarate dehydrogenase complex component E1 OS=Homo sapiens OX=9606 GN=OGDH PE=1 SV=3	1,092896175	1,674	1,406
Q13620	Cullin-4B OS=Homo sapiens OX=9606 GN=CUL4B PE=1 SV=4	0,414593698	1,674	3,702
P14618	Pyruvate kinase PKM OS=Homo sapiens OX=9606 GN=PKM PE=1 SV=4	0,410677618	1,672	3,97
P55072	Transitional endoplasmic reticulum ATPase OS=Homo sapiens OX=9606 GN=VCP PE=1 SV=4	1,165501166	1,671	1,292
P55036	26S proteasome non-ATPase regulatory subunit 4 OS=Homo sapiens OX=9606 GN=PSMD4 PE=1 SV=1	1,226993865	1,67	1,352
Q14134	Tripartite motif-containing protein 29 OS=Homo sapiens OX=9606 GN=TRIM29 PE=1 SV=2	0,262054507	1,67	0,522
Q92597	Protein NDRG1 OS=Homo sapiens OX=9606 GN=NDRG1 PE=1 SV=1	4,694835681	1,668	0,551
Q9HC38	Glyoxalase domain-containing protein 4 OS=Homo sapiens OX=9606 GN=GLOD4 PE=1 SV=1	3,300330033	1,667	0,515
P43246	DNA mismatch repair protein Msh2 OS=Homo sapiens OX=9606 GN=MSH2 PE=1 SV=1	3,597122302	1,666	0,615
Q8TAT6	Nuclear protein localization protein 4 homolog OS=Homo sapiens OX=9606 GN=NPLOC4 PE=1 SV=3	1,172332943	1,666	1,11
Q96A49	Synapse-associated protein 1 OS=Homo sapiens OX=9606 GN=SYAP1 PE=1 SV=1	1,017293998	1,666	1,62
Q15008	26S proteasome non-ATPase regulatory subunit 6 OS=Homo sapiens OX=9606 GN=PSMD6 PE=1 SV=1	1,259445844	1,665	1,189
O75390	Citrate synthase, mitochondrial OS=Homo sapiens OX=9606 GN=CS PE=1 SV=2	0,776397516	1,665	1,987
Q16890	Tumor protein D53 OS=Homo sapiens OX=9606 GN=TPD52L1 PE=1 SV=1	0,304692261	1,661	6,29
Q96QC0	Serine/threonine-protein phosphatase 1 regulatory subunit 10 OS=Homo sapiens OX=9606 GN=PPP1R10 PE=1 SV=1	2,024291498	1,659	0,943
Q15436	Protein transport protein Sec23A OS=Homo sapiens OX=9606 GN=SEC23A PE=1 SV=2	0,776397516	1,659	1,801
P11387	DNA topoisomerase 1 OS=Homo sapiens OX=9606 GN=TOP1 PE=1 SV=2	2,304147465	1,657	0,712
P53985	Monocarboxylate transporter 1 OS=Homo sapiens OX=9606 GN=SLC16A1 PE=1 SV=3	1,531393568	1,655	1,358
P51858	Hepatoma-derived growth factor OS=Homo sapiens OX=9606 GN=HDGF PE=1 SV=1	1,275510204	1,655	1,077
Q92598	Heat shock protein 105 kDa OS=Homo sapiens OX=9606 GN=HSPH1 PE=1 SV=1	1,838235294	1,654	0,965
Q86W42	THO complex subunit 6 homolog OS=Homo sapiens OX=9606 GN=THOC6 PE=1 SV=1	1,766784452	1,648	0,824
P17987	T-complex protein 1 subunit alpha OS=Homo sapiens OX=9606 GN=TCP1 PE=1 SV=1	1,426533524	1,645	1,125
P50454	Serpin H1 OS=Homo sapiens OX=9606 GN=SERPINH1 PE=1 SV=2	0,30703101	1,644	5,12
P29144	Tripeptidyl-peptidase 2 OS=Homo sapiens OX=9606 GN=TPP2 PE=1 SV=4	1,048218029	1,642	1,612
O00231	26S proteasome non-ATPase regulatory subunit 11 OS=Homo sapiens OX=9606 GN=PSMD11 PE=1 SV=3	1,356852103	1,635	1,328
P48449	Lanosterol synthase OS=Homo sapiens OX=9606 GN=LSS PE=1 SV=1	4,219409283	1,633	0,358
Q96M27	Protein PRRC1 OS=Homo sapiens OX=9606 GN=PRRC1 PE=1 SV=1	0,772200772	1,631	1,879
P23193	Transcription elongation factor A protein 1 OS=Homo sapiens OX=9606 GN=TCEA1 PE=1 SV=2	2,105263158	1,63	0,79
P26641	Elongation factor 1-gamma OS=Homo sapiens OX=9606 GN=EEF1G PE=1 SV=3	1,785714286	1,63	0,806
Q9Y606	Pseudouridylate synthase 1 homolog OS=Homo sapiens OX=9606 GN=PUS1 PE=1 SV=3	1,349527665	1,629	1,418
P26196	Probable ATP-dependent RNA helicase DDX6 OS=Homo sapiens OX=9606 GN=DDX6 PE=1 SV=2	2,049180328	1,628	0,711
A5YKK6	CCR4-NOT transcription complex subunit 1 OS=Homo sapiens OX=9606 GN=CNOT1 PE=1 SV=2	1,628664495	1,628	1,159
Q8TCJ2	Dolichyl-diphosphooligosaccharide--protein glycosyltransferase subunit STT3B OS=Homo sapiens OX=9606 GN=STT3B PE=1	1,383125864	1,628	1,268
P51659	Peroxisomal multifunctional enzyme type 2 OS=Homo sapiens OX=9606 GN=HSD17B4 PE=1 SV=3	0,669792364	1,627	2,167
Q9P2J5	Leucine--tRNA ligase, cytoplasmic OS=Homo sapiens OX=9606 GN=LARS1 PE=1 SV=2	1,661129568	1,625	0,972
P28066	Proteasome subunit alpha type-5 OS=Homo sapiens OX=9606 GN=PSMA5 PE=1 SV=3	0,905797101	1,617	1,623
O00571	ATP-dependent RNA helicase DDX3X OS=Homo sapiens OX=9606 GN=DDX3X PE=1 SV=3	2,087682672	1,615	0,927
Q15435	Protein phosphatase 1 regulatory subunit 7 OS=Homo sapiens OX=9606 GN=PPP1R7 PE=1 SV=1	1,773049645	1,61	0,994
O43242	26S proteasome non-ATPase regulatory subunit 3 OS=Homo sapiens OX=9606 GN=PSMD3 PE=1 SV=2	1,262626263	1,607	1,219
Q8TD43	Transient receptor potential cation channel subfamily M member 4 OS=Homo sapiens OX=9606 GN=TRPM4 PE=1 SV=1	0,453103761	1,607	3,719
Q6NYC8	Phostensin OS=Homo sapiens OX=9606 GN=PPP1R18 PE=1 SV=1	0,432713111	1,607	3,737
P26038	Moesin OS=Homo sapiens OX=9606 GN=MSN PE=1 SV=3	10,52631579	1,606	0,152
P51589	Cytochrome P450 2J2 OS=Homo sapiens OX=9606 GN=CYP2J2 PE=1 SV=2	2,487562189	1,605	0,646
Q99832	T-complex protein 1 subunit eta OS=Homo sapiens OX=9606 GN=CCT7 PE=1 SV=2	1,769911504	1,601	1,009
O43237	Cytoplasmic dynein 1 light intermediate chain 2 OS=Homo sapiens OX=9606 GN=DYNC1LI2 PE=1 SV=1	0,968054211	1,598	1,574
Q9H4A4	Aminopeptidase B OS=Homo sapiens OX=9606 GN=RNPEP PE=1 SV=2	0,825082508	1,597	1,875
P62195	26S proteasome regulatory subunit 8 OS=Homo sapiens OX=9606 GN=PSMC5 PE=1 SV=1	1,132502831	1,594	1,42
O96019	Actin-like protein 6A OS=Homo sapiens OX=9606 GN=ACTL6A PE=1 SV=1	1,464128843	1,593	1,2
O15160	DNA-directed RNA polymerases I and III subunit RPAC1 OS=Homo sapiens OX=9606 GN=POLR1C PE=1 SV=1	1,340482574	1,587	1,074
Q12929	Epidermal growth factor receptor kinase substrate 8 OS=Homo sapiens OX=9606 GN=EPS8 PE=1 SV=1	2,242152466	1,585	1,621
O75340	Programmed cell death protein 6 OS=Homo sapiens OX=9606 GN=PDCD6 PE=1 SV=1	0,789889415	1,585	2,396
P43243	Matrin-3 OS=Homo sapiens OX=9606 GN=MATR3 PE=1 SV=2	1,545595054	1,579	1,099
P47897	Glutamine--tRNA ligase OS=Homo sapiens OX=9606 GN=QARS1 PE=1 SV=1	1,358695652	1,578	1,087
P17480	Nucleolar transcription factor 1 OS=Homo sapiens OX=9606 GN=UBTF PE=1 SV=1	1,890359168	1,574	1,097
Q9Y490	Talin-1 OS=Homo sapiens OX=9606 GN=TLN1 PE=1 SV=3	0,316756414	1,57	4,806
Q99661	Kinesin-like protein KIF2C OS=Homo sapiens OX=9606 GN=KIF2C PE=1 SV=2	4,385964912	1,569	0,385
Q02543	Large ribosomal subunit protein eL20 OS=Homo sapiens OX=9606 GN=RPL18A PE=1 SV=2	2,247191011	1,567	0,646
P02533	Keratin, type I cytoskeletal 14 OS=Homo sapiens OX=9606 GN=KRT14 PE=1 SV=4	0,222766763	1,566	5,769
Q14151	Scaffold attachment factor B2 OS=Homo sapiens OX=9606 GN=SAFB2 PE=1 SV=1	1,652892562	1,563	1,066
P62081	Small ribosomal subunit protein eS7 OS=Homo sapiens OX=9606 GN=RPS7 PE=1 SV=1	1,377410468	1,563	1,23

Q00688	Peptidyl-prolyl cis-trans isomerase FKBP3 OS=Homo sapiens OX=9606 GN=FKBP3 PE=1 SV=1	2,375296912	1,562	0,692
Q8NE71	ATP-binding cassette sub-family F member 1 OS=Homo sapiens OX=9606 GN=ABCF1 PE=1 SV=2	2,93255132	1,559	0,51
P04264	Keratin, type II cytoskeletal 1 OS=Homo sapiens OX=9606 GN=KRT1 PE=1 SV=6	0,734753857	1,559	2,18
P23378	Glycine dehydrogenase (decarboxylating), mitochondrial OS=Homo sapiens OX=9606 GN=GLDC PE=1 SV=2	0,610873549	1,546	1,193
Q9UH62	Armadillo repeat-containing X-linked protein 3 OS=Homo sapiens OX=9606 GN=ARMCX3 PE=1 SV=1	1,145475372	1,545	1,322
Q9Y2Z4	Tyrosine--tRNA ligase, mitochondrial OS=Homo sapiens OX=9606 GN=YARS2 PE=1 SV=2	1,046025105	1,545	1,302
P35998	26S proteasome regulatory subunit 7 OS=Homo sapiens OX=9606 GN=PSMC2 PE=1 SV=3	1,239157373	1,542	1,187
P40227	T-complex protein 1 subunit zeta OS=Homo sapiens OX=9606 GN=CCT6A PE=1 SV=3	1,543209877	1,537	1,034
P11586	C-1-tetrahydrofolate synthase, cytoplasmic OS=Homo sapiens OX=9606 GN=MTHFD1 PE=1 SV=4	1,366120219	1,534	1,072
Q8TD30	Alanine aminotransferase 2 OS=Homo sapiens OX=9606 GN=GPT2 PE=1 SV=1	0,923361034	1,531	1,35
P00352	Aldehyde dehydrogenase 1A1 OS=Homo sapiens OX=9606 GN=ALDH1A1 PE=1 SV=2	0,082850041	1,531	9,602
Q9UH65	Switch-associated protein 70 OS=Homo sapiens OX=9606 GN=SWAP70 PE=1 SV=1	1,694915254	1,53	0,705
Q16531	DNA damage-binding protein 1 OS=Homo sapiens OX=9606 GN=DDB1 PE=1 SV=1	1,39275766	1,53	1,095
P30533	Alpha-2-macroglobulin receptor-associated protein OS=Homo sapiens OX=9606 GN=LRPAP1 PE=1 SV=1	1,206272618	1,53	1,293
Q9Y5Z4	Heme-binding protein 2 OS=Homo sapiens OX=9606 GN=HEBP2 PE=1 SV=1	1,089324619	1,529	1,361
P12956	X-ray repair cross-complementing protein 6 OS=Homo sapiens OX=9606 GN=XRCC6 PE=1 SV=2	1,512859304	1,528	0,989
P55795	Heterogeneous nuclear ribonucleoprotein H2 OS=Homo sapiens OX=9606 GN=HNRNPH2 PE=1 SV=1	1,19047619	1,528	1,225
Q9Y666	Solute carrier family 12 member 7 OS=Homo sapiens OX=9606 GN=SLC12A7 PE=1 SV=3	2,906976744	1,527	0,555
P78344	Eukaryotic translation initiation factor 4 gamma 2 OS=Homo sapiens OX=9606 GN=EIF4G2 PE=1 SV=1	1,703577513	1,526	0,973
Q9BX40	Protein LSM14 homolog B OS=Homo sapiens OX=9606 GN=LSM14B PE=1 SV=1	2,469135802	1,525	0,599
Q6KC79	Nipped-B-like protein OS=Homo sapiens OX=9606 GN=NIPBL PE=1 SV=2	1,754385965	1,524	0,862
Q16740	ATP-dependent Clp protease proteolytic subunit, mitochondrial OS=Homo sapiens OX=9606 GN=CLPP PE=1 SV=1	0,603500302	1,523	2,336
Q9UPQ0	LIM and calponin homology domains-containing protein 1 OS=Homo sapiens OX=9606 GN=LIMCH1 PE=1 SV=4	1,123595506	1,518	0,771
P52732	Kinesin-like protein KIF11 OS=Homo sapiens OX=9606 GN=KIF11 PE=1 SV=2	1,612903226	1,516	0,87
P41219	Peripherin OS=Homo sapiens OX=9606 GN=PRPH PE=1 SV=2	14,92537313	1,514	0,637
P05166	Propionyl-CoA carboxylase beta chain, mitochondrial OS=Homo sapiens OX=9606 GN=PCCB PE=1 SV=3	1,321003963	1,514	2,095
P30041	Peroxiredoxin-6 OS=Homo sapiens OX=9606 GN=PRDX6 PE=1 SV=3	0,437445319	1,514	3,746
P48556	26S proteasome non-ATPase regulatory subunit 8 OS=Homo sapiens OX=9606 GN=PSMD8 PE=1 SV=2	1,179245283	1,513	1,215
Q12792	Twinfilin-1 OS=Homo sapiens OX=9606 GN=TWF1 PE=1 SV=3	0,71787509	1,513	1,961
P04844	Dolichyl-diphosphooligosaccharide--protein glycosyltransferase subunit 2 OS=Homo sapiens OX=9606 GN=RPN2 PE=1 SV=3	0,868809731	1,511	1,775
Q9UBT2	SUMO-activating enzyme subunit 2 OS=Homo sapiens OX=9606 GN=UBA2 PE=1 SV=2	1,269035533	1,508	1,209
P83916	Chromobox protein homolog 1 OS=Homo sapiens OX=9606 GN=CBX1 PE=1 SV=1	1,926782274	1,506	0,762
Q96G03	Phosphopentomutase OS=Homo sapiens OX=9606 GN=PGM2 PE=1 SV=4	2,380952381	1,504	0,69
Q01844	RNA-binding protein EWS OS=Homo sapiens OX=9606 GN=EWSR1 PE=1 SV=1	1,724137931	1,504	0,614
O94979	Protein transport protein Sec31A OS=Homo sapiens OX=9606 GN=SEC31A PE=1 SV=3	0,633713561	1,503	2,357
Q63ZY3	KN motif and ankyrin repeat domain-containing protein 2 OS=Homo sapiens OX=9606 GN=KANK2 PE=1 SV=1	0,276319425	1,5	5,435
Q7Z5L9	Interferon regulatory factor 2-binding protein 2 OS=Homo sapiens OX=9606 GN=IRF2BP2 PE=1 SV=2	0,952380952	1,498	2,423
P04843	Dolichyl-diphosphooligosaccharide--protein glycosyltransferase subunit 1 OS=Homo sapiens OX=9606 GN=RPN1 PE=1 SV=1	0,74019245	1,498	1,716
P11216	Glycogen phosphorylase, brain form OS=Homo sapiens OX=9606 GN=PYGB PE=1 SV=5	0,939849624	1,497	1,422
P05455	Lupus La protein OS=Homo sapiens OX=9606 GN=SSB PE=1 SV=2	2,331002331	1,496	0,642
Q9NRV9	Heme-binding protein 1 OS=Homo sapiens OX=9606 GN=HEBP1 PE=1 SV=1	1,307189542	1,494	1,082
O75937	DnaJ homolog subfamily C member 8 OS=Homo sapiens OX=9606 GN=DNAJC8 PE=1 SV=2	3,389830508	1,491	0,378
P62269	Small ribosomal subunit protein uS13 OS=Homo sapiens OX=9606 GN=RPS18 PE=1 SV=3	2,583979328	1,491	0,714
Q13217	DnaJ homolog subfamily C member 3 OS=Homo sapiens OX=9606 GN=DNAJC3 PE=1 SV=1	0,899280576	1,49	1,683
P28070	Proteasome subunit beta type-4 OS=Homo sapiens OX=9606 GN=PSMB4 PE=1 SV=4	0,829875519	1,487	1,792
Q96AE4	Far upstream element-binding protein 1 OS=Homo sapiens OX=9606 GN=FUBP1 PE=1 SV=3	1,953125	1,484	0,715
Q7KZF4	Staphylococcal nuclease domain-containing protein 1 OS=Homo sapiens OX=9606 GN=SND1 PE=1 SV=1	1,798561151	1,483	0,849
Q7L2H7	Eukaryotic translation initiation factor 3 subunit M OS=Homo sapiens OX=9606 GN=EIF3M PE=1 SV=1	2,024291498	1,481	0,973
Q15021	Condensin complex subunit 1 OS=Homo sapiens OX=9606 GN=NCAPD2 PE=1 SV=3	3,610108303	1,479	0,514
Q15365	Poly(rC)-binding protein 1 OS=Homo sapiens OX=9606 GN=PCBP1 PE=1 SV=2	2,057613169	1,479	0,773
O00161	Synaptosomal-associated protein 23 OS=Homo sapiens OX=9606 GN=SNAP23 PE=1 SV=1	0,833333333	1,477	1,554
P17980	26S proteasome regulatory subunit 6A OS=Homo sapiens OX=9606 GN=PSMC3 PE=1 SV=3	1,515151515	1,474	1,066
Q13347	Eukaryotic translation initiation factor 3 subunit I OS=Homo sapiens OX=9606 GN=EIF3I PE=1 SV=1	1,672240803	1,471	0,7
P54886	Delta-1-pyrroline-5-carboxylate synthase OS=Homo sapiens OX=9606 GN=ALDH18A1 PE=1 SV=2	0,966183575	1,466	1,595
P54652	Heat shock-related 70 kDa protein 2 OS=Homo sapiens OX=9606 GN=HSPA2 PE=1 SV=1	1,589825119	1,464	0,874
P11142	Heat shock cognate 71 kDa protein OS=Homo sapiens OX=9606 GN=HSPA8 PE=1 SV=1	1,677852349	1,463	0,944
Q8NI27	THO complex subunit 2 OS=Homo sapiens OX=9606 GN=THOC2 PE=1 SV=2	1,436781609	1,463	1,089
Q7KZ85	Transcription elongation factor SPT6 OS=Homo sapiens OX=9606 GN=SUPT6H PE=1 SV=2	2,132196162	1,461	0,54
O00505	Importin subunit alpha-4 OS=Homo sapiens OX=9606 GN=KPNA3 PE=1 SV=2	1,721170396	1,46	0,746
P56192	Methionine--tRNA ligase, cytoplasmic OS=Homo sapiens OX=9606 GN=MARS1 PE=1 SV=2	1,503759398	1,46	0,953
Q9ULC3	Ras-related protein Rab-23 OS=Homo sapiens OX=9606 GN=RAB23 PE=1 SV=1	0,263365815	1,46	4,669
Q00796	Sorbitol dehydrogenase OS=Homo sapiens OX=9606 GN=SORD PE=1 SV=4	3,861003861	1,458	0,312
Q9UMS4	Pre-mRNA-processing factor 19 OS=Homo sapiens OX=9606 GN=PRPF19 PE=1 SV=1	1,288659794	1,457	0,933
Q16576	Histone-binding protein RBBP7 OS=Homo sapiens OX=9606 GN=RBBP7 PE=1 SV=1	1,319261214	1,456	0,878
P23284	Peptidyl-prolyl cis-trans isomerase B OS=Homo sapiens OX=9606 GN=PPIB PE=1 SV=2	0,82781457	1,453	1,569
P46777	Large ribosomal subunit protein uL18 OS=Homo sapiens OX=9606 GN=RPL5 PE=1 SV=3	1,474926254	1,451	0,991
P09493	Tropomyosin alpha-1 chain OS=Homo sapiens OX=9606 GN=TPM1 PE=1 SV=2	0,272925764	1,45	4,302
P11940	Polyadenylate-binding protein 1 OS=Homo sapiens OX=9606 GN=PABPC1 PE=1 SV=2	1,38121547	1,447	1,037
P62495	Eukaryotic peptide chain release factor subunit 1 OS=Homo sapiens OX=9606 GN=ETF1 PE=1 SV=3	1,278772379	1,447	0,985
Q6IN85	Serine/threonine-protein phosphatase 4 regulatory subunit 3A OS=Homo sapiens OX=9606 GN=PPP4R3A PE=1 SV=1	1,490312966	1,446	0,989
Q15392	Delta(24)-sterol reductase OS=Homo sapiens OX=9606 GN=DHCR24 PE=1 SV=2	1,730103806	1,445	0,809
P16671	Platelet glycoprotein 4 OS=Homo sapiens OX=9606 GN=CD36 PE=1 SV=2	0,018703475	1,445	188,864
Q9UNZ2	NSFL1 cofactor p47 OS=Homo sapiens OX=9606 GN=NSFL1C PE=1 SV=2	1,776198934	1,444	0,919
P62241	Small ribosomal subunit protein eS8 OS=Homo sapiens OX=9606 GN=RPS8 PE=1 SV=2	1,926782274	1,443	0,823
Q12888	TP53-binding protein 1 OS=Homo sapiens OX=9606 GN=TP53BP1 PE=1 SV=2	0,913242009	1,442	1,71
Q04446	1,4-alpha-glucan-branching enzyme OS=Homo sapiens OX=9606 GN=GBE1 PE=1 SV=3	0,489715965	1,439	2,324
Q15046	Lysine--tRNA ligase OS=Homo sapiens OX=9606 GN=KARS1 PE=1 SV=3	1,162790698	1,433	1,179
P25788	Proteasome subunit alpha type-3 OS=Homo sapiens OX=9606 GN=PSMA3 PE=1 SV=2	0,846740051	1,433	1,84
Q14152	Eukaryotic translation initiation factor 3 subunit A OS=Homo sapiens OX=9606 GN=EIF3A PE=1 SV=1	2,481389578	1,428	0,586
O60488	Long-chain-fatty-acid--CoA ligase 4 OS=Homo sapiens OX=9606 GN=ACSL4 PE=1 SV=2	0,404040404	1,428	3,315
P56545	C-terminal-binding protein 2 OS=Homo sapiens OX=9606 GN=CTBP2 PE=1 SV=1	1,831501832	1,426	0,739
P12532	Creatine kinase U-type, mitochondrial OS=Homo sapiens OX=9606 GN=CKMT1A PE=1 SV=1	0,638162093	1,425	1,983
Q99733	Nucleosome assembly protein 1-like 4 OS=Homo sapiens OX=9606 GN=NAP1L4 PE=1 SV=1	2,941176471	1,424	0,956

Q86TX2	Acyl-coenzyme A thioesterase 1 OS=Homo sapiens OX=9606 GN=ACOT1 PE=1 SV=1	0,411692054	1,424	3,558
Q9HCE1	Helicase MOV-10 OS=Homo sapiens OX=9606 GN=MOV10 PE=1 SV=2	1,223990208	1,423	1,091
P35908	Keratin, type II cytoskeletal 2 epidermal OS=Homo sapiens OX=9606 GN=KRT2 PE=1 SV=2	0,468164794	1,422	2,948
P07814	Bifunctional glutamate/proline--tRNA ligase OS=Homo sapiens OX=9606 GN=EPRS1 PE=1 SV=5	1,360544218	1,421	1,022
Q9Y639	Neuroplastin OS=Homo sapiens OX=9606 GN=NPTN PE=1 SV=2	0,206185567	1,421	6,857
Q15427	Splicing factor 3B subunit 4 OS=Homo sapiens OX=9606 GN=SF3B4 PE=1 SV=1	1,736111111	1,419	0,884
P40937	Replication factor C subunit 5 OS=Homo sapiens OX=9606 GN=RFC5 PE=1 SV=1	1,650165017	1,419	0,862
P48735	Isocitrate dehydrogenase [NADP], mitochondrial OS=Homo sapiens OX=9606 GN=IDH2 PE=1 SV=2	0,896860987	1,419	1,58
P13674	Prolyl 4-hydroxylase subunit alpha-1 OS=Homo sapiens OX=9606 GN=P4HA1 PE=1 SV=2	0,407830343	1,419	3,332
P62714	Serine/threonine-protein phosphatase 2A catalytic subunit beta isoform OS=Homo sapiens OX=9606 GN=PPP2CB PE=1 SV=1	1,754385965	1,418	0,76
Q8IZ81	ELMO domain-containing protein 2 OS=Homo sapiens OX=9606 GN=ELMOD2 PE=1 SV=1	1,663893511	1,416	0,992
O00232	26S proteasome non-ATPase regulatory subunit 12 OS=Homo sapiens OX=9606 GN=PSMD12 PE=1 SV=3	1,176470588	1,416	1,397
Q9BY44	Eukaryotic translation initiation factor 2A OS=Homo sapiens OX=9606 GN=EIF2A PE=1 SV=3	2,197802198	1,413	0,499
O43390	Heterogeneous nuclear ribonucleoprotein R OS=Homo sapiens OX=9606 GN=HNRNPR PE=1 SV=1	1,086956522	1,412	1,234
Q00839	Heterogeneous nuclear ribonucleoprotein U OS=Homo sapiens OX=9606 GN=HNRNPU PE=1 SV=6	1,470588235	1,409	0,935
O43752	Syntaxin-6 OS=Homo sapiens OX=9606 GN=STX6 PE=1 SV=1	0,969932105	1,407	0,896
P30622	CAP-Gly domain-containing linker protein 1 OS=Homo sapiens OX=9606 GN=CLIP1 PE=1 SV=2	0,689179876	1,405	2,948
P25786	Proteasome subunit alpha type-1 OS=Homo sapiens OX=9606 GN=PSMA1 PE=1 SV=1	0,763358779	1,404	1,82
Q13423	NAD(P) transhydrogenase, mitochondrial OS=Homo sapiens OX=9606 GN=NNT PE=1 SV=3	0,468603561	1,403	3,082
P13861	cAMP-dependent protein kinase type II-alpha regulatory subunit OS=Homo sapiens OX=9606 GN=PRKAR2A PE=1 SV=2	1,432664756	1,401	0,808
O60306	RNA helicase aquarius OS=Homo sapiens OX=9606 GN=AQR PE=1 SV=4	1,119820829	1,399	1,395
P67775	Serine/threonine-protein phosphatase 2A catalytic subunit alpha isoform OS=Homo sapiens OX=9606 GN=PPP2CA PE=1 SV=	1,805054152	1,397	0,726
P53618	Coatomer subunit beta OS=Homo sapiens OX=9606 GN=COPB1 PE=1 SV=3	0,817661488	1,396	1,662
O15498	Synaptobrevin homolog YKT6 OS=Homo sapiens OX=9606 GN=YKT6 PE=1 SV=1	1,652892562	1,395	0,34
Q96DH6	RNA-binding protein Musashi homolog 2 OS=Homo sapiens OX=9606 GN=MSI2 PE=1 SV=1	3,03030303	1,394	0,514
P23246	Splicing factor, proline- and glutamine-rich OS=Homo sapiens OX=9606 GN=SFPQ PE=1 SV=2	1,228501229	1,394	0,958
Q13148	TAR DNA-binding protein 43 OS=Homo sapiens OX=9606 GN=TARDBP PE=1 SV=1	1,526717557	1,393	0,967
Q9UNM6	26S proteasome non-ATPase regulatory subunit 13 OS=Homo sapiens OX=9606 GN=PSMD13 PE=1 SV=2	1,13507378	1,392	1,244
A0A0A0MQS9	Laminin subunit alpha 4 OS=Homo sapiens OX=9606 GN=LAMA4 PE=1 SV=1	0,64061499	1,391	1,308
Q9NPQ8	Synembryn-A OS=Homo sapiens OX=9606 GN=RIC8A PE=1 SV=3	1,373626374	1,39	1,176
P05388	Large ribosomal subunit protein uL10 OS=Homo sapiens OX=9606 GN=RPLP0 PE=1 SV=1	1,307189542	1,389	1,035
Q96JJ7	Protein disulfide-isomerase TMX3 OS=Homo sapiens OX=9606 GN=TMX3 PE=1 SV=2	0,28019053	1,389	5,354
Q14684	Ribosomal RNA processing protein 1 homolog B OS=Homo sapiens OX=9606 GN=RRP1B PE=1 SV=3	5,076142132	1,387	0,352
P55060	Exportin-2 OS=Homo sapiens OX=9606 GN=CSE1L PE=1 SV=3	2,309468822	1,386	0,654
Q9BT78	COP9 signalosome complex subunit 4 OS=Homo sapiens OX=9606 GN=COPS4 PE=1 SV=1	1,371742112	1,386	1,022
P46977	Dolichyl-diphosphooligosaccharide--protein glycosyltransferase subunit STT3A OS=Homo sapiens OX=9606 GN=STT3A PE=1	0,886524823	1,382	1,714
Q99986	Serine/threonine-protein kinase VRK1 OS=Homo sapiens OX=9606 GN=VRK1 PE=1 SV=1	2,392344498	1,381	0,794
P48637	Glutathione synthetase OS=Homo sapiens OX=9606 GN=GSS PE=1 SV=1	0,989119683	1,38	1,434
Q8NC56	LEM domain-containing protein 2 OS=Homo sapiens OX=9606 GN=LEMD2 PE=1 SV=1	0,762776506	1,38	1,776
Q9Y2W2	WW domain-binding protein 11 OS=Homo sapiens OX=9606 GN=WBP11 PE=1 SV=1	2,087682672	1,379	0,687
O94903	Pyridoxal phosphate homeostasis protein OS=Homo sapiens OX=9606 GN=PLPBP PE=1 SV=1	2,008032129	1,376	0,74
P82979	SAP domain-containing ribonucleoprotein OS=Homo sapiens OX=9606 GN=SARNP PE=1 SV=3	1,335113485	1,375	1,033
P35241	Radixin OS=Homo sapiens OX=9606 GN=RDX PE=1 SV=1	0,825763832	1,374	1,502
Q9UIG0	Tyrosine-protein kinase BAZ1B OS=Homo sapiens OX=9606 GN=BAZ1B PE=1 SV=2	1,872659176	1,37	0,693
P35606	Coatomer subunit beta' OS=Homo sapiens OX=9606 GN=COPB2 PE=1 SV=2	0,997008973	1,368	1,39
P53621	Coatomer subunit alpha OS=Homo sapiens OX=9606 GN=COPA PE=1 SV=2	1,051524711	1,363	1,216
P61353	Large ribosomal subunit protein eL27 OS=Homo sapiens OX=9606 GN=RPL27 PE=1 SV=2	1,023541453	1,362	1,25
A0A1B0GVI3	Keratin, type I cytoskeletal 10 OS=Homo sapiens OX=9606 GN=KRT10 PE=1 SV=2	0,577034045	1,361	2,26
A0A0J9YXF2	Paraoxonase OS=Homo sapiens OX=9606 GN=PON2 PE=1 SV=1	0,473709143	1,361	2,955
Q04917	14-3-3 protein eta OS=Homo sapiens OX=9606 GN=YWHAH PE=1 SV=4	1,251564456	1,36	1,248
P78527	DNA-dependent protein kinase catalytic subunit OS=Homo sapiens OX=9606 GN=PRKDC PE=1 SV=3	1,044932079	1,357	1,303
P51648	Aldehyde dehydrogenase family 3 member A2 OS=Homo sapiens OX=9606 GN=ALDH3A2 PE=1 SV=1	0,941619586	1,356	1,631
Q9NQW7	Xaa-Pro aminopeptidase 1 OS=Homo sapiens OX=9606 GN=XPNPEP1 PE=1 SV=3	0,734753857	1,356	1,81
P20700	Lamin-B1 OS=Homo sapiens OX=9606 GN=LMNB1 PE=1 SV=2	1,650165017	1,355	0,807
Q15293	Reticulocalbin-1 OS=Homo sapiens OX=9606 GN=RCN1 PE=1 SV=1	0,74682599	1,353	1,873
Q9P0K7	Ankycorbin OS=Homo sapiens OX=9606 GN=RAI14 PE=1 SV=2	0,444247001	1,35	3,091
Q08945	FACT complex subunit SSRP1 OS=Homo sapiens OX=9606 GN=SSRP1 PE=1 SV=1	2,976190476	1,349	0,433
O94973	AP-2 complex subunit alpha-2 OS=Homo sapiens OX=9606 GN=AP2A2 PE=1 SV=2	0,623830318	1,349	2,095
P08240	Signal recognition particle receptor subunit alpha OS=Homo sapiens OX=9606 GN=SRPRA PE=1 SV=2	1,828153565	1,343	1,061
Q13131	5'-AMP-activated protein kinase catalytic subunit alpha-1 OS=Homo sapiens OX=9606 GN=PRKAA1 PE=1 SV=4	0,777604977	1,342	0,949
Q96TC7	Regulator of microtubule dynamics protein 3 OS=Homo sapiens OX=9606 GN=RMDN3 PE=1 SV=2	0,698324022	1,342	2,238
Q99536	Synaptic vesicle membrane protein VAT-1 homolog OS=Homo sapiens OX=9606 GN=VAT1 PE=1 SV=2	0,650618087	1,34	2,196
P39656	Dolichyl-diphosphooligosaccharide--protein glycosyltransferase 48 kDa subunit OS=Homo sapiens OX=9606 GN=DDOST PE=	0,781860829	1,337	1,697
P62979	Ubiquitin-ribosomal protein eS31 fusion protein OS=Homo sapiens OX=9606 GN=RPS27A PE=1 SV=2	1,006036217	1,333	1,21
O00303	Eukaryotic translation initiation factor 3 subunit F OS=Homo sapiens OX=9606 GN=EIF3F PE=1 SV=1	1,675041876	1,332	0,918
P39023	Large ribosomal subunit protein uL3 OS=Homo sapiens OX=9606 GN=RPL3 PE=1 SV=2	1,577287066	1,33	0,763
P31943	Heterogeneous nuclear ribonucleoprotein H OS=Homo sapiens OX=9606 GN=HNRNPH1 PE=1 SV=4	1,422475107	1,33	0,976
Q9NR28	Diablo IAP-binding mitochondrial protein OS=Homo sapiens OX=9606 GN=DIABLO PE=1 SV=1	1,805054152	1,327	0,826
P12004	Proliferating cell nuclear antigen OS=Homo sapiens OX=9606 GN=PCNA PE=1 SV=1	1,308900524	1,327	0,764
Q9UGP8	Translocation protein SEC63 homolog OS=Homo sapiens OX=9606 GN=SEC63 PE=1 SV=2	1,655629139	1,323	0,758
Q8IY81	pre-rRNA 2'-O-ribose RNA methyltransferase FTSJ3 OS=Homo sapiens OX=9606 GN=FTSJ3 PE=1 SV=2	1,968503937	1,322	0,642
Q92688	Acidic leucine-rich nuclear phosphoprotein 32 family member B OS=Homo sapiens OX=9606 GN=ANP32B PE=1 SV=1	1,488095238	1,321	0,905
Q9H488	GDP-fucose protein O-fucosyltransferase 1 OS=Homo sapiens OX=9606 GN=POFUT1 PE=1 SV=1	0,597014925	1,321	2,103
Q9H0D6	5'-3' exoribonuclease 2 OS=Homo sapiens OX=9606 GN=XRN2 PE=1 SV=1	1,841620626	1,32	0,857
O75367	Core histone macro-H2A.1 OS=Homo sapiens OX=9606 GN=MACROH2A1 PE=1 SV=5	1,531393568	1,32	0,978
E7EVA0	Microtubule-associated protein OS=Homo sapiens OX=9606 GN=MAP4 PE=1 SV=1	0,634115409	1,319	2,391
P20073	Annexin A7 OS=Homo sapiens OX=9606 GN=ANXA7 PE=1 SV=3	0,514403292	1,319	2,552
P33991	DNA replication licensing factor MCM4 OS=Homo sapiens OX=9606 GN=MCM4 PE=1 SV=5	2,083333333	1,318	0,612
O14980	Exportin-1 OS=Homo sapiens OX=9606 GN=XPO1 PE=1 SV=1	1,538461538	1,318	0,908
O75436	Vacuolar protein sorting-associated protein 26A OS=Homo sapiens OX=9606 GN=VPS26A PE=1 SV=2	1,008064516	1,317	1,21
O14818	Proteasome subunit alpha type-7 OS=Homo sapiens OX=9606 GN=PSMA7 PE=1 SV=1	0,81366965	1,316	1,549
Q9Y263	Phospholipase A-2-activating protein OS=Homo sapiens OX=9606 GN=PLAA PE=1 SV=2	0,647668394	1,314	2,067
Q8NBS9	Thioredoxin domain-containing protein 5 OS=Homo sapiens OX=9606 GN=TXNDC5 PE=1 SV=2	0,615384615	1,313	1,669

Q5JRA6	Transport and Golgi organization protein 1 homolog OS=Homo sapiens OX=9606 GN=MIA3 PE=1 SV=1	0,543478261	1,313	2,226
Q15942	Zyxin OS=Homo sapiens OX=9606 GN=ZYX PE=1 SV=1	0,431592577	1,312	3,611
P62906	Large ribosomal subunit protein uL1 OS=Homo sapiens OX=9606 GN=RPL10A PE=1 SV=2	1,051524711	1,311	0,99
P11310	Medium-chain specific acyl-CoA dehydrogenase, mitochondrial OS=Homo sapiens OX=9606 GN=ACADM PE=1 SV=1	1,808318264	1,31	0,71
Q9UHX1	Poly(U)-binding-splicing factor PUF60 OS=Homo sapiens OX=9606 GN=PUF60 PE=1 SV=1	1,788908766	1,31	0,763
P23381	Tryptophan--tRNA ligase, cytoplasmic OS=Homo sapiens OX=9606 GN=WARS1 PE=1 SV=2	0,417536534	1,309	2,3
P06753	Tropomyosin alpha-3 chain OS=Homo sapiens OX=9606 GN=TPM3 PE=1 SV=2	0,416493128	1,306	3,394
P17844	Probable ATP-dependent RNA helicase DDX5 OS=Homo sapiens OX=9606 GN=DDX5 PE=1 SV=1	2	1,305	0,674
Q12972	Nuclear inhibitor of protein phosphatase 1 OS=Homo sapiens OX=9606 GN=PPP1R8 PE=1 SV=2	1,519756839	1,305	0,755
Q9Y285	Phenylalanine--tRNA ligase alpha subunit OS=Homo sapiens OX=9606 GN=FARSA PE=1 SV=3	1,479289941	1,305	0,804
Q14697	Neutral alpha-glucosidase AB OS=Homo sapiens OX=9606 GN=GANAB PE=1 SV=3	0,676589986	1,304	1,755
P60900	Proteasome subunit alpha type-6 OS=Homo sapiens OX=9606 GN=PSMA6 PE=1 SV=1	0,954198473	1,303	1,232
O60610	Protein diaphanous homolog 1 OS=Homo sapiens OX=9606 GN=DIAPH1 PE=1 SV=2	1,663893511	1,301	0,835
P84090	Enhancer of rudimentary homolog OS=Homo sapiens OX=9606 GN=ERH PE=1 SV=1	1,248439451	1,301	1,066
P49189	4-trimethylaminobutyraldehyde dehydrogenase OS=Homo sapiens OX=9606 GN=ALDH9A1 PE=1 SV=3	0,806451613	1,299	1,801
Q07960	Rho GTPase-activating protein 1 OS=Homo sapiens OX=9606 GN=ARHGAP1 PE=1 SV=1	0,702740689	1,296	1,8
Q9BSJ8	Extended synaptotagmin-1 OS=Homo sapiens OX=9606 GN=ESYT1 PE=1 SV=1	0,368324125	1,296	3,176
P37840	Alpha-synuclein OS=Homo sapiens OX=9606 GN=SNCA PE=1 SV=1	0,162495938	1,296	5,081
Q9NRL2	Bromodomain adjacent to zinc finger domain protein 1A OS=Homo sapiens OX=9606 GN=BAZ1A PE=1 SV=2	1,841620626	1,295	0,745
O43264	Centromere/kinetochore protein zw10 homolog OS=Homo sapiens OX=9606 GN=ZW10 PE=1 SV=3	1,291989664	1,29	0,713
O43396	Thioredoxin-like protein 1 OS=Homo sapiens OX=9606 GN=TXNL1 PE=1 SV=3	1,265822785	1,29	1,07
O95747	Serine/threonine-protein kinase OSR1 OS=Homo sapiens OX=9606 GN=OXSR1 PE=1 SV=1	1,164144354	1,289	1,173
Q9NX46	ADP-ribosylhydrolase ARH3 OS=Homo sapiens OX=9606 GN=ADPRS PE=1 SV=1	1,351351351	1,288	0,904
Q13263	Transcription intermediary factor 1-beta OS=Homo sapiens OX=9606 GN=TRIM28 PE=1 SV=5	1,124859393	1,287	1,115
O76070	Gamma-synuclein OS=Homo sapiens OX=9606 GN=SNCG PE=1 SV=2	0,059234688	1,286	14,244
P07099	Epoxide hydrolase 1 OS=Homo sapiens OX=9606 GN=EPHX1 PE=1 SV=1	0,06852131	1,285	20,891
P22033	Methylmalonyl-CoA mutase, mitochondrial OS=Homo sapiens OX=9606 GN=MMUT PE=1 SV=4	0,768049155	1,284	1,789
Q13283	Ras GTPase-activating protein-binding protein 1 OS=Homo sapiens OX=9606 GN=G3BP1 PE=1 SV=1	1,988071571	1,283	0,596
P15559	NAD(P)H dehydrogenase [quinone] 1 OS=Homo sapiens OX=9606 GN=NQO1 PE=1 SV=1	0,419287212	1,283	3,233
P62917	Large ribosomal subunit protein uL2 OS=Homo sapiens OX=9606 GN=RPL8 PE=1 SV=2	1,712328767	1,28	0,782
Q9Y4F1	FERM, ARHGEF and pleckstrin domain-containing protein 1 OS=Homo sapiens OX=9606 GN=FARP1 PE=1 SV=1	2,197802198	1,278	0,985
P62280	Small ribosomal subunit protein uS17 OS=Homo sapiens OX=9606 GN=RPS11 PE=1 SV=3	1,798561151	1,277	0,808
P30038	Delta-1-pyrroline-5-carboxylate dehydrogenase, mitochondrial OS=Homo sapiens OX=9606 GN=ALDH4A1 PE=1 SV=3	1,730103806	1,276	0,84
P62249	Small ribosomal subunit protein uS9 OS=Homo sapiens OX=9606 GN=RPS16 PE=1 SV=2	1,949317739	1,273	0,592
P62851	Small ribosomal subunit protein eS25 OS=Homo sapiens OX=9606 GN=RPS25 PE=1 SV=1	2,617801047	1,272	0,447
P29692	Elongation factor 1-delta OS=Homo sapiens OX=9606 GN=EEF1D PE=1 SV=5	1,414427157	1,271	1,218
Q14157	Ubiquitin-associated protein 2-like OS=Homo sapiens OX=9606 GN=UBAP2L PE=1 SV=2	1,184834123	1,27	1,053
P20618	Proteasome subunit beta type-1 OS=Homo sapiens OX=9606 GN=PSMB1 PE=1 SV=2	0,778210117	1,269	1,695
P23919	Thymidylate kinase OS=Homo sapiens OX=9606 GN=DTYMK PE=1 SV=4	1,689189189	1,265	0,637
P78406	mRNA export factor RAE1 OS=Homo sapiens OX=9606 GN=RAE1 PE=1 SV=1	1,893939394	1,263	0,567
P39019	Small ribosomal subunit protein eS19 OS=Homo sapiens OX=9606 GN=RPS19 PE=1 SV=2	1,240694789	1,263	0,968
P11177	Pyruvate dehydrogenase E1 component subunit beta, mitochondrial OS=Homo sapiens OX=9606 GN=PDHB PE=1 SV=3	1,075268817	1,262	1,199
Q15020	Squamous cell carcinoma antigen recognized by T-cells 3 OS=Homo sapiens OX=9606 GN=SART3 PE=1 SV=1	2,159827214	1,258	0,615
Q16401	26S proteasome non-ATPase regulatory subunit 5 OS=Homo sapiens OX=9606 GN=PSMD5 PE=1 SV=3	0,683994528	1,258	1,755
P21397	Amine oxidase [flavin-containing] A OS=Homo sapiens OX=9606 GN=MAOA PE=1 SV=1	0,677506775	1,256	2,184
Q14019	Coactosin-like protein OS=Homo sapiens OX=9606 GN=COTL1 PE=1 SV=3	0,778816199	1,255	1,477
Q9UBV2	Protein sel-1 homolog 1 OS=Homo sapiens OX=9606 GN=SEL1L PE=1 SV=3	0,574712644	1,254	2,289
Q99459	Cell division cycle 5-like protein OS=Homo sapiens OX=9606 GN=CDC5L PE=1 SV=2	1,694915254	1,253	0,776
Q53H12	Acylglycerol kinase, mitochondrial OS=Homo sapiens OX=9606 GN=AGK PE=1 SV=2	1,114827202	1,253	1,14
P12081	Histidine--tRNA ligase, cytoplasmic OS=Homo sapiens OX=9606 GN=HARS1 PE=1 SV=2	1,062699256	1,253	1,117
P62314	Small nuclear ribonucleoprotein Sm D1 OS=Homo sapiens OX=9606 GN=SNRPD1 PE=1 SV=1	1,353179973	1,252	1,055
Q9Y678	Coatomer subunit gamma-1 OS=Homo sapiens OX=9606 GN=COPG1 PE=1 SV=1	0,589970501	1,251	1,831
P15880	Small ribosomal subunit protein uS5 OS=Homo sapiens OX=9606 GN=RPS2 PE=1 SV=2	1,422475107	1,25	0,86
P27694	Replication protein A 70 kDa DNA-binding subunit OS=Homo sapiens OX=9606 GN=RPA1 PE=1 SV=2	2,518891688	1,248	0,484
O00541	Pescadillo homolog OS=Homo sapiens OX=9606 GN=PES1 PE=1 SV=1	2,666666667	1,245	0,393
P46783	Small ribosomal subunit protein eS10 OS=Homo sapiens OX=9606 GN=RPS10 PE=1 SV=1	1,394700139	1,245	0,692
Q9UIA9	Exportin-7 OS=Homo sapiens OX=9606 GN=XPO7 PE=1 SV=3	1,373626374	1,242	0,932
P13645	Keratin, type I cytoskeletal 10 OS=Homo sapiens OX=9606 GN=KRT10 PE=1 SV=6	0,494071146	1,242	2,444
Q9P035	Very-long-chain (3R)-3-hydroxyacyl-CoA dehydratase 3 OS=Homo sapiens OX=9606 GN=HACD3 PE=1 SV=2	1,524390244	1,24	0,734
P12931	Proto-oncogene tyrosine-protein kinase Src OS=Homo sapiens OX=9606 GN=SRC PE=1 SV=3	1,776198934	1,239	0,566
Q15424	Scaffold attachment factor B1 OS=Homo sapiens OX=9606 GN=SAFB PE=1 SV=4	1,709401709	1,239	0,811
Q9BZZ5	Apoptosis inhibitor 5 OS=Homo sapiens OX=9606 GN=API5 PE=1 SV=3	1,43472023	1,238	0,846
P60660	Myosin light polypeptide 6 OS=Homo sapiens OX=9606 GN=MYL6 PE=1 SV=2	0,281848929	1,238	3,269
P46781	Small ribosomal subunit protein uS4 OS=Homo sapiens OX=9606 GN=RPS9 PE=1 SV=3	2,212389381	1,236	0,589
P28482	Mitogen-activated protein kinase 1 OS=Homo sapiens OX=9606 GN=MAPK1 PE=1 SV=3	1,328021248	1,236	0,945
P53992	Protein transport protein Sec24C OS=Homo sapiens OX=9606 GN=SEC24C PE=1 SV=3	0,938967136	1,236	1,396
P61221	ATP-binding cassette sub-family E member 1 OS=Homo sapiens OX=9606 GN=ABCE1 PE=1 SV=1	1,644736842	1,234	0,826
P43490	Nicotinamide phosphoribosyltransferase OS=Homo sapiens OX=9606 GN=NAMPT PE=1 SV=1	0,593824228	1,234	1,947
P23396	Small ribosomal subunit protein uS3 OS=Homo sapiens OX=9606 GN=RPS3 PE=1 SV=2	1,307189542	1,23	0,881
Q13438	Protein OS-9 OS=Homo sapiens OX=9606 GN=OS9 PE=1 SV=1	0,459770115	1,23	2,912
Q9NSD9	Phenylalanine--tRNA ligase beta subunit OS=Homo sapiens OX=9606 GN=FARSB PE=1 SV=3	1,512859304	1,229	0,828
Q05519	Serine/arginine-rich splicing factor 11 OS=Homo sapiens OX=9606 GN=SRSF11 PE=1 SV=1	2,150537634	1,228	0,528
P48444	Coatomer subunit delta OS=Homo sapiens OX=9606 GN=ARCN1 PE=1 SV=1	1,222493888	1,228	0,953
Q99715	Collagen alpha-1(XII) chain OS=Homo sapiens OX=9606 GN=COL12A1 PE=1 SV=2	0,078363765	1,228	13,887
Q53GS9	Ubiquitin carboxyl-terminal hydrolase 39 OS=Homo sapiens OX=9606 GN=USP39 PE=1 SV=2	2,222222222	1,227	0,594
Q2TAY7	WD40 repeat-containing protein SMU1 OS=Homo sapiens OX=9606 GN=SMU1 PE=1 SV=2	1,404494382	1,226	0,813
Q13838	Spliceosome RNA helicase DDX39B OS=Homo sapiens OX=9606 GN=DDX39B PE=1 SV=1	1,324503311	1,226	0,835
Q86YP4	Transcriptional repressor p66-alpha OS=Homo sapiens OX=9606 GN=GATAD2A PE=1 SV=1	1,680672269	1,224	0,671
P27816	Microtubule-associated protein 4 OS=Homo sapiens OX=9606 GN=MAP4 PE=1 SV=3	0,546746856	1,22	2,421
P24534	Elongation factor 1-beta OS=Homo sapiens OX=9606 GN=EEF1B2 PE=1 SV=3	1,692047377	1,218	0,674
P62277	Small ribosomal subunit protein uS15 OS=Homo sapiens OX=9606 GN=RPS13 PE=1 SV=2	1,547987616	1,218	0,932
O43447	Peptidyl-prolyl cis-trans isomerase H OS=Homo sapiens OX=9606 GN=PPIH PE=1 SV=1	1,418439716	1,218	0,597
P37837	Transaldolase OS=Homo sapiens OX=9606 GN=TALDO1 PE=1 SV=2	0,683060109	1,217	2,132

P63208	S-phase kinase-associated protein 1 OS=Homo sapiens OX=9606 GN=SKP1 PE=1 SV=2	1,524390244	1,216	0,788
P78347	General transcription factor II-I OS=Homo sapiens OX=9606 GN=GTF2I PE=1 SV=2	1,85528757	1,209	0,547
P14678	Small nuclear ribonucleoprotein-associated proteins B and B' OS=Homo sapiens OX=9606 GN=SNRPB PE=1 SV=2	1,068376068	1,208	1,264
O75821	Eukaryotic translation initiation factor 3 subunit G OS=Homo sapiens OX=9606 GN=EIF3G PE=1 SV=2	1,811594203	1,206	0,594
Q9BUQ8	Probable ATP-dependent RNA helicase DDX23 OS=Homo sapiens OX=9606 GN=DDX23 PE=1 SV=3	1,385041551	1,206	0,877
P21291	Cysteine and glycine-rich protein 1 OS=Homo sapiens OX=9606 GN=CSRP1 PE=1 SV=3	0,391083301	1,206	2,811
Q99623	Prohibitin-2 OS=Homo sapiens OX=9606 GN=PHB2 PE=1 SV=2	1,070663812	1,204	0,984
Q9GZL7	Ribosome biogenesis protein WDR12 OS=Homo sapiens OX=9606 GN=WDR12 PE=1 SV=2	2,066115702	1,203	0,591
P37059	17-beta-hydroxysteroid dehydrogenase type 2 OS=Homo sapiens OX=9606 GN=HSD17B2 PE=1 SV=1	0,967117988	1,202	4,71
O15126	Secretory carrier-associated membrane protein 1 OS=Homo sapiens OX=9606 GN=SCAMP1 PE=1 SV=2	0,694927033	1,2	1,458
Q99613	Eukaryotic translation initiation factor 3 subunit C OS=Homo sapiens OX=9606 GN=EIF3C PE=1 SV=1	2,008032129	1,198	0,618
Q9H5V8	CUB domain-containing protein 1 OS=Homo sapiens OX=9606 GN=CDCP1 PE=1 SV=3	0,732064422	1,198	2,304
Q9HDC9	Adipocyte plasma membrane-associated protein OS=Homo sapiens OX=9606 GN=APMAP PE=1 SV=2	0,577700751	1,196	1,996
P18206	Vinculin OS=Homo sapiens OX=9606 GN=VCL PE=1 SV=4	0,292568754	1,196	3,911
P33993	DNA replication licensing factor MCM7 OS=Homo sapiens OX=9606 GN=MCM7 PE=1 SV=4	1,876172608	1,195	0,592
P26885	Peptidyl-prolyl cis-trans isomerase FKBP2 OS=Homo sapiens OX=9606 GN=FKBP2 PE=1 SV=2	0,682593857	1,195	1,896
O94925	Glutaminase kidney isoform, mitochondrial OS=Homo sapiens OX=9606 GN=GLS PE=1 SV=1	0,297000297	1,195	3,91
Q10570	Cleavage and polyadenylation specificity factor subunit 1 OS=Homo sapiens OX=9606 GN=CPSF1 PE=1 SV=2	1,661129568	1,194	0,716
P36578	Large ribosomal subunit protein uL4 OS=Homo sapiens OX=9606 GN=RPL4 PE=1 SV=5	1,254705144	1,193	0,817
P61978	Heterogeneous nuclear ribonucleoprotein K OS=Homo sapiens OX=9606 GN=HNRNPK PE=1 SV=1	1,226993865	1,191	0,878
O75643	U5 small nuclear ribonucleoprotein 200 kDa helicase OS=Homo sapiens OX=9606 GN=SNRNP200 PE=1 SV=2	1,85528757	1,186	0,686
Q9H2M9	Rab3 GTPase-activating protein non-catalytic subunit OS=Homo sapiens OX=9606 GN=RAB3GAP2 PE=1 SV=1	1,055966209	1,186	1,046
Q92890	Ubiquitin recognition factor in ER-associated degradation protein 1 OS=Homo sapiens OX=9606 GN=UFD1 PE=1 SV=3	0,922509225	1,184	1,258
Q99729	Heterogeneous nuclear ribonucleoprotein A/B OS=Homo sapiens OX=9606 GN=HNRNPAB PE=1 SV=2	1,736111111	1,183	0,59
P29966	Myristoylated alanine-rich C-kinase substrate OS=Homo sapiens OX=9606 GN=MARCKS PE=1 SV=4	1,428571429	1,183	0,695
P42166	Lamina-associated polypeptide 2, isoform alpha OS=Homo sapiens OX=9606 GN=TMPO PE=1 SV=2	1,642036125	1,182	0,787
P51991	Heterogeneous nuclear ribonucleoprotein A3 OS=Homo sapiens OX=9606 GN=HNRNPA3 PE=1 SV=2	1,272264631	1,181	0,979
O60841	Eukaryotic translation initiation factor 5B OS=Homo sapiens OX=9606 GN=EIF5B PE=1 SV=4	2,96735905	1,179	0,48
Q7L2E3	ATP-dependent RNA helicase DHX30 OS=Homo sapiens OX=9606 GN=DHX30 PE=1 SV=1	1,430615165	1,177	0,787
P13798	Acylamino-acid-releasing enzyme OS=Homo sapiens OX=9606 GN=APEH PE=1 SV=4	0,888888889	1,176	0,686
Q9NRG9	Aladin OS=Homo sapiens OX=9606 GN=AAAS PE=1 SV=1	1,443001443	1,172	0,747
Q8WYA6	Beta-catenin-like protein 1 OS=Homo sapiens OX=9606 GN=CTNNBL1 PE=1 SV=1	1,470588235	1,171	0,814
Q7Z2W4	Zinc finger CCCH-type antiviral protein 1 OS=Homo sapiens OX=9606 GN=ZC3HAV1 PE=1 SV=3	0,809061489	1,171	1,537
O60264	SWI/SNF-related matrix-associated actin-dependent regulator of chromatin subfamily A member 5 OS=Homo sapiens OX=96	2,232142857	1,168	0,48
O43809	Cleavage and polyadenylation specificity factor subunit 5 OS=Homo sapiens OX=9606 GN=NUDT21 PE=1 SV=1	1,540832049	1,168	0,775
Q969N2	GPI transamidase component PIG-T OS=Homo sapiens OX=9606 GN=PIGT PE=1 SV=1	0,904159132	1,168	1,006
Q8WWM7	Ataxin-2-like protein OS=Homo sapiens OX=9606 GN=ATXN2L PE=1 SV=2	1,136363636	1,167	1,086
Q00341	Vigilin OS=Homo sapiens OX=9606 GN=HDLBP PE=1 SV=3	1,023541453	1,166	1,026
P61011	Signal recognition particle subunit SRP54 OS=Homo sapiens OX=9606 GN=SRP54 PE=1 SV=1	0,88028169	1,164	1,286
P52292	Importin subunit alpha-1 OS=Homo sapiens OX=9606 GN=KPNA2 PE=1 SV=1	2,325581395	1,163	0,505
Q9BXP5	Serrate RNA effector molecule homolog OS=Homo sapiens OX=9606 GN=SRRT PE=1 SV=1	2,197802198	1,163	0,518
Q9Y5S9	RNA-binding protein 8A OS=Homo sapiens OX=9606 GN=RBM8A PE=1 SV=1	1,766784452	1,162	0,725
Q14103	Heterogeneous nuclear ribonucleoprotein D0 OS=Homo sapiens OX=9606 GN=HNRNPD PE=1 SV=1	1,394700139	1,162	0,69
O75131	Copine-3 OS=Homo sapiens OX=9606 GN=CPNE3 PE=1 SV=1	0,985221675	1,162	1,095
P43121	Cell surface glycoprotein MUC18 OS=Homo sapiens OX=9606 GN=MCAM PE=1 SV=2	0,080528265	1,162	11,131
Q9Y5K5	Ubiquitin carboxyl-terminal hydrolase isozyme L5 OS=Homo sapiens OX=9606 GN=UCHL5 PE=1 SV=3	0,881057269	1,16	1,351
Q9Y520	Protein PRRC2C OS=Homo sapiens OX=9606 GN=PRRC2C PE=1 SV=4	1,461988304	1,158	0,749
Q10567	AP-1 complex subunit beta-1 OS=Homo sapiens OX=9606 GN=AP1B1 PE=1 SV=3	0,823045267	1,158	1,143
P35613	Basigin OS=Homo sapiens OX=9606 GN=BSG PE=1 SV=2	0,447627574	1,158	2,481
P02461	Collagen alpha-1(III) chain OS=Homo sapiens OX=9606 GN=COL3A1 PE=1 SV=4	0,079846694	1,158	25,349
Q01085	Nucleolysin TIAR OS=Homo sapiens OX=9606 GN=TIAL1 PE=1 SV=1	1,481481481	1,156	0,867
P07741	Adenine phosphoribosyltransferase OS=Homo sapiens OX=9606 GN=APRT PE=1 SV=2	1,060445387	1,155	0,628
Q12955	Ankyrin-3 OS=Homo sapiens OX=9606 GN=ANK3 PE=1 SV=3	0,905797101	1,155	1,295
P35611	Alpha-adducin OS=Homo sapiens OX=9606 GN=ADD1 PE=1 SV=2	0,925925926	1,154	1,743
O14936	Peripheral plasma membrane protein CASK OS=Homo sapiens OX=9606 GN=CASK PE=1 SV=3	0,608642727	1,153	1,755
Q15459	Splicing factor 3A subunit 1 OS=Homo sapiens OX=9606 GN=SF3A1 PE=1 SV=1	1,35501355	1,151	0,773
G5E9E7	Tight junction protein 1 OS=Homo sapiens OX=9606 GN=TJP1 PE=1 SV=1	2,398081535	1,149	0,518
Q6P2Q9	Pre-mRNA-processing-splicing factor 8 OS=Homo sapiens OX=9606 GN=PRPF8 PE=1 SV=2	1,57480315	1,147	0,734
Q9BZK7	F-box-like/WD repeat-containing protein TBL1XR1 OS=Homo sapiens OX=9606 GN=TBL1XR1 PE=1 SV=1	1,259445844	1,146	0,776
Q8N3U4	Cohesin subunit SA-2 OS=Homo sapiens OX=9606 GN=STAG2 PE=1 SV=3	1,165501166	1,146	0,939
Q9NYU2	UDP-glucose:glycoprotein glucosyltransferase 1 OS=Homo sapiens OX=9606 GN=UGGT1 PE=1 SV=3	0,762195122	1,146	1,543
P49755	Transmembrane emp24 domain-containing protein 10 OS=Homo sapiens OX=9606 GN=TMED10 PE=1 SV=2	0,711237553	1,146	1,508
Q14137	Ribosome biogenesis protein BOP1 OS=Homo sapiens OX=9606 GN=BOP1 PE=1 SV=2	2,816901408	1,145	0,408
Q9NZW5	Protein PALS2 OS=Homo sapiens OX=9606 GN=PALS2 PE=1 SV=2	0,842459983	1,145	1,482
Q9Y5B9	FACT complex subunit SPT16 OS=Homo sapiens OX=9606 GN=SUPT16H PE=1 SV=1	1,968503937	1,144	0,523
P32322	Pyrroline-5-carboxylate reductase 1, mitochondrial OS=Homo sapiens OX=9606 GN=PYCR1 PE=1 SV=2	0,866551127	1,144	1,509
O75396	Vesicle-trafficking protein SEC22b OS=Homo sapiens OX=9606 GN=SEC22B PE=1 SV=5	0,673400673	1,144	1,648
Q07812	Apoptosis regulator BAX OS=Homo sapiens OX=9606 GN=BAX PE=1 SV=1	1,082251082	1,143	0,996
O75934	Pre-mRNA-splicing factor SPF27 OS=Homo sapiens OX=9606 GN=BCAS2 PE=1 SV=1	1,157407407	1,142	1,27
Q96IZ0	PRKC apoptosis WT1 regulator protein OS=Homo sapiens OX=9606 GN=PAWR PE=1 SV=1	0,472143532	1,142	2,341
P62263	Small ribosomal subunit protein uS11 OS=Homo sapiens OX=9606 GN=RPS14 PE=1 SV=3	1,179245283	1,141	0,94
Q14566	DNA replication licensing factor MCM6 OS=Homo sapiens OX=9606 GN=MCM6 PE=1 SV=1	1,945525292	1,14	0,539
Q969X5	Endoplasmic reticulum-Golgi intermediate compartment protein 1 OS=Homo sapiens OX=9606 GN=ERGIC1 PE=1 SV=1	1,923076923	1,137	0,561
Q15029	116 kDa U5 small nuclear ribonucleoprotein component OS=Homo sapiens OX=9606 GN=EFTUD2 PE=1 SV=1	1,497005988	1,137	0,785
Q12904	Aminoacyl tRNA synthase complex-interacting multifunctional protein 1 OS=Homo sapiens OX=9606 GN=AIMP1 PE=1 SV=2	0,913242009	1,137	1,343
O60762	Dolichol-phosphate mannosyltransferase subunit 1 OS=Homo sapiens OX=9606 GN=DPM1 PE=1 SV=1	0,767459708	1,137	1,345
P30419	Glycylpeptide N-tetradecanoyltransferase 1 OS=Homo sapiens OX=9606 GN=NMT1 PE=1 SV=2	1,865671642	1,136	0,663
P46087	Probable 28S rRNA (cytosine(4447)-C(5))-methyltransferase OS=Homo sapiens OX=9606 GN=NOP2 PE=1 SV=2	1,721170396	1,135	0,572
Q07020	Large ribosomal subunit protein eL18 OS=Homo sapiens OX=9606 GN=RPL18 PE=1 SV=2	1,245330012	1,133	0,816
Q9BXS5	AP-1 complex subunit mu-1 OS=Homo sapiens OX=9606 GN=AP1M1 PE=1 SV=3	0,782472613	1,132	0,711
Q6L8Q7	2',5'-phosphodiesterase 12 OS=Homo sapiens OX=9606 GN=PDE12 PE=1 SV=2	1,267427123	1,129	0,868
P40763	Signal transducer and activator of transcription 3 OS=Homo sapiens OX=9606 GN=STAT3 PE=1 SV=2	0,556792873	1,128	2,04
P55884	Eukaryotic translation initiation factor 3 subunit B OS=Homo sapiens OX=9606 GN=EIF3B PE=1 SV=3	1,996007984	1,127	0,577

O60832	H/ACA ribonucleoprotein complex subunit DKC1 OS=Homo sapiens OX=9606 GN=DKC1 PE=1 SV=3	1,724137931	1,127	0,637
P30044	Peroxiredoxin-5, mitochondrial OS=Homo sapiens OX=9606 GN=PRDX5 PE=1 SV=4	0,755287009	1,127	1,557
P12268	Inosine-5'-monophosphate dehydrogenase 2 OS=Homo sapiens OX=9606 GN=IMPDH2 PE=1 SV=2	1,692047377	1,126	0,715
P33316	Deoxyuridine 5'-triphosphate nucleotidohydrolase, mitochondrial OS=Homo sapiens OX=9606 GN=DUT PE=1 SV=4	1,6	1,125	0,827
Q8TC12	Retinol dehydrogenase 11 OS=Homo sapiens OX=9606 GN=RDH11 PE=1 SV=2	1,506024096	1,125	0,559
Q15007	Pre-mRNA-splicing regulator WTAP OS=Homo sapiens OX=9606 GN=WTAP PE=1 SV=2	1,503759398	1,124	0,799
Q6UVK1	Chondroitin sulfate proteoglycan 4 OS=Homo sapiens OX=9606 GN=CSPG4 PE=1 SV=2	0,192233756	1,123	5,155
P51149	Ras-related protein Rab-7a OS=Homo sapiens OX=9606 GN=RAB7A PE=1 SV=1	0,845308538	1,12	1,288
Q14444	Caprin-1 OS=Homo sapiens OX=9606 GN=CAPRIN1 PE=1 SV=2	1,689189189	1,117	0,709
P62424	Large ribosomal subunit protein eL8 OS=Homo sapiens OX=9606 GN=RPL7A PE=1 SV=2	1,658374793	1,117	0,605
Q15738	Sterol-4-alpha-carboxylate 3-dehydrogenase, decarboxylating OS=Homo sapiens OX=9606 GN=NSDHL PE=1 SV=2	1,610305958	1,116	0,664
Q99829	Copine-1 OS=Homo sapiens OX=9606 GN=CPNE1 PE=1 SV=1	2,028397566	1,115	0,554
O15371	Eukaryotic translation initiation factor 3 subunit D OS=Homo sapiens OX=9606 GN=EIF3D PE=1 SV=1	1,776198934	1,115	0,596
Q14320	Protein FAM50A OS=Homo sapiens OX=9606 GN=FAM50A PE=1 SV=2	1,594896332	1,115	0,741
P43897	Elongation factor Ts, mitochondrial OS=Homo sapiens OX=9606 GN=TSFM PE=1 SV=2	0,64516129	1,115	1,713
Q07157	Tight junction protein ZO-1 OS=Homo sapiens OX=9606 GN=TJP1 PE=1 SV=3	2,237136465	1,11	0,5
P49750	YLP motif-containing protein 1 OS=Homo sapiens OX=9606 GN=YLPM1 PE=1 SV=4	1,468428781	1,11	0,793
Q02818	Nucleobindin-1 OS=Homo sapiens OX=9606 GN=NUCB1 PE=1 SV=4	0,901713255	1,11	1,151
A0A9L9PXH6	Nucleobindin-1 OS=Homo sapiens OX=9606 GN=NUCB1 PE=1 SV=1	0,871839582	1,11	1,167
P41252	Isoleucine--tRNA ligase, cytoplasmic OS=Homo sapiens OX=9606 GN=IARS1 PE=1 SV=2	1,223990208	1,108	0,883
P61289	Proteasome activator complex subunit 3 OS=Homo sapiens OX=9606 GN=PSME3 PE=1 SV=1	1,092896175	1,107	1,082
Q6PKG0	La-related protein 1 OS=Homo sapiens OX=9606 GN=LARP1 PE=1 SV=2	2,392344498	1,106	0,629
Q8WXF1	Paraspeckle component 1 OS=Homo sapiens OX=9606 GN=PSPC1 PE=1 SV=1	1,828153565	1,105	0,546
P08865	Small ribosomal subunit protein uS2 OS=Homo sapiens OX=9606 GN=RPSA PE=1 SV=4	1,694915254	1,105	0,75
P35250	Replication factor C subunit 2 OS=Homo sapiens OX=9606 GN=RFC2 PE=1 SV=3	1,538461538	1,105	0,606
P38159	RNA-binding motif protein, X chromosome OS=Homo sapiens OX=9606 GN=RBMX PE=1 SV=3	1,34589502	1,105	0,806
Q96PZ0	Pseudouridylate synthase 7 homolog OS=Homo sapiens OX=9606 GN=PUS7 PE=1 SV=2	1,206272618	1,105	0,824
P51572	B-cell receptor-associated protein 31 OS=Homo sapiens OX=9606 GN=BCAP31 PE=1 SV=3	0,71787509	1,105	1,663
Q9NVI1	Fanconi anemia group I protein OS=Homo sapiens OX=9606 GN=FANCI PE=1 SV=4	2,78551532	1,103	0,436
Q92522	Histone H1.10 OS=Homo sapiens OX=9606 GN=H1-10 PE=1 SV=1	1,589825119	1,103	0,728
O15372	Eukaryotic translation initiation factor 3 subunit H OS=Homo sapiens OX=9606 GN=EIF3H PE=1 SV=1	1,315789474	1,1	0,793
P61247	Small ribosomal subunit protein eS1 OS=Homo sapiens OX=9606 GN=RPS3A PE=1 SV=2	1,642036125	1,095	0,606
Q00610	Clathrin heavy chain 1 OS=Homo sapiens OX=9606 GN=CLTC PE=1 SV=5	0,621118012	1,095	1,698
Q08211	ATP-dependent RNA helicase A OS=Homo sapiens OX=9606 GN=DHX9 PE=1 SV=4	1,107419712	1,093	0,985
P25205	DNA replication licensing factor MCM3 OS=Homo sapiens OX=9606 GN=MCM3 PE=1 SV=3	2,304147465	1,091	0,453
P63244	Small ribosomal subunit protein RACK1 OS=Homo sapiens OX=9606 GN=RACK1 PE=1 SV=3	1,191895113	1,091	0,826
P17858	ATP-dependent 6-phosphofructokinase, liver type OS=Homo sapiens OX=9606 GN=PFKL PE=1 SV=6	0,959692898	1,089	1,19
Q96DI7	U5 small nuclear ribonucleoprotein 40 kDa protein OS=Homo sapiens OX=9606 GN=SNRNP40 PE=1 SV=1	1,490312966	1,088	0,87
Q16836	Hydroxyacyl-coenzyme A dehydrogenase, mitochondrial OS=Homo sapiens OX=9606 GN=HADH PE=1 SV=3	1,161440186	1,088	0,756
P13073	Cytochrome c oxidase subunit 4 isoform 1, mitochondrial OS=Homo sapiens OX=9606 GN=COX4I1 PE=1 SV=1	1	1,088	1,122
Q96S52	GPI transamidase component PIG-S OS=Homo sapiens OX=9606 GN=PIGS PE=1 SV=3	0,777604977	1,088	1,273
Q14671	Pumilio homolog 1 OS=Homo sapiens OX=9606 GN=PUM1 PE=1 SV=3	1,360544218	1,086	0,721
Q14498	RNA-binding protein 39 OS=Homo sapiens OX=9606 GN=RBM39 PE=1 SV=2	1,709401709	1,085	1,049
Q14974	Importin subunit beta-1 OS=Homo sapiens OX=9606 GN=KPNB1 PE=1 SV=2	1,295336788	1,084	0,863
P69905	Hemoglobin subunit alpha OS=Homo sapiens OX=9606 GN=HBA1 PE=1 SV=2	0,203707476	1,083	4,375
O43143	ATP-dependent RNA helicase DHX15 OS=Homo sapiens OX=9606 GN=DHX15 PE=1 SV=2	2,631578947	1,082	0,437
O43684	Mitotic checkpoint protein BUB3 OS=Homo sapiens OX=9606 GN=BUB3 PE=1 SV=1	1,930501931	1,081	0,679
Q14651	Plastin-1 OS=Homo sapiens OX=9606 GN=PLS1 PE=1 SV=2	1,422475107	1,081	0,64
O14776	Transcription elongation regulator 1 OS=Homo sapiens OX=9606 GN=TCERG1 PE=1 SV=2	1,650165017	1,08	0,69
P30040	Endoplasmic reticulum resident protein 29 OS=Homo sapiens OX=9606 GN=ERP29 PE=1 SV=4	1,577287066	1,079	0,679
Q5RKV6	Exosome complex component MTR3 OS=Homo sapiens OX=9606 GN=EXOSC6 PE=1 SV=1	1,156069364	1,079	0,747
P84243	Histone H3.3 OS=Homo sapiens OX=9606 GN=H3-3A PE=1 SV=2	3,533568905	1,075	0,2
Q9UQE7	Structural maintenance of chromosomes protein 3 OS=Homo sapiens OX=9606 GN=SMC3 PE=1 SV=2	1,373626374	1,075	0,765
P07237	Protein disulfide-isomerase OS=Homo sapiens OX=9606 GN=P4HB PE=1 SV=3	0,413736036	1,073	2,583
P11498	Pyruvate carboxylase, mitochondrial OS=Homo sapiens OX=9606 GN=PC PE=1 SV=2	0,480307397	1,07	2,117
Q6P2E9	Enhancer of mRNA-decapping protein 4 OS=Homo sapiens OX=9606 GN=EDC4 PE=1 SV=1	1,930501931	1,069	0,623
P26373	Large ribosomal subunit protein eL13 OS=Homo sapiens OX=9606 GN=RPL13 PE=1 SV=4	1,278772379	1,069	0,69
P46939	Utrophin OS=Homo sapiens OX=9606 GN=UTRN PE=1 SV=2	0,277469478	1,066	3,005
Q14534	Squalene monooxygenase OS=Homo sapiens OX=9606 GN=SQLE PE=1 SV=3	2,544529262	1,063	0,382
P05387	Large ribosomal subunit protein P2 OS=Homo sapiens OX=9606 GN=RPLP2 PE=1 SV=1	1,129943503	1,063	1,062
O43776	Asparagine--tRNA ligase, cytoplasmic OS=Homo sapiens OX=9606 GN=NARS1 PE=1 SV=1	1,371742112	1,062	0,743
P40222	Alpha-taxilin OS=Homo sapiens OX=9606 GN=TXLNA PE=1 SV=3	1,317523057	1,061	0,847
P50851	Lipopolysaccharide-responsive and beige-like anchor protein OS=Homo sapiens OX=9606 GN=LRBA PE=1 SV=4	1,129943503	1,057	0,738
P82675	Small ribosomal subunit protein uS5m OS=Homo sapiens OX=9606 GN=MRPS5 PE=1 SV=2	1,328021248	1,056	0,389
P36543	V-type proton ATPase subunit E 1 OS=Homo sapiens OX=9606 GN=ATP6V1E1 PE=1 SV=1	0,961538462	1,055	1,232
P52907	F-actin-capping protein subunit alpha-1 OS=Homo sapiens OX=9606 GN=CAPZA1 PE=1 SV=3	0,833333333	1,055	1,185
P32969	Large ribosomal subunit protein uL6 OS=Homo sapiens OX=9606 GN=RPL9 PE=1 SV=1	1,016260163	1,053	1,252
Q9UJS0	Electrogenic aspartate/glutamate antiporter SLC25A13, mitochondrial OS=Homo sapiens OX=9606 GN=SLC25A13 PE=1 SV=2	2,380952381	1,052	0,434
Q9UJ70	N-acetyl-D-glucosamine kinase OS=Homo sapiens OX=9606 GN=NAGK PE=1 SV=4	0,868809731	1,05	1,308
P08559	Pyruvate dehydrogenase E1 component subunit alpha, somatic form, mitochondrial OS=Homo sapiens OX=9606 GN=PDHA1	1,156069364	1,049	0,818
P42167	Lamina-associated polypeptide 2, isoforms beta/gamma OS=Homo sapiens OX=9606 GN=TMPO PE=1 SV=2	1,552795031	1,048	0,765
Q14008	Cytoskeleton-associated protein 5 OS=Homo sapiens OX=9606 GN=CKAP5 PE=1 SV=3	1,314060447	1,047	0,816
P51114	RNA-binding protein FXR1 OS=Homo sapiens OX=9606 GN=FXR1 PE=1 SV=3	1,261034048	1,047	0,86
O75533	Splicing factor 3B subunit 1 OS=Homo sapiens OX=9606 GN=SF3B1 PE=1 SV=3	1,344086022	1,046	0,695
P61619	Protein transport protein Sec61 subunit alpha isoform 1 OS=Homo sapiens OX=9606 GN=SEC61A1 PE=1 SV=2	0,543183053	1,045	1,573
Q04637	Eukaryotic translation initiation factor 4 gamma 1 OS=Homo sapiens OX=9606 GN=EIF4G1 PE=1 SV=4	1,297016861	1,044	0,78
P25789	Proteasome subunit alpha type-4 OS=Homo sapiens OX=9606 GN=PSMA4 PE=1 SV=1	0,89206066	1,044	1,145
P55809	Succinyl-CoA:3-ketoacid coenzyme A transferase 1, mitochondrial OS=Homo sapiens OX=9606 GN=OXCT1 PE=1 SV=1	0,344708721	1,043	3,078
Q96SI9	Spermatid perinuclear RNA-binding protein OS=Homo sapiens OX=9606 GN=STRBP PE=1 SV=1	2,590673575	1,042	0,417
Q6NVY1	3-hydroxyisobutyryl-CoA hydrolase, mitochondrial OS=Homo sapiens OX=9606 GN=HIBCH PE=1 SV=2	1,828153565	1,042	0,668
Q969Z0	FAST kinase domain-containing protein 4 OS=Homo sapiens OX=9606 GN=TBRG4 PE=1 SV=1	1,742160279	1,042	0,597
Q9NQC3	Reticulon-4 OS=Homo sapiens OX=9606 GN=RTN4 PE=1 SV=2	0,416666667	1,042	1,517
Q12874	Splicing factor 3A subunit 3 OS=Homo sapiens OX=9606 GN=SF3A3 PE=1 SV=1	1,582278481	1,041	0,697

Q96T23	Remodeling and spacing factor 1 OS=Homo sapiens OX=9606 GN=RSF1 PE=1 SV=2	1,594896332	1,039	0,653
Q15393	Splicing factor 3B subunit 3 OS=Homo sapiens OX=9606 GN=SF3B3 PE=1 SV=4	1,166861144	1,039	0,806
Q9BTX1	Nucleoporin NDC1 OS=Homo sapiens OX=9606 GN=NDC1 PE=1 SV=2	2,070393375	1,038	0,767
P13995	Bifunctional methylenetetrahydrofolate dehydrogenase/cyclohydrolase, mitochondrial OS=Homo sapiens OX=9606 GN=MT	1,037344398	1,038	1,326
Q6UWP7	Lysocardiolipin acyltransferase 1 OS=Homo sapiens OX=9606 GN=LCLAT1 PE=1 SV=1	1,018329939	1,036	1,198
Q96A72	Protein mago nashi homolog 2 OS=Homo sapiens OX=9606 GN=MAGOHB PE=1 SV=1	1,485884101	1,034	0,669
O76094	Signal recognition particle subunit SRP72 OS=Homo sapiens OX=9606 GN=SRP72 PE=1 SV=3	1,821493625	1,033	0,531
Q96KR1	Zinc finger RNA-binding protein OS=Homo sapiens OX=9606 GN=ZFR PE=1 SV=2	1,569858713	1,033	0,587
Q9Y3A6	Transmembrane emp24 domain-containing protein 5 OS=Homo sapiens OX=9606 GN=TMED5 PE=1 SV=1	0,819000819	1,033	1,263
Q9Y371	Endophilin-B1 OS=Homo sapiens OX=9606 GN=SH3GLB1 PE=1 SV=1	0,631313131	1,033	2,209
Q9UKL0	REST corepressor 1 OS=Homo sapiens OX=9606 GN=RCOR1 PE=1 SV=2	1,39275766	1,032	0,941
Q14683	Structural maintenance of chromosomes protein 1A OS=Homo sapiens OX=9606 GN=SMC1A PE=1 SV=2	1,38121547	1,032	0,769
Q9GZS3	Superkiller complex protein 8 OS=Homo sapiens OX=9606 GN=SKIC8 PE=1 SV=1	0,801924619	1,031	1,359
Q9BXW7	Haloacid dehalogenase-like hydrolase domain-containing 5 OS=Homo sapiens OX=9606 GN=HDHD5 PE=1 SV=1	2,352941176	1,029	0,317
Q9H0S4	Probable ATP-dependent RNA helicase DDX47 OS=Homo sapiens OX=9606 GN=DDX47 PE=1 SV=1	2	1,029	0,591
Q02878	Large ribosomal subunit protein eL6 OS=Homo sapiens OX=9606 GN=RPL6 PE=1 SV=3	1,45137881	1,029	0,815
P56537	Eukaryotic translation initiation factor 6 OS=Homo sapiens OX=9606 GN=EIF6 PE=1 SV=1	1,047120419	1,029	0,983
P06737	Glycogen phosphorylase, liver form OS=Homo sapiens OX=9606 GN=PYGL PE=1 SV=4	0,554938957	1,027	1,877
Q9Y5M8	Signal recognition particle receptor subunit beta OS=Homo sapiens OX=9606 GN=SRPRB PE=1 SV=3	0,743494424	1,026	1,283
P67809	Y-box-binding protein 1 OS=Homo sapiens OX=9606 GN=YBX1 PE=1 SV=3	1,589825119	1,023	0,598
Q9NTJ3	Structural maintenance of chromosomes protein 4 OS=Homo sapiens OX=9606 GN=SMC4 PE=1 SV=2	1,246882793	1,023	0,673
Q8IY67	Ribonucleoprotein PTB-binding 1 OS=Homo sapiens OX=9606 GN=RAVER1 PE=1 SV=1	0,778816199	1,021	1,092
P31942	Heterogeneous nuclear ribonucleoprotein H3 OS=Homo sapiens OX=9606 GN=HNRNPH3 PE=1 SV=2	1,27388535	1,02	0,877
P10515	Dihydrolipoyllysine-residue acetyltransferase component of pyruvate dehydrogenase complex, mitochondrial OS=Homo sap	1,062699256	1,02	0,909
P36871	Phosphoglucomutase-1 OS=Homo sapiens OX=9606 GN=PGM1 PE=1 SV=3	0,74682599	1,02	1,268
O75694	Nuclear pore complex protein Nup155 OS=Homo sapiens OX=9606 GN=NUP155 PE=1 SV=1	1,098901099	1,019	0,916
O94905	Erlin-2 OS=Homo sapiens OX=9606 GN=ERLIN2 PE=1 SV=1	0,576368876	1,019	1,538
A0A8V8TNT7	WD repeat domain 1 OS=Homo sapiens OX=9606 GN=WDR1 PE=1 SV=1	0,720461095	1,016	1,294
Q9Y265	RuvB-like 1 OS=Homo sapiens OX=9606 GN=RUVBL1 PE=1 SV=1	1,278772379	1,015	0,793
Q02809	Procollagen-lysine,2-oxoglutarate 5-dioxygenase 1 OS=Homo sapiens OX=9606 GN=PLOD1 PE=1 SV=2	0,399680256	1,013	3,038
Q96HC4	PDZ and LIM domain protein 5 OS=Homo sapiens OX=9606 GN=PDLIM5 PE=1 SV=5	0,259134491	1,013	3,022
O14974	Protein phosphatase 1 regulatory subunit 12A OS=Homo sapiens OX=9606 GN=PPP1R12A PE=1 SV=1	0,59311981	1,01	1,819
Q8WX92	Negative elongation factor B OS=Homo sapiens OX=9606 GN=NELFB PE=1 SV=1	1,172332943	1,009	0,818
Q9NTI5	Sister chromatid cohesion protein PDS5 homolog B OS=Homo sapiens OX=9606 GN=PDS5B PE=1 SV=1	1,308900524	1,006	0,774
Q86U42	Polyadenylate-binding protein 2 OS=Homo sapiens OX=9606 GN=PABPN1 PE=1 SV=3	1,044932079	1,005	0,983
O95347	Structural maintenance of chromosomes protein 2 OS=Homo sapiens OX=9606 GN=SMC2 PE=1 SV=2	1,338688086	1,004	0,822
O95302	Peptidyl-prolyl cis-trans isomerase FKBP9 OS=Homo sapiens OX=9606 GN=FKBP9 PE=1 SV=2	0,765696784	1,003	1,299
P08779	Keratin, type I cytoskeletal 16 OS=Homo sapiens OX=9606 GN=KRT16 PE=1 SV=4	0,080392314	1,003	12,011
P60228	Eukaryotic translation initiation factor 3 subunit E OS=Homo sapiens OX=9606 GN=EIF3E PE=1 SV=1	1,512859304	1	0,733
P31689	DnaJ homolog subfamily A member 1 OS=Homo sapiens OX=9606 GN=DNAJA1 PE=1 SV=2	1,984126984	0,999	0,567
P00167	Cytochrome b5 OS=Homo sapiens OX=9606 GN=CYB5A PE=1 SV=2	0,351493849	0,997	2,867
Q15154	Pericentriolar material 1 protein OS=Homo sapiens OX=9606 GN=PCM1 PE=1 SV=6	1,044932079	0,995	0,289
Q13573	SNW domain-containing protein 1 OS=Homo sapiens OX=9606 GN=SNW1 PE=1 SV=1	1,584786054	0,993	0,624
Q86V81	THO complex subunit 4 OS=Homo sapiens OX=9606 GN=ALYREF PE=1 SV=3	0,981354269	0,992	0,936
Q9Y262	Eukaryotic translation initiation factor 3 subunit L OS=Homo sapiens OX=9606 GN=EIF3L PE=1 SV=1	1,592356688	0,991	0,727
P18124	Large ribosomal subunit protein uL30 OS=Homo sapiens OX=9606 GN=RPL7 PE=1 SV=1	1,375515818	0,991	0,617
P01111	GTPase NRas OS=Homo sapiens OX=9606 GN=NRAS PE=1 SV=1	0,928505107	0,991	1,07
O60814	Histone H2B type 1-K OS=Homo sapiens OX=9606 GN=H2BC12 PE=1 SV=3	2,049180328	0,99	0,499
Q03701	CCAAT/enhancer-binding protein zeta OS=Homo sapiens OX=9606 GN=CEBPZ PE=1 SV=3	2,801120448	0,989	0,318
Q969V3	BOS complex subunit NCLN OS=Homo sapiens OX=9606 GN=NCLN PE=1 SV=2	0,697836706	0,988	1,326
P35637	RNA-binding protein FUS OS=Homo sapiens OX=9606 GN=FUS PE=1 SV=1	1,497005988	0,986	0,404
Q92542	Nicastrin OS=Homo sapiens OX=9606 GN=NCSTN PE=1 SV=2	0,563380282	0,986	1,715
O95232	Luc7-like protein 3 OS=Homo sapiens OX=9606 GN=LUC7L3 PE=1 SV=2	2,159827214	0,985	0,432
P25325	3-mercaptopyruvate sulfurtransferase OS=Homo sapiens OX=9606 GN=MPST PE=1 SV=3	1,602564103	0,984	0,59
Q7L576	Cytoplasmic FMR1-interacting protein 1 OS=Homo sapiens OX=9606 GN=CYFIP1 PE=1 SV=1	0,978473581	0,984	1,047
O75475	PC4 and SFRS1-interacting protein OS=Homo sapiens OX=9606 GN=PSIP1 PE=1 SV=1	1,703577513	0,983	0,569
P27824	Calnexin OS=Homo sapiens OX=9606 GN=CANX PE=1 SV=2	1,644736842	0,983	0,634
Q8N1F7	Nuclear pore complex protein Nup93 OS=Homo sapiens OX=9606 GN=NUP93 PE=1 SV=2	1,371742112	0,982	0,734
P38919	Eukaryotic initiation factor 4A-III OS=Homo sapiens OX=9606 GN=EIF4A3 PE=1 SV=4	1,236093943	0,982	0,681
P23743	Diacylglycerol kinase alpha OS=Homo sapiens OX=9606 GN=DGKA PE=1 SV=3	0,371747212	0,982	3,134
Q9NUJ1	Palmitoyl-protein thioesterase ABHD10, mitochondrial OS=Homo sapiens OX=9606 GN=ABHD10 PE=1 SV=1	0,685871056	0,978	1,472
P47755	F-actin-capping protein subunit alpha-2 OS=Homo sapiens OX=9606 GN=CAPZA2 PE=1 SV=3	0,631313131	0,976	1,586
Q9NTX5	Ethylmalonyl-CoA decarboxylase OS=Homo sapiens OX=9606 GN=ECHDC1 PE=1 SV=2	1,029866117	0,975	0,98
Q8N3C0	Activating signal cointegrator 1 complex subunit 3 OS=Homo sapiens OX=9606 GN=ASCC3 PE=1 SV=3	1,39275766	0,974	1,015
Q9UBS4	DnaJ homolog subfamily B member 11 OS=Homo sapiens OX=9606 GN=DNAJB11 PE=1 SV=1	0,73964497	0,973	1,554
O95994	Anterior gradient protein 2 homolog OS=Homo sapiens OX=9606 GN=AGR2 PE=1 SV=1	0,654878847	0,972	1,566
P55786	Puromycin-sensitive aminopeptidase OS=Homo sapiens OX=9606 GN=NPEPPS PE=1 SV=2	0,825763832	0,97	1,149
P21127	Cyclin-dependent kinase 11B OS=Homo sapiens OX=9606 GN=CDK11B PE=1 SV=4	4,716981132	0,967	0,621
P49736	DNA replication licensing factor MCM2 OS=Homo sapiens OX=9606 GN=MCM2 PE=1 SV=4	1,988071571	0,967	0,508
P19338	Nucleolin OS=Homo sapiens OX=9606 GN=NCL PE=1 SV=3	3,174603175	0,966	0,287
Q08257	Quinone oxidoreductase OS=Homo sapiens OX=9606 GN=CRYZ PE=1 SV=1	1,290322581	0,966	0,695
O14773	Tripeptidyl-peptidase 1 OS=Homo sapiens OX=9606 GN=TPP1 PE=1 SV=2	0,278862242	0,966	3,15
Q96ST3	Paired amphipathic helix protein Sin3a OS=Homo sapiens OX=9606 GN=SIN3A PE=1 SV=2	1,886792453	0,964	0,486
P54136	Arginine--tRNA ligase, cytoplasmic OS=Homo sapiens OX=9606 GN=RARS1 PE=1 SV=2	1,331557923	0,961	0,768
P31946	14-3-3 protein beta/alpha OS=Homo sapiens OX=9606 GN=YWHAB PE=1 SV=3	0,947867299	0,961	1,084
Q9H8Y8	Golgi reassembly-stacking protein 2 OS=Homo sapiens OX=9606 GN=GORASP2 PE=1 SV=3	0,968054211	0,96	1,048
P47756	F-actin-capping protein subunit beta OS=Homo sapiens OX=9606 GN=CAPZB PE=1 SV=5	0,847457627	0,96	1,113
P49023	Paxillin OS=Homo sapiens OX=9606 GN=PXN PE=1 SV=3	0,393545848	0,96	3,173
P41091	Eukaryotic translation initiation factor 2 subunit 3 OS=Homo sapiens OX=9606 GN=EIF2S3 PE=1 SV=3	1,138952164	0,959	0,819
Q9H0U3	Magnesium transporter protein 1 OS=Homo sapiens OX=9606 GN=MAGT1 PE=1 SV=1	1,082251082	0,958	1,268
P27797	Calreticulin OS=Homo sapiens OX=9606 GN=CALR PE=1 SV=1	0,442869796	0,956	2,201
Q5BKZ1	DBIRD complex subunit ZNF326 OS=Homo sapiens OX=9606 GN=ZNF326 PE=1 SV=2	1,628664495	0,955	0,564
Q9NQG5	Regulation of nuclear pre-mRNA domain-containing protein 1B OS=Homo sapiens OX=9606 GN=RPRD1B PE=1 SV=1	1,388888889	0,954	0,796

Q9Y3L3	SH3 domain-binding protein 1 OS=Homo sapiens OX=9606 GN=SH3BP1 PE=1 SV=3	1,897533207	0,953	0,49
P35232	Prohibitin 1 OS=Homo sapiens OX=9606 GN=PHB1 PE=1 SV=1	1,040582726	0,953	0,921
Q93009	Ubiquitin carboxyl-terminal hydrolase 7 OS=Homo sapiens OX=9606 GN=USP7 PE=1 SV=2	0,900090009	0,953	1,044
Q96PC5	Melanoma inhibitory activity protein 2 OS=Homo sapiens OX=9606 GN=MIA2 PE=1 SV=4	0,449438202	0,953	1,39
P11021	Endoplasmic reticulum chaperone BiP OS=Homo sapiens OX=9606 GN=HSPA5 PE=1 SV=2	0,64061499	0,952	1,335
Q86UE4	Protein LYRIC OS=Homo sapiens OX=9606 GN=MTDH PE=1 SV=2	0,744047619	0,95	1,17
O43674	NADH dehydrogenase [ubiquinone] 1 beta subcomplex subunit 5, mitochondrial OS=Homo sapiens OX=9606 GN=NDUFB5 P	0,684931507	0,95	1,074
P10809	60 kDa heat shock protein, mitochondrial OS=Homo sapiens OX=9606 GN=HSPD1 PE=1 SV=2	2,192982456	0,948	0,339
P00367	Glutamate dehydrogenase 1, mitochondrial OS=Homo sapiens OX=9606 GN=GLUD1 PE=1 SV=2	0,841042893	0,947	0,838
P62316	Small nuclear ribonucleoprotein Sm D2 OS=Homo sapiens OX=9606 GN=SNRPD2 PE=1 SV=1	1,275510204	0,945	0,789
P17612	cAMP-dependent protein kinase catalytic subunit alpha OS=Homo sapiens OX=9606 GN=PRKACA PE=1 SV=2	0,686341798	0,945	1,293
O14828	Secretory carrier-associated membrane protein 3 OS=Homo sapiens OX=9606 GN=SCAMP3 PE=1 SV=3	0,407664085	0,945	2,419
Q14847	LIM and SH3 domain protein 1 OS=Homo sapiens OX=9606 GN=LASP1 PE=1 SV=2	1,028806584	0,944	0,693
A6NHR9	Structural maintenance of chromosomes flexible hinge domain-containing protein 1 OS=Homo sapiens OX=9606 GN=SMCH	0,816993464	0,944	1,093
Q7L014	Probable ATP-dependent RNA helicase DDX46 OS=Homo sapiens OX=9606 GN=DDX46 PE=1 SV=2	1,555209953	0,943	0,622
Q9Y3T9	Nucleolar complex protein 2 homolog OS=Homo sapiens OX=9606 GN=NOC2L PE=1 SV=4	1,937984496	0,942	0,507
P35249	Replication factor C subunit 4 OS=Homo sapiens OX=9606 GN=RFC4 PE=1 SV=2	1,587301587	0,941	0,578
Q9BY77	Polymerase delta-interacting protein 3 OS=Homo sapiens OX=9606 GN=POLDIP3 PE=1 SV=2	1,692047377	0,94	0,601
P40429	Large ribosomal subunit protein uL13 OS=Homo sapiens OX=9606 GN=RPL13A PE=1 SV=2	2,5	0,937	0,55
Q9H3N1	Thioredoxin-related transmembrane protein 1 OS=Homo sapiens OX=9606 GN=TMX1 PE=1 SV=1	0,737463127	0,937	1,139
P14868	Aspartate--tRNA ligase, cytoplasmic OS=Homo sapiens OX=9606 GN=DARS1 PE=1 SV=2	1,10864745	0,936	0,862
P16615	Sarcoplasmic/endoplasmic reticulum calcium ATPase 2 OS=Homo sapiens OX=9606 GN=ATP2A2 PE=1 SV=1	0,484027106	0,936	1,967
Q9Y3Y2	Chromatin target of PRMT1 protein OS=Homo sapiens OX=9606 GN=CHTOP PE=1 SV=2	2,036659878	0,935	0,727
O43148	mRNA cap guanine-N7 methyltransferase OS=Homo sapiens OX=9606 GN=RNMT PE=1 SV=1	1,6	0,934	0,429
P49257	Protein ERGIC-53 OS=Homo sapiens OX=9606 GN=LMAN1 PE=1 SV=2	0,768049155	0,934	1,275
O14964	Hepatocyte growth factor-regulated tyrosine kinase substrate OS=Homo sapiens OX=9606 GN=HGS PE=1 SV=1	0,700280112	0,933	1,397
P68400	Casein kinase II subunit alpha OS=Homo sapiens OX=9606 GN=CSNK2A1 PE=1 SV=1	1,303780965	0,931	0,638
Q13310	Polyadenylate-binding protein 4 OS=Homo sapiens OX=9606 GN=PABPC4 PE=1 SV=1	1,733102253	0,93	0,485
P68431	Histone H3.1 OS=Homo sapiens OX=9606 GN=H3C1 PE=1 SV=2	2,155172414	0,928	0,333
P24928	DNA-directed RNA polymerase II subunit RPB1 OS=Homo sapiens OX=9606 GN=POLR2A PE=1 SV=2	1,312335958	0,928	0,661
Q9HCU5	Prolactin regulatory element-binding protein OS=Homo sapiens OX=9606 GN=PREB PE=1 SV=2	0,922509225	0,928	0,744
O95793	Double-stranded RNA-binding protein Staufen homolog 1 OS=Homo sapiens OX=9606 GN=STAU1 PE=1 SV=2	1,45137881	0,924	0,633
P46459	Vesicle-fusing ATPase OS=Homo sapiens OX=9606 GN=NSF PE=1 SV=3	0,610873549	0,924	1,372
Q9UBU9	Nuclear RNA export factor 1 OS=Homo sapiens OX=9606 GN=NXF1 PE=1 SV=1	1,291989664	0,923	0,631
P98175	RNA-binding protein 10 OS=Homo sapiens OX=9606 GN=RBM10 PE=1 SV=3	1,618122977	0,922	0,543
O60216	Double-strand-break repair protein rad21 homolog OS=Homo sapiens OX=9606 GN=RAD21 PE=1 SV=2	1,126126126	0,92	0,831
P30048	Thioredoxin-dependent peroxide reductase, mitochondrial OS=Homo sapiens OX=9606 GN=PRDX3 PE=1 SV=3	0,534473544	0,92	1,685
Q96HS1	Serine/threonine-protein phosphatase PGAM5, mitochondrial OS=Homo sapiens OX=9606 GN=PGAM5 PE=1 SV=2	1,492537313	0,919	0,694
P42285	Exosome RNA helicase MTR4 OS=Homo sapiens OX=9606 GN=MTREX PE=1 SV=3	1,290322581	0,919	0,732
P57105	Synaptojanin-2-binding protein OS=Homo sapiens OX=9606 GN=SYNJ2BP PE=1 SV=2	0,699790063	0,919	1,207
P00387	NADH-cytochrome b5 reductase 3 OS=Homo sapiens OX=9606 GN=CYB5R3 PE=1 SV=3	0,43956044	0,919	2,423
Q7Z3B4	Nucleoporin p54 OS=Homo sapiens OX=9606 GN=NUP54 PE=1 SV=2	0,814995925	0,917	1,011
Q9BR76	Coronin-1B OS=Homo sapiens OX=9606 GN=CORO1B PE=1 SV=1	0,948766603	0,915	0,748
Q15907	Ras-related protein Rab-11B OS=Homo sapiens OX=9606 GN=RAB11B PE=1 SV=4	0,838222967	0,915	0,97
P33992	DNA replication licensing factor MCM5 OS=Homo sapiens OX=9606 GN=MCM5 PE=1 SV=5	1,831501832	0,914	0,517
Q06787	Fragile X messenger ribonucleoprotein 1 OS=Homo sapiens OX=9606 GN=FMR1 PE=1 SV=1	0,877963126	0,913	1,002
Q9H0A0	RNA cytidine acetyltransferase OS=Homo sapiens OX=9606 GN=NAT10 PE=1 SV=2	1,972386588	0,911	0,463
O94906	Pre-mRNA-processing factor 6 OS=Homo sapiens OX=9606 GN=PRPF6 PE=1 SV=1	1,302083333	0,911	0,722
Q8WUM0	Nuclear pore complex protein Nup133 OS=Homo sapiens OX=9606 GN=NUP133 PE=1 SV=2	1,203369434	0,91	0,815
Q9Y394	Dehydrogenase/reductase SDR family member 7 OS=Homo sapiens OX=9606 GN=DHRS7 PE=1 SV=1	0,369139904	0,91	2,657
Q13724	Mannosyl-oligosaccharide glucosidase OS=Homo sapiens OX=9606 GN=MOGS PE=1 SV=5	0,882612533	0,909	0,989
P16435	NADPH--cytochrome P450 reductase OS=Homo sapiens OX=9606 GN=POR PE=1 SV=2	0,560538117	0,909	1,533
P04075	Fructose-bisphosphate aldolase A OS=Homo sapiens OX=9606 GN=ALDOA PE=1 SV=2	0,54200542	0,906	1,625
Q92544	Transmembrane 9 superfamily member 4 OS=Homo sapiens OX=9606 GN=TM9SF4 PE=1 SV=2	0,680272109	0,904	1,265
P42704	Leucine-rich PPR motif-containing protein, mitochondrial OS=Homo sapiens OX=9606 GN=LRPPRC PE=1 SV=3	1,349527665	0,901	0,637
P14866	Heterogeneous nuclear ribonucleoprotein L OS=Homo sapiens OX=9606 GN=HNRNPL PE=1 SV=2	1,030927835	0,901	0,837
Q9UKF6	Cleavage and polyadenylation specificity factor subunit 3 OS=Homo sapiens OX=9606 GN=CPSF3 PE=1 SV=1	1,592356688	0,899	0,533
Q99988	Growth/differentiation factor 15 OS=Homo sapiens OX=9606 GN=GDF15 PE=1 SV=3	1000	0,897	0,001
Q9H061	Transmembrane protein 126A OS=Homo sapiens OX=9606 GN=TMEM126A PE=1 SV=1	0,716845878	0,897	1,131
O95831	Apoptosis-inducing factor 1, mitochondrial OS=Homo sapiens OX=9606 GN=AIFM1 PE=1 SV=1	0,79491256	0,895	1,048
Q92896	Golgi apparatus protein 1 OS=Homo sapiens OX=9606 GN=GLG1 PE=1 SV=2	0,53390283	0,893	1,796
Q15637	Splicing factor 1 OS=Homo sapiens OX=9606 GN=SF1 PE=1 SV=4	1,329787234	0,892	0,761
Q12769	Nuclear pore complex protein Nup160 OS=Homo sapiens OX=9606 GN=NUP160 PE=1 SV=3	1,148105626	0,891	0,712
Q9NVP1	ATP-dependent RNA helicase DDX18 OS=Homo sapiens OX=9606 GN=DDX18 PE=1 SV=2	3,378378378	0,89	0,275
P62805	Histone H4 OS=Homo sapiens OX=9606 GN=H4C1 PE=1 SV=2	1,47275405	0,89	0,608
Q12788	Transducin beta-like protein 3 OS=Homo sapiens OX=9606 GN=TBL3 PE=1 SV=2	1,124859393	0,89	0,688
Q12907	Vesicular integral-membrane protein VIP36 OS=Homo sapiens OX=9606 GN=LMAN2 PE=1 SV=1	0,610873549	0,888	1,416
Q16698	2,4-dienoyl-CoA reductase [(3E)-enoyl-CoA-producing], mitochondrial OS=Homo sapiens OX=9606 GN=DECR1 PE=1 SV=1	0,561167228	0,887	1,651
Q16706	Alpha-mannosidase 2 OS=Homo sapiens OX=9606 GN=MAN2A1 PE=1 SV=2	0,253292806	0,887	3,625
O00764	Pyridoxal kinase OS=Homo sapiens OX=9606 GN=PDXK PE=1 SV=1	0,486618005	0,886	2,097
A0AAG2UVC5	Collagen type III alpha 1 chain OS=Homo sapiens OX=9606 GN=COL3A1 PE=1 SV=1	0,103337811	0,884	15,686
P07996	Thrombospondin-1 OS=Homo sapiens OX=9606 GN=THBS1 PE=1 SV=2	0,166278683	0,883	5,9
P46779	Large ribosomal subunit protein eL28 OS=Homo sapiens OX=9606 GN=RPL28 PE=1 SV=3	1,329787234	0,881	0,619
P50416	Carnitine O-palmitoyltransferase 1, liver isoform OS=Homo sapiens OX=9606 GN=CPT1A PE=1 SV=2	0,160771704	0,881	4,582
Q8ND56	Protein LSM14 homolog A OS=Homo sapiens OX=9606 GN=LSM14A PE=1 SV=3	1,560062402	0,88	0,61
P07203	Glutathione peroxidase 1 OS=Homo sapiens OX=9606 GN=GPX1 PE=1 SV=4	0,415973378	0,879	1,876
Q15758	Neutral amino acid transporter B(0) OS=Homo sapiens OX=9606 GN=SLC1A5 PE=1 SV=2	0,139275766	0,879	6,469
Q9UKD2	mRNA turnover protein 4 homolog OS=Homo sapiens OX=9606 GN=MRTO4 PE=1 SV=2	1,968503937	0,877	0,418
O43681	ATPase GET3 OS=Homo sapiens OX=9606 GN=GET3 PE=1 SV=2	0,874125874	0,875	0,986
Q9Y230	RuvB-like 2 OS=Homo sapiens OX=9606 GN=RUVBL2 PE=1 SV=3	1,216545012	0,874	0,661
O14979	Heterogeneous nuclear ribonucleoprotein D-like OS=Homo sapiens OX=9606 GN=HNRNPDL PE=1 SV=3	0,884173298	0,874	1,03
Q9BVK6	Transmembrane emp24 domain-containing protein 9 OS=Homo sapiens OX=9606 GN=TMED9 PE=1 SV=2	0,677506775	0,873	1,134
P16401	Histone H1.5 OS=Homo sapiens OX=9606 GN=H1-5 PE=1 SV=3	3,300330033	0,872	0,273

Q12905	Interleukin enhancer-binding factor 2 OS=Homo sapiens OX=9606 GN=ILF2 PE=1 SV=2	1,097694841	0,87	0,794
P62491	Ras-related protein Rab-11A OS=Homo sapiens OX=9606 GN=RAB11A PE=1 SV=3	0,978473581	0,87	0,872
O75083	WD repeat-containing protein 1 OS=Homo sapiens OX=9606 GN=WDR1 PE=1 SV=4	0,61500615	0,87	1,326
P41212	Transcription factor ETV6 OS=Homo sapiens OX=9606 GN=ETV6 PE=1 SV=1	0,601684717	0,87	1,449
P08123	Collagen alpha-2(I) chain OS=Homo sapiens OX=9606 GN=COL1A2 PE=1 SV=7	0,098667982	0,869	12,42
Q9UBM7	7-dehydrocholesterol reductase OS=Homo sapiens OX=9606 GN=DHCR7 PE=1 SV=1	5,524861878	0,867	0,173
P30876	DNA-directed RNA polymerase II subunit RPB2 OS=Homo sapiens OX=9606 GN=POLR2B PE=1 SV=1	1,293661061	0,865	0,773
Q9UHK6	Alpha-methylacyl-CoA racemase OS=Homo sapiens OX=9606 GN=AMACR PE=1 SV=2	0,669344043	0,865	4,627
Q8TEM1	Nuclear pore membrane glycoprotein 210 OS=Homo sapiens OX=9606 GN=NUP210 PE=1 SV=3	14,70588235	0,863	0,05
Q96CS3	FAS-associated factor 2 OS=Homo sapiens OX=9606 GN=FAF2 PE=1 SV=2	0,836120401	0,863	0,905
P04040	Catalase OS=Homo sapiens OX=9606 GN=CAT PE=1 SV=3	0,380083618	0,863	2,419
Q12797	Aspartyl/asparaginyl beta-hydroxylase OS=Homo sapiens OX=9606 GN=ASPH PE=1 SV=3	0,3003003	0,863	2,92
P61313	Large ribosomal subunit protein eL15 OS=Homo sapiens OX=9606 GN=RPL15 PE=1 SV=2	1,739130435	0,862	0,306
Q9NR30	Nucleolar RNA helicase 2 OS=Homo sapiens OX=9606 GN=DDX21 PE=1 SV=5	1,692047377	0,862	0,503
P61160	Actin-related protein 2 OS=Homo sapiens OX=9606 GN=ACTR2 PE=1 SV=1	0,64808814	0,862	1,316
O95470	Sphingosine-1-phosphate lyase 1 OS=Homo sapiens OX=9606 GN=SGPL1 PE=1 SV=3	1,011122346	0,861	0,875
P36551	Oxygen-dependent coproporphyrinogen-III oxidase, mitochondrial OS=Homo sapiens OX=9606 GN=CPOX PE=1 SV=3	0,09936407	0,861	8,163
Q13435	Splicing factor 3B subunit 2 OS=Homo sapiens OX=9606 GN=SF3B2 PE=1 SV=2	1,964636542	0,86	0,51
Q9UKX7	Nuclear pore complex protein Nup50 OS=Homo sapiens OX=9606 GN=NUP50 PE=1 SV=2	1,212121212	0,86	0,843
Q8NHH9	Atlastin-2 OS=Homo sapiens OX=9606 GN=ATL2 PE=1 SV=2	1,529051988	0,859	0,603
P51148	Ras-related protein Rab-5C OS=Homo sapiens OX=9606 GN=RAB5C PE=1 SV=2	0,618046972	0,859	1,334
Q9GZT3	SRA stem-loop-interacting RNA-binding protein, mitochondrial OS=Homo sapiens OX=9606 GN=SLIRP PE=1 SV=1	2,597402597	0,857	0,369
P05198	Eukaryotic translation initiation factor 2 subunit 1 OS=Homo sapiens OX=9606 GN=EIF2S1 PE=1 SV=3	1,222493888	0,856	0,623
Q53GQ0	Very-long-chain 3-oxoacyl-CoA reductase OS=Homo sapiens OX=9606 GN=HSD17B12 PE=1 SV=2	0,647249191	0,856	1,001
Q9BW27	Nuclear pore complex protein Nup85 OS=Homo sapiens OX=9606 GN=NUP85 PE=1 SV=1	1,047120419	0,855	0,832
P06748	Nucleophosmin OS=Homo sapiens OX=9606 GN=NPM1 PE=1 SV=2	2,69541779	0,854	0,302
P39748	Flap endonuclease 1 OS=Homo sapiens OX=9606 GN=FEN1 PE=1 SV=1	2,538071066	0,853	0,319
O43290	U4/U6.U5 tri-snRNP-associated protein 1 OS=Homo sapiens OX=9606 GN=SART1 PE=1 SV=1	1,485884101	0,853	0,609
Q8WUM4	Programmed cell death 6-interacting protein OS=Homo sapiens OX=9606 GN=PDCD6IP PE=1 SV=1	0,701262272	0,853	1,272
P26368	Splicing factor U2AF 65 kDa subunit OS=Homo sapiens OX=9606 GN=U2AF2 PE=1 SV=4	1,663893511	0,851	0,493
Q14966	Zinc finger protein 638 OS=Homo sapiens OX=9606 GN=ZNF638 PE=1 SV=2	2,680965147	0,85	0,409
Q9UG63	ATP-binding cassette sub-family F member 2 OS=Homo sapiens OX=9606 GN=ABCF2 PE=1 SV=2	1,152073733	0,849	0,684
Q9UHG3	Prenylcysteine oxidase 1 OS=Homo sapiens OX=9606 GN=PCYOX1 PE=1 SV=3	1,133786848	0,849	0,784
Q99873	Protein arginine N-methyltransferase 1 OS=Homo sapiens OX=9606 GN=PRMT1 PE=1 SV=3	1,564945227	0,848	0,513
Q96T51	RUN and FYVE domain-containing protein 1 OS=Homo sapiens OX=9606 GN=RUFY1 PE=1 SV=2	1,303780965	0,848	0,65
Q8WWY3	U4/U6 small nuclear ribonucleoprotein Prp31 OS=Homo sapiens OX=9606 GN=PRPF31 PE=1 SV=2	0,991080278	0,848	0,917
P57740	Nuclear pore complex protein Nup107 OS=Homo sapiens OX=9606 GN=NUP107 PE=1 SV=1	1,204819277	0,847	0,781
Q9Y2A7	Nck-associated protein 1 OS=Homo sapiens OX=9606 GN=NCKAP1 PE=1 SV=1	1,025641026	0,846	0,794
P45880	Voltage-dependent anion-selective channel protein 2 OS=Homo sapiens OX=9606 GN=VDAC2 PE=1 SV=2	0,750187547	0,845	0,973
P18031	Tyrosine-protein phosphatase non-receptor type 1 OS=Homo sapiens OX=9606 GN=PTPN1 PE=1 SV=1	0,648929267	0,844	1,402
P40926	Malate dehydrogenase, mitochondrial OS=Homo sapiens OX=9606 GN=MDH2 PE=1 SV=3	0,947867299	0,843	0,845
P09661	U2 small nuclear ribonucleoprotein A' OS=Homo sapiens OX=9606 GN=SNRPA1 PE=1 SV=2	1,095290252	0,842	0,796
Q96KP4	Cytosolic non-specific dipeptidase OS=Homo sapiens OX=9606 GN=CNDP2 PE=1 SV=2	1,709401709	0,841	0,471
P00338	L-lactate dehydrogenase A chain OS=Homo sapiens OX=9606 GN=LDHA PE=1 SV=2	0,92936803	0,841	0,955
P06744	Glucose-6-phosphate isomerase OS=Homo sapiens OX=9606 GN=GPI PE=1 SV=4	0,736377025	0,841	1,169
P08574	Cytochrome c1, heme protein, mitochondrial OS=Homo sapiens OX=9606 GN=CYC1 PE=1 SV=3	1,004016064	0,84	0,724
O43615	Mitochondrial import inner membrane translocase subunit TIM44 OS=Homo sapiens OX=9606 GN=TIMM44 PE=1 SV=2	0,82781457	0,84	0,989
O00116	Alkyldihydroxyacetonephosphate synthase, peroxisomal OS=Homo sapiens OX=9606 GN=AGPS PE=1 SV=1	0,754716981	0,839	1,123
Q5JWF2	Guanine nucleotide-binding protein G(s) subunit alpha isoforms XLas OS=Homo sapiens OX=9606 GN=GNAS PE=1 SV=2	0,771010023	0,836	1,025
P0DMV9	Heat shock 70 kDa protein 1B OS=Homo sapiens OX=9606 GN=HSPA1B PE=1 SV=1	0,635727908	0,836	1,291
P63173	Large ribosomal subunit protein eL38 OS=Homo sapiens OX=9606 GN=RPL38 PE=1 SV=2	1,126126126	0,835	0,826
Q15397	Pumilio homolog 3 OS=Homo sapiens OX=9606 GN=PUM3 PE=1 SV=3	0,762195122	0,835	0,966
Q86UP2	Kinectin OS=Homo sapiens OX=9606 GN=KTN1 PE=1 SV=1	0,524109015	0,834	1,599
Q9P0J0	NADH dehydrogenase [ubiquinone] 1 alpha subcomplex subunit 13 OS=Homo sapiens OX=9606 GN=NDUFA13 PE=1 SV=3	0,64061499	0,833	1,264
P46060	Ran GTPase-activating protein 1 OS=Homo sapiens OX=9606 GN=RANGAP1 PE=1 SV=1	1,333333333	0,832	0,574
Q9UHB9	Signal recognition particle subunit SRP68 OS=Homo sapiens OX=9606 GN=SRP68 PE=1 SV=2	1,149425287	0,832	0,582
Q13011	Delta(3,5)-Delta(2,4)-dienoyl-CoA isomerase, mitochondrial OS=Homo sapiens OX=9606 GN=ECH1 PE=1 SV=2	0,53561864	0,831	1,458
Q70UQ0	Inhibitor of nuclear factor kappa-B kinase-interacting protein OS=Homo sapiens OX=9606 GN=IKBIP PE=1 SV=1	0,232774674	0,831	3,616
O95602	DNA-directed RNA polymerase I subunit RPA1 OS=Homo sapiens OX=9606 GN=POLR1A PE=1 SV=2	1,633986928	0,83	0,654
Q6IBS0	Twinfilin-2 OS=Homo sapiens OX=9606 GN=TWF2 PE=1 SV=2	0,672043011	0,83	1,21
Q96PK6	RNA-binding protein 14 OS=Homo sapiens OX=9606 GN=RBM14 PE=1 SV=2	1,331557923	0,829	0,578
Q9NVJ2	ADP-ribosylation factor-like protein 8B OS=Homo sapiens OX=9606 GN=ARL8B PE=1 SV=1	0,652315721	0,827	1,324
Q9H3P7	Golgi resident protein GCP60 OS=Homo sapiens OX=9606 GN=ACBD3 PE=1 SV=4	0,573394495	0,827	1,543
Q9UJU6	Drebrin-like protein OS=Homo sapiens OX=9606 GN=DBNL PE=1 SV=1	0,854700855	0,825	0,786
P04899	Guanine nucleotide-binding protein G(i) subunit alpha-2 OS=Homo sapiens OX=9606 GN=GNAI2 PE=1 SV=3	0,568828214	0,825	1,669
Q8IVF2	Protein AHNAK2 OS=Homo sapiens OX=9606 GN=AHNAK2 PE=1 SV=2	0,082209799	0,825	10,633
Q96A26	Protein FAM162A OS=Homo sapiens OX=9606 GN=FAM162A PE=1 SV=2	1,098901099	0,824	0,966
Q9UN86	Ras GTPase-activating protein-binding protein 2 OS=Homo sapiens OX=9606 GN=G3BP2 PE=1 SV=2	1,468428781	0,822	0,605
Q96A33	PAT complex subunit CCDC47 OS=Homo sapiens OX=9606 GN=CCDC47 PE=1 SV=1	1,008064516	0,821	0,698
Q8WVM8	Sec1 family domain-containing protein 1 OS=Homo sapiens OX=9606 GN=SCFD1 PE=1 SV=4	0,632511069	0,821	1,004
A3KMH1	von Willebrand factor A domain-containing protein 8 OS=Homo sapiens OX=9606 GN=VWA8 PE=1 SV=2	1,200480192	0,82	0,676
Q8N6T3	ADP-ribosylation factor GTPase-activating protein 1 OS=Homo sapiens OX=9606 GN=ARFGAP1 PE=1 SV=2	0,812347685	0,818	1,031
P28288	ATP-binding cassette sub-family D member 3 OS=Homo sapiens OX=9606 GN=ABCD3 PE=1 SV=1	0,824402308	0,817	0,893
Q9BUL8	Programmed cell death protein 10 OS=Homo sapiens OX=9606 GN=PDCD10 PE=1 SV=1	1,272264631	0,816	0,915
Q9Y277	Voltage-dependent anion-selective channel protein 3 OS=Homo sapiens OX=9606 GN=VDAC3 PE=1 SV=1	1,17370892	0,814	0,951
P07954	Fumarate hydratase, mitochondrial OS=Homo sapiens OX=9606 GN=FH PE=1 SV=3	0,658761528	0,813	1,195
Q09161	Nuclear cap-binding protein subunit 1 OS=Homo sapiens OX=9606 GN=NCBP1 PE=1 SV=1	1,089324619	0,811	0,678
P37198	Nuclear pore glycoprotein p62 OS=Homo sapiens OX=9606 GN=NUP62 PE=1 SV=3	0,921658986	0,809	0,867
P05026	Sodium/potassium-transporting ATPase subunit beta-1 OS=Homo sapiens OX=9606 GN=ATP1B1 PE=1 SV=1	0,306748466	0,809	2,364
P00568	Adenylate kinase isoenzyme 1 OS=Homo sapiens OX=9606 GN=AK1 PE=1 SV=3	0,588581519	0,805	1,449
P61421	V-type proton ATPase subunit d 1 OS=Homo sapiens OX=9606 GN=ATP6V0D1 PE=1 SV=1	0,571102227	0,805	1,556
Q12906	Interleukin enhancer-binding factor 3 OS=Homo sapiens OX=9606 GN=ILF3 PE=1 SV=3	1,338688086	0,804	0,62
Q13123	Protein Red OS=Homo sapiens OX=9606 GN=IK PE=1 SV=3	1,757469244	0,803	0,378

Q86U38	Nucleolar protein 9 OS=Homo sapiens OX=9606 GN=NOP9 PE=1 SV=1	1,814882033	0,802	0,46
P0DP25	Calmodulin-3 OS=Homo sapiens OX=9606 GN=CALM3 PE=1 SV=1	1,445086705	0,801	0,618
Q14694	Ubiquitin carboxyl-terminal hydrolase 10 OS=Homo sapiens OX=9606 GN=USP10 PE=1 SV=2	1,420454545	0,8	0,582
Q8TDN6	Ribosome biogenesis protein BRX1 homolog OS=Homo sapiens OX=9606 GN=BRIX1 PE=1 SV=2	2,421307506	0,799	0,588
Q9Y6C9	Mitochondrial carrier homolog 2 OS=Homo sapiens OX=9606 GN=MTCH2 PE=1 SV=1	0,592768228	0,799	1,395
Q92979	Ribosomal RNA small subunit methyltransferase NEP1 OS=Homo sapiens OX=9606 GN=EMG1 PE=1 SV=4	1,233045623	0,798	0,624
O14786	Neuropilin-1 OS=Homo sapiens OX=9606 GN=NRP1 PE=1 SV=3	0,068259386	0,796	12,338
P50897	Palmitoyl-protein thioesterase 1 OS=Homo sapiens OX=9606 GN=PPT1 PE=1 SV=1	1,300390117	0,795	0,572
Q00059	Transcription factor A, mitochondrial OS=Homo sapiens OX=9606 GN=TFAM PE=1 SV=1	0,887311446	0,793	0,734
P36776	Lon protease homolog, mitochondrial OS=Homo sapiens OX=9606 GN=LONP1 PE=1 SV=2	0,757575758	0,792	1,051
Q96SQ9	Cytochrome P450 2S1 OS=Homo sapiens OX=9606 GN=CYP2S1 PE=1 SV=2	0,456412597	0,792	1,327
P54727	UV excision repair protein RAD23 homolog B OS=Homo sapiens OX=9606 GN=RAD23B PE=1 SV=1	0,987166831	0,791	0,828
O15042	U2 snRNP-associated SURP motif-containing protein OS=Homo sapiens OX=9606 GN=U2SURP PE=1 SV=2	1,428571429	0,79	0,519
P49792	E3 SUMO-protein ligase RanBP2 OS=Homo sapiens OX=9606 GN=RANBP2 PE=1 SV=2	1,386962552	0,79	0,592
Q14690	Protein RRP5 homolog OS=Homo sapiens OX=9606 GN=PDCD11 PE=1 SV=3	2,762430939	0,788	0,32
P36542	ATP synthase subunit gamma, mitochondrial OS=Homo sapiens OX=9606 GN=ATP5F1C PE=1 SV=1	0,855431993	0,788	0,875
Q9NTJ5	Phosphatidylinositol-3-phosphatase SAC1 OS=Homo sapiens OX=9606 GN=SACM1L PE=1 SV=2	0,834724541	0,788	0,875
Q8NBX0	Saccharopine dehydrogenase-like oxidoreductase OS=Homo sapiens OX=9606 GN=SCCPDH PE=1 SV=1	0,466635558	0,788	1,881
P31040	Succinate dehydrogenase [ubiquinone] flavoprotein subunit, mitochondrial OS=Homo sapiens OX=9606 GN=SDHA PE=1 SV=	0,596658711	0,787	0,991
Q5SSJ5	Heterochromatin protein 1-binding protein 3 OS=Homo sapiens OX=9606 GN=HP1BP3 PE=1 SV=1	1,023541453	0,786	0,713
Q01105	Protein SET OS=Homo sapiens OX=9606 GN=SET PE=1 SV=3	1,886792453	0,784	0,428
Q9H3Z4	DnaJ homolog subfamily C member 5 OS=Homo sapiens OX=9606 GN=DNAJC5 PE=1 SV=1	0,704225352	0,784	0,889
P10644	cAMP-dependent protein kinase type I-alpha regulatory subunit OS=Homo sapiens OX=9606 GN=PRKAR1A PE=1 SV=1	0,487329435	0,784	1,724
Q13045	Protein flightless-1 homolog OS=Homo sapiens OX=9606 GN=FLII PE=1 SV=2	0,758725341	0,783	1,013
Q16795	NADH dehydrogenase [ubiquinone] 1 alpha subcomplex subunit 9, mitochondrial OS=Homo sapiens OX=9606 GN=NDUFA9	0,744047619	0,783	1,131
Q99714	3-hydroxyacyl-CoA dehydrogenase type-2 OS=Homo sapiens OX=9606 GN=HSD17B10 PE=1 SV=3	0,672494956	0,783	1,154
Q16363	Laminin subunit alpha-4 OS=Homo sapiens OX=9606 GN=LAMA4 PE=1 SV=4	0,125140783	0,783	0,448
P49756	RNA-binding protein 25 OS=Homo sapiens OX=9606 GN=RBM25 PE=1 SV=3	1,394700139	0,782	0,518
Q6P1J9	Parafibromin OS=Homo sapiens OX=9606 GN=CDC73 PE=1 SV=1	0,973709834	0,782	0,809
P0DP23	Calmodulin-1 OS=Homo sapiens OX=9606 GN=CALM1 PE=1 SV=1	1,526717557	0,78	0,585
P22626	Heterogeneous nuclear ribonucleoproteins A2/B1 OS=Homo sapiens OX=9606 GN=HNRNPA2B1 PE=1 SV=2	1,022494888	0,778	0,651
O43747	AP-1 complex subunit gamma-1 OS=Homo sapiens OX=9606 GN=AP1G1 PE=1 SV=5	0,547945205	0,776	1,122
O60884	DnaJ homolog subfamily A member 2 OS=Homo sapiens OX=9606 GN=DNAJA2 PE=1 SV=1	1,677852349	0,775	0,717
O94776	Metastasis-associated protein MTA2 OS=Homo sapiens OX=9606 GN=MTA2 PE=1 SV=1	1,020408163	0,771	0,736
P06576	ATP synthase subunit beta, mitochondrial OS=Homo sapiens OX=9606 GN=ATP5F1B PE=1 SV=3	0,730994152	0,77	1,059
Q9BUJ2	Heterogeneous nuclear ribonucleoprotein U-like protein 1 OS=Homo sapiens OX=9606 GN=HNRNPUL1 PE=1 SV=2	0,727802038	0,77	1,132
Q9H6E4	Coiled-coil domain-containing protein 134 OS=Homo sapiens OX=9606 GN=CCDC134 PE=1 SV=1	2,298850575	0,769	0,441
P29317	Ephrin type-A receptor 2 OS=Homo sapiens OX=9606 GN=EPHA2 PE=1 SV=2	0,42662116	0,769	1,833
P61964	WD repeat-containing protein 5 OS=Homo sapiens OX=9606 GN=WDR5 PE=1 SV=1	1,083423619	0,768	0,747
Q96AG4	Leucine-rich repeat-containing protein 59 OS=Homo sapiens OX=9606 GN=LRRC59 PE=1 SV=1	0,654022237	0,768	1,043
O75306	NADH dehydrogenase [ubiquinone] iron-sulfur protein 2, mitochondrial OS=Homo sapiens OX=9606 GN=NDUFS2 PE=1 SV=2	0,516262261	0,768	1,302
O43172	U4/U6 small nuclear ribonucleoprotein Prp4 OS=Homo sapiens OX=9606 GN=PRPF4 PE=1 SV=2	0,81300813	0,766	0,96
Q03252	Lamin-B2 OS=Homo sapiens OX=9606 GN=LMNB2 PE=1 SV=4	0,660938533	0,763	1,136
Q8NI36	WD repeat-containing protein 36 OS=Homo sapiens OX=9606 GN=WDR36 PE=1 SV=1	1,582278481	0,762	0,431
Q9H9B4	Sideroflexin-1 OS=Homo sapiens OX=9606 GN=SFXN1 PE=1 SV=4	1,597444089	0,759	0,517
P61006	Ras-related protein Rab-8A OS=Homo sapiens OX=9606 GN=RAB8A PE=1 SV=1	0,778816199	0,759	0,804
P24844	Myosin regulatory light polypeptide 9 OS=Homo sapiens OX=9606 GN=MYL9 PE=1 SV=4	0,419287212	0,759	2,025
Q9BZJ0	Crooked neck-like protein 1 OS=Homo sapiens OX=9606 GN=CRNKL1 PE=1 SV=4	1,785714286	0,758	0,43
P52597	Heterogeneous nuclear ribonucleoprotein F OS=Homo sapiens OX=9606 GN=HNRNPF PE=1 SV=3	1,075268817	0,758	1,219
Q96TA2	ATP-dependent zinc metalloprotease YME1L1 OS=Homo sapiens OX=9606 GN=YME1L1 PE=1 SV=2	1,08577633	0,757	0,735
P11388	DNA topoisomerase 2-alpha OS=Homo sapiens OX=9606 GN=TOP2A PE=1 SV=3	3,154574132	0,756	0,263
Q92888	Rho guanine nucleotide exchange factor 1 OS=Homo sapiens OX=9606 GN=ARHGEF1 PE=1 SV=2	0,666222518	0,755	1,035
P05023	Sodium/potassium-transporting ATPase subunit alpha-1 OS=Homo sapiens OX=9606 GN=ATP1A1 PE=1 SV=1	0,662690524	0,755	1,048
Q9Y3L5	Ras-related protein Rap-2c OS=Homo sapiens OX=9606 GN=RAP2C PE=1 SV=1	0,946969697	0,754	0,717
Q92599	Septin-8 OS=Homo sapiens OX=9606 GN=SEPTIN8 PE=1 SV=4	0,652741514	0,753	1,035
Q8TEQ6	Gem-associated protein 5 OS=Homo sapiens OX=9606 GN=GEMIN5 PE=1 SV=3	3,003003003	0,752	0,273
O15144	Actin-related protein 2/3 complex subunit 2 OS=Homo sapiens OX=9606 GN=ARPC2 PE=1 SV=1	0,636537237	0,752	1,195
Q13492	Phosphatidylinositol-binding clathrin assembly protein OS=Homo sapiens OX=9606 GN=PICALM PE=1 SV=2	0,374391614	0,752	2,161
P20042	Eukaryotic translation initiation factor 2 subunit 2 OS=Homo sapiens OX=9606 GN=EIF2S2 PE=1 SV=2	1,175088132	0,749	0,676
Q6NUK1	Mitochondrial adenyl nucleotide antiporter SLC25A24 OS=Homo sapiens OX=9606 GN=SLC25A24 PE=1 SV=2	0,725163162	0,748	1,214
O75691	Small subunit processome component 20 homolog OS=Homo sapiens OX=9606 GN=UTP20 PE=1 SV=3	1,545595054	0,747	0,476
P53007	Tricarboxylate transport protein, mitochondrial OS=Homo sapiens OX=9606 GN=SLC25A1 PE=1 SV=2	1,416430595	0,747	0,37
Q99614	Tetratricopeptide repeat protein 1 OS=Homo sapiens OX=9606 GN=TTC1 PE=1 SV=1	1,477104874	0,745	0,485
Q8WUF5	RelA-associated inhibitor OS=Homo sapiens OX=9606 GN=PPP1R13L PE=1 SV=4	0,58411215	0,744	0,701
Q8IYB3	Serine/arginine repetitive matrix protein 1 OS=Homo sapiens OX=9606 GN=SRRM1 PE=1 SV=2	2,288329519	0,743	0,23
P54920	Alpha-soluble NSF attachment protein OS=Homo sapiens OX=9606 GN=NAPA PE=1 SV=3	0,562746201	0,743	1,281
P19367	Hexokinase-1 OS=Homo sapiens OX=9606 GN=HK1 PE=1 SV=3	0,378787879	0,743	2,08
P38646	Stress-70 protein, mitochondrial OS=Homo sapiens OX=9606 GN=HSPA9 PE=1 SV=2	0,898472597	0,742	0,858
Q07021	Complement component 1 Q subcomponent-binding protein, mitochondrial OS=Homo sapiens OX=9606 GN=C1QBP PE=1 S	1,615508885	0,74	0,433
Q13243	Serine/arginine-rich splicing factor 5 OS=Homo sapiens OX=9606 GN=SRSF5 PE=1 SV=1	2,024291498	0,739	0,348
Q6IAA8	Ragulator complex protein LAMTOR1 OS=Homo sapiens OX=9606 GN=LAMTOR1 PE=1 SV=2	0,800640512	0,739	0,936
Q10713	Mitochondrial-processing peptidase subunit alpha OS=Homo sapiens OX=9606 GN=PMPCA PE=1 SV=2	1,133786848	0,737	0,599
P82673	Small ribosomal subunit protein mS35 OS=Homo sapiens OX=9606 GN=MRPS35 PE=1 SV=1	0,942507069	0,737	0,779
Q92621	Nuclear pore complex protein Nup205 OS=Homo sapiens OX=9606 GN=NUP205 PE=1 SV=3	1,096491228	0,736	0,658
O75165	DnaJ homolog subfamily C member 13 OS=Homo sapiens OX=9606 GN=DNAJC13 PE=1 SV=5	0,738552437	0,734	0,983
Q6YP21	Kynurenine--oxoglutarate transaminase 3 OS=Homo sapiens OX=9606 GN=KYAT3 PE=1 SV=1	1,308900524	0,731	0,559
Q7Z417	FMR1-interacting protein NUFIP2 OS=Homo sapiens OX=9606 GN=NUFIP2 PE=1 SV=1	1,199040767	0,731	0,623
P48047	ATP synthase subunit O, mitochondrial OS=Homo sapiens OX=9606 GN=ATP5PO PE=1 SV=1	1,282051282	0,73	0,484
P82933	Small ribosomal subunit protein uS9m OS=Homo sapiens OX=9606 GN=MRPS9 PE=1 SV=2	1,100110011	0,73	0,478
Q9P0L0	Vesicle-associated membrane protein-associated protein A OS=Homo sapiens OX=9606 GN=VAPA PE=1 SV=3	0,556173526	0,729	1,033
P09012	U1 small nuclear ribonucleoprotein A OS=Homo sapiens OX=9606 GN=SNRPA PE=1 SV=3	1,269035533	0,728	0,625
Q9Y3C1	Nucleolar protein 16 OS=Homo sapiens OX=9606 GN=NOP16 PE=1 SV=2	1,19047619	0,727	0,619
Q8NFH4	Nucleoporin Nup37 OS=Homo sapiens OX=9606 GN=NUP37 PE=1 SV=1	1,161440186	0,726	0,674

Q01780	Exosome complex component 10 OS=Homo sapiens OX=9606 GN=EXOSC10 PE=1 SV=2	1,572327044	0,724	0,456
O60506	Heterogeneous nuclear ribonucleoprotein Q OS=Homo sapiens OX=9606 GN=SYNCRIP PE=1 SV=2	0,964320154	0,724	0,699
Q00325	Solute carrier family 25 member 3 OS=Homo sapiens OX=9606 GN=SLC25A3 PE=1 SV=2	1,018329939	0,722	0,568
Q8IXI2	Mitochondrial Rho GTPase 1 OS=Homo sapiens OX=9606 GN=RHOT1 PE=1 SV=2	1,100110011	0,721	0,621
Q8WUY1	Protein THEM6 OS=Homo sapiens OX=9606 GN=THEM6 PE=1 SV=2	1,034126163	0,72	0,504
P35658	Nuclear pore complex protein Nup214 OS=Homo sapiens OX=9606 GN=NUP214 PE=1 SV=2	1,349527665	0,718	0,662
P55265	Double-stranded RNA-specific adenosine deaminase OS=Homo sapiens OX=9606 GN=ADAR PE=1 SV=4	0,428632662	0,718	1,771
Q9BTV4	Transmembrane protein 43 OS=Homo sapiens OX=9606 GN=TMEM43 PE=1 SV=1	0,407996736	0,717	1,598
Q1KMD3	Heterogeneous nuclear ribonucleoprotein U-like protein 2 OS=Homo sapiens OX=9606 GN=HNRNPUL2 PE=1 SV=1	0,906618314	0,716	0,776
P80188	Neutrophil gelatinase-associated lipocalin OS=Homo sapiens OX=9606 GN=LCN2 PE=1 SV=2	0,384024578	0,716	1,864
Q6P1A2	Lysophospholipid acyltransferase 5 OS=Homo sapiens OX=9606 GN=LPCAT3 PE=1 SV=1	0,30892802	0,716	2,417
Q9H773	dCTP pyrophosphatase 1 OS=Homo sapiens OX=9606 GN=DCTPP1 PE=1 SV=1	1,184834123	0,715	0,487
O75494	Serine/arginine-rich splicing factor 10 OS=Homo sapiens OX=9606 GN=SRSF10 PE=1 SV=1	0,952380952	0,714	0,468
P37235	Hippocalcin-like protein 1 OS=Homo sapiens OX=9606 GN=HPCAL1 PE=1 SV=3	0,150738619	0,713	4,521
Q8NBJ7	Inactive C-alpha-formylglycine-generating enzyme 2 OS=Homo sapiens OX=9606 GN=SUMF2 PE=1 SV=2	0,461467467	0,712	1,547
O75400	Pre-mRNA-processing factor 40 homolog A OS=Homo sapiens OX=9606 GN=PRPF40A PE=1 SV=2	1,297016861	0,711	0,407
Q9UNX4	WD repeat-containing protein 3 OS=Homo sapiens OX=9606 GN=WDR3 PE=1 SV=1	1,904761905	0,71	0,462
Q8NCN5	Pyruvate dehydrogenase phosphatase regulatory subunit, mitochondrial OS=Homo sapiens OX=9606 GN=PDPR PE=1 SV=2	0,871080139	0,71	0,765
P02768	Albumin OS=Homo sapiens OX=9606 GN=ALB PE=1 SV=2	1,095290252	0,709	2,19
Q92797	Symplekin OS=Homo sapiens OX=9606 GN=SYMPK PE=1 SV=2	0,889679715	0,708	0,664
Q99567	Nuclear pore complex protein Nup88 OS=Homo sapiens OX=9606 GN=NUP88 PE=1 SV=2	1,180637544	0,707	0,622
P54709	Sodium/potassium-transporting ATPase subunit beta-3 OS=Homo sapiens OX=9606 GN=ATP1B3 PE=1 SV=1	1,095290252	0,707	0,618
Q04837	Single-stranded DNA-binding protein, mitochondrial OS=Homo sapiens OX=9606 GN=SSBP1 PE=1 SV=1	0,758150114	0,706	0,888
Q92747	Actin-related protein 2/3 complex subunit 1A OS=Homo sapiens OX=9606 GN=ARPC1A PE=1 SV=2	2,164502165	0,705	0,319
Q9H7B2	Ribosome production factor 2 homolog OS=Homo sapiens OX=9606 GN=RPF2 PE=1 SV=2	1,683501684	0,705	0,434
O95336	6-phosphogluconolactonase OS=Homo sapiens OX=9606 GN=PGLS PE=1 SV=2	0,939849624	0,705	0,741
Q9P253	Vacuolar protein sorting-associated protein 18 homolog OS=Homo sapiens OX=9606 GN=VPS18 PE=1 SV=2	0,726744186	0,702	1,339
P22087	rRNA 2'-O-methyltransferase fibrillarin OS=Homo sapiens OX=9606 GN=FBL PE=1 SV=2	1,126126126	0,701	0,45
Q9NP72	Ras-related protein Rab-18 OS=Homo sapiens OX=9606 GN=RAB18 PE=1 SV=1	0,467071462	0,701	1,407
Q9UKV3	Apoptotic chromatin condensation inducer in the nucleus OS=Homo sapiens OX=9606 GN=ACIN1 PE=1 SV=2	1,766784452	0,699	0,387
Q15223	Nectin-1 OS=Homo sapiens OX=9606 GN=NECTIN1 PE=1 SV=3	4,166666667	0,695	0,252
Q92692	Nectin-2 OS=Homo sapiens OX=9606 GN=NECTIN2 PE=1 SV=1	0,40371417	0,695	1,59
O75947	ATP synthase subunit d, mitochondrial OS=Homo sapiens OX=9606 GN=ATP5PD PE=1 SV=3	0,785545954	0,694	0,748
Q9BVJ6	U3 small nucleolar RNA-associated protein 14 homolog A OS=Homo sapiens OX=9606 GN=UTP14A PE=1 SV=1	2	0,693	0,373
Q8WXH0	Nesprin-2 OS=Homo sapiens OX=9606 GN=SYNE2 PE=1 SV=3	1,455604076	0,693	0,544
O00754	Lysosomal alpha-mannosidase OS=Homo sapiens OX=9606 GN=MAN2B1 PE=1 SV=3	0,496277916	0,693	1,316
O00267	Transcription elongation factor SPT5 OS=Homo sapiens OX=9606 GN=SUPT5H PE=1 SV=1	1,383125864	0,692	0,525
Q29RF7	Sister chromatid cohesion protein PDS5 homolog A OS=Homo sapiens OX=9606 GN=PDS5A PE=1 SV=1	1,223990208	0,692	0,556
Q8IWT6	Volume-regulated anion channel subunit LRRC8A OS=Homo sapiens OX=9606 GN=LRRC8A PE=1 SV=1	0,928505107	0,692	0,826
P59998	Actin-related protein 2/3 complex subunit 4 OS=Homo sapiens OX=9606 GN=ARPC4 PE=1 SV=3	0,786782061	0,692	0,839
P53597	Succinate--CoA ligase [ADP/GDP-forming] subunit alpha, mitochondrial OS=Homo sapiens OX=9606 GN=SUCLG1 PE=1 SV=4	1,282051282	0,691	0,585
Q8N684	Cleavage and polyadenylation specificity factor subunit 7 OS=Homo sapiens OX=9606 GN=CPSF7 PE=1 SV=1	1,36239782	0,69	0,519
Q7L0Y3	tRNA methyltransferase 10 homolog C OS=Homo sapiens OX=9606 GN=TRMT10C PE=1 SV=2	1,107419712	0,69	0,582
O60313	Dynamin-like 120 kDa protein, mitochondrial OS=Homo sapiens OX=9606 GN=OPA1 PE=1 SV=3	1,269035533	0,689	0,516
Q6UN15	Pre-mRNA 3'-end-processing factor FIP1 OS=Homo sapiens OX=9606 GN=FIP1L1 PE=1 SV=1	1,222493888	0,689	0,621
Q12996	Cleavage stimulation factor subunit 3 OS=Homo sapiens OX=9606 GN=CSTF3 PE=1 SV=1	1,200480192	0,689	0,589
Q9NVH1	DnaJ homolog subfamily C member 11 OS=Homo sapiens OX=9606 GN=DNAJC11 PE=1 SV=2	1,18623962	0,688	0,644
Q99848	Probable rRNA-processing protein EBP2 OS=Homo sapiens OX=9606 GN=EBNA1BP2 PE=1 SV=2	1,628664495	0,686	0,435
Q99805	Transmembrane 9 superfamily member 2 OS=Homo sapiens OX=9606 GN=TM9SF2 PE=1 SV=1	0,504540868	0,686	0,763
Q92665	Small ribosomal subunit protein mS31 OS=Homo sapiens OX=9606 GN=MRPS31 PE=1 SV=3	1,207729469	0,684	0,547
Q9BTU6	Phosphatidylinositol 4-kinase type 2-alpha OS=Homo sapiens OX=9606 GN=PI4K2A PE=1 SV=1	0,757002271	0,684	0,722
Q9H583	HEAT repeat-containing protein 1 OS=Homo sapiens OX=9606 GN=HEATR1 PE=1 SV=3	1,818181818	0,682	0,371
P04792	Heat shock protein beta-1 OS=Homo sapiens OX=9606 GN=HSPB1 PE=1 SV=2	0,267594327	0,682	1,898
Q99653	Calcineurin B homologous protein 1 OS=Homo sapiens OX=9606 GN=CHP1 PE=1 SV=3	0,702247191	0,681	0,934
P52948	Nuclear pore complex protein Nup98-Nup96 OS=Homo sapiens OX=9606 GN=NUP98 PE=1 SV=4	1,107419712	0,68	0,636
Q9BYD3	Large ribosomal subunit protein uL4m OS=Homo sapiens OX=9606 GN=MRPL4 PE=1 SV=1	1,36425648	0,679	0,73
P57088	Transmembrane protein 33 OS=Homo sapiens OX=9606 GN=TMEM33 PE=1 SV=2	1,206272618	0,679	0,457
P02452	Collagen alpha-1(I) chain OS=Homo sapiens OX=9606 GN=COL1A1 PE=1 SV=6	0,04820671	0,679	12,747
A0AVT1	Ubiquitin-like modifier-activating enzyme 6 OS=Homo sapiens OX=9606 GN=UBA6 PE=1 SV=1	0,901713255	0,674	0,824
P05141	ADP/ATP translocase 2 OS=Homo sapiens OX=9606 GN=SLC25A5 PE=1 SV=7	1,225490196	0,672	0,489
Q5JTH9	RRP12-like protein OS=Homo sapiens OX=9606 GN=RRP12 PE=1 SV=2	1,550387597	0,668	0,426
O75915	PRA1 family protein 3 OS=Homo sapiens OX=9606 GN=ARL6IP5 PE=1 SV=1	0,41736227	0,668	1,483
P19404	NADH dehydrogenase [ubiquinone] flavoprotein 2, mitochondrial OS=Homo sapiens OX=9606 GN=NDUFV2 PE=1 SV=2	0,576701269	0,666	1,009
Q9NP81	Serine--tRNA ligase, mitochondrial OS=Homo sapiens OX=9606 GN=SARS2 PE=1 SV=1	1,464128843	0,664	0,371
O95292	Vesicle-associated membrane protein-associated protein B/C OS=Homo sapiens OX=9606 GN=VAPB PE=1 SV=3	0,963391137	0,66	0,67
Q9NUP9	Protein lin-7 homolog C OS=Homo sapiens OX=9606 GN=LIN7C PE=1 SV=1	0,48709206	0,66	1,596
O43491	Band 4.1-like protein 2 OS=Homo sapiens OX=9606 GN=EPB41L2 PE=1 SV=1	4,854368932	0,658	0,169
Q8N5K1	CDGSH iron-sulfur domain-containing protein 2 OS=Homo sapiens OX=9606 GN=CISD2 PE=1 SV=1	0,871080139	0,658	0,642
Q14914	Prostaglandin reductase 1 OS=Homo sapiens OX=9606 GN=PTGR1 PE=1 SV=2	0,365230095	0,658	1,826
O14950	Myosin regulatory light chain 12B OS=Homo sapiens OX=9606 GN=MYL12B PE=1 SV=2	0,337268128	0,657	2,062
O43324	Eukaryotic translation elongation factor 1 epsilon-1 OS=Homo sapiens OX=9606 GN=EEF1E1 PE=1 SV=1	0,705218618	0,656	0,792
Q8IZL8	Proline-, glutamic acid- and leucine-rich protein 1 OS=Homo sapiens OX=9606 GN=PELP1 PE=1 SV=2	1,424501425	0,655	0,445
Q9Y673	Dolichyl-phosphate beta-glucosyltransferase OS=Homo sapiens OX=9606 GN=ALG5 PE=1 SV=1	0,902527076	0,655	0,768
Q8TE77	Protein phosphatase Slingshot homolog 3 OS=Homo sapiens OX=9606 GN=SSH3 PE=1 SV=2	0,538793103	0,655	1,192
Q9NW13	RNA-binding protein 28 OS=Homo sapiens OX=9606 GN=RBM28 PE=1 SV=3	1,540832049	0,654	0,53
O43395	U4/U6 small nuclear ribonucleoprotein Prp3 OS=Homo sapiens OX=9606 GN=PRPF3 PE=1 SV=2	0,946073794	0,653	0,602
Q9BYG3	MKI67 FHA domain-interacting nucleolar phosphoprotein OS=Homo sapiens OX=9606 GN=NIFK PE=1 SV=1	1,960784314	0,652	0,45
Q8N163	Cell cycle and apoptosis regulator protein 2 OS=Homo sapiens OX=9606 GN=CCAR2 PE=1 SV=2	1,037344398	0,65	0,549
P61026	Ras-related protein Rab-10 OS=Homo sapiens OX=9606 GN=RAB10 PE=1 SV=1	0,622277536	0,65	1,167
Q9BTD8	RNA-binding protein 42 OS=Homo sapiens OX=9606 GN=RBM42 PE=1 SV=1	0,603500302	0,65	0,949
O75489	NADH dehydrogenase [ubiquinone] iron-sulfur protein 3, mitochondrial OS=Homo sapiens OX=9606 GN=NDUFS3 PE=1 SV=1	0,659630607	0,648	1,011
P25705	ATP synthase subunit alpha, mitochondrial OS=Homo sapiens OX=9606 GN=ATP5F1A PE=1 SV=1	0,716845878	0,647	0,92
P50440	Glycine amidinotransferase, mitochondrial OS=Homo sapiens OX=9606 GN=GATM PE=1 SV=1	1000	0,646	0,001

Q9BQG0	Myb-binding protein 1A OS=Homo sapiens OX=9606 GN=MYBBP1A PE=1 SV=2	2,100840336	0,646	0,317
P09913	Interferon-induced protein with tetratricopeptide repeats 2 OS=Homo sapiens OX=9606 GN=IFIT2 PE=1 SV=1	0,177430802	0,646	5,547
Q9BYD1	Large ribosomal subunit protein uL13m OS=Homo sapiens OX=9606 GN=MRPL13 PE=1 SV=1	1,62601626	0,645	0,664
O76021	Ribosomal L1 domain-containing protein 1 OS=Homo sapiens OX=9606 GN=RSL1D1 PE=1 SV=3	1,264222503	0,645	0,455
P20340	Ras-related protein Rab-6A OS=Homo sapiens OX=9606 GN=RAB6A PE=1 SV=3	0,657462196	0,645	0,958
P06756	Integrin alpha-V OS=Homo sapiens OX=9606 GN=ITGAV PE=1 SV=2	0,365230095	0,645	1,992
P36957	Dihydrolipoyllysine-residue succinyltransferase component of 2-oxoglutarate dehydrogenase complex, mitochondrial OS=H	0,793021412	0,644	0,826
Q8ND30	Liprin-beta-2 OS=Homo sapiens OX=9606 GN=PPFIBP2 PE=1 SV=3	0,620347395	0,643	1,076
P49406	Large ribosomal subunit protein bL19m OS=Homo sapiens OX=9606 GN=MRPL19 PE=1 SV=2	0,853970965	0,642	0,744
Q9BVP2	Guanine nucleotide-binding protein-like 3 OS=Homo sapiens OX=9606 GN=GNL3 PE=1 SV=2	1,579778831	0,641	0,436
P50402	Emerin OS=Homo sapiens OX=9606 GN=EMD PE=1 SV=1	0,579710145	0,64	1,153
O75439	Mitochondrial-processing peptidase subunit beta OS=Homo sapiens OX=9606 GN=PMPCB PE=1 SV=2	0,969932105	0,637	0,63
P07476	Involucrin OS=Homo sapiens OX=9606 GN=IVL PE=1 SV=2		0,636	
Q9Y2W1	Thyroid hormone receptor-associated protein 3 OS=Homo sapiens OX=9606 GN=THRAP3 PE=1 SV=2	1,798561151	0,636	0,327
Q5UIP0	Telomere-associated protein RIF1 OS=Homo sapiens OX=9606 GN=RIF1 PE=1 SV=2	1,540832049	0,636	0,384
Q9Y376	Calcium-binding protein 39 OS=Homo sapiens OX=9606 GN=CAB39 PE=1 SV=1	0,901713255	0,636	0,716
P63010	AP-2 complex subunit beta OS=Homo sapiens OX=9606 GN=AP2B1 PE=1 SV=1	0,630914826	0,636	1,037
Q92841	Probable ATP-dependent RNA helicase DDX17 OS=Homo sapiens OX=9606 GN=DDX17 PE=1 SV=2	1,481481481	0,633	0,419
Q3ZCQ8	Mitochondrial import inner membrane translocase subunit TIM50 OS=Homo sapiens OX=9606 GN=TIMM50 PE=1 SV=2	0,858369099	0,633	0,68
Q8N766	ER membrane protein complex subunit 1 OS=Homo sapiens OX=9606 GN=EMC1 PE=1 SV=1	0,639795266	0,633	1,059
O75844	CAAX prenyl protease 1 homolog OS=Homo sapiens OX=9606 GN=ZMPSTE24 PE=1 SV=2	0,903342367	0,632	0,777
P22307	Sterol carrier protein 2 OS=Homo sapiens OX=9606 GN=SCP2 PE=1 SV=2	0,347342827	0,632	1,52
P61158	Actin-related protein 3 OS=Homo sapiens OX=9606 GN=ACTR3 PE=1 SV=3	0,668896321	0,631	1,072
P21796	Voltage-dependent anion-selective channel protein 1 OS=Homo sapiens OX=9606 GN=VDAC1 PE=1 SV=2	0,539083558	0,631	1,091
O94826	Mitochondrial import receptor subunit TOM70 OS=Homo sapiens OX=9606 GN=TOMM70 PE=1 SV=1	0,871080139	0,63	0,743
P28331	NADH-ubiquinone oxidoreductase 75 kDa subunit, mitochondrial OS=Homo sapiens OX=9606 GN=NDUFS1 PE=1 SV=3	0,726744186	0,63	0,91
P35579	Myosin-9 OS=Homo sapiens OX=9606 GN=MYH9 PE=1 SV=4	0,237812128	0,63	2,467
P99999	Cytochrome c OS=Homo sapiens OX=9606 GN=CYCS PE=1 SV=2	1,034126163	0,629	0,601
P12270	Nucleoprotein TPR OS=Homo sapiens OX=9606 GN=TPR PE=1 SV=3	0,866551127	0,628	0,734
P31930	Cytochrome b-c1 complex subunit 1, mitochondrial OS=Homo sapiens OX=9606 GN=UQCRC1 PE=1 SV=3	0,833333333	0,628	0,799
P29992	Guanine nucleotide-binding protein subunit alpha-11 OS=Homo sapiens OX=9606 GN=GNA11 PE=1 SV=2	0,317057705	0,628	1,911
Q13823	Nucleolar GTP-binding protein 2 OS=Homo sapiens OX=9606 GN=GNL2 PE=1 SV=1	1,908396947	0,627	0,363
Q96GQ7	Probable ATP-dependent RNA helicase DDX27 OS=Homo sapiens OX=9606 GN=DDX27 PE=1 SV=2	1,149425287	0,627	0,43
P10586	Receptor-type tyrosine-protein phosphatase F OS=Homo sapiens OX=9606 GN=PTPRF PE=1 SV=2	0,897666068	0,627	0,759
P45954	Short/branched chain specific acyl-CoA dehydrogenase, mitochondrial OS=Homo sapiens OX=9606 GN=ACADSB PE=1 SV=1	1,461988304	0,626	0,528
Q86VM9	Zinc finger CCCH domain-containing protein 18 OS=Homo sapiens OX=9606 GN=ZC3H18 PE=1 SV=2	1,251564456	0,626	0,283
Q16543	Hsp90 co-chaperone Cdc37 OS=Homo sapiens OX=9606 GN=CDC37 PE=1 SV=1	1,091703057	0,626	0,574
Q14696	LRP chaperone MESD OS=Homo sapiens OX=9606 GN=MESD PE=1 SV=2	0,809061489	0,626	0,76
P43304	Glycerol-3-phosphate dehydrogenase, mitochondrial OS=Homo sapiens OX=9606 GN=GPD2 PE=1 SV=3	0,758150114	0,626	0,794
Q15269	Periodic tryptophan protein 2 homolog OS=Homo sapiens OX=9606 GN=PWP2 PE=1 SV=2	1,470588235	0,625	0,483
Q9NS69	Mitochondrial import receptor subunit TOM22 homolog OS=Homo sapiens OX=9606 GN=TOMM22 PE=1 SV=3	1,013171226	0,624	0,534
O75251	NADH dehydrogenase [ubiquinone] iron-sulfur protein 7, mitochondrial OS=Homo sapiens OX=9606 GN=NDUFS7 PE=1 SV=3	0,851788756	0,624	0,85
Q96I24	Far upstream element-binding protein 3 OS=Homo sapiens OX=9606 GN=FUBP3 PE=1 SV=2	0,762195122	0,623	0,824
O95299	NADH dehydrogenase [ubiquinone] 1 alpha subcomplex subunit 10, mitochondrial OS=Homo sapiens OX=9606 GN=NDUFA1	0,538502962	0,623	1,673
O15118	NPC intracellular cholesterol transporter 1 OS=Homo sapiens OX=9606 GN=NPC1 PE=1 SV=2	0,917431193	0,622	0,716
P46782	Small ribosomal subunit protein uS7 OS=Homo sapiens OX=9606 GN=RPS5 PE=1 SV=4	2,380952381	0,62	0,277
P40939	Trifunctional enzyme subunit alpha, mitochondrial OS=Homo sapiens OX=9606 GN=HADHA PE=1 SV=2	0,771604938	0,62	0,768
Q9NYF8	Bcl-2-associated transcription factor 1 OS=Homo sapiens OX=9606 GN=BCLAF1 PE=1 SV=2	1,655629139	0,619	0,383
Q86XP3	ATP-dependent RNA helicase DDX42 OS=Homo sapiens OX=9606 GN=DDX42 PE=1 SV=1	1,968503937	0,618	0,297
Q92900	Regulator of nonsense transcripts 1 OS=Homo sapiens OX=9606 GN=UPF1 PE=1 SV=2	1,453488372	0,618	0,368
O14975	Long-chain fatty acid transport protein 2 OS=Homo sapiens OX=9606 GN=SLC27A2 PE=1 SV=2	0,171703297	0,618	6,521
P09601	Heme oxygenase 1 OS=Homo sapiens OX=9606 GN=HMOX1 PE=1 SV=1	0,078326937	0,618	7,406
O43520	Phospholipid-transporting ATPase IC OS=Homo sapiens OX=9606 GN=ATP8B1 PE=1 SV=3	0,535905681	0,617	0,474
P35237	Serpin B6 OS=Homo sapiens OX=9606 GN=SERPINB6 PE=1 SV=3	0,237079184	0,616	2,573
Q9Y2X3	Nucleolar protein 58 OS=Homo sapiens OX=9606 GN=NOP58 PE=1 SV=1	1,683501684	0,615	0,343
P51649	Succinate-semialdehyde dehydrogenase, mitochondrial OS=Homo sapiens OX=9606 GN=ALDH5A1 PE=1 SV=2	2,06185567	0,614	0,213
P50570	Dynamin-2 OS=Homo sapiens OX=9606 GN=DNM2 PE=1 SV=2	0,967117988	0,613	0,67
Q13510	Acid ceramidase OS=Homo sapiens OX=9606 GN=ASAH1 PE=1 SV=5	0,407497963	0,613	1,466
Q8TDB6	E3 ubiquitin-protein ligase DTX3L OS=Homo sapiens OX=9606 GN=DTX3L PE=1 SV=1	0,358037952	0,613	1,016
P18583	Protein SON OS=Homo sapiens OX=9606 GN=SON PE=1 SV=4	2	0,612	0,287
P19320	Vascular cell adhesion protein 1 OS=Homo sapiens OX=9606 GN=VCAM1 PE=1 SV=1	0,817661488	0,611	0,811
Q9BZE4	GTP-binding protein 4 OS=Homo sapiens OX=9606 GN=GTPBP4 PE=1 SV=3	1,795332136	0,61	0,345
P09543	2',3'-cyclic-nucleotide 3'-phosphodiesterase OS=Homo sapiens OX=9606 GN=CNP PE=1 SV=2	0,558347292	0,61	1,097
Q9ULW0	Targeting protein for Xklp2 OS=Homo sapiens OX=9606 GN=TPX2 PE=1 SV=2	1,689189189	0,609	0,347
Q96S66	Chloride channel CLIC-like protein 1 OS=Homo sapiens OX=9606 GN=CLCC1 PE=1 SV=1	0,849617672	0,608	0,676
Q4G0N4	NAD kinase 2, mitochondrial OS=Homo sapiens OX=9606 GN=NADK2 PE=1 SV=2	0,729394602	0,607	0,699
P47985	Cytochrome b-c1 complex subunit Rieske, mitochondrial OS=Homo sapiens OX=9606 GN=UQCRFS1 PE=1 SV=2	0,729394602	0,607	0,634
Q16718	NADH dehydrogenase [ubiquinone] 1 alpha subcomplex subunit 5 OS=Homo sapiens OX=9606 GN=NDUFA5 PE=1 SV=3	0,594530321	0,607	1,058
Q9UIJ7	GTP:AMP phosphotransferase AK3, mitochondrial OS=Homo sapiens OX=9606 GN=AK3 PE=1 SV=4	0,471253534	0,607	1,236
Q9NVI7	ATPase family AAA domain-containing protein 3A OS=Homo sapiens OX=9606 GN=ATAD3A PE=1 SV=2	1,414427157	0,606	0,427
Q96EY7	Small ribosomal subunit protein mS39 OS=Homo sapiens OX=9606 GN=PTCD3 PE=1 SV=3	1,182033097	0,605	0,534
Q9Y3D9	Small ribosomal subunit protein mS23 OS=Homo sapiens OX=9606 GN=MRPS23 PE=1 SV=2	1,404494382	0,601	0,459
O76031	ATP-dependent Clp protease ATP-binding subunit clpX-like, mitochondrial OS=Homo sapiens OX=9606 GN=CLPX PE=1 SV=2	1,189060642	0,601	0,467
Q9H3U1	Protein unc-45 homolog A OS=Homo sapiens OX=9606 GN=UNC45A PE=1 SV=1	0,720980534	0,601	0,788
P55957	BH3-interacting domain death agonist OS=Homo sapiens OX=9606 GN=BID PE=1 SV=1	1,636661211	0,6	0,485
P24752	Acetyl-CoA acetyltransferase, mitochondrial OS=Homo sapiens OX=9606 GN=ACAT1 PE=1 SV=1	0,643086817	0,6	0,891
Q9HD45	Transmembrane 9 superfamily member 3 OS=Homo sapiens OX=9606 GN=TM9SF3 PE=1 SV=2	0,499251123	0,598	1,373
Q8WYP5	Protein ELYS OS=Homo sapiens OX=9606 GN=AHCTF1 PE=1 SV=3	1,158748552	0,597	0,502
P09622	Dihydrolipoyl dehydrogenase, mitochondrial OS=Homo sapiens OX=9606 GN=DLD PE=1 SV=2	1,210653753	0,595	0,329
Q32MZ4	Leucine-rich repeat flightless-interacting protein 1 OS=Homo sapiens OX=9606 GN=LRRFIP1 PE=1 SV=2	0,668002672	0,595	0,996
P43007	Neutral amino acid transporter A OS=Homo sapiens OX=9606 GN=SLC1A4 PE=1 SV=1	0,215703192	0,594	6,276
Q86SF2	N-acetylgalactosaminyltransferase 7 OS=Homo sapiens OX=9606 GN=GALNT7 PE=1 SV=1	0,604594921	0,593	0,763
P24539	ATP synthase F(0) complex subunit B1, mitochondrial OS=Homo sapiens OX=9606 GN=ATP5PB PE=1 SV=2	0,790513834	0,592	1,237

P49959	Double-strand break repair protein MRE11 OS=Homo sapiens OX=9606 GN=MRE11 PE=1 SV=3	0,613496933	0,592	0,915
A0A087X1N8	Serpin B6 OS=Homo sapiens OX=9606 GN=SERPINB6 PE=1 SV=1	0,234027615	0,592	2,7
P07686	Beta-hexosaminidase subunit beta OS=Homo sapiens OX=9606 GN=HEXB PE=1 SV=4	0,272331155	0,591	2,286
Q86V48	Leucine zipper protein 1 OS=Homo sapiens OX=9606 GN=LUZP1 PE=1 SV=2	0,772200772	0,59	0,759
Q9UKM9	RNA-binding protein Raly OS=Homo sapiens OX=9606 GN=RALY PE=1 SV=1	1,233045623	0,589	0,495
Q9Y2R9	Small ribosomal subunit protein uS7m OS=Homo sapiens OX=9606 GN=MRPS7 PE=1 SV=2	0,798084597	0,588	0,756
Q14258	E3 ubiquitin/ISG15 ligase TRIM25 OS=Homo sapiens OX=9606 GN=TRIM25 PE=1 SV=2	0,3066544	0,588	1,799
L0R6Q1	SLC35A4 upstream open reading frame protein OS=Homo sapiens OX=9606 GN=SLC35A4 PE=3 SV=1	0,666222518	0,587	0,851
P08621	U1 small nuclear ribonucleoprotein 70 kDa OS=Homo sapiens OX=9606 GN=SNRNP70 PE=1 SV=2	1,46627566	0,586	0,39
Q6PJT7	Zinc finger CCCH domain-containing protein 14 OS=Homo sapiens OX=9606 GN=ZC3H14 PE=1 SV=1	1,236093943	0,586	0,496
P22061	Protein-L-isoaspartate(D-aspartate) O-methyltransferase OS=Homo sapiens OX=9606 GN=PCMT1 PE=1 SV=4	1,184834123	0,585	0,521
Q02978	Mitochondrial 2-oxoglutarate/malate carrier protein OS=Homo sapiens OX=9606 GN=SLC25A11 PE=1 SV=3	0,996015936	0,585	0,528
P67936	Tropomyosin alpha-4 chain OS=Homo sapiens OX=9606 GN=TPM4 PE=1 SV=3	0,237191651	0,583	2,363
Q9NYK5	Large ribosomal subunit protein mL39 OS=Homo sapiens OX=9606 GN=MRPL39 PE=1 SV=3	0,92936803	0,579	0,746
Q6ZRP7	Sulfhydryl oxidase 2 OS=Homo sapiens OX=9606 GN=QSOX2 PE=1 SV=3	0,838926174	0,579	0,642
Q05639	Elongation factor 1-alpha 2 OS=Homo sapiens OX=9606 GN=EEF1A2 PE=1 SV=1	1,529051988	0,578	0,222
Q15050	Ribosome biogenesis regulatory protein homolog OS=Homo sapiens OX=9606 GN=RRS1 PE=1 SV=2	1,383125864	0,578	0,44
Q9Y399	Small ribosomal subunit protein uS2m OS=Homo sapiens OX=9606 GN=MRPS2 PE=1 SV=1	0,839630563	0,578	0,277
Q9ULV4	Coronin-1C OS=Homo sapiens OX=9606 GN=CORO1C PE=1 SV=1	0,816993464	0,577	0,544
Q96I25	Splicing factor 45 OS=Homo sapiens OX=9606 GN=RBM17 PE=1 SV=1	1,226993865	0,576	0,505
Q9NWH9	SAFB-like transcription modulator OS=Homo sapiens OX=9606 GN=SLTM PE=1 SV=2	2,320185615	0,575	0,305
P61604	10 kDa heat shock protein, mitochondrial OS=Homo sapiens OX=9606 GN=HSPE1 PE=1 SV=2	1,577287066	0,574	0,367
Q9P2E9	Ribosome-binding protein 1 OS=Homo sapiens OX=9606 GN=RRBP1 PE=1 SV=5	0,728862974	0,573	0,726
Q9NR12	PDZ and LIM domain protein 7 OS=Homo sapiens OX=9606 GN=PDLIM7 PE=1 SV=1	0,412031314	0,573	1,727
Q07955	Serine/arginine-rich splicing factor 1 OS=Homo sapiens OX=9606 GN=SRSF1 PE=1 SV=2	1,501501502	0,572	0,342
P07910	Heterogeneous nuclear ribonucleoproteins C1/C2 OS=Homo sapiens OX=9606 GN=HNRNPC PE=1 SV=4	0,934579439	0,572	0,64
Q10471	Polypeptide N-acetylgalactosaminyltransferase 2 OS=Homo sapiens OX=9606 GN=GALNT2 PE=1 SV=1	0,326797386	0,572	1,774
P18754	Regulator of chromosome condensation OS=Homo sapiens OX=9606 GN=RCC1 PE=1 SV=1	1,34589502	0,57	0,422
P50552	Vasodilator-stimulated phosphoprotein OS=Homo sapiens OX=9606 GN=VASP PE=1 SV=3	0,299850075	0,57	1,943
Q13268	Dehydrogenase/reductase SDR family member 2, mitochondrial OS=Homo sapiens OX=9606 GN=DHRS2 PE=1 SV=4	0,544365814	0,569	0,324
P09525	Annexin A4 OS=Homo sapiens OX=9606 GN=ANXA4 PE=1 SV=4	0,149521531	0,569	3,894
O00567	Nucleolar protein 56 OS=Homo sapiens OX=9606 GN=NOP56 PE=1 SV=4	1,582278481	0,568	0,349
Q9Y697	Cysteine desulfurase OS=Homo sapiens OX=9606 GN=NFS1 PE=1 SV=3	0,878734622	0,568	0,516
P49821	NADH dehydrogenase [ubiquinone] flavoprotein 1, mitochondrial OS=Homo sapiens OX=9606 GN=NDUFV1 PE=1 SV=4	0,771010023	0,567	0,825
O43399	Tumor protein D54 OS=Homo sapiens OX=9606 GN=TPD52L2 PE=1 SV=2	0,517330574	0,567	1,02
Q14980	Nuclear mitotic apparatus protein 1 OS=Homo sapiens OX=9606 GN=NUMA1 PE=1 SV=2	1,166861144	0,564	0,511
O75152	Zinc finger CCCH domain-containing protein 11A OS=Homo sapiens OX=9606 GN=ZC3H11A PE=1 SV=3	1,161440186	0,563	0,47
O60885	Bromodomain-containing protein 4 OS=Homo sapiens OX=9606 GN=BRD4 PE=1 SV=2	1,447178003	0,562	0,444
Q8IXM6	Nurim OS=Homo sapiens OX=9606 GN=NRM PE=1 SV=1	1,445086705	0,562	1,96
O43464	Serine protease HTRA2, mitochondrial OS=Homo sapiens OX=9606 GN=HTRA2 PE=1 SV=2	1,349527665	0,562	0,516
Q96C36	Pyrroline-5-carboxylate reductase 2 OS=Homo sapiens OX=9606 GN=PYCR2 PE=1 SV=1	0,745712155	0,562	0,704
Q9H6W3	Ribosomal oxygenase 1 OS=Homo sapiens OX=9606 GN=RIOX1 PE=1 SV=2	1,251564456	0,561	0,426
O96008	Mitochondrial import receptor subunit TOM40 homolog OS=Homo sapiens OX=9606 GN=TOMM40 PE=1 SV=1	0,78125	0,56	0,668
P33527	Multidrug resistance-associated protein 1 OS=Homo sapiens OX=9606 GN=ABCC1 PE=1 SV=3	0,273747605	0,559	2,243
Q9H307	Pinin OS=Homo sapiens OX=9606 GN=PNN PE=1 SV=5	1,773049645	0,558	0,321
P55084	Trifunctional enzyme subunit beta, mitochondrial OS=Homo sapiens OX=9606 GN=HADHB PE=1 SV=3	0,761035008	0,558	0,687
Q12849	G-rich sequence factor 1 OS=Homo sapiens OX=9606 GN=GRSF1 PE=1 SV=3	1,107419712	0,557	0,773
P21912	Succinate dehydrogenase [ubiquinone] iron-sulfur subunit, mitochondrial OS=Homo sapiens OX=9606 GN=SDHB PE=1 SV=3	0,580046404	0,556	0,785
P17655	Calpain-2 catalytic subunit OS=Homo sapiens OX=9606 GN=CAPN2 PE=1 SV=6	0,169808117	0,556	2,93
O60568	Multifunctional procollagen lysine hydroxylase and glycosyltransferase LH3 OS=Homo sapiens OX=9606 GN=PLOD3 PE=1 SV	0,455373406	0,555	1,478
Q15075	Early endosome antigen 1 OS=Homo sapiens OX=9606 GN=EEA1 PE=1 SV=2	0,225937641	0,555	2,04
Q09666	Neuroblast differentiation-associated protein AHNAK OS=Homo sapiens OX=9606 GN=AHNAK PE=1 SV=2	0,140252454	0,555	3,765
P37802	Transgelin-2 OS=Homo sapiens OX=9606 GN=TAGLN2 PE=1 SV=3	0,319795331	0,553	1,447
P10606	Cytochrome c oxidase subunit 5B, mitochondrial OS=Homo sapiens OX=9606 GN=COX5B PE=1 SV=2	0,901713255	0,552	0,567
O75477	Erlin-1 OS=Homo sapiens OX=9606 GN=ERLIN1 PE=1 SV=2	0,374251497	0,552	1,506
Q9UQ35	Serine/arginine repetitive matrix protein 2 OS=Homo sapiens OX=9606 GN=SRRM2 PE=1 SV=2	1,261034048	0,551	0,406
P02786	Transferrin receptor protein 1 OS=Homo sapiens OX=9606 GN=TFRC PE=1 SV=2	0,842459983	0,551	0,632
Q99798	Aconitate hydratase, mitochondrial OS=Homo sapiens OX=9606 GN=ACO2 PE=1 SV=2	0,620347395	0,55	0,847
Q9UNF0	Protein kinase C and casein kinase substrate in neurons protein 2 OS=Homo sapiens OX=9606 GN=PACSIN2 PE=1 SV=2	0,513083633	0,55	1,191
P15311	Ezrin OS=Homo sapiens OX=9606 GN=EZR PE=1 SV=4	2,873563218	0,548	0,204
Q96CW1	AP-2 complex subunit mu OS=Homo sapiens OX=9606 GN=AP2M1 PE=1 SV=2	0,558035714	0,548	0,979
Q9BZE1	Large ribosomal subunit protein mL37 OS=Homo sapiens OX=9606 GN=MRPL37 PE=1 SV=2	0,84317032	0,547	0,638
Q08379	Golgin subfamily A member 2 OS=Homo sapiens OX=9606 GN=GOLGA2 PE=1 SV=3	0,77579519	0,547	0,633
P14927	Cytochrome b-c1 complex subunit 7 OS=Homo sapiens OX=9606 GN=UQCRB PE=1 SV=2	0,852514919	0,546	0,707
Q9Y3B7	Large ribosomal subunit protein uL11m OS=Homo sapiens OX=9606 GN=MRPL11 PE=1 SV=1	0,737463127	0,546	0,705
O15460	Prolyl 4-hydroxylase subunit alpha-2 OS=Homo sapiens OX=9606 GN=P4HA2 PE=1 SV=1	0,162443145	0,546	3,243
Q9H9J2	Large ribosomal subunit protein mL44 OS=Homo sapiens OX=9606 GN=MRPL44 PE=1 SV=1	0,801282051	0,545	0,823
P22695	Cytochrome b-c1 complex subunit 2, mitochondrial OS=Homo sapiens OX=9606 GN=UQCRC2 PE=1 SV=3	0,734753857	0,544	0,668
O14672	Disintegrin and metalloproteinase domain-containing protein 10 OS=Homo sapiens OX=9606 GN=ADAM10 PE=1 SV=1	0,491883915	0,544	1,253
P51153	Ras-related protein Rab-13 OS=Homo sapiens OX=9606 GN=RAB13 PE=1 SV=1	0,319081047	0,544	1,784
A0A804HII9	Alpha-actinin-1 OS=Homo sapiens OX=9606 GN=ACTN1 PE=1 SV=1	0,513874615	0,541	0,959
P61106	Ras-related protein Rab-14 OS=Homo sapiens OX=9606 GN=RAB14 PE=1 SV=4	0,259538022	0,54	1,779
Q01082	Spectrin beta chain, non-erythrocytic 1 OS=Homo sapiens OX=9606 GN=SPTBN1 PE=1 SV=2	0,687285223	0,539	0,785
P51398	Small ribosomal subunit protein mS29 OS=Homo sapiens OX=9606 GN=DAP3 PE=1 SV=1	0,829187396	0,538	0,643
P42566	Epidermal growth factor receptor substrate 15 OS=Homo sapiens OX=9606 GN=EPS15 PE=1 SV=2	0,764525994	0,538	0,748
O43169	Cytochrome b5 type B OS=Homo sapiens OX=9606 GN=CYB5B PE=1 SV=3	0,337495781	0,538	1,32
Q9NZN4	EH domain-containing protein 2 OS=Homo sapiens OX=9606 GN=EHD2 PE=1 SV=2	0,039197241	0,537	20,75
P80404	4-aminobutyrate aminotransferase, mitochondrial OS=Homo sapiens OX=9606 GN=ABAT PE=1 SV=3	1,461988304	0,536	1,02
Q14160	Protein scribble homolog OS=Homo sapiens OX=9606 GN=SCRIB PE=1 SV=5	1,187648456	0,536	0,418
P18858	DNA ligase 1 OS=Homo sapiens OX=9606 GN=LIG1 PE=1 SV=1	2,849002849	0,533	0,342
Q9Y512	Sorting and assembly machinery component 50 homolog OS=Homo sapiens OX=9606 GN=SAMM50 PE=1 SV=3	1,182033097	0,533	0,411
Q9P0I2	ER membrane protein complex subunit 3 OS=Homo sapiens OX=9606 GN=EMC3 PE=1 SV=3	0,51072523	0,533	0,931
P15121	Aldo-keto reductase family 1 member B1 OS=Homo sapiens OX=9606 GN=AKR1B1 PE=1 SV=3	0,170794193	0,533	3,036

Q9BTC0	Death-inducer obliterator 1 OS=Homo sapiens OX=9606 GN=DIDO1 PE=1 SV=5	0,831255195	0,532	0,62
Q9UHA4	Ragulator complex protein LAMTOR3 OS=Homo sapiens OX=9606 GN=LAMTOR3 PE=1 SV=1	0,824402308	0,532	0,657
Q7Z2K8	G protein-regulated inducer of neurite outgrowth 1 OS=Homo sapiens OX=9606 GN=GPRIN1 PE=1 SV=2	0,58685446	0,532	0,841
Q9Y305	Acyl-coenzyme A thioesterase 9, mitochondrial OS=Homo sapiens OX=9606 GN=ACOT9 PE=1 SV=2	0,440528634	0,531	1,106
O75381	Peroxisomal membrane protein PEX14 OS=Homo sapiens OX=9606 GN=PEX14 PE=1 SV=1	0,899280576	0,529	0,711
P26440	Isovaleryl-CoA dehydrogenase, mitochondrial OS=Homo sapiens OX=9606 GN=IVD PE=1 SV=2	0,744047619	0,528	0,717
Q12959	Disks large homolog 1 OS=Homo sapiens OX=9606 GN=DLG1 PE=1 SV=2	0,446428571	0,528	1,164
O60763	General vesicular transport factor p115 OS=Homo sapiens OX=9606 GN=USO1 PE=1 SV=2	0,892857143	0,527	0,573
Q14677	Clathrin interactor 1 OS=Homo sapiens OX=9606 GN=CLINT1 PE=1 SV=1	1,088139282	0,523	0,386
Q96RT1	Erbin OS=Homo sapiens OX=9606 GN=ERBIN PE=1 SV=2	0,462748727	0,523	1,219
Q92552	Small ribosomal subunit protein mS27 OS=Homo sapiens OX=9606 GN=MRPS27 PE=1 SV=3	0,768639508	0,519	0,73
P30084	Enoyl-CoA hydratase, mitochondrial OS=Homo sapiens OX=9606 GN=ECHS1 PE=1 SV=4	0,766871166	0,519	0,715
O15143	Actin-related protein 2/3 complex subunit 1B OS=Homo sapiens OX=9606 GN=ARPC1B PE=1 SV=3	0,41356493	0,517	1,227
O95782	AP-2 complex subunit alpha-1 OS=Homo sapiens OX=9606 GN=AP2A1 PE=1 SV=3	0,462748727	0,516	1,05
Q14807	Kinesin-like protein KIF22 OS=Homo sapiens OX=9606 GN=KIF22 PE=1 SV=5	1,19474313	0,515	0,411
O95573	Fatty acid CoA ligase Acsl3 OS=Homo sapiens OX=9606 GN=ACSL3 PE=1 SV=3	0,760456274	0,515	0,693
Q9C0C2	182 kDa tankyrase-1-binding protein OS=Homo sapiens OX=9606 GN=TNKS1BP1 PE=1 SV=4	0,364830354	0,515	1,304
Q9UBC2	Epidermal growth factor receptor substrate 15-like 1 OS=Homo sapiens OX=9606 GN=EPS15L1 PE=1 SV=1	0,883392226	0,514	0,623
Q14247	Src substrate cortactin OS=Homo sapiens OX=9606 GN=CTTN PE=1 SV=2	0,604960678	0,514	0,828
Q15006	ER membrane protein complex subunit 2 OS=Homo sapiens OX=9606 GN=EMC2 PE=1 SV=1	0,479846449	0,514	1,05
O00469	Procollagen-lysine,2-oxoglutarate 5-dioxygenase 2 OS=Homo sapiens OX=9606 GN=PLOD2 PE=1 SV=2	0,106428267	0,514	4,183
O14745	Na(+)/H(+) exchange regulatory cofactor NHE-RF1 OS=Homo sapiens OX=9606 GN=NHERF1 PE=1 SV=4	0,55157198	0,512	0,911
P05556	Integrin beta-1 OS=Homo sapiens OX=9606 GN=ITGB1 PE=1 SV=2	0,145116819	0,512	3,571
Q9H444	Charged multivesicular body protein 4b OS=Homo sapiens OX=9606 GN=CHMP4B PE=1 SV=1	0,851788756	0,509	0,666
Q14318	Peptidyl-prolyl cis-trans isomerase FKBP8 OS=Homo sapiens OX=9606 GN=FKBP8 PE=1 SV=2	0,766283525	0,509	0,681
Q13586	Stromal interaction molecule 1 OS=Homo sapiens OX=9606 GN=STIM1 PE=1 SV=3	0,308451573	0,509	1,626
Q9H6R4	Nucleolar protein 6 OS=Homo sapiens OX=9606 GN=NOL6 PE=1 SV=2	1,206272618	0,508	0,421
A0A0B4J2D5	Putative glutamine amidotransferase-like class 1 domain-containing protein 3B, mitochondrial OS=Homo sapiens OX=9606	0,840336134	0,508	0,687
P52815	Large ribosomal subunit protein bL12m OS=Homo sapiens OX=9606 GN=MRPL12 PE=1 SV=2	0,819000819	0,508	0,52
Q9BW92	Threonine--tRNA ligase, mitochondrial OS=Homo sapiens OX=9606 GN=TARS2 PE=1 SV=1	1,112347052	0,507	0,47
Q13501	Sequestosome-1 OS=Homo sapiens OX=9606 GN=SQSTM1 PE=1 SV=1	0,594883998	0,506	2,438
Q8NFQ8	Torsin-1A-interacting protein 2 OS=Homo sapiens OX=9606 GN=TOR1AIP2 PE=1 SV=1	0,549450549	0,505	1,119
O15027	Protein transport protein Sec16A OS=Homo sapiens OX=9606 GN=SEC16A PE=1 SV=4	0,874890639	0,504	0,462
P49411	Elongation factor Tu, mitochondrial OS=Homo sapiens OX=9606 GN=TUFM PE=1 SV=3	0,621118012	0,504	0,914
O95168	NADH dehydrogenase [ubiquinone] 1 beta subcomplex subunit 4 OS=Homo sapiens OX=9606 GN=NDUFB4 PE=1 SV=3	0,575373993	0,504	1,073
Q16891	MICOS complex subunit MIC60 OS=Homo sapiens OX=9606 GN=IMMT PE=1 SV=1	1,335113485	0,502	0,383
P09914	Interferon-induced protein with tetratricopeptide repeats 1 OS=Homo sapiens OX=9606 GN=IFIT1 PE=1 SV=2	0,181851246	0,501	2,44
O15270	Serine palmitoyltransferase 2 OS=Homo sapiens OX=9606 GN=SPTLC2 PE=1 SV=1	0,760456274	0,499	0,71
P00505	Aspartate aminotransferase, mitochondrial OS=Homo sapiens OX=9606 GN=GOT2 PE=1 SV=3	1,086956522	0,498	0,41
Q15629	Translocating chain-associated membrane protein 1 OS=Homo sapiens OX=9606 GN=TRAM1 PE=1 SV=3	0,275709953	0,497	2,306
P23141	Liver carboxylesterase 1 OS=Homo sapiens OX=9606 GN=CES1 PE=1 SV=2	0,09431293	0,497	9,496
Q9UIQ6	Leucyl-cystinyl aminopeptidase OS=Homo sapiens OX=9606 GN=LNPEP PE=1 SV=3	0,123777695	0,496	3,393
Q9P2R7	Succinate--CoA ligase [ADP-forming] subunit beta, mitochondrial OS=Homo sapiens OX=9606 GN=SUCLA2 PE=1 SV=3	1,101321586	0,492	0,412
P58107	Epiplakin OS=Homo sapiens OX=9606 GN=EPPK1 PE=1 SV=3	0,596658711	0,492	1,193
Q92520	Protein FAM3C OS=Homo sapiens OX=9606 GN=FAM3C PE=1 SV=1	0,481463649	0,492	1,105
P06865	Beta-hexosaminidase subunit alpha OS=Homo sapiens OX=9606 GN=HEXA PE=1 SV=2	0,455788514	0,489	0,962
Q9NSE4	Isoleucine--tRNA ligase, mitochondrial OS=Homo sapiens OX=9606 GN=IARS2 PE=1 SV=2	0,595238095	0,487	0,758
P12814	Alpha-actinin-1 OS=Homo sapiens OX=9606 GN=ACTN1 PE=1 SV=2	0,444247001	0,486	0,971
O14879	Interferon-induced protein with tetratricopeptide repeats 3 OS=Homo sapiens OX=9606 GN=IFIT3 PE=1 SV=1	0,173160173	0,485	2,87
Q8IXI1	Mitochondrial Rho GTPase 2 OS=Homo sapiens OX=9606 GN=RHOT2 PE=1 SV=2	0,753012048	0,483	0,681
Q6UB35	Monofunctional C1-tetrahydrofolate synthase, mitochondrial OS=Homo sapiens OX=9606 GN=MTHFD1L PE=1 SV=1	0,62774639	0,482	0,701
Q13813	Spectrin alpha chain, non-erythrocytic 1 OS=Homo sapiens OX=9606 GN=SPTAN1 PE=1 SV=3	0,625390869	0,48	0,772
Q8NE86	Calcium uniporter protein, mitochondrial OS=Homo sapiens OX=9606 GN=MCU PE=1 SV=1	0,200883889	0,48	2,323
P16989	Y-box-binding protein 3 OS=Homo sapiens OX=9606 GN=YBX3 PE=1 SV=4	1,259445844	0,479	0,416
Q9BQ95	Evolutionarily conserved signaling intermediate in Toll pathway, mitochondrial OS=Homo sapiens OX=9606 GN=ECSIT PE=1	0,791765637	0,477	0,667
Q9H9P8	L-2-hydroxyglutarate dehydrogenase, mitochondrial OS=Homo sapiens OX=9606 GN=L2HGDH PE=1 SV=3	0,672494956	0,477	0,719
P50995	Annexin A11 OS=Homo sapiens OX=9606 GN=ANXA11 PE=1 SV=1	0,291036088	0,477	1,545
Q15005	Signal peptidase complex subunit 2 OS=Homo sapiens OX=9606 GN=SPCS2 PE=1 SV=3	0,626959248	0,476	0,821
P62330	ADP-ribosylation factor 6 OS=Homo sapiens OX=9606 GN=ARF6 PE=1 SV=2	0,480538203	0,476	1,26
Q5T9A4	ATPase family AAA domain-containing protein 3B OS=Homo sapiens OX=9606 GN=ATAD3B PE=1 SV=1	1,474926254	0,475	0,295
Q9Y2S7	Polymerase delta-interacting protein 2 OS=Homo sapiens OX=9606 GN=POLDIP2 PE=1 SV=1	1,256281407	0,474	0,391
A0AV96	RNA-binding protein 47 OS=Homo sapiens OX=9606 GN=RBM47 PE=1 SV=2	0,914076782	0,474	0,813
Q14789	Golgin subfamily B member 1 OS=Homo sapiens OX=9606 GN=GOLGB1 PE=1 SV=2	0,604960678	0,471	0,742
Q9P015	Large ribosomal subunit protein uL15m OS=Homo sapiens OX=9606 GN=MRPL15 PE=1 SV=1	1,022494888	0,469	0,648
P82650	Small ribosomal subunit protein mS22 OS=Homo sapiens OX=9606 GN=MRPS22 PE=1 SV=1	0,838926174	0,469	0,599
Q9NYL9	Tropomodulin-3 OS=Homo sapiens OX=9606 GN=TMOD3 PE=1 SV=1	0,500751127	0,469	0,77
Q96HY6	DDRGK domain-containing protein 1 OS=Homo sapiens OX=9606 GN=DDRGK1 PE=1 SV=2	0,853970965	0,468	0,581
O15031	Plexin-B2 OS=Homo sapiens OX=9606 GN=PLXNB2 PE=1 SV=3	0,516795866	0,467	0,82
P20592	Interferon-induced GTP-binding protein Mx2 OS=Homo sapiens OX=9606 GN=MX2 PE=1 SV=1	1,015228426	0,466	0,927
P35222	Catenin beta-1 OS=Homo sapiens OX=9606 GN=CTNNB1 PE=1 SV=1	0,963391137	0,466	0,524
Q15646	2'-5'-oligoadenylate synthase-like protein OS=Homo sapiens OX=9606 GN=OASL PE=1 SV=2	0,092954081	0,466	5,752
P05997	Collagen alpha-2(V) chain OS=Homo sapiens OX=9606 GN=COL5A2 PE=1 SV=3	0,275709953	0,463	2,347
A0FGR8	Extended synaptotagmin-2 OS=Homo sapiens OX=9606 GN=ESYT2 PE=1 SV=1	0,273972603	0,463	1,642
Q9BV38	WD repeat-containing protein 18 OS=Homo sapiens OX=9606 GN=WDR18 PE=1 SV=2	1,112347052	0,462	0,427
Q9Y5K6	CD2-associated protein OS=Homo sapiens OX=9606 GN=CD2AP PE=1 SV=1	0,291120815	0,462	1,545
P23368	NAD-dependent malic enzyme, mitochondrial OS=Homo sapiens OX=9606 GN=ME2 PE=1 SV=1	1,246882793	0,458	0,372
O60218	Aldo-keto reductase family 1 member B10 OS=Homo sapiens OX=9606 GN=AKR1B10 PE=1 SV=2	0,096814793	0,454	3,814
Q13084	Large ribosomal subunit protein bL28m OS=Homo sapiens OX=9606 GN=MRPL28 PE=1 SV=4	0,727802038	0,453	0,377
P07384	Calpain-1 catalytic subunit OS=Homo sapiens OX=9606 GN=CAPN1 PE=1 SV=1	0,599880024	0,451	0,781
P21964	Catechol O-methyltransferase OS=Homo sapiens OX=9606 GN=COMT PE=1 SV=2	0,154392466	0,451	2,397
Q96RP9	Elongation factor G, mitochondrial OS=Homo sapiens OX=9606 GN=GFM1 PE=1 SV=2	0,54884742	0,45	0,833
Q9HD20	Endoplasmic reticulum transmembrane helix translocase OS=Homo sapiens OX=9606 GN=ATP13A1 PE=1 SV=2	0,318979266	0,45	1,326
Q13428	Treacle protein OS=Homo sapiens OX=9606 GN=TCOF1 PE=1 SV=3	3,154574132	0,448	0,135

Q96JB5	CDK5 regulatory subunit-associated protein 3 OS=Homo sapiens OX=9606 GN=CDK5RAP3 PE=1 SV=2	0,545553737	0,448	0,685
Q9H2U2	Inorganic pyrophosphatase 2, mitochondrial OS=Homo sapiens OX=9606 GN=PPA2 PE=1 SV=2	1,060445387	0,447	0,479
P42765	3-ketoacyl-CoA thiolase, mitochondrial OS=Homo sapiens OX=9606 GN=ACAA2 PE=1 SV=2	1,008064516	0,446	0,451
Q6NUQ4	Transmembrane protein 214 OS=Homo sapiens OX=9606 GN=TMEM214 PE=1 SV=2	0,754147813	0,443	0,62
Q9BTT6	Leucine-rich repeat-containing protein 1 OS=Homo sapiens OX=9606 GN=LRRC1 PE=1 SV=1	0,147427392	0,443	3,02
Q9NPL8	Complex I assembly factor TIMMDC1, mitochondrial OS=Homo sapiens OX=9606 GN=TIMMDC1 PE=1 SV=2	0,695410292	0,442	0,686
Q9BYD6	Large ribosomal subunit protein uL1m OS=Homo sapiens OX=9606 GN=MRPL1 PE=1 SV=2	0,666222518	0,442	0,726
Q9BRJ2	Large ribosomal subunit protein mL45 OS=Homo sapiens OX=9606 GN=MRPL45 PE=1 SV=2	0,773993808	0,44	0,619
P67812	Signal peptidase complex catalytic subunit SEC11A OS=Homo sapiens OX=9606 GN=SEC11A PE=1 SV=1	0,609384522	0,439	0,817
O43818	U3 small nucleolar RNA-interacting protein 2 OS=Homo sapiens OX=9606 GN=RRP9 PE=1 SV=1	1,828153565	0,438	0,257
Q9UH99	SUN domain-containing protein 2 OS=Homo sapiens OX=9606 GN=SUN2 PE=1 SV=3	0,423370025	0,438	1,027
Q6YN16	Hydroxysteroid dehydrogenase-like protein 2 OS=Homo sapiens OX=9606 GN=HSDL2 PE=1 SV=1	0,341180484	0,437	1,275
Q8N5N7	Large ribosomal subunit protein mL50 OS=Homo sapiens OX=9606 GN=MRPL50 PE=1 SV=2	0,577367206	0,435	0,608
Q13439	Golgin subfamily A member 4 OS=Homo sapiens OX=9606 GN=GOLGA4 PE=1 SV=1	0,620732464	0,434	0,947
Q15067	Peroxisomal acyl-coenzyme A oxidase 1 OS=Homo sapiens OX=9606 GN=ACOX1 PE=1 SV=3	0,463606861	0,432	0,905
Q8WXG1	S-adenosylmethionine-dependent nucleotide dehydratase RSAD2 OS=Homo sapiens OX=9606 GN=RSAD2 PE=1 SV=1	0,43956044	0,43	1,483
P13797	Plastin-3 OS=Homo sapiens OX=9606 GN=PLS3 PE=1 SV=4	0,384172109	0,43	1,065
Q9Y2D5	PALM2-AKAP2 fusion protein OS=Homo sapiens OX=9606 GN=PALM2AKAP2 PE=1 SV=4	0,32435939	0,428	1,19
O95786	Antiviral innate immune response receptor RIG-I OS=Homo sapiens OX=9606 GN=RIGI PE=1 SV=2	0,310366232	0,427	1,404
P23786	Carnitine O-palmitoyltransferase 2, mitochondrial OS=Homo sapiens OX=9606 GN=CPT2 PE=1 SV=2	0,904159132	0,423	0,562
Q9H845	Complex I assembly factor ACAD9, mitochondrial OS=Homo sapiens OX=9606 GN=ACAD9 PE=1 SV=1	0,724112962	0,423	0,553
O14656	Torsin-1A OS=Homo sapiens OX=9606 GN=TOR1A PE=1 SV=1	0,398724083	0,423	1,149
Q9BQP7	Mitochondrial genome maintenance exonuclease 1 OS=Homo sapiens OX=9606 GN=MGME1 PE=1 SV=1	1,582278481	0,422	0,28
Q13505	Metaxin-1 OS=Homo sapiens OX=9606 GN=MTX1 PE=1 SV=3	1,136363636	0,422	0,354
O75746	Electrogenic aspartate/glutamate antiporter SLC25A12, mitochondrial OS=Homo sapiens OX=9606 GN=SLC25A12 PE=1 SV=2	0,492125984	0,422	0,774
Q9H223	EH domain-containing protein 4 OS=Homo sapiens OX=9606 GN=EHD4 PE=1 SV=1	0,410509031	0,421	0,938
O00148	ATP-dependent RNA helicase DDX39A OS=Homo sapiens OX=9606 GN=DDX39A PE=1 SV=2	1,479289941	0,42	0,266
Q13523	Serine/threonine-protein kinase PRP4 homolog OS=Homo sapiens OX=9606 GN=PRPF4B PE=1 SV=3	1,018329939	0,419	0,372
Q5SRE5	Nucleoporin NUP188 OS=Homo sapiens OX=9606 GN=NUP188 PE=1 SV=1	0,938967136	0,417	0,553
Q9Y4W6	AFG3-like protein 2 OS=Homo sapiens OX=9606 GN=AFG3L2 PE=1 SV=2	0,714285714	0,416	0,633
P23229	Integrin alpha-6 OS=Homo sapiens OX=9606 GN=ITGA6 PE=1 SV=5	0,603864734	0,416	0,676
P42224	Signal transducer and activator of transcription 1-alpha/beta OS=Homo sapiens OX=9606 GN=STAT1 PE=1 SV=2	0,244857982	0,414	1,79
Q9Y2R0	Cytochrome c oxidase assembly factor 3 homolog, mitochondrial OS=Homo sapiens OX=9606 GN=COA3 PE=1 SV=1	0,506329114	0,413	0,669
O15173	Membrane-associated progesterone receptor component 2 OS=Homo sapiens OX=9606 GN=PGRMC2 PE=1 SV=1	0,409668169	0,413	0,992
P19525	Interferon-induced, double-stranded RNA-activated protein kinase OS=Homo sapiens OX=9606 GN=EIF2AK2 PE=1 SV=2	0,526592944	0,412	0,828
P18859	ATP synthase-coupling factor 6, mitochondrial OS=Homo sapiens OX=9606 GN=ATP5PF PE=1 SV=1	0,697836706	0,41	0,575
Q9Y320	Thioredoxin-related transmembrane protein 2 OS=Homo sapiens OX=9606 GN=TMX2 PE=1 SV=1	0,594530321	0,41	0,655
Q9UJZ1	Stomatin-like protein 2, mitochondrial OS=Homo sapiens OX=9606 GN=STOML2 PE=1 SV=1	0,604960678	0,409	0,713
P04062	Lysosomal acid glucosylceramidase OS=Homo sapiens OX=9606 GN=GBA1 PE=1 SV=3	0,280112045	0,406	1,654
O00330	Pyruvate dehydrogenase protein X component, mitochondrial OS=Homo sapiens OX=9606 GN=PDHX PE=1 SV=3	0,782472613	0,404	0,644
Q9UP95	Solute carrier family 12 member 4 OS=Homo sapiens OX=9606 GN=SLC12A4 PE=1 SV=2	0,477326969	0,403	0,968
Q9H4M9	EH domain-containing protein 1 OS=Homo sapiens OX=9606 GN=EHD1 PE=1 SV=2	0,35932447	0,403	1,05
Q9UBQ7	Glyoxylate reductase/hydroxypyruvate reductase OS=Homo sapiens OX=9606 GN=GRHPR PE=1 SV=1	0,746268657	0,402	0,77
Q9HCC0	Methylcrotonoyl-CoA carboxylase beta chain, mitochondrial OS=Homo sapiens OX=9606 GN=MCCC2 PE=1 SV=1	0,532481363	0,4	0,69
Q8WXX5	DnaJ homolog subfamily C member 9 OS=Homo sapiens OX=9606 GN=DNAJC9 PE=1 SV=1	1,373626374	0,399	0,278
Q9Y4P3	Transducin beta-like protein 2 OS=Homo sapiens OX=9606 GN=TBL2 PE=1 SV=1	0,766871166	0,397	0,651
Q15031	Leucine--tRNA ligase, mitochondrial OS=Homo sapiens OX=9606 GN=LARS2 PE=1 SV=2	0,653167864	0,397	0,68
Q6DD88	Atlastin-3 OS=Homo sapiens OX=9606 GN=ATL3 PE=1 SV=1	0,23557126	0,395	1,608
P04632	Calpain small subunit 1 OS=Homo sapiens OX=9606 GN=CAPNS1 PE=1 SV=1	0,229252636	0,395	1,816
P14314	Glucosidase 2 subunit beta OS=Homo sapiens OX=9606 GN=PRKCSH PE=1 SV=2	0,589970501	0,393	0,717
Q8TCS8	Polyribonucleotide nucleotidyltransferase 1, mitochondrial OS=Homo sapiens OX=9606 GN=PNPT1 PE=1 SV=2	0,609013398	0,392	0,72
Q13057	Bifunctional coenzyme A synthase OS=Homo sapiens OX=9606 GN=COASY PE=1 SV=4	0,529100529	0,392	0,681
P17931	Galectin-3 OS=Homo sapiens OX=9606 GN=LGALS3 PE=1 SV=5	0,093632959	0,392	4,218
O94874	E3 UFM1-protein ligase 1 OS=Homo sapiens OX=9606 GN=UFL1 PE=1 SV=2	0,69637883	0,391	0,679
P31937	3-hydroxyisobutyrate dehydrogenase, mitochondrial OS=Homo sapiens OX=9606 GN=HIBADH PE=1 SV=2	0,237191651	0,391	1,708
Q9Y6M5	Proton-coupled zinc antiporter SLC30A1 OS=Homo sapiens OX=9606 GN=SLC30A1 PE=1 SV=3	0,107181136	0,39	2,53
Q9NX63	MICOS complex subunit MIC19 OS=Homo sapiens OX=9606 GN=CHCHD3 PE=1 SV=1	1,114827202	0,386	0,36
Q12931	Heat shock protein 75 kDa, mitochondrial OS=Homo sapiens OX=9606 GN=TRAP1 PE=1 SV=3	0,931098696	0,386	0,434
Q9NZ01	Very-long-chain enoyl-CoA reductase OS=Homo sapiens OX=9606 GN=TECR PE=1 SV=1	0,794281176	0,383	0,428
P04179	Superoxide dismutase [Mn], mitochondrial OS=Homo sapiens OX=9606 GN=SOD2 PE=1 SV=3	0,170852554	0,383	2,012
P15924	Desmoplakin OS=Homo sapiens OX=9606 GN=DSP PE=1 SV=3	0,822368421	0,382	0,497
Q9BQS8	FYVE and coiled-coil domain-containing protein 1 OS=Homo sapiens OX=9606 GN=FYCO1 PE=1 SV=3	0,751879699	0,382	0,529
P42126	Enoyl-CoA delta isomerase 1, mitochondrial OS=Homo sapiens OX=9606 GN=ECI1 PE=1 SV=1	0,404694456	0,379	0,901
Q8TAE8	Large ribosomal subunit protein mL64 OS=Homo sapiens OX=9606 GN=GADD45GIP1 PE=1 SV=1	0,904159132	0,377	0,419
Q9BYN8	Small ribosomal subunit protein mS26 OS=Homo sapiens OX=9606 GN=MRPS26 PE=1 SV=1	0,604960678	0,377	0,536
A0A1Y0BRN3	Unconventional myosin-VI OS=Homo sapiens OX=9606 GN=MYO6 PE=1 SV=1	3,105590062	0,374	0,111
Q9UHB6	LIM domain and actin-binding protein 1 OS=Homo sapiens OX=9606 GN=LIMA1 PE=1 SV=1	0,19054878	0,374	1,46
O15400	Syntaxin-7 OS=Homo sapiens OX=9606 GN=STX7 PE=1 SV=4	0,88028169	0,373	0,534
P16278	Beta-galactosidase OS=Homo sapiens OX=9606 GN=GLB1 PE=1 SV=2	0,403877221	0,372	0,86
P40616	ADP-ribosylation factor-like protein 1 OS=Homo sapiens OX=9606 GN=ARL1 PE=1 SV=1	0,470588235	0,371	0,861
P23497	Nuclear autoantigen Sp-100 OS=Homo sapiens OX=9606 GN=SP100 PE=1 SV=3	1,98019802	0,37	0,659
Q8TBA6	Golgin subfamily A member 5 OS=Homo sapiens OX=9606 GN=GOLGA5 PE=1 SV=3	0,544662309	0,37	0,529
Q9H2W6	Large ribosomal subunit protein mL46 OS=Homo sapiens OX=9606 GN=MRPL46 PE=1 SV=1	0,628140704	0,369	0,616
Q9HC07	Putative divalent cation/proton antiporter TMEM165 OS=Homo sapiens OX=9606 GN=TMEM165 PE=1 SV=1	0,268672757	0,369	1,442
Q92878	DNA repair protein RAD50 OS=Homo sapiens OX=9606 GN=RAD50 PE=1 SV=1	0,406504065	0,367	0,937
Q5M775	Cytospin-B OS=Homo sapiens OX=9606 GN=SPECC1 PE=1 SV=1	0,387596899	0,367	0,931
O43795	Unconventional myosin-Ib OS=Homo sapiens OX=9606 GN=MYO1B PE=1 SV=3	0,996015936	0,365	0,402
P33121	Long-chain-fatty-acid--CoA ligase 1 OS=Homo sapiens OX=9606 GN=ACSL1 PE=1 SV=1	0,923361034	0,364	0,524
O43823	A-kinase anchor protein 8 OS=Homo sapiens OX=9606 GN=AKAP8 PE=1 SV=1	1,015228426	0,362	0,391
Q9NWU5	Large ribosomal subunit protein uL22m OS=Homo sapiens OX=9606 GN=MRPL22 PE=1 SV=1	0,771010023	0,362	0,542
Q15149	Plectin OS=Homo sapiens OX=9606 GN=PLEC PE=1 SV=3	0,303214069	0,359	1,028
Q9NX40	OCIA domain-containing protein 1 OS=Homo sapiens OX=9606 GN=OCIAD1 PE=1 SV=1	0,643086817	0,356	0,565
P19634	Sodium/hydrogen exchanger 1 OS=Homo sapiens OX=9606 GN=SLC9A1 PE=1 SV=2	0,432900433	0,353	0,656

Q96TA1	Protein Niban 2 OS=Homo sapiens OX=9606 GN=NIBAN2 PE=1 SV=3	0,188146754	0,353	2,038
P42892	Endothelin-converting enzyme 1 OS=Homo sapiens OX=9606 GN=ECE1 PE=1 SV=2	0,288933834	0,352	1,282
Q14108	Lysosome membrane protein 2 OS=Homo sapiens OX=9606 GN=SCARB2 PE=1 SV=2	0,392927308	0,349	0,871
P30511	HLA class I histocompatibility antigen, alpha chain F OS=Homo sapiens OX=9606 GN=HLA-F PE=1 SV=3	0,660501982	0,348	0,557
O94901	SUN domain-containing protein 1 OS=Homo sapiens OX=9606 GN=SUN1 PE=1 SV=4	0,312793244	0,348	1,107
Q9H0H5	Rac GTPase-activating protein 1 OS=Homo sapiens OX=9606 GN=RACGAP1 PE=1 SV=1	0,982318271	0,345	0,331
O00519	Fatty-acid amide hydrolase 1 OS=Homo sapiens OX=9606 GN=FAAH PE=1 SV=2	0,666666667	0,345	0,507
P07355	Annexin A2 OS=Homo sapiens OX=9606 GN=ANXA2 PE=1 SV=2	0,15725743	0,345	2,175
O43707	Alpha-actinin-4 OS=Homo sapiens OX=9606 GN=ACTN4 PE=1 SV=2	0,379650721	0,344	0,905
Q96DB5	Regulator of microtubule dynamics protein 1 OS=Homo sapiens OX=9606 GN=RMDN1 PE=1 SV=1	0,55157198	0,342	0,655
P10619	Lysosomal protective protein OS=Homo sapiens OX=9606 GN=CTSA PE=1 SV=2	0,237473284	0,341	1,83
Q9BZG1	Ras-related protein Rab-34 OS=Homo sapiens OX=9606 GN=RAB34 PE=1 SV=1	1,34589502	0,339	0,134
P35221	Catenin alpha-1 OS=Homo sapiens OX=9606 GN=CTNNA1 PE=1 SV=1	0,595947557	0,339	0,516
Q7L5N7	Lysophosphatidylcholine acyltransferase 2 OS=Homo sapiens OX=9606 GN=LPCAT2 PE=1 SV=1	0,658327847	0,338	1,749
Q8NBJ5	Procollagen galactosyltransferase 1 OS=Homo sapiens OX=9606 GN=COLGALT1 PE=1 SV=1	0,259000259	0,337	1,387
P20591	Interferon-induced GTP-binding protein Mx1 OS=Homo sapiens OX=9606 GN=MX1 PE=1 SV=4	0,097446891	0,337	3,424
P30837	Aldehyde dehydrogenase X, mitochondrial OS=Homo sapiens OX=9606 GN=ALDH1B1 PE=1 SV=4	0,716845878	0,336	0,459
P09110	3-ketoacyl-CoA thiolase, peroxisomal OS=Homo sapiens OX=9606 GN=ACAA1 PE=1 SV=2	0,409836066	0,336	0,779
Q9Y3Z3	Deoxynucleoside triphosphate triphosphohydrolase SAMHD1 OS=Homo sapiens OX=9606 GN=SAMHD1 PE=1 SV=2	0,169664065	0,335	1,961
Q5JTV8	Torsin-1A-interacting protein 1 OS=Homo sapiens OX=9606 GN=TOR1AIP1 PE=1 SV=2	0,401606426	0,332	0,796
O15439	ATP-binding cassette sub-family C member 4 OS=Homo sapiens OX=9606 GN=ABCC4 PE=1 SV=3	0,388802488	0,331	1,032
P20810	Calpastatin OS=Homo sapiens OX=9606 GN=CAST PE=1 SV=4	0,294204178	0,331	1,097
O76024	Wolframin OS=Homo sapiens OX=9606 GN=WFS1 PE=1 SV=2	0,22568269	0,331	1,563
P30519	Heme oxygenase 2 OS=Homo sapiens OX=9606 GN=HMOX2 PE=1 SV=2	0,387596899	0,33	0,88
H0YMW4	Annexin OS=Homo sapiens OX=9606 GN=ANXA2 PE=1 SV=2	0,165672631	0,327	2,009
P11166	Solute carrier family 2, facilitated glucose transporter member 1 OS=Homo sapiens OX=9606 GN=SLC2A1 PE=1 SV=2	0,619195046	0,324	0,524
Q6P1M0	Long-chain fatty acid transport protein 4 OS=Homo sapiens OX=9606 GN=SLC27A4 PE=1 SV=1	0,729927007	0,322	0,381
Q9HAV7	GrpE protein homolog 1, mitochondrial OS=Homo sapiens OX=9606 GN=GRPEL1 PE=1 SV=2	0,762776506	0,321	0,403
Q96I99	Succinate--CoA ligase [GDP-forming] subunit beta, mitochondrial OS=Homo sapiens OX=9606 GN=SUCLG2 PE=1 SV=2	0,482858522	0,317	0,69
O75521	Enoyl-CoA delta isomerase 2 OS=Homo sapiens OX=9606 GN=ECI2 PE=1 SV=4	1,338688086	0,316	0,257
P55327	Tumor protein D52 OS=Homo sapiens OX=9606 GN=TPD52 PE=1 SV=2	1,288659794	0,316	0,261
Q9UM54	Unconventional myosin-VI OS=Homo sapiens OX=9606 GN=MYO6 PE=1 SV=4	2,645502646	0,315	0,129
O43837	Isocitrate dehydrogenase [NAD] subunit beta, mitochondrial OS=Homo sapiens OX=9606 GN=IDH3B PE=1 SV=2	0,493583416	0,315	0,573
P13796	Plastin-2 OS=Homo sapiens OX=9606 GN=LCP1 PE=1 SV=6	0,861326443	0,312	0,647
P46013	Proliferation marker protein Ki-67 OS=Homo sapiens OX=9606 GN=MKI67 PE=1 SV=2	1,552795031	0,311	0,201
Q9BS26	Endoplasmic reticulum resident protein 44 OS=Homo sapiens OX=9606 GN=ERP44 PE=1 SV=1	0,364298725	0,311	0,857
P04083	Annexin A1 OS=Homo sapiens OX=9606 GN=ANXA1 PE=1 SV=2	0,16772895	0,31	1,742
O95202	Mitochondrial proton/calcium exchanger protein OS=Homo sapiens OX=9606 GN=LETM1 PE=1 SV=1	0,776397516	0,309	0,376
P54819	Adenylate kinase 2, mitochondrial OS=Homo sapiens OX=9606 GN=AK2 PE=1 SV=2	0,507099391	0,309	0,641
P50213	Isocitrate dehydrogenase [NAD] subunit alpha, mitochondrial OS=Homo sapiens OX=9606 GN=IDH3A PE=1 SV=1	0,675675676	0,307	0,51
P10253	Lysosomal alpha-glucosidase OS=Homo sapiens OX=9606 GN=GAA PE=1 SV=4	1,005025126	0,306	0,626
P25685	DnaJ homolog subfamily B member 1 OS=Homo sapiens OX=9606 GN=DNAJB1 PE=1 SV=4	0,964320154	0,306	0,229
A1L0T0	2-hydroxyacyl-CoA lyase 2 OS=Homo sapiens OX=9606 GN=ILVBL PE=1 SV=2	0,598802395	0,306	0,5
Q14126	Desmoglein-2 OS=Homo sapiens OX=9606 GN=DSG2 PE=1 SV=2	1,114827202	0,304	0,042
Q92817	Envoplakin OS=Homo sapiens OX=9606 GN=EVPL PE=1 SV=3	0,348310693	0,303	1,287
P54725	UV excision repair protein RAD23 homolog A OS=Homo sapiens OX=9606 GN=RAD23A PE=1 SV=1	0,549148819	0,302	0,496
P13804	Electron transfer flavoprotein subunit alpha, mitochondrial OS=Homo sapiens OX=9606 GN=ETFA PE=1 SV=1	0,313087038	0,3	1,006
Q9UKG9	Peroxisomal carnitine O-octanoyltransferase OS=Homo sapiens OX=9606 GN=CROT PE=1 SV=2	0,390625	0,299	0,853
Q460N5	Protein mono-ADP-ribosyltransferase PARP14 OS=Homo sapiens OX=9606 GN=PARP14 PE=1 SV=3	0,279251606	0,298	1,123
Q8IVT2	Mitotic interactor and substrate of PLK1 OS=Homo sapiens OX=9606 GN=MISP PE=1 SV=1	1000	0,296	0,001
Q16666	Gamma-interferon-inducible protein 16 OS=Homo sapiens OX=9606 GN=IFI16 PE=1 SV=3	0,151080224	0,296	1,994
Q01813	ATP-dependent 6-phosphofructokinase, platelet type OS=Homo sapiens OX=9606 GN=PFKP PE=1 SV=2	0,251889169	0,295	1,345
Q96RQ3	Methylcrotonoyl-CoA carboxylase subunit alpha, mitochondrial OS=Homo sapiens OX=9606 GN=MCCC1 PE=1 SV=3	0,660938533	0,294	0,412
Q8WWI1	LIM domain only protein 7 OS=Homo sapiens OX=9606 GN=LMO7 PE=1 SV=3	0,147557916	0,292	2,35
Q9UL46	Proteasome activator complex subunit 2 OS=Homo sapiens OX=9606 GN=PSME2 PE=1 SV=4	0,325203252	0,291	0,985
Q14764	Major vault protein OS=Homo sapiens OX=9606 GN=MVP PE=1 SV=4	0,128435654	0,291	5,413
Q96HE7	ERO1-like protein alpha OS=Homo sapiens OX=9606 GN=ERO1A PE=1 SV=2	0,333889816	0,289	0,858
O75356	Nucleoside diphosphate phosphatase ENTPD5 OS=Homo sapiens OX=9606 GN=ENTPD5 PE=1 SV=1	0,422654269	0,288	0,617
P46940	Ras GTPase-activating-like protein IQGAP1 OS=Homo sapiens OX=9606 GN=IQGAP1 PE=1 SV=1	0,202224469	0,288	1,405
O14787	Transportin-2 OS=Homo sapiens OX=9606 GN=TNPO2 PE=1 SV=3	0,913242009	0,286	0,236
O00186	Syntaxin-binding protein 3 OS=Homo sapiens OX=9606 GN=STXBP3 PE=1 SV=2	0,302388872	0,286	0,912
P01024	Complement C3 OS=Homo sapiens OX=9606 GN=C3 PE=1 SV=2	0,282565697	0,286	2,854
Q8TCT9	Minor histocompatibility antigen H13 OS=Homo sapiens OX=9606 GN=HM13 PE=1 SV=1	0,760456274	0,285	0,368
O14908	PDZ domain-containing protein GIPC1 OS=Homo sapiens OX=9606 GN=GIPC1 PE=1 SV=2	0,609756098	0,284	0,498
O94832	Unconventional myosin-Id OS=Homo sapiens OX=9606 GN=MYO1D PE=1 SV=2	1,307189542	0,283	0,256
Q9BWM7	Sideroflexin-3 OS=Homo sapiens OX=9606 GN=SFXN3 PE=1 SV=3	0,104395031	0,282	3,084
P38117	Electron transfer flavoprotein subunit beta OS=Homo sapiens OX=9606 GN=ETFB PE=1 SV=3	0,289351852	0,281	0,953
Q8NBN7	Retinol dehydrogenase 13 OS=Homo sapiens OX=9606 GN=RDH13 PE=1 SV=2	1,386962552	0,279	0,253
O60437	Periplakin OS=Homo sapiens OX=9606 GN=PPL PE=1 SV=4	1,066098081	0,279	0,57
O00151	PDZ and LIM domain protein 1 OS=Homo sapiens OX=9606 GN=PDLIM1 PE=1 SV=4	0,248818114	0,279	1,07
Q9H7Z7	Prostaglandin E synthase 2 OS=Homo sapiens OX=9606 GN=PTGES2 PE=1 SV=1	0,560538117	0,278	0,652
P00533	Epidermal growth factor receptor OS=Homo sapiens OX=9606 GN=EGFR PE=1 SV=2	0,35971223	0,276	0,751
A0A2R8YDH4	Tight junction protein ZO-2 OS=Homo sapiens OX=9606 PE=1 SV=1	1,226993865	0,275	0,307
O96005	Putative lipid scramblase CLPTM1 OS=Homo sapiens OX=9606 GN=CLPTM1 PE=1 SV=1	0,583771162	0,275	0,492
P07339	Cathepsin D OS=Homo sapiens OX=9606 GN=CTSD PE=1 SV=1	0,560538117	0,275	0,504
Q7Z2K6	Endoplasmic reticulum metallopeptidase 1 OS=Homo sapiens OX=9606 GN=ERMP1 PE=1 SV=2	0,077172403	0,273	3,163
P35659	Protein DEK OS=Homo sapiens OX=9606 GN=DEK PE=1 SV=1	2,150537634	0,272	0,124
Q96HY7	2-oxoadipate dehydrogenase complex component E1 OS=Homo sapiens OX=9606 GN=DHTKD1 PE=1 SV=2	1,251564456	0,268	0,21
Q02241	Kinesin-like protein KIF23 OS=Homo sapiens OX=9606 GN=KIF23 PE=1 SV=3	1,757469244	0,266	0,168
Q00577	Transcriptional activator protein Pur-alpha OS=Homo sapiens OX=9606 GN=PURA PE=1 SV=2	0,363240102	0,266	0,941
P21589	5'-nucleotidase OS=Homo sapiens OX=9606 GN=NT5E PE=1 SV=1	0,062030891	0,266	4,339
Q96C19	EF-hand domain-containing protein D2 OS=Homo sapiens OX=9606 GN=EFHD2 PE=1 SV=1	0,822368421	0,265	0,332
Q6KB66	Keratin, type II cytoskeletal 80 OS=Homo sapiens OX=9606 GN=KRT80 PE=1 SV=2	0,158227848	0,265	1,644

O15254	Peroxisomal acyl-coenzyme A oxidase 3 OS=Homo sapiens OX=9606 GN=ACOX3 PE=1 SV=2	1,001001001	0,263	0,225
Q9H4G0	Band 4.1-like protein 1 OS=Homo sapiens OX=9606 GN=EPB41L1 PE=1 SV=2	0,34106412	0,262	1,071
Q9NZB2	Constitutive coactivator of PPAR-gamma-like protein 1 OS=Homo sapiens OX=9606 GN=FAM120A PE=1 SV=2	0,330578512	0,251	0,68
Q12846	Syntaxin-4 OS=Homo sapiens OX=9606 GN=STX4 PE=1 SV=2	0,268312316	0,247	0,747
Q16822	Phosphoenolpyruvate carboxykinase [GTP], mitochondrial OS=Homo sapiens OX=9606 GN=PCK2 PE=1 SV=4	0,293513355	0,245	0,852
J3KQL8	Apolipoprotein L2 OS=Homo sapiens OX=9606 GN=APOL2 PE=1 SV=2	0,15211439	0,243	0,951
Q9NR46	Endophilin-B2 OS=Homo sapiens OX=9606 GN=SH3GLB2 PE=1 SV=1	0,577367206	0,242	0,37
Q8IY21	Probable ATP-dependent RNA helicase DDX60 OS=Homo sapiens OX=9606 GN=DDX60 PE=1 SV=3	0,380517504	0,239	0,725
Q13751	Laminin subunit beta-3 OS=Homo sapiens OX=9606 GN=LAMB3 PE=1 SV=1	0,257466529	0,237	0,965
Q7Z434	Mitochondrial antiviral-signaling protein OS=Homo sapiens OX=9606 GN=MAVS PE=1 SV=2	0,775193798	0,229	0,269
Q6PI48	Aspartate--tRNA ligase, mitochondrial OS=Homo sapiens OX=9606 GN=DARS2 PE=1 SV=1	0,658761528	0,229	0,356
O00159	Unconventional myosin-Ic OS=Homo sapiens OX=9606 GN=MYO1C PE=1 SV=4	0,220022002	0,229	1,009
Q27J81	Inverted formin-2 OS=Homo sapiens OX=9606 GN=INF2 PE=1 SV=2	0,273972603	0,227	0,862
Q16625	Occludin OS=Homo sapiens OX=9606 GN=OCLN PE=1 SV=1	0,896860987	0,224	0,25
P83111	Serine beta-lactamase-like protein LACTB, mitochondrial OS=Homo sapiens OX=9606 GN=LACTB PE=1 SV=2	0,249190132	0,224	0,83
O15533	Tapasin OS=Homo sapiens OX=9606 GN=TAPBP PE=1 SV=2	0,035568202	0,224	2,475
P21980	Protein-glutamine gamma-glutamyltransferase 2 OS=Homo sapiens OX=9606 GN=TGM2 PE=1 SV=2	0,252016129	0,222	0,884
Q9UKS6	Protein kinase C and casein kinase substrate in neurons protein 3 OS=Homo sapiens OX=9606 GN=PACSIN3 PE=1 SV=2	0,701262272	0,221	0,305
Q06323	Proteasome activator complex subunit 1 OS=Homo sapiens OX=9606 GN=PSME1 PE=1 SV=1	0,240847784	0,217	0,927
Q5JRX3	Presequence protease, mitochondrial OS=Homo sapiens OX=9606 GN=PITRM1 PE=1 SV=3	0,513347023	0,216	0,417
P16144	Integrin beta-4 OS=Homo sapiens OX=9606 GN=ITGB4 PE=1 SV=5	0,674763833	0,214	0,242
P49748	Very long-chain specific acyl-CoA dehydrogenase, mitochondrial OS=Homo sapiens OX=9606 GN=ACADVL PE=1 SV=1	0,416666667	0,214	0,528
P49790	Nuclear pore complex protein Nup153 OS=Homo sapiens OX=9606 GN=NUP153 PE=1 SV=2	0,856898029	0,213	0,251
Q96CM8	Medium-chain acyl-CoA ligase ACSF2, mitochondrial OS=Homo sapiens OX=9606 GN=ACSF2 PE=1 SV=2	0,560538117	0,213	0,43
O00592	Podocalyxin OS=Homo sapiens OX=9606 GN=PODXL PE=1 SV=2	0,19219681	0,212	1,389
Q14573	Inositol 1,4,5-trisphosphate receptor type 3 OS=Homo sapiens OX=9606 GN=ITPR3 PE=1 SV=2	0,128716695	0,212	1,61
Q16787	Laminin subunit alpha-3 OS=Homo sapiens OX=9606 GN=LAMA3 PE=1 SV=3	0,515729758	0,211	0,247
P08195	Amino acid transporter heavy chain SLC3A2 OS=Homo sapiens OX=9606 GN=SLC3A2 PE=1 SV=3	0,236574403	0,211	0,812
Q92882	Osteoclast-stimulating factor 1 OS=Homo sapiens OX=9606 GN=OSTF1 PE=1 SV=2	0,356760614	0,21	0,527
Q9BXK5	Bcl-2-like protein 13 OS=Homo sapiens OX=9606 GN=BCL2L13 PE=1 SV=1	0,155738981	0,207	1,377
Q9Y6K5	2'-5'-oligoadenylate synthase 3 OS=Homo sapiens OX=9606 GN=OAS3 PE=1 SV=3	0,159083678	0,206	1,705
Q63HN8	E3 ubiquitin-protein ligase RNF213 OS=Homo sapiens OX=9606 GN=RNF213 PE=1 SV=3	0,52882073	0,203	0,456
O00515	Ladinin-1 OS=Homo sapiens OX=9606 GN=LAD1 PE=1 SV=2	0,595592615	0,201	0,345
P29323	Ephrin type-B receptor 2 OS=Homo sapiens OX=9606 GN=EPHB2 PE=1 SV=5	0,249625562	0,2	1,436
P40121	Macrophage-capping protein OS=Homo sapiens OX=9606 GN=CAPG PE=1 SV=2	0,151653018	0,2	1,091
Q9H6F5	Coiled-coil domain-containing protein 86 OS=Homo sapiens OX=9606 GN=CCDC86 PE=1 SV=1	1,285347044	0,198	0,168
Q7Z406	Myosin-14 OS=Homo sapiens OX=9606 GN=MYH14 PE=1 SV=2	1,172332943	0,196	0,141
P13647	Keratin, type II cytoskeletal 5 OS=Homo sapiens OX=9606 GN=KRT5 PE=1 SV=3	0,458505273	0,192	0,363
O75369	Filamin-B OS=Homo sapiens OX=9606 GN=FLNB PE=1 SV=2	0,228206298	0,192	0,884
O60716	Catenin delta-1 OS=Homo sapiens OX=9606 GN=CTNND1 PE=1 SV=1	0,369959304	0,19	0,542
Q9H553	Alpha-1,3/1,6-mannosyltransferase ALG2 OS=Homo sapiens OX=9606 GN=ALG2 PE=1 SV=1	0,236910685	0,189	0,839
E9PDI4	Ladinin-1 OS=Homo sapiens OX=9606 GN=LAD1 PE=1 SV=1	0,798722045	0,188	0,256
Q6WCQ1	Myosin phosphatase Rho-interacting protein OS=Homo sapiens OX=9606 GN=MPRIP PE=1 SV=3	0,315955766	0,188	0,573
Q16762	Thiosulfate sulfurtransferase OS=Homo sapiens OX=9606 GN=TST PE=1 SV=4	0,345184674	0,186	0,506
Q8IXQ6	Protein mono-ADP-ribosyltransferase PARP9 OS=Homo sapiens OX=9606 GN=PARP9 PE=1 SV=2	0,174094708	0,186	1,099
P26006	Integrin alpha-3 OS=Homo sapiens OX=9606 GN=ITGA3 PE=1 SV=5	0,086820629	0,186	2,506
P49419	Alpha-aminoadipic semialdehyde dehydrogenase OS=Homo sapiens OX=9606 GN=ALDH7A1 PE=1 SV=5	0,800640512	0,184	0,521
Q99959	Plakophilin-2 OS=Homo sapiens OX=9606 GN=PKP2 PE=1 SV=2	0,455788514	0,184	0,392
Q9Y6N5	Sulfide:quinone oxidoreductase, mitochondrial OS=Homo sapiens OX=9606 GN=SQOR PE=1 SV=1	0,122910521	0,183	1,215
Q86SQ0	Pleckstrin homology-like domain family B member 2 OS=Homo sapiens OX=9606 GN=PHLDB2 PE=1 SV=2	1,841620626	0,181	0,108
Q7Z4W1	L-xylulose reductase OS=Homo sapiens OX=9606 GN=DCXR PE=1 SV=2	1,071811361	0,177	0,289
O95425	Supervillin OS=Homo sapiens OX=9606 GN=SVIL PE=1 SV=2	0,364963504	0,177	0,485
P28838	Cytosol aminopeptidase OS=Homo sapiens OX=9606 GN=LAP3 PE=1 SV=3	0,412541254	0,175	0,433
Q9UDY2	Tight junction protein ZO-2 OS=Homo sapiens OX=9606 GN=TJP2 PE=1 SV=2	1,047120419	0,171	0,193
P22570	NADPH:adrenodoxin oxidoreductase, mitochondrial OS=Homo sapiens OX=9606 GN=FDXR PE=1 SV=3	0,997008973	0,168	0,139
Q8IWA5	Choline transporter-like protein 2 OS=Homo sapiens OX=9606 GN=SLC44A2 PE=1 SV=3	0,109541023	0,167	1,373
P02751	Fibronectin OS=Homo sapiens OX=9606 GN=FN1 PE=1 SV=5	0,361271676	0,162	0,453
Q9ULC5	Long-chain-fatty-acid--CoA ligase 5 OS=Homo sapiens OX=9606 GN=ACSL5 PE=1 SV=1	0,058400981	0,161	3,245
P14923	Junction plakoglobin OS=Homo sapiens OX=9606 GN=JUP PE=1 SV=3	0,507356672	0,159	0,308
Q6ZRV2	Protein FAM83H OS=Homo sapiens OX=9606 GN=FAM83H PE=1 SV=3	0,992063492	0,156	0,147
P52566	Rho GDP-dissociation inhibitor 2 OS=Homo sapiens OX=9606 GN=ARHGDIB PE=1 SV=3	1,526717557	0,152	0,275
P09497	Clathrin light chain B OS=Homo sapiens OX=9606 GN=CLTB PE=1 SV=1	0,445434298	0,151	0,35
Q9UQB8	Brain-specific angiogenesis inhibitor 1-associated protein 2 OS=Homo sapiens OX=9606 GN=BAIAP2 PE=1 SV=1	0,638977636	0,15	0,2
Q8NFV4	sn-1-specific diacylglycerol lipase ABHD11 OS=Homo sapiens OX=9606 GN=ABHD11 PE=1 SV=2	0,686341798	0,148	0,266
Q86X29	Lipolysis-stimulated lipoprotein receptor OS=Homo sapiens OX=9606 GN=LSR PE=1 SV=4	1,650165017	0,141	0,075
H0YD14	Myoferlin (Fragment) OS=Homo sapiens OX=9606 GN=MYOF PE=1 SV=1	0,068064253	0,141	2,177
P29590	Protein PML OS=Homo sapiens OX=9606 GN=PML PE=1 SV=3	0,146713615	0,134	0,687
P02545	Prelamin-A/C OS=Homo sapiens OX=9606 GN=LMNA PE=1 SV=1	0,056449337	0,133	2,081
P27144	Adenylate kinase 4, mitochondrial OS=Homo sapiens OX=9606 GN=AK4 PE=1 SV=1	2,680965147	0,131	0,079
Q9NZ08	Endoplasmic reticulum aminopeptidase 1 OS=Homo sapiens OX=9606 GN=ERAP1 PE=1 SV=3	0,102606197	0,131	1,189
P35270	Sepiapterin reductase OS=Homo sapiens OX=9606 GN=SPR PE=1 SV=1	1	0,13	0,579
Q96N66	Lysophospholipid acyltransferase 7 OS=Homo sapiens OX=9606 GN=MBOAT7 PE=1 SV=2	0,256147541	0,13	0,465
P05549	Transcription factor AP-2-alpha OS=Homo sapiens OX=9606 GN=TFAP2A PE=1 SV=1	1,652892562	0,124	0,079
Q8IVL5	Prolyl 3-hydroxylase 2 OS=Homo sapiens OX=9606 GN=P3H2 PE=1 SV=1	0,070303712	0,122	3,429
Q6PIU2	Neutral cholesterol ester hydrolase 1 OS=Homo sapiens OX=9606 GN=NCEH1 PE=1 SV=3	0,142106011	0,119	0,845
P12429	Annexin A3 OS=Homo sapiens OX=9606 GN=ANXA3 PE=1 SV=3	0,20044097	0,118	0,485
P32455	Guanylate-binding protein 1 OS=Homo sapiens OX=9606 GN=GBP1 PE=1 SV=2	0,05245764	0,117	2,455
Q04912	Macrophage-stimulating protein receptor OS=Homo sapiens OX=9606 GN=MST1R PE=1 SV=3	0,61842919	0,116	0,187
Q9P206	NHS-like protein 3 OS=Homo sapiens OX=9606 GN=NHSL3 PE=1 SV=2	0,243072436	0,116	0,472
Q08380	Galectin-3-binding protein OS=Homo sapiens OX=9606 GN=LGALS3BP PE=1 SV=1	0,115340254	0,116	0,657
Q9BQE5	Apolipoprotein L2 OS=Homo sapiens OX=9606 GN=APOL2 PE=1 SV=2	0,097333074	0,116	0,78
P31947	14-3-3 protein sigma OS=Homo sapiens OX=9606 GN=SFN PE=1 SV=1	1,212121212	0,115	0,072
P17275	Transcription factor JunB OS=Homo sapiens OX=9606 GN=JUNB PE=1 SV=1	0,475963827	0,115	0,318

Q03135	Caveolin-1 OS=Homo sapiens OX=9606 GN=CAV1 PE=1 SV=4	0,038346499	0,113	2,937
P05783	Keratin, type I cytoskeletal 18 OS=Homo sapiens OX=9606 GN=KRT18 PE=1 SV=2	1,028806584	0,112	0,109
Q9Y2Q3	Glutathione S-transferase kappa 1 OS=Homo sapiens OX=9606 GN=GSTK1 PE=1 SV=3	0,12318305	0,106	1,14
Q01650	Large neutral amino acids transporter small subunit 1 OS=Homo sapiens OX=9606 GN=SLC7A5 PE=1 SV=2	0,175901495	0,104	0,529
P57735	Ras-related protein Rab-25 OS=Homo sapiens OX=9606 GN=RAB25 PE=1 SV=2	1000	0,103	0,001
Q03518	Antigen peptide transporter 1 OS=Homo sapiens OX=9606 GN=TAP1 PE=1 SV=3	0,035069262	0,102	1,497
P04181	Ornithine aminotransferase, mitochondrial OS=Homo sapiens OX=9606 GN=OAT PE=1 SV=1	1,908396947	0,099	0,045
P29728	2'-5'-oligoadenylate synthase 2 OS=Homo sapiens OX=9606 GN=OAS2 PE=1 SV=3	0,090122567	0,099	1,319
Q9NZM1	Myoferlin OS=Homo sapiens OX=9606 GN=MYOF PE=1 SV=1	0,038904451	0,097	2,364
Q13740	CD166 antigen OS=Homo sapiens OX=9606 GN=ALCAM PE=1 SV=2	0,228675966	0,095	0,367
Q6NZI2	Caveolae-associated protein 1 OS=Homo sapiens OX=9606 GN=CAVIN1 PE=1 SV=1	0,029273148	0,095	2,681
P09668	Pro-cathepsin H OS=Homo sapiens OX=9606 GN=CTSH PE=1 SV=4	0,67294751	0,092	0,204
P00966	Argininosuccinate synthase OS=Homo sapiens OX=9606 GN=ASS1 PE=1 SV=2	0,142795945	0,09	0,582
Q9Y446	Plakophilin-3 OS=Homo sapiens OX=9606 GN=PKP3 PE=1 SV=1		0,089	0,088
Q6YHK3	CD109 antigen OS=Homo sapiens OX=9606 GN=CD109 PE=1 SV=2	0,138734739	0,087	1,514
P19224	UDP-glucuronosyltransferase 1-6 OS=Homo sapiens OX=9606 GN=UGT1A6 PE=1 SV=2	0,372856078	0,085	1,574
P36952	Serpin B5 OS=Homo sapiens OX=9606 GN=SERPINB5 PE=1 SV=2	0,437445319	0,083	0,262
P16070	CD44 antigen OS=Homo sapiens OX=9606 GN=CD44 PE=1 SV=3	0,017118598	0,083	3,779
Q9Y624	Junctional adhesion molecule A OS=Homo sapiens OX=9606 GN=F11R PE=1 SV=1	0,283607487	0,082	0,17
Q9UGT4	Sushi domain-containing protein 2 OS=Homo sapiens OX=9606 GN=SUSD2 PE=1 SV=1		0,081	0,19
Q9BYK8	3'-5' exoribonuclease HELZ2 OS=Homo sapiens OX=9606 GN=HELZ2 PE=1 SV=7	0,079725743	0,079	0,842
P05787	Keratin, type II cytoskeletal 8 OS=Homo sapiens OX=9606 GN=KRT8 PE=1 SV=7	1,543209877	0,075	0,052
Q5K651	Sterile alpha motif domain-containing protein 9 OS=Homo sapiens OX=9606 GN=SAMD9 PE=1 SV=1	0,340831629	0,075	0,669
P28065	Proteasome subunit beta type-9 OS=Homo sapiens OX=9606 GN=PSMB9 PE=1 SV=2	0,019145718	0,075	3,421
P32004	Neural cell adhesion molecule L1 OS=Homo sapiens OX=9606 GN=L1CAM PE=1 SV=2	1,331557923	0,074	0,059
P04439	HLA class I histocompatibility antigen, A alpha chain OS=Homo sapiens OX=9606 GN=HLA-A PE=1 SV=2	0,074183976	0,073	0,895
Q03519	Antigen peptide transporter 2 OS=Homo sapiens OX=9606 GN=TAP2 PE=1 SV=1	0,061020259	0,073	1,42
P04637	Cellular tumor antigen p53 OS=Homo sapiens OX=9606 GN=TP53 PE=1 SV=4	0,859845228	0,072	0,083
Q9ULZ3	Apoptosis-associated speck-like protein containing a CARD OS=Homo sapiens OX=9606 GN=PYCARD PE=1 SV=2	0,251130085	0,072	0,442
P05362	Intercellular adhesion molecule 1 OS=Homo sapiens OX=9606 GN=ICAM1 PE=1 SV=2	0,079700327	0,071	0,917
Q9UHQ9	NADH-cytochrome b5 reductase 1 OS=Homo sapiens OX=9606 GN=CYB5R1 PE=1 SV=1	0,404694456	0,07	0,204
P22223	Cadherin-3 OS=Homo sapiens OX=9606 GN=CDH3 PE=1 SV=2	0,208029956	0,051	0,123
P17301	Integrin alpha-2 OS=Homo sapiens OX=9606 GN=ITGA2 PE=1 SV=2	0,03623451	0,041	1,909
P04233	HLA class II histocompatibility antigen gamma chain OS=Homo sapiens OX=9606 GN=CD74 PE=1 SV=3	0,406173842	0,039	0,12
Q16647	Prostacyclin synthase OS=Homo sapiens OX=9606 GN=PTGIS PE=1 SV=1	1,834862385	0,037	0,224
O15427	Monocarboxylate transporter 4 OS=Homo sapiens OX=9606 GN=SLC16A3 PE=1 SV=1	0,016567263	0,036	2,028
P12830	Cadherin-1 OS=Homo sapiens OX=9606 GN=CDH1 PE=1 SV=3	0,901713255	0,03	0,032
O15382	Branched-chain-amino-acid aminotransferase, mitochondrial OS=Homo sapiens OX=9606 GN=BCAT2 PE=1 SV=2	0,109217999	0,029	1,122
P50895	Basal cell adhesion molecule OS=Homo sapiens OX=9606 GN=BCAM PE=1 SV=2	0,244140625	0,026	0,1
O43278	Kunitz-type protease inhibitor 1 OS=Homo sapiens OX=9606 GN=SPINT1 PE=1 SV=2	0,385208012	0,025	0,303
Q04695	Keratin, type I cytoskeletal 17 OS=Homo sapiens OX=9606 GN=KRT17 PE=1 SV=2	0,271223217	0,021	0,049
P08727	Keratin, type I cytoskeletal 19 OS=Homo sapiens OX=9606 GN=KRT19 PE=1 SV=4	0,394321767	0,019	0,022
P09758	Tumor-associated calcium signal transducer 2 OS=Homo sapiens OX=9606 GN=TACSTD2 PE=1 SV=3	0,292483182	0,019	0,024
P47895	Retinaldehyde dehydrogenase 3 OS=Homo sapiens OX=9606 GN=ALDH1A3 PE=1 SV=2	0,534473544	0,017	0,037
Q9BV40	Vesicle-associated membrane protein 8 OS=Homo sapiens OX=9606 GN=VAMP8 PE=1 SV=1	0,256147541	0,014	0,048
P08729	Keratin, type II cytoskeletal 7 OS=Homo sapiens OX=9606 GN=KRT7 PE=1 SV=5	0,688705234	0,004	0,006
Q15833	Syntaxin-binding protein 2 OS=Homo sapiens OX=9606 GN=STXBP2 PE=1 SV=2	0,001	0,001	1,238
Q96Q06	Perilipin-4 OS=Homo sapiens OX=9606 GN=PLIN4 PE=1 SV=3	0,001	0,001	1,22
P80217	Interferon-induced 35 kDa protein OS=Homo sapiens OX=9606 GN=IFI35 PE=1 SV=5	0,001	0,001	0,836
Q8N335	Glycerol-3-phosphate dehydrogenase 1-like protein OS=Homo sapiens OX=9606 GN=GPD1L PE=1 SV=1	0,001	0,001	
P00450	Ceruloplasmin OS=Homo sapiens OX=9606 GN=CP PE=1 SV=2	0,001	0,001	
P27487	Dipeptidyl peptidase 4 OS=Homo sapiens OX=9606 GN=DPP4 PE=1 SV=2	0,001	0,001	9,904
O15438	ATP-binding cassette sub-family C member 3 OS=Homo sapiens OX=9606 GN=ABCC3 PE=1 SV=3	0,001	0,001	3,606
Q9BRQ8	Ferroptosis suppressor protein 1 OS=Homo sapiens OX=9606 GN=AIFM2 PE=1 SV=1	0,001	0,001	3,285
Q16719	Kynureninase OS=Homo sapiens OX=9606 GN=KYNU PE=1 SV=1	0,001	0,001	2,661
P16219	Short-chain specific acyl-CoA dehydrogenase, mitochondrial OS=Homo sapiens OX=9606 GN=ACADS PE=1 SV=1	0,001	0,001	0,473
O95833	Chloride intracellular channel protein 3 OS=Homo sapiens OX=9606 GN=CLIC3 PE=1 SV=2	0,001	0,001	0,305
Q9H6S3	Epidermal growth factor receptor kinase substrate 8-like protein 2 OS=Homo sapiens OX=9606 GN=EPS8L2 PE=1 SV=2	0,001	0,001	0,16
P13646	Keratin, type I cytoskeletal 13 OS=Homo sapiens OX=9606 GN=KRT13 PE=1 SV=4	0,001	0,001	0,034
O15231	Zinc finger protein 185 OS=Homo sapiens OX=9606 GN=ZNF185 PE=1 SV=3		0,001	0,001
Q8WWA1	Transmembrane protein 40 OS=Homo sapiens OX=9606 GN=TMEM40 PE=1 SV=2		0,001	0,001
Q9C002	Normal mucosa of esophagus-specific gene 1 protein OS=Homo sapiens OX=9606 GN=NMES1 PE=1 SV=1		0,001	0,001
O60603	Toll-like receptor 2 OS=Homo sapiens OX=9606 GN=TLR2 PE=1 SV=1		0,001	0,001

	DOWNREGULATED
	UPREGULATED
	NOT CHANGED

Regulated proteins identified with unique peptide number equal to or more than 5

Accession	Description	Abundance Ratio: (HTB-5) / (HTB-5 PR)	Abundance Ratio: (HTB-5 PR) / (SV-HUC-1)	Abundance Ratio: (HTB-5) / (SV-HUC-1)
P07476	Involucrin OS=Homo sapiens OX=9606 GN=IVL PE=1 SV=2		0,636	
Q8N335	Glycerol-3-phosphate dehydrogenase 1-like protein OS=Homo sapiens OX=9606 GN=GPD1L PE=1 SV=1	1000	0,001	
P00450	Ceruloplasmin OS=Homo sapiens OX=9606 GN=CP PE=1 SV=2	1000	0,001	
O75363	Breast carcinoma-amplified sequence 1 OS=Homo sapiens OX=9606 GN=BCAS1 PE=1 SV=2	0,001	1000	
P07196	Neurofilament light polypeptide OS=Homo sapiens OX=9606 GN=NEFL PE=1 SV=3	0,127	180,587	
Q15813	Tubulin-specific chaperone E OS=Homo sapiens OX=9606 GN=TBCE PE=1 SV=1	0,373	3,654	
Q9UDR5	Alpha-aminoadipic semialdehyde synthase, mitochondrial OS=Homo sapiens OX=9606 GN=AASS PE=1 SV=1		1000	1000
P26022	Pentraxin-related protein PTX3 OS=Homo sapiens OX=9606 GN=PTX3 PE=1 SV=3	3,168	1000	1000
Q99985	Semaphorin-3C OS=Homo sapiens OX=9606 GN=SEMA3C PE=2 SV=2	2,819	1000	1000
P51884	Lumican OS=Homo sapiens OX=9606 GN=LUM PE=1 SV=2	1000		1000
P52895	Aldo-keto reductase family 1 member C2 OS=Homo sapiens OX=9606 GN=AKR1C2 PE=1 SV=3	1,722	1000	1000
P15428	15-hydroxyprostaglandin dehydrogenase [NAD(+)] OS=Homo sapiens OX=9606 GN=HPGD PE=1 SV=1	1,039	1000	1000
P54868	Hydroxymethylglutaryl-CoA synthase, mitochondrial OS=Homo sapiens OX=9606 GN=HMGCS2 PE=1 SV=1	1,027	1000	1000
Q10472	Polypeptide N-acetylgalactosaminyltransferase 1 OS=Homo sapiens OX=9606 GN=GALNT1 PE=1 SV=1	0,935	1000	1000
Q92673	Sortilin-related receptor OS=Homo sapiens OX=9606 GN=SORL1 PE=1 SV=2	0,785	1000	1000
O60245	Protocadherin-7 OS=Homo sapiens OX=9606 GN=PCDH7 PE=1 SV=2	0,4	1000	1000
Q9BPU6	Dihydropyrimidinase-related protein 5 OS=Homo sapiens OX=9606 GN=DPYSL5 PE=1 SV=1	0,025	1000	1000
P16671	Platelet glycoprotein 4 OS=Homo sapiens OX=9606 GN=CD36 PE=1 SV=2	53,466	1,445	188,864
P08133	Annexin A6 OS=Homo sapiens OX=9606 GN=ANXA6 PE=1 SV=3	0,67	63,943	45,162
P08648	Integrin alpha-5 OS=Homo sapiens OX=9606 GN=ITGA5 PE=1 SV=2	11,868	2,979	44,474
P23219	Prostaglandin G/H synthase 1 OS=Homo sapiens OX=9606 GN=PTGS1 PE=1 SV=2	27,162	1,863	32,559
Q8NBQ5	Estradiol 17-beta-dehydrogenase 11 OS=Homo sapiens OX=9606 GN=HSD17B11 PE=1 SV=4	1,758	14,022	28,187
P06703	Protein S100-A6 OS=Homo sapiens OX=9606 GN=S100A6 PE=1 SV=1	12,677	2,181	27,387
O95340	Bifunctional 3'-phosphoadenosine 5'-phosphosulfate synthase 2 OS=Homo sapiens OX=9606 GN=PAPSS2 PE=1 SV=2	4,006	5,778	27,152
P12277	Creatine kinase B-type OS=Homo sapiens OX=9606 GN=CKB PE=1 SV=1	0,229	104,267	26,258
P02461	Collagen alpha-1(III) chain OS=Homo sapiens OX=9606 GN=COL3A1 PE=1 SV=4	12,524	1,158	25,349
P98082	Disabled homolog 2 OS=Homo sapiens OX=9606 GN=DAB2 PE=1 SV=3	3,788	14,418	21,621
P07099	Epoxide hydrolase 1 OS=Homo sapiens OX=9606 GN=EPHX1 PE=1 SV=1	14,594	1,285	20,891
Q9NZN4	EH domain-containing protein 2 OS=Homo sapiens OX=9606 GN=EHD2 PE=1 SV=2	25,512	0,537	20,75
P08473	Neprilysin OS=Homo sapiens OX=9606 GN=MME PE=1 SV=2	6,421	5,829	17,823
P09467	Fructose-1,6-bisphosphatase 1 OS=Homo sapiens OX=9606 GN=FBP1 PE=1 SV=5	2,242	4,091	17,006
O75368	Adapter SH3BGRL OS=Homo sapiens OX=9606 GN=SH3BGRL PE=1 SV=1	0,723	23,356	16,746
Q14195	Dihydropyrimidinase-related protein 3 OS=Homo sapiens OX=9606 GN=DPYSL3 PE=1 SV=1	4,356	4,095	16,599
P35052	Glypican-1 OS=Homo sapiens OX=9606 GN=GPC1 PE=1 SV=2	6,776	2,642	16,466
Q14194	Dihydropyrimidinase-related protein 1 OS=Homo sapiens OX=9606 GN=CRMP1 PE=1 SV=1	0,568	14,76	16,085
A0AAG2U\	Collagen type III alpha 1 chain OS=Homo sapiens OX=9606 GN=COL3A1 PE=1 SV=1	9,677	0,884	15,686
O76070	Gamma-synuclein OS=Homo sapiens OX=9606 GN=SNCG PE=1 SV=2	16,882	1,286	14,244
Q99715	Collagen alpha-1(XII) chain OS=Homo sapiens OX=9606 GN=COL12A1 PE=1 SV=2	12,761	1,228	13,887
P02452	Collagen alpha-1(I) chain OS=Homo sapiens OX=9606 GN=COL1A1 PE=1 SV=6	20,744	0,679	12,747
P08123	Collagen alpha-2(I) chain OS=Homo sapiens OX=9606 GN=COL1A2 PE=1 SV=7	10,135	0,869	12,42
O14786	Neuropilin-1 OS=Homo sapiens OX=9606 GN=NRP1 PE=1 SV=3	14,65	0,796	12,338
P08779	Keratin, type I cytoskeletal 16 OS=Homo sapiens OX=9606 GN=KRT16 PE=1 SV=4	12,439	1,003	12,011
P62736	Actin, aortic smooth muscle OS=Homo sapiens OX=9606 GN=ACTA2 PE=1 SV=1	3,388	3,025	11,967
P19022	Cadherin-2 OS=Homo sapiens OX=9606 GN=CDH2 PE=1 SV=4	2,634	2,363	11,598
Q14554	Protein disulfide-isomerase A5 OS=Homo sapiens OX=9606 GN=PDIA5 PE=1 SV=1	1,816	6,327	11,155
P43121	Cell surface glycoprotein MUC18 OS=Homo sapiens OX=9606 GN=MCAM PE=1 SV=2	12,418	1,162	11,131
P23634	Plasma membrane calcium-transporting ATPase 4 OS=Homo sapiens OX=9606 GN=ATP2B4 PE=1 SV=2	4,119	2,68	10,937
Q8IVF2	Protein AHNAK2 OS=Homo sapiens OX=9606 GN=AHNAK2 PE=1 SV=2	12,164	0,825	10,633
Q16881	Thioredoxin reductase 1, cytoplasmic OS=Homo sapiens OX=9606 GN=TXNRD1 PE=1 SV=3	1,442	8,604	10,196
Q05682	Caldesmon OS=Homo sapiens OX=9606 GN=CALD1 PE=1 SV=3	4,576	2,396	10,126
P27487	Dipeptidyl peptidase 4 OS=Homo sapiens OX=9606 GN=DPP4 PE=1 SV=2	1000	0,001	9,904
P00352	Aldehyde dehydrogenase 1A1 OS=Homo sapiens OX=9606 GN=ALDH1A1 PE=1 SV=2	12,07	1,531	9,602
P23141	Liver carboxylesterase 1 OS=Homo sapiens OX=9606 GN=CES1 PE=1 SV=2	10,603	0,497	9,496
Q01995	Transgelin OS=Homo sapiens OX=9606 GN=TAGLN PE=1 SV=4	5,218	1,79	8,959
Q9UKX5	Integrin alpha-11 OS=Homo sapiens OX=9606 GN=ITGA11 PE=1 SV=2	7,375		8,385
O75891	Cytosolic 10-formyltetrahydrofolate dehydrogenase OS=Homo sapiens OX=9606 GN=ALDH1L1 PE=1 SV=2	1,305	6,744	8,354
Q07954	Prolow-density lipoprotein receptor-related protein 1 OS=Homo sapiens OX=9606 GN=LRP1 PE=1 SV=2	2,553	3,941	8,319
P36551	Oxygen-dependent coproporphyrinogen-III oxidase, mitochondrial OS=Homo sapiens OX=9606 GN=CPOX PE=1 SV=3	10,064	0,861	8,163
Q16658	Fascin OS=Homo sapiens OX=9606 GN=FSCN1 PE=1 SV=3	0,38	20,954	8,108
P39060	Collagen alpha-1(XVIII) chain OS=Homo sapiens OX=9606 GN=COL18A1 PE=1 SV=5	4,957	1,867	7,517
P09601	Heme oxygenase 1 OS=Homo sapiens OX=9606 GN=HMOX1 PE=1 SV=1	12,767	0,618	7,406
O95865	Putative hydrolase DDAH2 OS=Homo sapiens OX=9606 GN=DDAH2 PE=1 SV=1	0,291	25,508	7,11
P80303	Nucleobindin-2 OS=Homo sapiens OX=9606 GN=NUCB2 PE=1 SV=3	2,727	2,372	7,102
Q9Y639	Neuroplastin OS=Homo sapiens OX=9606 GN=NPTN PE=1 SV=2	4,85	1,421	6,857
Q32P28	Prolyl 3-hydroxylase 1 OS=Homo sapiens OX=9606 GN=P3H1 PE=1 SV=2	1,395	5,484	6,743
P46821	Microtubule-associated protein 1B OS=Homo sapiens OX=9606 GN=MAP1B PE=1 SV=2	0,255	17,908	6,686
Q07065	Cytoskeleton-associated protein 4 OS=Homo sapiens OX=9606 GN=CKAP4 PE=1 SV=2	2,13	3,353	6,667
O14975	Long-chain fatty acid transport protein 2 OS=Homo sapiens OX=9606 GN=SLC27A2 PE=1 SV=2	5,824	0,618	6,521
P04406	Glyceraldehyde-3-phosphate dehydrogenase OS=Homo sapiens OX=9606 GN=GAPDH PE=1 SV=3	0,878	5,057	6,508
Q15758	Neutral amino acid transporter B(0) OS=Homo sapiens OX=9606 GN=SLC1A5 PE=1 SV=2	7,18	0,879	6,469
P21266	Glutathione S-transferase Mu 3 OS=Homo sapiens OX=9606 GN=GSTM3 PE=1 SV=3	0,57	10,182	6,463
Q969H8	Myeloid-derived growth factor OS=Homo sapiens OX=9606 GN=MYDGF PE=1 SV=1	2,755	2,222	6,364
Q16890	Tumor protein D53 OS=Homo sapiens OX=9606 GN=TPD52L1 PE=1 SV=1	3,282	1,661	6,29
Q5VV42	Threonylcarbamoyladenosine tRNA methylthiotransferase OS=Homo sapiens OX=9606 GN=CDKAL1 PE=1 SV=1	1,094	8,077	6,282
P43007	Neutral amino acid transporter A OS=Homo sapiens OX=9606 GN=SLC1A4 PE=1 SV=1	4,636	0,594	6,276
P20020	Plasma membrane calcium-transporting ATPase 1 OS=Homo sapiens OX=9606 GN=ATP2B1 PE=1 SV=4	1,539	3,758	6,154
P15090	Fatty acid-binding protein, adipocyte OS=Homo sapiens OX=9606 GN=FABP4 PE=1 SV=3	1,256	8,735	6,075
P11717	Cation-independent mannose-6-phosphate receptor OS=Homo sapiens OX=9606 GN=IGF2R PE=1 SV=3	1,506	3,781	6,052
Q6DKJ4	Nucleoredoxin OS=Homo sapiens OX=9606 GN=NXN PE=1 SV=2	0,379	15,184	5,997

P07996	Thrombospondin-1 OS=Homo sapiens OX=9606 GN=THBS1 PE=1 SV=2	6,014	0,883	5,9
Q16555	Dihydropyrimidinase-related protein 2 OS=Homo sapiens OX=9606 GN=DPYSL2 PE=1 SV=1	0,37	17,36	5,877
P02533	Keratin, type I cytoskeletal 14 OS=Homo sapiens OX=9606 GN=KRT14 PE=1 SV=4	4,489	1,566	5,769
Q13418	Integrin-linked protein kinase OS=Homo sapiens OX=9606 GN=ILK PE=1 SV=2	1,589	3,425	5,766
O75718	Cartilage-associated protein OS=Homo sapiens OX=9606 GN=CRTAP PE=1 SV=1	1,988	2,177	5,752
Q15646	2'-5'-oligoadenylate synthase-like protein OS=Homo sapiens OX=9606 GN=OASL PE=1 SV=2	10,758	0,466	5,752
A0A1C7CY	Dihydropyrimidinase-related protein 2 OS=Homo sapiens OX=9606 GN=DPYSL2 PE=1 SV=1	0,316	19,439	5,612
P09913	Interferon-induced protein with tetratricopeptide repeats 2 OS=Homo sapiens OX=9606 GN=IFIT2 PE=1 SV=1	5,636	0,646	5,547
Q63ZY3	KN motif and ankyrin repeat domain-containing protein 2 OS=Homo sapiens OX=9606 GN=KANK2 PE=1 SV=1	3,619	1,5	5,435
Q14764	Major vault protein OS=Homo sapiens OX=9606 GN=MVP PE=1 SV=4	7,786	0,291	5,413
Q96JJ7	Protein disulfide-isomerase TMX3 OS=Homo sapiens OX=9606 GN=TMX3 PE=1 SV=2	3,569	1,389	5,354
Q9UNF1	Melanoma-associated antigen D2 OS=Homo sapiens OX=9606 GN=MAGED2 PE=1 SV=2	0,241	17,202	5,229
Q6UVK1	Chondroitin sulfate proteoglycan 4 OS=Homo sapiens OX=9606 GN=CSPG4 PE=1 SV=2	5,202	1,123	5,155
O75962	Triple functional domain protein OS=Homo sapiens OX=9606 GN=TRIO PE=1 SV=2	1,913	2,653	5,152
P50454	Serpin H1 OS=Homo sapiens OX=9606 GN=SERPINH1 PE=1 SV=2	3,257	1,644	5,12
P37840	Alpha-synuclein OS=Homo sapiens OX=9606 GN=SNCA PE=1 SV=1	6,154	1,296	5,081
Q8IVL6	Prolyl 3-hydroxylase 3 OS=Homo sapiens OX=9606 GN=P3H3 PE=1 SV=1	2,208	5,284	4,932
Q7Z4H8	Protein O-glucosyltransferase 3 OS=Homo sapiens OX=9606 GN=POGLUT3 PE=1 SV=2	2,038	2,138	4,817
Q9Y490	Talin-1 OS=Homo sapiens OX=9606 GN=TLN1 PE=1 SV=3	3,157	1,57	4,806
Q13509	Tubulin beta-3 chain OS=Homo sapiens OX=9606 GN=TUBB3 PE=1 SV=2	0,157	28,988	4,788
P50281	Matrix metalloproteinase-14 OS=Homo sapiens OX=9606 GN=MMP14 PE=1 SV=3	2,546	2,091	4,725
Q9Y625	Glypican-6 OS=Homo sapiens OX=9606 GN=GPC6 PE=1 SV=1	1,855	2,285	4,722
P11413	Glucose-6-phosphate 1-dehydrogenase OS=Homo sapiens OX=9606 GN=G6PD PE=1 SV=4	1,823	3,141	4,715
P37059	17-beta-hydroxysteroid dehydrogenase type 2 OS=Homo sapiens OX=9606 GN=HSD17B2 PE=1 SV=1	1,034	1,202	4,71
Q9ULC3	Ras-related protein Rab-23 OS=Homo sapiens OX=9606 GN=RAB23 PE=1 SV=1	3,797	1,46	4,669
P09936	Ubiquitin carboxyl-terminal hydrolase isozyme L1 OS=Homo sapiens OX=9606 GN=UCHL1 PE=1 SV=2	0,458	12,322	4,662
Q8N0X7	Spartin OS=Homo sapiens OX=9606 GN=SPART PE=1 SV=1	1,42	3,01	4,641
Q9UHK6	Alpha-methylacyl-CoA racemase OS=Homo sapiens OX=9606 GN=AMACR PE=1 SV=2	1,494	0,865	4,627
P50416	Carnitine O-palmitoyltransferase 1, liver isoform OS=Homo sapiens OX=9606 GN=CPT1A PE=1 SV=2	6,22	0,881	4,582
P07437	Tubulin beta chain OS=Homo sapiens OX=9606 GN=TUBB PE=1 SV=2	0,296	15,06	4,527
P37235	Hippocalcin-like protein 1 OS=Homo sapiens OX=9606 GN=HPCAL1 PE=1 SV=3	6,634	0,713	4,521
Q96AY3	Peptidyl-prolyl cis-trans isomerase FKBP10 OS=Homo sapiens OX=9606 GN=FKBP10 PE=1 SV=1	2,046	2,363	4,491
Q15417	Calponin-3 OS=Homo sapiens OX=9606 GN=CNN3 PE=1 SV=1	0,372	10,989	4,484
O43852	Calumenin OS=Homo sapiens OX=9606 GN=CALU PE=1 SV=2	2,119	2,193	4,432
O00299	Chloride intracellular channel protein 1 OS=Homo sapiens OX=9606 GN=CLIC1 PE=1 SV=4	0,843	5,251	4,378
P69905	Hemoglobin subunit alpha OS=Homo sapiens OX=9606 GN=HBA1 PE=1 SV=2	4,909	1,083	4,375
P15586	N-acetylglucosamine-6-sulfatase OS=Homo sapiens OX=9606 GN=GNS PE=1 SV=3	0,813	5,593	4,344
P21589	5'-nucleotidase OS=Homo sapiens OX=9606 GN=NT5E PE=1 SV=1	16,121	0,266	4,339
P09493	Tropomyosin alpha-1 chain OS=Homo sapiens OX=9606 GN=TPM1 PE=1 SV=2	3,664	1,45	4,302
O60664	Perilipin-3 OS=Homo sapiens OX=9606 GN=PLIN3 PE=1 SV=3	2,224	1,823	4,248
P17931	Galectin-3 OS=Homo sapiens OX=9606 GN=LGALS3 PE=1 SV=5	10,68	0,392	4,218
Q93052	Lipoma-preferred partner OS=Homo sapiens OX=9606 GN=LPP PE=1 SV=1	1,878	2,441	4,195
O00469	Procollagen-lysine,2-oxoglutarate 5-dioxygenase 2 OS=Homo sapiens OX=9606 GN=PLOD2 PE=1 SV=2	9,396	0,514	4,183
Q13308	Inactive tyrosine-protein kinase 7 OS=Homo sapiens OX=9606 GN=PTK7 PE=1 SV=2	1,478	2,497	4,082
P21333	Filamin-A OS=Homo sapiens OX=9606 GN=FLNA PE=1 SV=4	1,593	2,822	4,008
P14618	Pyruvate kinase PKM OS=Homo sapiens OX=9606 GN=PKM PE=1 SV=4	2,435	1,672	3,97
Q96AC1	Fermitin family homolog 2 OS=Homo sapiens OX=9606 GN=FERMT2 PE=1 SV=1	1,102	3,649	3,928
P18206	Vinculin OS=Homo sapiens OX=9606 GN=VCL PE=1 SV=4	3,418	1,196	3,911
O94925	Glutaminase kidney isoform, mitochondrial OS=Homo sapiens OX=9606 GN=GLS PE=1 SV=1	3,367	1,195	3,91
P09525	Annexin A4 OS=Homo sapiens OX=9606 GN=ANXA4 PE=1 SV=4	6,688	0,569	3,894
O43847	Nardilysin OS=Homo sapiens OX=9606 GN=NRDC PE=1 SV=3	0,555	5,424	3,824
P26640	Valine--tRNA ligase OS=Homo sapiens OX=9606 GN=VARS1 PE=1 SV=4	0,439	8,117	3,819
O60218	Aldo-keto reductase family 1 member B10 OS=Homo sapiens OX=9606 GN=AKR1B10 PE=1 SV=2	10,329	0,454	3,814
P09104	Gamma-enolase OS=Homo sapiens OX=9606 GN=ENO2 PE=1 SV=3	0,806	11,044	3,809
P16070	CD44 antigen OS=Homo sapiens OX=9606 GN=CD44 PE=1 SV=3	58,416	0,083	3,779
P61163	Alpha-centractin OS=Homo sapiens OX=9606 GN=ACTR1A PE=1 SV=1	0,924	3,9	3,765
Q09666	Neuroblast differentiation-associated protein AHNAK OS=Homo sapiens OX=9606 GN=AHNAK PE=1 SV=2	7,13	0,555	3,765
P30041	Peroxiredoxin-6 OS=Homo sapiens OX=9606 GN=PRDX6 PE=1 SV=3	2,286	1,514	3,746
Q6NYC8	Phostensin OS=Homo sapiens OX=9606 GN=PPP1R18 PE=1 SV=1	2,311	1,607	3,737
O15067	Phosphoribosylformylglycinamidine synthase OS=Homo sapiens OX=9606 GN=PFAS PE=1 SV=4	0,311	14,321	3,725
Q8TD43	Transient receptor potential cation channel subfamily M member 4 OS=Homo sapiens OX=9606 GN=TRPM4 PE=1 SV=1	2,207	1,607	3,719
Q13620	Cullin-4B OS=Homo sapiens OX=9606 GN=CUL4B PE=1 SV=4	2,412	1,674	3,702
O95870	Phosphatidylserine lipase ABHD16A OS=Homo sapiens OX=9606 GN=ABHD16A PE=1 SV=3	1,22	3,025	3,684
O75955	Flotillin-1 OS=Homo sapiens OX=9606 GN=FLOT1 PE=1 SV=3	1,441	2,361	3,647
Q16706	Alpha-mannosidase 2 OS=Homo sapiens OX=9606 GN=MAN2A1 PE=1 SV=2	3,948	0,887	3,625
Q70UQ0	Inhibitor of nuclear factor kappa-B kinase-interacting protein OS=Homo sapiens OX=9606 GN=IKBIP PE=1 SV=1	4,296	0,831	3,616
Q15942	Zyxin OS=Homo sapiens OX=9606 GN=ZYX PE=1 SV=1	2,317	1,312	3,611
O15438	ATP-binding cassette sub-family C member 3 OS=Homo sapiens OX=9606 GN=ABCC3 PE=1 SV=3	1000	0,001	3,606
P07108	Acyl-CoA-binding protein OS=Homo sapiens OX=9606 GN=DBI PE=1 SV=2	0,519	4,073	3,601
P80723	Brain acid soluble protein 1 OS=Homo sapiens OX=9606 GN=BASP1 PE=1 SV=2	1,788	2,223	3,586
P11802	Cyclin-dependent kinase 4 OS=Homo sapiens OX=9606 GN=CDK4 PE=1 SV=2	0,371	7,389	3,576
P05556	Integrin beta-1 OS=Homo sapiens OX=9606 GN=ITGB1 PE=1 SV=2	6,891	0,512	3,571
Q86TX2	Acyl-coenzyme A thioesterase 1 OS=Homo sapiens OX=9606 GN=ACOT1 PE=1 SV=1	2,429	1,424	3,558
P30101	Protein disulfide-isomerase A3 OS=Homo sapiens OX=9606 GN=PDIA3 PE=1 SV=4	1,377	2,39	3,539
Q8N8S7	Protein enabled homolog OS=Homo sapiens OX=9606 GN=ENAH PE=1 SV=2	1,078	3,158	3,464
Q9HB40	Retinoid-inducible serine carboxypeptidase OS=Homo sapiens OX=9606 GN=SCPEP1 PE=1 SV=1	1,512	2,106	3,435
P14735	Insulin-degrading enzyme OS=Homo sapiens OX=9606 GN=IDE PE=1 SV=4	1,009	3,651	3,431
Q8IVL5	Prolyl 3-hydroxylase 2 OS=Homo sapiens OX=9606 GN=P3H2 PE=1 SV=1	14,224	0,122	3,429
P20591	Interferon-induced GTP-binding protein Mx1 OS=Homo sapiens OX=9606 GN=MX1 PE=1 SV=4	10,262	0,337	3,424
P28065	Proteasome subunit beta type-9 OS=Homo sapiens OX=9606 GN=PSMB9 PE=1 SV=2	52,231	0,075	3,421
P07195	L-lactate dehydrogenase B chain OS=Homo sapiens OX=9606 GN=LDHB PE=1 SV=2	0,303	9,268	3,396
P06753	Tropomyosin alpha-3 chain OS=Homo sapiens OX=9606 GN=TPM3 PE=1 SV=2	2,401	1,306	3,394

Q9UIQ6	Leucyl-cystinyl aminopeptidase OS=Homo sapiens OX=9606 GN=LNPEP PE=1 SV=3	8,079	0,496	3,393
P18085	ADP-ribosylation factor 4 OS=Homo sapiens OX=9606 GN=ARF4 PE=1 SV=3	1,438	1,767	3,387
Q15165	Serum paraoxonase/arylesterase 2 OS=Homo sapiens OX=9606 GN=PON2 PE=1 SV=4	1,551	2,177	3,384
Q9HAU0	Pleckstrin homology domain-containing family A member 5 OS=Homo sapiens OX=9606 GN=PLEKHA5 PE=1 SV=1	2,073	1,757	3,345
Q9Y617	Phosphoserine aminotransferase OS=Homo sapiens OX=9606 GN=PSAT1 PE=1 SV=2	1,062	3,486	3,336
P13674	Prolyl 4-hydroxylase subunit alpha-1 OS=Homo sapiens OX=9606 GN=P4HA1 PE=1 SV=2	2,452	1,419	3,332
Q99439	Calponin-2 OS=Homo sapiens OX=9606 GN=CNN2 PE=1 SV=4	1,716	1,877	3,33
O60488	Long-chain-fatty-acid--CoA ligase 4 OS=Homo sapiens OX=9606 GN=ACSL4 PE=1 SV=2	2,475	1,428	3,315
Q9BRQ8	Ferroptosis suppressor protein 1 OS=Homo sapiens OX=9606 GN=AIFM2 PE=1 SV=1	1000	0,001	3,285
P60660	Myosin light polypeptide 6 OS=Homo sapiens OX=9606 GN=MYL6 PE=1 SV=2	3,548	1,238	3,269
O60271	C-Jun-amino-terminal kinase-interacting protein 4 OS=Homo sapiens OX=9606 GN=SPAG9 PE=1 SV=4	1,397	2,357	3,267
Q9BZF1	Oxysterol-binding protein-related protein 8 OS=Homo sapiens OX=9606 GN=OSBPL8 PE=1 SV=3	0,866	3,582	3,257
Q9ULC5	Long-chain-fatty-acid--CoA ligase 5 OS=Homo sapiens OX=9606 GN=ACSL5 PE=1 SV=1	17,123	0,161	3,245
O15460	Prolyl 4-hydroxylase subunit alpha-2 OS=Homo sapiens OX=9606 GN=P4HA2 PE=1 SV=1	6,156	0,546	3,243
P15559	NAD(P)H dehydrogenase [quinone] 1 OS=Homo sapiens OX=9606 GN=NQO1 PE=1 SV=1	2,385	1,283	3,233
O43175	D-3-phosphoglycerate dehydrogenase OS=Homo sapiens OX=9606 GN=PHGDH PE=1 SV=4	0,425	7,112	3,188
A0A0U1RR	ENAH actin regulator OS=Homo sapiens OX=9606 GN=ENAH PE=1 SV=1	0,443	5,557	3,179
Q9BSJ8	Extended synaptotagmin-1 OS=Homo sapiens OX=9606 GN=ESYT1 PE=1 SV=1	2,715	1,296	3,176
P18669	Phosphoglycerate mutase 1 OS=Homo sapiens OX=9606 GN=PGAM1 PE=1 SV=2	0,828	3,52	3,175
P49023	Paxillin OS=Homo sapiens OX=9606 GN=PXN PE=1 SV=3	2,541	0,96	3,173
Q7Z2K6	Endoplasmic reticulum metallopeptidase 1 OS=Homo sapiens OX=9606 GN=ERMP1 PE=1 SV=2	12,958	0,273	3,163
O14773	Tripeptidyl-peptidase 1 OS=Homo sapiens OX=9606 GN=TPP1 PE=1 SV=2	3,586	0,966	3,15
P23743	Diacylglycerol kinase alpha OS=Homo sapiens OX=9606 GN=DGKA PE=1 SV=3	2,69	0,982	3,134
Q03169	Tumor necrosis factor alpha-induced protein 2 OS=Homo sapiens OX=9606 GN=TNFAIP2 PE=1 SV=2	3,923		3,124
P49588	Alanine--tRNA ligase, cytoplasmic OS=Homo sapiens OX=9606 GN=AARS1 PE=1 SV=2	1,618	2,03	3,094
Q9P0K7	Ankycorbin OS=Homo sapiens OX=9606 GN=RAI14 PE=1 SV=2	2,251	1,35	3,091
Q9BWM7	Sideroflexin-3 OS=Homo sapiens OX=9606 GN=SFXN3 PE=1 SV=3	9,579	0,282	3,084
Q13423	NAD(P) transhydrogenase, mitochondrial OS=Homo sapiens OX=9606 GN=NNT PE=1 SV=3	2,134	1,403	3,082
P55809	Succinyl-CoA:3-ketoacid coenzyme A transferase 1, mitochondrial OS=Homo sapiens OX=9606 GN=OXCT1 PE=1 SV=1	2,901	1,043	3,078
Q9UNH7	Sorting nexin-6 OS=Homo sapiens OX=9606 GN=SNX6 PE=1 SV=1	1,212	2,337	3,075
Q9BTW9	Tubulin-specific chaperone D OS=Homo sapiens OX=9606 GN=TBCD PE=1 SV=2	0,502	5,372	3,068
Q99471	Prefoldin subunit 5 OS=Homo sapiens OX=9606 GN=PFDN5 PE=1 SV=2	0,43	5,899	3,058
P11047	Laminin subunit gamma-1 OS=Homo sapiens OX=9606 GN=LAMC1 PE=1 SV=3	1,174	2,708	3,05
P09496	Clathrin light chain A OS=Homo sapiens OX=9606 GN=CLTA PE=1 SV=1	1,59	1,89	3,049
Q709C8	Intermembrane lipid transfer protein VPS13C OS=Homo sapiens OX=9606 GN=VPS13C PE=1 SV=1	0,544	2,215	3,041
Q02809	Procollagen-lysine,2-oxoglutarate 5-dioxygenase 1 OS=Homo sapiens OX=9606 GN=PLOD1 PE=1 SV=2	2,502	1,013	3,038
Q6UW02	Cytochrome P450 20A1 OS=Homo sapiens OX=9606 GN=CYP20A1 PE=1 SV=1	1,455	1,731	3,037
P15121	Aldo-keto reductase family 1 member B1 OS=Homo sapiens OX=9606 GN=AKR1B1 PE=1 SV=3	5,855	0,533	3,036
Q96HC4	PDZ and LIM domain protein 5 OS=Homo sapiens OX=9606 GN=PDLIM5 PE=1 SV=5	3,859	1,013	3,022
Q9BTT6	Leucine-rich repeat-containing protein 1 OS=Homo sapiens OX=9606 GN=LRRC1 PE=1 SV=1	6,783	0,443	3,02
P46939	Utrophin OS=Homo sapiens OX=9606 GN=UTRN PE=1 SV=2	3,604	1,066	3,005
Q96CX2	BTB/POZ domain-containing protein KCTD12 OS=Homo sapiens OX=9606 GN=KCTD12 PE=1 SV=1	1,657	1,976	3,002
A0A669KB	Alpha-synuclein OS=Homo sapiens OX=9606 GN=SNCA PE=1 SV=1	3,626	2,696	2,975
P31150	Rab GDP dissociation inhibitor alpha OS=Homo sapiens OX=9606 GN=GDI1 PE=1 SV=2	0,629	5,764	2,958
A0A0J9YXI	Paraoxonase OS=Homo sapiens OX=9606 GN=PON2 PE=1 SV=1	2,111	1,361	2,955
P42330	Aldo-keto reductase family 1 member C3 OS=Homo sapiens OX=9606 GN=AKR1C3 PE=1 SV=4	2,604	2,68	2,949
P35908	Keratin, type II cytoskeletal 2 epidermal OS=Homo sapiens OX=9606 GN=KRT2 PE=1 SV=2	2,136	1,422	2,948
P30622	CAP-Gly domain-containing linker protein 1 OS=Homo sapiens OX=9606 GN=CLIP1 PE=1 SV=2	1,451	1,405	2,948
Q03135	Caveolin-1 OS=Homo sapiens OX=9606 GN=CAV1 PE=1 SV=4	26,078	0,113	2,937
P17655	Calpain-2 catalytic subunit OS=Homo sapiens OX=9606 GN=CAPN2 PE=1 SV=6	5,889	0,556	2,93
P49721	Proteasome subunit beta type-2 OS=Homo sapiens OX=9606 GN=PSMB2 PE=1 SV=1	1,742	1,835	2,924
Q12797	Aspartyl/asparaginyl beta-hydroxylase OS=Homo sapiens OX=9606 GN=ASPH PE=1 SV=3	3,33	0,863	2,92
Q13438	Protein OS-9 OS=Homo sapiens OX=9606 GN=OS9 PE=1 SV=1	2,175	1,23	2,912
P48506	Glutamate--cysteine ligase catalytic subunit OS=Homo sapiens OX=9606 GN=GCLC PE=1 SV=2	0,718	6,77	2,895
P08758	Annexin A5 OS=Homo sapiens OX=9606 GN=ANXA5 PE=1 SV=2	1,194	2,732	2,884
Q15363	Transmembrane emp24 domain-containing protein 2 OS=Homo sapiens OX=9606 GN=TMED2 PE=1 SV=1	1,244	2,359	2,878
O14879	Interferon-induced protein with tetratricopeptide repeats 3 OS=Homo sapiens OX=9606 GN=IFIT3 PE=1 SV=1	5,775	0,485	2,87
P00167	Cytochrome b5 OS=Homo sapiens OX=9606 GN=CYB5A PE=1 SV=2	2,845	0,997	2,867
Q16527	Cysteine and glycine-rich protein 2 OS=Homo sapiens OX=9606 GN=CSRP2 PE=1 SV=3	0,157	10,63	2,856
P01024	Complement C3 OS=Homo sapiens OX=9606 GN=C3 PE=1 SV=2	3,539	0,286	2,854
P11171	Protein 4.1 OS=Homo sapiens OX=9606 GN=EPB41 PE=1 SV=4	0,417	6,053	2,822
Q13126	S-methyl-5'-thioadenosine phosphorylase OS=Homo sapiens OX=9606 GN=MTAP PE=1 SV=2	1,011	2,938	2,82
P55011	Solute carrier family 12 member 2 OS=Homo sapiens OX=9606 GN=SLC12A2 PE=1 SV=1	1,293	2,075	2,815
P21291	Cysteine and glycine-rich protein 1 OS=Homo sapiens OX=9606 GN=CSRP1 PE=1 SV=3	2,557	1,206	2,811
P55209	Nucleosome assembly protein 1-like 1 OS=Homo sapiens OX=9606 GN=NAP1L1 PE=1 SV=1	0,307	8,387	2,806
Q9Y2B0	Protein canopy homolog 2 OS=Homo sapiens OX=9606 GN=CNPY2 PE=1 SV=1	1,5	1,84	2,784
O00429	Dynamin-1-like protein OS=Homo sapiens OX=9606 GN=DNM1L PE=1 SV=2	0,786	3,213	2,778
P60174	Triosephosphate isomerase OS=Homo sapiens OX=9606 GN=TPI1 PE=1 SV=4	0,707	3,679	2,756
P35527	Keratin, type I cytoskeletal 9 OS=Homo sapiens OX=9606 GN=KRT9 PE=1 SV=3	1,11	2,482	2,729
P08243	Asparagine synthetase [glutamine-hydrolyzing] OS=Homo sapiens OX=9606 GN=ASNS PE=1 SV=4	0,84	3,17	2,72
P48723	Heat shock 70 kDa protein 13 OS=Homo sapiens OX=9606 GN=HSPA13 PE=1 SV=1	0,749	2,803	2,713
A0A087X1	Serpin B6 OS=Homo sapiens OX=9606 GN=SERPINB6 PE=1 SV=1	4,273	0,592	2,7
O00291	Huntingtin-interacting protein 1 OS=Homo sapiens OX=9606 GN=HIP1 PE=1 SV=5	1,171	1,827	2,699
Q6NZI2	Caveolae-associated protein 1 OS=Homo sapiens OX=9606 GN=CAVIN1 PE=1 SV=1	34,161	0,095	2,681
Q04760	Lactoylglutathione lyase OS=Homo sapiens OX=9606 GN=GLO1 PE=1 SV=4	0,313	11,785	2,674
Q16719	Kynureninase OS=Homo sapiens OX=9606 GN=KYNU PE=1 SV=1	1000	0,001	2,661
Q9Y394	Dehydrogenase/reductase SDR family member 7 OS=Homo sapiens OX=9606 GN=DHRS7 PE=1 SV=1	2,709	0,91	2,657
Q9Y6Y8	SEC23-interacting protein OS=Homo sapiens OX=9606 GN=SEC23IP PE=1 SV=1	1,347	2,074	2,638
Q01518	Adenylyl cyclase-associated protein 1 OS=Homo sapiens OX=9606 GN=CAP1 PE=1 SV=5	1,278	2,378	2,634
Q9HC35	Echinoderm microtubule-associated protein-like 4 OS=Homo sapiens OX=9606 GN=EML4 PE=1 SV=3	0,531	4,956	2,628
Q14738	Serine/threonine-protein phosphatase 2A 56 kDa regulatory subunit delta isoform OS=Homo sapiens OX=9606 GN=PPP2R5D	0,506	7,85	2,61
Q08378	Golgin subfamily A member 3 OS=Homo sapiens OX=9606 GN=GOLGA3 PE=1 SV=2	1,417	2,012	2,596

P07237	Protein disulfide-isomerase OS=Homo sapiens OX=9606 GN=P4HB PE=1 SV=3	2,417	1,073	2,583
P35237	Serpin B6 OS=Homo sapiens OX=9606 GN=SERPINB6 PE=1 SV=3	4,218	0,616	2,573
P20073	Annexin A7 OS=Homo sapiens OX=9606 GN=ANXA7 PE=1 SV=3	1,944	1,319	2,552
P07942	Laminin subunit beta-1 OS=Homo sapiens OX=9606 GN=LAMB1 PE=1 SV=2	1,042	2,388	2,547
P07858	Cathepsin B OS=Homo sapiens OX=9606 GN=CTSB PE=1 SV=3	1,355	2,046	2,539
Q9Y6M5	Proton-coupled zinc antiporter SLC30A1 OS=Homo sapiens OX=9606 GN=SLC30A1 PE=1 SV=3	9,33	0,39	2,53
P26006	Integrin alpha-3 OS=Homo sapiens OX=9606 GN=ITGA3 PE=1 SV=5	11,518	0,186	2,506
P35613	Basigin OS=Homo sapiens OX=9606 GN=BSG PE=1 SV=2	2,234	1,158	2,481
O15533	Tapasin OS=Homo sapiens OX=9606 GN=TAPBP PE=1 SV=2	28,115	0,224	2,475
O75874	Isocitrate dehydrogenase [NADP] cytoplasmic OS=Homo sapiens OX=9606 GN=IDH1 PE=1 SV=2	0,429	6,203	2,467
P35579	Myosin-9 OS=Homo sapiens OX=9606 GN=MYH9 PE=1 SV=4	4,205	0,63	2,467
P32455	Guanylate-binding protein 1 OS=Homo sapiens OX=9606 GN=GBP1 PE=1 SV=2	19,063	0,117	2,455
P13645	Keratin, type I cytoskeletal 10 OS=Homo sapiens OX=9606 GN=KRT10 PE=1 SV=6	2,024	1,242	2,444
P09914	Interferon-induced protein with tetratricopeptide repeats 1 OS=Homo sapiens OX=9606 GN=IFIT1 PE=1 SV=2	5,499	0,501	2,44
Q13501	Sequestosome-1 OS=Homo sapiens OX=9606 GN=SQSTM1 PE=1 SV=1	1,681	0,506	2,438
Q8NHP8	Putative phospholipase B-like 2 OS=Homo sapiens OX=9606 GN=PLBD2 PE=1 SV=2	1,101	2,031	2,436
P53999	Activated RNA polymerase II transcriptional coactivator p15 OS=Homo sapiens OX=9606 GN=SUB1 PE=1 SV=3	0,636	3,681	2,431
Q7Z5L9	Interferon regulatory factor 2-binding protein 2 OS=Homo sapiens OX=9606 GN=IRF2BP2 PE=1 SV=2	1,05	1,498	2,423
P00387	NADH-cytochrome b5 reductase 3 OS=Homo sapiens OX=9606 GN=CYB5R3 PE=1 SV=3	2,275	0,919	2,423
P27816	Microtubule-associated protein 4 OS=Homo sapiens OX=9606 GN=MAP4 PE=1 SV=3	1,829	1,22	2,421
O14828	Secretory carrier-associated membrane protein 3 OS=Homo sapiens OX=9606 GN=SCAMP3 PE=1 SV=3	2,453	0,945	2,419
P04040	Catalase OS=Homo sapiens OX=9606 GN=CAT PE=1 SV=3	2,631	0,863	2,419
Q6P1A2	Lysophospholipid acyltransferase 5 OS=Homo sapiens OX=9606 GN=LPCAT3 PE=1 SV=1	3,237	0,716	2,417
Q9NZI8	Insulin-like growth factor 2 mRNA-binding protein 1 OS=Homo sapiens OX=9606 GN=IGF2BP1 PE=1 SV=2	0,112	22,163	2,413
P15170	Eukaryotic peptide chain release factor GTP-binding subunit ERF3A OS=Homo sapiens OX=9606 GN=GSPT1 PE=1 SV=1	0,848	2,28	2,405
P22314	Ubiquitin-like modifier-activating enzyme 1 OS=Homo sapiens OX=9606 GN=UBA1 PE=1 SV=3	0,669	3,562	2,398
P21964	Catechol O-methyltransferase OS=Homo sapiens OX=9606 GN=COMT PE=1 SV=2	6,477	0,451	2,397
O75340	Programmed cell death protein 6 OS=Homo sapiens OX=9606 GN=PDCD6 PE=1 SV=1	1,266	1,585	2,396
O60502	Protein O-GlcNAcase OS=Homo sapiens OX=9606 GN=OGA PE=1 SV=2	1,226	2,056	2,395
E7EVA0	Microtubule-associated protein OS=Homo sapiens OX=9606 GN=MAP4 PE=1 SV=1	1,577	1,319	2,391
Q96KA5	Lipid scramblase CLPTM1L OS=Homo sapiens OX=9606 GN=CLPTM1L PE=1 SV=1	0,973	2,879	2,39
O00461	Golgi integral membrane protein 4 OS=Homo sapiens OX=9606 GN=GOLIM4 PE=1 SV=1	1,098	2,358	2,378
P05026	Sodium/potassium-transporting ATPase subunit beta-1 OS=Homo sapiens OX=9606 GN=ATP1B1 PE=1 SV=1	3,26	0,809	2,364
Q9NZM1	Myoferlin OS=Homo sapiens OX=9606 GN=MYOF PE=1 SV=1	25,704	0,097	2,364
P67936	Tropomyosin alpha-4 chain OS=Homo sapiens OX=9606 GN=TPM4 PE=1 SV=3	4,216	0,583	2,363
P10155	RNA-binding protein RO60 OS=Homo sapiens OX=9606 GN=RO60 PE=1 SV=2	0,518	4,314	2,357
O94979	Protein transport protein Sec31A OS=Homo sapiens OX=9606 GN=SEC31A PE=1 SV=3	1,578	1,503	2,357
Q8WWI1	LIM domain only protein 7 OS=Homo sapiens OX=9606 GN=LMO7 PE=1 SV=3	6,777	0,292	2,35
P05997	Collagen alpha-2(V) chain OS=Homo sapiens OX=9606 GN=COL5A2 PE=1 SV=3	3,627	0,463	2,347
P54577	Tyrosine--tRNA ligase, cytoplasmic OS=Homo sapiens OX=9606 GN=YARS1 PE=1 SV=4	0,822	3,067	2,342
Q96IZ0	PRKC apoptosis WT1 regulator protein OS=Homo sapiens OX=9606 GN=PAWR PE=1 SV=1	2,118	1,142	2,341
Q16740	ATP-dependent Clp protease proteolytic subunit, mitochondrial OS=Homo sapiens OX=9606 GN=CLPP PE=1 SV=1	1,657	1,523	2,336
Q9Y4L1	Hypoxia up-regulated protein 1 OS=Homo sapiens OX=9606 GN=HYOU1 PE=1 SV=1	0,801	3,135	2,331
P41250	Glycine--tRNA ligase OS=Homo sapiens OX=9606 GN=GARS1 PE=1 SV=3	1,114	2,335	2,327
Q86VP6	Cullin-associated NEDD8-dissociated protein 1 OS=Homo sapiens OX=9606 GN=CAND1 PE=1 SV=2	0,55	3,661	2,326
Q04446	1,4-alpha-glucan-branching enzyme OS=Homo sapiens OX=9606 GN=GBE1 PE=1 SV=3	2,042	1,439	2,324
Q3LXA3	Triokinase/FMN cyclase OS=Homo sapiens OX=9606 GN=TKFC PE=1 SV=2	0,506	3,418	2,323
Q8NE86	Calcium uniporter protein, mitochondrial OS=Homo sapiens OX=9606 GN=MCU PE=1 SV=1	4,978	0,48	2,323
P13639	Elongation factor 2 OS=Homo sapiens OX=9606 GN=EEF2 PE=1 SV=4	0,645	3,803	2,313
Q15629	Translocating chain-associated membrane protein 1 OS=Homo sapiens OX=9606 GN=TRAM1 PE=1 SV=3	3,627	0,497	2,306
Q9H5V8	CUB domain-containing protein 1 OS=Homo sapiens OX=9606 GN=CDCP1 PE=1 SV=3	1,366	1,198	2,304
P23381	Tryptophan--tRNA ligase, cytoplasmic OS=Homo sapiens OX=9606 GN=WARS1 PE=1 SV=2	2,395	1,309	2,3
Q14254	Flotillin-2 OS=Homo sapiens OX=9606 GN=FLOT2 PE=1 SV=2	1,518	1,689	2,296
Q9UBV2	Protein sel-1 homolog 1 OS=Homo sapiens OX=9606 GN=SEL1L PE=1 SV=3	1,74	1,254	2,289
P07686	Beta-hexosaminidase subunit beta OS=Homo sapiens OX=9606 GN=HEXB PE=1 SV=4	3,672	0,591	2,286
O60701	UDP-glucose 6-dehydrogenase OS=Homo sapiens OX=9606 GN=UGDH PE=1 SV=1	0,699	3,129	2,275
Q13561	Dynactin subunit 2 OS=Homo sapiens OX=9606 GN=DCTN2 PE=1 SV=4	0,919	2,329	2,26
A0A1B0GV	Keratin, type I cytoskeletal 10 OS=Homo sapiens OX=9606 GN=KRT10 PE=1 SV=2	1,733	1,361	2,26
P33527	Multidrug resistance-associated protein 1 OS=Homo sapiens OX=9606 GN=ABCC1 PE=1 SV=3	3,653	0,559	2,243
P00492	Hypoxanthine-guanine phosphoribosyltransferase OS=Homo sapiens OX=9606 GN=HPRT1 PE=1 SV=2	1,496	1,783	2,242
Q96TC7	Regulator of microtubule dynamics protein 3 OS=Homo sapiens OX=9606 GN=RMDN3 PE=1 SV=2	1,432	1,342	2,238
Q9BW19	Kinesin-like protein KIFC1 OS=Homo sapiens OX=9606 GN=KIFC1 PE=1 SV=2	0,797	2,48	2,23
P52272	Heterogeneous nuclear ribonucleoprotein M OS=Homo sapiens OX=9606 GN=HNRNPM PE=1 SV=3	0,856	2,875	2,229
Q5JRA6	Transport and Golgi organization protein 1 homolog OS=Homo sapiens OX=9606 GN=MIA3 PE=1 SV=1	1,84	1,313	2,226
Q9H1B7	Probable E3 ubiquitin-protein ligase IRF2BPL OS=Homo sapiens OX=9606 GN=IRF2BPL PE=1 SV=1	0,548	3,995	2,212
Q13162	Peroxiredoxin-4 OS=Homo sapiens OX=9606 GN=PRDX4 PE=1 SV=1	1,161	2,436	2,212
Q9Y371	Endophilin-B1 OS=Homo sapiens OX=9606 GN=SH3GLB1 PE=1 SV=1	1,584	1,033	2,209
P27797	Calreticulin OS=Homo sapiens OX=9606 GN=CALR PE=1 SV=1	2,258	0,956	2,201
Q99536	Synaptic vesicle membrane protein VAT-1 homolog OS=Homo sapiens OX=9606 GN=VAT1 PE=1 SV=2	1,537	1,34	2,196
P02768	Albumin OS=Homo sapiens OX=9606 GN=ALB PE=1 SV=2	0,913	0,709	2,19
P11766	Alcohol dehydrogenase class-3 OS=Homo sapiens OX=9606 GN=ADH5 PE=1 SV=4	0,425	5,136	2,187
Q14203	Dynactin subunit 1 OS=Homo sapiens OX=9606 GN=DCTN1 PE=1 SV=3	0,856	2,79	2,187
P21397	Amine oxidase [flavin-containing] A OS=Homo sapiens OX=9606 GN=MAOA PE=1 SV=1	1,476	1,256	2,184
Q99816	Tumor susceptibility gene 101 protein OS=Homo sapiens OX=9606 GN=TSG101 PE=1 SV=2	0,823	2,23	2,182
P04264	Keratin, type II cytoskeletal 1 OS=Homo sapiens OX=9606 GN=KRT1 PE=1 SV=6	1,361	1,559	2,18
H0YD14	Myoferlin (Fragment) OS=Homo sapiens OX=9606 GN=MYOF PE=1 SV=1	14,692	0,141	2,177
P07355	Annexin A2 OS=Homo sapiens OX=9606 GN=ANXA2 PE=1 SV=2	6,359	0,345	2,175
Q14165	Malectin OS=Homo sapiens OX=9606 GN=MLEC PE=1 SV=1	0,809	2,631	2,167
P51659	Peroxisomal multifunctional enzyme type 2 OS=Homo sapiens OX=9606 GN=HSD17B4 PE=1 SV=3	1,493	1,627	2,167
P49589	Cysteine--tRNA ligase, cytoplasmic OS=Homo sapiens OX=9606 GN=CARS1 PE=1 SV=3	1,062	1,974	2,162
Q13492	Phosphatidylinositol-binding clathrin assembly protein OS=Homo sapiens OX=9606 GN=PICALM PE=1 SV=2	2,671	0,752	2,161
P55145	Mesencephalic astrocyte-derived neurotrophic factor OS=Homo sapiens OX=9606 GN=MANF PE=1 SV=3	1,177	1,754	2,16

P52788	Spermine synthase OS=Homo sapiens OX=9606 GN=SMS PE=1 SV=2	0,848	2,386	2,153
P49720	Proteasome subunit beta type-3 OS=Homo sapiens OX=9606 GN=PSMB3 PE=1 SV=2	1,342	1,829	2,149
Q96P70	Importin-9 OS=Homo sapiens OX=9606 GN=IPO9 PE=1 SV=3	0,681	3,326	2,14
P37837	Transaldolase OS=Homo sapiens OX=9606 GN=TALDO1 PE=1 SV=2	1,464	1,217	2,132
Q71U36	Tubulin alpha-1A chain OS=Homo sapiens OX=9606 GN=TUBA1A PE=1 SV=1	0,335	6,994	2,127
Q9UI12	V-type proton ATPase subunit H OS=Homo sapiens OX=9606 GN=ATP6V1H PE=1 SV=1	0,882	2,648	2,12
P00390	Glutathione reductase, mitochondrial OS=Homo sapiens OX=9606 GN=GSR PE=1 SV=2	0,446	4,372	2,119
P11498	Pyruvate carboxylase, mitochondrial OS=Homo sapiens OX=9606 GN=PC PE=1 SV=2	2,082	1,07	2,117
P52565	Rho GDP-dissociation inhibitor 1 OS=Homo sapiens OX=9606 GN=ARHGDIA PE=1 SV=3	0,912	2,37	2,108
P08238	Heat shock protein HSP 90-beta OS=Homo sapiens OX=9606 GN=HSP90AB1 PE=1 SV=4	0,403	5,484	2,104
Q9H488	GDP-fucose protein O-fucosyltransferase 1 OS=Homo sapiens OX=9606 GN=POFUT1 PE=1 SV=1	1,675	1,321	2,103
Q92820	Gamma-glutamyl hydrolase OS=Homo sapiens OX=9606 GN=GGH PE=1 SV=2	0,986	1,797	2,098
O00764	Pyridoxal kinase OS=Homo sapiens OX=9606 GN=PDXK PE=1 SV=1	2,055	0,886	2,097
P05166	Propionyl-CoA carboxylase beta chain, mitochondrial OS=Homo sapiens OX=9606 GN=PCCB PE=1 SV=3	0,757	1,514	2,095
O94973	AP-2 complex subunit alpha-2 OS=Homo sapiens OX=9606 GN=AP2A2 PE=1 SV=2	1,603	1,349	2,095
P02545	Prelamin-A/C OS=Homo sapiens OX=9606 GN=LMNA PE=1 SV=1	17,715	0,133	2,081
P19367	Hexokinase-1 OS=Homo sapiens OX=9606 GN=HK1 PE=1 SV=3	2,64	0,743	2,08
Q99541	Perilipin-2 OS=Homo sapiens OX=9606 GN=PLIN2 PE=1 SV=2	1,215	2,008	2,07
Q9Y263	Phospholipase A-2-activating protein OS=Homo sapiens OX=9606 GN=PLAA PE=1 SV=2	1,544	1,314	2,067
O14950	Myosin regulatory light chain 12B OS=Homo sapiens OX=9606 GN=MYL12B PE=1 SV=2	2,965	0,657	2,062
Q9BRP8	Partner of Y14 and mago OS=Homo sapiens OX=9606 GN=PYM1 PE=1 SV=1	0,593	3,593	2,04
P40763	Signal transducer and activator of transcription 3 OS=Homo sapiens OX=9606 GN=STAT3 PE=1 SV=2	1,796	1,128	2,04
Q15075	Early endosome antigen 1 OS=Homo sapiens OX=9606 GN=EEA1 PE=1 SV=2	4,426	0,555	2,04
Q96TA1	Protein Niban 2 OS=Homo sapiens OX=9606 GN=NIBAN2 PE=1 SV=3	5,315	0,353	2,038
P06733	Alpha-enolase OS=Homo sapiens OX=9606 GN=ENO1 PE=1 SV=2	0,724	3,015	2,036
O15427	Monocarboxylate transporter 4 OS=Homo sapiens OX=9606 GN=SLC16A3 PE=1 SV=1	60,36	0,036	2,028
P24844	Myosin regulatory light polypeptide 9 OS=Homo sapiens OX=9606 GN=MYL9 PE=1 SV=4	2,385	0,759	2,025
P04179	Superoxide dismutase [Mn], mitochondrial OS=Homo sapiens OX=9606 GN=SOD2 PE=1 SV=3	5,853	0,383	2,012
H0YMW4	Annexin OS=Homo sapiens OX=9606 GN=ANXA2 PE=1 SV=2	6,036	0,327	2,009
Q16666	Gamma-interferon-inducible protein 16 OS=Homo sapiens OX=9606 GN=IFI16 PE=1 SV=3	6,619	0,296	1,994
P06756	Integrin alpha-V OS=Homo sapiens OX=9606 GN=ITGAV PE=1 SV=2	2,738	0,645	1,992
P63104	14-3-3 protein zeta/delta OS=Homo sapiens OX=9606 GN=YWHAZ PE=1 SV=1	0,848	2,402	1,988
P07737	Profilin-1 OS=Homo sapiens OX=9606 GN=PFN1 PE=1 SV=2	0,769	2,607	1,979
P10768	S-formylglutathione hydrolase OS=Homo sapiens OX=9606 GN=ESD PE=1 SV=2	0,785	3,385	1,978
P53634	Dipeptidyl peptidase 1 OS=Homo sapiens OX=9606 GN=CTSC PE=1 SV=2	0,274	4,167	1,971
P16615	Sarcoplasmic/endoplasmic reticulum calcium ATPase 2 OS=Homo sapiens OX=9606 GN=ATP2A2 PE=1 SV=1	2,066	0,936	1,967
Q8WZA9	Immunity-related GTPase family Q protein OS=Homo sapiens OX=9606 GN=IRGQ PE=1 SV=1	0,571	3,695	1,966
Q9Y3Z3	Deoxynucleoside triphosphate triphosphohydrolase SAMHD1 OS=Homo sapiens OX=9606 GN=SAMHD1 PE=1 SV=2	5,894	0,335	1,961
O00625	Pirin OS=Homo sapiens OX=9606 GN=PIR PE=1 SV=1	0,416	5,021	1,958
Q9Y6G9	Cytoplasmic dynein 1 light intermediate chain 1 OS=Homo sapiens OX=9606 GN=DYNC1LI1 PE=1 SV=3	0,449	3,753	1,945
P50552	Vasodilator-stimulated phosphoprotein OS=Homo sapiens OX=9606 GN=VASP PE=1 SV=3	3,335	0,57	1,943
P27708	Multifunctional protein CAD OS=Homo sapiens OX=9606 GN=CAD PE=1 SV=3	0,391	5,145	1,942
P49902	Cytosolic purine 5'-nucleotidase OS=Homo sapiens OX=9606 GN=NT5C2 PE=1 SV=1	0,991	2,372	1,941
P29992	Guanine nucleotide-binding protein subunit alpha-11 OS=Homo sapiens OX=9606 GN=GNA11 PE=1 SV=2	3,154	0,628	1,911
P21281	V-type proton ATPase subunit B, brain isoform OS=Homo sapiens OX=9606 GN=ATP6V1B2 PE=1 SV=3	0,919	2,017	1,909
P17301	Integrin alpha-2 OS=Homo sapiens OX=9606 GN=ITGA2 PE=1 SV=2	27,598	0,041	1,909
P04792	Heat shock protein beta-1 OS=Homo sapiens OX=9606 GN=HSPB1 PE=1 SV=2	3,737	0,682	1,898
Q14204	Cytoplasmic dynein 1 heavy chain 1 OS=Homo sapiens OX=9606 GN=DYNC1H1 PE=1 SV=5	0,576	3,143	1,892
Q9UPN3	Microtubule-actin cross-linking factor 1, isoforms 1/2/3/4/5 OS=Homo sapiens OX=9606 GN=MACF1 PE=1 SV=4	0,634	3,055	1,882
Q8NBX0	Saccharopine dehydrogenase-like oxidoreductase OS=Homo sapiens OX=9606 GN=SCCPDH PE=1 SV=1	2,143	0,788	1,881
P07203	Glutathione peroxidase 1 OS=Homo sapiens OX=9606 GN=GPX1 PE=1 SV=4	2,404	0,879	1,876
P80188	Neutrophil gelatinase-associated lipocalin OS=Homo sapiens OX=9606 GN=LCN2 PE=1 SV=2	2,604	0,716	1,864
P13667	Protein disulfide-isomerase A4 OS=Homo sapiens OX=9606 GN=PDIA4 PE=1 SV=2	0,71	2,818	1,847
P49591	Serine--tRNA ligase, cytoplasmic OS=Homo sapiens OX=9606 GN=SARS1 PE=1 SV=3	0,361	5,134	1,833
P29317	Ephrin type-A receptor 2 OS=Homo sapiens OX=9606 GN=EPHA2 PE=1 SV=2	2,344	0,769	1,833
P10619	Lysosomal protective protein OS=Homo sapiens OX=9606 GN=CTSA PE=1 SV=2	4,211	0,341	1,83
Q14914	Prostaglandin reductase 1 OS=Homo sapiens OX=9606 GN=PTGR1 PE=1 SV=2	2,738	0,658	1,826
Q9H074	Polyadenylate-binding protein-interacting protein 1 OS=Homo sapiens OX=9606 GN=PAIP1 PE=1 SV=1	0,637	3,293	1,817
P04632	Calpain small subunit 1 OS=Homo sapiens OX=9606 GN=CAPNS1 PE=1 SV=1	4,362	0,395	1,816
P07197	Neurofilament medium polypeptide OS=Homo sapiens OX=9606 GN=NEFM PE=1 SV=3	0,053	19,872	1,805
P45974	Ubiquitin carboxyl-terminal hydrolase 5 OS=Homo sapiens OX=9606 GN=USP5 PE=1 SV=2	0,552	3,702	1,804
Q14258	E3 ubiquitin/ISG15 ligase TRIM25 OS=Homo sapiens OX=9606 GN=TRIM25 PE=1 SV=2	3,261	0,588	1,799
Q8NBF2	NHL repeat-containing protein 2 OS=Homo sapiens OX=9606 GN=NHLRC2 PE=1 SV=1	0,367	7,052	1,795
P42224	Signal transducer and activator of transcription 1-alpha/beta OS=Homo sapiens OX=9606 GN=STAT1 PE=1 SV=2	4,084	0,414	1,79
P51153	Ras-related protein Rab-13 OS=Homo sapiens OX=9606 GN=RAB13 PE=1 SV=1	3,134	0,544	1,784
P61106	Ras-related protein Rab-14 OS=Homo sapiens OX=9606 GN=RAB14 PE=1 SV=4	3,853	0,54	1,779
Q10471	Polypeptide N-acetylgalactosaminyltransferase 2 OS=Homo sapiens OX=9606 GN=GALNT2 PE=1 SV=1	3,06	0,572	1,774
P55265	Double-stranded RNA-specific adenosine deaminase OS=Homo sapiens OX=9606 GN=ADAR PE=1 SV=4	2,333	0,718	1,771
O60749	Sorting nexin-2 OS=Homo sapiens OX=9606 GN=SNX2 PE=1 SV=2	0,482	2,809	1,769
P26639	Threonine--tRNA ligase 1, cytoplasmic OS=Homo sapiens OX=9606 GN=TARS1 PE=1 SV=3	0,49	3,958	1,761
P30085	UMP-CMP kinase OS=Homo sapiens OX=9606 GN=CMPK1 PE=1 SV=3	0,714	2,052	1,761
P34897	Serine hydroxymethyltransferase, mitochondrial OS=Homo sapiens OX=9606 GN=SHMT2 PE=1 SV=3	0,741	2,025	1,758
Q92616	Stalled ribosome sensor GCN1 OS=Homo sapiens OX=9606 GN=GCN1 PE=1 SV=7	0,808	2,06	1,756
P98172	Ephrin-B1 OS=Homo sapiens OX=9606 GN=EFNB1 PE=1 SV=1	0,287	2,495	1,749
Q7L5N7	Lysophosphatidylcholine acyltransferase 2 OS=Homo sapiens OX=9606 GN=LPCAT2 PE=1 SV=1	1,519	0,338	1,749
Q9Y6E2	eIF5-mimic protein 1 OS=Homo sapiens OX=9606 GN=BZW2 PE=1 SV=1	0,455	4,131	1,745
P04083	Annexin A1 OS=Homo sapiens OX=9606 GN=ANXA1 PE=1 SV=2	5,962	0,31	1,742
O75347	Tubulin-specific chaperone A OS=Homo sapiens OX=9606 GN=TBCA PE=1 SV=3	0,309	5,711	1,741
P63151	Serine/threonine-protein phosphatase 2A 55 kDa regulatory subunit B alpha isoform OS=Homo sapiens OX=9606 GN=PPP2R	0,523	2,121	1,737
Q9NR12	PDZ and LIM domain protein 7 OS=Homo sapiens OX=9606 GN=PDLIM7 PE=1 SV=1	2,427	0,573	1,727
P33176	Kinesin-1 heavy chain OS=Homo sapiens OX=9606 GN=KIF5B PE=1 SV=1	0,822	2,15	1,724
P10644	cAMP-dependent protein kinase type I-alpha regulatory subunit OS=Homo sapiens OX=9606 GN=PRKAR1A PE=1 SV=1	2,052	0,784	1,724

Q5SW79	Centrosomal protein of 170 kDa OS=Homo sapiens OX=9606 GN=CEP170 PE=1 SV=1	0,801	2,371	1,723
P31937	3-hydroxyisobutyrate dehydrogenase, mitochondrial OS=Homo sapiens OX=9606 GN=HIBADH PE=1 SV=2	4,216	0,391	1,708
Q9Y6K5	2'-5'-oligoadenylate synthase 3 OS=Homo sapiens OX=9606 GN=OAS3 PE=1 SV=3	6,286	0,206	1,705
P62244	Small ribosomal subunit protein uS8 OS=Homo sapiens OX=9606 GN=RPS15A PE=1 SV=2	0,86	2,076	1,696
A1X283	SH3 and PX domain-containing protein 2B OS=Homo sapiens OX=9606 GN=SH3PXD2B PE=1 SV=3	0,524	3,923	1,684
P46379	Large proline-rich protein BAG6 OS=Homo sapiens OX=9606 GN=BAG6 PE=1 SV=2	0,555	3,046	1,666
Q9UKK9	ADP-sugar pyrophosphatase OS=Homo sapiens OX=9606 GN=NUDT5 PE=1 SV=1	0,492	1,926	1,659
P14550	Aldo-keto reductase family 1 member A1 OS=Homo sapiens OX=9606 GN=AKR1A1 PE=1 SV=3	0,475	4,519	1,657
Q9H9A6	Leucine-rich repeat-containing protein 40 OS=Homo sapiens OX=9606 GN=LRRC40 PE=1 SV=1	0,432	3,703	1,657
P04062	Lysosomal acid glucosylceramidase OS=Homo sapiens OX=9606 GN=GBA1 PE=1 SV=3	3,57	0,406	1,654
P04818	Thymidylate synthase OS=Homo sapiens OX=9606 GN=TYMS PE=1 SV=3	0,186	10,026	1,648
P38606	V-type proton ATPase catalytic subunit A OS=Homo sapiens OX=9606 GN=ATP6V1A PE=1 SV=2	0,666	2,497	1,645
Q6KB66	Keratin, type II cytoskeletal 80 OS=Homo sapiens OX=9606 GN=KRT80 PE=1 SV=2	6,32	0,265	1,644
A0FGR8	Extended synaptotagmin-2 OS=Homo sapiens OX=9606 GN=ESYT2 PE=1 SV=1	3,65	0,463	1,642
P53396	ATP-citrate synthase OS=Homo sapiens OX=9606 GN=ACLY PE=1 SV=3	0,331	4,718	1,641
Q9NXU5	ADP-ribosylation factor-like protein 15 OS=Homo sapiens OX=9606 GN=ARL15 PE=1 SV=1	0,778	2,063	1,639
A0A7I2V5(	Large proline-rich protein BAG6 OS=Homo sapiens OX=9606 GN=BAG6 PE=1 SV=1	0,48	3,439	1,637
Q15019	Septin-2 OS=Homo sapiens OX=9606 GN=SEPTIN2 PE=1 SV=1	0,706	2,245	1,631
Q13586	Stromal interaction molecule 1 OS=Homo sapiens OX=9606 GN=STIM1 PE=1 SV=3	3,242	0,509	1,626
Q12929	Epidermal growth factor receptor kinase substrate 8 OS=Homo sapiens OX=9606 GN=EPS8 PE=1 SV=1	0,446	1,585	1,621
Q93008	Probable ubiquitin carboxyl-terminal hydrolase FAF-X OS=Homo sapiens OX=9606 GN=USP9X PE=1 SV=4	0,639	2,009	1,62
Q9BVA1	Tubulin beta-2B chain OS=Homo sapiens OX=9606 GN=TUBB2B PE=1 SV=1	0,238	6,357	1,614
P11441	Ubiquitin-like protein 4A OS=Homo sapiens OX=9606 GN=UBL4A PE=1 SV=1	0,749	2,624	1,614
Q09028	Histone-binding protein RBBP4 OS=Homo sapiens OX=9606 GN=RBBP4 PE=1 SV=3	0,411	5,9	1,613
Q14573	Inositol 1,4,5-trisphosphate receptor type 3 OS=Homo sapiens OX=9606 GN=ITPR3 PE=1 SV=2	7,769	0,212	1,61
Q6DD88	Atlastin-3 OS=Homo sapiens OX=9606 GN=ATL3 PE=1 SV=1	4,245	0,395	1,608
Q9HAV4	Exportin-5 OS=Homo sapiens OX=9606 GN=XPO5 PE=1 SV=1	0,288	6,008	1,6
Q9BTV4	Transmembrane protein 43 OS=Homo sapiens OX=9606 GN=TMEM43 PE=1 SV=1	2,451	0,717	1,598
Q9NUP9	Protein lin-7 homolog C OS=Homo sapiens OX=9606 GN=LIN7C PE=1 SV=1	2,053	0,66	1,596
Q92692	Nectin-2 OS=Homo sapiens OX=9606 GN=NECTIN2 PE=1 SV=1	2,477	0,695	1,59
Q9UMR2	ATP-dependent RNA helicase DDX19B OS=Homo sapiens OX=9606 GN=DDX19B PE=1 SV=1	0,713	2,148	1,582
P19224	UDP-glucuronosyltransferase 1-6 OS=Homo sapiens OX=9606 GN=UGT1A6 PE=1 SV=2	2,682	0,085	1,574
Q15582	Transforming growth factor-beta-induced protein ig-h3 OS=Homo sapiens OX=9606 GN=TGFBI PE=1 SV=1	0,277	5,99	1,565
Q14315	Filamin-C OS=Homo sapiens OX=9606 GN=FLNC PE=1 SV=3	0,655	2,426	1,565
O76024	Wolframin OS=Homo sapiens OX=9606 GN=WFS1 PE=1 SV=2	4,431	0,331	1,563
Q9NVA2	Septin-11 OS=Homo sapiens OX=9606 GN=SEPTIN11 PE=1 SV=3	0,478	3,124	1,551
Q8NBJ7	Inactive C-alpha-formylglycine-generating enzyme 2 OS=Homo sapiens OX=9606 GN=SUMF2 PE=1 SV=2	2,167	0,712	1,547
P50995	Annexin A11 OS=Homo sapiens OX=9606 GN=ANXA11 PE=1 SV=1	3,436	0,477	1,545
Q9Y5K6	CD2-associated protein OS=Homo sapiens OX=9606 GN=CD2AP PE=1 SV=1	3,435	0,462	1,545
Q15366	Poly(rC)-binding protein 2 OS=Homo sapiens OX=9606 GN=PCBP2 PE=1 SV=1	0,504	3,019	1,543
P09651	Heterogeneous nuclear ribonucleoprotein A1 OS=Homo sapiens OX=9606 GN=HNRNPA1 PE=1 SV=5	0,775	2,024	1,542
P68104	Elongation factor 1-alpha 1 OS=Homo sapiens OX=9606 GN=EEF1A1 PE=1 SV=1	0,67	6,263	1,536
O00410	Importin-5 OS=Homo sapiens OX=9606 GN=IPO5 PE=1 SV=4	0,514	3,429	1,534
Q13177	Serine/threonine-protein kinase PAK 2 OS=Homo sapiens OX=9606 GN=PAK2 PE=1 SV=3	0,648	2,806	1,53
Q14739	Delta(14)-sterol reductase LBR OS=Homo sapiens OX=9606 GN=LBR PE=1 SV=2	0,722	2,106	1,525
P49915	GMP synthase [glutamine-hydrolyzing] OS=Homo sapiens OX=9606 GN=GMPS PE=1 SV=1	0,597	2,16	1,523
Q14232	Translation initiation factor eIF2B subunit alpha OS=Homo sapiens OX=9606 GN=EIF2B1 PE=1 SV=1	0,737	2,929	1,522
P22307	Sterol carrier protein 2 OS=Homo sapiens OX=9606 GN=SCP2 PE=1 SV=2	2,879	0,632	1,52
Q9NQC3	Reticulon-4 OS=Homo sapiens OX=9606 GN=RTN4 PE=1 SV=2	2,4	1,042	1,517
Q15181	Inorganic pyrophosphatase OS=Homo sapiens OX=9606 GN=PPA1 PE=1 SV=2	0,511	3,092	1,514
Q6YHK3	CD109 antigen OS=Homo sapiens OX=9606 GN=CD109 PE=1 SV=2	7,208	0,087	1,514
B7ZLQ5	SMARCA1 protein OS=Homo sapiens OX=9606 GN=SMARCA1 PE=1 SV=1	0,389	4,033	1,513
Q15645	Pachytene checkpoint protein 2 homolog OS=Homo sapiens OX=9606 GN=TRIP13 PE=1 SV=2	0,426	3,34	1,512
P17174	Aspartate aminotransferase, cytoplasmic OS=Homo sapiens OX=9606 GN=GOT1 PE=1 SV=3	0,493	2,616	1,509
O75477	Erlin-1 OS=Homo sapiens OX=9606 GN=ERLIN1 PE=1 SV=2	2,672	0,552	1,506
P15531	Nucleoside diphosphate kinase A OS=Homo sapiens OX=9606 GN=NME1 PE=1 SV=1	0,583	2,152	1,504
Q03518	Antigen peptide transporter 1 OS=Homo sapiens OX=9606 GN=TAP1 PE=1 SV=3	28,515	0,102	1,497
Q14141	Septin-6 OS=Homo sapiens OX=9606 GN=SEPTIN6 PE=1 SV=4	0,364	5,865	1,486
O76003	Glutaredoxin-3 OS=Homo sapiens OX=9606 GN=GLRX3 PE=1 SV=2	0,624	2,937	1,485
O75915	PRA1 family protein 3 OS=Homo sapiens OX=9606 GN=ARL6IP5 PE=1 SV=1	2,396	0,668	1,483
Q8WXG1	S-adenosylmethionine-dependent nucleotide dehydratase RSAD2 OS=Homo sapiens OX=9606 GN=RSAD2 PE=1 SV=1	2,275	0,43	1,483
O60568	Multifunctional procollagen lysine hydroxylase and glycosyltransferase LH3 OS=Homo sapiens OX=9606 GN=PLOD3 PE=1 SV=	2,196	0,555	1,478
Q9NY33	Dipeptidyl peptidase 3 OS=Homo sapiens OX=9606 GN=DPP3 PE=1 SV=2	0,671	2,092	1,476
Q8WW12	PEST proteolytic signal-containing nuclear protein OS=Homo sapiens OX=9606 GN=PCNP PE=1 SV=2	0,581	2,523	1,471
Q13510	Acid ceramidase OS=Homo sapiens OX=9606 GN=ASAH1 PE=1 SV=5	2,454	0,613	1,466
Q9UHB6	LIM domain and actin-binding protein 1 OS=Homo sapiens OX=9606 GN=LIMA1 PE=1 SV=1	5,248	0,374	1,46
P55285	Cadherin-6 OS=Homo sapiens OX=9606 GN=CDH6 PE=1 SV=1	0,216	5,604	1,453
P61088	Ubiquitin-conjugating enzyme E2 N OS=Homo sapiens OX=9606 GN=UBE2N PE=1 SV=1	0,538	2,748	1,453
P37802	Transgelin-2 OS=Homo sapiens OX=9606 GN=TAGLN2 PE=1 SV=3	3,127	0,553	1,447
Q9HC07	Putative divalent cation/proton antiporter TMEM165 OS=Homo sapiens OX=9606 GN=TMEM165 PE=1 SV=1	3,722	0,369	1,442
P60842	Eukaryotic initiation factor 4A-I OS=Homo sapiens OX=9606 GN=EIF4A1 PE=1 SV=1	0,495	2,812	1,438
P29323	Ephrin type-B receptor 2 OS=Homo sapiens OX=9606 GN=EPHB2 PE=1 SV=5	4,006	0,2	1,436
Q06210	Glutamine--fructose-6-phosphate aminotransferase [isomerizing] 1 OS=Homo sapiens OX=9606 GN=GFPT1 PE=1 SV=3	0,544	2,744	1,435
O14617	AP-3 complex subunit delta-1 OS=Homo sapiens OX=9606 GN=AP3D1 PE=1 SV=1	0,624	2,19	1,434
Q03519	Antigen peptide transporter 2 OS=Homo sapiens OX=9606 GN=TAP2 PE=1 SV=1	16,388	0,073	1,42
P62937	Peptidyl-prolyl cis-trans isomerase A OS=Homo sapiens OX=9606 GN=PPIA PE=1 SV=2	0,558	2,222	1,418
P13591	Neural cell adhesion molecule 1 OS=Homo sapiens OX=9606 GN=NCAM1 PE=1 SV=3	0,157	4,503	1,417
Q9NP72	Ras-related protein Rab-18 OS=Homo sapiens OX=9606 GN=RAB18 PE=1 SV=1	2,141	0,701	1,407
P46940	Ras GTPase-activating-like protein IQGAP1 OS=Homo sapiens OX=9606 GN=IQGAP1 PE=1 SV=1	4,945	0,288	1,405
O95786	Antiviral innate immune response receptor RIG-I OS=Homo sapiens OX=9606 GN=RIGI PE=1 SV=2	3,222	0,427	1,404
Q13409	Cytoplasmic dynein 1 intermediate chain 2 OS=Homo sapiens OX=9606 GN=DYNC1I2 PE=1 SV=3	0,604	2,689	1,403
P52926	High mobility group protein HMGI-C OS=Homo sapiens OX=9606 GN=HMGA2 PE=1 SV=1	0,019	68,759	1,398

Q96PC5	Melanoma inhibitory activity protein 2 OS=Homo sapiens OX=9606 GN=MIA2 PE=1 SV=4	2,225	0,953	1,39
O00592	Podocalyxin OS=Homo sapiens OX=9606 GN=PODXL PE=1 SV=2	5,203	0,212	1,389
P16949	Stathmin OS=Homo sapiens OX=9606 GN=STMN1 PE=1 SV=3	0,287	4,946	1,388
Q8NBJ5	Procollagen galactosyltransferase 1 OS=Homo sapiens OX=9606 GN=COLGALT1 PE=1 SV=1	3,861	0,337	1,387
Q9H857	5'-nucleotidase domain-containing protein 2 OS=Homo sapiens OX=9606 GN=NT5DC2 PE=1 SV=1	0,184	5,426	1,379
Q9BXK5	Bcl-2-like protein 13 OS=Homo sapiens OX=9606 GN=BCL2L13 PE=1 SV=1	6,421	0,207	1,377
Q9UBF2	Coatomer subunit gamma-2 OS=Homo sapiens OX=9606 GN=COPG2 PE=1 SV=1	0,596	2,32	1,374
Q9HD45	Transmembrane 9 superfamily member 3 OS=Homo sapiens OX=9606 GN=TM9SF3 PE=1 SV=2	2,003	0,598	1,373
Q8IWA5	Choline transporter-like protein 2 OS=Homo sapiens OX=9606 GN=SLC44A2 PE=1 SV=3	9,129	0,167	1,373
Q9UK76	Jupiter microtubule associated homolog 1 OS=Homo sapiens OX=9606 GN=JPT1 PE=1 SV=3	0,653	2,826	1,369
P14324	Farnesyl pyrophosphate synthase OS=Homo sapiens OX=9606 GN=FDPS PE=1 SV=4	0,276	4,836	1,363
Q7Z6Z7	E3 ubiquitin-protein ligase HUWE1 OS=Homo sapiens OX=9606 GN=HUWE1 PE=1 SV=3	0,508	2,608	1,359
P26358	DNA (cytosine-5)-methyltransferase 1 OS=Homo sapiens OX=9606 GN=DNMT1 PE=1 SV=2	0,348	2,379	1,354
Q96QK1	Vacuolar protein sorting-associated protein 35 OS=Homo sapiens OX=9606 GN=VPS35 PE=1 SV=2	0,779	2,06	1,348
Q01813	ATP-dependent 6-phosphofructokinase, platelet type OS=Homo sapiens OX=9606 GN=PFKP PE=1 SV=2	3,97	0,295	1,345
O15347	High mobility group protein B3 OS=Homo sapiens OX=9606 GN=HMGB3 PE=1 SV=4	0,254	5,672	1,344
Q13576	Ras GTPase-activating-like protein IQGAP2 OS=Homo sapiens OX=9606 GN=IQGAP2 PE=1 SV=4	0,51	2,883	1,338
Q9Y3F4	Serine-threonine kinase receptor-associated protein OS=Homo sapiens OX=9606 GN=STRAP PE=1 SV=1	0,399	3,063	1,329
Q13404	Ubiquitin-conjugating enzyme E2 variant 1 OS=Homo sapiens OX=9606 GN=UBE2V1 PE=1 SV=2	0,563	2,363	1,328
Q13895	Bystin OS=Homo sapiens OX=9606 GN=BYSL PE=1 SV=3	0,427	2,265	1,327
P62333	26S proteasome regulatory subunit 10B OS=Homo sapiens OX=9606 GN=PSMC6 PE=1 SV=1	0,67	2,039	1,327
Q96SQ9	Cytochrome P450 2S1 OS=Homo sapiens OX=9606 GN=CYP2S1 PE=1 SV=2	2,191	0,792	1,327
O95373	Importin-7 OS=Homo sapiens OX=9606 GN=IPO7 PE=1 SV=1	0,597	2,205	1,326
Q9HD20	Endoplasmic reticulum transmembrane helix translocase OS=Homo sapiens OX=9606 GN=ATP13A1 PE=1 SV=2	3,135	0,45	1,326
O43169	Cytochrome b5 type B OS=Homo sapiens OX=9606 GN=CYB5B PE=1 SV=3	2,963	0,538	1,32
P29728	2'-5'-oligoadenylate synthase 2 OS=Homo sapiens OX=9606 GN=OAS2 PE=1 SV=3	11,096	0,099	1,319
P41227	N-alpha-acetyltransferase 10 OS=Homo sapiens OX=9606 GN=NAA10 PE=1 SV=1	0,645	2,165	1,316
O00754	Lysosomal alpha-mannosidase OS=Homo sapiens OX=9606 GN=MAN2B1 PE=1 SV=3	2,015	0,693	1,316
Q9C0C2	182 kDa tankyrase-1-binding protein OS=Homo sapiens OX=9606 GN=TNKS1BP1 PE=1 SV=4	2,741	0,515	1,304
P43034	Platelet-activating factor acetylhydrolase IB subunit beta OS=Homo sapiens OX=9606 GN=PAFAH1B1 PE=1 SV=2	0,52	2,474	1,289
P46926	Glucosamine-6-phosphate isomerase 1 OS=Homo sapiens OX=9606 GN=GNPDA1 PE=1 SV=1	0,557	3,085	1,288
Q8IWZ3	Ankyrin repeat and KH domain-containing protein 1 OS=Homo sapiens OX=9606 GN=ANKHD1 PE=1 SV=1	0,569	2,722	1,288
P27348	14-3-3 protein theta OS=Homo sapiens OX=9606 GN=YWHAQ PE=1 SV=1	0,536	2,45	1,288
Q92817	Envoplakin OS=Homo sapiens OX=9606 GN=EVPL PE=1 SV=3	2,871	0,303	1,287
P17812	CTP synthase 1 OS=Homo sapiens OX=9606 GN=CTPS1 PE=1 SV=2	0,351	3,701	1,285
P42892	Endothelin-converting enzyme 1 OS=Homo sapiens OX=9606 GN=ECE1 PE=1 SV=2	3,461	0,352	1,282
P43686	26S proteasome regulatory subunit 6B OS=Homo sapiens OX=9606 GN=PSMC4 PE=1 SV=2	0,746	2,075	1,28
P50395	Rab GDP dissociation inhibitor beta OS=Homo sapiens OX=9606 GN=GDI2 PE=1 SV=2	0,515	2,551	1,276
Q6YN16	Hydroxysteroid dehydrogenase-like protein 2 OS=Homo sapiens OX=9606 GN=HSDL2 PE=1 SV=1	2,931	0,437	1,275
P19174	1-phosphatidylinositol 4,5-bisphosphate phosphodiesterase gamma-1 OS=Homo sapiens OX=9606 GN=PLCG1 PE=1 SV=1	0,344	3,286	1,266
O95433	Activator of 90 kDa heat shock protein ATPase homolog 1 OS=Homo sapiens OX=9606 GN=AHSA1 PE=1 SV=1	0,383	2,685	1,264
P62330	ADP-ribosylation factor 6 OS=Homo sapiens OX=9606 GN=ARF6 PE=1 SV=2	2,081	0,476	1,26
Q13867	Bleomycin hydrolase OS=Homo sapiens OX=9606 GN=BLMH PE=1 SV=1	0,355	2,911	1,259
O60341	Lysine-specific histone demethylase 1A OS=Homo sapiens OX=9606 GN=KDM1A PE=1 SV=2	0,307	3,614	1,256
O14929	Histone acetyltransferase type B catalytic subunit OS=Homo sapiens OX=9606 GN=HAT1 PE=1 SV=2	0,465	2,402	1,256
O14672	Disintegrin and metalloproteinase domain-containing protein 10 OS=Homo sapiens OX=9606 GN=ADAM10 PE=1 SV=1	2,033	0,544	1,253
A0A6Q8PF	Platelet-activating factor acetylhydrolase IB subunit alpha OS=Homo sapiens OX=9606 GN=PAFAH1B1 PE=1 SV=1	0,509	2,645	1,243
P41240	Tyrosine-protein kinase CSK OS=Homo sapiens OX=9606 GN=CSK PE=1 SV=1	0,44	3,833	1,242
Q9Y696	Chloride intracellular channel protein 4 OS=Homo sapiens OX=9606 GN=CLIC4 PE=1 SV=4	0,395	2,809	1,239
P30086	Phosphatidylethanolamine-binding protein 1 OS=Homo sapiens OX=9606 GN=PEBP1 PE=1 SV=3	0,318	3,688	1,238
Q15833	Syntaxin-binding protein 2 OS=Homo sapiens OX=9606 GN=STXBP2 PE=1 SV=2	1000	0,001	1,238
Q9UIJ7	GTP:AMP phosphotransferase AK3, mitochondrial OS=Homo sapiens OX=9606 GN=AK3 PE=1 SV=4	2,122	0,607	1,236
Q16181	Septin-7 OS=Homo sapiens OX=9606 GN=SEPTIN7 PE=1 SV=2	0,462	2,815	1,235
P23528	Cofilin-1 OS=Homo sapiens OX=9606 GN=CFL1 PE=1 SV=3	0,675	2,161	1,235
O15143	Actin-related protein 2/3 complex subunit 1B OS=Homo sapiens OX=9606 GN=ARPC1B PE=1 SV=3	2,418	0,517	1,227
Q06830	Peroxiredoxin-1 OS=Homo sapiens OX=9606 GN=PRDX1 PE=1 SV=1	0,388	3,022	1,22
Q96Q06	Perilipin-4 OS=Homo sapiens OX=9606 GN=PLIN4 PE=1 SV=3	1000	0,001	1,22
Q96RT1	Erbin OS=Homo sapiens OX=9606 GN=ERBIN PE=1 SV=2	2,161	0,523	1,219
Q9Y6N5	Sulfide:quinone oxidoreductase, mitochondrial OS=Homo sapiens OX=9606 GN=SQOR PE=1 SV=1	8,136	0,183	1,215
Q12765	Secernin-1 OS=Homo sapiens OX=9606 GN=SCRN1 PE=1 SV=2	0,5	2,448	1,214
P52209	6-phosphogluconate dehydrogenase, decarboxylating OS=Homo sapiens OX=9606 GN=PGD PE=1 SV=3	0,152	7,988	1,211
Q99497	Parkinson disease protein 7 OS=Homo sapiens OX=9606 GN=PARK7 PE=1 SV=2	0,518	2,396	1,207
P78371	T-complex protein 1 subunit beta OS=Homo sapiens OX=9606 GN=CCT2 PE=1 SV=4	0,617	2,223	1,193
P58107	Epiplakin OS=Homo sapiens OX=9606 GN=EPPK1 PE=1 SV=3	1,676	0,492	1,193
Q9Y2D5	PALM2-AKAP2 fusion protein OS=Homo sapiens OX=9606 GN=PALM2AKAP2 PE=1 SV=4	3,083	0,428	1,19
Q9NZ08	Endoplasmic reticulum aminopeptidase 1 OS=Homo sapiens OX=9606 GN=ERAP1 PE=1 SV=3	9,746	0,131	1,189
Q14CN2	Calcium-activated chloride channel regulator 4 OS=Homo sapiens OX=9606 GN=CLCA4 PE=1 SV=2			1,187
Q16537	Serine/threonine-protein phosphatase 2A 56 kDa regulatory subunit epsilon isoform OS=Homo sapiens OX=9606 GN=PPP2R!	0,457	2,543	1,185
P40925	Malate dehydrogenase, cytoplasmic OS=Homo sapiens OX=9606 GN=MDH1 PE=1 SV=4	0,508	2,096	1,179
E7EPK1	Septin OS=Homo sapiens OX=9606 GN=SEPTIN7 PE=1 SV=2	0,401	3,588	1,167
Q12959	Disks large homolog 1 OS=Homo sapiens OX=9606 GN=DLG1 PE=1 SV=2	2,24	0,528	1,164
Q9H2P0	Activity-dependent neuroprotector homeobox protein OS=Homo sapiens OX=9606 GN=ADNP PE=1 SV=1	0,375	2,91	1,163
Q01433	AMP deaminase 2 OS=Homo sapiens OX=9606 GN=AMPD2 PE=1 SV=3	0,539	4,16	1,157
O14497	AT-rich interactive domain-containing protein 1A OS=Homo sapiens OX=9606 GN=ARID1A PE=1 SV=3	0,437	2,38	1,155
Q96FW1	Ubiquitin thioesterase OTUB1 OS=Homo sapiens OX=9606 GN=OTUB1 PE=1 SV=2	0,423	2,875	1,152
P48643	T-complex protein 1 subunit epsilon OS=Homo sapiens OX=9606 GN=CCT5 PE=1 SV=1	0,606	2,021	1,152
O14656	Torsin-1A OS=Homo sapiens OX=9606 GN=TOR1A PE=1 SV=1	2,508	0,423	1,149
P68036	Ubiquitin-conjugating enzyme E2 L3 OS=Homo sapiens OX=9606 GN=UBE2L3 PE=1 SV=1	0,567	2,03	1,148
Q9NTZ6	RNA-binding protein 12 OS=Homo sapiens OX=9606 GN=RBM12 PE=1 SV=1	0,542	2,215	1,142
O00139	Kinesin-like protein KIF2A OS=Homo sapiens OX=9606 GN=KIF2A PE=1 SV=3	0,53	2,069	1,141
Q9Y2Q3	Glutathione S-transferase kappa 1 OS=Homo sapiens OX=9606 GN=GSTK1 PE=1 SV=3	8,118	0,106	1,14
P48147	Prolyl endopeptidase OS=Homo sapiens OX=9606 GN=PREP PE=1 SV=2	0,496	1,885	1,138

Q9UQ80	Proliferation-associated protein 2G4 OS=Homo sapiens OX=9606 GN=PA2G4 PE=1 SV=3	0,579	2,134	1,137
Q16513	Serine/threonine-protein kinase N2 OS=Homo sapiens OX=9606 GN=PKN2 PE=1 SV=1	0,473	2,914	1,134
Q460N5	Protein mono-ADP-ribosyltransferase PARP14 OS=Homo sapiens OX=9606 GN=PARP14 PE=1 SV=3	3,581	0,298	1,123
O15382	Branched-chain-amino-acid aminotransferase, mitochondrial OS=Homo sapiens OX=9606 GN=BCAT2 PE=1 SV=2	9,156	0,029	1,122
Q9BTT0	Acidic leucine-rich nuclear phosphoprotein 32 family member E OS=Homo sapiens OX=9606 GN=ANP32E PE=1 SV=1	0,418	2,491	1,119
Q15233	Non-POU domain-containing octamer-binding protein OS=Homo sapiens OX=9606 GN=NONO PE=1 SV=4	0,598	2,034	1,116
P31153	S-adenosylmethionine synthase isoform type-2 OS=Homo sapiens OX=9606 GN=MAT2A PE=1 SV=1	0,507	2,384	1,115
Q9P032	NADH dehydrogenase [ubiquinone] 1 alpha subcomplex assembly factor 4 OS=Homo sapiens OX=9606 GN=NDUFAF4 PE=1 S	0,855	2,254	1,113
Q6FI81	Anamorsin OS=Homo sapiens OX=9606 GN=CIAPIN1 PE=1 SV=2	0,469	3,448	1,111
O94901	SUN domain-containing protein 1 OS=Homo sapiens OX=9606 GN=SUN1 PE=1 SV=4	3,197	0,348	1,107
Q9Y305	Acyl-coenzyme A thioesterase 9, mitochondrial OS=Homo sapiens OX=9606 GN=ACOT9 PE=1 SV=2	2,27	0,531	1,106
Q92520	Protein FAM3C OS=Homo sapiens OX=9606 GN=FAM3C PE=1 SV=1	2,077	0,492	1,105
P27635	Large ribosomal subunit protein uL16 OS=Homo sapiens OX=9606 GN=RPL10 PE=1 SV=5	0,488	3,342	1,102
Q8IXQ6	Protein mono-ADP-ribosyltransferase PARP9 OS=Homo sapiens OX=9606 GN=PARP9 PE=1 SV=2	5,744	0,186	1,099
P20810	Calpastatin OS=Homo sapiens OX=9606 GN=CAST PE=1 SV=4	3,399	0,331	1,097
P12955	Xaa-Pro dipeptidase OS=Homo sapiens OX=9606 GN=PEPD PE=1 SV=3	0,709	2,374	1,092
P40121	Macrophage-capping protein OS=Homo sapiens OX=9606 GN=CAPG PE=1 SV=2	6,594	0,2	1,091
Q9UKA9	Polypyrimidine tract-binding protein 2 OS=Homo sapiens OX=9606 GN=PTBP2 PE=1 SV=1	0,06	14,792	1,089
Q16643	Drebrin OS=Homo sapiens OX=9606 GN=DBN1 PE=1 SV=4	0,341	3,295	1,082
Q9H4G0	Band 4.1-like protein 1 OS=Homo sapiens OX=9606 GN=EPB41L1 PE=1 SV=2	2,932	0,262	1,071
O00151	PDZ and LIM domain protein 1 OS=Homo sapiens OX=9606 GN=PDLIM1 PE=1 SV=4	4,019	0,279	1,07
Q14257	Reticulocalbin-2 OS=Homo sapiens OX=9606 GN=RCN2 PE=1 SV=1	0,264	4,063	1,066
P13797	Plastin-3 OS=Homo sapiens OX=9606 GN=PLS3 PE=1 SV=4	2,603	0,43	1,065
Q02790	Peptidyl-prolyl cis-trans isomerase FKBP4 OS=Homo sapiens OX=9606 GN=FKBP4 PE=1 SV=3	0,333	3,347	1,063
P43487	Ran-specific GTPase-activating protein OS=Homo sapiens OX=9606 GN=RANBP1 PE=1 SV=1	0,284	2,975	1,059
Q96N67	Dedicator of cytokinesis protein 7 OS=Homo sapiens OX=9606 GN=DOCK7 PE=1 SV=4	0,449	1,707	1,054
O95782	AP-2 complex subunit alpha-1 OS=Homo sapiens OX=9606 GN=AP2A1 PE=1 SV=3	2,161	0,516	1,05
Q15006	ER membrane protein complex subunit 2 OS=Homo sapiens OX=9606 GN=EMC2 PE=1 SV=1	2,084	0,514	1,05
Q9H4M9	EH domain-containing protein 1 OS=Homo sapiens OX=9606 GN=EHD1 PE=1 SV=2	2,783	0,403	1,05
P45973	Chromobox protein homolog 5 OS=Homo sapiens OX=9606 GN=CBX5 PE=1 SV=1	0,222	3,491	1,041
Q13242	Serine/arginine-rich splicing factor 9 OS=Homo sapiens OX=9606 GN=SRSF9 PE=1 SV=1	0,448	2,1	1,041
P09429	High mobility group protein B1 OS=Homo sapiens OX=9606 GN=HMGB1 PE=1 SV=3	0,404	2,567	1,04
P63241	Eukaryotic translation initiation factor 5A-1 OS=Homo sapiens OX=9606 GN=EIF5A PE=1 SV=2	0,27	3,008	1,039
E9PAV3	Nascent polypeptide-associated complex subunit alpha, muscle-specific form OS=Homo sapiens OX=9606 GN=NACA PE=1 SV	0,357	2,668	1,036
O15439	ATP-binding cassette sub-family C member 4 OS=Homo sapiens OX=9606 GN=ABCC4 PE=1 SV=3	2,572	0,331	1,032
Q15149	Plectin OS=Homo sapiens OX=9606 GN=PLEC PE=1 SV=3	3,298	0,359	1,028
Q9UH99	SUN domain-containing protein 2 OS=Homo sapiens OX=9606 GN=SUN2 PE=1 SV=3	2,362	0,438	1,027
Q6UXN9	WD repeat-containing protein 82 OS=Homo sapiens OX=9606 GN=WDR82 PE=1 SV=1	0,673	2,057	1,022
Q8TDB6	E3 ubiquitin-protein ligase DTX3L OS=Homo sapiens OX=9606 GN=DTX3L PE=1 SV=1	2,793	0,613	1,016
P61254	Large ribosomal subunit protein uL24 OS=Homo sapiens OX=9606 GN=RPL26 PE=1 SV=1	0,518	2,196	1,014
O00159	Unconventional myosin-Ic OS=Homo sapiens OX=9606 GN=MYO1C PE=1 SV=4	4,545	0,229	1,009
P31350	Ribonucleoside-diphosphate reductase subunit M2 OS=Homo sapiens OX=9606 GN=RRM2 PE=1 SV=1	0,453	1,809	1,006
P13804	Electron transfer flavoprotein subunit alpha, mitochondrial OS=Homo sapiens OX=9606 GN=ETFA PE=1 SV=1	3,194	0,3	1,006
Q8TDI0	Chromodomain-helicase-DNA-binding protein 5 OS=Homo sapiens OX=9606 GN=CHD5 PE=1 SV=1	0,37	3,277	1,004
P49321	Nuclear autoantigenic sperm protein OS=Homo sapiens OX=9606 GN=NASP PE=1 SV=2	0,323	2,788	0,999
P20290	Transcription factor BTF3 OS=Homo sapiens OX=9606 GN=BTF3 PE=1 SV=1	0,507	2,388	0,999
Q08J23	RNA cytosine C(5)-methyltransferase NSUN2 OS=Homo sapiens OX=9606 GN=NSUN2 PE=1 SV=2	0,457	2,277	0,998
P51003	Poly(A) polymerase alpha OS=Homo sapiens OX=9606 GN=PAPOLA PE=1 SV=4	0,326	2,665	0,997
Q8NF37	Lysophosphatidylcholine acyltransferase 1 OS=Homo sapiens OX=9606 GN=LPCAT1 PE=1 SV=2	0,252	3,545	0,994
O15173	Membrane-associated progesterone receptor component 2 OS=Homo sapiens OX=9606 GN=PGRMC2 PE=1 SV=1	2,441	0,413	0,992
P02649	Apolipoprotein E OS=Homo sapiens OX=9606 GN=APOE PE=1 SV=1	0,213	3,098	0,99
Q9Y4F1	FERM, ARHGEF and pleckstrin domain-containing protein 1 OS=Homo sapiens OX=9606 GN=FARP1 PE=1 SV=1	0,455	1,278	0,985
Q9UL46	Proteasome activator complex subunit 2 OS=Homo sapiens OX=9606 GN=PSME2 PE=1 SV=4	3,075	0,291	0,985
Q7L2H7	Eukaryotic translation initiation factor 3 subunit M OS=Homo sapiens OX=9606 GN=EIF3M PE=1 SV=1	0,494	1,481	0,973
P12814	Alpha-actinin-1 OS=Homo sapiens OX=9606 GN=ACTN1 PE=1 SV=2	2,251	0,486	0,971
Q9UP95	Solute carrier family 12 member 4 OS=Homo sapiens OX=9606 GN=SLC12A4 PE=1 SV=2	2,095	0,403	0,968
Q9UBB4	Ataxin-10 OS=Homo sapiens OX=9606 GN=ATXN10 PE=1 SV=1	0,35	2,658	0,966
Q13751	Laminin subunit beta-3 OS=Homo sapiens OX=9606 GN=LAMB3 PE=1 SV=1	3,884	0,237	0,965
Q9HB71	Calcyclin-binding protein OS=Homo sapiens OX=9606 GN=CACYBP PE=1 SV=2	0,352	2,286	0,962
P06865	Beta-hexosaminidase subunit alpha OS=Homo sapiens OX=9606 GN=HEXA PE=1 SV=2	2,194	0,489	0,962
Q99733	Nucleosome assembly protein 1-like 4 OS=Homo sapiens OX=9606 GN=NAP1L4 PE=1 SV=1	0,34	1,424	0,956
P38117	Electron transfer flavoprotein subunit beta OS=Homo sapiens OX=9606 GN=ETFB PE=1 SV=3	3,456	0,281	0,953
J3KQL8	Apolipoprotein L2 OS=Homo sapiens OX=9606 GN=APOL2 PE=1 SV=2	6,574	0,243	0,951
P23921	Ribonucleoside-diphosphate reductase large subunit OS=Homo sapiens OX=9606 GN=RRM1 PE=1 SV=1	0,321	3,619	0,948
Q8IXT5	RNA-binding protein 12B OS=Homo sapiens OX=9606 GN=RBM12B PE=1 SV=2	0,346	2,248	0,948
Q13439	Golgin subfamily A member 4 OS=Homo sapiens OX=9606 GN=GOLGA4 PE=1 SV=1	1,611	0,434	0,947
Q96QC0	Serine/threonine-protein phosphatase 1 regulatory subunit 10 OS=Homo sapiens OX=9606 GN=PPP1R10 PE=1 SV=1	0,494	1,659	0,943
Q00577	Transcriptional activator protein Pur-alpha OS=Homo sapiens OX=9606 GN=PURA PE=1 SV=2	2,753	0,266	0,941
P24941	Cyclin-dependent kinase 2 OS=Homo sapiens OX=9606 GN=CDK2 PE=1 SV=2	0,49	2,109	0,94
P11172	Uridine 5'-monophosphate synthase OS=Homo sapiens OX=9606 GN=UMPS PE=1 SV=1	0,461	2,067	0,94
Q9H223	EH domain-containing protein 4 OS=Homo sapiens OX=9606 GN=EHD4 PE=1 SV=1	2,436	0,421	0,938
Q92878	DNA repair protein RAD50 OS=Homo sapiens OX=9606 GN=RAD50 PE=1 SV=1	2,46	0,367	0,937
Q5M775	Cytospin-B OS=Homo sapiens OX=9606 GN=SPECC1 PE=1 SV=1	2,58	0,367	0,931
Q00169	Phosphatidylinositol transfer protein alpha isoform OS=Homo sapiens OX=9606 GN=PITPNA PE=1 SV=2	0,325	3,236	0,929
O00571	ATP-dependent RNA helicase DDX3X OS=Homo sapiens OX=9606 GN=DDX3X PE=1 SV=3	0,479	1,615	0,927
P20592	Interferon-induced GTP-binding protein Mx2 OS=Homo sapiens OX=9606 GN=MX2 PE=1 SV=1	0,985	0,466	0,927
Q06323	Proteasome activator complex subunit 1 OS=Homo sapiens OX=9606 GN=PSME1 PE=1 SV=1	4,152	0,217	0,927
P32119	Peroxiredoxin-2 OS=Homo sapiens OX=9606 GN=PRDX2 PE=1 SV=5	0,488	1,852	0,925
Q15631	Translin OS=Homo sapiens OX=9606 GN=TSN PE=1 SV=1	0,44	2,106	0,924
P22234	Bifunctional phosphoribosylaminoimidazole carboxylase/phosphoribosylaminoimidazole succinocarboxamide synthetase OS=	0,362	2,635	0,919
P05362	Intercellular adhesion molecule 1 OS=Homo sapiens OX=9606 GN=ICAM1 PE=1 SV=2	12,547	0,071	0,917
P30566	Adenylosuccinate lyase OS=Homo sapiens OX=9606 GN=ADSL PE=1 SV=2	0,21	3,731	0,916

Q92499	ATP-dependent RNA helicase DDX1 OS=Homo sapiens OX=9606 GN=DDX1 PE=1 SV=2	0,482	1,899	0,916
O00186	Syntaxin-binding protein 3 OS=Homo sapiens OX=9606 GN=STXBP3 PE=1 SV=2	3,307	0,286	0,912
Q96JM3	Chromosome alignment-maintaining phosphoprotein 1 OS=Homo sapiens OX=9606 GN=CHAMP1 PE=1 SV=2	0,489	1,892	0,906
Q15067	Peroxisomal acyl-coenzyme A oxidase 1 OS=Homo sapiens OX=9606 GN=ACOX1 PE=1 SV=3	2,157	0,432	0,905
O43707	Alpha-actinin-4 OS=Homo sapiens OX=9606 GN=ACTN4 PE=1 SV=2	2,634	0,344	0,905
P42126	Enoyl-CoA delta isomerase 1, mitochondrial OS=Homo sapiens OX=9606 GN=ECI1 PE=1 SV=1	2,471	0,379	0,901
P20674	Cytochrome c oxidase subunit 5A, mitochondrial OS=Homo sapiens OX=9606 GN=COX5A PE=1 SV=2	0,324	2,017	0,897
O43252	Bifunctional 3'-phosphoadenosine 5'-phosphosulfate synthase 1 OS=Homo sapiens OX=9606 GN=PAPSS1 PE=1 SV=2	0,365	2,302	0,896
O00264	Membrane-associated progesterone receptor component 1 OS=Homo sapiens OX=9606 GN=PGRMC1 PE=1 SV=3	0,265	3,455	0,895
P04439	HLA class I histocompatibility antigen, A alpha chain OS=Homo sapiens OX=9606 GN=HLA-A PE=1 SV=2	13,48	0,073	0,895
Q9Y4K1	Beta/gamma crystallin domain-containing protein 1 OS=Homo sapiens OX=9606 GN=CRYBG1 PE=1 SV=3	2,383		0,888
P21980	Protein-glutamine gamma-glutamyltransferase 2 OS=Homo sapiens OX=9606 GN=TGM2 PE=1 SV=2	3,968	0,222	0,884
O75369	Filamin-B OS=Homo sapiens OX=9606 GN=FLNB PE=1 SV=2	4,382	0,192	0,884
A0A0D9SF	RNA helicase OS=Homo sapiens OX=9606 GN=DDX3X PE=1 SV=1	0,394	1,935	0,882
P30519	Heme oxygenase 2 OS=Homo sapiens OX=9606 GN=HMOX2 PE=1 SV=2	2,58	0,33	0,88
Q9Y2Z0	Protein SGT1 homolog OS=Homo sapiens OX=9606 GN=SUGT1 PE=1 SV=3	0,349	2,265	0,871
Q14108	Lysosome membrane protein 2 OS=Homo sapiens OX=9606 GN=SCARB2 PE=1 SV=2	2,545	0,349	0,871
Q5T7N2	LINE-1 type transposase domain-containing protein 1 OS=Homo sapiens OX=9606 GN=L1TD1 PE=1 SV=1	0,07	10,954	0,866
P62826	GTP-binding nuclear protein Ran OS=Homo sapiens OX=9606 GN=RAN PE=1 SV=3	0,378	2,437	0,864
Q27J81	Inverted formin-2 OS=Homo sapiens OX=9606 GN=INF2 PE=1 SV=2	3,65	0,227	0,862
Q15056	Eukaryotic translation initiation factor 4H OS=Homo sapiens OX=9606 GN=EIF4H PE=1 SV=5	0,42	1,677	0,861
P40616	ADP-ribosylation factor-like protein 1 OS=Homo sapiens OX=9606 GN=ARL1 PE=1 SV=1	2,125	0,371	0,861
P16278	Beta-galactosidase OS=Homo sapiens OX=9606 GN=GLB1 PE=1 SV=2	2,476	0,372	0,86
Q9BQ39	ATP-dependent RNA helicase DDX50 OS=Homo sapiens OX=9606 GN=DDX50 PE=1 SV=1	0,414	2,001	0,858
Q96HE7	ERO1-like protein alpha OS=Homo sapiens OX=9606 GN=ERO1A PE=1 SV=2	2,995	0,289	0,858
Q9BS26	Endoplasmic reticulum resident protein 44 OS=Homo sapiens OX=9606 GN=ERP44 PE=1 SV=1	2,745	0,311	0,857
Q9UKG9	Peroxisomal carnitine O-octanoyltransferase OS=Homo sapiens OX=9606 GN=CROT PE=1 SV=2	2,56	0,299	0,853
Q16822	Phosphoenolpyruvate carboxykinase [GTP], mitochondrial OS=Homo sapiens OX=9606 GN=PCK2 PE=1 SV=4	3,407	0,245	0,852
Q9Y266	Nuclear migration protein nudC OS=Homo sapiens OX=9606 GN=NUDC PE=1 SV=1	0,362	2,581	0,848
Q9BYT8	Neurolysin, mitochondrial OS=Homo sapiens OX=9606 GN=NLN PE=1 SV=1	0,385	2,423	0,845
Q6PIU2	Neutral cholesterol ester hydrolase 1 OS=Homo sapiens OX=9606 GN=NCEH1 PE=1 SV=3	7,037	0,119	0,845
P09211	Glutathione S-transferase P OS=Homo sapiens OX=9606 GN=GSTP1 PE=1 SV=2	0,19	4,633	0,842
Q9BYK8	3'-5' exoribonuclease HELZ2 OS=Homo sapiens OX=9606 GN=HELZ2 PE=1 SV=7	12,543	0,079	0,842
P22102	Trifunctional purine biosynthetic protein adenosine-3 OS=Homo sapiens OX=9606 GN=GART PE=1 SV=1	0,379	2,38	0,841
Q96T88	E3 ubiquitin-protein ligase UHRF1 OS=Homo sapiens OX=9606 GN=UHRF1 PE=1 SV=1	0,402	2,139	0,84
Q9H553	Alpha-1,3/1,6-mannosyltransferase ALG2 OS=Homo sapiens OX=9606 GN=ALG2 PE=1 SV=1	4,221	0,189	0,839
Q15102	Platelet-activating factor acetylhydrolase IB subunit alpha1 OS=Homo sapiens OX=9606 GN=PAFAH1B3 PE=1 SV=1	0,334	2,238	0,837
Q13085	Acetyl-CoA carboxylase 1 OS=Homo sapiens OX=9606 GN=ACACA PE=1 SV=2	0,266	4,605	0,836
P51531	Probable global transcription activator SNF2L2 OS=Homo sapiens OX=9606 GN=SMARCA2 PE=1 SV=2	0,383	2,988	0,836
P80217	Interferon-induced 35 kDa protein OS=Homo sapiens OX=9606 GN=IFI35 PE=1 SV=5	1000	0,001	0,836
Q96RP9	Elongation factor G, mitochondrial OS=Homo sapiens OX=9606 GN=GFM1 PE=1 SV=2	1,822	0,45	0,833
Q9P2D1	Chromodomain-helicase-DNA-binding protein 7 OS=Homo sapiens OX=9606 GN=CHD7 PE=1 SV=3	0,23	2,556	0,831
P83111	Serine beta-lactamase-like protein LACTB, mitochondrial OS=Homo sapiens OX=9606 GN=LACTB PE=1 SV=2	4,013	0,224	0,83
P19525	Interferon-induced, double-stranded RNA-activated protein kinase OS=Homo sapiens OX=9606 GN=EIF2AK2 PE=1 SV=2	1,899	0,412	0,828
Q15005	Signal peptidase complex subunit 2 OS=Homo sapiens OX=9606 GN=SPCS2 PE=1 SV=3	1,595	0,476	0,821
O15031	Plexin-B2 OS=Homo sapiens OX=9606 GN=PLXNB2 PE=1 SV=3	1,935	0,467	0,82
Q13151	Heterogeneous nuclear ribonucleoprotein A0 OS=Homo sapiens OX=9606 GN=HNRNPA0 PE=1 SV=1	0,344	1,935	0,818
P67812	Signal peptidase complex catalytic subunit SEC11A OS=Homo sapiens OX=9606 GN=SEC11A PE=1 SV=1	1,641	0,439	0,817
A0AV96	RNA-binding protein 47 OS=Homo sapiens OX=9606 GN=RBM47 PE=1 SV=2	1,094	0,474	0,813
P08195	Amino acid transporter heavy chain SLC3A2 OS=Homo sapiens OX=9606 GN=SLC3A2 PE=1 SV=3	4,227	0,211	0,812
Q15691	Microtubule-associated protein RP/EB family member 1 OS=Homo sapiens OX=9606 GN=MAPRE1 PE=1 SV=3	0,481	2,007	0,809
P23588	Eukaryotic translation initiation factor 4B OS=Homo sapiens OX=9606 GN=EIF4B PE=1 SV=2	0,458	2,324	0,806
Q14676	Mediator of DNA damage checkpoint protein 1 OS=Homo sapiens OX=9606 GN=MDC1 PE=1 SV=3	0,328	2,327	0,805
O14744	Protein arginine N-methyltransferase 5 OS=Homo sapiens OX=9606 GN=PRMT5 PE=1 SV=4	0,409	1,946	0,8
Q8WVX9	Fatty acyl-CoA reductase 1 OS=Homo sapiens OX=9606 GN=FAR1 PE=1 SV=1	0,265	3,137	0,798
Q5JTV8	Torsin-1A-interacting protein 1 OS=Homo sapiens OX=9606 GN=TOR1AIP1 PE=1 SV=2	2,49	0,332	0,796
P31939	Bifunctional purine biosynthesis protein ATIC OS=Homo sapiens OX=9606 GN=ATIC PE=1 SV=3	0,336	2,161	0,794
Q99986	Serine/threonine-protein kinase VRK1 OS=Homo sapiens OX=9606 GN=VRK1 PE=1 SV=1	0,418	1,381	0,794
P23193	Transcription elongation factor A protein 1 OS=Homo sapiens OX=9606 GN=TCEA1 PE=1 SV=2	0,475	1,63	0,79
Q8N1G4	Leucine-rich repeat-containing protein 47 OS=Homo sapiens OX=9606 GN=LRRC47 PE=1 SV=1	0,413	2,025	0,787
P35580	Myosin-10 OS=Homo sapiens OX=9606 GN=MYH10 PE=1 SV=3	0,225	3,489	0,786
P62266	Small ribosomal subunit protein uS12 OS=Homo sapiens OX=9606 GN=RPS23 PE=1 SV=3	0,272	2,808	0,786
P07384	Calpain-1 catalytic subunit OS=Homo sapiens OX=9606 GN=CAPN1 PE=1 SV=1	1,667	0,451	0,781
P28074	Proteasome subunit beta type-5 OS=Homo sapiens OX=9606 GN=PSMB5 PE=1 SV=3	0,263	2,741	0,78
Q9BQE5	Apolipoprotein L2 OS=Homo sapiens OX=9606 GN=APOL2 PE=1 SV=2	10,274	0,116	0,78
P09110	3-ketoacyl-CoA thiolase, peroxisomal OS=Homo sapiens OX=9606 GN=ACAA1 PE=1 SV=2	2,44	0,336	0,779
O75534	Cold shock domain-containing protein E1 OS=Homo sapiens OX=9606 GN=CSDE1 PE=1 SV=2	0,38	2,024	0,776
P27695	DNA-(apurinic or apyrimidinic site) endonuclease OS=Homo sapiens OX=9606 GN=APEX1 PE=1 SV=2	0,379	2,682	0,774
Q96K17	Transcription factor BTF3 homolog 4 OS=Homo sapiens OX=9606 GN=BTF3L4 PE=1 SV=1	0,447	2,369	0,774
O75746	Electrogenic aspartate/glutamate antiporter SLC25A12, mitochondrial OS=Homo sapiens OX=9606 GN=SLC25A12 PE=1 SV=2	2,032	0,422	0,774
Q15365	Poly(rC)-binding protein 1 OS=Homo sapiens OX=9606 GN=PCBP1 PE=1 SV=2	0,486	1,479	0,773
Q13813	Spectrin alpha chain, non-erythrocytic 1 OS=Homo sapiens OX=9606 GN=SPTAN1 PE=1 SV=3	1,599	0,48	0,772
Q9NYL9	Tropomodulin-3 OS=Homo sapiens OX=9606 GN=TMOD3 PE=1 SV=1	1,997	0,469	0,77
Q9UBQ7	Glyoxylate reductase/hydroxypyruvate reductase OS=Homo sapiens OX=9606 GN=GRHPR PE=1 SV=1	1,34	0,402	0,77
Q9BTX1	Nucleoporin NDC1 OS=Homo sapiens OX=9606 GN=NDC1 PE=1 SV=2	0,483	1,038	0,767
P50579	Methionine aminopeptidase 2 OS=Homo sapiens OX=9606 GN=METAP2 PE=1 SV=1	0,34	2,295	0,766
P31948	Stress-induced-phosphoprotein 1 OS=Homo sapiens OX=9606 GN=STIP1 PE=1 SV=1	0,443	1,735	0,761
Q9BWD1	Acetyl-CoA acetyltransferase, cytosolic OS=Homo sapiens OX=9606 GN=ACAT2 PE=1 SV=2	0,151	5,348	0,759
Q9NSE4	Isoleucine--tRNA ligase, mitochondrial OS=Homo sapiens OX=9606 GN=IARS2 PE=1 SV=2	1,68	0,487	0,758
P07900	Heat shock protein HSP 90-alpha OS=Homo sapiens OX=9606 GN=HSP90AA1 PE=1 SV=5	0,356	1,88	0,756
O00273	DNA fragmentation factor subunit alpha OS=Homo sapiens OX=9606 GN=DFFA PE=1 SV=1	0,173	2,91	0,755
Q15785	Mitochondrial import receptor subunit TOM34 OS=Homo sapiens OX=9606 GN=TOMM34 PE=1 SV=2	0,281	2,399	0,755

Q9BWF3	RNA-binding protein 4 OS=Homo sapiens OX=9606 GN=RBM4 PE=1 SV=1	0,466	1,684	0,755
Q16850	Lanosterol 14-alpha demethylase OS=Homo sapiens OX=9606 GN=CYP51A1 PE=1 SV=4	0,203	3,368	0,752
P00533	Epidermal growth factor receptor OS=Homo sapiens OX=9606 GN=EGFR PE=1 SV=2	2,78	0,276	0,751
Q12846	Syntaxin-4 OS=Homo sapiens OX=9606 GN=STX4 PE=1 SV=2	3,727	0,247	0,747
Q6P179	Endoplasmic reticulum aminopeptidase 2 OS=Homo sapiens OX=9606 GN=ERAP2 PE=1 SV=2			0,742
Q14789	Golgin subfamily B member 1 OS=Homo sapiens OX=9606 GN=GOLGB1 PE=1 SV=2	1,653	0,471	0,742
O94903	Pyridoxal phosphate homeostasis protein OS=Homo sapiens OX=9606 GN=PLPBP PE=1 SV=1	0,498	1,376	0,74
O43719	17S U2 SnRNP complex component HTATSF1 OS=Homo sapiens OX=9606 GN=HTATSF1 PE=1 SV=1	0,453	1,877	0,732
P62258	14-3-3 protein epsilon OS=Homo sapiens OX=9606 GN=YWHAE PE=1 SV=1	0,349	1,984	0,73
Q9Y3Y2	Chromatin target of PRMT1 protein OS=Homo sapiens OX=9606 GN=CHTOP PE=1 SV=2	0,491	0,935	0,727
Q9BYD6	Large ribosomal subunit protein uL1m OS=Homo sapiens OX=9606 GN=MRPL1 PE=1 SV=2	1,501	0,442	0,726
Q14839	Chromodomain-helicase-DNA-binding protein 4 OS=Homo sapiens OX=9606 GN=CHD4 PE=1 SV=2	0,381	1,711	0,725
Q8IY21	Probable ATP-dependent RNA helicase DDX60 OS=Homo sapiens OX=9606 GN=DDX60 PE=1 SV=3	2,628	0,239	0,725
Q8TCS8	Polyribonucleotide nucleotidyltransferase 1, mitochondrial OS=Homo sapiens OX=9606 GN=PNPT1 PE=1 SV=2	1,642	0,392	0,72
Q9H9P8	L-2-hydroxyglutarate dehydrogenase, mitochondrial OS=Homo sapiens OX=9606 GN=L2HGDH PE=1 SV=3	1,487	0,477	0,719
Q7Z460	CLIP-associating protein 1 OS=Homo sapiens OX=9606 GN=CLASP1 PE=1 SV=1	0,364	2,099	0,718
P14314	Glucosidase 2 subunit beta OS=Homo sapiens OX=9606 GN=PRKCSH PE=1 SV=2	1,695	0,393	0,717
P34896	Serine hydroxymethyltransferase, cytosolic OS=Homo sapiens OX=9606 GN=SHMT1 PE=1 SV=1	0,379	1,941	0,714
P62269	Small ribosomal subunit protein uS13 OS=Homo sapiens OX=9606 GN=RPS18 PE=1 SV=3	0,387	1,491	0,714
Q9UJZ1	Stomatin-like protein 2, mitochondrial OS=Homo sapiens OX=9606 GN=STOML2 PE=1 SV=1	1,653	0,409	0,713
P62829	Large ribosomal subunit protein uL14 OS=Homo sapiens OX=9606 GN=RPL23 PE=1 SV=1	0,381	1,915	0,712
P11387	DNA topoisomerase 1 OS=Homo sapiens OX=9606 GN=TOP1 PE=1 SV=2	0,434	1,657	0,712
P26196	Probable ATP-dependent RNA helicase DDX6 OS=Homo sapiens OX=9606 GN=DDX6 PE=1 SV=2	0,488	1,628	0,711
O15270	Serine palmitoyltransferase 2 OS=Homo sapiens OX=9606 GN=SPTLC2 PE=1 SV=1	1,315	0,499	0,71
Q13309	S-phase kinase-associated protein 2 OS=Homo sapiens OX=9606 GN=SKP2 PE=1 SV=2	0,114	9,283	0,705
Q6UB35	Monofunctional C1-tetrahydrofolate synthase, mitochondrial OS=Homo sapiens OX=9606 GN=MTHFD1L PE=1 SV=1	1,593	0,482	0,701
Q8TEX9	Importin-4 OS=Homo sapiens OX=9606 GN=IPO4 PE=1 SV=2	0,289	2,92	0,695
Q00688	Peptidyl-prolyl cis-trans isomerase FKBP3 OS=Homo sapiens OX=9606 GN=FKBP3 PE=1 SV=1	0,421	1,562	0,692
Q9Y5L0	Transportin-3 OS=Homo sapiens OX=9606 GN=TNPO3 PE=1 SV=3	0,352	1,741	0,691
Q96G03	Phosphopentomutase OS=Homo sapiens OX=9606 GN=PGM2 PE=1 SV=4	0,42	1,504	0,69
Q9HCC0	Methylcrotonoyl-CoA carboxylase beta chain, mitochondrial OS=Homo sapiens OX=9606 GN=MCCC2 PE=1 SV=1	1,878	0,4	0,69
Q96I99	Succinate--CoA ligase [GDP-forming] subunit beta, mitochondrial OS=Homo sapiens OX=9606 GN=SUCLG2 PE=1 SV=2	2,071	0,317	0,69
P24666	Low molecular weight phosphotyrosine protein phosphatase OS=Homo sapiens OX=9606 GN=ACP1 PE=1 SV=3	0,252	2,901	0,687
Q9Y2W2	WW domain-binding protein 11 OS=Homo sapiens OX=9606 GN=WBP11 PE=1 SV=1	0,479	1,379	0,687
P29590	Protein PML OS=Homo sapiens OX=9606 GN=PML PE=1 SV=3	6,816	0,134	0,687
P62899	Large ribosomal subunit protein eL31 OS=Homo sapiens OX=9606 GN=RPL31 PE=1 SV=1	0,347	2,976	0,686
Q9NPL8	Complex I assembly factor TIMMDC1, mitochondrial OS=Homo sapiens OX=9606 GN=TIMMDC1 PE=1 SV=2	1,438	0,442	0,686
P62701	Small ribosomal subunit protein eS4, X isoform OS=Homo sapiens OX=9606 GN=RPS4X PE=1 SV=2	0,379	1,685	0,685
Q96JB5	CDK5 regulatory subunit-associated protein 3 OS=Homo sapiens OX=9606 GN=CDK5RAP3 PE=1 SV=2	1,833	0,448	0,685
Q8IXI1	Mitochondrial Rho GTPase 2 OS=Homo sapiens OX=9606 GN=RHOT2 PE=1 SV=2	1,328	0,483	0,681
Q13057	Bifunctional coenzyme A synthase OS=Homo sapiens OX=9606 GN=COASY PE=1 SV=4	1,89	0,392	0,681
Q9Y5X3	Sorting nexin-5 OS=Homo sapiens OX=9606 GN=SNX5 PE=1 SV=1	0,257	2,235	0,68
Q15031	Leucine--tRNA ligase, mitochondrial OS=Homo sapiens OX=9606 GN=LARS2 PE=1 SV=2	1,531	0,397	0,68
Q9NZB2	Constitutive coactivator of PPAR-gamma-like protein 1 OS=Homo sapiens OX=9606 GN=FAM120A PE=1 SV=2	3,025	0,251	0,68
O94874	E3 UFM1-protein ligase 1 OS=Homo sapiens OX=9606 GN=UFL1 PE=1 SV=2	1,436	0,391	0,679
Q9P258	Protein RCC2 OS=Homo sapiens OX=9606 GN=RCC2 PE=1 SV=2	0,091	2,847	0,676
P23229	Integrin alpha-6 OS=Homo sapiens OX=9606 GN=ITGA6 PE=1 SV=5	1,656	0,416	0,676
P00491	Purine nucleoside phosphorylase OS=Homo sapiens OX=9606 GN=PNP PE=1 SV=2	0,379	2,234	0,673
Q9P0M6	Core histone macro-H2A.2 OS=Homo sapiens OX=9606 GN=MACROH2A2 PE=1 SV=3	0,296	2,218	0,671
Q9Y2R0	Cytochrome c oxidase assembly factor 3 homolog, mitochondrial OS=Homo sapiens OX=9606 GN=COA3 PE=1 SV=1	1,975	0,413	0,669
Q5K651	Sterile alpha motif domain-containing protein 9 OS=Homo sapiens OX=9606 GN=SAMD9 PE=1 SV=1	2,934	0,075	0,669
Q9BQ95	Evolutionarily conserved signaling intermediate in Toll pathway, mitochondrial OS=Homo sapiens OX=9606 GN=ECSIT PE=1 S	1,263	0,477	0,667
P51532	Transcription activator BRG1 OS=Homo sapiens OX=9606 GN=SMARCA4 PE=1 SV=2	0,356	2,094	0,666
P23497	Nuclear autoantigen Sp-100 OS=Homo sapiens OX=9606 GN=SP100 PE=1 SV=3	0,505	0,37	0,659
Q01581	Hydroxymethylglutaryl-CoA synthase, cytoplasmic OS=Homo sapiens OX=9606 GN=HMGCS1 PE=1 SV=2	0,026	20,276	0,657
Q08380	Galectin-3-binding protein OS=Homo sapiens OX=9606 GN=LGALS3BP PE=1 SV=1	8,67	0,116	0,657
P19634	Sodium/hydrogen exchanger 1 OS=Homo sapiens OX=9606 GN=SLC9A1 PE=1 SV=2	2,31	0,353	0,656
Q9Y320	Thioredoxin-related transmembrane protein 2 OS=Homo sapiens OX=9606 GN=TMX2 PE=1 SV=1	1,682	0,41	0,655
Q96DB5	Regulator of microtubule dynamics protein 1 OS=Homo sapiens OX=9606 GN=RMDN1 PE=1 SV=1	1,813	0,342	0,655
P55060	Exportin-2 OS=Homo sapiens OX=9606 GN=CSE1L PE=1 SV=3	0,433	1,386	0,654
Q9H7Z7	Prostaglandin E synthase 2 OS=Homo sapiens OX=9606 GN=PTGES2 PE=1 SV=1	1,784	0,278	0,652
Q9Y4P3	Transducin beta-like protein 2 OS=Homo sapiens OX=9606 GN=TBL2 PE=1 SV=1	1,304	0,397	0,651
P23526	Adenosylhomocysteinase OS=Homo sapiens OX=9606 GN=AHCY PE=1 SV=4	0,311	2,05	0,649
Q9P015	Large ribosomal subunit protein uL15m OS=Homo sapiens OX=9606 GN=MRPL15 PE=1 SV=1	0,978	0,469	0,648
P17677	Neuromodulin OS=Homo sapiens OX=9606 GN=GAP43 PE=1 SV=1	0,041	7,189	0,647
P13796	Plastin-2 OS=Homo sapiens OX=9606 GN=LCP1 PE=1 SV=6	1,161	0,312	0,647
P51589	Cytochrome P450 2J2 OS=Homo sapiens OX=9606 GN=CYP2J2 PE=1 SV=2	0,402	1,605	0,646
Q02543	Large ribosomal subunit protein eL20 OS=Homo sapiens OX=9606 GN=RPL18A PE=1 SV=2	0,445	1,567	0,646
O00330	Pyruvate dehydrogenase protein X component, mitochondrial OS=Homo sapiens OX=9606 GN=PDHX PE=1 SV=3	1,278	0,404	0,644
P05455	Lupus La protein OS=Homo sapiens OX=9606 GN=SSB PE=1 SV=2	0,429	1,496	0,642
P54819	Adenylate kinase 2, mitochondrial OS=Homo sapiens OX=9606 GN=AK2 PE=1 SV=2	1,972	0,309	0,641
P48681	Nestin OS=Homo sapiens OX=9606 GN=NES PE=1 SV=2	0,135	5,837	0,638
P41219	Peripherin OS=Homo sapiens OX=9606 GN=PRPH PE=1 SV=2	0,067	1,514	0,637
P49327	Fatty acid synthase OS=Homo sapiens OX=9606 GN=FASN PE=1 SV=3	0,096	6,476	0,636
P08670	Vimentin OS=Homo sapiens OX=9606 GN=VIM PE=1 SV=4	0,035	30,806	0,635
Q9Y4W6	AFG3-like protein 2 OS=Homo sapiens OX=9606 GN=AFG3L2 PE=1 SV=2	1,4	0,416	0,633
Q13330	Metastasis-associated protein MTA1 OS=Homo sapiens OX=9606 GN=MTA1 PE=1 SV=2	0,339	1,848	0,632
Q6PKG0	La-related protein 1 OS=Homo sapiens OX=9606 GN=LARP1 PE=1 SV=2	0,418	1,106	0,629
P10253	Lysosomal alpha-glucosidase OS=Homo sapiens OX=9606 GN=GAA PE=1 SV=4	0,995	0,306	0,626
Q02880	DNA topoisomerase 2-beta OS=Homo sapiens OX=9606 GN=TOP2B PE=1 SV=3	0,283	2,149	0,625
P21127	Cyclin-dependent kinase 11B OS=Homo sapiens OX=9606 GN=CDK11B PE=1 SV=4	0,212	0,967	0,621
Q6NUQ4	Transmembrane protein 214 OS=Homo sapiens OX=9606 GN=TMEM214 PE=1 SV=2	1,326	0,443	0,62

O75569	Interferon-inducible double-stranded RNA-dependent protein kinase activator A OS=Homo sapiens OX=9606 GN=PRKRA PE=	0,361	1,683	0,619
Q9BRJ2	Large ribosomal subunit protein mL45 OS=Homo sapiens OX=9606 GN=MRPL45 PE=1 SV=2	1,292	0,44	0,619
Q99613	Eukaryotic translation initiation factor 3 subunit C OS=Homo sapiens OX=9606 GN=EIF3C PE=1 SV=1	0,498	1,198	0,618
O75356	Nucleoside diphosphate phosphatase ENTPD5 OS=Homo sapiens OX=9606 GN=ENTPD5 PE=1 SV=1	2,366	0,288	0,617
Q9H2W6	Large ribosomal subunit protein mL46 OS=Homo sapiens OX=9606 GN=MRPL46 PE=1 SV=1	1,592	0,369	0,616
P43246	DNA mismatch repair protein Msh2 OS=Homo sapiens OX=9606 GN=MSH2 PE=1 SV=1	0,278	1,666	0,615
Q15020	Squamous cell carcinoma antigen recognized by T-cells 3 OS=Homo sapiens OX=9606 GN=SART3 PE=1 SV=1	0,463	1,258	0,615
Q9NTK5	Obg-like ATPase 1 OS=Homo sapiens OX=9606 GN=OLA1 PE=1 SV=2	0,262	2,155	0,614
O15355	Protein phosphatase 1G OS=Homo sapiens OX=9606 GN=PPM1G PE=1 SV=1	0,285	2,153	0,613
Q96SB4	SRSF protein kinase 1 OS=Homo sapiens OX=9606 GN=SRPK1 PE=1 SV=2	0,297	2,775	0,612
P33991	DNA replication licensing factor MCM4 OS=Homo sapiens OX=9606 GN=MCM4 PE=1 SV=5	0,48	1,318	0,612
Q8N5N7	Large ribosomal subunit protein mL50 OS=Homo sapiens OX=9606 GN=MRPL50 PE=1 SV=2	1,732	0,435	0,608
Q9BX40	Protein LSM14 homolog B OS=Homo sapiens OX=9606 GN=LSM14B PE=1 SV=1	0,405	1,525	0,599
P82650	Small ribosomal subunit protein mS22 OS=Homo sapiens OX=9606 GN=MRPS22 PE=1 SV=1	1,192	0,469	0,599
P62750	Large ribosomal subunit protein uL23 OS=Homo sapiens OX=9606 GN=RPL23A PE=1 SV=1	0,357	1,691	0,594
Q53GS9	Ubiquitin carboxyl-terminal hydrolase 39 OS=Homo sapiens OX=9606 GN=USP39 PE=1 SV=2	0,45	1,227	0,594
Q9GZL7	Ribosome biogenesis protein WDR12 OS=Homo sapiens OX=9606 GN=WDR12 PE=1 SV=2	0,484	1,203	0,591
P46781	Small ribosomal subunit protein uS4 OS=Homo sapiens OX=9606 GN=RPS9 PE=1 SV=3	0,452	1,236	0,589
P49916	DNA ligase 3 OS=Homo sapiens OX=9606 GN=LIG3 PE=1 SV=2	0,239	2,131	0,588
Q8TDN6	Ribosome biogenesis protein BRX1 homolog OS=Homo sapiens OX=9606 GN=BRIX1 PE=1 SV=2	0,413	0,799	0,588
Q14152	Eukaryotic translation initiation factor 3 subunit A OS=Homo sapiens OX=9606 GN=EIF3A PE=1 SV=1	0,403	1,428	0,586
P00966	Argininosuccinate synthase OS=Homo sapiens OX=9606 GN=ASS1 PE=1 SV=2	7,003	0,09	0,582
Q9Y383	Putative RNA-binding protein Luc7-like 2 OS=Homo sapiens OX=9606 GN=LUC7L2 PE=1 SV=2	0,238	2,054	0,581
Q96HY6	DDRGK domain-containing protein 1 OS=Homo sapiens OX=9606 GN=DDRGK1 PE=1 SV=2	1,171	0,468	0,581
P35270	Sepiapterin reductase OS=Homo sapiens OX=9606 GN=SPR PE=1 SV=1	1	0,13	0,579
Q92922	SWI/SNF complex subunit SMARCC1 OS=Homo sapiens OX=9606 GN=SMARCC1 PE=1 SV=3	0,204	2,256	0,576
P18859	ATP synthase-coupling factor 6, mitochondrial OS=Homo sapiens OX=9606 GN=ATP5PF PE=1 SV=1	1,433	0,41	0,575
O43837	Isocitrate dehydrogenase [NAD] subunit beta, mitochondrial OS=Homo sapiens OX=9606 GN=IDH3B PE=1 SV=2	2,026	0,315	0,573
Q6WCQ1	Myosin phosphatase Rho-interacting protein OS=Homo sapiens OX=9606 GN=MPRIP PE=1 SV=3	3,165	0,188	0,573
O60437	Periplakin OS=Homo sapiens OX=9606 GN=PPL PE=1 SV=4	0,938	0,279	0,57
P08708	Small ribosomal subunit protein eS17 OS=Homo sapiens OX=9606 GN=RPS17 PE=1 SV=2	0,454	1,767	0,565
Q9NX40	OCIA domain-containing protein 1 OS=Homo sapiens OX=9606 GN=OCIAD1 PE=1 SV=1	1,555	0,356	0,565
P23786	Carnitine O-palmitoyltransferase 2, mitochondrial OS=Homo sapiens OX=9606 GN=CPT2 PE=1 SV=2	1,106	0,423	0,562
P30511	HLA class I histocompatibility antigen, alpha chain F OS=Homo sapiens OX=9606 GN=HLA-F PE=1 SV=3	1,514	0,348	0,557
Q9Y666	Solute carrier family 12 member 7 OS=Homo sapiens OX=9606 GN=SLC12A7 PE=1 SV=3	0,344	1,527	0,555
Q99829	Copine-1 OS=Homo sapiens OX=9606 GN=CPNE1 PE=1 SV=1	0,493	1,115	0,554
Q9H845	Complex I assembly factor ACAD9, mitochondrial OS=Homo sapiens OX=9606 GN=ACAD9 PE=1 SV=1	1,381	0,423	0,553
Q5SRE5	Nucleoporin NUP188 OS=Homo sapiens OX=9606 GN=NUP188 PE=1 SV=1	1,065	0,417	0,553
Q92597	Protein NDRG1 OS=Homo sapiens OX=9606 GN=NDRG1 PE=1 SV=1	0,213	1,668	0,551
P40429	Large ribosomal subunit protein uL13 OS=Homo sapiens OX=9606 GN=RPL13A PE=1 SV=2	0,4	0,937	0,55
Q9NWU5	Large ribosomal subunit protein uL22m OS=Homo sapiens OX=9606 GN=MRPL22 PE=1 SV=1	1,297	0,362	0,542
O60716	Catenin delta-1 OS=Homo sapiens OX=9606 GN=CTNND1 PE=1 SV=1	2,703	0,19	0,542
Q7KZ85	Transcription elongation factor SPT6 OS=Homo sapiens OX=9606 GN=SUPT6H PE=1 SV=2	0,469	1,461	0,54
O43347	RNA-binding protein Musashi homolog 1 OS=Homo sapiens OX=9606 GN=MSI1 PE=1 SV=1	0,35	7,442	0,536
Q9BYN8	Small ribosomal subunit protein mS26 OS=Homo sapiens OX=9606 GN=MRPS26 PE=1 SV=1	1,653	0,377	0,536
O15400	Syntaxin-7 OS=Homo sapiens OX=9606 GN=STX7 PE=1 SV=4	1,136	0,373	0,534
P52701	DNA mismatch repair protein Msh6 OS=Homo sapiens OX=9606 GN=MSH6 PE=1 SV=2	0,265	2,247	0,532
Q9BQS8	FYVE and coiled-coil domain-containing protein 1 OS=Homo sapiens OX=9606 GN=FYCO1 PE=1 SV=3	1,33	0,382	0,529
Q8TBA6	Golgin subfamily A member 5 OS=Homo sapiens OX=9606 GN=GOLGA5 PE=1 SV=3	1,836	0,37	0,529
Q01650	Large neutral amino acids transporter small subunit 1 OS=Homo sapiens OX=9606 GN=SLC7A5 PE=1 SV=2	5,685	0,104	0,529
Q05519	Serine/arginine-rich splicing factor 11 OS=Homo sapiens OX=9606 GN=SRSF11 PE=1 SV=1	0,465	1,228	0,528
P49748	Very long-chain specific acyl-CoA dehydrogenase, mitochondrial OS=Homo sapiens OX=9606 GN=ACADVL PE=1 SV=1	2,4	0,214	0,528
Q92882	Osteoclast-stimulating factor 1 OS=Homo sapiens OX=9606 GN=OSTF1 PE=1 SV=2	2,803	0,21	0,527
P35222	Catenin beta-1 OS=Homo sapiens OX=9606 GN=CTNNB1 PE=1 SV=1	1,038	0,466	0,524
P33121	Long-chain-fatty-acid--CoA ligase 1 OS=Homo sapiens OX=9606 GN=ACSL1 PE=1 SV=1	1,083	0,364	0,524
P11166	Solute carrier family 2, facilitated glucose transporter member 1 OS=Homo sapiens OX=9606 GN=SLC2A1 PE=1 SV=2	1,615	0,324	0,524
Q14134	Tripartite motif-containing protein 29 OS=Homo sapiens OX=9606 GN=TRIM29 PE=1 SV=2	3,816	1,67	0,522
P49419	Alpha-aminoadipic semialdehyde dehydrogenase OS=Homo sapiens OX=9606 GN=ALDH7A1 PE=1 SV=5	1,249	0,184	0,521
P50502	Hsc70-interacting protein OS=Homo sapiens OX=9606 GN=ST13 PE=1 SV=2	0,199	3,021	0,519
Q9BXP5	Serrate RNA effector molecule homolog OS=Homo sapiens OX=9606 GN=SRRT PE=1 SV=1	0,455	1,163	0,518
G5E9E7	Tight junction protein 1 OS=Homo sapiens OX=9606 GN=TJP1 PE=1 SV=1	0,417	1,149	0,518
P35221	Catenin alpha-1 OS=Homo sapiens OX=9606 GN=CTNNA1 PE=1 SV=1	1,678	0,339	0,516
Q9HC38	Glyoxalase domain-containing protein 4 OS=Homo sapiens OX=9606 GN=GLOD4 PE=1 SV=1	0,303	1,667	0,515
Q13451	Peptidyl-prolyl cis-trans isomerase FKBP5 OS=Homo sapiens OX=9606 GN=FKBP5 PE=1 SV=2	0,067	5,89	0,514
Q15021	Condensin complex subunit 1 OS=Homo sapiens OX=9606 GN=NCAPD2 PE=1 SV=3	0,277	1,479	0,514
Q96DH6	RNA-binding protein Musashi homolog 2 OS=Homo sapiens OX=9606 GN=MSI2 PE=1 SV=1	0,33	1,394	0,514
Q8NE71	ATP-binding cassette sub-family F member 1 OS=Homo sapiens OX=9606 GN=ABCF1 PE=1 SV=2	0,341	1,559	0,51
P50213	Isocitrate dehydrogenase [NAD] subunit alpha, mitochondrial OS=Homo sapiens OX=9606 GN=IDH3A PE=1 SV=1	1,48	0,307	0,51
O00519	Fatty-acid amide hydrolase 1 OS=Homo sapiens OX=9606 GN=FAAH PE=1 SV=2	1,5	0,345	0,507
Q16762	Thiosulfate sulfurtransferase OS=Homo sapiens OX=9606 GN=TST PE=1 SV=4	2,897	0,186	0,506
P52292	Importin subunit alpha-1 OS=Homo sapiens OX=9606 GN=KPNA2 PE=1 SV=1	0,43	1,163	0,505
P07339	Cathepsin D OS=Homo sapiens OX=9606 GN=CTSD PE=1 SV=1	1,784	0,275	0,504
P26583	High mobility group protein B2 OS=Homo sapiens OX=9606 GN=HMGB2 PE=1 SV=2	0,217	1,848	0,501
Q07157	Tight junction protein ZO-1 OS=Homo sapiens OX=9606 GN=TJP1 PE=1 SV=3	0,447	1,11	0,5
A1L0T0	2-hydroxyacyl-CoA lyase 2 OS=Homo sapiens OX=9606 GN=ILVBL PE=1 SV=2	1,67	0,306	0,5
Q9BY44	Eukaryotic translation initiation factor 2A OS=Homo sapiens OX=9606 GN=EIF2A PE=1 SV=3	0,455	1,413	0,499
O60814	Histone H2B type 1-K OS=Homo sapiens OX=9606 GN=H2BC12 PE=1 SV=3	0,488	0,99	0,499
O14908	PDZ domain-containing protein GIPC1 OS=Homo sapiens OX=9606 GN=GIPC1 PE=1 SV=2	1,64	0,284	0,498
P15924	Desmoplakin OS=Homo sapiens OX=9606 GN=DSP PE=1 SV=3	1,216	0,382	0,497
Q6PJT7	Zinc finger CCCH domain-containing protein 14 OS=Homo sapiens OX=9606 GN=ZC3H14 PE=1 SV=1	0,809	0,586	0,496
P54725	UV excision repair protein RAD23 homolog A OS=Homo sapiens OX=9606 GN=RAD23A PE=1 SV=1	1,821	0,302	0,496
Q9UKM9	RNA-binding protein Raly OS=Homo sapiens OX=9606 GN=RALY PE=1 SV=1	0,811	0,589	0,495

P26368	Splicing factor U2AF 65 kDa subunit OS=Homo sapiens OX=9606 GN=U2AF2 PE=1 SV=4	0,601	0,851	0,493
O96005	Putative lipid scramblase CLPTM1 OS=Homo sapiens OX=9606 GN=CLPTM1 PE=1 SV=1	1,713	0,275	0,492
Q9Y3L3	SH3 domain-binding protein 1 OS=Homo sapiens OX=9606 GN=SH3BP1 PE=1 SV=3	0,527	0,953	0,49
P05141	ADP/ATP translocase 2 OS=Homo sapiens OX=9606 GN=SLC25A5 PE=1 SV=7	0,816	0,672	0,489
Q9H773	dCTP pyrophosphatase 1 OS=Homo sapiens OX=9606 GN=DCTPP1 PE=1 SV=1	0,844	0,715	0,487
Q96ST3	Paired amphipathic helix protein Sin3a OS=Homo sapiens OX=9606 GN=SIN3A PE=1 SV=2	0,53	0,964	0,486
Q13310	Polyadenylate-binding protein 4 OS=Homo sapiens OX=9606 GN=PABPC4 PE=1 SV=1	0,577	0,93	0,485
Q99614	Tetratricopeptide repeat protein 1 OS=Homo sapiens OX=9606 GN=TTC1 PE=1 SV=1	0,677	0,745	0,485
P55957	BH3-interacting domain death agonist OS=Homo sapiens OX=9606 GN=BID PE=1 SV=1	0,611	0,6	0,485
O95425	Supervillin OS=Homo sapiens OX=9606 GN=SVIL PE=1 SV=2	2,74	0,177	0,485
P12429	Annexin A3 OS=Homo sapiens OX=9606 GN=ANXA3 PE=1 SV=3	4,989	0,118	0,485
P27694	Replication protein A 70 kDa DNA-binding subunit OS=Homo sapiens OX=9606 GN=RPA1 PE=1 SV=2	0,397	1,248	0,484
P48047	ATP synthase subunit O, mitochondrial OS=Homo sapiens OX=9606 GN=ATP5PO PE=1 SV=1	0,78	0,73	0,484
Q15269	Periodic tryptophan protein 2 homolog OS=Homo sapiens OX=9606 GN=PWP2 PE=1 SV=2	0,68	0,625	0,483
P11137	Microtubule-associated protein 2 OS=Homo sapiens OX=9606 GN=MAP2 PE=1 SV=4	0,191	6,17	0,481
O60841	Eukaryotic translation initiation factor 5B OS=Homo sapiens OX=9606 GN=EIF5B PE=1 SV=4	0,337	1,179	0,48
O60264	SWI/SNF-related matrix-associated actin-dependent regulator of chromatin subfamily A member 5 OS=Homo sapiens OX=96	0,448	1,168	0,48
Q9H2U2	Inorganic pyrophosphatase 2, mitochondrial OS=Homo sapiens OX=9606 GN=PPA2 PE=1 SV=2	0,943	0,447	0,479
P82933	Small ribosomal subunit protein uS9m OS=Homo sapiens OX=9606 GN=MRPS9 PE=1 SV=2	0,909	0,73	0,478
O75691	Small subunit processome component 20 homolog OS=Homo sapiens OX=9606 GN=UTP20 PE=1 SV=3	0,647	0,747	0,476
O43520	Phospholipid-transporting ATPase IC OS=Homo sapiens OX=9606 GN=ATP8B1 PE=1 SV=3	1,866	0,617	0,474
P16219	Short-chain specific acyl-CoA dehydrogenase, mitochondrial OS=Homo sapiens OX=9606 GN=ACADS PE=1 SV=1	1000	0,001	0,473
Q9P206	NHS-like protein 3 OS=Homo sapiens OX=9606 GN=NHSL3 PE=1 SV=2	4,114	0,116	0,472
Q96KP4	Cytosolic non-specific dipeptidase OS=Homo sapiens OX=9606 GN=CNDP2 PE=1 SV=2	0,585	0,841	0,471
O75152	Zinc finger CCCH domain-containing protein 11A OS=Homo sapiens OX=9606 GN=ZC3H11A PE=1 SV=3	0,861	0,563	0,47
Q9BW92	Threonine--tRNA ligase, mitochondrial OS=Homo sapiens OX=9606 GN=TARS2 PE=1 SV=1	0,899	0,507	0,47
O75494	Serine/arginine-rich splicing factor 10 OS=Homo sapiens OX=9606 GN=SRSF10 PE=1 SV=1	1,05	0,714	0,468
O76031	ATP-dependent Clp protease ATP-binding subunit clpX-like, mitochondrial OS=Homo sapiens OX=9606 GN=CLPX PE=1 SV=2	0,841	0,601	0,467
P09874	Poly [ADP-ribose] polymerase 1 OS=Homo sapiens OX=9606 GN=PARP1 PE=1 SV=4	0,171	2,555	0,466
Q96N66	Lysophospholipid acyltransferase 7 OS=Homo sapiens OX=9606 GN=MBOAT7 PE=1 SV=2	3,904	0,13	0,465
A0A669KB	Microtubule-associated protein OS=Homo sapiens OX=9606 GN=MAP2 PE=1 SV=1	0,338	1,719	0,463
Q9H0A0	RNA cytidine acetyltransferase OS=Homo sapiens OX=9606 GN=NAT10 PE=1 SV=2	0,507	0,911	0,463
Q9UNX4	WD repeat-containing protein 3 OS=Homo sapiens OX=9606 GN=WDR3 PE=1 SV=1	0,525	0,71	0,462
O15027	Protein transport protein Sec16A OS=Homo sapiens OX=9606 GN=SEC16A PE=1 SV=4	1,143	0,504	0,462
O00425	Insulin-like growth factor 2 mRNA-binding protein 3 OS=Homo sapiens OX=9606 GN=IGF2BP3 PE=1 SV=2	0,301	1,822	0,46
Q86U38	Nucleolar protein 9 OS=Homo sapiens OX=9606 GN=NOP9 PE=1 SV=1	0,551	0,802	0,46
Q9Y3D9	Small ribosomal subunit protein mS23 OS=Homo sapiens OX=9606 GN=MRPS23 PE=1 SV=2	0,712	0,601	0,459
P30837	Aldehyde dehydrogenase X, mitochondrial OS=Homo sapiens OX=9606 GN=ALDH1B1 PE=1 SV=4	1,395	0,336	0,459
P57088	Transmembrane protein 33 OS=Homo sapiens OX=9606 GN=TMEM33 PE=1 SV=2	0,829	0,679	0,457
Q01780	Exosome complex component 10 OS=Homo sapiens OX=9606 GN=EXOSC10 PE=1 SV=2	0,636	0,724	0,456
Q63HN8	E3 ubiquitin-protein ligase RNF213 OS=Homo sapiens OX=9606 GN=RNF213 PE=1 SV=3	1,891	0,203	0,456
O76021	Ribosomal L1 domain-containing protein 1 OS=Homo sapiens OX=9606 GN=RSL1D1 PE=1 SV=3	0,791	0,645	0,455
P25205	DNA replication licensing factor MCM3 OS=Homo sapiens OX=9606 GN=MCM3 PE=1 SV=3	0,434	1,091	0,453
P02751	Fibronectin OS=Homo sapiens OX=9606 GN=FN1 PE=1 SV=5	2,768	0,162	0,453
P42765	3-ketoacyl-CoA thiolase, mitochondrial OS=Homo sapiens OX=9606 GN=ACAA2 PE=1 SV=2	0,992	0,446	0,451
P22087	rRNA 2'-O-methyltransferase fibrillarin OS=Homo sapiens OX=9606 GN=FBL PE=1 SV=2	0,888	0,701	0,45
Q9BYG3	MKI67 FHA domain-interacting nucleolar phosphoprotein OS=Homo sapiens OX=9606 GN=NIFK PE=1 SV=1	0,51	0,652	0,45
Q16363	Laminin subunit alpha-4 OS=Homo sapiens OX=9606 GN=LAMA4 PE=1 SV=4	7,991	0,783	0,448
P62851	Small ribosomal subunit protein eS25 OS=Homo sapiens OX=9606 GN=RPS25 PE=1 SV=1	0,382	1,272	0,447
Q8IZL8	Proline-, glutamic acid- and leucine-rich protein 1 OS=Homo sapiens OX=9606 GN=PELP1 PE=1 SV=2	0,702	0,655	0,445
Q9NZL9	Methionine adenosyltransferase 2 subunit beta OS=Homo sapiens OX=9606 GN=MAT2B PE=1 SV=1	0,27	1,803	0,444
O60885	Bromodomain-containing protein 4 OS=Homo sapiens OX=9606 GN=BRD4 PE=1 SV=2	0,691	0,562	0,444
Q9ULZ3	Apoptosis-associated speck-like protein containing a CARD OS=Homo sapiens OX=9606 GN=PYCARD PE=1 SV=2	3,982	0,072	0,442
Q9H6E4	Coiled-coil domain-containing protein 134 OS=Homo sapiens OX=9606 GN=CCDC134 PE=1 SV=1	0,435	0,769	0,441
Q15050	Ribosome biogenesis regulatory protein homolog OS=Homo sapiens OX=9606 GN=RRS1 PE=1 SV=2	0,723	0,578	0,44
O43143	ATP-dependent RNA helicase DHX15 OS=Homo sapiens OX=9606 GN=DHX15 PE=1 SV=2	0,38	1,082	0,437
Q9NVI1	Fanconi anemia group I protein OS=Homo sapiens OX=9606 GN=FANCI PE=1 SV=4	0,359	1,103	0,436
Q9BVP2	Guanine nucleotide-binding protein-like 3 OS=Homo sapiens OX=9606 GN=GNL3 PE=1 SV=2	0,633	0,641	0,436
Q99848	Probable rRNA-processing protein EBP2 OS=Homo sapiens OX=9606 GN=EBNA1BP2 PE=1 SV=2	0,614	0,686	0,435
Q9UJS0	Electrogenic aspartate/glutamate antiporter SLC25A13, mitochondrial OS=Homo sapiens OX=9606 GN=SLC25A13 PE=1 SV=2	0,42	1,052	0,434
Q9H7B2	Ribosome production factor 2 homolog OS=Homo sapiens OX=9606 GN=RPF2 PE=1 SV=2	0,594	0,705	0,434
Q12931	Heat shock protein 75 kDa, mitochondrial OS=Homo sapiens OX=9606 GN=TRAP1 PE=1 SV=3	1,074	0,386	0,434
Q08945	FACT complex subunit SSRP1 OS=Homo sapiens OX=9606 GN=SSRP1 PE=1 SV=1	0,336	1,349	0,433
Q07021	Complement component 1 Q subcomponent-binding protein, mitochondrial OS=Homo sapiens OX=9606 GN=C1QBP PE=1 SV	0,619	0,74	0,433
P28838	Cytosol aminopeptidase OS=Homo sapiens OX=9606 GN=LAP3 PE=1 SV=3	2,424	0,175	0,433
O95232	Luc7-like protein 3 OS=Homo sapiens OX=9606 GN=LUC7L3 PE=1 SV=2	0,463	0,985	0,432
Q8NI36	WD repeat-containing protein 36 OS=Homo sapiens OX=9606 GN=WDR36 PE=1 SV=1	0,632	0,762	0,431
Q9BZJ0	Crooked neck-like protein 1 OS=Homo sapiens OX=9606 GN=CRNKL1 PE=1 SV=4	0,56	0,758	0,43
Q96GQ7	Probable ATP-dependent RNA helicase DDX27 OS=Homo sapiens OX=9606 GN=DDX27 PE=1 SV=2	0,87	0,627	0,43
Q96CM8	Medium-chain acyl-CoA ligase ACSF2, mitochondrial OS=Homo sapiens OX=9606 GN=ACSF2 PE=1 SV=2	1,784	0,213	0,43
O43148	mRNA cap guanine-N7 methyltransferase OS=Homo sapiens OX=9606 GN=RNMT PE=1 SV=1	0,625	0,934	0,429
Q01105	Protein SET OS=Homo sapiens OX=9606 GN=SET PE=1 SV=3	0,53	0,784	0,428
Q9NZ01	Very-long-chain enoyl-CoA reductase OS=Homo sapiens OX=9606 GN=TECR PE=1 SV=1	1,259	0,383	0,428
Q9NVI7	ATPase family AAA domain-containing protein 3A OS=Homo sapiens OX=9606 GN=ATAD3A PE=1 SV=2	0,707	0,606	0,427
Q9BV38	WD repeat-containing protein 18 OS=Homo sapiens OX=9606 GN=WDR18 PE=1 SV=2	0,899	0,462	0,427
Q5JTH9	RRP12-like protein OS=Homo sapiens OX=9606 GN=RRP12 PE=1 SV=2	0,645	0,668	0,426
Q9H6W3	Ribosomal oxygenase 1 OS=Homo sapiens OX=9606 GN=RIOX1 PE=1 SV=2	0,799	0,561	0,426
P18754	Regulator of chromosome condensation OS=Homo sapiens OX=9606 GN=RCC1 PE=1 SV=1	0,743	0,57	0,422
Q9H6R4	Nucleolar protein 6 OS=Homo sapiens OX=9606 GN=NOL6 PE=1 SV=2	0,829	0,508	0,421
Q92841	Probable ATP-dependent RNA helicase DDX17 OS=Homo sapiens OX=9606 GN=DDX17 PE=1 SV=2	0,675	0,633	0,419
Q8TAE8	Large ribosomal subunit protein mL64 OS=Homo sapiens OX=9606 GN=GADD45GIP1 PE=1 SV=1	1,106	0,377	0,419
Q9UKD2	mRNA turnover protein 4 homolog OS=Homo sapiens OX=9606 GN=MRTO4 PE=1 SV=2	0,508	0,877	0,418

Q14160	Protein scribble homolog OS=Homo sapiens OX=9606 GN=SCRIB PE=1 SV=5	0,842	0,536	0,418
Q96SI9	Spermatid perinuclear RNA-binding protein OS=Homo sapiens OX=9606 GN=STRBP PE=1 SV=1	0,386	1,042	0,417
Q5JRX3	Presequence protease, mitochondrial OS=Homo sapiens OX=9606 GN=PITRM1 PE=1 SV=3	1,948	0,216	0,417
P16989	Y-box-binding protein 3 OS=Homo sapiens OX=9606 GN=YBX3 PE=1 SV=4	0,794	0,479	0,416
Q9P2R7	Succinate--CoA ligase [ADP-forming] subunit beta, mitochondrial OS=Homo sapiens OX=9606 GN=SUCLA2 PE=1 SV=3	0,908	0,492	0,412
Q96RQ3	Methylcrotonoyl-CoA carboxylase subunit alpha, mitochondrial OS=Homo sapiens OX=9606 GN=MCCC1 PE=1 SV=3	1,513	0,294	0,412
Q9Y512	Sorting and assembly machinery component 50 homolog OS=Homo sapiens OX=9606 GN=SAMM50 PE=1 SV=3	0,846	0,533	0,411
Q14807	Kinesin-like protein KIF22 OS=Homo sapiens OX=9606 GN=KIF22 PE=1 SV=5	0,837	0,515	0,411
P00505	Aspartate aminotransferase, mitochondrial OS=Homo sapiens OX=9606 GN=GOT2 PE=1 SV=3	0,92	0,498	0,41
Q14966	Zinc finger protein 638 OS=Homo sapiens OX=9606 GN=ZNF638 PE=1 SV=2	0,373	0,85	0,409
Q14137	Ribosome biogenesis protein BOP1 OS=Homo sapiens OX=9606 GN=BOP1 PE=1 SV=2	0,355	1,145	0,408
O75400	Pre-mRNA-processing factor 40 homolog A OS=Homo sapiens OX=9606 GN=PRPF40A PE=1 SV=2	0,771	0,711	0,407
Q9UQ35	Serine/arginine repetitive matrix protein 2 OS=Homo sapiens OX=9606 GN=SRRM2 PE=1 SV=2	0,793	0,551	0,406
P35637	RNA-binding protein FUS OS=Homo sapiens OX=9606 GN=FUS PE=1 SV=1	0,668	0,986	0,404
Q9HAV7	GrpE protein homolog 1, mitochondrial OS=Homo sapiens OX=9606 GN=GRPEL1 PE=1 SV=2	1,311	0,321	0,403
O43795	Unconventional myosin-Ib OS=Homo sapiens OX=9606 GN=MYO1B PE=1 SV=3	1,004	0,365	0,402
O00541	Pescadillo homolog OS=Homo sapiens OX=9606 GN=PES1 PE=1 SV=1	0,375	1,245	0,393
Q99959	Plakophilin-2 OS=Homo sapiens OX=9606 GN=PKP2 PE=1 SV=2	2,194	0,184	0,392
Q13618	Cullin-3 OS=Homo sapiens OX=9606 GN=CUL3 PE=1 SV=2	0,264	1,828	0,391
Q9Y2S7	Polymerase delta-interacting protein 2 OS=Homo sapiens OX=9606 GN=POLDIP2 PE=1 SV=1	0,796	0,474	0,391
O43823	A-kinase anchor protein 8 OS=Homo sapiens OX=9606 GN=AKAP8 PE=1 SV=1	0,985	0,362	0,391
P08621	U1 small nuclear ribonucleoprotein 70 kDa OS=Homo sapiens OX=9606 GN=SNRNP70 PE=1 SV=2	0,682	0,586	0,39
P82675	Small ribosomal subunit protein uS5m OS=Homo sapiens OX=9606 GN=MRPS5 PE=1 SV=2	0,753	1,056	0,389
Q9UKV3	Apoptotic chromatin condensation inducer in the nucleus OS=Homo sapiens OX=9606 GN=ACIN1 PE=1 SV=2	0,566	0,699	0,387
Q14677	Clathrin interactor 1 OS=Homo sapiens OX=9606 GN=CLINT1 PE=1 SV=1	0,919	0,523	0,386
Q99661	Kinesin-like protein KIF2C OS=Homo sapiens OX=9606 GN=KIF2C PE=1 SV=2	0,228	1,569	0,385
Q5UIP0	Telomere-associated protein RIF1 OS=Homo sapiens OX=9606 GN=RIF1 PE=1 SV=2	0,649	0,636	0,384
Q9NYF8	Bcl-2-associated transcription factor 1 OS=Homo sapiens OX=9606 GN=BCLAF1 PE=1 SV=2	0,604	0,619	0,383
Q16891	MICOS complex subunit MIC60 OS=Homo sapiens OX=9606 GN=IMMT PE=1 SV=1	0,749	0,502	0,383
Q14534	Squalene monooxygenase OS=Homo sapiens OX=9606 GN=SQLE PE=1 SV=3	0,393	1,063	0,382
Q6P1M0	Long-chain fatty acid transport protein 4 OS=Homo sapiens OX=9606 GN=SLC27A4 PE=1 SV=1	1,37	0,322	0,381
O75937	DnaJ homolog subfamily C member 8 OS=Homo sapiens OX=9606 GN=DNAJC8 PE=1 SV=2	0,295	1,491	0,378
Q13123	Protein Red OS=Homo sapiens OX=9606 GN=IK PE=1 SV=3	0,569	0,803	0,378
Q13084	Large ribosomal subunit protein bL28m OS=Homo sapiens OX=9606 GN=MRPL28 PE=1 SV=4	1,374	0,453	0,377
O95202	Mitochondrial proton/calcium exchanger protein OS=Homo sapiens OX=9606 GN=LETM1 PE=1 SV=1	1,288	0,309	0,376
Q9BVJ6	U3 small nucleolar RNA-associated protein 14 homolog A OS=Homo sapiens OX=9606 GN=UTP14A PE=1 SV=1	0,5	0,693	0,373
P23368	NAD-dependent malic enzyme, mitochondrial OS=Homo sapiens OX=9606 GN=ME2 PE=1 SV=1	0,802	0,458	0,372
Q13523	Serine/threonine-protein kinase PRP4 homolog OS=Homo sapiens OX=9606 GN=PRPF4B PE=1 SV=3	0,982	0,419	0,372
Q9H583	HEAT repeat-containing protein 1 OS=Homo sapiens OX=9606 GN=HEATR1 PE=1 SV=3	0,55	0,682	0,371
Q9NP81	Serine--tRNA ligase, mitochondrial OS=Homo sapiens OX=9606 GN=SARS2 PE=1 SV=1	0,683	0,664	0,371
P53007	Tricarboxylate transport protein, mitochondrial OS=Homo sapiens OX=9606 GN=SLC25A1 PE=1 SV=2	0,706	0,747	0,37
Q9NR46	Endophilin-B2 OS=Homo sapiens OX=9606 GN=SH3GLB2 PE=1 SV=1	1,732	0,242	0,37
Q9GZT3	SRA stem-loop-interacting RNA-binding protein, mitochondrial OS=Homo sapiens OX=9606 GN=SLIRP PE=1 SV=1	0,385	0,857	0,369
Q92900	Regulator of nonsense transcripts 1 OS=Homo sapiens OX=9606 GN=UPF1 PE=1 SV=2	0,688	0,618	0,368
Q8TCT9	Minor histocompatibility antigen H13 OS=Homo sapiens OX=9606 GN=HM13 PE=1 SV=1	1,315	0,285	0,368
P61604	10 kDa heat shock protein, mitochondrial OS=Homo sapiens OX=9606 GN=HSPE1 PE=1 SV=2	0,634	0,574	0,367
Q13740	CD166 antigen OS=Homo sapiens OX=9606 GN=ALCAM PE=1 SV=2	4,373	0,095	0,367
Q9Y6M1	Insulin-like growth factor 2 mRNA-binding protein 2 OS=Homo sapiens OX=9606 GN=IGF2BP2 PE=1 SV=2	0,266	1,682	0,364
Q13823	Nucleolar GTP-binding protein 2 OS=Homo sapiens OX=9606 GN=GNL2 PE=1 SV=1	0,524	0,627	0,363
P13647	Keratin, type II cytoskeletal 5 OS=Homo sapiens OX=9606 GN=KRT5 PE=1 SV=3	2,181	0,192	0,363
Q6P1M3	LLGL scribble cell polarity complex component 2 OS=Homo sapiens OX=9606 GN=LLGL2 PE=1 SV=2			0,36
Q9NX63	MICOS complex subunit MIC19 OS=Homo sapiens OX=9606 GN=CHCHD3 PE=1 SV=1	0,897	0,386	0,36
P48449	Lanosterol synthase OS=Homo sapiens OX=9606 GN=LSS PE=1 SV=1	0,237	1,633	0,358
Q6PI48	Aspartate--tRNA ligase, mitochondrial OS=Homo sapiens OX=9606 GN=DARS2 PE=1 SV=1	1,518	0,229	0,356
Q13505	Metaxin-1 OS=Homo sapiens OX=9606 GN=MTX1 PE=1 SV=3	0,88	0,422	0,354
Q14684	Ribosomal RNA processing protein 1 homolog B OS=Homo sapiens OX=9606 GN=RRP1B PE=1 SV=3	0,197	1,387	0,352
P09497	Clathrin light chain B OS=Homo sapiens OX=9606 GN=CLTB PE=1 SV=1	2,245	0,151	0,35
O00567	Nucleolar protein 56 OS=Homo sapiens OX=9606 GN=NOP56 PE=1 SV=4	0,632	0,568	0,349
Q13243	Serine/arginine-rich splicing factor 5 OS=Homo sapiens OX=9606 GN=SRSF5 PE=1 SV=1	0,494	0,739	0,348
Q9ULW0	Targeting protein for Xklp2 OS=Homo sapiens OX=9606 GN=TPX2 PE=1 SV=2	0,592	0,609	0,347
Q9BZE4	GTP-binding protein 4 OS=Homo sapiens OX=9606 GN=GTPBP4 PE=1 SV=3	0,557	0,61	0,345
O00515	Ladinin-1 OS=Homo sapiens OX=9606 GN=LAD1 PE=1 SV=2	1,679	0,201	0,345
Q9Y2X3	Nucleolar protein 58 OS=Homo sapiens OX=9606 GN=NOP58 PE=1 SV=1	0,594	0,615	0,343
Q07955	Serine/arginine-rich splicing factor 1 OS=Homo sapiens OX=9606 GN=SRSF1 PE=1 SV=2	0,666	0,572	0,342
P18858	DNA ligase 1 OS=Homo sapiens OX=9606 GN=LIG1 PE=1 SV=1	0,351	0,533	0,342
O15498	Synaptobrevin homolog YKT6 OS=Homo sapiens OX=9606 GN=YKT6 PE=1 SV=1	0,605	1,395	0,34
P10809	60 kDa heat shock protein, mitochondrial OS=Homo sapiens OX=9606 GN=HSPD1 PE=1 SV=2	0,456	0,948	0,339
P68431	Histone H3.1 OS=Homo sapiens OX=9606 GN=H3C1 PE=1 SV=2	0,464	0,928	0,333
Q96C19	EF-hand domain-containing protein D2 OS=Homo sapiens OX=9606 GN=EFHD2 PE=1 SV=1	1,216	0,265	0,332
Q9H0H5	Rac GTPase-activating protein 1 OS=Homo sapiens OX=9606 GN=RACGAP1 PE=1 SV=1	1,018	0,345	0,331
P09622	Dihydrolipoyl dehydrogenase, mitochondrial OS=Homo sapiens OX=9606 GN=DLD PE=1 SV=2	0,826	0,595	0,329
Q9Y2W1	Thyroid hormone receptor-associated protein 3 OS=Homo sapiens OX=9606 GN=THRAP3 PE=1 SV=2	0,556	0,636	0,327
P37268	Squalene synthase OS=Homo sapiens OX=9606 GN=FDFT1 PE=1 SV=1	0,106	3,622	0,326
Q13268	Dehydrogenase/reductase SDR family member 2, mitochondrial OS=Homo sapiens OX=9606 GN=DHRS2 PE=1 SV=4	1,837	0,569	0,324
Q9H307	Pinin OS=Homo sapiens OX=9606 GN=PNN PE=1 SV=5	0,564	0,558	0,321
Q14690	Protein RRP5 homolog OS=Homo sapiens OX=9606 GN=PDCD11 PE=1 SV=3	0,362	0,788	0,32
P39748	Flap endonuclease 1 OS=Homo sapiens OX=9606 GN=FEN1 PE=1 SV=1	0,394	0,853	0,319
Q92747	Actin-related protein 2/3 complex subunit 1A OS=Homo sapiens OX=9606 GN=ARPC1A PE=1 SV=2	0,462	0,705	0,319
Q03701	CCAAT/enhancer-binding protein zeta OS=Homo sapiens OX=9606 GN=CEBPZ PE=1 SV=3	0,357	0,989	0,318
P17275	Transcription factor JunB OS=Homo sapiens OX=9606 GN=JUNB PE=1 SV=1	2,101	0,115	0,318
Q9BXW7	Haloacid dehalogenase-like hydrolase domain-containing 5 OS=Homo sapiens OX=9606 GN=HDHD5 PE=1 SV=1	0,425	1,029	0,317
Q9BQG0	Myb-binding protein 1A OS=Homo sapiens OX=9606 GN=MYBBP1A PE=1 SV=2	0,476	0,646	0,317

Q00796	Sorbitol dehydrogenase OS=Homo sapiens OX=9606 GN=SORD PE=1 SV=4	0,259	1,458	0,312
P14923	Junction plakoglobin OS=Homo sapiens OX=9606 GN=JUP PE=1 SV=3	1,971	0,159	0,308
A0A2R8YD	Tight junction protein ZO-2 OS=Homo sapiens OX=9606 PE=1 SV=1	0,815	0,275	0,307
P61313	Large ribosomal subunit protein eL15 OS=Homo sapiens OX=9606 GN=RPL15 PE=1 SV=2	0,575	0,862	0,306
Q9NWH9	SAFB-like transcription modulator OS=Homo sapiens OX=9606 GN=SLTM PE=1 SV=2	0,431	0,575	0,305
Q9UKS6	Protein kinase C and casein kinase substrate in neurons protein 3 OS=Homo sapiens OX=9606 GN=PACSIN3 PE=1 SV=2	1,426	0,221	0,305
O95833	Chloride intracellular channel protein 3 OS=Homo sapiens OX=9606 GN=CLIC3 PE=1 SV=2	1000	0,001	0,305
O43278	Kunitz-type protease inhibitor 1 OS=Homo sapiens OX=9606 GN=SPINT1 PE=1 SV=2	2,596	0,025	0,303
P06748	Nucleophosmin OS=Homo sapiens OX=9606 GN=NPM1 PE=1 SV=2	0,371	0,854	0,302
Q86XP3	ATP-dependent RNA helicase DDX42 OS=Homo sapiens OX=9606 GN=DDX42 PE=1 SV=1	0,508	0,618	0,297
Q5T9A4	ATPase family AAA domain-containing protein 3B OS=Homo sapiens OX=9606 GN=ATAD3B PE=1 SV=1	0,678	0,475	0,295
Q15154	Pericentriolar material 1 protein OS=Homo sapiens OX=9606 GN=PCM1 PE=1 SV=6	0,957	0,995	0,289
Q7Z4W1	L-xylulose reductase OS=Homo sapiens OX=9606 GN=DCXR PE=1 SV=2	0,933	0,177	0,289
P19338	Nucleolin OS=Homo sapiens OX=9606 GN=NCL PE=1 SV=3	0,315	0,966	0,287
P18583	Protein SON OS=Homo sapiens OX=9606 GN=SON PE=1 SV=4	0,5	0,612	0,287
Q86VM9	Zinc finger CCCH domain-containing protein 18 OS=Homo sapiens OX=9606 GN=ZC3H18 PE=1 SV=2	0,799	0,626	0,283
Q9BQP7	Mitochondrial genome maintenance exonuclease 1 OS=Homo sapiens OX=9606 GN=MGME1 PE=1 SV=1	0,632	0,422	0,28
Q8WXX5	DnaJ homolog subfamily C member 9 OS=Homo sapiens OX=9606 GN=DNAJC9 PE=1 SV=1	0,728	0,399	0,278
P46782	Small ribosomal subunit protein uS7 OS=Homo sapiens OX=9606 GN=RPS5 PE=1 SV=4	0,42	0,62	0,277
Q9Y399	Small ribosomal subunit protein uS2m OS=Homo sapiens OX=9606 GN=MRPS2 PE=1 SV=1	1,191	0,578	0,277
Q9NVP1	ATP-dependent RNA helicase DDX18 OS=Homo sapiens OX=9606 GN=DDX18 PE=1 SV=2	0,296	0,89	0,275
P52566	Rho GDP-dissociation inhibitor 2 OS=Homo sapiens OX=9606 GN=ARHGDIB PE=1 SV=3	0,655	0,152	0,275
P16401	Histone H1.5 OS=Homo sapiens OX=9606 GN=H1-5 PE=1 SV=3	0,303	0,872	0,273
Q8TEQ6	Gem-associated protein 5 OS=Homo sapiens OX=9606 GN=GEMIN5 PE=1 SV=3	0,333	0,752	0,273
Q7Z434	Mitochondrial antiviral-signaling protein OS=Homo sapiens OX=9606 GN=MAVS PE=1 SV=2	1,29	0,229	0,269
O00148	ATP-dependent RNA helicase DDX39A OS=Homo sapiens OX=9606 GN=DDX39A PE=1 SV=2	0,676	0,42	0,266
Q8NFV4	sn-1-specific diacylglycerol lipase ABHD11 OS=Homo sapiens OX=9606 GN=ABHD11 PE=1 SV=2	1,457	0,148	0,266
Q02952	A-kinase anchor protein 12 OS=Homo sapiens OX=9606 GN=AKAP12 PE=1 SV=4	0,143	1,942	0,265
P11388	DNA topoisomerase 2-alpha OS=Homo sapiens OX=9606 GN=TOP2A PE=1 SV=3	0,317	0,756	0,263
P36952	Serpin B5 OS=Homo sapiens OX=9606 GN=SERPINB5 PE=1 SV=2	2,286	0,083	0,262
P55327	Tumor protein D52 OS=Homo sapiens OX=9606 GN=TPD52 PE=1 SV=2	0,776	0,316	0,261
O43818	U3 small nucleolar RNA-interacting protein 2 OS=Homo sapiens OX=9606 GN=RRP9 PE=1 SV=1	0,547	0,438	0,257
O75521	Enoyl-CoA delta isomerase 2 OS=Homo sapiens OX=9606 GN=ECI2 PE=1 SV=4	0,747	0,316	0,257
O94832	Unconventional myosin-Id OS=Homo sapiens OX=9606 GN=MYO1D PE=1 SV=2	0,765	0,283	0,256
E9PDI4	Ladinin-1 OS=Homo sapiens OX=9606 GN=LAD1 PE=1 SV=1	1,252	0,188	0,256
Q8NBN7	Retinol dehydrogenase 13 OS=Homo sapiens OX=9606 GN=RDH13 PE=1 SV=2	0,721	0,279	0,253
Q15223	Nectin-1 OS=Homo sapiens OX=9606 GN=NECTIN1 PE=1 SV=3	0,24	0,695	0,252
P49790	Nuclear pore complex protein Nup153 OS=Homo sapiens OX=9606 GN=NUP153 PE=1 SV=2	1,167	0,213	0,251
Q16625	Occludin OS=Homo sapiens OX=9606 GN=OCLN PE=1 SV=1	1,115	0,224	0,25
P78310	Coxsackievirus and adenovirus receptor OS=Homo sapiens OX=9606 GN=CXADR PE=1 SV=1	0,376	4,341	0,247
Q16787	Laminin subunit alpha-3 OS=Homo sapiens OX=9606 GN=LAMA3 PE=1 SV=3	1,939	0,211	0,247
P16144	Integrin beta-4 OS=Homo sapiens OX=9606 GN=ITGB4 PE=1 SV=5	1,482	0,214	0,242
O14787	Transportin-2 OS=Homo sapiens OX=9606 GN=TNPO2 PE=1 SV=3	1,095	0,286	0,236
Q8IYB3	Serine/arginine repetitive matrix protein 1 OS=Homo sapiens OX=9606 GN=SRRM1 PE=1 SV=2	0,437	0,743	0,23
P25685	DnaJ homolog subfamily B member 1 OS=Homo sapiens OX=9606 GN=DNAJB1 PE=1 SV=4	1,037	0,306	0,229
O15254	Peroxisomal acyl-coenzyme A oxidase 3 OS=Homo sapiens OX=9606 GN=ACOX3 PE=1 SV=2	0,999	0,263	0,225
Q16647	Prostacyclin synthase OS=Homo sapiens OX=9606 GN=PTGIS PE=1 SV=1	0,545	0,037	0,224
Q05639	Elongation factor 1-alpha 2 OS=Homo sapiens OX=9606 GN=EEF1A2 PE=1 SV=1	0,654	0,578	0,222
P51649	Succinate-semialdehyde dehydrogenase, mitochondrial OS=Homo sapiens OX=9606 GN=ALDH5A1 PE=1 SV=2	0,485	0,614	0,213
Q96HY7	2-oxoadipate dehydrogenase complex component E1 OS=Homo sapiens OX=9606 GN=DHTKD1 PE=1 SV=2	0,799	0,268	0,21
P15311	Ezrin OS=Homo sapiens OX=9606 GN=EZR PE=1 SV=4	0,348	0,548	0,204
P09668	Pro-cathepsin H OS=Homo sapiens OX=9606 GN=CTSH PE=1 SV=4	1,486	0,092	0,204
Q9UHQ9	NADH-cytochrome b5 reductase 1 OS=Homo sapiens OX=9606 GN=CYB5R1 PE=1 SV=1	2,471	0,07	0,204
P46013	Proliferation marker protein Ki-67 OS=Homo sapiens OX=9606 GN=MKI67 PE=1 SV=2	0,644	0,311	0,201
P84243	Histone H3.3 OS=Homo sapiens OX=9606 GN=H3-3A PE=1 SV=2	0,283	1,075	0,2
Q9UQB8	Brain-specific angiogenesis inhibitor 1-associated protein 2 OS=Homo sapiens OX=9606 GN=BAIAP2 PE=1 SV=1	1,565	0,15	0,2
Q9UDY2	Tight junction protein ZO-2 OS=Homo sapiens OX=9606 GN=TJP2 PE=1 SV=2	0,955	0,171	0,193
Q9UGT4	Sushi domain-containing protein 2 OS=Homo sapiens OX=9606 GN=SUSD2 PE=1 SV=1		0,081	0,19
Q04912	Macrophage-stimulating protein receptor OS=Homo sapiens OX=9606 GN=MST1R PE=1 SV=3	1,617	0,116	0,187
Q9UBM7	7-dehydrocholesterol reductase OS=Homo sapiens OX=9606 GN=DHCR7 PE=1 SV=1	0,181	0,867	0,173
Q9Y624	Junctional adhesion molecule A OS=Homo sapiens OX=9606 GN=F11R PE=1 SV=1	3,526	0,082	0,17
O43491	Band 4.1-like protein 2 OS=Homo sapiens OX=9606 GN=EPB41L2 PE=1 SV=1	0,206	0,658	0,169
Q02241	Kinesin-like protein KIF23 OS=Homo sapiens OX=9606 GN=KIF23 PE=1 SV=3	0,569	0,266	0,168
Q9H6F5	Coiled-coil domain-containing protein 86 OS=Homo sapiens OX=9606 GN=CCDC86 PE=1 SV=1	0,778	0,198	0,168
Q9H6S3	Epidermal growth factor receptor kinase substrate 8-like protein 2 OS=Homo sapiens OX=9606 GN=EPS8L2 PE=1 SV=2	1000	0,001	0,16
P26038	Moesin OS=Homo sapiens OX=9606 GN=MSN PE=1 SV=3	0,095	1,606	0,152
Q6ZRV2	Protein FAM83H OS=Homo sapiens OX=9606 GN=FAM83H PE=1 SV=3	1,008	0,156	0,147
Q7Z406	Myosin-14 OS=Homo sapiens OX=9606 GN=MYH14 PE=1 SV=2	0,853	0,196	0,141
P22570	NADPH:adrenodoxin oxidoreductase, mitochondrial OS=Homo sapiens OX=9606 GN=FDXR PE=1 SV=3	1,003	0,168	0,139
Q13428	Treacle protein OS=Homo sapiens OX=9606 GN=TCOF1 PE=1 SV=3	0,317	0,448	0,135
Q9BZG1	Ras-related protein Rab-34 OS=Homo sapiens OX=9606 GN=RAB34 PE=1 SV=1	0,743	0,339	0,134
Q9UM54	Unconventional myosin-VI OS=Homo sapiens OX=9606 GN=MYO6 PE=1 SV=4	0,378	0,315	0,129
P35659	Protein DEK OS=Homo sapiens OX=9606 GN=DEK PE=1 SV=1	0,465	0,272	0,124
P22223	Cadherin-3 OS=Homo sapiens OX=9606 GN=CDH3 PE=1 SV=2	4,807	0,051	0,123
P04233	HLA class II histocompatibility antigen gamma chain OS=Homo sapiens OX=9606 GN=CD74 PE=1 SV=3	2,462	0,039	0,12
A0A1Y0BR	Unconventional myosin-VI OS=Homo sapiens OX=9606 GN=MYO6 PE=1 SV=1	0,322	0,374	0,111
P05783	Keratin, type I cytoskeletal 18 OS=Homo sapiens OX=9606 GN=KRT18 PE=1 SV=2	0,972	0,112	0,109
Q86SQ0	Pleckstrin homology-like domain family B member 2 OS=Homo sapiens OX=9606 GN=PHLDB2 PE=1 SV=2	0,543	0,181	0,108
P50895	Basal cell adhesion molecule OS=Homo sapiens OX=9606 GN=BCAM PE=1 SV=2	4,096	0,026	0,1
Q9Y446	Plakophilin-3 OS=Homo sapiens OX=9606 GN=PKP3 PE=1 SV=1		0,089	0,088
P04637	Cellular tumor antigen p53 OS=Homo sapiens OX=9606 GN=TP53 PE=1 SV=4	1,163	0,072	0,083
P27144	Adenylate kinase 4, mitochondrial OS=Homo sapiens OX=9606 GN=AK4 PE=1 SV=1	0,373	0,131	0,079

P05549	Transcription factor AP-2-alpha OS=Homo sapiens OX=9606 GN=TFAP2A PE=1 SV=1	0,605	0,124	0,079
Q86X29	Lipolysis-stimulated lipoprotein receptor OS=Homo sapiens OX=9606 GN=LSR PE=1 SV=4	0,606	0,141	0,075
P31947	14-3-3 protein sigma OS=Homo sapiens OX=9606 GN=SFN PE=1 SV=1	0,825	0,115	0,072
P32004	Neural cell adhesion molecule L1 OS=Homo sapiens OX=9606 GN=L1CAM PE=1 SV=2	0,751	0,074	0,059
P05787	Keratin, type II cytoskeletal 8 OS=Homo sapiens OX=9606 GN=KRT8 PE=1 SV=7	0,648	0,075	0,052
Q8TEM1	Nuclear pore membrane glycoprotein 210 OS=Homo sapiens OX=9606 GN=NUP210 PE=1 SV=3	0,068	0,863	0,05
Q04695	Keratin, type I cytoskeletal 17 OS=Homo sapiens OX=9606 GN=KRT17 PE=1 SV=2	3,687	0,021	0,049
Q9BV40	Vesicle-associated membrane protein 8 OS=Homo sapiens OX=9606 GN=VAMP8 PE=1 SV=1	3,904	0,014	0,048
P04181	Ornithine aminotransferase, mitochondrial OS=Homo sapiens OX=9606 GN=OAT PE=1 SV=1	0,524	0,099	0,045
Q14126	Desmoglein-2 OS=Homo sapiens OX=9606 GN=DSG2 PE=1 SV=2	0,897	0,304	0,042
Q9BRX8	Peroxiredoxin-like 2A OS=Homo sapiens OX=9606 GN=PRXL2A PE=1 SV=3	0,007	4,622	0,041
P47895	Retinaldehyde dehydrogenase 3 OS=Homo sapiens OX=9606 GN=ALDH1A3 PE=1 SV=2	1,871	0,017	0,037
P13646	Keratin, type I cytoskeletal 13 OS=Homo sapiens OX=9606 GN=KRT13 PE=1 SV=4	1000	0,001	0,034
P12830	Cadherin-1 OS=Homo sapiens OX=9606 GN=CDH1 PE=1 SV=3	1,109	0,03	0,032
P09758	Tumor-associated calcium signal transducer 2 OS=Homo sapiens OX=9606 GN=TACSTD2 PE=1 SV=3	3,419	0,019	0,024
P08727	Keratin, type I cytoskeletal 19 OS=Homo sapiens OX=9606 GN=KRT19 PE=1 SV=4	2,536	0,019	0,022
P08729	Keratin, type II cytoskeletal 7 OS=Homo sapiens OX=9606 GN=KRT7 PE=1 SV=5	1,452	0,004	0,006
O95864	Acyl-CoA 6-desaturase OS=Homo sapiens OX=9606 GN=FADS2 PE=1 SV=1	0,001	9,728	0,001
Q9NU22	Midasin OS=Homo sapiens OX=9606 GN=MDN1 PE=1 SV=2	0,001	1,896	0,001
Q16352	Alpha-internexin OS=Homo sapiens OX=9606 GN=INA PE=1 SV=2	0,001	1,873	0,001
Q99988	Growth/differentiation factor 15 OS=Homo sapiens OX=9606 GN=GDF15 PE=1 SV=3	0,001	0,897	0,001
P50440	Glycine amidinotransferase, mitochondrial OS=Homo sapiens OX=9606 GN=GATM PE=1 SV=1	0,001	0,646	0,001
Q8IVT2	Mitotic interactor and substrate of PLK1 OS=Homo sapiens OX=9606 GN=MISP PE=1 SV=1	0,001	0,296	0,001
P57735	Ras-related protein Rab-25 OS=Homo sapiens OX=9606 GN=RAB25 PE=1 SV=2	0,001	0,103	0,001
O15231	Zinc finger protein 185 OS=Homo sapiens OX=9606 GN=ZNF185 PE=1 SV=3		0,001	0,001
Q8WWA1	Transmembrane protein 40 OS=Homo sapiens OX=9606 GN=TMEM40 PE=1 SV=2		0,001	0,001
Q9C002	Normal mucosa of esophagus-specific gene 1 protein OS=Homo sapiens OX=9606 GN=NMES1 PE=1 SV=1		0,001	0,001
O60603	Toll-like receptor 2 OS=Homo sapiens OX=9606 GN=TLR2 PE=1 SV=1		0,001	0,001

(pink)	UPREGULATED
(light blue)	DOWNREGULATED
(grey)	NOT CHANGED

Normalised upregulated-downregulated proteins and graphs used in figures

	Unique peptide n≥2	Unique peptide n≥5	Regulated proteins
Regulated	2522	1173	281
Non-regulated	1132	796	1334
			348

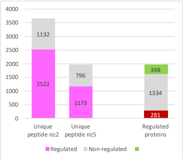

Tends to normalize	Normalised upregules	Normalised downreguled	Tends to normalize
P26022	P16671	O60814	P35659
Q99985	P02461	Q9BY44	Q9UM54
P08648	P07099	Q6PJT7	P27144
P06703	Q9NZN4	Q9UKM9	Q13428
O95340	O76070	P26368	Q8IVT2
P98082	Q99715	Q9Y3L3	P57735
P08473	P02452	Q9H773	A0A1Y0BRN3
P09467	P08123	Q96ST3	
Q14195	O14786	P55957	
P35052	P08779	Q13310	
P62736	P43121	P48047	
P19022	Q8IVF2	P27694	
P23634	P00352	Q15269	
Q05682	Q01995	O60264	
Q07954	P36551	O60841	
P80303	P39060	P82933	
Q07065	P09601	O75691	
Q969H8	Q9Y639	O43520	
Q8IVL6	O14975	Q96KP4	
Q7Z4H8	Q15758	Q9BW92	
P50281	Q16890	O75152	
Q96AY3	P43007	O75494	
O43852	P07996	O76031	
A0A669KBH5	P02533	Q9H0A0	
P42330	P09913	O15027	
	Q63ZY3	Q9UNX4	
	Q96JJ7	Q86U38	
	Q6UVK1	O00425	
	P50454	Q9Y3D9	
	P37840	P57088	
	Q9Y490	Q01780	
	P37059	O76021	
	Q9ULC3	P25205	
	Q9UHK6	P22087	
	P50416	Q9BYG3	
	P37235	Q16363	
	P69905	P62851	
	P09493	Q8IZL8	
	O60664	O60885	
	O00469	Q9NZL9	
	P14618	Q9H6E4	
	P18206	Q15050	
	O94925	O43143	
	P09525	Q9BVP2	
	Q09666	Q9NVI1	
	P30041	Q99848	
	Q6NYC8	Q9H7B2	
	Q8TD43	Q9UJS0	
	Q13620	Q07021	
	Q16706	Q08945	
	Q70UQ0	O95232	
	Q15942	Q8NI36	
	P05556	Q96GQ7	
	Q86TX2	Q9BZJ0	
	P06753	O43148	
	P18085	Q01105	
	Q9HAU0	Q9NVI7	
	P13674	Q9H6W3	
	Q99439	Q5JTH9	
	O60488	P18754	
	P60660	Q9H6R4	
	O15460	Q92841	
	P15559	Q14160	
	Q9BSJ8	Q9UKD2	
	P49023	Q9Y512	
	O14773	Q14807	
	P23743	Q14966	
	Q9P0K7	Q14137	
	Q13423	O75400	
	P55809	Q9UQ35	
	P09496	P35637	
	Q02809	O00541	
	Q6UW02	Q13618	
	P15121	P08621	
	Q96HC4	P82675	
	P46939	Q9UKV3	
	Q96CX2	Q14677	
	P35908	Q99661	
	P30622	Q5UIP0	
	P17655	Q16891	
	P49721	Q9NYF8	
	Q12797	Q14534	
	Q13438	Q13123	
	P00167	O75937	
	P21291	Q9BVJ6	
	Q9Y2B0	Q9NP81	
	O00291	Q9H583	
	Q9Y394	P53007	
	P07237	Q9GZT3	
	P35237	Q92900	
	P20073	P61604	
	P35613	Q9Y6M1	
	P35579	Q13823	
	P13645	P48449	
	P09914	Q14684	
	Q13501	O00567	
	P00387	Q13243	
	Q7Z5L9	Q9ULW0	
	P27816	Q9BZE4	
	P04040	Q9Y2X3	
	O14828	Q07955	
	Q6P1A2	P18858	
	O75340	O15498	
	P05026	P10809	
	P67936	P09622	
	O94979	Q9Y2W1	
	Q96IZ0	Q13268	
	Q16740	Q9H307	
	Q04446	Q14690	
	Q9H5V8	Q92747	
	P23381	P39748	
	Q14254	Q03701	
	Q9UBV2	Q9BQG0	
	P07686	Q9BXW7	
	P33527	Q00796	
	P00492	P61313	
	Q96TC7	Q9NWH9	
	Q9Y371	P06748	
	P27797	Q86XP3	
	Q99536	Q15154	
	P02768	P18583	
	P21397	P19338	
	P04264	Q86VM9	
	P51659	Q9Y399	
	P49589	P46782	
	Q13492	Q9NVP1	
	P55145	Q8TEQ6	
	P49720	P16401	
	P37837	Q02952	
	P11498	P11388	
	Q9H488	Q15223	
	Q92820	Q8IYB3	
	O00764	Q05639	
	O94973	P51649	
	P05166	P15311	
	P19367	Q9UBM7	
	Q9Y263	O43491	
	O14950	P26038	
	Q15075	Q8TEM1	
	P40763	Q9NU22	
	P23219	Q16352	
	A0AAG2UVC5	Q99988	
	A0A0J9YXF2	P50440	
	A0A087X1N8	P05141	
	E7EVA0	Q99614	
	A0A1B0GVI3	A0A669KB77	
	Q5JRA6	Q96SI9	
	P24844	P68431	
		P84243	

	Total	Normalised	Tends to normalize	Others
Upreguled	348	148	25	175
Downregule	281	149	7	125
		Normilized	Approach to Normality	Lock of normalization
	629	297	32	293

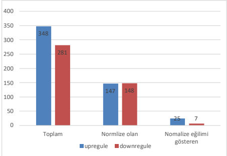

	HTB-5/SV-HUC	HTB-5 PR/SV-HUC
Lock of normalizatio	281	126
Approach to Normality		7
Normilzed		295
Approach to Normality		25
Lock of normalizatio	348	176

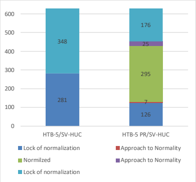

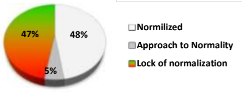

Supplement: Supplementary file 1 — Supplementary Material 1. Proteome data including the proteins with a minimum of 5 identified unique peptides. All regulated proteins (upregulated and downregulated) in the reprogrammed HTB-5 cells are listed. [file 12885_2026_15634_MOESM1_ESM.pdf]
