# Supplementary material for: Conjoining cell reprogramming and mass spectrometry to identify the proteomic variations in the reprogrammed bladder cancer cells: finding cues of normalisation
Source: BMC Cancer. 2026 Feb 6;26:338. doi: 10.1186/s12885-026-15634-x (PMC12977642; doi:10.1186/s12885-026-15634-x)
Supplement: Supplementary file 2 — Supplementary Material 2. Abundance values of proteins involved in pluripotency, stem cell differentiation and extracellular matrix used in hierarchical clustering. [file 12885_2026_15634_MOESM2_ESM.pdf]

Supplementary File 2

Conjoining cell reprogramming and mass spectrometry to identify the proteomic variations in the reprogrammed bladder cancer cells: Finding cues of normalisation

Banu Iskender[1,*], Mehmet Sarihan[1], Bengi Su Rumeysa Barlak[1], Gurler Akpinar[1], Murat Kasap[1]

[1] Kocaeli University Faculty of Medicine Department of Medical Biology Protein Research and

Proteomics Laboratory, Umuttepe, 41001, Izmit, Kocaeli-Turkey

*Corresponding Author: Kocaeli University Faculty of Medicine Department of Medical Biology Protein Research and Proteomics Laboratory, Umuttepe, 41001, Izmit, Kocaeli-Turkey banu.iskender@yahoo.com

Supplementary File 2 Abundance values of proteins involved in pluripotency, stem cell differentiation and extracellular matrix used in hierarchical clustering.

Abundance values used in hierarchical clustering (Extracellular matrix)

UNIQID	NAME	HTB-5 PR	HTB-5	SV-HUC-1
COL12A1	Collagen alpha-1(XII) chain OS=Homo sapiens OX=9606 GN=COL12A1 PE=1 SV=2	4591347	73263798	4173558
ITGB4	Integrin beta-4 OS=Homo sapiens OX=9606 GN=ITGB4 PE=1 SV=5	2420327	8363459	43934959
KRT1	Keratin, type II cytoskeletal 1 OS=Homo sapiens OX=9606 GN=KRT1 PE=1 SV=6	6,13E+08	8,9E+08	3,59E+08
HNRNPM	Heterogeneous nuclear ribonucleoprotein M OS=Homo sapiens OX=9606 GN=HNRNPM PE=1 SV=3	1,49E+08	1,26E+08	66144072
SMC3	Structural maintenance of chromosomes protein 3 OS=Homo sapiens OX=9606 GN=SMC3 PE=1 SV=2	32282810	22506814	31805205
ANXA6	Annexin A6 OS=Homo sapiens OX=9606 GN=ANXA6 PE=1 SV=3	1,12E+08	73929609	621924,6
COL1A1	Collagen alpha-1(I) chain OS=Homo sapiens OX=9606 GN=COL1A1 PE=1 SV=6	5111452	1,4E+08	94110,58
PKM	Pyruvate kinase PKM OS=Homo sapiens OX=9606 GN=PKM PE=1 SV=4	3,69E+08	7,88E+08	2,28E+08
FN1	Fibronectin OS=Homo sapiens OX=9606 GN=FN1 PE=1 SV=5	7481904	23087350	53110092
ITGA6	Integrin alpha-6 OS=Homo sapiens OX=9606 GN=ITGA6 PE=1 SV=5	5345271	10095313	18448033
ANXA2	Annexin A2 OS=Homo sapiens OX=9606 GN=ANXA2 PE=1 SV=2	1,33E+08	7,83E+08	4,08E+08
ANXA2	Annexin OS=Homo sapiens OX=9606 GN=ANXA2 PE=1 SV=2	1,31E+08	7,14E+08	3,94E+08
COL1A2	Collagen alpha-2(I) chain OS=Homo sapiens OX=9606 GN=COL1A2 PE=1 SV=7	1489253	72595529	4958899
SERPINH1	Serpin H1 OS=Homo sapiens OX=9606 GN=SERPINH1 PE=1 SV=2	2,34E+08	7,46E+08	1,5E+08
PLXNB2	Plexin-B2 OS=Homo sapiens OX=9606 GN=PLXNB2 PE=1 SV=3	4806227	11259299	14200175
HSP90AA1	Heat shock protein HSP 90-alpha OS=Homo sapiens OX=9606 GN=HSP90AA1 PE=1 SV=5	1,89E+08	86536903	1,02E+08
ANXA1	Annexin A1 OS=Homo sapiens OX=9606 GN=ANXA1 PE=1 SV=2	42157131	2,37E+08	1,36E+08
PLOD1	Procollagen-lysine,2-oxoglutarate 5-dioxygenase 1 OS=Homo sapiens OX=9606 GN=PLOD1 PE=1 SV=2	14122625	33599293	15328688
CALR	Calreticulin OS=Homo sapiens OX=9606 GN=CALR PE=1 SV=1	1,89E+08	4,04E+08	2,28E+08
PLOD2	Procollagen-lysine,2-oxoglutarate 5-dioxygenase 2 OS=Homo sapiens OX=9606 GN=PLOD2 PE=1 SV=2	7909583	78308072	27960481
LAD1	Ladinin-1 OS=Homo sapiens OX=9606 GN=LAD1 PE=1 SV=2	370866,2	769397,6	13633109
HCFC1	Host cell factor 1 OS=Homo sapiens OX=9606 GN=HCFC1 PE=1 SV=2	14504276	8198667	8050903
CSPG4	Chondroitin sulfate proteoglycan 4 OS=Homo sapiens OX=9606 GN=CSPG4 PE=1 SV=2	1666897	10706168	638981,2
CASK	Peripheral plasma membrane protein CASK OS=Homo sapiens OX=9606 GN=CASK PE=1 SV=3	4942337	10381763	4787304
LAMB1	Laminin subunit beta-1 OS=Homo sapiens OX=9606 GN=LAMB1 PE=1 SV=2	15641843	15922351	5619862
THBS1	Thrombospondin-1 OS=Homo sapiens OX=9606 GN=THBS1 PE=1 SV=2	804693,1	6455307	761951,8
LAMA3	Laminin subunit alpha-3 OS=Homo sapiens OX=9606 GN=LAMA3 PE=1 SV=3	1973216	3179458	13610608
RPSA	Small ribosomal subunit protein uS2 OS=Homo sapiens OX=9606 GN=RPSA PE=1 SV=4	93577196	51344688	84243966
COL3A1	Collagen alpha-1(III) chain OS=Homo sapiens OX=9606 GN=COL3A1 PE=1 SV=4	1316356	34879146	326421,9
LMAN1	Protein ERGIC-53 OS=Homo sapiens OX=9606 GN=LMAN1 PE=1 SV=2	25542495	31473305	25340573
ANXA5	Annexin A5 OS=Homo sapiens OX=9606 GN=ANXA5 PE=1 SV=2	64764610	76792810	23666196
TGFBI	Transforming growth factor-beta-induced protein ig-h3 OS=Homo sapiens OX=9606 GN=TGFBI PE=1 S	18611723	4449816	3410048
COL3A1	Collagen type III alpha 1 chain OS=Homo sapiens OX=9606 GN=COL3A1 PE=1 SV=1	3632336	38982703	3806095
ANXA4	Annexin A4 OS=Homo sapiens OX=9606 GN=ANXA4 PE=1 SV=4	2418490	17469320	4448722
DLG1	Disks large homolog 1 OS=Homo sapiens OX=9606 GN=DLG1 PE=1 SV=2	2868075	6393275	6985584
CTSD	Cathepsin D OS=Homo sapiens OX=9606 GN=CTSD PE=1 SV=1	26538142	44915860	95223283
HSD17B12	Very-long-chain 3-oxoacyl-CoA reductase OS=Homo sapiens OX=9606 GN=HSD17B12 PE=1 SV=2	15526429	14002103	18716984
ITGB1	Integrin beta-1 OS=Homo sapiens OX=9606 GN=ITGB1 PE=1 SV=2	30266230	1,95E+08	57634200
LAMB3	Laminin subunit beta-3 OS=Homo sapiens OX=9606 GN=LAMB3 PE=1 SV=1	169607,7	316608	3356465
PLOD3	Multifunctional procollagen lysine hydroxylase and glycosyltransferase LH3 OS=Homo sapiens OX=96	3349061	10783515	6430559
ANXA3	Annexin A3 OS=Homo sapiens OX=9606 GN=ANXA3 PE=1 SV=3	441377,7	13461969	27616654
TGM2	Protein-glutamine gamma-glutamyltransferase 2 OS=Homo sapiens OX=9606 GN=TGM2 PE=1 SV=2	1391787	7183656	9349020
ANXA11	Annexin A11 OS=Homo sapiens OX=9606 GN=ANXA11 PE=1 SV=1	4374115	14034181	9510141
GSTO1	Glutathione S-transferase omega-1 OS=Homo sapiens OX=9606 GN=GSTO1 PE=1 SV=2	19990424	16125491	10694194
COL18A1	Collagen alpha-1(XVIII) chain OS=Homo sapiens OX=9606 GN=COL18A1 PE=1 SV=5	1856971	10068331	941327,5
MMP14	Matrix metalloproteinase-14 OS=Homo sapiens OX=9606 GN=MMP14 PE=1 SV=3	7457858	19392031	2323321
LAD1	Ladinin-1 OS=Homo sapiens OX=9606 GN=LAD1 PE=1 SV=1	51974938	19803005	34294195
ACTA2	Actin, aortic smooth muscle OS=Homo sapiens OX=9606 GN=ACTA2 PE=1 SV=1	2,4E+08	4,38E+08	3,36E+08
P3H1	Prolyl 3-hydroxylase 1 OS=Homo sapiens OX=9606 GN=P3H1 PE=1 SV=2	12447930	18128825	1383509
LAMC1	Laminin subunit gamma-1 OS=Homo sapiens OX=9606 GN=LAMC1 PE=1 SV=3	8697430	10772643	2966574
LUM	Lumican OS=Homo sapiens OX=9606 GN=LUM PE=1 SV=2		15940968	
LGALS3	Galectin-3 OS=Homo sapiens OX=9606 GN=LGALS3 PE=1 SV=5	3656954	37718584	6733712
GPC1	Glypican-1 OS=Homo sapiens OX=9606 GN=GPC1 PE=1 SV=2	1464346	10849335	379000,7
COL5A2	Collagen alpha-2(V) chain OS=Homo sapiens OX=9606 GN=COL5A2 PE=1 SV=3	562790,3	5351295	402918
ALB	Albumin OS=Homo sapiens OX=9606 GN=ALB PE=1 SV=2	23257839	21047699	30849898
LAMA4	Laminin subunit alpha-4 OS=Homo sapiens OX=9606 GN=LAMA4 PE=1 SV=4	8469562	6053797	10724770
ANXA7	Annexin A7 OS=Homo sapiens OX=9606 GN=ANXA7 PE=1 SV=3	7019329	14171690	6819346
LAMA4	Laminin subunit alpha 4 OS=Homo sapiens OX=9606 GN=LAMA4 PE=1 SV=1	9635288	6261704	10096335
CLIC3	Chloride intracellular channel protein 3 OS=Homo sapiens OX=9606 GN=CLIC3 PE=1 SV=2		1420225	6483546

S100A6	Protein S100-A6 OS=Homo sapiens OX=9606 GN=S100A6 PE=1 SV=1	5034806	92846719	2517376
PTX3	Pentraxin-related protein PTX3 OS=Homo sapiens OX=9606 GN=PTX3 PE=1 SV=3	121965,1	690871,6	
SEMA3C	Semaphorin-3C OS=Homo sapiens OX=9606 GN=SEMA3C PE=2 SV=2	397932,9	1055090	
ADAM10	Disintegrin and metalloproteinase domain-containing protein 10 OS=Homo sapiens OX=9606 GN=AD	864731,5	4066401	5722180
GLG1	Golgi apparatus protein 1 OS=Homo sapiens OX=9606 GN=GLG1 PE=1 SV=2	4888328	4519455	5528250
GPC6	Glypican-6 OS=Homo sapiens OX=9606 GN=GPC6 PE=1 SV=1	5979459	4823860	164735,6
CTSB	Cathepsin B OS=Homo sapiens OX=9606 GN=CTSB PE=1 SV=3	7755469	5779314	3603143
CTSC	Dipeptidyl peptidase 1 OS=Homo sapiens OX=9606 GN=CTSC PE=1 SV=2	10818050	2479466	1689115
APOE	Apolipoprotein E OS=Homo sapiens OX=9606 GN=APOE PE=1 SV=1	3811413	911769,8	630119,7
F3	Tissue factor OS=Homo sapiens OX=9606 GN=F3 PE=1 SV=1		2302064	5509814
LGALS1	Galectin-1 OS=Homo sapiens OX=9606 GN=LGALS1 PE=1 SV=2	6595067	1,24E+08	1,28E+08
SPARC	SPARC OS=Homo sapiens OX=9606 GN=SPARC PE=1 SV=1	5171588	23202215	2749137
LAMB2	Laminin subunit beta-2 OS=Homo sapiens OX=9606 GN=LAMB2 PE=1 SV=2	160790,4	1173655	700456,5
FBLN1	Fibulin-1 OS=Homo sapiens OX=9606 GN=FBLN1 PE=1 SV=4	1485431	3682781	143842,8
CLU	Clusterin OS=Homo sapiens OX=9606 GN=CLU PE=1 SV=1	570283,8	3348970	205533,1
MFGE8	Lactadherin OS=Homo sapiens OX=9606 GN=MFGE8 PE=1 SV=3	2159963	1421872	170546,1
GPC4	Glypican-4 OS=Homo sapiens OX=9606 GN=GPC4 PE=1 SV=4	4011469	728614,4	62735,56
GPC2	Glypican-2 OS=Homo sapiens OX=9606 GN=GPC2 PE=1 SV=1	1828548	40373,7	155160,6
ALPL	Alkaline phosphatase, tissue-nonspecific isozyme OS=Homo sapiens OX=9606 GN=ALPL PE=1 SV=4	1557431	70794,45	
FBLN1	Fibulin-1 OS=Homo sapiens OX=9606 GN=FBLN1 PE=1 SV=1	1736669	3459174	110585,8
CTSZ	Cathepsin Z OS=Homo sapiens OX=9606 GN=CTSZ PE=1 SV=1	280857,6	2712425	17355656
S100A11	Protein S100-A11 OS=Homo sapiens OX=9606 GN=S100A11 PE=1 SV=2	10237756	25809850	26356401
S100A13	Protein S100-A13 OS=Homo sapiens OX=9606 GN=S100A13 PE=1 SV=1	3535932	8177156	5642835
DAG1	Dystroglycan 1 OS=Homo sapiens OX=9606 GN=DAG1 PE=1 SV=2	1059792	1320869	1357441
CSTB	Cystatin-B OS=Homo sapiens OX=9606 GN=CSTB PE=1 SV=2	21006487	20674623	6995225
COL6A3	Collagen alpha-3(VI) chain OS=Homo sapiens OX=9606 GN=COL6A3 PE=1 SV=5	706415,4	39619,41	
LAMC2	Laminin subunit gamma-2 OS=Homo sapiens OX=9606 GN=LAMC2 PE=1 SV=2	442775,1		1117199
LGALS9	Galectin-9 OS=Homo sapiens OX=9606 GN=LGALS9 PE=1 SV=2	1406862	4481096	1705543
MUC1	Mucin-1 OS=Homo sapiens OX=9606 GN=MUC1 PE=1 SV=3		2098993	450355,2
COL15A1	Collagen alpha-1(XV) chain OS=Homo sapiens OX=9606 GN=COL15A1 PE=1 SV=2		1069211	230659,1
SERPINE1	Plasminogen activator inhibitor 1 OS=Homo sapiens OX=9606 GN=SERPINE1 PE=1 SV=1		174638,3	485085
S100A10	Protein S100-A10 OS=Homo sapiens OX=9606 GN=S100A10 PE=1 SV=2	3626464	43490974	18593707
ECM1	Extracellular matrix protein 1 OS=Homo sapiens OX=9606 GN=ECM1 PE=1 SV=2	67554,95	1207719	317840,1
VASN	Vasorin OS=Homo sapiens OX=9606 GN=VASN PE=1 SV=1	228387,2	2062924	
SERPINE2	Glia-derived nexin OS=Homo sapiens OX=9606 GN=SERPINE2 PE=1 SV=1	111623,4	1426027	
BGN	Biglycan OS=Homo sapiens OX=9606 GN=BGN PE=1 SV=2	347084,8	1212725	
LAMA5	Laminin subunit alpha-5 OS=Homo sapiens OX=9606 GN=LAMA5 PE=1 SV=8	257946,1	338576	207829,4
COL6A1	Collagen alpha-1(VI) chain OS=Homo sapiens OX=9606 GN=COL6A1 PE=1 SV=3	764259,1	1263873	
TNC	Tenascin OS=Homo sapiens OX=9606 GN=TNC PE=1 SV=3	565836,8	711742,5	1185712
VCAN	Versican core protein OS=Homo sapiens OX=9606 GN=VCAN PE=1 SV=3	1514392	1131697	
FREM2	FRAS1-related extracellular matrix protein 2 OS=Homo sapiens OX=9606 GN=FREM2 PE=1 SV=3	393138,8		
PXDN	Peroxidasin homolog OS=Homo sapiens OX=9606 GN=PXDN PE=1 SV=2	20395,43		457252,7
GPC3	Glypican-3 OS=Homo sapiens OX=9606 GN=GPC3 PE=1 SV=1	579917,1		135744,2
AGRN	Agrin OS=Homo sapiens OX=9606 GN=AGRN PE=1 SV=1	94614,16	375723,2	233681,9
CTSL	Procathepsin L OS=Homo sapiens OX=9606 GN=CTSL PE=1 SV=2	28891,97	718593,6	156345,5
PXDN	Peroxidasin (Fragment) OS=Homo sapiens OX=9606 GN=PXDN PE=1 SV=1	20560,96		391836

Abundance values used in hierarchical clustering (Pluripotency)

UNIQID	NAME	HTB-5 PR	HTB-5	SV-HUC-1
SMPDL3B	Acid sphingomyelinase-like phosphodiesterase 3b OS=Homo sapiens OX=9606 GN=SMPDL3B PE=1 SV=2			1065869
ESRP1	Epithelial splicing regulatory protein 1 OS=Homo sapiens OX=9606 GN=ESRP1 PE=1 SV=2			326696,8
RPL22L1	Ribosomal protein eL22-like OS=Homo sapiens OX=9606 GN=RPL22L1 PE=1 SV=2		343575	161458,7
RRAS2	Ras-related protein R-Ras2 OS=Homo sapiens OX=9606 GN=RRAS2 PE=1 SV=1	1659851	8467336	6805688
MYO1E	Unconventional myosin-Ie OS=Homo sapiens OX=9606 GN=MYO1E PE=1 SV=2	117177,1	352219,3	1405264
MTHFD1L	Monofunctional C1-tetrahydrofolate synthase, mitochondrial OS=Homo sapiens OX=9606 GN=MTHFD1L PE=1 SV	5114754	9132392	11269534
FGF2	Fibroblast growth factor OS=Homo sapiens OX=9606 GN=FGF2 PE=1 SV=1	955343,2	1366987	922786
EEF1E1	Eukaryotic translation elongation factor 1 epsilon-1 OS=Homo sapiens OX=9606 GN=EEF1E1 PE=1 SV=1	1506302	2348653	3004335
MRPS30	Large ribosomal subunit protein mL65 OS=Homo sapiens OX=9606 GN=MRPS30 PE=1 SV=2	1090340	1381886	2384275
TOMM40	Mitochondrial import receptor subunit TOM40 homolog OS=Homo sapiens OX=9606 GN=TOMM40 PE=1 SV=1	12613454	11141944	22462865
FGF2	Fibroblast growth factor 2 OS=Homo sapiens OX=9606 GN=FGF2 PE=1 SV=3	1416367	1448440	108751,7
TFAM	Transcription factor A, mitochondrial OS=Homo sapiens OX=9606 GN=TFAM PE=1 SV=1	12791705	10125353	15378955
MTAP	S-methyl-5'-thioadenosine phosphorylase OS=Homo sapiens OX=9606 GN=MTAP PE=1 SV=2	3839063	3708397	1274066
PSME3	Proteasome activator complex subunit 3 OS=Homo sapiens OX=9606 GN=PSME3 PE=1 SV=1	13273136	10750654	11228233
NUP160	Nuclear pore complex protein Nup160 OS=Homo sapiens OX=9606 GN=NUP160 PE=1 SV=3	6360725	3507412	8275030
EMG1	Ribosomal RNA small subunit methyltransferase NEP1 OS=Homo sapiens OX=9606 GN=EMG1 PE=1 SV=4	2361371	1981098	3049544
LARP7	La-related protein 7 OS=Homo sapiens OX=9606 GN=LARP7 PE=1 SV=1	456442,6	372241,8	626430,4
G3BP2	Ras GTPase-activating protein-binding protein 2 OS=Homo sapiens OX=9606 GN=G3BP2 PE=1 SV=2	7328505	5496156	8175975
TMPO	Lamina-associated polypeptide 2, isoforms beta/gamma OS=Homo sapiens OX=9606 GN=TMPO PE=1 SV=2	37503534	22715372	29119987
DENR	Density-regulated protein OS=Homo sapiens OX=9606 GN=DENR PE=1 SV=2	1621397	1519944	1303902
SEPHS1	Selenide, water dikinase 1 OS=Homo sapiens OX=9606 GN=SEPHS1 PE=1 SV=2	1455911	871323,2	222135
TMPO	Lamina-associated polypeptide 2, isoform alpha OS=Homo sapiens OX=9606 GN=TMPO PE=1 SV=2	73957268	48816185	66408493
DDX21	Nucleolar RNA helicase 2 OS=Homo sapiens OX=9606 GN=DDX21 PE=1 SV=5	27812808	14649797	32608451
NOLC1	Nucleolar and coiled-body phosphoprotein 1 OS=Homo sapiens OX=9606 GN=NOLC1 PE=1 SV=2	1378394	1542148	3726220
DDX6	Probable ATP-dependent RNA helicase DDX6 OS=Homo sapiens OX=9606 GN=DDX6 PE=1 SV=2	18814505	8195262	11823901
PNO1	RNA-binding protein PNO1 OS=Homo sapiens OX=9606 GN=PNO1 PE=1 SV=1	580126,7	292600,5	1031336
HSPD1	60 kDa heat shock protein, mitochondrial OS=Homo sapiens OX=9606 GN=HSPD1 PE=1 SV=2	4,02E+08	1,7E+08	5,82E+08
RRM2	Ribonucleoside-diphosphate reductase subunit M2 OS=Homo sapiens OX=9606 GN=RRM2 PE=1 SV=1	8386515	2409392	2939579
NIP7	60S ribosome subunit biogenesis protein NIP7 homolog OS=Homo sapiens OX=9606 GN=NIP7 PE=1 SV=1	1176944	579686,8	278666,8
NLN	Neurolysin, mitochondrial OS=Homo sapiens OX=9606 GN=NLN PE=1 SV=1	6446006	2283641	2571439
SLIRP	SRA stem-loop-interacting RNA-binding protein, mitochondrial OS=Homo sapiens OX=9606 GN=SLIRP PE=1 SV=1	24573087	8813492	26049588
FKBP4	Peptidyl-prolyl cis-trans isomerase FKBP4 OS=Homo sapiens OX=9606 GN=FKBP4 PE=1 SV=3	37501032	10537582	11044548
DDX18	ATP-dependent RNA helicase DDX18 OS=Homo sapiens OX=9606 GN=DDX18 PE=1 SV=2	3505467	988946,3	4667663
MSH2	DNA mismatch repair protein Msh2 OS=Homo sapiens OX=9606 GN=MSH2 PE=1 SV=1	7548255	1717044	4732011
SNX5	Sorting nexin-5 OS=Homo sapiens OX=9606 GN=SNX5 PE=1 SV=1	5399382	1411921	1966326
TUBB2B	Tubulin beta-2B chain OS=Homo sapiens OX=9606 GN=TUBB2B PE=1 SV=1	8,38E+08	2,36E+08	1,17E+08
BCAT1	Branched-chain-amino-acid aminotransferase, cytosolic OS=Homo sapiens OX=9606 GN=BCAT1 PE=1 SV=3	7597979	1240836	533147,7
L1TD1	LINE-1 type transposase domain-containing protein 1 OS=Homo sapiens OX=9606 GN=L1TD1 PE=1 SV=1	34190289	1194786	776888,5
MDN1	Midasin OS=Homo sapiens OX=9606 GN=MDN1 PE=1 SV=2	1414904		403273,3

Abundance values used in hierarchical clustering (Stem Cell Differentiation)

UNIQID	NAME	HTB-5 PR	HTB-5	SV-HUC-1
FOXA1	Hepatocyte nuclear factor 3-alpha OS=Homo sapiens OX=9606 GN=FOXA1 PE=1 SV=2			
EPCAM	Epithelial cell adhesion molecule OS=Homo sapiens OX=9606 GN=EPCAM PE=1 SV=2	141334,3613	52504,17	8510465
A2M	Alpha-2-macroglobulin OS=Homo sapiens OX=9606 GN=A2M PE=1 SV=3		1318524	
NRP1	Neuropilin-1 OS=Homo sapiens OX=9606 GN=NRP1 PE=1 SV=3	107627,6806	5380070	135325,9
ITGB1	Integrin beta-1 OS=Homo sapiens OX=9606 GN=ITGB1 PE=1 SV=2	30266229,94	1,95E+08	57634200
KRT14	Keratin, type I cytoskeletal 14 OS=Homo sapiens OX=9606 GN=KRT14 PE=1 SV=4	15365623,54	63650182	15700189
TACSTD2	Tumor-associated calcium signal transducer 2 OS=Homo sapiens OX=9606 GN=TACSTD2 I	339007,1363	2265305	96757697
ENG	Endoglin OS=Homo sapiens OX=9606 GN=ENG PE=1 SV=2	595565,2044	2327094	1621499
SEMA3C	Semaphorin-3C OS=Homo sapiens OX=9606 GN=SEMA3C PE=2 SV=2	397932,8909	1055090	
FN1	Fibronectin OS=Homo sapiens OX=9606 GN=FN1 PE=1 SV=5	7481903,838	23087350	53110092
CDH2	Cadherin-2 OS=Homo sapiens OX=9606 GN=CDH2 PE=1 SV=4	2465161,62	9209150	454104,7
LAMA5	Laminin subunit alpha-5 OS=Homo sapiens OX=9606 GN=LAMA5 PE=1 SV=8	257946,1341	338576	207829,4
SLC9A1	Sodium/hydrogen exchanger 1 OS=Homo sapiens OX=9606 GN=SLC9A1 PE=1 SV=2	507669,9813	1223358	1545964
ALDH1A2	Retinal dehydrogenase 2 OS=Homo sapiens OX=9606 GN=ALDH1A2 PE=1 SV=3	3865297,985	8192787	4161122
KRT10	Keratin, type I cytoskeletal 10 OS=Homo sapiens OX=9606 GN=KRT10 PE=1 SV=6	190852038,6	3,94E+08	1,78E+08
GAK	Cyclin-G-associated kinase OS=Homo sapiens OX=9606 GN=GAK PE=1 SV=2	308533,0731	521332	355931,5
STAT3	Signal transducer and activator of transcription 3 OS=Homo sapiens OX=9606 GN=STAT3	3059619,249	5504609	4238649
KRT10	Keratin, type I cytoskeletal 10 OS=Homo sapiens OX=9606 GN=KRT10 PE=1 SV=2	225678706,1	4,03E+08	1,85E+08
MTCH2	Mitochondrial carrier homolog 2 OS=Homo sapiens OX=9606 GN=MTCH2 PE=1 SV=1	9337054,673	14476369	11799465
AP2A2	AP-2 complex subunit alpha-2 OS=Homo sapiens OX=9606 GN=AP2A2 PE=1 SV=2	1173552,275	2820390	784826,9
CDC42	Cell division control protein 42 homolog OS=Homo sapiens OX=9606 GN=CDC42 PE=1 SV=	4118006,873	6495950	7939428
FGF2	Fibroblast growth factor OS=Homo sapiens OX=9606 GN=FGF2 PE=1 SV=1	955343,1902	1366987	922786
UFL1	E3 UFM1-protein ligase 1 OS=Homo sapiens OX=9606 GN=UFL1 PE=1 SV=2	2122561,209	2127640	4550516
PDCD6	Programmed cell death protein 6 OS=Homo sapiens OX=9606 GN=PDCD6 PE=1 SV=1	10635458,38	12006659	7417390
CORO1C	Coronin-1C OS=Homo sapiens OX=9606 GN=CORO1C PE=1 SV=1	4707529,047	4658031	8192319
FGF2	Fibroblast growth factor 2 OS=Homo sapiens OX=9606 GN=FGF2 PE=1 SV=3	1416366,695	1448440	108751,7
MAPK14	Mitogen-activated protein kinase 14 OS=Homo sapiens OX=9606 GN=MAPK14 PE=1 SV=3	1064621,437	1288645	305555,8
HSPA9	Stress-70 protein, mitochondrial OS=Homo sapiens OX=9606 GN=HSPA9 PE=1 SV=2	192861432,9	2,19E+08	2,9E+08
CTNNB1	Catenin beta-1 OS=Homo sapiens OX=9606 GN=CTNNB1 PE=1 SV=1	15756033,17	16757092	36131193
MTA2	Metastasis-associated protein MTA2 OS=Homo sapiens OX=9606 GN=MTA2 PE=1 SV=1	11882607,58	9289870	15332431
PRKDC	DNA-dependent protein kinase catalytic subunit OS=Homo sapiens OX=9606 GN=PRKDC I	172274807,5	1,51E+08	1,21E+08
NELFB	Negative elongation factor B OS=Homo sapiens OX=9606 GN=NELFB PE=1 SV=1	2834553,478	932010,4	2180235
PUS7	Pseudouridylate synthase 7 homolog OS=Homo sapiens OX=9606 GN=PUS7 PE=1 SV=2	2199129,235	631463,5	1877811
MTA3	Metastasis-associated protein MTA3 OS=Homo sapiens OX=9606 GN=MTA3 PE=1 SV=2	1619995,897	1741091	3173561
MEN1	Menin OS=Homo sapiens OX=9606 GN=MEN1 PE=1 SV=5	586222,9291	481212,8	338107,9
NDUFS6	NADH dehydrogenase [ubiquinone] iron-sulfur protein 6, mitochondrial OS=Homo sapier	2632959,687	1676819	2033854
RBBP7	Histone-binding protein RBBP7 OS=Homo sapiens OX=9606 GN=RBBP7 PE=1 SV=1	36075360,98	18121414	23809414
MAPK1	Mitogen-activated protein kinase 1 OS=Homo sapiens OX=9606 GN=MAPK1 PE=1 SV=3	5529863,438	3805848	4014849
PWP1	Periodic tryptophan protein 1 homolog OS=Homo sapiens OX=9606 GN=PWP1 PE=1 SV=1	1463758,424	1291853	2036591
PSMD11	26S proteasome non-ATPase regulatory subunit 11 OS=Homo sapiens OX=9606 GN=PSM	35008755,37	23255596	18229718
PUM1	Pumilio homolog 1 OS=Homo sapiens OX=9606 GN=PUM1 PE=1 SV=3	4816602,534	1872420	4159452
NELFB	Negative elongation factor B OS=Homo sapiens OX=9606 GN=NELFB PE=1 SV=1	4276442,098	4994287	3741940
RPS7	Small ribosomal subunit protein eS7 OS=Homo sapiens OX=9606 GN=RPS7 PE=1 SV=1	41585921,87	31594054	34418477
DHX36	ATP-dependent DNA/RNA helicase DHX36 OS=Homo sapiens OX=9606 GN=DHX36 PE=1 S	1617868,798	1149358	1644063
HDAC1	Histone deacetylase 1 OS=Homo sapiens OX=9606 GN=HDAC1 PE=1 SV=1	3898452,001	2767621	3023027
HNRNPU	Heterogeneous nuclear ribonucleoprotein U OS=Homo sapiens OX=9606 GN=HNRNPU PE	293252350,3	1,91E+08	2,09E+08
CFL1	Cofilin-1 OS=Homo sapiens OX=9606 GN=CFL1 PE=1 SV=3	167608271,9	85257285	78290053
YAP1	Transcriptional coactivator YAP1 OS=Homo sapiens OX=9606 GN=YAP1 PE=1 SV=2	1807518,504	1055702	1133249
XRCC5	X-ray repair cross-complementing protein 5 OS=Homo sapiens OX=9606 GN=XRCC5 PE=1	85489832,21	54832155	51995971
NUDT21	Cleavage and polyadenylation specificity factor subunit 5 OS=Homo sapiens OX=9606 GN	19974345,12	12028450	17478989
GSK3B	Glycogen synthase kinase-3 beta OS=Homo sapiens OX=9606 GN=GSK3B PE=1 SV=2	1469127,178	882402,8	637014,2
YTHDF2	YTH domain-containing family protein 2 OS=Homo sapiens OX=9606 GN=YTHDF2 PE=1 SV	2881752,506	1727614	1208977
TFAP2A	Transcription factor AP-2-alpha OS=Homo sapiens OX=9606 GN=TFAP2A PE=1 SV=1	954077,6094	611561,4	10684469
GATAD2A	Transcriptional repressor p66-alpha OS=Homo sapiens OX=9606 GN=GATAD2A PE=1 SV=:	5262359,112	3575014	4161278
GATAD2B	Transcriptional repressor p66-beta OS=Homo sapiens OX=9606 GN=GATAD2B PE=1 SV=1	1892669,599	649873,3	1707376

NOLC1	Nucleolar and coiled-body phosphoprotein 1 OS=Homo sapiens OX=9606 GN=NOLC1 PE=	1378393,891	1542148	3726220
NSUN2	RNA cytosine C(5)-methyltransferase NSUN2 OS=Homo sapiens OX=9606 GN=NSUN2 PE=	21983355,14	8898256	9532540
RBBP4	Histone-binding protein RBBP4 OS=Homo sapiens OX=9606 GN=RBBP4 PE=1 SV=3	32844736,61	12769210	10328456
CHD4	Chromodomain-helicase-DNA-binding protein 4 OS=Homo sapiens OX=9606 GN=CHD4 PI	31003138,55	10029052	15968306
MTA1	Metastasis-associated protein MTA1 OS=Homo sapiens OX=9606 GN=MTA1 PE=1 SV=2	4432417,79	1168280	2192765
MBD3	Methyl-CpG-binding domain protein 3 OS=Homo sapiens OX=9606 GN=MBD3 PE=1 SV=1	3944468,508	1064903	2323067
TCOF1	Treacle protein OS=Homo sapiens OX=9606 GN=TCOF1 PE=1 SV=3	16565984,16	5166750	36545133
EFNB1	Ephrin-B1 OS=Homo sapiens OX=9606 GN=EFNB1 PE=1 SV=1	2986905,082	1153226	401505
PEF1	Peflin OS=Homo sapiens OX=9606 GN=PEF1 PE=1 SV=1	1304591,127	391018,2	495677,7
HDAC2	Histone deacetylase 2 OS=Homo sapiens OX=9606 GN=HDAC2 PE=1 SV=2	24681204,82	9079371	11227002
