# Supplementary material for: Conjoining cell reprogramming and mass spectrometry to identify the proteomic variations in the reprogrammed bladder cancer cells: finding cues of normalisation
Source: BMC Cancer. 2026 Feb 6;26:338. doi: 10.1186/s12885-026-15634-x (PMC12977642; doi:10.1186/s12885-026-15634-x)
Supplement: Supplementary file 3 — Supplementary Material 3. Abundance values of proteins involved in epithelial cell differentiation and neural development used in hierarchical clustering. [file 12885_2026_15634_MOESM3_ESM.pdf]

**Supplementary File 3**

**Conjoining cell reprogramming and mass spectrometry to identify the proteomic variations in the reprogrammed bladder cancer cells: Finding cues of normalisation**

Banu Iskender[1,*], Mehmet Sarihan[1], Bengi Su Rumeysa Barlak[1], Gurler Akpinar[1], Murat Kasap[1]

[1] Kocaeli University Faculty of Medicine Department of Medical Biology Protein Research and Proteomics Laboratory, Umuttepe, 41001, Izmit, Kocaeli-Turkey

*Corresponding Author: Kocaeli University Faculty of Medicine Department of Medical Biology Protein Research and Proteomics Laboratory, Umuttepe, 41001, Izmit, Kocaeli-Turkey banu.iskender@yahoo.com

**Supplementary File 3** Abundance values of proteins involved in epithelial cell differentiation and neural development used in hierarchical clustering.

**Abundance ratios used in hierarchical clustering (Epithelial cell differentiation)**

| UNIQID | NAME | HTB-5 PR | HTB-5 | SV-HUC-1 |
|---|---|---|---|---|
| O75369 | FLNB | 1,11E+08 | 4,37E+08 | 480328454 |
| Q15149 | PLEC | 47479186 | 1,58E+08 | 165116707 |
| P04083 | ANXA1 | 42157131 | 2,37E+08 | 135698673 |
| O60716 | CTNND1 | 12588413 | 35954459 | 77629425 |
| P49748 | ACADVL | 13535170 | 30615995 | 72225241 |
| P46940 | IQGAP1 | 18607554 | 96132519 | 71353105 |
| P42224 | STAT1 | 17928619 | 73363657 | 43292494 |
| Q9Y2Q3 | GSTK1 | 3575784 | 38710511 | 38564800 |
| Q16822 | PCK2 | 7510417 | 29762337 | 35053671 |
| P17301 | ITGA2 | 987991,4 | 64536013 | 34095142 |
| P05362 | ICAM1 | 977861 | 10875156 | 12611875 |
| O00592 | PODXL | 2794982 | 7694069 | 12405024 |
| Q16762 | TST | 2191566 | 5431586 | 11783887 |
| Q92520 | FAM3C | 5385857 | 8139007 | 11655520 |
| Q6YHK3 | CD109 | 754114,1 | 15454282 | 10849376 |
| Q92817 | EVPL | 395517,3 | 2561680 | 5257663,1 |
| Q6KB66 | KRT80 | 240744,5 | 1809433 | 2459048,5 |
| Q9Y5K6 | CD2AP | 1090874 | 2943323 | 2079677,6 |

**Abundance ratios used in hierarchical clustering (Neural development)**

| UNIQID | NAME | HTB-5 PR | HTB-5 | SV-HUC-1 |
|---|---|---|---|---|
| Q15813 | TBCE | 2254642 | 256827 | 465993,41 |
| Q8N158 | GPC2 | 1828548 | 40373,7 | 155160,59 |
| P07196 | NEFL | 30416482 | 1479326 | 26024,427 |
| O75110 | ATP9A | 168872,5 | | |
| Q0ZGT2 | NEXN | 24511,04 | 1286634 | |
| P07093 | SERPINE2 | 111623,4 | 1426027 | |
| Q99985 | SEMA3C | 397932,9 | 1055090 | |
| P17302 | GJA1 | 629236,6 | 1085956 | |
| P40424 | PBX1 | 1050051 | 818776,2 | |
| P36969 | GPX4 | 1123212 | 719081,3 | |
| Q03001 | DST | 781205,9 | 1085665 | |
| P15882 | CHN1 | 230680,8 | 28257,6 | |
| O60282 | KIF5C | 697941,7 | 26848,44 | |
| Q9BPU6 | DPYSL5 | 15706303 | 150501,2 | |
| P04216 | THY1 | 3823282 | 25819320 | 394580,73 |
| P98082 | DAB2 | 2574876 | 10425942 | 144261,63 |
| Q14195 | DPYSL3 | 11456348 | 50296053 | 2216617,8 |
| Q14194 | CRMP1 | 18958553 | 1050586 | 259510,65 |
| P19022 | CDH2 | 2465162 | 9209150 | 454104,68 |
| Q07954 | LRP1 | 6900570 | 19212610 | 981840 |
| Q01814 | ATP2B2 | 2938579 | 7071875 | 638735,41 |
| Q32P28 | P3H1 | 12447930 | 18128825 | 1383508,7 |
| P46821 | MAP1B | 1,23E+08 | 27340813 | 1936345,3 |
| Q16555 | DPYSL2 | 1,71E+08 | 57858150 | 22402491 |
| Q13418 | ILK | 2424478 | 3755566 | 644552,32 |
| A0A1C7CY | DPYSL2 | 1,66E+08 | 47175116 | 17989852 |
| Q9UL25 | RAB21 | 4262176 | 3590680 | 796344,61 |
| O75962 | TRIO | 878412,8 | 1274868 | 232950,24 |
| O94907 | DKK1 | 4364435 | 431266,7 | 33714,931 |
| Q13509 | TUBB3 | 43466043 | 7189986 | 1376677,7 |
| P09936 | UCHL1 | 31673785 | 13950756 | 2428715,7 |
| P36507 | MAP2K2 | 827648,2 | 617737,5 | 61542,31 |
| Q8N0X7 | SPART | 5271413 | 7288037 | 1552064,5 |
| Q13308 | PTK7 | 16299813 | 22595640 | 5434317 |
| P21333 | FLNA | 4,94E+08 | 7,27E+08 | 177284201 |
| O43847 | NRDC | 3138336 | 2134065 | 812742,59 |
| O75955 | FLOT1 | 12378351 | 17943268 | 4845662,5 |
| Q8N8S7 | ENAH | 4557554 | 3570897 | 1237584,3 |
| O60271 | SPAG9 | 4735557 | 5394839 | 1445847,9 |
| E9PFN4 | SLC4A7 | 1405711 | 1248839 | 362906,88 |
| O43175 | PHGDH | 1,41E+08 | 60026043 | 20803892 |
| A0A0U1RR | ENAH | 4022561 | 2411339 | 858598,8 |
| Q9BTW9 | TBCD | 7427083 | 12548866 | 3388855 |
| P11047 | LAMC1 | 8697430 | 10772643 | 2966573,7 |
| P31150 | GDI1 | 11432592 | 6173523 | 1944844,5 |
| O00429 | DNM1L | 5371306 | 3291631 | 1383529,5 |

| | | | | |
|---|---|---|---|---|
| Q14738 | PPP2R5D | 6988997 | 2898527 | 454077,37 |
| P07942 | LAMB1 | 15641843 | 15922351 | 5619862,2 |
| Q9NZI8 | IGF2BP1 | 88667920 | 10525286 | 5737997,1 |
| Q5T9L3 | WLS | 2705520 | 1583219 | 656455,27 |
| O60701 | UGDH | 21558724 | 22752187 | 11014734 |
| P29373 | CRABP2 | 27429065 | 680548,5 | 896462,12 |
| Q71U36 | TUBA1A | 1,43E+09 | 4,7E+08 | 216182703 |
| P63104 | YWHAZ | 2,49E+08 | 2,15E+08 | 103594597 |
| Q9UBN7 | HDAC6 | 1313177 | 863488,2 | 387799,13 |
| Q9UPN3 | MACF1 | 14002957 | 8027948 | 3414746,8 |
| O95155 | UBE4B | 953877 | 238788,3 | 120117,64 |
| P07197 | NEFM | 79890034 | 2620938 | 1780917,7 |
| P98172 | EFNB1 | 2986905 | 1153226 | 401505,02 |
| Q00535 | CDK5 | 627349 | 477362 | 287456,08 |
| P33176 | KIF5B | 18211528 | 14167685 | 8054712,6 |
| Q9NZD8 | SPG21 | 150201,8 | 585818,9 | 359452,64 |
| Q15019 | SEPTIN2 | 39284398 | 28063964 | 15501867 |
| Q93008 | USP9X | 1376527 | 864571,9 | 490916,63 |
| Q9BVA1 | TUBB2B | 8,38E+08 | 2,36E+08 | 116805192 |
| Q9UBB6 | NCDN | 698558,7 | 382486 | 172537,69 |
| O43251 | RBFOX2 | 4066273 | 2409085 | 1658710,5 |
| A0A8Q3W | RBFOX2 | 4793407 | 2406944 | 1297069,9 |
| Q13177 | PAK2 | 9606442 | 5449281 | 3484390,4 |
| P15531 | NME1 | 1,16E+08 | 74813634 | 63170345 |
| P13591 | NCAM1 | 4492993 | 265042 | 547692,31 |
| Q13409 | DYNC1I2 | 9006383 | 5080133 | 3510063,1 |
| P16949 | STMN1 | 1,08E+08 | 31267457 | 20703041 |
| P49841 | GSK3B | 1469127 | 882402,8 | 637014,15 |
| Q06124 | PTPN11 | 1674940 | 389735,7 | 395776,07 |
| P43034 | PAFAH1B1 | 8696371 | 4344095 | 3417137,3 |
| P46109 | CRKL | 3995627 | 2124059 | 1923251,7 |
| O60341 | KDM1A | 12619351 | 3485041 | 3079857,1 |
| A0A6Q8PF | PAFAH1B1 | 8935284 | 4055588 | 3516295,2 |
| P23528 | CFL1 | 1,68E+08 | 85257285 | 78290053 |
| Q16181 | SEPTIN7 | 35208075 | 15867446 | 14171423 |
| E7EPK1 | SEPTIN7 | 34064217 | 13472460 | 12526000 |
| Q9H2P0 | ADNP | 8951367 | 3475165 | 2548314,7 |
| Q92769 | HDAC2 | 24681205 | 9079371 | 11227002 |
| Q16643 | DBN1 | 88922989 | 29799652 | 25754371 |
| Q02790 | FKBP4 | 37501032 | 10537582 | 11044548 |
| P43487 | RANBP1 | 48529489 | 16160745 | 15517884 |
| P02649 | APOE | 3811413 | 911769,8 | 630119,69 |
| Q9UBB4 | ATXN10 | 6747962 | 2753672 | 2996980,9 |
| Q00169 | PITPNA | 2955892 | 671178,8 | 637245,85 |
| Q9NQX7 | ITM2C | 1271674 | 71150,97 | 239038,07 |
| P35580 | MYH10 | 48463589 | 10377125 | 14150195 |
| Q9UQ16 | DNM3 | 256811,5 | 77733,96 | 98989,519 |

| | | | | |
|---|---|---|---|---|
| P17677 | GAP43 | 2779406 | 9844,096 | 33321,406 |
| P08670 | VIM | 1,73E+08 | 2000019 | 4122648,5 |
| Q86U86 | PBRM1 | 1460999 | 343800,7 | 472705,53 |
| Q02880 | TOP2B | 14851776 | 4194355 | 6609798 |
| P11137 | MAP2 | 6822140 | 392893,1 | 606360,98 |
| A0A669KB | MAP2 | 8159750 | 1216681 | 4635054,4 |
| Q9NUM4 | TMEM106 | 1588732 | | 479100,91 |
