# Supplementary material for: Conjoining cell reprogramming and mass spectrometry to identify the proteomic variations in the reprogrammed bladder cancer cells: finding cues of normalisation
Source: BMC Cancer. 2026 Feb 6;26:338. doi: 10.1186/s12885-026-15634-x (PMC12977642; doi:10.1186/s12885-026-15634-x)
Supplement: Supplementary file 4 — Supplementary Material 4. Comparative expression analysis of the prospective biomarker candidates revealed by mass spectrometry during the reprogramming of bladder cancer cells with the expression profiles in multiple cancer types using the GEPIA tool. [file 12885_2026_15634_MOESM4_ESM.pdf]

Supplementary File 4

Conjoining cell reprogramming and mass spectrometry to identify the proteomic variations in the reprogrammed bladder cancer cells: Finding cues of normalisation

Banu Iskender[1,*], Mehmet Sarihan[1], Bengi Su Rumeysa Barlak[1], Gurler Akpinar[1], Murat Kasap[1]

[1] Kocaeli University Faculty of Medicine Department of Medical Biology Protein Research and Proteomics Laboratory, Umuttepe, 41001, Izmit, Kocaeli-Turkey

*Corresponding Author: Kocaeli University Faculty of Medicine Department of Medical Biology Protein Research and Proteomics Laboratory, Umuttepe, 41001, Izmit, Kocaeli-Turkey banu.iskender@yahoo.com

Supplementary File 4 Comparative expression analysis of the prospective biomarker candidates revealed by mass spectrometry during the reprogramming of bladder cancer cells with the expression profiles in multiple cancer types using GEPIA tool.

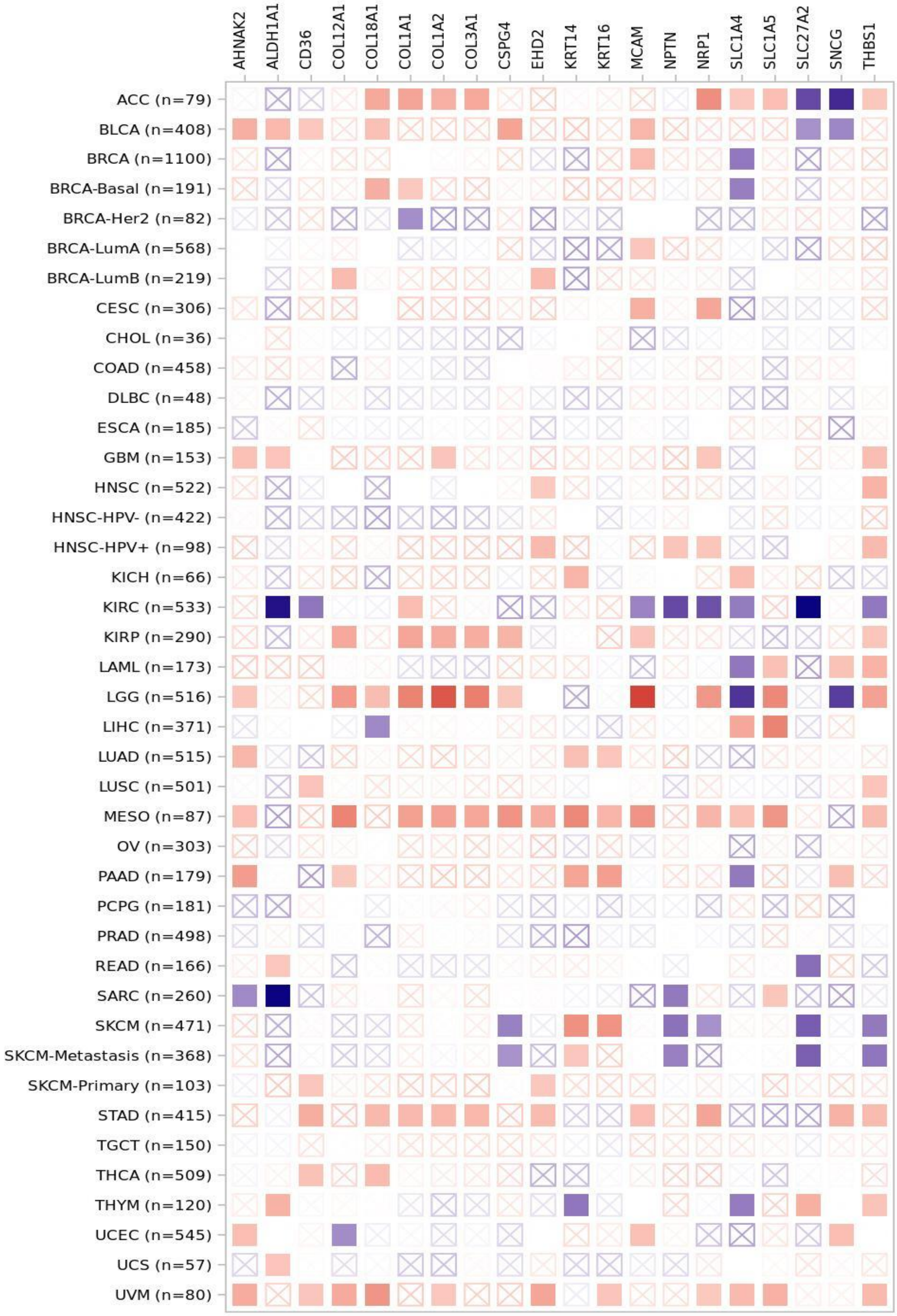
AHNAK2
ALDH1A1
CD36
COL12A1
COL18A1
COL1A1
COL1A2
COL3A1
CSPG4
EHD2
KRT14
KRT16
MCAM
NPTN
NRP1
SLC1A4
SLC1A5
SLC27A2
SNCG
THBS1
ACC (n=79)
BLCA (n=408)
BRCA (n=1100)
BRCA-Basal (n=191)
BRCA-Her2 (n=82)
BRCA-LumA (n=568)
BRCA-LumB (n=219)
CESC (n=306)
CHOL (n=36)
COAD (n=458)
DLBC (n=48)
ESCA (n=185)
GBM (n=153)
HNSC (n=522)
HNSC-HPV- (n=422)
HNSC-HPV+ (n=98)
KICH (n=66)
KIRC (n=533)
KIRP (n=290)
LAML (n=173)
LGG (n=516)
LIHC (n=371)
LUAD (n=515)
LUSC (n=501)
MESO (n=87)
OV (n=303)
PAAD (n=179)
PCPG (n=181)
PRAD (n=498)
READ (n=166)
SARC (n=260)
SKCM (n=471)
SKCM-Metastasis (n=368)
SKCM-Primary (n=103)
STAD (n=415)
TGCT (n=150)
THCA (n=509)
THYM (n=120)
UCEC (n=545)
UCS (n=57)
UVM (n=80)
